# Supplementary material for: In situ single-crystal synchrotron X-ray diffraction studies of biologically active gases in metal-organic frameworks
Source: Commun Chem. 2023 Mar 1;6:44. doi: 10.1038/s42004-023-00845-1 (PMC9977776; doi:10.1038/s42004-023-00845-1)
Supplement: Supplementary file 2 — Supplemental Information [file 42004_2023_845_MOESM2_ESM.pdf]

In Situ single-crystal synchrotron X-ray Diffraction Studies of Biologically Active Gases in Metal-Organic Frameworks

Supplementary Information

Russell M. Main, Simon M. Vornholt, Cameron M. Rice, Caroline Elliott, Samantha E. Russell, Peter J. Kerr, Mark R. Warren, and Russell E. Morris

1. Supplementary Methods

All samples were indexed with DIALS 3.6.2-g16e93f55b-release[1]. Visualisation was performed using Olex2 1.5 GUI[2] and ShelXT 2014/4[3] for solving and ShelXL 2018/3[4] for refinement.

For the dehydrated samples, the water bound oxygens were all constrained so the Uiso was of the same order as the metal to which it was bound, and were kept isotropic. This was to allow easy comparison of different samples without contribution from the pore environment.

For CO in Ni-CPO-27, the occupancies of C5 and O5 (the CO gas molecule) were set equal via a free variable. For the 300 K sample an EADP restraint was added for C5 and O6 (the water).

For CO in Co-4,6-dhip, the occupancies of C6 and O4 (the CO gas molecule) were set equal via a free variable. The C6 O4 bond length was restrained to 1.13 Å (the gas bond length [5]) via a DFIX restraint, the C6 Co1 bond length was restrained to 2.2 Å via a DFIX restraint and the O6A Co1 bond length was restrained to 2.09 Å (the Co-O bond length of the hydrated sample) via a DFIX restraint . Atoms Co1 O4 C6 (the CO bound to the metal) were subject to a SIMU restraint of deviation 0.08 and distance 3.7 Å. All these restraints were necessary due to the low CO occupancy.

For NO in Co-4,6-dhip it was necessary to restrain the N-O bond using a DFIX restraint. The bond lengths were restrained to 1.15 Å, the gas bond length.[6] To produce anisotropic Os it was necessary to use a SIMU restraint of deviation 0.02 and distance 2.7 Å between the Os and the N. A SUMP constraint was used on the O occupancies, with the sum total varied so that it matched the N occupancy to two decimal places.

In order to calculate free pore volume and electron density the Olex2 mask command was used.[7] Standard parameters (1.2 Å probe) were used. The mask was not used on the published structures, but as a calculation after refinement was finished. The total, theoretical, dehydrated pore volumes were calculated in Olex2 by removing any metal bound oxygen and calculating porosity with standard parameters. For Ni-CPO-27 this value was 2364.7 $Å^3$ / unit cell and for Co-4,6-dhip this was 2351.3 $Å^3$ / unit cell.

In order to calculate the number of gas molecules free in the pore from the observed electron densities the following calculation was performed. The activated e^- density was taken from the gas loaded e^- density to remove any input from disorder within the structure. This corrected value was divided by 14 to obtain the CO molecular density, then multiplied by the free pore volume to obtain the number of CO molecules per unit cell.

Supplementary Table 1: Containing information on temperature, loading and R_1 values for each dataset presented.

Sample	Temp / K	Loading	R_1	CCDC number
Ni-CPO-27 dehydrated	450	6.06(2) % water	9.19	2208832
Ni-CPO-27 CO loaded	450	38(3) % carbon CO	9.12	2208829
Ni-CPO-27 CO loaded	300	63(4) % CO<br>27(3) % water	9.68	2208833
Co-4,6-dhip dehydrated	500	9.0(4) % water	3.22	2208828
Co-4,6-dhip CO loaded	500	10.7(15) % CO<br>15.2(15) % water	3.09	2208827
Co-4,6-dhip dehydrated	450	7.7(4) % water	3.66	2208831
Co-4,6-dhip NO loaded	450	84.5(15) % NO	4.11	2208834
Co-4,6-dhip NO loaded	300	92(3) % NO	6.24	2208839

2. Supplementary Analysis

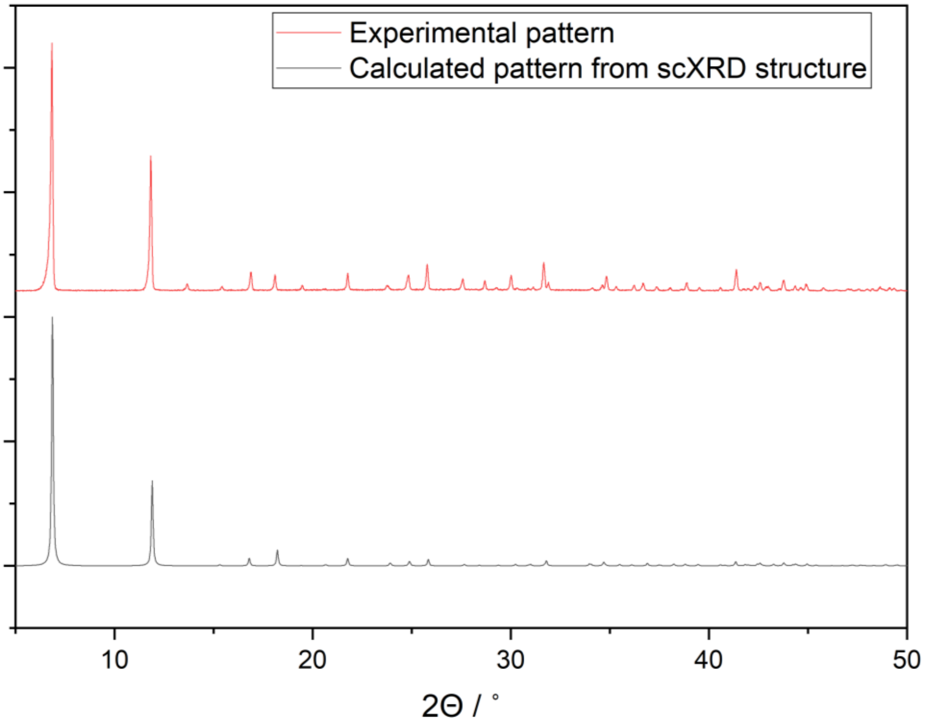

Figure S1: Powder X-ray diffraction patterns of Ni-CPO-27 showing: The as synthesised structure (red) and the calculated pattern from the dehydrated structure (black). Measured with Cu radiation (1.54 Å) on a STOE STADIP diffractometer.

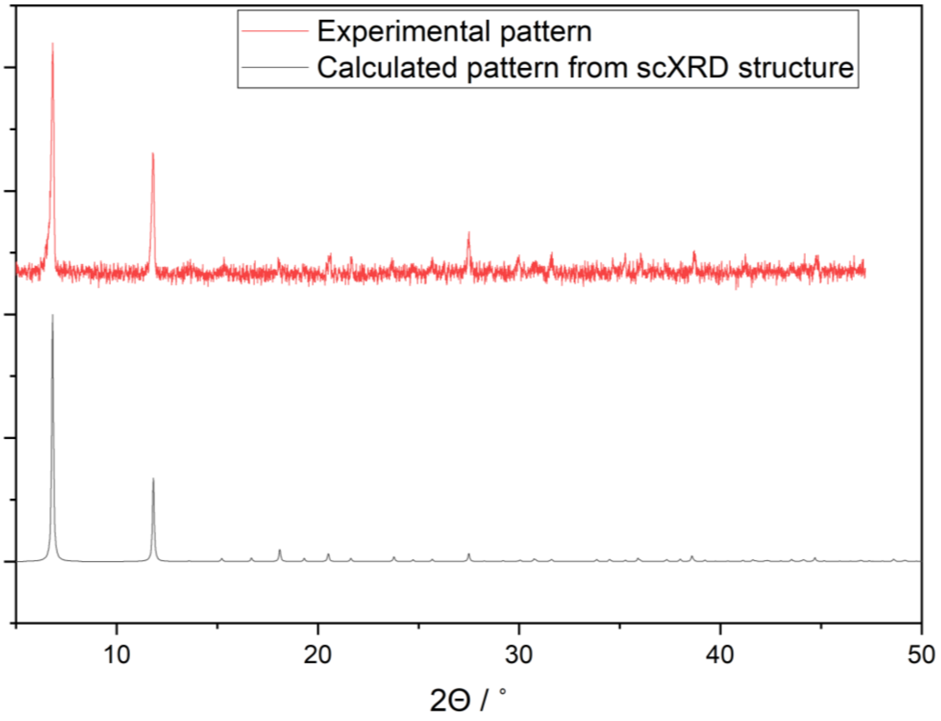

Figure S2: Powder X-ray diffraction patterns of Co-4,6-dhip showing: The as synthesised structure (red) and the calculated pattern from the dehydrated structure (black). Measured with Cu radiation (1.54 Å) on a STOE STADIP diffractometer.

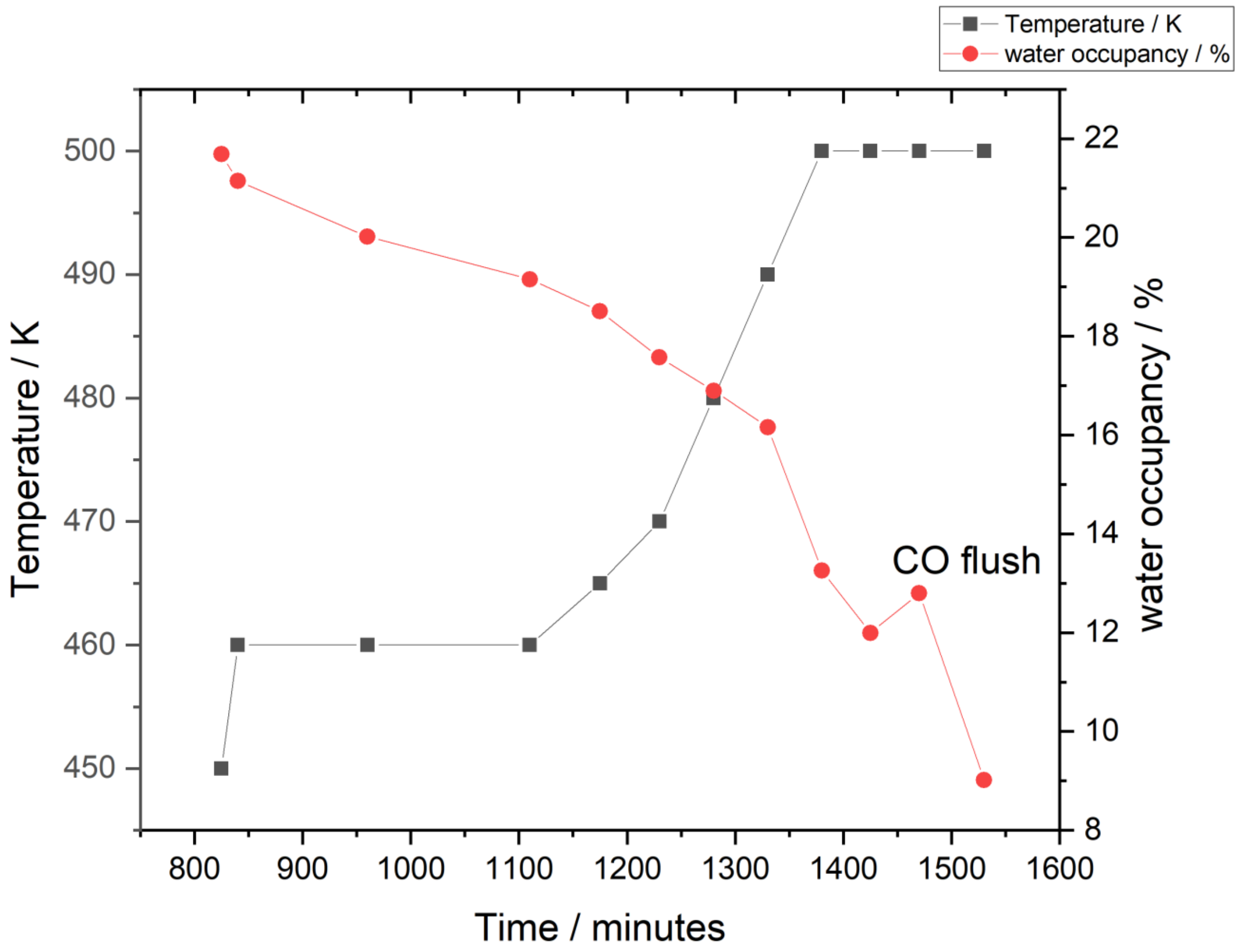

Figure S3: Chart showing the high temperature treatment required to dehydrate the Co-4,6-dhip crystal used for CO loading. The water atom (O4) was constrained as outlined in section 1.

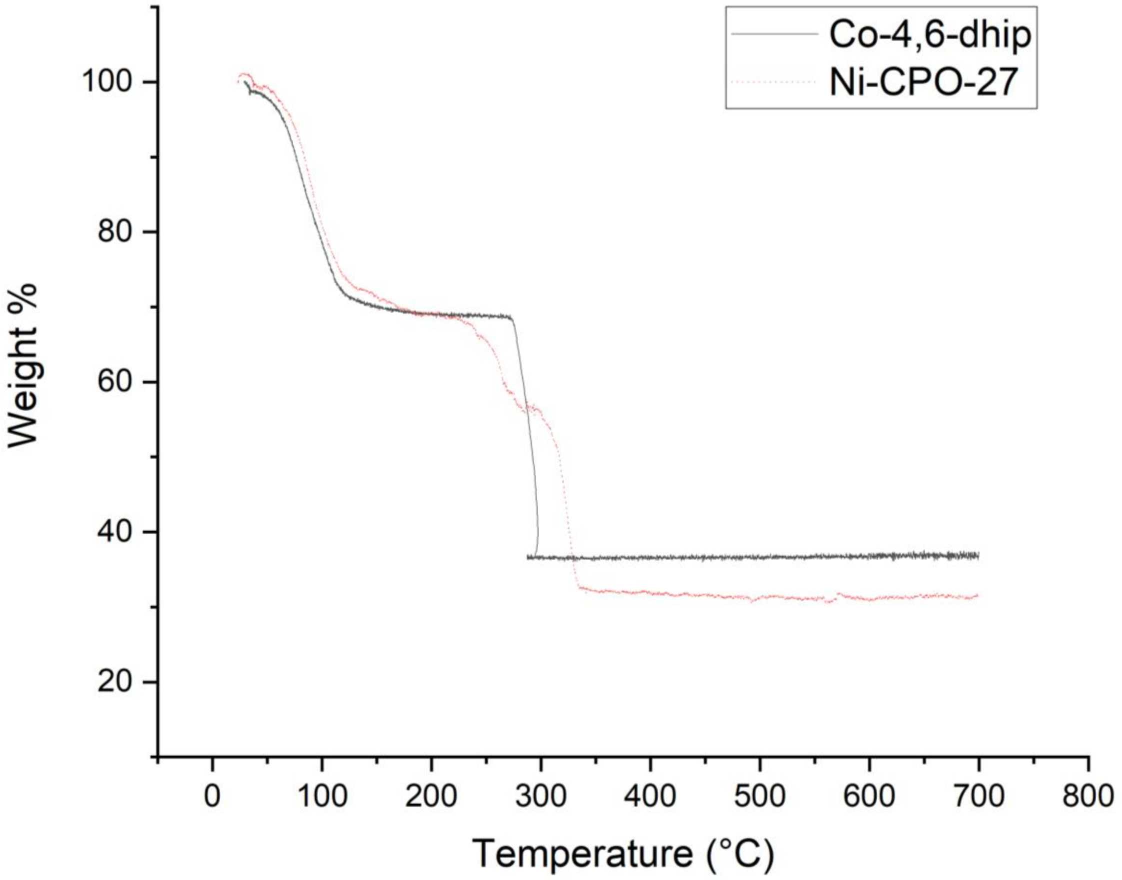

Figure S4: Thermal Gravimetric Analysis profiles of Ni-CPO-27 (red) and Co-4,6-dhip (black). Performed on a STA780 TG1000 at a heating rate of 5 ˚Cmin^{-1} in air.

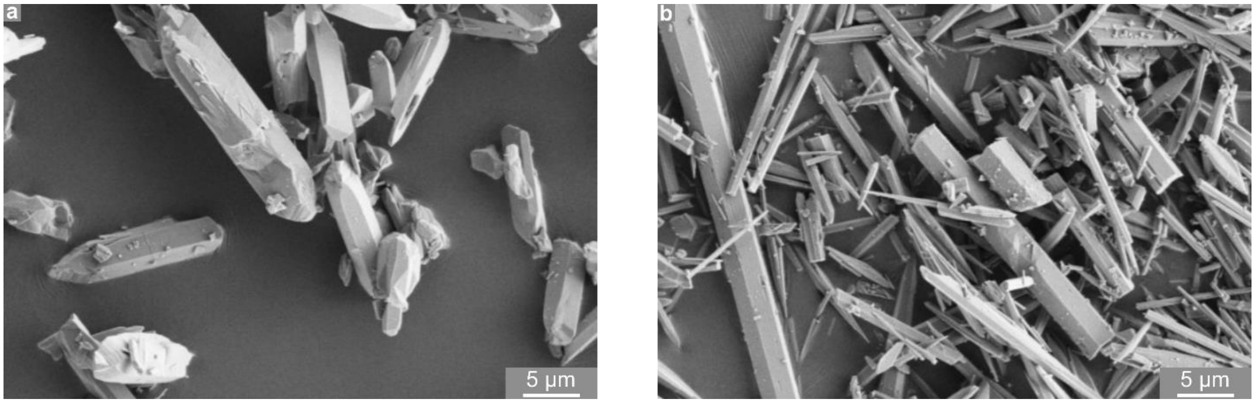

Figure S5:Scanning Electron Microscopy image of single crystals of Ni-CPO-27 (a) and Co-4,6-dhip (b) performed on a FEI Scios DualBeam Scanning Electron Microscope.

3. Supplementary References

1. Winter, G. *et al.* DIALS: Implementation and evaluation of a new integration package. *Acta Crystallogr. Sect. D Struct. Biol.* **74**, 85–97 (2018).

2. Dolomanov, O. V., Bourhis, L. J., Gildea, R. J., Howard, J. A. K. & Puschmann, H. OLEX2: A complete structure solution, refinement and analysis program. *J. Appl. Crystallogr.* **42**, 339–341 (2009).

3. Sheldrick, G. M. A short history of SHELX. *Acta Crystallogr. Sect. A Found. Crystallogr.* **64**, 112–122 (2008).

4. Sheldrick, G. M. Crystal structure refinement with SHELXL. *Acta Crystallogr. Sect. C Struct. Chem.* **71**, 3–8 (2015).

5. Demaison, J. & Császár, A. G. Equilibrium CO bond lengths. *J. Mol. Struct.* **1023**, 7–14 (2012).

6. Diatomic Spectral Database | NIST. https://www.nist.gov/pml/diatomic-spectral-database.

7. Rees, B., Jenner, L. & Yusupov, M. Bulk-solvent correction in large macromolecular structures. *urn:issn:0907-4449* **61**, 1299–1301 (2005).
