# Supplementary material for: In situ single-crystal synchrotron X-ray diffraction studies of biologically active gases in metal-organic frameworks
Source: Commun Chem. 2023 Mar 1;6:44. doi: 10.1038/s42004-023-00845-1 (PMC9977776; doi:10.1038/s42004-023-00845-1)
Supplement: Supplementary file 3 — Description of Additional Supplementary Files [file 42004_2023_845_MOESM3_ESM.pdf]

Description of Additional Supplementary Files

File name: Supplementary Data 1

Description: Details for structure 2208827

File name: Supplementary Data 2

Description: Details for structure 2208828

File name: Supplementary Data 3

Description: Details for structure 2208829

File name: Supplementary Data 4

Description: Details for structure 2208830

File name: Supplementary Data 5

Description: Details for structure 2208831

File name: Supplementary Data 6

Description: Details for structure 2208832

File name: Supplementary Data 7

Description: Details for structure 2208833

File name: Supplementary Data 8

Description: Details for structure 2208834
