# Supplementary material for: In situ single-crystal synchrotron X-ray diffraction studies of biologically active gases in metal-organic frameworks
Source: Commun Chem. 2023 Mar 1;6:44. doi: 10.1038/s42004-023-00845-1 (PMC9977776; doi:10.1038/s42004-023-00845-1)
Supplement: Supplementary file 7 — Supplementary Data 4 [file 42004_2023_845_MOESM7_ESM.pdf]

```
data_shelxt
_audit_creation_date              2022-06-08
_audit_creation_method
# start Validation Reply Form
_vrf_RINTA01_shelxt
;
PROBLEM: The value of Rint is greater than 0.25
RESPONSE: Experiment performed on needle crystal in a gas cell.
Diffraction from gas cell produces ring in pattern affecting the
intensities of some peaks. Difficulty was also found centring this
crystal due to its morphology
;
_vrf_PLAT026_shelxt
;
PROBLEM: Ratio Observed / Unique Reflections (too) Low ..        23%
Check
RESPONSE: Experiment performed on needle like crystal in a gas cell.
Leads to large background noise affecting 2sigma/I ratio. Actuall
number of peaks is still high
;
_vrf_PLAT602_shelxt
;
PROBLEM: Solvent Accessible VOID(S) in Structure ........          !
Check
RESPONSE: Expected in a metal organic framework
;
_vrf_PLAT043_shelxt
;
PROBLEM: Calculated and Reported Mol. Weight Differ by ..      36.42
Check
RESPONSE: Due to difference of calculated and reported Z

;
# end Validation Reply Form
;
Olex2 1.5
(compiled 2022.04.07 svn.rca3783a0 for OlexSys, GUI svn.r6498)
;
_shelx_SHELXL_version_number       '2018/3'
_audit_contact_author_address      ?
_audit_contact_author_email        ?
_audit_contact_author_name         ''
_audit_contact_author_phone        ?
_publ_contact_author_id_orcid      ?
_publ_section_references
;
Dolomanov, O.V., Bourhis, L.J., Gildea, R.J, Howard, J.A.K. &
Puschmann, H.
 (2009), J. Appl. Cryst. 42, 339-341.

Sheldrick, G.M. (2015). Acta Cryst. C71, 3-8.
;
_chemical_name_common              ?
_chemical_name_systematic          'Co-4,6-dhip NO loaded'
```

```
_chemical_formula_moiety          'C2 H0.5 Co0.5 N0.458 O1.955'
_chemical_formula_sum             'C2 H0.50 Co0.50 N0.51 O1.85'
_chemical_formula_weight          90.71
_chemical_absolute_configuration  ?
_chemical_melting_point           ?
loop_
  _atom_type_symbol
  _atom_type_description
  _atom_type_scat_dispersion_real
  _atom_type_scat_dispersion_imag
  _atom_type_scat_source
 'C' 'C' -0.0004 0.0007 'International Tables Vol C Tables 4.2.6.8
and 6.1.1.4'
 'H' 'H' 0.0000 0.0000 'International Tables Vol C Tables 4.2.6.8
and 6.1.1.4'
 'Co' 'Co' 0.2145 0.4840 'International Tables Vol C Tables 4.2.6.8
and 6.1.1.4'
 'N' 'N' -0.0004 0.0014 'International Tables Vol C Tables 4.2.6.8
and 6.1.1.4'
 'O' 'O' 0.0006 0.0026 'International Tables Vol C Tables 4.2.6.8
and 6.1.1.4'

_shelx_space_group_comment
;
The symmetry employed for this shelxl refinement is uniquely defined
by the following loop, which should always be used as a source of
symmetry information in preference to the above space-group names.
They are only intended as comments.
;
_space_group_crystal_system       'trigonal'
_space_group_IT_number            160
_space_group_name_H-M_alt         'R 3 m'
_space_group_name_Hall            'R 3 -2"'
loop_
  _space_group_symop_operation_xyz
 'x, y, z'
 '-y, x-y, z'
 '-x+y, -x, z'
 '-y, -x, z'
 '-x+y, y, z'
 'x, x-y, z'
 'x+2/3, y+1/3, z+1/3'
 '-y+2/3, x-y+1/3, z+1/3'
 '-x+y+2/3, -x+1/3, z+1/3'
 '-y+2/3, -x+1/3, z+1/3'
 '-x+y+2/3, y+1/3, z+1/3'
 'x+2/3, x-y+1/3, z+1/3'
 'x+1/3, y+2/3, z+2/3'
 '-y+1/3, x-y+2/3, z+2/3'
 '-x+y+1/3, -x+2/3, z+2/3'
 '-y+1/3, -x+2/3, z+2/3'
 '-x+y+1/3, y+2/3, z+2/3'
 'x+1/3, x-y+2/3, z+2/3'
```

```
_cell_length_a                    25.7854(6)
_cell_length_b                    25.7854(6)
_cell_length_c                    6.7749(2)
_cell_angle_alpha                 90
_cell_angle_beta                  90
_cell_angle_gamma                 120
_cell_volume                      3901.0(2)
_cell_formula_units_Z             36
_cell_measurement_reflns_used     1573
_cell_measurement_temperature     300
_cell_measurement_theta_max       15.21049808985503
_cell_measurement_theta_min       1.857732238412936
_shelx_estimated_absorpt_T_max    ?
_shelx_estimated_absorpt_T_min    ?
_exptl_absorpt_coefficient_mu     0.666
_exptl_absorpt_correction_T_max   1.0
_exptl_absorpt_correction_T_min   0.9761421411721107
_exptl_absorpt_correction_type    empirical
_exptl_absorpt_process_details
;
DIALS 3.6.2-g16e93f55b-release
Scaling & analysis of unmerged intensities, absorption correction
using spherical harmonics
;
_exptl_absorpt_special_details    ?
_exptl_crystal_colour             ?
_exptl_crystal_density_diffrn     1.390
_exptl_crystal_density_meas       ?
_exptl_crystal_density_method     ?
_exptl_crystal_description        needle
_exptl_crystal_F_000              1597
_exptl_crystal_size_max           0.15
_exptl_crystal_size_mid           0.01
_exptl_crystal_size_min           0.005
_exptl_transmission_factor_max    ?
_exptl_transmission_factor_min    ?
_diffrn_reflns_av_R_equivalents   0.4725
_diffrn_reflns_av_unetI/netI      0.5138
_diffrn_reflns_Laue_measured_fraction_full  0.995
_diffrn_reflns_Laue_measured_fraction_max  0.862
_diffrn_reflns_limit_h_max        40
_diffrn_reflns_limit_h_min        -40
_diffrn_reflns_limit_k_max        43
_diffrn_reflns_limit_k_min        -49
_diffrn_reflns_limit_l_max        13
_diffrn_reflns_limit_l_min        -10
_diffrn_reflns_number             36694
_diffrn_reflns_point_group_measured_fraction_full  0.995
_diffrn_reflns_point_group_measured_fraction_max  0.715
_diffrn_reflns_theta_full         16.950
_diffrn_reflns_theta_max          28.607
_diffrn_reflns_theta_min          1.870
_diffrn_ambient_temperature       300
_diffrn_detector_area_resol_mean  ?
```

```
_diffrn_measured_fraction_theta_full  0.995
_diffrn_measured_fraction_theta_max  0.862

_diffrn_measurement_specimen_support  'MiTeGen loop'
_diffrn_measurement_device_type    'Eiger 4M CdTe'
_diffrn_measurement_device         'Newport IS4CCD (4 circle)'
_diffrn_measurement_method         'phi scan'
_diffrn_radiation_source           'Diamond Light Source Beamline
I19'
_diffrn_radiation_monochromator    'double crystal Silicon 111'
_diffrn_radiation_type             'Synchrotron'
_diffrn_radiation_wavelength       0.4859
_diffrn_source                     ?
_reflns_Friedel_coverage           0.652
_reflns_Friedel_fraction_full      0.995
_reflns_Friedel_fraction_max       0.566
_reflns_number_gt                  1187
_reflns_number_total               5203
_reflns_special_details
;
 Reflections were merged by SHELXL according to the crystal
 class for the calculation of statistics and refinement.

 _reflns_Friedel_fraction is defined as the number of unique
 Friedel pairs measured divided by the number that would be
 possible theoretically, ignoring centric projections and
 systematic absences.
;
_reflns_threshold_expression       'I > 2\s(I)'
_computing_cell_refinement
;
DIALS 3.10.2-1-g02720afcd-release (Winter, G. et al., 2018)
XIA2 3.10.1 (Winter, G., 2010)
;
_computing_data_collection         'GDA 9.20; generic data
acquisition software'
_computing_data_reduction
;
DIALS 3.10.2-1-g02720afcd-release (Winter, G. et al., 2018)
dials.scale (Beilsten-Edmands, J. et al., 2020)
XIA2 3.10.1 (Winter, G., 2010)
;
_computing_molecular_graphics      'Olex2 1.5 (Dolomanov et al.,
2009)'
_computing_publication_material    'Olex2 1.5 (Dolomanov et al.,
2009)'
_computing_structure_refinement    'SHELXL 2018/3 (Sheldrick, 2015)'
_computing_structure_solution      'SHELXT 2018/2 (Sheldrick, 2018)'
_refine_diff_density_max           0.553
_refine_diff_density_min           -0.355
_refine_diff_density_rms           0.091
_refine_ls_abs_structure_details
;
 Flack x determined using 404 quotients [(I+)-(I-)]/[(I+)+(I-)]
```

```
 (Parsons, Flack and Wagner, Acta Cryst. B69 (2013) 249-259).
;
_refine_ls_abs_structure_Flack     -0.04(8)
_refine_ls_extinction_coef         .
_refine_ls_extinction_method       none
_refine_ls_goodness_of_fit_ref     0.704
_refine_ls_hydrogen_treatment      constr
_refine_ls_matrix_type             full
_refine_ls_number_parameters       121
_refine_ls_number_reflns           5203
_refine_ls_number_restraints       66
_refine_ls_R_factor_all            0.3118
_refine_ls_R_factor_gt             0.0624
_refine_ls_restrained_S_all        0.702
_refine_ls_shift/su_max            0.000
_refine_ls_shift/su_mean           0.000
_refine_ls_structure_factor_coef   Fsqd
_refine_ls_weighting_details
 'w=1/[\s^2^(Fo^2^)+(0.0313P)^2^] where P=(Fo^2^+2Fc^2^)/3'
_refine_ls_weighting_scheme        calc
_refine_ls_wR_factor_gt            0.1027
_refine_ls_wR_factor_ref           0.1452
_refine_special_details            ?
_olex2_refinement_description
;
1. Fixed Uiso
 At 1.2 times of:
  All C(H) groups
2. Restrained distances
 O4C-N1 = O4D-N1 = O4A-N1 = O4B-N1
 1.15 with sigma of 0.02
3. Uiso/Uaniso restraints and constraints
O4C \\sim O4B \\sim O4A \\sim O4D \\sim N1: within 2.7A with sigma
of 0.02 and
sigma for terminal atoms of 0.04 within 2.7A
4. Others
 1*[Sof(O4A)]+1*[Sof(O4B)]+1*[Sof(O4C)]+1*[Sof(O4D)]=0.91 with esd
of 0.001
5.a Aromatic/amide H refined with riding coordinates:
 C5(H5), C4(H4)
;
_atom_sites_solution_hydrogens     geom
_atom_sites_solution_primary       ?
_atom_sites_solution_secondary     ?
loop_
  _atom_site_label
  _atom_site_type_symbol
  _atom_site_fract_x
  _atom_site_fract_y
  _atom_site_fract_z
  _atom_site_U_iso_or_equiv
  _atom_site_adp_type
  _atom_site_occupancy
  _atom_site_site_symmetry_order
```

```
  _atom_site_calc_flag
  _atom_site_refinement_flags_posn
  _atom_site_refinement_flags_adp
  _atom_site_refinement_flags_occupancy
  _atom_site_disorder_assembly
  _atom_site_disorder_group
 Co1 Co 0.94990(5) 0.62434(5) 0.14461(13) 0.0374(3) Uani 1 1
d . . . . .
 O1 O 0.8953(2) 0.6125(3) 0.3687(6) 0.0430(15) Uani 1 1 d . . . . .
 O3 O 0.9276(3) 0.6732(3) 0.9607(9) 0.0397(14) Uani 1 1 d . . . . .
 O2 O 0.9628(2) 0.6503(2) 0.6066(6) 0.0390(15) Uani 1 1 d . . . . .
 N1 N 0.8919(5) 0.5490(5) 0.0299(13) 0.054(4) Uani 0.92(3) 1 d D
U . . .
 C5 C 0.8329(3) 0.6658(6) 0.9585(18) 0.041(3) Uani 1 2 d S T P . .
 H5 H 0.837494 0.674989 1.092428 0.050 Uiso 1 2 calc R U P . .
 C1 C 0.9109(4) 0.6331(3) 0.5395(9) 0.036(2) Uani 1 1 d . . . . .
 C2 C 0.8677(4) 0.6408(4) 0.6592(9) 0.038(2) Uani 1 1 d . . . . .
 C3 C 0.8764(3) 0.6595(3) 0.8659(10) 0.0339(19) Uani 1 1 d . . . . .
 C4 C 0.8155(3) 0.6310(5) 0.5716(14) 0.039(3) Uani 1 2 d S T P . .
 H4 H 0.808363 0.616726 0.442690 0.047 Uiso 1 2 calc R U P . .
 O4B O 0.8638(16) 0.5033(11) 0.088(4) 0.092(11) Uani 0.31(2) 1 d D U
P A 2
 O4C O 0.888(3) 0.519(3) -0.101(7) 0.092(17) Uani 0.131(16) 1 d D U
P A 3
 O4A O 0.8428(10) 0.5287(13) 0.034(5) 0.084(9) Uani 0.37(3) 1 d D U
P A 1
 O4D O 0.854(3) 0.534(4) -0.079(13) 0.068(14) Uiso 0.10(2) 1 d D U P
A 4

loop_
  _atom_site_aniso_label
  _atom_site_aniso_U_11
  _atom_site_aniso_U_22
  _atom_site_aniso_U_33
  _atom_site_aniso_U_23
  _atom_site_aniso_U_13
  _atom_site_aniso_U_12
 Co1 0.0444(7) 0.0441(7) 0.0228(3) -0.0004(5) 0.0008(6) 0.0213(6)
 O1 0.041(3) 0.065(4) 0.022(2) -0.017(2) -0.002(2) 0.026(3)
 O3 0.040(4) 0.055(4) 0.028(3) -0.008(2) 0.002(3) 0.027(3)
 O2 0.045(4) 0.050(4) 0.024(3) -0.008(2) -0.005(2) 0.025(3)
 N1 0.051(8) 0.048(7) 0.053(5) -0.002(4) -0.003(5) 0.018(6)
 C5 0.044(5) 0.057(8) 0.027(5) -0.016(5) -0.008(2) 0.029(4)
 C1 0.038(5) 0.042(5) 0.022(3) 0.002(3) 0.000(3) 0.016(4)
 C2 0.049(5) 0.045(6) 0.022(3) -0.003(3) -0.002(3) 0.026(5)
 C3 0.035(5) 0.033(5) 0.030(3) 0.001(3) -0.001(3) 0.015(4)
 C4 0.042(5) 0.057(8) 0.024(4) -0.005(5) -0.003(2) 0.028(4)
 O4B 0.089(17) 0.071(17) 0.084(16) -0.007(13) -0.049(14) 0.016(15)
 O4C 0.06(2) 0.09(2) 0.10(2) -0.01(2) -0.01(2) 0.02(2)
 O4A 0.074(16) 0.074(15) 0.086(18) -0.009(13) -0.023(12) 0.023(13)

_geom_special_details
;
 All esds (except the esd in the dihedral angle between two l.s.
```

```
planes)
 are estimated using the full covariance matrix.  The cell esds are
taken
 into account individually in the estimation of esds in distances,
angles
 and torsion angles; correlations between esds in cell parameters
are only
 used when they are defined by crystal symmetry.  An approximate
(isotropic)
 treatment of cell esds is used for estimating esds involving l.s.
planes.
;
loop_
  _geom_bond_atom_site_label_1
  _geom_bond_atom_site_label_2
  _geom_bond_distance
  _geom_bond_site_symmetry_2
  _geom_bond_publ_flag
 Co1 O1 1.987(5) . ?
 Co1 O3 2.059(6) 8_654 ?
 Co1 O3 2.048(6) 1_554 ?
 Co1 O2 2.054(5) 8_654 ?
 Co1 O2 2.134(5) 15_664 ?
 Co1 N1 1.925(10) . ?
 O1 C1 1.253(8) . ?
 O3 C3 1.348(9) . ?
 O2 C1 1.266(8) . ?
 N1 O4B 1.10(2) . ?
 N1 O4C 1.14(2) . ?
 N1 O4A 1.102(18) . ?
 N1 O4D 1.14(3) . ?
 C5 H5 0.9300 . ?
 C5 C3 1.363(9) 5_655 ?
 C5 C3 1.363(9) . ?
 C1 C2 1.469(10) . ?
 C2 C3 1.461(9) . ?
 C2 C4 1.374(8) . ?
 C4 H4 0.9300 . ?

loop_
  _geom_angle_atom_site_label_1
  _geom_angle_atom_site_label_2
  _geom_angle_atom_site_label_3
  _geom_angle
  _geom_angle_site_symmetry_1
  _geom_angle_site_symmetry_3
  _geom_angle_publ_flag
 O1 Co1 O3 99.5(2) . 1_554 ?
 O1 Co1 O3 93.5(2) . 8_654 ?
 O1 Co1 O2 171.4(2) . 8_654 ?
 O1 Co1 O2 92.09(19) . 15_664 ?
 O3 Co1 O3 165.1(2) 1_554 8_654 ?
 O3 Co1 O2 91.5(3) 1_554 15_664 ?
 O3 Co1 O2 82.9(2) 1_554 8_654 ?
```

```
 O3 Co1 O2 80.7(2) 8_654 15_664 ?
 O2 Co1 O3 83.3(2) 8_654 8_654 ?
 O2 Co1 O2 79.50(6) 8_654 15_664 ?
 N1 Co1 O1 91.1(4) . . ?
 N1 Co1 O3 93.4(4) . 1_554 ?
 N1 Co1 O3 93.6(3) . 8_654 ?
 N1 Co1 O2 97.1(3) . 8_654 ?
 N1 Co1 O2 173.6(4) . 15_664 ?
 C1 O1 Co1 126.0(5) . . ?
 Co1 O3 Co1 96.9(2) 1_556 15_665 ?
 C3 O3 Co1 131.9(6) . 1_556 ?
 C3 O3 Co1 119.6(5) . 15_665 ?
 Co1 O2 Co1 94.4(2) 15_665 8_655 ?
 C1 O2 Co1 126.2(5) . 15_665 ?
 C1 O2 Co1 133.0(4) . 8_655 ?
 O4B N1 Co1 134.3(16) . . ?
 O4C N1 Co1 138(3) . . ?
 O4A N1 Co1 126.6(18) . . ?
 O4D N1 Co1 135(5) . . ?
 C3 C5 H5 118.1 . . ?
 C3 C5 H5 118.1 5_655 . ?
 C3 C5 C3 123.7(10) . 5_655 ?
 O1 C1 O2 122.6(7) . . ?
 O1 C1 C2 117.6(7) . . ?
 O2 C1 C2 119.7(6) . . ?
 C3 C2 C1 124.9(7) . . ?
 C4 C2 C1 118.4(6) . . ?
 C4 C2 C3 116.6(7) . . ?
 O3 C3 C5 120.4(7) . . ?
 O3 C3 C2 120.8(7) . . ?
 C5 C3 C2 118.8(8) . . ?
 C2 C4 C2 125.2(9) . 5_655 ?
 C2 C4 H4 117.4 . . ?
 C2 C4 H4 117.4 5_655 . ?

loop_
  _geom_torsion_atom_site_label_1
  _geom_torsion_atom_site_label_2
  _geom_torsion_atom_site_label_3
  _geom_torsion_atom_site_label_4
  _geom_torsion
  _geom_torsion_site_symmetry_1
  _geom_torsion_site_symmetry_2
  _geom_torsion_site_symmetry_3
  _geom_torsion_site_symmetry_4
  _geom_torsion_publ_flag
 Co1 O1 C1 O2 -20.1(11) . . . . ?
 Co1 O1 C1 C2 156.0(5) . . . . ?
 Co1 O3 C3 C5 -139.7(8) 15_665 . . . ?
 Co1 O3 C3 C5 86.1(11) 1_556 . . . ?
 Co1 O3 C3 C2 -97.4(9) 1_556 . . . ?
 Co1 O3 C3 C2 36.9(9) 15_665 . . . ?
 Co1 O2 C1 O1 5.2(12) 8_655 . . . ?
 Co1 O2 C1 O1 149.7(6) 15_665 . . . ?
```

```
 Co1 O2 C1 C2 -170.8(5) 8_655 . . . ?
 Co1 O2 C1 C2 -26.3(9) 15_665 . . . ?
 O1 C1 C2 C3 174.7(8) . . . . ?
 O1 C1 C2 C4 -6.7(12) . . . . ?
 O2 C1 C2 C3 -9.1(12) . . . . ?
 O2 C1 C2 C4 169.5(8) . . . . ?
 C1 C2 C3 O3 2.2(12) . . . . ?
 C1 C2 C3 C5 178.9(10) . . . . ?
 C1 C2 C4 C2 -174.8(8) . . . 5_655 ?
 C3 C5 C3 O3 172.2(9) 5_655 . . . ?
 C3 C5 C3 C2 -4.4(19) 5_655 . . . ?
 C3 C2 C4 C2 3.9(17) . . . 5_655 ?
 C4 C2 C3 O3 -176.3(8) . . . . ?
 C4 C2 C3 C5 0.3(13) . . . . ?

_mask_special_details
;
unused in refinement
_smtbx_masks_special_details       unused in refinement
loop_
  _smtbx_masks_void_nr
  _smtbx_masks_void_average_x
  _smtbx_masks_void_average_y
  _smtbx_masks_void_average_z
  _smtbx_masks_void_volume
  _smtbx_masks_void_count_electrons
  _smtbx_masks_void_content
 1 0.000 0.000 -0.867 473.4 314.3 ?
 2 0.333 0.667 -0.298 473.4 314.3 ?
 3 0.667 0.333 -0.631 473.4 314.3 ?

_smtbx_masks_void_probe_radius     1.2
_smtbx_masks_void_truncation_radius  1.2
;

_shelx_res_file
;
TITL shelxt_a.res in R3m
    shelxt.res
    created by SHELXL-2018/3 at 14:28:55 on 08-Jun-2022
REM Old TITL shelxt_a.res in R3m
REM SHELXT solution in R3m: R1 0.153, Rweak 0.049, Alpha 0.003
REM <I/s> 0.000 for 0 systematic absences, Orientation as input
REM Flack x = 0.112 ( 0.053 ) from 414 Parsons' quotients
REM Formula found by SHELXT: C23 N13 O18 Co6
CELL 0.4859 25.7854 25.7854 6.7749 90 90 120
ZERR 36 0.0006 0.0006 0.0002 0 0 0
LATT -3
SYMM -Y,+X-Y,+Z
SYMM +Y-X,-X,+Z
SYMM -Y,-X,+Z
SYMM +Y-X,+Y,+Z
SYMM +X,+X-Y,+Z
SFAC C H Co N O
```

```
DISP C -0.0004 0.0007 6.0677
DISP Co 0.2145 0.484 1359.6524
DISP H 0 0 0.6065
DISP N -0.0004 0.0014 8.9832
DISP O 0.0006 0.0026 13.3363
UNIT 72 18 18 18.31 66.59
DFIX 1.15 O4C N1 O4D N1 O4A N1 O4B N1
SIMU 0.02 0.04 2.7 O4C O4B O4A O4D N1
SUMP 0.91 0.001 1 4 1 3 1 2 1 5

L.S. 20
PLAN  16
CONF
BOND
list 4
MORE -1
BOND $H
fmap 2 53
acta
OMIT -1 2 0
OMIT 0 5 1
OMIT -5 5 -1
OMIT -2 4 0
OMIT 0 1 2
OMIT -1 1 -2
OMIT 0 1 -1
REM <olex2.extras>
REM <HklSrc "%.\\shelxt.hkl">
REM </olex2.extras>

WGHT    0.031300
FVAR       0.18870   0.13120   0.30870   0.36846   0.10140
CO1   3    0.949902    0.624338    0.144610    11.00000    0.04436
0.04412 =
         0.02278   -0.00042    0.00079    0.02130
O1    5    0.895325    0.612452    0.368667    11.00000    0.04057
0.06520 =
         0.02204   -0.01653   -0.00173    0.02562
O3    5    0.927607    0.673195    0.960743    11.00000    0.03967
0.05519 =
         0.02791   -0.00756    0.00180    0.02656
O2    5    0.962822    0.650264    0.606627    11.00000    0.04474
0.04977 =
         0.02426   -0.00847   -0.00476    0.02486
N1    4    0.891914    0.549040    0.029923     0.91688    0.05144
0.04818 =
         0.05338   -0.00245   -0.00284    0.01798
C5    1    0.832907    0.665814    0.958529    10.50000    0.04436
0.05725 =
         0.02681   -0.01592   -0.00796    0.02862
AFIX  43
H5    2    0.837494    0.674989    1.092428    10.50000   -1.20000
AFIX   0
C1    1    0.910903    0.633101    0.539535    11.00000    0.03850
```

```
0.04155 =
         0.02200    0.00224   -0.00012    0.01649
C2    1    0.867692    0.640772    0.659233    11.00000    0.04943
0.04457 =
         0.02239   -0.00261   -0.00227    0.02564
C3    1    0.876353    0.659470    0.865935    11.00000    0.03533
0.03296 =
         0.03038    0.00124   -0.00092    0.01475
C4    1    0.815520    0.631041    0.571599    10.50000    0.04199
0.05682 =
         0.02380   -0.00513   -0.00257    0.02841
AFIX  43
H4    2    0.808363    0.616726    0.442690    10.50000   -1.20000
AFIX   0
PART 2
O4B   5    0.863760    0.503335    0.088127    31.00000    0.08887
0.07095 =
         0.08418   -0.00657   -0.04911    0.01620
PART 3
O4C   5    0.888392    0.519471   -0.100509    21.00000    0.06055
0.09395 =
         0.09894   -0.01408   -0.00611    0.02226
PART 1
O4A   5    0.842811    0.528739    0.034471    41.00000    0.07413
0.07419 =
         0.08586   -0.00942   -0.02297    0.02349
PART 4
O4D   5    0.853565    0.534358   -0.079116    51.00000    0.06843
HKLF 4




REM  shelxt_a.res in R3m
REM wR2 = 0.1452, GooF = S = 0.704, Restrained GooF = 0.702 for all
data
REM R1 = 0.0624 for 1187 Fo > 4sig(Fo) and 0.3118 for all 5203 data
REM 121 parameters refined using 66 restraints

END

WGHT      0.0313      0.0000

REM Highest difference peak  0.553,  deepest hole -0.355,  1-sigma
level  0.091
Q1    1   0.7070  0.4140 -0.1842  10.50000  0.05    0.55
Q2    1   0.6667  0.3333 -0.2193  10.16667  0.05    0.47
Q3    1   0.7474  0.4949  0.3943  10.50000  0.05    0.46
Q4    1   0.9922  0.6625  0.1470  11.00000  0.05    0.41
Q5    1   0.8292  0.4146 -0.1120  10.50000  0.05    0.37
Q6    1   0.9039  0.6145  0.1524  11.00000  0.05    0.37
Q7    1   0.7867  0.3934  0.3078  10.50000  0.05    0.37
Q8    1   0.9044  0.5971  0.1272  11.00000  0.05    0.36
Q9    1   0.9916  0.6229  0.1475  11.00000  0.05    0.36
```

```
Q10   1   0.7330  0.3665 -0.1764  10.50000  0.05    0.36
Q11   1   0.8626  0.4313 -0.2303  10.50000  0.05    0.35
Q12   1   0.8306  0.4295 -0.2097  11.00000  0.05    0.35
Q13   1   0.7578  0.5156  0.5164  10.50000  0.05    0.33
Q14   1   0.9507  0.6661  0.1782  11.00000  0.05    0.32
Q15   1   0.9477  0.7516  1.1183  11.00000  0.05    0.32
Q16   1   0.7125  0.4250  0.4343  10.50000  0.05    0.31
;
_shelx_res_checksum               81289
_shelx_hkl_file
;
   9 -13   7  -62.60  125.85
  31 -20   9  973.29  564.03
  -1  -3   2  259.76   70.26
   1  -7  11 -943.18  309.02
   2  -9   8  100.89  148.70
  -6  -2  11 -205.35  407.54
 -16   8  12  503.81  734.39
  32  -1  -3  -67.48  182.90
  13  12  -5  -62.46  104.20
  32  -9  -1    0.13  161.38
   7  15  -5  159.53   77.65
  35 -14   1  -52.03  223.98
  29  -9  -1  -42.00  116.48
  34 -16   2    0.00  186.07
  32 -14   1  -71.31  162.44
  24 -13   1   67.48  111.59
  28 -18   4  -64.71  168.91
  26 -19   6 1270.16  204.83
 -31  38   0 -108.82  181.72
  20 -17  10    4.89  139.59
  19 -17   6  689.62  121.50
 -19  21  -1 -111.86   85.84
   3  -4   1  672.06   68.01
 -13  11   0  -11.62   65.90
  -9   5   1  632.84   73.43
 -18  14   1  546.60  117.80
 -28  23   3  126.78  158.87
 -11   5   2  351.81   69.86
 -13   5  12  104.72  224.37
 -27  19   5  -81.22  168.91
  -7  -2  10   47.01  133.38
 -20  11  11   51.90  275.08
  -9  -1   7 -222.52   90.46
 -11   1   6  270.59   89.14
 -15   5  10  -77.39  192.15
 -24  14   7   23.64  199.15
  31   5  -4 -186.34  238.63
  27   4  -4   -3.83  214.86
  11  13  -5  233.22  108.82
  16   5  -4  -41.60  108.69
  23  -5  -2  120.18  126.25
  34 -20   6 -280.37  220.41
  25 -11   0   99.97  135.10
```

−10	24	−4	300.31	84.12
30	−20	8	−0.13	213.41
−16	26	−3	81.35	100.23
−27	34	−1	516.89	215.66
25	−19	8	−183.43	179.60
−19	25	−2	−34.47	105.25
−26	32	−1	4.75	190.43
−25	30	−1	−28.92	171.28
−15	16	−1	60.88	93.24
−25	26	0	90.33	126.12
−33	34	2	−255.80	220.67
−32	32	2	0.40	205.09
−27	20	4	96.67	155.96
−8	−1	5	−4.09	94.16
−23	13	9	−139.72	223.18
18	11	−5	40.94	206.15
21	4	−4	−6.47	150.15
9	14	−5	2.38	94.03
34	−20	9	−306.78	320.38
19	−7	−1	335.43	148.70
26	−15	2	99.71	145.40
−28	36	−1	16.11	226.88
−32	40	0	46.09	256.99
26	−19	9	−58.11	204.69
21	−17	5	11.36	115.69
−10	8	0	−10.43	64.58
−30	28	2	28.53	249.33
−23	16	3	−31.03	160.72
−11	2	11	33.41	199.54
−16	7	4	33.15	122.95
−28	19	7	−12.41	226.22
−23	13	6	14.13	190.43
−25	15	8	−8.06	233.22
33	0	−3	−54.15	266.90
29	−1	−3	−106.04	256.33
24	4	−4	−32.75	226.62
32	−5	−2	−90.33	284.20
23	−1	−3	−153.85	166.66
37	−17	3	33.94	255.41
18	0	−3	268.61	124.14
31	−17	3	118.99	220.41
16	−3	−2	182.51	125.19
31	−19	5	0.00	234.67
−29	38	−1	4.49	244.58
−11	15	−2	75.54	81.88
−24	28	−1	143.15	155.96
19	−17	9	−99.44	138.27
−16	14	0	119.38	112.52
−24	19	2	0.00	148.44
−20	14	2	72.90	165.21
−32	26	5	35.39	249.33
−19	10	4	108.95	130.74
−18	7	8	30.90	220.15
26	−1	−3	280.76	194.00

14	6	−4	85.18	118.59
34	−17	3	−209.71	263.33
24	−8	−1	74.48	153.06
33	−20	8	−187.00	304.66
18	−9	0	81.88	130.34
21	−15	3	26.94	130.34
15	−10	1	278.91	101.69
−8	12	−2	90.20	54.54
−24	24	0	0.00	166.53
−29	26	2	−14.79	230.97
−32	27	4	0.00	267.95
−3	−3	3	722.77	76.60
−27	21	3	−26.41	190.96
−5	−2	12	−0.13	223.45
−10	2	12	19.15	255.93
−4	−5	7	54.94	80.43
−13	4	4	115.16	138.00
−12	2	10	−39.88	197.43
−15	4	8	152.79	187.26
−17	6	7	−26.02	166.40
28	5	−4	212.35	262.80
34	−11	0	64.31	276.80
37	−18	4	24.70	262.80
37	−19	5	−361.19	283.93
28	−11	0	−70.65	179.47
35	−20	7	−333.19	340.45
34	−19	5	68.54	255.41
4	11	−4	−19.15	78.05
31	−18	4	232.96	257.65
30	−19	10	0.00	333.72
19	−13	2	107.89	154.64
22	−18	7	−23.64	188.19
−31	24	5	−6.34	284.20
−16	8	3	241.28	144.34
−26	17	5	−12.81	229.65
−20	9	7	5.68	244.18
−21	10	8	0.00	238.24
16	12	−5	201.92	215.52
−8	16	−3	251.58	76.20
33	−8	−1	103.67	280.37
−23	26	−1	−73.29	156.76
−6	7	−1	258.44	63.13
31	−11	0	−87.03	207.20
36	−13	1	−380.07	268.22
27	−13	1	146.19	179.07
−21	17	1	284.20	152.53
29	−15	2	−28.13	176.70
−19	11	3	−32.49	150.42
34	−18	4	−44.50	247.75
21	−16	4	−149.23	141.17
−31	25	4	155.57	260.56
−26	18	4	0.00	183.17
26	−18	5	76.99	207.73
−13	3	5	1.06	119.91

−31	23	6	−43.84	287.89
−27	17	7	−154.51	254.22
−14	3	7	4.62	151.87
−26	16	9	0.00	301.89
−16	5	9	−3.17	226.62
−19	8	9	−204.17	252.24
19	5	−4	−166.79	159.79
30	0	−3	167.45	265.18
16	1	−3	37.90	137.61
−15	24	−3	0.00	121.10
21	−4	−2	96.01	139.72
14	−2	−2	46.62	107.89
−18	23	−2	1.85	132.19
27	−8	−1	29.19	168.25
17	−6	−1	416.78	135.10
−18	19	−1	−12.94	145.00
35	−15	2	−240.35	265.71
−28	24	2	13.21	187.66
26	−16	3	182.24	155.57
−33	30	3	137.21	286.97
−13	5	3	315.49	118.33
−22	13	4	167.45	163.23
−22	12	5	−12.41	169.04
29	−19	6	−90.46	230.71
17	−16	6	−126.25	118.33
27	−19	7	−113.57	226.48
29	6	−4	265.05	260.82
25	5	−4	31.56	251.44
12	7	−4	361.85	122.68
33	−4	−2	−0.79	250.52
−14	18	−2	17.70	124.40
30	−8	−1	−47.28	193.47
23	−10	0	−154.78	151.87
−19	17	0	41.60	150.55
33	−13	1	23.64	250.52
30	−13	1	213.01	173.40
13	−9	1	355.90	95.08
−25	22	1	−67.22	178.41
32	−15	2	43.84	241.54
−32	28	3	146.72	259.24
26	−17	4	0.00	157.68
−30	22	5	10.43	246.95
24	−18	6	−103.01	216.05
−19	8	6	55.73	159.53
−23	12	7	−42.92	246.43
−7	−3	8	82.54	145.00
−28	18	8	47.67	276.14
−8	−2	9	95.08	178.55
−22	11	9	163.36	254.09
25	−18	10	226.35	272.57
22	5	−4	0.00	192.54
21	0	−3	274.42	155.44
−11	19	−3	72.50	99.97
−23	22	0	89.67	145.93

24	−14	2	0.00	153.06
−23	17	2	−36.98	155.04
−26	19	3	298.19	174.58
−30	23	4	−10.43	256.20
−3	−5	5	50.58	83.86
−16	5	6	−62.60	147.78
−12	1	8	−61.80	170.23
−24	13	8	−11.49	235.46
17	−15	11	81.09	159.00
−16	6	11	97.59	278.38
−19	9	11	0.00	311.14
30	7	−4	201.00	271.65
10	8	−4	48.86	107.89
6	10	−4	67.75	97.20
27	0	−3	−72.37	230.45
−26	36	−2	140.91	238.77
−25	34	−2	−4.75	237.31
−24	32	−2	0.00	227.54
−22	24	−1	170.62	149.36
16	−8	0	410.31	114.50
−31	26	3	196.64	256.86
−22	14	3	1.32	159.27
19	−16	5	13.73	135.36
32	−19	6	0.00	232.56
−30	21	6	93.76	250.39
−22	11	6	0.00	191.75
28	−19	8	55.99	242.73
29	−19	9	173.00	278.38
−13	2	9	0.00	198.88
18	−16	10	−40.81	164.42
−2	−6	10	0.00	134.70
−1	−6	11	−164.42	158.21
−8	−1	11	0.00	223.05
−15	6	12	−28.39	312.85
−18	9	12	−139.72	370.83
14	13	−5	122.95	176.30
8	9	−4	109.87	105.52
31	1	−3	−140.64	246.69
24	0	−3	22.71	164.55
34	−3	−2	52.82	242.86
30	−4	−2	43.45	249.20
12	−1	−2	215.52	94.69
−23	30	−2	0.00	180.26
34	−7	−1	115.95	282.21
22	−7	−1	91.78	148.30
35	−10	0	−191.09	254.75
−31	35	0	181.58	237.58
−30	33	0	141.70	243.26
−29	31	0	−274.55	265.31
−33	35	1	−250.92	248.14
−32	33	1	−437.12	256.33
−31	31	1	57.97	260.42
17	−12	2	158.34	131.80
−27	22	2	−13.87	176.30

-8	2	2	1666.87	156.36
29	-16	3	-40.67	186.21
35	-19	6	30.51	262.67
-22	12	11	-20.87	295.29
2	19	-5	49.13	64.84
26	6	-4	41.73	239.29
17	6	-4	54.28	145.53
14	2	-3	250.92	142.23
-20	31	-3	0.00	195.19
-32	37	0	72.77	240.48
11	-8	1	211.03	75.41
-30	29	1	-89.67	243.39
-14	9	1	14.13	92.44
-17	12	1	307.04	139.06
35	-16	3	-242.99	257.65
-10	1	4	18.22	118.72
-25	16	4	3.57	172.74
29	-18	5	-11.49	227.01
-29	20	5	28.79	240.75
-25	15	5	-0.66	196.51
-8	-2	6	0.79	105.38
-26	15	7	-15.45	252.50
24	-18	9	0.00	206.02
-25	14	9	102.48	266.90
-9	-1	10	-146.46	191.49
-17	6	10	-55.33	238.90
-20	9	10	525.21	269.93
15	-5	-1	166.53	104.20
32	-10	0	-106.57	212.88
26	-10	0	58.24	158.74
-33	39	0	-195.45	262.54
-28	29	0	-46.49	213.67
-34	33	2	-5.41	241.94
-16	9	2	461.95	147.78
32	-16	3	-72.11	244.58
-30	24	3	34.73	238.11
29	-17	4	-121.63	202.32
-29	21	4	178.81	235.07
30	-19	7	-77.26	227.01
-30	20	7	0.00	249.07
-11	0	7	203.90	133.65
32	-19	9	22.05	282.74
17	-16	9	-41.60	154.91
-13	3	11	0.00	241.41
12	14	-5	91.39	151.74
32	2	-3	-41.86	242.86
-19	29	-3	0.00	152.66
19	-3	-2	35.00	151.87
10	0	-2	4936.84	416.78
-22	28	-2	-111.72	157.55
31	-7	-1	413.22	233.09
29	-10	0	229.92	180.13
25	-12	1	-194.92	157.94
20	-11	1	314.04	163.49

−29	27	1	−41.47	211.43
−24	20	1	92.18	153.98
36	−14	2	450.99	274.29
−33	31	2	−109.74	246.03
−19	12	2	188.85	169.57
19	−15	4	98.12	125.19
35	−18	5	−31.69	231.77
32	−18	5	−87.03	222.79
−13	2	6	22.71	134.04
−25	14	6	−155.83	239.29
20	−17	7	−15.32	196.51
−27	16	8	24.17	273.63
−7	−1	12	−87.29	241.67
−12	3	12	68.54	266.10
27	7	−4	−136.15	226.09
23	6	−4	1.45	221.86
20	6	−4	119.25	161.11
−13	27	−4	76.73	118.06
−6	19	−4	175.51	51.24
28	1	−3	−170.62	249.46
19	1	−3	143.68	137.34
−17	21	−2	153.98	120.04
35	−6	−1	−56.39	236.52
25	−7	−1	220.28	152.00
−29	35	−1	9.51	230.18
−28	33	−1	43.98	240.09
36	−9	0	−97.20	234.67
21	−9	0	13.47	161.11
14	−7	0	1480.93	158.61
−27	27	0	−24.56	170.49
−7	5	0	−71.71	79.50
34	−12	1	62.99	248.67
9	−7	1	1094.26	115.03
5	−5	1	879.26	99.18
−20	15	1	353.79	164.02
27	−14	2	134.44	168.64
24	−15	3	0.00	127.17
−25	17	3	40.67	165.47
35	−17	4	106.84	221.20
32	−17	4	70.52	234.41
24	−17	5	205.75	162.70
15	−15	6	55.73	112.65
34	−19	8	−11.89	278.12
31	−19	8	−48.86	254.48
14	−15	8	−16.51	126.25
0	−8	8	−15.19	88.88
−20	8	8	10.30	219.22
−14	3	10	191.22	207.73
12	3	−3	20.60	122.02
−14	22	−3	0.00	103.54
31	−3	−2	102.48	251.97
8	1	−2	2208.98	198.88
6	2	−2	1319.16	127.31
28	−7	−1	288.82	167.98

−30	37	−1	0.00	225.16
−27	31	−1	71.31	223.05
−21	22	−1	199.15	131.53
33	−14	2	−56.65	228.99
−26	20	2	140.38	172.08
−13	6	2	101.03	97.73
−10	2	3	339.93	95.08
−18	8	4	139.46	133.12
−21	11	4	27.47	144.61
−10	0	5	12.81	120.31
−1	−7	6	−91.91	69.46
−19	7	7	−17.70	192.02
−17	5	8	−190.43	197.43
28	−18	10	0.00	265.84
−9	22	−4	260.82	90.07
−21	26	−2	119.91	138.53
−31	39	−1	217.90	240.09
−11	11	−1	1153.42	127.31
−15	12	0	255.41	113.31
31	−12	1	−228.60	193.73
28	−12	1	33.02	165.87
7	−6	1	221.47	65.24
30	−14	2	111.99	175.77
22	−13	2	19.28	135.10
−31	27	2	160.98	235.46
−22	15	2	−13.87	136.82
−29	22	3	15.32	215.39
24	−16	4	103.54	148.30
−28	18	5	51.77	226.22
27	−18	6	363.43	207.20
25	−18	7	−19.81	192.68
−22	10	7	229.65	222.92
−23	11	8	−88.61	223.84
−21	9	9	105.12	225.82
10	15	−5	91.91	131.93
28	8	−4	29.71	227.28
15	7	−4	170.36	136.02
25	1	−3	0.00	190.70
22	1	−3	−41.60	155.57
−18	27	−3	139.98	139.72
−5	13	−3	282.08	55.33
20	−6	−1	174.72	155.44
13	−4	−1	306.25	104.20
−26	29	−1	26.41	181.85
−17	17	−1	287.10	140.91
−26	25	0	−35.13	159.79
−11	6	1	58.90	76.60
15	−11	2	15.19	106.44
−28	19	4	0.00	197.04
−15	5	4	116.61	149.76
17	−15	5	−2.25	117.80
22	−17	6	39.49	172.74
−6	−4	7	53.62	105.65
−29	18	7	16.24	227.41

−18	6	9	0.00	206.41
4	18	−5	0.00	91.78
29	2	−3	−18.88	228.33
2	8	−3	37.11	52.03
28	−3	−2	−55.99	188.19
33	−9	0	45.83	233.35
−18	15	0	76.46	145.93
−6	2	1	758.16	102.61
−18	9	3	−124.40	152.13
−33	26	4	−0.53	229.39
−24	13	5	−47.28	164.94
36	−18	6	43.98	241.54
−16	4	7	−22.19	146.46
−10	−1	9	110.27	168.38
−24	12	9	0.00	238.11
31	−18	10	−86.50	282.21
23	−17	10	−125.19	209.05
8	16	−5	110.01	118.99
24	7	−4	127.70	228.47
10	4	−3	358.41	116.08
32	−2	−2	−82.41	237.05
17	−2	−2	121.76	141.17
32	−6	−1	−83.73	238.24
−25	27	−1	94.56	166.93
−23	18	1	40.81	136.15
−30	25	2	−202.58	221.33
−21	12	3	57.45	132.99
−24	14	4	75.14	159.13
−32	23	5	1.98	218.69
−15	4	5	106.04	126.78
30	−18	6	23.24	201.13
−25	13	7	202.05	216.45
−26	14	8	0.00	219.88
27	−18	9	−91.39	217.77
−4	−5	10	−23.37	145.40
−3	−5	11	24.43	157.55
−18	7	11	191.36	241.54
−21	10	11	−107.10	271.39
−17	7	12	−260.82	265.84
6	17	−5	−12.68	106.04
17	2	−3	117.93	124.40
24	−9	0	6.74	146.98
12	−6	0	106.84	87.95
−12	9	0	325.13	90.46
35	−11	1	42.79	237.18
−34	29	3	4.49	220.54
−15	6	3	93.76	146.06
33	−18	6	113.97	213.54
−32	22	6	0.00	211.96
−18	6	6	7.13	148.83
−21	9	6	−120.44	173.53
26	−18	8	−28.26	198.36
21	−17	8	11.62	160.72
−9	−2	8	102.48	148.17

−14	2	8	0.00	164.28
−15	3	9	94.56	183.96
16	−15	10	0.00	142.63
−10	0	11	85.05	180.00
0	−6	12	0.00	159.93
21	7	−4	0.00	180.26
18	7	−4	96.01	140.25
30	3	−3	−6.87	220.81
30	−9	0	55.86	174.98
23	−11	1	87.82	135.10
18	−10	1	75.41	127.04
−27	23	1	−175.24	163.89
−25	18	2	0.00	154.12
−28	20	3	−136.02	176.57
36	−16	4	162.96	211.96
17	−14	4	276.80	115.16
−32	24	4	0.00	226.09
36	−17	5	133.65	215.66
27	−17	5	114.89	201.66
−24	12	6	53.48	216.45
−19	7	10	243.12	229.65
−22	10	10	−134.83	255.14
15	−14	11	0.00	141.57
25	8	−4	29.19	227.67
13	8	−4	−50.05	126.12
26	2	−3	50.98	208.39
8	5	−3	394.60	108.82
33	−1	−2	0.00	215.66
−20	24	−2	−68.80	134.44
−13	16	−2	197.70	116.08
29	−6	−1	8.45	172.34
23	−6	−1	32.88	138.80
11	−3	−1	397.90	87.82
−20	20	−1	36.58	134.57
27	−9	0	7.53	154.91
19	−8	0	382.05	157.81
−25	23	0	−65.50	152.40
−21	18	0	70.26	140.38
−34	34	1	5.94	215.92
34	−13	2	189.38	228.47
−33	27	3	42.39	231.90
−24	15	3	43.98	167.32
27	−16	4	0.26	172.21
13	−14	6	−38.17	99.18
28	−18	7	0.00	194.26
22	−17	9	21.66	171.42
15	−15	9	−107.10	130.21
−27	15	9	103.14	232.03
−11	0	10	0.00	171.28
−15	4	11	0.00	211.56
−12	25	−4	44.90	103.14
31	4	−3	190.30	213.94
−17	25	−3	63.13	120.18
29	−2	−2	−12.55	218.69

−27	35	−2	155.17	206.15
−26	33	−2	17.96	213.81
33	−5	−1	122.82	252.90
26	−6	−1	83.20	157.94
−24	25	−1	121.36	152.93
34	−8	0	23.37	234.01
−33	36	0	168.77	211.56
−32	34	0	−4.23	208.00
32	−11	1	65.24	201.13
−33	32	1	−132.06	224.24
25	−13	2	192.94	151.21
−29	23	2	−44.64	183.56
−5	−2	3	460.50	76.99
33	−16	4	33.15	205.62
−27	17	4	−85.31	176.43
33	−17	5	68.14	202.58
30	−17	5	163.76	204.17
−31	21	5	0.00	211.69
−27	16	5	15.85	209.05
34	−18	7	132.33	224.37
18	−16	7	36.18	164.02
−28	16	7	19.28	208.79
33	−18	9	0.00	239.16
30	−18	9	6.47	222.13
−9	0	12	71.58	213.28
−14	4	12	0.00	225.96
26	9	−4	106.18	205.75
23	2	−3	0.00	149.23
20	2	−3	29.58	141.83
6	6	−3	337.15	89.41
4	7	−3	1346.89	134.31
−5	9	−2	1306.74	117.67
−16	19	−2	330.42	141.17
18	−5	−1	0.00	160.59
−34	38	0	260.03	216.45
−31	32	0	−23.37	225.56
−32	30	1	−32.22	218.43
13	−10	2	269.27	94.69
30	−16	4	110.01	215.13
−31	22	4	136.42	230.84
22	−16	5	120.31	136.29
−5	−4	5	237.71	102.08
−10	−1	6	59.16	116.08
31	−18	7	169.17	211.83
−13	1	7	15.45	128.50
29	−18	8	−115.95	211.69
−29	17	8	0.00	221.33
−16	4	10	13.87	186.60
−25	13	10	0.53	222.52
−25	31	−2	−73.03	192.41
10	−5	0	11.36	76.73
−30	30	0	81.48	215.26
36	−10	1	0.00	228.20
29	−11	1	−28.26	162.83

26	−11	1	0.00	150.02
−19	13	1	208.92	151.21
31	−13	2	115.95	185.55
28	−13	2	32.75	158.47
20	−12	2	164.28	150.55
−35	32	2	−211.83	209.45
−32	25	3	0.00	230.05
22	−15	4	290.27	143.15
−12	2	4	63.79	135.89
−15	3	6	76.99	141.17
−27	15	6	−113.44	221.47
32	−18	8	−165.21	228.07
12	−14	8	−30.77	119.52
−22	9	8	−212.22	198.88
27	10	−4	180.79	214.07
22	8	−4	−108.29	205.09
27	3	−3	−130.34	214.47
−22	32	−3	−45.30	194.53
26	−2	−2	−11.89	151.87
15	−1	−2	−32.22	143.68
−10	13	−2	264.91	85.05
−31	28	1	−167.85	216.32
−26	21	1	149.10	156.36
−16	10	1	12.68	120.18
−10	3	2	3666.28	317.47
−18	10	2	206.15	145.00
−21	13	2	−4.62	154.25
15	−14	5	145.40	108.29
25	−17	6	−19.68	194.00
20	−16	6	25.36	138.14
23	−17	7	−150.42	176.43
−2	−7	8	55.60	97.06
−19	6	8	218.30	195.98
26	−17	10	−42.39	202.71
16	8	−4	50.71	127.97
11	9	−4	26.68	110.14
15	3	−3	262.93	136.55
30	−1	−2	−42.13	224.37
−24	29	−2	412.69	166.93
30	−5	−1	66.43	186.21
9	−2	−1	1031.26	122.02
−31	36	−1	9.64	209.18
−30	34	−1	139.19	203.77
−29	28	0	−137.87	206.41
−27	18	3	−0.66	164.15
−20	9	4	11.75	140.38
−12	1	5	−50.45	117.27
−21	8	7	179.87	202.45
−24	11	7	349.43	204.17
−25	12	8	−24.43	211.43
−20	7	9	1.72	184.89
−23	10	9	−1.58	197.83
−2	−5	12	−5.81	150.55
19	8	−4	104.99	156.23

−13	20	−3	89.01	102.74
−32	38	−1	143.15	206.54
−29	32	−1	27.07	208.26
35	−7	0	74.22	207.60
31	−8	0	207.47	183.56
22	−8	0	167.72	155.83
16	−9	1	325.93	118.59
−22	16	1	130.61	148.83
35	−12	2	0.00	203.64
−3	−2	2	352.87	60.35
−28	21	2	212.22	156.10
−31	23	3	70.52	209.58
−23	12	4	−42.39	142.49
−30	19	5	−50.18	216.05
−23	11	5	93.37	152.53
−8	−3	7	258.18	118.85
−5	−4	11	−60.48	151.61
−21	30	−3	−27.60	160.98
−10	17	−3	186.21	104.99
−19	22	−2	276.93	129.95
−8	8	−1	75.01	67.62
−23	23	−1	387.60	147.25
−33	28	2	197.43	210.77
−15	7	2	107.50	122.95
−24	16	2	−55.07	153.32
−12	3	3	422.20	121.50
−30	20	4	−6.07	224.11
34	−17	6	−10.83	215.92
−3	−6	6	49.52	77.12
−18	5	7	−119.52	170.62
−12	0	9	1.98	156.36
−26	13	9	39.49	225.16
−6	−4	10	−1.06	139.72
−12	1	11	−48.86	178.02
−20	8	11	28.79	216.98
−23	11	11	−14.00	237.58
−19	8	12	76.99	236.39
23	9	−4	−28.00	201.39
28	4	−3	27.60	210.37
24	3	−3	−114.36	168.64
27	−5	−1	90.73	155.17
21	−5	−1	−5.81	148.30
−28	30	−1	133.91	197.17
28	−8	0	−53.75	161.91
17	−7	0	1101.79	168.91
33	−10	1	354.58	224.77
21	−10	1	358.41	168.64
−20	10	3	−14.53	140.51
15	−13	4	91.52	118.99
−17	6	4	206.94	134.97
−26	15	4	102.08	158.74
−26	14	5	0.00	192.28
28	−17	6	58.11	188.72
−27	14	7	192.41	192.41

−11	−1	8	19.28	143.02
−28	15	8	−7.13	201.13
32	−17	10	168.91	231.24
29	−17	10	0.00	211.69
21	−16	10	−67.88	164.55
31	0	−2	136.42	208.39
−23	27	−2	86.76	137.48
24	−5	−1	−47.67	143.68
−19	18	−1	−69.46	149.36
25	−8	0	20.07	144.87
8	−4	0	353.40	87.16
−28	26	0	1.98	156.49
−17	13	0	143.29	143.02
−32	26	2	−69.73	214.60
−23	13	3	18.22	150.95
34	−15	4	−9.64	201.39
34	−16	5	−6.47	194.66
25	−16	5	−130.34	153.19
−17	5	5	37.90	136.42
31	−17	6	−133.25	187.00
−23	10	6	143.81	187.39
24	−17	8	28.53	169.83
−16	3	8	49.52	166.26
25	−17	9	29.58	176.04
−17	4	9	0.79	175.51
9	10	−4	209.84	103.01
29	5	−3	−287.36	206.02
21	3	−3	11.23	143.02
18	3	−3	257.78	133.12
−16	23	−3	110.40	117.27
27	−1	−2	0.00	164.94
31	−4	−1	−186.07	211.03
16	−4	−1	585.03	137.08
7	−1	−1	279.44	79.50
−20	16	0	0.92	155.57
−13	7	1	−5.94	96.40
−25	19	1	118.19	143.81
32	−12	2	98.52	192.15
23	−12	2	14.66	130.48
11	−9	2	780.48	108.29
25	−15	4	5.02	141.04
−34	25	4	86.90	197.96
11	−13	6	86.90	84.65
−20	7	6	−60.75	149.63
35	−17	7	−11.36	210.90
−13	1	10	−74.09	161.91
−21	8	10	−167.45	207.86
−24	11	10	0.00	209.58
−17	5	11	48.47	187.39
−11	1	12	−15.32	188.19
24	10	−4	−58.11	196.77
−15	28	−4	−8.45	115.29
30	6	−3	0.00	184.23
−20	28	−3	−35.26	131.00

32	1	−2	74.48	207.07
−27	28	−1	−153.06	164.15
−13	12	−1	301.89	99.44
32	−7	0	−89.54	217.50
−9	6	0	489.55	90.86
30	−10	1	−38.03	180.92
−29	24	1	81.09	161.51
36	−11	2	−143.15	200.73
−30	21	3	122.82	195.05
−17	7	3	251.44	153.59
−26	13	6	−71.71	210.77
26	−17	7	28.53	174.72
16	−15	7	5.55	122.82
20	−16	9	−76.99	148.44
20	−15	11	−46.22	162.04
−16	5	12	126.38	209.18
25	11	−4	−76.60	188.58
20	9	−4	−47.94	178.81
14	9	−4	203.24	134.17
13	4	−3	68.94	135.76
24	−10	1	145.00	151.74
29	−12	2	167.98	156.10
18	−11	2	176.43	137.87
−27	19	2	51.77	153.59
35	−13	3	0.00	184.36
−35	28	3	−94.69	198.49
−26	16	3	47.94	166.79
31	−15	4	−7.40	225.43
28	−15	4	9.11	169.04
31	−16	5	40.67	185.68
28	−16	5	−63.13	189.64
20	−15	5	29.32	126.78
−29	17	5	77.39	221.07
32	−17	7	−126.38	189.77
−30	17	7	−205.22	199.41
28	−17	9	−164.55	185.28
14	−14	10	−22.71	114.50
−18	5	10	−106.84	174.72
−4	−4	12	184.49	152.27
17	9	−4	31.30	136.68
25	4	−3	151.74	194.39
24	−1	−2	47.15	146.72
−35	37	0	−112.38	203.37
−34	35	0	−60.88	189.77
−27	24	0	−130.74	157.28
−14	10	0	948.73	130.87
34	−9	1	214.47	206.02
27	−10	1	326.06	157.15
−35	33	1	55.20	197.30
−8	3	1	78.71	82.01
26	−12	2	130.87	151.74
−31	24	2	−3.96	202.32
20	−14	4	8.45	126.91
−29	18	4	90.99	208.26

−33	22	5	−93.90	193.21
−12	0	6	577.90	137.74
29	−17	7	17.43	175.38
−15	2	7	232.30	138.27
33	−17	8	−82.80	201.00
−4	−6	8	1.98	101.82
31	−17	9	0.13	194.92
13	−14	9	19.02	105.38
28	0	−2	26.54	187.53
−29	36	−2	81.22	179.60
−22	25	−2	92.18	132.85
5	0	−1	906.33	111.72
1	2	−1	541.85	54.41
−22	21	−1	4.89	130.34
6	−3	0	104.72	82.93
−33	33	0	−0.13	182.11
−34	31	1	−18.62	192.68
−34	26	3	1.45	187.39
−33	23	4	146.85	201.39
13	−13	5	92.71	94.95
−7	−3	5	396.18	122.68
35	−16	6	−27.86	185.41
−17	4	6	3.83	129.16
−29	16	6	−113.18	192.02
27	−17	8	25.22	169.57
−24	10	8	139.85	177.89
13	−13	11	58.11	107.76
−7	−3	11	96.27	146.06
7	11	−4	92.31	95.08
−28	34	−2	60.09	186.21
28	−4	−1	105.25	153.59
−26	26	−1	364.49	157.42
33	−6	0	−16.38	216.05
29	−7	0	−49.65	158.87
20	−7	0	122.55	157.28
14	−8	1	279.84	101.95
−7	−1	3	201.79	78.97
−14	3	4	554.52	152.79
23	−16	6	87.16	163.76
18	−15	6	−103.54	115.69
−23	9	7	−124.53	193.87
30	−17	8	0.00	184.62
−21	7	8	34.73	173.13
−27	13	8	0.26	187.26
21	10	−4	−179.21	196.11
−11	23	−4	80.82	88.88
26	5	−3	−36.71	204.43
−27	32	−2	51.77	192.41
−15	17	−2	147.78	126.25
19	−4	−1	31.56	150.81
15	−6	0	323.42	111.99
−32	31	0	88.35	190.56
−33	29	1	80.95	198.22
−28	22	1	−72.90	162.04

-21	14	1	665.72	174.98
33	-11	2	197.04	202.32
-20	11	2	-163.10	170.36
-23	14	2	-74.61	135.76
32	-13	3	28.66	191.09
35	-14	4	-26.41	180.79
35	-15	5	307.44	182.38
21	-16	7	-163.49	165.34
-10	-2	7	120.04	115.55
-26	12	7	-133.78	189.24
10	-13	8	36.85	98.65
-14	1	9	43.18	148.17
-22	8	9	197.83	182.38
-25	11	9	0.00	190.30
-8	-3	10	216.05	138.40
-14	2	11	-59.56	165.21
22	4	-3	49.79	147.64
16	4	-3	29.98	121.76
-19	26	-3	25.49	122.02
-18	20	-2	40.94	112.91
3	1	-1	900.26	96.40
35	-8	1	-0.40	197.83
19	-9	1	372.41	151.47
-18	11	1	578.82	163.10
-12	4	2	55.73	91.78
36	-12	3	-27.73	179.47
-29	19	3	19.02	169.57
-22	10	4	247.09	142.49
-25	13	4	-0.26	151.21
-14	2	5	149.49	116.48
-32	20	5	0.00	183.96
-25	12	5	-13.47	158.87
-5	-5	6	121.23	89.67
-20	6	7	133.65	183.70
24	-16	10	-50.45	162.70
-22	9	11	0.00	202.58
-21	9	12	-148.70	206.15
-1	15	-4	57.84	43.05
29	1	-2	-359.73	217.90
25	-4	-1	-4.49	142.36
14	-3	-1	1634.92	182.90
-34	39	-1	-136.68	185.81
-33	37	-1	3.43	182.38
-32	35	-1	-103.01	179.87
26	-7	0	213.54	154.64
23	-7	0	388.13	152.40
-31	29	0	-3.70	208.92
-26	22	0	0.13	143.42
31	-9	1	108.55	166.53
-24	17	1	111.59	145.40
-35	29	2	113.57	185.55
-30	22	2	5.41	167.06
-33	24	3	0.00	200.86
13	-12	4	132.99	114.76

−32	21	4	22.98	192.54
32	−16	6	38.83	173.13
−29	15	7	−96.54	181.06
−13	0	8	0.00	141.57
−13	2	12	30.64	170.23
22	11	−4	11.09	175.91
12	10	−4	181.19	120.18
5	12	−4	45.43	85.71
27	6	−3	45.16	189.38
19	4	−3	108.16	137.21
11	5	−3	57.18	126.65
−24	33	−3	−144.87	187.79
25	0	−2	−91.12	153.32
−26	30	−2	−113.31	178.41
22	−4	−1	63.52	134.17
−31	33	−1	−170.09	189.38
34	−5	0	−122.16	188.32
−32	27	1	−44.11	198.62
9	−8	2	1625.94	154.12
−17	8	2	87.29	142.23
−26	17	2	148.17	148.44
−14	4	3	293.70	132.19
−19	7	4	117.67	131.40
−28	15	5	88.08	209.98
26	−16	6	0.00	181.58
−18	4	8	−33.41	157.15
−19	5	9	35.79	165.34
−15	2	10	29.05	152.79
−19	6	11	−0.13	172.47
−6	−3	12	−109.21	147.38
18	10	−4	−30.77	148.96
−14	26	−4	−8.45	101.42
28	7	−3	76.73	170.36
−12	14	−2	113.44	100.76
29	−3	−1	162.83	171.42
−25	24	−1	87.03	137.48
4	−2	0	407.67	50.84
30	−11	2	81.22	158.08
21	−11	2	184.36	144.08
−22	11	3	79.90	151.74
32	−14	4	20.60	198.49
23	−14	4	5.02	139.72
−28	16	4	100.23	169.83
32	−15	5	−136.02	178.68
23	−15	5	106.18	129.02
−19	6	5	84.39	137.48
29	−16	6	0.00	170.89
−22	8	6	115.29	160.32
−25	11	6	−234.14	193.87
30	−16	10	−89.54	182.38
27	−16	10	−1.98	166.93
−23	9	10	136.68	189.64
−26	12	10	−64.71	167.98
−18	6	12	−74.09	185.28

23	12	−4	54.01	176.83
15	10	−4	106.04	127.44
29	8	−3	0.00	175.91
−23	31	−3	−28.13	172.47
30	2	−2	0.00	185.15
−21	23	−2	−17.17	127.97
−30	31	−1	−9.51	198.75
−18	16	−1	−4.36	140.38
30	−6	0	−189.64	158.87
−30	27	0	−3.70	189.38
28	−9	1	−16.51	159.13
−5	−1	2	1559.90	145.40
−34	27	2	−21.39	209.84
−25	14	3	126.38	151.34
−28	14	6	62.86	192.81
33	−16	7	81.61	176.57
23	−16	9	−94.82	146.72
19	−15	10	0.00	135.23
−8	20	−4	0.00	66.43
23	5	−3	16.64	165.08
−7	14	−3	1230.15	125.99
−15	21	−3	15.19	102.74
31	3	−2	155.17	190.56
22	0	−2	261.88	146.06
−25	28	−2	−54.81	142.10
−10	9	−1	23.64	74.35
−21	19	−1	−21.53	126.38
−19	14	0	214.20	149.89
−22	17	0	36.58	129.95
22	−9	1	83.46	146.72
−27	20	1	318.27	153.32
34	−10	2	84.39	191.88
16	−10	2	174.58	129.16
−32	22	3	−29.58	199.94
−19	8	3	98.39	146.72
29	−14	4	0.26	172.34
24	−16	7	−14.26	157.42
17	−15	8	−127.44	133.25
−6	−5	8	156.89	106.31
32	−16	9	0.00	181.72
−20	6	10	−35.39	172.60
−9	−2	11	−60.22	140.25
3	13	−4	102.08	78.97
−29	29	−1	98.25	186.73
32	−8	1	95.88	203.11
25	−9	1	−1.85	141.17
−15	8	1	0.53	104.99
27	−11	2	0.00	145.14
24	−11	2	251.71	142.10
−29	20	2	−75.27	134.57
33	−12	3	35.92	197.56
−28	17	3	14.26	144.21
26	−14	4	117.80	144.34
29	−15	5	82.80	187.92

26	−15	5	−69.07	158.21
18	−14	5	121.89	116.21
−31	18	5	165.08	197.70
9	−12	6	85.97	73.43
−14	1	6	53.22	122.55
−17	3	7	−12.15	142.23
19	11	−4	7.13	176.30
1	14	−4	3.43	63.65
−12	18	−3	35.66	114.23
−18	24	−3	133.51	119.52
26	1	−2	−80.16	171.15
−7	10	−2	3125.88	265.97
26	−3	−1	150.15	146.19
−11	7	0	753.28	104.86
−33	25	2	−84.92	198.49
11	−10	3	108.29	87.82
18	−13	4	150.02	111.46
−31	19	4	0.00	201.66
−9	−2	5	−32.22	115.16
−19	5	6	95.22	135.89
30	−16	7	24.17	164.02
14	−14	7	139.06	103.67
−26	11	8	110.01	166.93
−10	−2	10	−68.54	132.85
24	6	−3	−122.29	179.21
−22	29	−3	−0.13	146.85
31	−5	0	−6.21	183.70
27	−6	0	156.36	149.36
18	−6	0	408.86	148.17
2	−1	0	−4.23	9.77
−36	36	0	40.15	183.43
−29	25	0	−47.01	163.23
−16	11	0	482.55	127.31
12	−7	1	608.01	98.78
−10	4	1	1462.31	150.55
−35	24	4	27.20	178.28
−31	17	6	241.67	174.06
27	−16	7	21.26	158.74
31	−16	8	147.38	173.79
−23	8	8	27.47	163.89
−29	14	8	−24.56	171.15
29	−16	9	0.00	164.02
26	−16	9	−91.91	161.77
18	−15	9	−45.96	126.51
−16	2	9	−9.64	144.74
−24	9	9	128.76	172.21
−27	12	9	−178.81	183.30
18	−14	11	−20.60	127.70
−16	3	11	−172.08	161.64
10	11	−4	31.03	109.48
20	5	−3	1.72	130.08
14	5	−3	174.85	127.04
9	6	−3	210.24	114.50
17	−3	−1	1229.49	191.62

13	−5	0	639.18	114.89
−35	34	0	−58.77	167.72
−36	32	1	−117.80	178.15
35	−9	2	−85.18	185.55
−9	0	3	201.53	91.39
33	−13	4	250.26	177.75
−16	4	4	152.93	130.48
33	−15	6	56.39	171.68
21	−15	6	88.22	136.42
−7	−4	6	272.57	99.97
−12	−1	7	0.79	115.95
−25	10	7	31.30	166.66
−28	13	7	−127.31	171.68
−24	10	11	69.20	174.58
−8	−2	12	8.58	135.49
−15	3	12	112.91	163.76
17	5	−3	116.08	128.89
−30	35	−2	−14.13	165.47
−24	26	−2	−29.71	120.31
23	−3	−1	92.18	143.42
12	−2	−1	776.39	119.78
−28	27	−1	−46.49	155.96
−34	32	0	−106.04	183.43
33	−7	1	248.27	210.64
31	−10	2	0.79	187.53
−22	12	2	−132.19	134.83
30	−12	3	−48.73	165.60
−24	11	4	10.04	141.44
11	−12	5	−44.24	84.25
−16	3	5	−13.60	122.55
−27	13	5	−46.09	179.87
−22	7	7	−115.55	177.23
28	−16	8	−8.06	168.77
−15	1	8	37.24	141.57
20	12	−4	−70.26	177.62
16	11	−4	−169.83	134.44
25	7	−3	−12.15	189.90
27	2	−2	131.93	188.72
23	1	−2	31.56	140.38
24	−6	0	207.34	132.59
21	−6	0	73.56	156.10
29	−8	1	−177.89	153.98
17	−8	1	50.18	125.46
−35	30	1	104.86	187.92
−30	23	1	167.72	160.06
−23	15	1	163.76	135.23
−14	5	2	−64.58	111.06
−25	15	2	287.10	143.55
34	−11	3	0.00	194.00
21	−12	3	−22.85	120.57
−31	20	3	128.63	184.23
−27	14	4	59.56	149.36
33	−14	5	133.25	184.36
−34	21	5	75.01	187.79

16	−14	6	−21.53	106.44
34	−15	7	92.05	166.93
19	−15	7	−19.55	148.83
−20	5	8	107.37	156.36
−21	6	9	−4.49	158.08
12	−13	10	15.45	90.20
−17	3	10	73.95	151.21
−21	7	11	−0.26	169.17
−20	7	12	−16.64	174.19
−29	33	−2	−96.80	175.11
−17	18	−2	13.60	130.34
32	0	−1	105.25	196.51
32	−4	0	107.37	205.62
−33	30	0	190.30	181.45
−20	12	1	400.41	159.00
−32	23	2	45.83	200.73
−35	25	3	66.95	187.00
−16	5	3	384.83	151.87
−34	22	4	−185.02	170.36
30	−15	6	0.00	160.59
−31	16	7	−23.51	170.89
−8	−4	8	23.37	111.46
31	−15	10	0.00	169.04
−25	10	10	−280.50	178.94
21	13	−4	0.00	163.62
13	11	−4	118.06	123.48
26	8	−3	−17.56	168.11
−21	27	−3	7.66	118.72
28	3	−2	−92.18	201.13
−20	21	−2	64.97	120.70
27	−2	−1	103.67	140.51
−35	38	−1	132.33	169.70
−34	36	−1	−36.45	162.04
−28	23	0	110.80	147.91
−26	18	1	77.26	144.74
7	−7	2	7138.95	587.01
−19	9	2	−8.45	155.30
−28	18	2	0.00	152.27
27	−12	3	127.04	147.78
24	−12	3	30.24	141.97
11	−11	4	300.31	108.95
−30	17	4	144.47	181.85
−21	7	5	366.21	143.42
−30	16	5	184.36	189.77
−24	9	6	140.51	186.73
−27	12	6	0.00	182.38
11	−13	9	51.64	88.08
22	−15	10	0.00	136.29
−11	−1	11	−138.80	138.80
28	10	−3	0.00	163.49
27	9	−3	16.90	161.25
21	6	−3	118.72	134.17
20	1	−2	103.54	137.21
−28	31	−2	−1.98	174.19

-33	34	-1	0.00	162.57
28	-5	0	14.39	147.64
34	-6	1	-35.66	179.87
-34	28	1	90.86	174.72
28	-10	2	57.05	136.02
19	-10	2	156.10	147.38
-7	0	2	9119.21	750.50
-24	12	3	68.14	138.27
-27	15	3	-29.45	139.98
30	-13	4	-26.68	187.13
27	-15	6	-0.66	168.51
24	-15	6	0.00	161.91
-22	7	10	-114.63	167.06
-13	24	-4	11.75	91.39
29	4	-2	0.00	175.11
17	1	-2	22.98	126.38
-32	32	-1	53.09	176.96
-27	25	-1	65.11	141.44
33	-3	0	166.79	180.00
-32	28	0	-44.37	184.36
26	-8	1	11.62	132.46
32	-9	2	124.40	186.21
-36	28	2	155.04	174.45
35	-10	3	52.03	167.06
16	-11	3	97.20	129.68
34	-12	4	-37.51	165.74
21	-13	4	43.18	124.27
30	-14	5	33.02	180.66
21	-14	5	137.21	120.44
34	-14	6	68.28	171.28
-16	2	6	23.64	124.14
-30	15	6	-167.85	172.34
-19	4	7	156.10	165.34
28	-15	10	-16.77	154.12
-12	-1	10	25.22	132.06
17	12	-4	72.63	146.98
30	5	-2	-1.19	173.13
-14	15	-2	-15.05	112.38
-23	24	-2	-86.63	126.78
-20	17	-1	50.05	141.97
-21	15	0	48.20	151.47
23	-8	1	179.60	127.57
20	-8	1	71.05	155.44
-29	21	1	-80.29	142.49
14	-9	2	74.09	103.01
-34	23	3	-12.55	189.24
-21	9	3	52.69	137.08
-11	-1	5	7.26	106.97
-33	19	5	-15.05	166.53
31	-15	7	-79.37	158.34
32	-15	8	-136.42	171.94
25	-15	10	-32.35	147.64
11	-12	11	129.82	85.71
-18	4	11	-111.33	148.04

−10	−1	12	−25.49	126.65
8	12	−4	−22.58	98.39
−16	27	−4	143.29	107.50
−25	32	−3	65.77	172.21
24	2	−2	−33.54	143.55
−27	29	−2	−121.10	163.36
−23	20	−1	164.81	131.80
30	−7	1	208.52	156.62
−17	9	1	60.75	130.61
25	−10	2	−57.18	140.51
22	−10	2	80.82	121.10
−31	21	2	197.43	182.38
31	−11	3	0.26	188.05
−30	18	3	112.38	174.58
27	−13	4	−17.30	141.04
24	−13	4	27.07	131.00
−33	20	4	−133.65	181.72
34	−13	5	159.27	159.66
27	−14	5	−1.19	166.79
−9	−3	6	316.55	108.69
−21	6	6	74.75	142.76
−14	0	7	226.22	125.72
20	−15	8	−52.16	133.78
−25	9	8	−61.54	153.32
−28	12	8	−73.82	156.23
−18	3	9	−153.72	148.04
−17	4	12	7.53	158.34
22	7	−3	−23.11	144.08
18	6	−3	239.56	135.89
12	6	−3	130.87	131.00
7	7	−3	1495.46	162.44
−17	22	−3	115.29	110.14
24	−2	−1	96.93	142.10
−31	30	−1	54.28	179.07
−12	10	−1	837.40	112.25
−13	8	0	599.42	112.78
−35	26	2	121.10	174.19
−11	1	3	224.37	110.27
−18	5	4	153.06	130.87
24	−14	5	10.17	125.46
−33	18	6	−36.45	159.79
22	−15	7	0.00	158.21
−27	11	7	−22.85	160.06
8	−12	8	7.00	63.52
30	−15	9	−49.39	159.40
21	−15	9	−20.21	127.70
−26	10	9	175.64	166.40
21	−14	11	0.00	136.82
15	−2	−1	703.36	138.00
29	−4	0	−62.20	155.44
25	−5	0	133.65	132.06
16	−5	0	618.05	137.48
−31	26	0	132.33	185.02
−18	12	0	265.71	140.12

10	−6	1	1465.75	147.25
−12	5	1	782.99	118.59
16	−13	5	0.00	103.80
−18	4	5	275.08	132.06
28	−15	7	−12.68	150.68
−1	−8	7	82.14	65.50
−30	14	7	82.27	161.25
15	−14	8	0.40	126.25
−10	−3	8	0.00	123.21
−17	2	8	141.97	139.06
17	−14	10	−57.45	118.85
−19	4	10	−76.46	148.96
18	13	−4	70.39	172.08
14	12	−4	−65.90	121.89
15	6	−3	−30.37	117.93
−24	30	−3	0.00	157.55
−14	19	−3	71.84	104.99
−20	25	−3	95.35	119.52
−9	11	−2	955.07	111.06
11	−4	0	951.76	120.97
33	−8	2	−61.67	182.90
35	−11	4	3.43	157.15
16	−12	4	96.14	113.31
−26	12	4	−108.82	139.46
−29	14	5	−93.37	182.24
25	−15	7	170.09	152.66
−24	8	7	−30.90	163.62
−22	6	8	20.21	150.81
−23	7	9	−67.88	155.96
−13	0	11	57.71	133.78
−23	8	11	−174.06	170.49
−22	8	12	−10.43	171.15
11	12	−4	64.84	109.61
23	8	−3	−133.51	178.02
−9	15	−3	188.32	83.46
25	3	−2	49.65	155.96
21	−2	−1	−84.25	147.78
10	−1	−1	0.00	80.56
−30	28	−1	60.22	174.19
−26	23	−1	62.46	136.82
31	−6	1	45.03	184.23
−32	24	1	0.13	177.23
−25	16	1	−48.47	136.82
29	−9	2	112.38	158.08
−16	6	2	305.46	129.55
−24	13	2	−79.37	137.21
−27	16	2	−86.63	150.68
28	−11	3	52.82	140.78
9	−9	3	188.05	77.92
−33	21	3	17.70	190.17
31	−12	4	21.79	181.19
−29	15	4	−19.55	187.26
31	−14	6	0.00	152.00
−3	−7	7	262.14	80.69

27	−15	9	43.18	145.93
24	−15	9	99.18	141.44
24	−14	11	−0.92	144.74
19	14	−4	30.24	170.36
−10	21	−4	184.89	79.10
21	2	−2	187.66	145.66
−32	36	−2	−92.57	151.87
−26	27	−2	23.24	140.51
30	1	−1	3.17	182.11
18	−2	−1	0.00	159.13
22	−5	0	135.89	139.06
19	−5	0	129.55	157.15
−36	33	0	4.23	158.21
27	−7	1	21.92	144.87
15	−7	1	84.12	117.53
−37	31	1	21.79	178.28
−22	13	1	33.54	150.15
32	−10	3	−36.18	182.11
−18	6	3	41.07	152.00
−23	9	4	340.19	147.64
31	−13	5	−263.07	167.06
−23	8	5	49.26	133.12
19	−14	6	54.94	112.65
−14	0	10	−15.05	130.87
27	12	−3	36.85	167.32
24	9	−3	0.00	180.92
−31	34	−2	−215.00	160.98
25	−1	−1	119.78	141.04
30	−3	0	113.31	173.53
−28	19	1	46.62	135.49
34	−7	2	162.30	181.32
−9	1	2	2181.25	201.26
−34	24	2	35.26	175.64
−21	10	2	120.97	142.36
−30	19	2	87.56	146.85
19	−11	3	93.76	133.12
−32	17	5	129.68	168.38
−26	10	6	90.07	176.83
−29	13	6	115.82	176.43
32	−14	7	167.19	159.13
12	−13	7	69.46	87.69
1	−9	7	0.00	47.28
16	−14	9	−0.13	108.42
−24	8	10	21.00	157.02
−12	0	12	15.72	137.48
26	11	−3	151.34	163.23
25	10	−3	−17.17	161.38
19	7	−3	146.32	133.91
26	4	−2	−1.85	179.47
−19	19	−2	286.57	127.31
−22	22	−2	85.84	126.91
31	2	−1	195.58	167.72
−36	37	−1	37.64	165.34
−4	2	0	466.70	55.47

−35	31	0	−28.13	159.53
−30	24	0	38.30	156.36
−36	29	1	−96.01	167.59
−26	13	3	54.94	140.12
−29	16	3	−43.18	147.64
−32	18	4	−30.11	180.53
−13	0	5	159.79	111.20
−11	−2	6	237.84	113.31
−18	3	6	35.00	122.42
−32	16	6	194.92	160.72
−5	−6	7	68.14	85.18
−21	5	7	−81.48	164.68
16	−13	11	78.71	113.97
−23	28	−3	160.98	134.57
27	5	−2	−3.04	186.60
18	2	−2	−19.94	126.65
15	2	−2	119.65	149.36
−35	35	−1	142.76	155.04
31	−2	0	216.32	191.75
26	−4	0	114.89	151.61
32	−5	1	41.60	187.39
25	−11	3	41.20	131.66
28	−14	6	36.71	159.53
14	−13	6	146.46	98.78
17	−14	7	181.98	122.95
−16	1	7	−31.56	128.10
−20	4	9	−19.15	134.97
29	−14	10	104.20	150.81
−20	5	11	7.66	148.70
15	13	−4	83.73	135.10
6	13	−4	121.76	91.78
−30	32	−2	−54.67	169.96
−29	26	−1	138.00	150.42
−34	29	0	101.55	164.28
24	−7	1	252.76	143.68
−19	10	1	490.21	151.47
26	−9	2	35.52	142.23
17	−9	2	−34.07	130.08
22	−11	3	20.34	135.23
−13	2	3	36.45	115.03
−36	24	3	251.58	161.51
−23	10	3	133.12	141.57
28	−12	4	−53.09	149.63
9	−11	5	24.30	80.16
7	−11	6	168.38	61.80
−23	7	6	94.42	167.85
−12	−2	8	6.34	126.91
−27	10	8	80.82	154.51
−30	13	8	−90.99	160.19
31	−14	9	1.98	156.10
29	7	−2	134.83	156.23
28	6	−2	107.76	161.77
22	3	−2	6.87	132.59
−16	16	−2	388.92	133.91

−25	25	−2	−93.63	127.31
−34	33	−1	−172.21	155.17
−22	18	−1	87.16	123.61
32	−1	0	−56.52	171.68
−23	16	0	280.50	135.63
−26	19	0	316.55	143.42
33	−4	1	154.78	174.06
18	−7	1	509.49	145.80
−31	22	1	98.78	164.81
30	−8	2	103.93	154.64
−33	22	2	61.67	175.64
33	−9	3	7.66	177.75
−32	19	3	0.00	183.96
32	−11	4	0.00	178.55
−20	6	4	196.64	137.08
−35	21	4	121.10	159.93
28	−13	5	−131.80	171.94
32	−13	6	−48.07	146.72
25	−14	6	124.27	167.32
22	−14	6	−131.00	135.63
−19	3	8	−35.26	142.89
−28	11	9	38.17	158.47
−21	5	10	17.43	146.19
−15	1	11	71.18	133.78
−15	25	−4	3.57	93.76
20	8	−3	86.50	128.89
16	7	−3	248.80	129.68
10	7	−3	221.73	128.10
5	8	−3	286.31	90.20
−14	11	−1	1346.76	152.13
−25	21	−1	3.04	132.06
−15	9	0	208.79	117.01
28	−6	1	−78.05	145.66
−14	6	1	193.87	109.74
14	−10	3	297.40	118.19
19	−12	4	143.15	115.82
32	−12	5	−83.20	164.81
19	−13	5	−36.32	114.63
−20	5	5	63.13	126.12
29	−14	7	−31.17	144.34
−7	−5	7	143.42	93.10
−29	12	7	47.94	166.93
−32	15	7	0.00	152.40
20	−14	10	−92.84	125.99
−27	33	−3	−40.94	156.62
−19	23	−3	263.99	120.97
−29	30	−2	222.13	174.85
22	−1	−1	38.30	133.78
−33	31	−1	60.48	166.79
−20	13	0	90.07	152.93
23	−9	2	−9.11	125.99
20	−9	2	−29.05	149.89
12	−8	2	134.17	89.14
5	−6	2	1180.23	111.06

−37	27	2	148.17	167.85
29	−10	3	−93.63	149.76
25	−12	4	−106.31	128.23
9	−10	4	0.00	84.78
33	−13	7	−90.59	157.42
−26	9	7	−2.11	156.36
−24	7	8	−22.19	145.14
−25	8	9	44.64	153.19
26	−14	10	15.32	136.82
−16	1	10	−14.79	131.40
−14	1	12	−33.54	122.82
16	14	−4	0.00	155.96
12	13	−4	105.12	115.16
−18	28	−4	−142.10	112.38
13	7	−3	5.28	113.84
−11	12	−2	203.24	86.24
27	−3	0	−53.62	153.45
23	−4	0	286.44	138.66
−6	3	0	48.86	92.44
−33	27	0	16.64	185.68
−11	2	2	1016.34	129.16
−18	7	2	301.63	145.40
34	−8	3	−118.06	164.02
22	−12	4	170.89	129.16
−28	13	4	−64.45	162.44
25	−13	5	63.92	134.31
−31	15	5	18.49	150.68
23	−14	10	18.49	134.44
9	13	−4	114.23	107.63
21	9	−3	51.64	153.45
23	4	−2	−82.41	143.29
13	−1	−1	439.10	117.27
−28	24	−1	174.72	136.02
14	−4	0	1288.52	153.85
−27	17	1	−106.31	138.40
31	−7	2	0.00	176.17
−26	14	2	0.00	137.08
−29	17	2	−18.75	153.06
−20	7	3	51.64	129.68
−35	22	3	−77.39	168.64
−25	10	4	−105.52	139.59
−31	16	4	−3.43	180.66
22	−13	5	17.43	119.25
−25	9	5	−61.67	149.49
−13	−1	6	110.80	115.29
28	−14	9	8.45	146.06
−26	9	10	75.54	151.34
17	15	−4	−172.34	161.64
26	14	−3	171.28	164.42
−11	16	−3	65.90	101.82
−26	31	−3	7.13	163.36
−16	20	−3	−8.19	101.95
−22	26	−3	72.24	121.50
19	−1	−1	−37.51	151.21

−32	29	−1	−160.98	178.68
29	−5	1	−51.64	143.68
−24	14	1	328.83	140.12
−15	1	5	−52.56	115.55
−34	18	5	123.48	154.91
−20	4	6	0.00	130.34
−28	11	6	13.34	166.26
−31	14	6	139.85	158.74
26	−14	7	27.20	149.63
20	−14	7	0.00	138.80
18	−14	8	144.87	131.53
19	−14	9	30.64	115.82
10	−12	10	21.13	77.65
−3	−7	10	−15.98	83.59
−22	6	11	115.55	148.96
25	13	−3	3.83	143.95
22	10	−3	16.77	165.08
19	3	−2	72.37	132.06
−28	28	−2	−56.26	155.04
28	2	−1	−110.80	180.66
8	0	−1	179.47	79.37
20	−4	0	74.61	156.10
9	−3	0	541.85	98.39
−30	20	1	57.84	138.27
27	−8	2	−119.91	151.61
−36	25	2	−9.64	162.17
−23	11	2	114.76	125.19
−32	20	2	−30.90	167.32
−28	14	3	101.69	135.63
29	−11	4	−30.11	162.17
−34	19	4	180.92	165.74
33	−11	5	99.18	163.49
33	−12	6	13.34	147.12
29	−13	6	−88.74	155.44
−9	−4	7	22.05	98.78
−23	6	7	91.12	164.94
−14	−1	8	71.71	129.29
−5	−6	10	−29.98	95.74
19	−13	11	57.71	120.57
−12	22	−4	191.75	87.29
24	12	−3	99.31	160.45
23	11	−3	102.48	163.10
24	5	−2	2.64	152.53
−21	20	−2	182.77	115.82
16	−1	−1	71.71	131.93
28	−2	0	3.30	151.21
17	−4	0	267.82	140.78
−37	32	0	13.87	152.66
−32	25	0	−105.12	173.92
25	−6	1	−40.01	131.00
8	−5	1	43.18	75.14
30	−9	3	32.49	162.44
26	−10	3	83.33	129.42
−15	3	3	31.17	129.42

−31	17	3	−167.72	177.49
14	−12	5	112.25	96.14
−25	8	6	−147.25	164.28
23	−14	7	61.01	145.40
25	−14	9	48.47	141.57
30	−13	10	−164.55	153.32
25	−13	11	−17.30	142.63
−4	−6	11	0.00	89.27
−17	2	11	35.66	130.87
17	8	−3	31.69	127.70
−33	35	−2	0.00	146.06
−24	23	−2	63.65	126.51
30	4	−1	−105.78	165.47
29	3	−1	43.98	179.07
23	0	−1	208.39	139.98
−3	4	−1	802.93	83.59
−5	5	−1	540.26	85.05
−8	4	0	412.82	92.18
13	−6	1	26.02	90.46
−21	11	1	408.33	161.51
32	−6	2	56.13	177.75
−25	11	3	131.66	139.59
14	−11	4	250.78	126.25
29	−12	5	1.98	175.11
−21	4	8	35.79	142.36
−29	11	8	−9.64	148.17
22	−14	9	−97.33	131.93
9	−12	9	−8.19	76.07
−1	−8	10	8.45	70.78
−7	−5	10	−38.69	101.16
−23	6	10	−9.77	145.80
22	−13	11	0.00	130.21
−2	−7	11	−70.52	79.76
−6	−5	11	190.30	102.74
13	14	−4	−50.98	125.46
−25	29	−3	−106.57	152.27
25	6	−2	37.64	163.89
16	3	−2	150.81	138.80
−32	33	−2	64.58	150.29
−37	36	−1	−45.96	148.30
−31	27	−1	−104.99	169.96
−25	17	0	0.00	133.65
30	−4	1	13.21	173.26
−16	7	1	266.76	125.85
−22	7	4	68.54	131.14
−22	6	5	30.51	131.80
30	−13	7	−72.90	143.55
−31	13	7	−35.52	167.98
24	−14	8	0.00	129.68
21	−14	8	−49.65	134.57
15	−13	10	1.85	102.35
−18	2	10	−90.73	134.70
4	14	−4	221.99	82.14
28	9	−2	209.45	152.27

26	7	−2	−205.49	176.43
13	3	−2	394.47	134.31
−18	17	−2	141.17	135.76
−36	34	−1	141.83	153.32
−16	12	−1	662.15	130.61
−24	19	−1	−90.73	129.29
29	−1	0	−102.22	163.62
24	−3	0	125.59	139.59
−36	30	0	0.00	158.87
−17	10	0	948.07	148.83
−33	23	1	24.17	184.49
33	−5	2	−78.71	173.00
−13	3	2	299.91	108.16
−35	23	2	1.72	160.45
23	−10	3	4.09	126.65
17	−10	3	336.89	134.44
−34	20	3	−46.22	161.38
34	−10	5	−62.99	145.66
17	−13	6	57.58	105.78
−28	10	7	144.21	159.53
13	−13	8	36.98	113.31
−26	8	8	131.66	146.59
27	8	−2	0.00	167.59
20	4	−2	278.91	138.00
−27	26	−2	77.78	134.31
−27	22	−1	83.59	135.49
−22	14	0	299.51	145.80
22	−6	1	125.99	130.87
−37	28	1	0.92	165.21
24	−8	2	118.59	145.80
26	−11	4	−39.09	130.48
26	−13	6	216.98	164.02
−15	0	6	114.36	116.08
−11	−3	7	205.62	105.78
−9	−4	10	66.16	107.76
−8	−4	11	78.18	104.59
18	9	−3	−29.32	127.97
−13	13	−2	550.69	109.35
−31	31	−2	−8.32	157.55
−7	6	−1	233.35	74.09
−35	32	−1	−25.49	159.27
30	0	0	0.00	179.87
31	−3	1	−12.41	179.74
28	−7	2	131.40	134.17
−20	8	2	190.30	152.93
31	−8	3	34.86	171.94
20	−10	3	−92.71	128.63
7	−8	3	155.57	63.52
−22	8	3	253.43	117.14
−30	14	4	6.74	189.90
−17	2	5	−201.79	130.48
−16	0	8	−26.02	129.82
29	−13	9	−1.06	146.06
27	−13	10	55.99	139.46

14	15	−4	12.41	134.31
−17	26	−4	−22.05	101.69
14	8	−3	188.58	117.53
8	8	−3	201.13	108.95
−21	24	−3	139.59	115.82
20	0	−1	39.49	131.80
31	1	0	−118.46	177.62
−35	28	0	17.83	165.74
−31	23	0	252.10	165.21
32	−2	1	81.61	175.11
26	−5	1	41.34	140.12
15	−8	2	18.09	111.20
−28	15	2	−94.29	138.93
−31	18	2	0.00	157.55
−27	11	4	−19.28	145.80
−33	17	4	82.67	185.41
26	−12	5	−57.84	153.72
−27	10	5	−31.83	166.66
30	−12	6	0.00	146.98
−22	5	6	0.00	148.30
−20	3	7	0.00	156.36
14	−13	9	0.00	100.10
−4	−7	9	0.00	91.39
−6	−6	9	−164.28	103.54
−24	7	11	−20.60	151.34
10	14	−4	208.79	111.20
−20	29	−4	−87.56	119.38
3	9	−3	587.80	88.08
−13	17	−3	146.98	111.86
−18	21	−3	155.17	106.84
−30	25	−1	72.37	148.70
−10	5	0	75.54	85.71
19	−6	1	141.70	157.15
16	−6	1	−2.25	111.46
−26	15	1	5.55	143.81
−29	18	1	24.17	140.12
−25	12	2	139.06	136.15
27	−9	3	−22.45	129.95
−17	4	3	163.76	157.42
30	−11	5	160.72	160.32
23	−13	6	−41.73	137.74
20	−13	6	−1.85	118.85
−30	12	6	46.49	152.00
−33	15	6	125.06	149.10
15	−13	7	98.91	101.16
−25	7	7	157.81	152.40
−19	3	11	0.00	130.21
15	16	−4	−69.99	161.11
7	14	−4	232.96	101.16
19	10	−3	0.00	134.31
11	8	−3	167.19	122.68
−28	32	−3	146.98	151.21
−24	27	−3	0.00	129.55
25	2	−1	0.00	116.74

−34	30	−1	−44.90	165.21
25	−2	0	96.27	141.31
21	−3	0	37.24	137.61
32	−7	3	127.57	176.57
−30	15	3	124.80	154.78
23	−11	4	52.69	139.59
17	−11	4	235.20	110.67
31	−12	7	−27.73	144.61
27	−13	7	29.45	138.80
28	−13	8	90.86	142.49
−23	5	8	−39.49	136.42
−8	−5	9	−25.09	107.76
−11	−3	10	−45.56	114.76
9	−11	11	0.00	72.11
−10	−3	11	73.56	113.31
24	15	−3	−28.53	144.47
21	5	−2	133.38	132.46
−30	29	−2	7.40	165.74
−9	7	−1	1153.42	122.02
−5	0	1	745.09	104.59
−32	21	1	108.55	168.11
29	−6	2	0.92	142.49
18	−8	2	204.17	146.46
−34	21	2	59.69	181.06
−27	12	3	5.02	134.57
−33	18	3	10.30	172.08
−6	−4	4	272.97	96.27
−36	20	4	62.60	153.32
17	−12	5	183.56	114.10
12	−12	6	145.66	93.37
−27	9	6	−22.85	159.66
10	−12	7	48.07	75.54
−13	−2	7	228.73	113.04
−2	−8	9	11.23	74.88
24	−13	10	−107.89	136.02
−20	3	10	−68.54	133.91
−25	7	10	−96.27	150.02
14	−12	11	0.79	103.01
0	−8	11	1.85	51.90
−14	23	−4	−5.81	90.99
23	14	−3	219.49	146.85
20	11	−3	125.46	157.94
17	4	−2	177.62	125.72
−23	21	−2	−115.03	125.99
11	0	−1	1358.51	148.30
−34	26	0	0.00	164.68
−7	1	1	292.12	81.88
−18	8	1	20.07	143.95
−23	12	1	124.01	149.10
−15	4	2	222.79	122.02
12	−9	3	290.80	102.08
20	−11	4	96.67	117.93
−4	−5	4	460.76	86.10
−24	8	4	−62.60	130.87

23	−12	5	80.16	125.19
−24	7	5	−25.62	136.29
−17	1	6	−142.10	114.50
−31	12	8	−46.62	144.87
18	−13	10	40.54	110.40
26	−12	11	41.73	142.63
22	13	−3	−63.65	159.00
21	12	−3	37.37	163.89
−35	36	−2	−25.75	144.34
−26	24	−2	−42.00	123.87
26	3	−1	31.03	159.00
17	0	−1	561.79	161.77
−18	13	−1	21.00	134.17
12	−3	0	638.12	109.87
−19	11	0	190.17	143.15
−27	18	0	17.30	135.76
27	−4	1	17.04	131.27
33	−6	3	20.87	167.59
−8	−3	4	63.52	102.88
20	−12	5	−124.40	121.10
−28	9	8	0.00	146.59
26	−13	9	−26.02	138.93
−10	−4	9	143.55	118.19
21	−13	10	−71.45	125.59
15	9	−3	104.46	121.63
−27	30	−3	−29.19	156.36
22	6	−2	91.78	133.25
−20	18	−2	38.56	115.82
27	4	−1	−7.40	178.41
−33	28	−1	63.92	166.00
−26	20	−1	79.37	128.76
26	−1	0	0.00	138.00
18	−3	0	105.91	138.40
−12	6	0	204.43	86.24
23	−5	1	−10.56	121.23
−35	24	1	−15.45	169.57
25	−7	2	57.97	136.95
10	−7	2	3206.97	278.12
−36	21	3	−191.75	168.77
31	−10	5	181.58	156.62
−19	3	5	208.26	126.78
31	−11	6	4.09	150.02
24	−13	7	−94.95	146.06
−30	11	7	0.00	151.74
−18	1	8	137.34	133.65
30	−12	9	−20.73	146.32
−12	−2	11	5.02	116.61
11	15	−4	−23.37	112.25
27	11	−2	−43.18	142.89
−34	34	−2	−96.80	155.44
−15	14	−2	37.24	118.72
29	6	−1	−16.77	157.02
28	5	−1	129.82	187.92
21	1	−1	3.83	124.93

14	0	−1	1910.66	204.69
−23	17	−1	−132.59	125.59
−29	23	−1	62.86	132.33
−38	31	0	−17.43	156.36
−24	15	0	−11.89	126.91
−9	2	1	1295.25	139.06
30	−5	2	−92.05	176.83
−22	9	2	57.31	126.38
−37	24	2	−98.65	162.57
28	−8	3	124.67	142.63
24	−9	3	−44.90	141.44
−24	9	3	208.66	137.61
−6	−5	5	−63.65	92.71
27	−12	6	−23.64	159.79
32	−11	7	−73.03	146.85
−22	4	7	5.81	147.12
25	−13	8	74.61	137.08
28	−12	10	34.20	140.64
−13	−2	10	120.44	114.50
−21	4	11	−9.24	134.57
26	10	−2	44.11	150.81
23	7	−2	45.56	142.36
−29	27	−2	53.48	167.72
−11	8	−1	285.25	85.97
15	−3	0	410.45	123.48
28	−3	1	109.21	143.15
−3	−1	1	845.85	93.50
−19	5	3	−7.79	135.10
−29	12	4	−159.00	161.64
−32	15	4	134.31	172.34
27	−11	5	−33.15	161.91
−4	−6	5	152.40	86.10
−8	−4	5	254.35	100.10
−29	11	5	117.53	181.98
−24	6	6	22.05	160.06
18	−13	7	14.13	127.04
−15	−1	7	69.07	122.55
−27	8	7	93.63	146.19
−19	27	−4	−2.64	108.55
−15	18	−3	85.31	105.52
−23	25	−3	−91.78	114.76
25	9	−2	5.68	169.04
24	8	−2	227.28	174.98
14	4	−2	165.60	134.83
−33	32	−2	0.00	145.66
27	0	0	123.74	143.81
22	−2	0	38.56	124.93
−33	24	0	26.02	171.81
31	−4	2	−30.11	175.11
−30	16	2	57.45	140.91
−33	19	2	159.00	169.17
−35	18	4	−4.09	159.53
−32	13	6	41.20	148.17
21	−13	7	−34.20	149.10

29	−12	8	−66.43	148.30
16	−13	8	−39.75	131.14
23	−13	9	102.08	134.57
17	−13	9	123.08	114.10
−12	−3	9	51.11	122.16
23	−12	11	0.00	132.19
−20	22	−3	112.25	117.93
6	1	−1	382.18	78.71
−37	33	−1	19.94	151.08
7	−2	0	0.00	89.14
−37	29	0	−34.86	158.08
−28	16	1	85.97	138.93
−31	19	1	−39.09	151.21
−17	5	2	−24.70	142.49
−27	13	2	−7.92	137.87
33	−7	4	57.45	148.83
32	−9	5	−86.76	150.15
7	−10	5	291.99	78.05
−10	−3	5	16.51	100.23
32	−10	6	44.24	142.23
−19	2	6	−74.61	124.93
28	−12	7	70.92	139.98
−25	6	8	−27.86	141.70
1	−9	10	0.00	31.03
−22	4	10	0.00	129.55
17	−12	11	29.05	113.44
−14	−1	11	9.24	119.38
12	16	−4	79.37	124.14
2	15	−4	25.88	66.95
16	10	−3	54.28	118.19
18	5	−2	62.99	124.67
11	4	−2	136.82	116.48
−32	26	−1	−80.69	163.49
28	1	0	−47.01	162.04
29	−2	1	−197.04	160.98
11	−5	1	38.30	77.92
−11	3	1	267.16	86.76
−20	9	1	161.25	152.66
32	−3	2	41.34	167.06
22	−7	2	61.80	124.40
29	−7	3	0.00	148.70
−6	−3	3	225.82	74.22
−8	−2	3	336.76	92.84
−32	16	3	−71.45	174.45
−2	−6	4	219.62	64.71
−29	10	6	−91.65	152.13
22	−13	8	−0.66	127.97
20	−13	9	−47.81	121.10
−27	8	10	−98.52	143.42
20	−12	11	11.36	124.67
12	9	−3	−77.12	106.18
−26	28	−3	−16.64	146.72
−36	31	−1	0.00	148.04
29	2	0	−62.20	178.28

−14	7	0	1629.63	174.58
−21	12	0	49.52	146.72
30	−1	1	143.02	172.74
20	−5	1	231.90	148.44
−25	13	1	0.00	137.48
−34	22	1	−40.01	195.71
26	−6	2	212.35	137.21
−36	22	2	0.00	163.89
15	−9	3	125.99	121.89
−29	13	3	27.73	137.48
−35	19	3	−108.95	152.53
24	−10	4	118.06	114.63
12	−10	4	0.00	106.84
−26	9	4	42.66	141.57
12	−11	5	5.55	90.07
−21	4	5	35.39	122.42
−26	8	5	23.90	154.78
24	−12	6	−4.62	151.47
19	−13	8	62.73	131.27
−20	2	8	−61.28	134.44
−15	−1	10	7.00	118.19
27	−11	11	−125.19	145.14
8	15	−4	62.20	99.05
−16	24	−4	−24.04	97.46
−30	33	−3	108.16	147.64
−32	30	−2	−138.14	167.59
18	1	−1	−166.26	145.40
−13	9	−1	379.41	99.05
31	0	1	3.04	164.94
24	−4	1	8.06	125.99
−10	−1	3	119.52	102.22
7	−9	4	105.52	75.54
33	−8	5	29.19	141.70
−12	−2	5	138.27	105.52
−30	10	8	60.88	140.64
27	−12	9	55.60	141.83
−14	−2	9	103.01	121.63
13	17	−4	−100.37	148.44
22	16	−3	0.00	139.98
17	11	−3	193.21	122.16
−17	15	−2	−7.66	129.55
−22	19	−2	23.37	117.27
−28	25	−2	−14.53	134.44
−36	27	0	−68.41	154.91
−29	19	0	42.92	132.59
−24	10	2	9.90	136.55
25	−8	3	92.05	130.61
18	−9	3	110.67	134.04
28	−10	5	40.15	163.10
24	−11	5	46.49	129.16
28	−11	6	78.44	151.08
15	−12	6	72.50	102.08
−17	0	7	64.97	127.31
−24	5	7	−26.81	152.79

−32	12	7	−106.97	150.29
25	−12	10	−70.65	131.27
−23	5	11	44.24	135.63
14	18	−4	0.00	145.40
21	15	−3	73.16	150.81
6	9	−3	13.34	95.22
19	6	−2	−58.77	129.95
23	3	−1	134.17	128.50
−28	21	−1	195.32	133.78
23	−1	0	45.69	128.89
−26	16	0	206.41	136.42
17	−5	1	373.07	136.29
6	−4	1	869.23	106.97
−13	4	1	81.22	98.91
30	−6	3	−124.67	194.00
−4	−4	3	387.20	71.58
−21	6	3	142.49	124.93
−26	10	3	80.56	134.44
−26	7	6	145.27	162.83
30	−11	8	74.09	146.59
−7	−6	8	−71.71	91.52
−9	−5	8	65.77	107.89
−16	0	11	−50.71	123.34
5	15	−4	0.00	79.63
20	14	−3	292.38	168.91
18	12	−3	164.28	136.82
9	9	−3	−19.94	119.91
−17	19	−3	168.38	103.54
−29	31	−3	−164.55	154.25
−35	29	−1	−25.09	162.30
−25	18	−1	−22.32	134.31
−31	24	−1	12.68	152.66
19	−2	0	234.28	149.36
14	−5	1	47.28	102.35
−37	25	1	0.00	154.51
27	−5	2	−61.54	137.34
19	−7	2	409.52	156.49
13	−7	2	−26.81	88.74
−19	6	2	159.66	150.42
−12	0	3	191.49	118.72
−34	16	4	56.92	154.12
−31	12	5	47.28	164.94
21	−12	6	32.09	119.65
29	−11	7	0.00	134.57
25	−12	7	79.24	145.53
−29	9	7	−24.17	141.44
26	−12	8	−20.47	134.70
−23	4	9	0.00	147.12
−28	8	9	−146.06	149.89
13	−12	10	−15.98	89.80
19	13	−3	62.86	162.17
26	13	−2	23.77	149.10
−36	35	−2	76.20	149.49
−31	28	−2	−70.39	163.49

24	4	−1	147.51	130.74
−16	8	0	274.55	111.59
25	−3	1	94.29	136.15
−32	17	2	3.96	175.11
21	−10	4	207.34	127.70
−31	13	4	144.47	170.36
−14	−1	5	−26.81	110.40
18	−12	6	−37.51	104.59
−21	3	6	172.87	144.08
−34	14	6	0.00	142.23
−5	−7	8	−6.60	48.99
−11	−4	8	−55.20	115.16
−27	7	8	4.09	136.68
−17	0	10	−177.09	124.67
−24	5	10	0.00	137.48
−7	18	−4	128.10	40.01
−9	19	−4	134.57	52.96
−21	28	−4	105.91	123.74
−25	26	−3	−23.77	122.55
20	7	−2	141.57	126.78
15	5	−2	41.60	139.72
28	8	−1	−9.11	150.29
27	7	−1	−218.16	169.43
25	5	−1	−26.41	150.81
−15	10	−1	289.35	110.54
−35	25	0	66.29	167.19
−22	10	1	296.61	153.45
−30	17	1	−38.96	141.70
−33	20	1	−80.69	171.15
16	−7	2	236.13	120.84
−29	14	2	111.20	139.85
−35	20	2	82.14	170.62
31	−5	3	80.95	175.91
−23	6	4	0.00	129.02
−8	−5	6	−85.05	91.25
11	−12	8	74.48	108.55
−22	3	8	100.37	136.29
0	−9	9	−82.93	30.77
−16	−1	9	−128.50	124.01
13	10	−3	152.66	114.36
−22	23	−3	−34.60	115.55
25	12	−2	−161.38	156.89
−35	33	−2	−5.94	144.21
26	6	−1	0.00	179.47
15	1	−1	174.06	143.95
24	0	0	170.36	129.68
−15	5	1	290.67	112.25
23	−6	2	0.00	125.59
32	−4	3	−48.99	155.04
−34	17	3	60.35	164.55
15	−10	4	147.38	124.80
−28	10	4	130.21	157.28
29	−9	5	−29.32	170.09
21	−11	5	190.70	124.67

−23	5	5	27.86	132.06
−28	9	5	6.07	160.72
29	−10	6	−64.31	149.89
−6	−6	6	53.88	78.31
−10	−4	6	366.73	107.10
−31	11	6	0.53	148.70
−19	1	7	129.29	133.38
22	−12	10	111.33	131.93
24	−11	11	36.85	135.23
9	16	−4	67.09	103.54
−18	25	−4	74.48	102.35
24	11	−2	0.00	158.47
21	8	−2	0.00	129.55
19	2	−1	−36.05	128.36
−34	27	−1	66.43	166.40
−23	13	0	34.73	129.82
21	−4	1	329.62	151.21
−27	14	1	197.70	144.61
28	−4	2	0.00	138.80
26	−7	3	1.98	131.14
−14	1	3	−13.47	127.97
−31	14	3	0.00	162.70
30	−7	4	12.15	170.49
18	−10	4	104.59	113.84
15	−11	5	−27.86	100.76
−13	−3	8	−101.55	120.97
28	−11	9	−86.10	149.23
24	−12	9	−98.78	137.61
−18	1	11	−115.29	130.48
−11	20	−4	3.83	76.20
1	10	−3	550.56	73.43
−28	29	−3	−27.73	151.87
23	10	−2	−16.64	171.94
22	9	−2	0.00	147.51
−19	16	−2	44.64	135.36
−27	23	−2	−45.56	123.21
9	1	−1	−49.39	76.07
25	1	0	1.72	129.16
16	−2	0	0.92	130.48
26	−2	1	−70.65	141.97
−36	23	1	−221.33	168.64
−26	11	2	70.92	138.93
−38	23	2	−167.85	155.44
22	−8	3	165.87	119.25
−37	20	3	167.59	152.93
18	−11	5	94.69	111.06
−16	0	5	273.76	119.52
25	−11	6	−120.84	158.61
−12	−3	6	209.32	106.84
30	−10	7	−40.94	140.25
22	−12	7	−21.66	153.72
13	−12	7	−9.24	90.33
−26	6	7	−42.92	141.44
12	−12	9	−24.17	93.63

8	−11	10	103.67	56.26
−25	6	11	145.14	148.17
−34	31	−2	49.79	152.00
−24	20	−2	36.71	125.46
12	1	−1	421.93	105.52
−38	32	−1	70.12	146.98
10	−2	0	−32.75	83.07
29	−3	2	−8.19	166.13
−21	7	2	267.69	136.29
−23	7	3	62.46	128.89
−28	11	3	0.00	131.00
25	−10	5	24.30	136.42
−28	8	6	4.36	148.70
23	−12	8	−83.20	134.57
−18	0	9	167.59	127.97
−25	5	9	−67.09	141.31
26	−11	10	58.24	136.82
19	−12	10	−12.41	117.27
16	−12	10	103.01	108.95
−19	1	10	−88.08	124.27
−19	20	−3	4.36	105.65
12	5	−2	−87.69	121.23
−30	26	−2	193.47	154.38
−30	22	−1	88.61	141.83
26	2	0	−57.31	139.85
20	−1	0	−53.88	143.95
−18	9	0	−13.47	134.83
−38	28	0	−61.54	154.78
−28	17	0	45.83	130.61
−31	20	0	2.77	129.68
31	−6	4	188.98	172.74
30	−8	5	29.19	159.53
30	−9	6	64.84	154.38
−23	4	6	286.44	160.45
27	−11	8	17.56	136.68
−15	−2	8	76.20	121.23
−26	6	10	0.00	137.34
10	17	−4	62.73	110.93
14	11	−3	175.77	121.89
16	6	−2	6.47	114.23
−17	11	−1	383.90	128.23
−27	19	−1	36.58	128.50
29	5	0	−100.37	159.66
27	3	0	−2.77	171.94
13	−2	0	280.23	98.12
−34	23	0	26.81	191.49
27	−1	1	−22.98	136.02
−17	6	1	358.81	137.87
30	−2	2	62.33	170.49
−16	2	3	285.25	146.46
−33	14	4	24.83	163.89
−36	17	4	0.00	145.40
−33	13	5	−129.02	151.34
5	−10	6	121.89	31.56

−14	−2	6	7.40	110.80
31	−9	7	−24.83	145.14
26	−11	7	−96.27	142.76
−21	2	7	−71.71	150.95
−31	10	7	−38.30	139.06
−24	4	8	108.29	138.14
−29	8	8	80.16	142.23
12	−11	11	75.14	94.95
−13	21	−4	12.02	84.12
20	17	−3	45.43	143.42
−31	32	−3	−75.67	145.00
6	5	−2	471.33	78.18
−10	10	−2	3979.39	338.21
−37	30	−1	−94.42	143.02
−33	25	−1	0.00	178.15
28	0	1	0.00	158.61
−24	11	1	115.55	127.17
31	−1	2	10.83	161.91
24	−5	2	0.00	126.78
−34	18	2	54.41	176.96
27	−6	3	0.00	144.47
10	−8	3	245.77	82.27
32	−5	4	26.02	164.02
−25	7	4	0.00	123.21
−25	6	5	−8.58	145.00
−4	−7	6	335.43	78.58
19	−12	7	−21.13	134.97
−3	−8	8	9.38	78.71
29	−10	9	1.72	138.80
21	−12	9	−36.58	126.91
21	−11	11	30.37	124.80
−20	2	11	−69.60	126.51
11	18	−4	−32.49	133.51
10	10	−3	0.53	105.65
−10	14	−3	352.73	93.10
25	15	−2	−32.49	150.68
−38	36	−2	−171.55	148.04
−33	29	−2	79.90	153.19
30	2	1	−19.15	174.58
29	1	1	17.43	192.28
−32	18	1	78.31	169.57
20	−6	2	−70.52	149.49
−10	0	2	4772.55	405.43
−31	15	2	74.35	150.95
31	−7	5	−2.91	159.13
−18	1	5	242.20	119.65
−30	10	5	−60.48	159.40
10	−11	6	60.88	81.35
−33	12	6	98.78	147.12
16	−12	7	50.05	110.80
12	19	−4	−37.64	148.44
6	16	−4	65.90	97.46
−23	29	−4	141.04	139.72
19	16	−3	11.36	152.66

15	12	−3	−9.51	114.63
−8	13	−3	386.67	76.60
−24	24	−3	136.68	118.72
−27	27	−3	−45.03	145.80
9	5	−2	−23.24	93.10
−12	11	−2	230.18	86.37
−21	17	−2	−58.37	113.57
−25	14	0	8.19	128.50
−37	26	0	0.00	150.95
22	−3	1	56.52	129.82
18	−4	1	716.43	158.87
−29	15	1	10.96	131.80
−35	21	1	104.59	181.32
−8	−1	2	2238.17	203.11
−37	21	2	239.56	155.96
19	−8	3	−24.70	127.70
−33	15	3	76.73	174.32
−36	18	3	−74.09	156.76
−30	11	4	111.06	167.72
31	−8	6	0.00	142.10
22	−11	6	0.00	127.44
−16	−1	6	−124.80	115.55
20	−12	8	106.84	134.31
−17	−1	8	67.09	123.48
7	−11	9	275.61	53.62
−20	1	9	133.12	130.08
−21	2	10	24.17	129.55
25	−10	11	0.00	138.40
−20	26	−4	207.07	111.99
−12	15	−3	52.03	100.63
24	14	−2	−60.48	145.53
−37	34	−2	10.56	145.80
27	10	−1	−13.34	143.55
21	4	−1	234.14	134.83
16	2	−1	46.75	148.96
21	0	0	222.92	131.00
−20	10	0	144.47	142.36
8	−6	2	1247.18	124.01
3	−5	2	421.14	50.45
−12	1	2	270.73	106.44
−23	8	2	−4.62	125.33
−28	12	2	0.00	132.33
28	−5	3	−10.83	145.93
−2	−5	3	469.61	62.60
−18	3	3	55.99	125.99
−25	8	3	−66.56	133.65
32	−6	5	−7.79	153.72
26	−10	6	10.83	159.13
−28	7	7	139.59	145.00
14	−12	8	5.28	122.16
25	−11	9	26.68	137.87
18	−12	9	0.00	104.06
15	−12	9	25.75	108.82
27	−10	10	201.00	145.14

18	−11	11	−18.49	119.91
18	15	−3	62.46	163.89
17	14	−3	38.30	144.21
16	13	−3	−119.91	123.87
17	7	−2	70.26	119.12
−29	24	−2	87.95	139.85
−19	12	−1	268.61	140.91
−36	28	−1	−206.41	159.93
−19	7	1	370.30	155.57
−38	24	1	0.00	148.70
23	−7	3	50.18	135.23
−30	12	3	152.00	154.12
26	−9	5	27.47	158.47
22	−10	5	162.57	125.72
−25	5	6	61.94	167.32
−30	9	6	−96.14	148.83
28	−10	8	−87.29	147.12
−27	6	9	7.00	145.00
23	−11	10	−24.04	133.51
15	−11	11	−72.11	109.61
−15	22	−4	130.48	96.27
−21	21	−3	334.77	125.59
−30	30	−3	−61.67	160.98
−14	12	−2	152.40	105.38
−26	21	−2	61.14	121.63
26	9	−1	−85.84	171.81
22	5	−1	82.41	129.29
25	−4	2	9.77	139.06
−14	2	2	−71.31	117.93
27	−10	7	−34.34	138.80
−23	3	7	0.00	144.74
17	−12	8	−6.47	134.83
−26	5	8	−42.39	140.51
−28	7	10	32.75	148.44
−22	3	11	0.00	131.27
0	16	−4	121.10	60.48
−14	16	−3	113.44	113.84
23	13	−2	50.84	163.10
18	8	−2	40.67	126.25
−36	32	−2	0.00	144.74
−32	27	−2	−101.42	170.75
25	8	−1	29.71	167.59
24	7	−1	0.13	156.10
23	6	−1	59.16	141.31
−29	20	−1	−102.08	137.48
−32	23	−1	−119.91	155.30
17	−1	0	−9.11	140.12
−30	18	0	87.03	134.17
23	−2	1	0.00	131.00
−26	12	1	205.75	143.15
−6	−2	2	1504.84	143.15
29	−4	3	−53.75	163.49
16	−8	3	401.47	134.04
13	−8	3	229.13	107.10

27	−7	4	−50.84	151.47
−27	8	4	32.88	143.42
−20	2	5	73.95	123.34
−18	0	6	4.49	120.04
23	−11	7	−9.77	147.78
8	−11	7	62.73	68.67
−33	11	7	0.00	144.61
24	−11	8	43.98	134.83
−31	9	8	−142.89	152.66
3	16	−4	193.47	81.22
7	10	−3	294.36	107.76
22	12	−2	161.51	179.74
22	1	0	−49.65	137.21
−36	24	0	−19.15	161.91
15	−4	1	252.90	109.08
9	−4	1	617.39	93.90
17	−6	2	221.07	143.15
30	−3	3	0.00	171.42
5	−7	3	188.45	50.18
−35	15	4	−10.17	165.34
−27	7	5	−152.27	161.64
−35	14	5	14.00	147.25
19	−11	6	68.01	111.06
−19	0	8	−40.67	131.66
−22	2	9	108.03	131.53
−23	3	10	−79.63	134.31
21	11	−2	−9.24	159.00
19	9	−2	34.47	124.93
13	6	−2	388.92	146.85
−27	15	0	123.74	136.95
26	−3	2	28.00	132.19
−16	3	2	115.82	125.72
−33	16	2	−115.29	178.81
−36	19	2	24.96	166.26
31	−2	3	0.00	164.42
−20	4	3	67.09	117.14
27	−9	6	162.57	156.36
29	−9	8	−66.82	146.98
7	17	−4	0.00	100.10
11	11	−3	72.77	106.57
4	10	−3	895.24	117.67
−33	33	−3	0.00	132.85
−26	25	−3	50.98	127.70
24	17	−2	33.81	155.83
20	10	−2	−39.88	143.68
−16	13	−2	68.28	121.36
−23	18	−2	205.49	122.02
−21	13	−1	−8.06	148.30
−39	31	−1	56.52	153.85
−35	26	−1	−111.72	160.19
−22	11	0	2.77	157.02
−21	8	1	389.45	162.30
−34	19	1	−132.46	178.94
21	−5	2	0.00	121.89

−25	9	2	69.73	132.72
−27	9	3	48.47	128.23
28	−6	4	−53.22	164.02
−32	12	4	0.00	167.45
27	−8	5	0.00	175.91
−32	11	5	−63.92	152.00
−10	−5	7	81.22	96.67
−12	−4	7	5.15	107.23
7	−10	11	100.63	75.14
−17	23	−4	4.49	97.99
−22	27	−4	−17.43	123.61
−16	17	−3	42.92	118.72
−29	28	−3	−88.61	155.83
−35	30	−2	136.02	149.63
13	2	−1	389.84	120.18
4	2	−1	811.12	101.42
28	7	0	0.00	160.98
23	2	0	94.56	151.08
24	−1	1	0.00	144.47
19	−3	1	214.86	150.95
12	−4	1	196.24	87.56
−31	16	1	−104.86	144.34
−37	22	1	0.00	168.11
−30	13	2	−103.01	138.80
24	−6	3	120.97	145.66
−35	16	3	177.75	176.04
10	−9	4	59.16	98.91
19	−10	5	269.40	121.89
13	−11	6	20.87	95.74
−20	1	6	141.97	139.06
−27	6	6	−40.67	152.79
28	−9	7	70.39	155.96
−14	−3	7	41.20	114.50
−30	8	7	178.55	148.57
26	−10	9	−101.69	144.74
−29	7	9	9.11	141.31
20	−11	10	105.78	129.95
22	−10	11	5.55	131.40
−6	12	−3	272.31	60.09
−23	22	−3	16.24	117.14
27	6	0	−25.49	177.49
−39	27	0	−28.92	149.10
27	−2	2	−21.13	140.64
14	−6	2	181.45	110.14
−18	4	2	279.97	147.25
10	−10	5	29.05	89.93
−22	3	5	0.00	123.21
23	−10	6	98.52	145.00
16	−11	6	14.00	104.59
−32	10	6	136.68	145.66
−8	−6	7	45.83	90.20
−25	4	7	144.74	144.87
−21	1	8	−39.35	133.25
22	−11	9	−42.79	130.34

−24	4	11	−12.55	141.97
−28	22	−2	86.76	130.08
−31	25	−2	237.84	165.47
−38	29	−1	187.13	151.61
25	4	0	0.00	128.50
24	3	0	−30.77	136.55
5	−1	0	968.14	106.04
25	0	1	−7.26	132.85
28	−1	2	0.00	162.30
11	−6	2	470.40	95.48
20	−7	3	136.29	118.85
−32	13	3	43.32	174.45
29	−5	4	0.13	171.81
28	−7	5	−102.22	185.81
23	−9	5	45.43	126.12
−28	6	8	−8.06	148.17
24	−10	10	−93.37	139.85
8	18	−4	−42.00	104.46
18	18	−3	51.77	151.21
12	12	−3	29.58	112.78
−32	31	−3	77.12	149.23
23	16	−2	59.16	154.38
−18	14	−2	13.87	136.55
26	12	−1	0.00	146.06
18	0	0	664.13	178.28
14	−1	0	187.39	109.35
−32	19	0	131.66	160.06
29	4	1	88.08	168.38
−28	13	1	31.03	137.48
30	1	2	10.56	164.15
29	0	2	−94.16	176.57
30	−4	4	0.00	170.89
−29	9	4	−84.52	171.15
−29	8	5	0.00	164.02
28	−8	6	−78.58	162.30
29	−8	7	17.43	141.17
20	−11	7	186.34	152.13
−16	−2	7	−26.41	120.04
−24	3	9	100.63	140.78
−12	−4	10	16.77	108.03
−25	4	10	0.00	141.04
−18	18	−3	233.48	107.37
14	7	−2	324.34	129.02
−34	28	−2	−109.48	157.28
−31	21	−1	−120.57	145.27
−34	24	−1	69.73	184.36
28	3	1	−104.72	186.73
27	2	1	47.67	153.59
26	1	1	0.00	137.61
−23	9	1	164.55	123.74
25	−5	3	28.53	136.15
31	−3	4	45.03	161.51
13	−9	4	144.21	120.97
16	−10	5	119.25	108.69

24	−10	7	99.84	153.06
25	−10	8	101.95	136.68
21	−11	8	−5.28	132.99
11	−11	10	28.39	92.44
−10	−5	10	83.73	105.91
−14	−3	10	−24.17	114.76
−11	−4	11	−67.62	108.03
−13	−3	11	−60.35	116.21
9	19	−4	62.60	125.99
−19	24	−4	−28.00	103.27
17	17	−3	75.94	167.85
10	6	−2	298.85	119.91
−38	33	−2	43.05	151.08
−25	19	−2	−29.05	128.63
−23	14	−1	−21.00	131.27
−28	18	−1	0.00	133.65
−11	4	0	897.62	115.03
−13	5	0	681.70	120.84
−24	12	0	101.69	116.48
−29	16	0	132.33	139.85
−38	25	0	58.77	153.06
22	−4	2	0.00	125.59
−20	5	2	74.35	139.06
−27	10	2	89.54	134.57
29	−6	5	−6.34	157.15
−22	2	6	71.31	154.25
27	−9	9	25.49	146.06
17	−11	10	−17.96	118.33
10	20	−4	−203.64	149.36
13	13	−3	−14.39	117.01
−28	26	−3	0.00	151.61
22	15	−2	71.18	156.49
25	11	−1	−20.47	158.21
10	2	−1	1171.51	136.82
−15	6	0	201.26	110.54
−36	20	1	−129.02	165.87
−35	17	2	122.02	172.47
−38	20	2	44.11	156.23
−29	10	3	21.13	147.38
24	−7	4	181.58	130.48
−34	13	4	−24.83	158.34
13	−10	5	107.76	96.67
−24	4	5	64.45	148.57
−34	12	5	28.00	146.85
29	−7	6	51.11	147.51
−2	−8	6	37.37	56.65
−29	7	6	59.43	150.68
30	−7	7	14.53	148.17
−18	−1	7	−0.26	130.74
−27	5	7	144.34	146.59
−32	9	7	−218.30	152.27
−23	2	8	−211.03	141.57
−16	−2	10	9.38	112.65
−9	−5	11	−48.86	101.69

−15	−2	11	−145.93	121.23
−26	5	11	−75.27	152.53
−24	28	−4	−8.32	148.04
16	16	−3	13.07	160.06
14	14	−3	35.66	118.33
−20	15	−2	167.59	133.78
19	5	−1	80.29	132.85
−37	27	−1	0.00	149.36
20	−2	1	107.37	137.61
−33	17	1	209.45	168.51
−32	14	2	97.06	169.83
−37	17	3	−136.82	155.44
30	−5	5	−99.57	152.13
−6	−7	7	39.88	78.31
19	−11	9	40.28	129.82
14	−11	10	107.63	110.14
−8	−6	10	−37.37	95.61
19	−10	11	−9.77	126.51
15	15	−3	−56.39	129.42
8	11	−3	74.35	110.01
−20	19	−3	−53.35	111.72
−25	23	−3	85.44	121.63
−31	29	−3	−21.26	161.38
21	14	−2	−77.52	168.77
15	8	−2	59.56	115.69
−37	31	−2	−68.80	146.85
24	10	−1	21.92	169.96
7	2	−1	1526.49	148.57
−12	7	−1	611.44	101.55
19	1	0	36.58	142.36
11	−1	0	391.30	96.01
−9	3	0	319.32	86.37
18	−5	2	181.58	140.25
−4	−3	2	2043.12	179.21
26	−4	3	−19.02	134.70
−24	6	3	162.30	140.91
30	−6	6	0.00	147.25
24	−9	6	17.04	158.74
−34	11	6	22.05	148.44
17	−11	7	21.66	123.34
−30	7	8	−42.26	151.47
−13	−4	9	0.00	115.42
−26	4	9	29.19	151.21
25	−9	10	−18.88	142.10
−27	5	10	−1.85	151.34
23	−9	11	3.96	134.44
−17	−1	11	−0.13	125.19
23	−10	9	−107.63	143.15
20	−10	6	0.00	121.36
26	−9	8	−17.17	139.98
28	−8	9	141.97	151.74
24	−8	5	75.54	134.97
17	−7	3	424.05	144.21
−11	−5	9	−68.28	112.25

16	−3	1	25.09	128.10
−15	−3	9	54.54	122.82
−18	−1	10	−36.18	125.59
−20	0	7	146.06	134.83
14	3	−1	1331.57	178.68
20	6	−1	8.32	128.50
−17	7	0	725.68	139.85
−14	8	−1	133.12	104.20
23	9	−1	−179.74	161.64
−31	9	5	9.24	163.36
−31	10	4	−7.40	164.81
−30	14	1	40.67	133.91
−34	14	3	112.12	164.42
−30	23	−2	103.80	149.10
11	−11	7	93.24	89.01
21	−10	10	−31.69	136.55
25	−9	7	−6.21	153.32
21	−6	3	6.87	132.19
27	−3	3	0.00	151.47
23	−3	2	53.35	132.99
8	−1	0	706.13	101.29
−24	3	6	0.00	159.66
−10	6	−1	1363.93	141.57
−22	6	2	−66.29	129.95
21	7	−1	16.51	128.36
22	8	−1	160.32	141.83
−16	9	−1	359.87	118.85
−25	10	1	−101.29	131.80
20	13	−2	222.26	166.53
−26	13	0	259.10	141.44
4	17	−4	101.03	91.91
−34	20	0	−21.53	175.11
−21	25	−4	−27.47	110.93
−33	26	−2	−3.43	168.25
14	−11	7	111.33	101.42
9	−11	8	−16.64	94.03
20	−9	5	0.00	121.36
25	−6	4	7.26	133.38
−9	−6	9	33.28	102.22
28	−2	3	−128.23	170.49
−13	−2	4	109.21	117.40
21	−1	1	54.01	127.84
−15	−1	4	202.85	116.61
−19	0	11	0.00	129.68
−12	2	1	936.31	123.34
−14	3	1	1194.49	152.13
−25	3	8	0.00	142.10
−26	5	5	0.00	157.81
27	9	0	−42.26	172.34
16	9	−2	144.47	125.33
−29	11	2	3.96	132.99
−27	20	−2	10.56	131.40
−33	22	−1	0.00	179.34
−37	23	0	−61.80	161.91

−34	32	−3	−14.00	146.46
16	−11	9	−60.09	124.01
10	−11	9	72.63	94.16
16	−10	11	−67.48	122.02
−7	−6	11	36.71	94.29
−11	−3	4	336.49	129.95
−13	−3	5	122.29	105.25
29	−1	3	135.23	182.77
30	0	3	18.09	162.96
−20	0	10	103.93	132.06
20	2	0	139.72	130.48
−16	4	1	332.66	131.40
7	6	−2	159.00	80.95
−29	6	7	−10.43	143.55
−19	8	0	332.00	153.45
−31	8	6	−35.13	151.87
17	10	−2	−3.83	129.02
−31	11	3	−206.28	167.45
19	12	−2	65.50	141.97
−22	16	−2	−28.39	118.59
−31	17	0	−40.01	144.08
22	18	−2	−53.35	146.72
−30	19	−1	17.17	129.68
−36	25	−1	−134.44	164.55
−36	29	−2	13.73	148.83
22	−10	8	−51.90	138.93
21	−10	7	117.40	156.62
27	−8	8	−60.88	144.61
26	−8	10	0.00	148.44
24	−8	11	0.00	146.46
24	−2	2	144.08	157.42
−15	−2	5	33.54	114.63
−19	−1	9	−62.46	130.74
15	0	0	77.52	125.06
−17	0	4	429.20	124.67
−10	1	1	−3.04	77.65
−22	1	7	−136.55	148.04
−28	5	9	0.00	149.89
−26	7	3	50.98	140.64
26	8	0	45.16	173.66
−18	10	−1	63.65	134.17
18	11	−2	−24.04	123.34
25	14	−1	91.78	159.13
−36	14	4	−0.13	147.91
−37	18	2	188.72	167.19
−22	20	−3	47.01	120.57
−30	27	−3	−9.77	169.43
10	−10	11	32.75	98.52
−1	−9	8	36.05	53.48
25	−8	6	−89.41	170.23
25	−7	5	37.77	148.83
26	−5	4	34.73	141.17
−11	−4	5	−20.47	100.89
−17	−1	5	16.11	121.10

-21	1	11	-11.23	137.87
-7	2	0	60.22	89.80
-26	4	6	-32.35	158.34
-18	5	1	-37.77	139.72
-24	7	2	52.96	126.65
-34	15	2	-324.74	190.83
-35	18	1	62.86	176.17
-38	21	1	-110.40	160.32
-27	24	-3	-41.47	135.10
15	-11	8	96.93	136.68
13	-11	9	-81.61	114.23
13	-10	11	-44.24	115.95
26	-8	7	-118.19	155.96
14	-7	3	1422.83	173.79
-6	-7	10	-36.71	85.31
-9	-4	4	168.51	111.59
25	-1	2	82.27	141.57
-13	-1	3	137.74	118.19
22	0	1	3.70	139.59
-15	0	3	-108.42	145.00
-19	1	4	33.02	123.34
-22	1	10	-86.63	135.49
29	3	2	80.29	162.70
21	3	0	96.14	138.14
28	6	1	0.00	168.51
4	6	-2	1072.86	109.21
25	7	0	-1.06	186.21
11	7	-2	279.71	130.74
-21	9	0	245.77	152.66
-27	11	1	-12.41	148.44
9	12	-3	0.00	103.27
-36	15	3	0.00	155.96
21	17	-2	-70.39	152.79
-39	28	-1	0.00	146.85
-33	30	-3	-97.99	152.40
12	-11	8	31.03	122.16
17	-10	6	351.55	121.50
20	-9	11	-63.92	141.57
28	-7	8	-9.64	161.77
21	-7	4	58.90	129.16
22	-5	3	38.96	135.10
15	-5	2	218.03	123.87
27	-4	4	-17.56	163.49
19	-4	2	61.80	150.81
13	-3	1	229.39	100.63
-11	-2	3	68.41	111.20
26	0	2	192.94	151.61
-19	0	5	260.69	124.93
-17	1	3	77.52	136.55
28	2	2	110.54	191.88
22	4	0	9.90	156.76
-27	4	8	3.43	148.04
-8	5	-1	65.37	75.27
-28	6	5	-90.99	174.19

−33	10	5	−2.11	150.42
−20	11	−1	421.01	155.44
24	13	−1	70.92	159.66
−28	14	0	202.85	147.51
−32	15	1	0.00	159.13
5	18	−4	97.20	96.93
16	19	−3	−1.06	159.13
−14	20	−4	68.28	90.07
−32	24	−2	−2.38	172.60
−23	26	−4	181.85	130.61
−39	32	−2	−11.62	149.89
0	−8	5	62.99	45.96
26	−6	5	−70.65	179.60
−9	−5	5	23.51	88.35
4	−3	1	664.40	82.93
17	−2	1	293.57	145.27
−21	0	9	79.76	134.97
27	1	2	191.88	157.68
23	1	1	42.66	145.80
−24	2	7	−59.43	147.91
27	5	1	−40.54	183.43
−20	6	1	68.14	149.36
−31	12	2	0.00	158.34
−24	17	−2	0.00	140.64
−16	21	−4	38.56	101.55
−29	21	−2	51.24	132.19
−35	27	−2	−99.84	162.44
18	−10	10	0.00	130.48
22	−9	10	109.61	143.95
21	−9	6	−84.78	127.97
5	−9	5	84.92	64.05
27	−7	7	−60.35	153.32
26	−7	6	264.78	171.02
8	−7	3	156.36	71.84
−7	−7	9	37.64	93.10
−14	−4	8	190.43	125.72
28	−3	4	6.60	183.56
−16	−3	8	−30.11	126.25
29	−2	4	208.00	173.92
30	−1	4	0.00	166.93
24	2	1	37.64	153.06
−19	2	3	113.04	120.04
−21	2	4	0.00	127.97
−23	2	11	−1.06	143.68
25	3	1	75.27	139.98
26	4	1	118.72	167.45
−31	7	7	−106.44	151.21
−28	8	3	47.28	148.83
−33	9	6	21.26	153.98
5	11	−3	167.19	102.08
−33	12	3	88.35	199.94
−33	18	0	−146.85	167.19
−12	19	−4	184.49	77.92
−32	20	−1	0.00	156.36

−24	21	−3	−0.13	123.34
−35	23	−1	−84.39	182.38
17	−9	5	27.07	113.70
21	−8	5	2.64	126.38
5	−8	4	120.57	58.37
11	−7	3	1377.40	149.23
−12	−5	8	30.51	114.89
−15	−3	6	55.47	111.33
−8	0	1	136.55	77.52
−21	1	5	42.66	129.82
−24	2	10	0.00	139.19
11	3	−1	1687.48	175.51
−28	5	6	0.00	149.63
−30	6	9	−56.65	152.79
−26	8	2	136.15	139.59
−23	10	0	56.52	140.12
23	12	−1	−49.92	175.11
20	16	−2	175.11	160.32
15	18	−3	55.60	166.79
−18	22	−4	6.07	103.14
−39	24	0	−82.67	156.62
−38	26	−1	72.63	154.25
23	−9	8	84.92	143.15
−4	−8	7	61.80	67.75
18	−6	3	−76.33	157.81
27	−5	5	141.17	179.34
23	−4	3	61.14	150.68
−13	−4	6	−15.98	108.82
−17	−2	6	−66.69	119.12
16	1	0	0.13	151.21
−23	1	9	−274.95	143.02
−22	12	−1	105.52	139.46
−29	12	1	69.99	137.61
10	13	−3	−3.70	108.29
1	17	−4	25.49	73.43
6	19	−4	−9.38	101.55
21	20	−2	−2.25	146.46
8	21	−4	0.00	124.01
−29	25	−3	−110.67	159.93
−32	28	−3	−115.03	168.25
−38	30	−2	−116.35	153.85
22	−9	7	48.47	162.17
28	−6	7	−73.95	152.27
27	−6	6	−69.33	173.66
−9	−3	3	511.21	105.91
−21	3	3	277.20	128.36
−26	3	7	23.77	146.19
−29	5	8	28.92	160.85
−22	7	1	−48.33	141.17
−30	7	5	112.65	152.79
12	8	−2	127.70	136.02
26	11	0	−54.28	161.64
22	11	−1	−134.31	177.49
−30	15	0	−96.67	138.66

−36	16	2	−113.84	174.85
14	17	−3	−15.05	146.46
−37	19	1	−44.24	167.72
7	20	−4	−45.03	115.82
−25	27	−4	90.20	153.45
19	−10	8	56.13	140.38
18	−10	7	−2.77	130.08
14	−10	6	320.12	109.87
8	−10	6	−43.71	73.16
22	−6	4	72.77	143.81
−10	−6	8	81.35	109.74
29	−5	7	−184.23	155.70
28	−5	6	−146.19	172.08
−7	−5	4	250.92	100.89
−11	−5	6	122.16	107.10
20	−3	2	14.26	135.63
10	−3	1	938.43	116.74
−19	−1	6	7.53	129.55
−20	−1	8	−168.11	137.21
12	0	0	792.10	122.16
−23	3	4	213.67	131.14
−25	3	11	−200.73	155.70
−15	11	−2	52.16	108.29
−35	11	5	0.00	161.77
−17	12	−2	36.98	131.80
11	14	−3	58.24	116.21
19	15	−2	−121.23	176.17
−15	15	−3	16.51	118.19
24	16	−1	−116.21	167.32
13	16	−3	86.24	127.44
−17	16	−3	−33.28	117.40
−34	16	1	−22.85	186.21
−26	18	−2	−28.53	138.80
12	−5	2	299.78	98.78
29	−4	6	81.35	153.98
24	−3	3	76.99	155.83
−23	2	5	103.54	143.02
17	6	−1	0.00	118.85
−30	9	3	191.09	168.11
21	10	−1	107.76	150.29
−25	11	0	84.92	132.72
−33	13	2	71.45	179.47
−13	14	−3	16.11	108.03
12	15	−3	35.13	115.03
−26	22	−3	−16.51	125.46
−31	22	−2	139.46	171.55
−20	23	−4	29.05	109.21
−34	25	−2	−56.52	173.00
−35	31	−3	26.02	151.08
6	−10	10	0.00	76.46
23	−8	10	−19.81	151.21
21	−8	11	40.67	143.15
18	−1	1	189.24	152.00
−21	0	6	−61.28	157.42

−22	0	8	31.17	143.15
−15	1	2	158.61	129.68
−17	2	2	−15.05	150.95
−25	2	9	−5.81	150.15
−26	3	10	0.00	151.61
−23	4	3	222.65	146.46
−30	6	6	67.22	147.38
18	7	−1	−7.26	139.06
−24	8	1	0.00	140.12
−33	8	7	−72.37	152.66
20	9	−1	115.29	138.40
−28	9	2	46.35	134.17
25	10	0	56.13	182.90
−19	13	−2	98.78	141.97
−24	13	−1	167.19	131.27
−35	13	3	−121.76	164.55
18	14	−2	0.00	160.06
−19	17	−3	97.33	108.95
−10	18	−4	52.96	68.28
20	19	−2	−44.90	148.57
−37	28	−2	33.15	153.19
15	−10	10	31.30	121.76
11	−10	6	76.33	94.82
17	−9	11	0.00	128.23
24	−8	8	27.47	143.42
22	−8	6	0.00	138.40
22	−7	5	−70.92	129.68
−5	−7	11	96.40	79.90
−7	−6	5	125.99	91.12
25	−2	3	40.28	145.66
−13	0	2	454.69	120.84
29	2	3	−33.15	173.66
17	2	0	158.34	155.44
−25	4	4	46.09	143.95
19	8	−1	−104.72	134.17
13	9	−2	130.21	122.82
−35	19	0	242.07	181.98
−34	21	−1	−133.51	183.83
−37	24	−1	−90.59	174.85
14	−9	5	−54.94	104.06
7	−3	1	2468.22	221.99
21	−2	2	112.12	144.34
26	−1	3	23.64	156.10
27	0	3	30.90	182.90
28	1	3	0.13	177.09
−19	3	2	254.22	152.79
−25	3	5	−50.45	175.77
−28	4	7	55.07	146.85
8	7	−2	212.22	100.50
17	13	−2	−24.83	133.78
−31	13	1	49.52	149.89
23	15	−1	97.99	164.42
−22	24	−4	−59.16	119.38
23	−8	7	−56.52	168.91

-9	-6	6	249.60	100.23
23	-5	4	116.35	142.10
19	-5	3	188.19	141.83
6	-5	2	1255.24	121.36
16	-4	2	233.48	135.49
-23	1	6	90.07	163.49
28	5	2	-9.24	178.94
-32	8	5	-60.62	165.47
24	9	0	-18.75	192.41
14	10	-2	126.78	122.16
2	11	-3	0.00	76.73
-11	13	-3	0.40	95.35
-21	14	-2	198.75	133.51
-32	16	0	0.00	161.77
-38	17	2	126.51	169.83
-21	18	-3	297.53	123.74
-31	18	-1	95.48	152.93
-28	19	-2	-56.39	131.66
-31	26	-3	-22.45	171.94
-27	28	-4	-23.37	163.62
-34	29	-3	18.62	155.96
19	-9	10	0.00	141.17
18	-9	6	0.00	123.08
24	-7	10	-62.99	155.30
9	-5	2	1197.27	124.93
14	-2	1	795.54	136.82
-11	-1	2	248.67	102.88
19	0	1	126.91	151.08
-24	1	8	0.00	142.23
-27	3	9	116.87	157.02
12	4	-1	924.69	145.00
-21	4	2	295.68	139.32
-28	4	10	106.18	166.13
-25	5	3	16.64	142.76
-26	9	1	279.04	152.53
15	11	-2	15.58	128.63
16	12	-2	-105.91	130.87
-27	12	0	-103.27	149.49
-26	14	-1	103.54	132.99
-36	17	1	-102.35	177.62
15	-10	7	15.19	111.86
25	-7	8	63.39	147.78
22	-7	11	-26.94	154.12
-8	-7	8	-59.69	100.63
15	-6	3	11.23	131.40
-7	-4	3	1713.89	169.43
22	-1	2	6.34	145.53
18	3	0	175.24	140.64
8	3	-1	278.52	86.24
27	4	2	-52.03	197.83
-27	5	4	4.36	172.74
26	7	1	298.19	200.07
23	8	0	130.21	161.77
-30	10	2	7.66	150.42

−32	10	3	−74.48	178.68
6	12	−3	53.22	111.06
22	14	−1	8.58	166.79
−35	14	2	−38.69	177.36
19	18	−2	151.47	158.34
−28	23	−3	−40.41	143.95
16	−10	8	143.02	150.02
12	−10	10	64.84	114.63
20	−9	8	27.07	155.17
18	−8	5	62.60	125.33
23	−7	6	0.00	159.66
23	−6	5	−65.77	139.98
24	−4	4	101.95	141.44
−5	1	0	791.05	98.65
−25	2	6	0.00	156.76
−6	4	−1	773.75	106.44
−27	4	5	21.13	173.13
−23	5	2	−76.07	135.23
−32	7	6	143.81	157.55
−37	14	3	23.24	161.51
−23	15	−2	−3.30	125.46
−23	19	−3	32.49	124.14
14	20	−3	−37.77	167.72
−33	23	−2	0.00	178.02
−24	25	−4	178.68	134.97
−36	26	−2	81.88	174.98
−37	32	−3	−50.32	158.87
9	−10	10	−53.75	101.42
19	−9	7	95.22	149.49
11	−9	5	−37.64	106.84
8	−8	4	−35.26	88.08
24	−7	7	47.94	160.45
26	−6	8	26.54	151.08
25	−6	10	7.92	157.42
−15	−4	7	102.61	127.57
−17	−3	7	175.91	134.04
−19	−2	7	−19.94	123.87
−6	−1	1	222.13	76.99
23	0	2	94.56	140.91
29	1	4	64.97	166.13
24	1	2	0.00	139.19
20	1	1	66.95	143.95
25	2	2	−70.92	144.08
26	3	2	169.70	164.02
19	4	0	178.55	129.55
20	5	0	147.91	143.95
−30	5	7	0.00	156.49
25	6	1	−8.32	178.15
25	13	0	0.13	163.23
−33	14	1	11.09	183.17
−37	20	0	52.03	173.26
−36	22	−1	−68.01	173.79
14	−9	11	−4.09	124.67
−5	−8	9	92.57	85.18

25	−3	4	−75.01	145.93
28	0	4	8.85	180.26
9	0	0	1235.96	137.08
13	1	0	80.29	110.93
24	5	1	−12.94	153.45
−16	5	0	421.67	127.84
−27	6	3	59.56	139.72
−34	9	5	−114.76	156.76
−28	10	1	128.23	138.66
−29	13	0	99.18	140.78
−28	15	−1	0.00	122.29
−2	17	−4	54.28	51.77
2	18	−4	93.24	87.95
−30	20	−2	−20.47	143.81
−39	25	−1	225.96	173.79
−29	29	−4	−61.54	156.62
−39	29	−2	0.00	152.79
8	−9	5	108.42	92.18
23	−6	11	52.69	162.04
27	−5	8	55.73	155.57
24	−5	5	23.24	153.72
−13	−5	7	−72.37	120.97
20	−4	3	332.40	140.51
26	−2	4	−2.25	165.21
27	−1	4	0.00	208.39
−21	−1	7	105.52	139.46
21	2	1	64.05	136.68
22	3	1	0.00	144.87
23	4	1	8.85	136.82
−29	4	9	−49.79	161.77
−18	6	0	195.19	145.93
−1	11	−3	360.39	53.48
21	13	−1	40.81	176.70
18	17	−2	−24.56	173.53
−34	17	0	−232.03	189.51
13	19	−3	50.18	165.21
−33	19	−1	0.00	181.98
19	21	−2	0.00	152.53
−33	27	−3	35.92	163.76
18	−8	11	−69.46	141.04
−4	−8	10	23.11	70.92
25	−6	7	83.59	159.66
24	−6	6	−20.34	175.38
17	−3	2	519.00	150.55
−27	3	6	−104.06	155.17
−14	4	0	1149.59	146.46
−29	5	5	−4.23	176.96
−25	6	2	−33.94	142.63
−29	6	4	−0.53	191.62
−20	7	0	151.08	154.51
−17	8	−1	10.04	126.12
−32	11	2	2.38	177.23
−34	11	3	0.26	188.45
−25	20	−3	100.63	130.74

−30	24	−3	−62.99	171.15
−36	30	−3	−52.69	158.34
20	−8	10	25.88	147.91
13	−8	12	0.00	132.59
19	−6	4	169.83	126.51
−17	−3	10	−2.91	135.63
−9	−2	2	1199.38	136.29
−19	−2	10	76.60	142.10
−23	0	7	−125.46	152.66
−32	6	7	53.88	158.08
−15	7	−1	6.07	106.31
9	8	−2	124.40	118.46
−34	8	6	2.38	163.36
−19	9	−1	352.34	149.89
24	12	0	59.43	186.21
7	13	−3	176.17	104.33
−37	15	2	−45.83	171.15
22	17	−1	0.00	158.74
−38	18	1	−121.10	167.98
−26	26	−4	57.58	158.74
12	−10	7	24.17	96.40
23	−7	9	151.08	156.49
17	−7	12	2.38	154.12
−5	−6	4	341.91	93.37
26	−5	7	−111.33	157.42
25	−5	6	−15.05	183.83
−15	−4	10	0.00	124.67
−16	−3	11	−66.16	134.97
−18	−2	11	−56.52	139.19
−21	−1	10	45.03	137.87
−29	7	3	0.00	176.43
−22	8	0	287.36	151.47
−21	10	−1	120.84	151.08
20	12	−1	−16.11	161.51
17	16	−2	89.67	174.58
12	18	−3	−24.56	138.40
−35	24	−2	−163.36	184.36
−38	27	−2	19.02	168.64
6	−10	7	83.46	68.14
4	−10	8	87.56	56.65
21	−8	8	42.66	154.12
19	−8	6	48.47	127.84
−7	−7	6	425.50	99.05
−11	−6	7	325.79	113.04
27	−4	7	−122.82	158.21
28	−3	7	34.60	162.96
21	−3	3	60.88	148.17
28	−1	5	−126.38	170.36
15	−1	1	151.61	122.95
−20	−1	11	−25.75	143.15
−17	3	1	627.55	156.62
28	4	3	−115.16	185.55
−27	7	2	112.78	137.08
26	10	1	−0.40	194.00

−30	11	1	106.97	154.64
−9	12	−3	229.65	81.75
−31	14	0	−48.47	153.06
−35	15	1	0.00	193.07
−27	17	−2	47.01	133.12
3	19	−4	91.39	94.82
−17	20	−4	104.33	105.25
−32	21	−2	−74.88	181.45
13	−10	8	81.35	132.85
5	−10	9	94.82	74.88
16	−9	10	−82.27	133.51
15	−9	6	163.36	119.78
11	−9	11	−80.43	119.52
19	−7	5	152.00	132.99
−13	−5	10	−20.87	120.97
26	−4	6	−76.86	165.08
13	−4	2	86.63	111.33
−14	−4	11	41.73	129.16
27	−3	6	97.06	162.70
−18	−3	9	53.62	131.93
28	−2	6	3.04	160.59
−18	−1	4	176.04	130.21
−22	0	11	10.43	153.85
−25	1	7	96.27	152.53
−12	3	0	452.18	102.35
−19	4	1	72.11	143.42
−29	4	6	−89.67	158.87
−13	6	−1	−8.85	96.40
−31	7	4	0.00	181.45
−24	9	0	29.58	140.25
19	11	−1	−56.13	140.12
−23	11	−1	214.07	133.12
11	17	−3	77.12	123.34
18	20	−2	−19.41	159.53
−19	21	−4	155.04	110.67
−27	21	−3	148.70	130.74
−39	21	0	0.00	152.00
−38	23	−1	−35.92	161.91
5	−9	11	−2.38	83.46
20	−8	7	108.69	167.85
24	−6	9	−62.99	161.91
12	−6	3	332.27	106.18
−16	−4	9	0.00	129.16
22	−2	3	213.15	146.06
−16	−2	4	−52.56	116.48
−20	−2	9	−85.97	137.48
−20	0	4	−81.61	144.08
−23	0	10	−160.98	153.45
14	2	0	31.56	128.76
−15	2	1	298.46	128.50
27	3	3	0.00	202.45
5	3	−1	91.52	69.86
−21	5	1	−5.28	140.91
−31	6	5	−118.06	168.51

27	7	2	90.99	178.41
5	7	−2	273.50	80.29
23	11	0	161.11	193.73
−34	12	2	19.68	188.32
−36	12	3	−180.92	168.11
8	14	−3	−80.16	107.10
16	15	−2	−56.52	148.96
21	16	−1	−137.21	176.17
−36	18	0	55.86	183.83
−15	19	−4	53.22	96.80
4	20	−4	58.50	101.69
−35	20	−1	71.05	179.74
5	21	−4	53.62	115.03
−21	22	−4	69.33	119.65
−32	25	−3	43.98	180.13
−28	27	−4	−172.74	174.06
−35	28	−3	0.00	160.98
9	−10	7	−1.19	86.90
15	−8	5	17.96	110.93
−5	−7	5	127.97	82.27
20	−5	4	389.98	143.02
16	−5	3	−34.20	146.98
−2	−4	2	99.18	47.41
18	−2	2	166.40	169.70
11	−2	1	604.18	103.40
−18	−2	5	65.90	128.63
−20	−1	5	−58.24	133.91
−22	−1	9	0.00	142.49
−22	1	4	145.14	146.19
26	2	3	−5.28	180.40
−30	4	8	0.00	171.15
15	7	−1	18.22	129.16
−31	8	3	−12.94	180.92
18	10	−1	85.71	135.10
9	15	−3	25.09	117.53
10	16	−3	183.96	119.91
17	−9	8	3.17	154.25
10	−8	12	−77.26	127.04
21	−7	10	0.00	153.85
19	−7	11	118.33	154.38
−14	−5	9	1.19	125.46
−14	−3	4	312.59	119.91
−16	−3	5	88.35	118.06
23	−1	3	80.56	153.85
6	0	0	2764.43	249.60
−18	0	3	235.60	127.57
25	1	3	0.00	154.25
−20	1	3	62.07	123.34
−25	1	10	−153.06	159.93
−27	2	7	0.00	157.28
16	8	−1	−35.39	129.42
−29	8	2	−94.69	167.06
17	9	−1	0.00	137.21
10	9	−2	319.06	138.00

−26	10	0	31.17	142.36
−25	12	−1	0.00	144.74
−32	12	1	−93.90	171.42
24	15	0	−33.02	165.47
−23	23	−4	125.85	123.08
−38	31	−3	−88.08	169.17
16	−9	7	33.81	123.87
8	−9	11	39.75	106.84
−6	−8	8	39.49	91.12
22	−7	8	111.33	157.02
25	−5	9	−12.94	164.02
−16	−1	3	19.94	134.44
24	0	3	68.41	141.97
−22	0	5	−72.63	148.17
−24	0	9	37.77	145.80
−13	1	1	273.37	107.76
−24	2	4	21.79	140.64
9	4	−1	289.61	95.08
−31	5	6	1.58	157.55
26	6	2	−2.51	196.37
22	10	0	75.54	170.09
15	14	−2	73.82	132.72
−33	15	0	130.48	187.39
−37	16	1	73.95	168.77
−29	18	−2	13.60	144.21
−37	25	−2	12.15	163.23
20	−7	6	36.32	130.74
18	−6	12	1.06	164.02
−12	−5	11	111.72	117.27
−14	−4	5	219.09	117.80
16	0	1	434.61	150.68
−22	2	3	97.46	144.87
−25	7	1	148.17	151.34
−33	7	5	−44.37	162.57
24	8	1	98.91	184.62
−33	8	4	103.01	180.66
−18	15	−3	118.06	127.97
−20	16	−3	125.33	114.63
21	19	−1	0.00	159.79
17	19	−2	−212.49	173.92
−29	22	−3	−34.86	153.85
−34	22	−2	−32.09	196.51
10	−10	8	279.18	119.91
19	−8	9	8.19	143.81
15	−8	11	26.54	142.10
7	−8	12	−59.56	115.16
21	−7	7	145.40	175.91
20	−6	5	91.39	133.25
−11	−6	10	−57.58	114.89
21	−4	4	57.84	137.61
−14	−2	3	36.45	138.27
19	−1	2	103.93	136.68
−24	1	5	23.37	166.13
−27	2	10	0.00	161.38

28	3	4	−0.13	164.94
15	3	0	220.67	151.74
−24	3	3	−85.31	139.46
25	5	2	−149.89	185.28
−11	5	−1	64.97	77.78
−32	5	8	31.17	140.64
21	9	0	−31.17	154.51
11	10	−2	209.18	130.34
−20	12	−2	0.00	147.25
14	13	−2	−49.52	132.06
−27	13	−1	−6.74	144.74
−36	13	2	11.23	176.96
−16	14	−3	53.88	131.40
20	15	−1	0.00	208.39
−8	17	−4	58.90	57.05
−22	17	−3	25.75	124.14
−13	18	−4	31.17	86.10
−37	21	−1	0.00	173.00
−25	24	−4	104.46	142.23
−30	28	−4	0.00	163.36
−9	−7	7	152.53	98.12
23	−6	8	200.73	158.21
22	−6	10	−126.78	162.83
−12	−6	9	45.16	115.03
−12	−4	4	129.16	120.18
−26	1	9	−116.48	158.47
−10	2	0	12.81	81.48
−26	3	4	38.83	168.64
−29	3	7	−74.22	161.51
−31	9	2	0.00	180.92
−33	9	3	−90.07	181.19
12	11	−2	237.18	120.70
−28	11	0	71.71	140.51
13	12	−2	−0.79	125.06
3	12	−3	158.08	94.95
−22	13	−2	−6.07	129.42
23	14	0	0.00	185.02
−38	19	0	334.64	177.23
12	21	−3	−204.96	176.17
−34	26	−3	1.06	175.11
−37	29	−3	−21.79	159.53
20	−6	11	−45.83	163.36
−5	−5	3	678.27	94.29
−18	−3	6	73.69	132.85
−20	−2	6	1.72	142.63
20	0	2	128.36	141.31
17	1	1	293.70	156.76
10	1	0	339.66	90.99
27	2	4	0.00	193.21
−26	2	5	98.65	178.41
24	4	2	−101.03	158.74
23	7	1	32.35	158.08
−27	8	1	−144.87	151.87
−34	13	1	0.00	184.62

−24	18	−3	10.17	132.46
−31	19	−2	−31.03	158.74
17	22	−2	143.68	162.70
7	−10	8	38.56	90.99
13	−9	10	−4.09	122.55
12	−9	6	181.32	109.21
17	−8	10	−13.07	144.08
14	−7	12	−49.65	147.25
24	−5	8	−80.16	160.06
23	−5	10	52.43	166.40
17	−4	3	28.00	150.42
22	−3	4	143.95	140.51
−7	−3	2	4062.20	345.47
−22	−1	6	21.13	169.57
−11	0	1	1789.69	177.89
21	1	2	−25.36	151.34
22	2	2	189.90	150.15
23	3	2	324.87	155.44
16	4	0	0.00	153.06
−26	4	3	105.65	144.21
−33	6	6	−14.66	167.72
25	12	1	28.92	178.02
19	14	−1	165.21	195.98
−24	14	−2	0.00	133.25
−35	16	0	−156.10	198.75
16	18	−2	−170.09	187.13
−31	23	−3	−127.57	179.34
22	−6	7	−78.97	170.09
21	−6	6	34.86	146.72
16	−6	4	4.36	130.21
21	−5	5	0.00	135.36
19	−5	12	−168.11	177.75
−12	−5	5	173.66	113.84
−15	−5	8	−140.91	124.27
−16	−4	6	25.62	120.18
14	−3	2	577.37	125.99
23	−2	4	0.00	147.64
25	0	4	−14.00	174.85
−24	0	6	−4.62	168.11
26	1	4	−10.30	190.56
−18	1	2	112.78	158.47
−20	2	2	−11.09	124.93
−28	2	9	−141.70	172.21
−29	3	10	113.70	180.79
−28	4	4	189.51	191.62
17	5	0	32.09	149.36
22	6	1	52.69	140.38
18	6	0	45.69	135.63
−35	8	5	79.50	176.83
−35	9	4	−155.70	166.79
−30	12	0	107.76	150.55
−14	13	−3	−10.96	118.19
−29	14	−1	120.31	141.17
−36	23	−2	0.00	181.45

-27	25	-4	22.85	167.85
-39	26	-2	-95.22	168.51
16	-8	6	59.03	119.78
25	-4	8	20.21	158.34
26	-3	8	0.00	163.62
-12	-3	3	-99.18	129.55
24	-1	4	13.07	151.74
-25	0	8	172.21	165.21
18	2	1	87.42	156.62
2	3	-1	1341.74	130.21
-22	3	2	0.13	136.15
-28	3	5	-13.34	171.15
-31	4	7	-53.09	173.40
21	5	1	51.50	142.76
27	6	3	-12.02	185.81
-29	9	1	44.24	151.87
-33	10	2	-67.48	188.05
-35	10	3	6.74	174.72
22	13	0	-92.57	194.92
-38	14	2	2.11	159.93
-26	15	-2	46.49	144.21
20	18	-1	56.65	182.77
-26	19	-3	170.75	133.78
11	20	-3	74.75	162.04
-32	29	-4	-1.32	160.72
18	-8	8	72.63	165.34
20	-7	9	-11.09	149.23
9	-6	3	160.06	80.95
21	-5	11	0.13	169.57
10	-4	2	948.20	120.18
-16	0	2	342.04	150.95
19	3	1	176.43	145.66
20	4	1	-62.20	158.21
-24	4	2	-31.17	153.19
-28	5	3	-54.94	174.98
26	9	2	0.00	193.60
-39	22	-1	175.51	169.70
16	-7	5	127.04	120.18
23	-5	7	-151.61	173.66
22	-5	6	-78.05	160.98
-14	-5	6	57.45	114.76
12	-1	1	38.03	97.20
3	0	0	9305.15	757.50
27	1	5	28.26	180.53
-26	1	6	-31.30	164.68
10	5	-1	193.73	109.87
-30	5	4	0.00	191.88
24	11	1	-72.24	196.11
18	13	-1	-46.35	147.51
-36	14	1	-124.27	187.53
-33	20	-2	0.00	189.90
20	21	-1	-104.33	164.68
-36	27	-3	-66.95	178.68
17	-8	7	3.17	132.19

12	-8	5	168.64	115.82
-10	-6	11	-69.99	113.31
24	-4	7	68.80	167.72
18	-3	3	-82.14	139.06
26	0	5	11.75	204.96
-27	1	8	9.90	165.60
-30	3	9	87.95	179.60
-30	4	5	140.38	165.34
26	5	3	92.31	190.43
-26	5	2	56.79	147.91
6	8	-2	87.82	91.78
-32	13	0	0.26	173.53
-31	15	-1	-33.15	149.63
-28	16	-2	72.77	142.36
15	17	-2	113.31	159.27
-37	17	0	-173.92	183.30
-36	19	-1	139.59	178.55
-28	20	-3	0.00	140.64
16	21	-2	85.31	169.17
-33	24	-3	-49.79	182.51
-29	26	-4	194.39	183.96
-39	30	-3	-70.26	172.08
14	-9	8	-159.66	155.70
13	-9	7	31.83	107.63
16	-7	11	-83.20	152.53
21	-6	9	74.35	157.15
-13	-6	8	-119.38	126.12
13	-5	3	238.77	120.97
-10	-5	4	-83.46	120.84
23	-4	6	18.36	178.55
25	-3	7	13.34	167.85
26	-2	7	27.07	163.76
8	-2	1	772.16	105.25
25	-1	5	-256.59	197.56
-14	-1	2	224.37	133.25
27	0	6	-23.11	167.19
-28	2	6	0.00	160.19
-33	5	7	0.00	166.00
-30	6	3	-135.49	194.00
25	8	2	-47.28	198.88
-31	10	1	3.96	171.68
21	12	0	71.18	189.24
12	-8	11	-104.59	120.97
18	-7	10	-77.78	160.85
-10	-7	9	22.45	111.33
24	-3	6	84.12	190.17
25	-2	6	-35.52	173.66
26	-1	6	13.87	189.24
-29	2	8	52.43	172.74
25	4	3	-75.01	199.02
-19	5	0	186.34	154.12
-21	6	0	91.52	161.25
-23	7	0	85.71	133.91
-35	11	2	86.24	182.64

−37	11	3	69.86	171.15
17	12	−1	26.02	143.02
19	17	−1	47.67	187.66
−1	18	−4	109.08	71.71
−20	20	−4	28.13	114.10
−22	21	−4	112.12	122.16
−38	24	−2	−118.72	176.04
−9	−7	10	−21.26	101.03
15	−6	12	12.55	160.59
−10	−6	5	−83.33	96.67
17	−5	4	42.52	129.82
19	−2	3	−8.85	132.72
15	−2	2	287.89	140.78
−9	4	−1	695.70	100.76
−28	6	2	−8.32	149.76
−32	6	4	87.95	174.58
−20	8	−1	274.16	160.45
−22	9	−1	−111.86	141.44
−12	12	−3	117.53	105.65
4	13	−3	−38.56	112.52
22	16	0	−207.86	183.83
−11	17	−4	162.30	78.18
−18	19	−4	−78.97	112.12
−35	21	−2	30.51	185.68
−24	22	−4	11.23	124.40
10	−9	10	44.11	118.06
−5	−8	6	99.05	80.03
19	−7	8	98.25	158.21
−12	−6	6	−24.30	110.54
22	−5	9	19.68	165.21
−20	−3	7	0.00	145.66
−4	−2	1	1035.49	117.01
−22	−2	7	−76.07	152.40
11	2	0	142.23	99.57
24	3	3	30.51	167.98
−30	3	6	−45.96	165.87
−17	4	0	185.41	137.48
27	5	4	−13.73	157.68
−32	5	5	0.00	166.79
2	7	−2	734.39	81.61
−18	7	−1	229.65	147.51
−25	8	0	104.99	151.08
−34	14	0	190.04	197.04
−38	15	1	0.00	169.17
14	16	−2	294.23	148.44
−30	17	−2	−57.58	149.10
9	18	−3	−40.81	124.14
−30	21	−3	69.99	168.51
−31	27	−4	−75.94	168.25
−38	28	−3	−117.80	175.38
9	−9	6	8.98	89.41
−2	−9	7	106.71	22.58
16	−8	9	0.00	148.17
17	−7	6	151.74	131.40

11	−7	12	−53.88	140.25
−10	−4	3	330.55	113.04
−18	−4	7	32.35	141.97
20	−1	3	5.81	132.59
−24	−1	7	0.00	156.10
−8	1	0	686.06	106.44
23	2	3	113.04	151.61
24	7	2	−14.92	188.85
−32	7	3	−59.56	192.15
20	11	0	−16.38	150.55
16	11	−1	−48.47	138.00
−33	11	1	−52.96	196.90
24	14	1	−117.80	163.10
19	20	−1	96.80	168.91
−38	20	−1	−50.58	188.45
−35	25	−3	−47.67	167.72
18	−7	7	13.73	147.91
19	−6	10	10.83	164.68
17	−6	5	84.92	128.89
23	−4	9	25.88	170.09
24	−3	9	−66.56	172.21
−9	−1	1	21.79	82.41
21	0	3	58.90	147.51
−26	0	7	129.16	161.38
22	1	3	34.47	144.08
−31	3	8	0.26	177.62
−22	4	1	54.94	136.42
−16	6	−1	61.41	114.89
−30	7	2	131.40	168.91
−27	9	0	286.31	159.27
−26	11	−1	75.54	146.98
−39	18	0	0.00	174.32
15	20	−2	88.08	183.30
−26	23	−4	−81.09	148.44
14	−8	10	−33.15	137.74
17	−6	11	33.15	161.38
−16	−5	7	−76.86	139.19
−12	−2	2	171.15	121.63
13	0	1	230.84	116.87
−15	3	0	445.97	129.16
−20	3	1	58.64	153.45
26	4	4	−82.93	201.66
−24	5	1	−84.65	147.38
23	6	2	−21.92	159.40
−34	7	4	139.98	188.05
22	9	1	0.00	163.89
15	10	−1	21.39	145.00
−37	12	2	24.43	173.66
5	14	−3	260.95	107.10
13	15	−2	0.00	128.50
18	16	−1	−67.88	199.02
8	17	−3	3.83	120.44
−16	18	−4	86.10	104.33
−7	−8	7	63.13	86.76

−11	−7	8	59.03	121.63
20	−6	8	50.84	169.43
6	−6	3	142.49	62.07
18	−4	4	111.72	133.12
16	−1	2	191.49	150.95
−28	1	7	255.80	173.79
−18	2	1	25.75	156.10
6	4	−1	1886.62	174.58
−32	4	6	52.56	169.96
−34	6	5	20.07	170.75
26	8	3	27.73	194.00
13	8	−1	52.03	151.74
−34	8	3	−46.22	184.75
14	9	−1	75.27	127.97
7	9	−2	410.58	115.82
19	10	0	20.73	154.64
−29	10	0	−139.98	151.21
25	11	2	−4.62	185.81
−4	11	−3	919.94	81.88
−7	11	−3	1529.40	142.76
−28	12	−1	0.00	157.81
21	15	0	45.43	189.90
−21	15	−3	83.46	120.97
−23	16	−3	58.90	131.66
−32	18	−2	−108.16	173.92
3	22	−4	0.00	114.10
−32	22	−3	60.22	186.87
−37	22	−2	196.11	180.40
−28	24	−4	−45.43	175.11
−40	25	−2	187.92	161.91
−33	28	−4	−117.01	168.91
16	−5	12	−160.19	176.57
−20	−3	10	−159.40	155.70
−22	−2	10	52.16	162.96
−21	−1	4	−70.26	133.12
−24	−1	10	21.79	162.30
−23	0	4	173.92	144.87
−26	6	1	20.21	148.96
−32	8	2	256.86	220.41
−35	12	1	2.11	202.19
−19	14	−3	104.06	123.61
6	15	−3	221.47	104.72
−36	15	0	−153.32	198.88
7	16	−3	78.18	118.85
−25	17	−3	160.98	143.55
0	19	−4	103.40	80.69
10	22	−3	−278.91	182.11
15	23	−2	47.01	166.93
13	−8	6	−58.90	115.16
20	−5	10	78.44	168.64
−19	−2	4	163.49	136.42
−25	1	4	−17.30	170.09
25	3	4	16.51	200.60
12	3	0	678.00	132.06

22	5	2	−3.57	154.64
21	8	1	8.32	145.80
23	13	1	−12.55	185.94
12	14	−2	152.66	132.99
2	21	−4	89.67	97.99
−37	26	−3	0.00	169.83
−40	29	−3	7.40	181.06
15	−8	8	−155.17	168.91
17	−7	9	83.73	157.02
19	−6	7	−18.62	168.64
18	−6	6	−49.92	131.53
21	−5	8	39.22	167.72
18	−5	5	0.00	136.02
14	−4	3	686.72	147.64
−18	−4	10	127.70	144.34
19	−3	4	252.24	137.34
−21	−3	9	432.90	154.78
−21	−2	5	51.64	149.23
−23	−2	9	−62.33	161.77
−23	−1	5	152.13	174.58
17	0	2	519.53	169.17
−25	0	5	70.39	180.53
−26	0	10	−134.04	173.53
−16	1	1	268.74	150.29
−27	2	4	55.33	185.28
−30	2	7	−223.71	181.19
−14	5	−1	−7.79	99.71
−34	5	6	109.08	185.02
−28	7	1	57.31	147.64
−36	8	4	0.00	170.89
−31	11	0	−57.58	158.34
−25	13	−2	221.47	146.59
−30	13	−1	63.52	149.23
17	15	−1	−23.11	161.77
21	18	0	−47.41	185.28
−27	18	−3	−21.53	141.57
18	19	−1	218.03	180.53
14	19	−2	149.49	186.87
1	20	−4	73.69	92.31
11	−9	8	72.90	134.04
7	−9	10	−29.19	98.25
1	−9	10	17.56	29.71
9	−8	5	263.20	114.89
9	−8	11	88.74	129.02
−14	−6	7	−58.77	132.19
18	−5	11	119.91	172.47
22	−4	8	−34.07	166.26
21	−4	10	314.70	189.38
−19	−4	9	0.00	146.98
11	−3	2	443.33	105.65
−17	−3	4	312.32	133.12
−19	−3	5	102.88	129.82
25	−1	8	85.05	173.13
−25	−1	9	−5.68	162.96

14	1	1	292.38	140.25
7	1	0	715.24	102.08
24	2	4	−41.20	182.24
26	3	5	68.94	203.37
−4	3	−1	679.45	84.52
21	4	2	0.00	145.66
25	7	3	−54.01	206.15
20	7	1	−101.42	148.17
8	10	−2	118.33	126.25
11	13	−2	−72.63	125.59
−17	13	−3	85.18	141.17
20	14	0	185.02	204.83
−34	19	−2	58.24	191.75
−34	23	−3	−31.17	192.94
−30	25	−4	−23.64	187.39
10	−9	7	0.00	99.05
14	−8	7	124.80	121.89
13	−7	5	185.02	113.57
13	−7	11	48.73	149.76
−8	−6	4	21.26	116.61
7	−4	2	1968.50	179.34
23	−3	8	−59.82	167.72
22	−3	10	−103.01	182.90
24	−2	8	−48.33	169.57
20	−2	4	178.68	144.34
−27	0	9	−1.06	177.23
23	1	4	−111.33	171.15
18	1	2	−18.75	144.87
−27	1	5	−69.46	174.98
−28	1	10	−57.97	178.81
19	2	2	−33.68	138.80
−13	2	0	80.43	101.69
20	3	2	23.51	152.27
13	4	0	491.27	137.34
−30	8	1	−10.43	165.74
−34	9	2	138.14	186.34
−36	9	3	−94.16	174.06
24	10	2	154.78	203.37
−37	13	1	−40.94	183.96
−27	14	−2	3.83	145.53
18	22	−1	9.11	171.42
8	−7	12	−79.50	138.40
−10	−7	6	163.10	104.33
20	−5	7	140.25	182.11
−16	−5	10	−37.77	141.17
17	−4	12	−57.71	181.45
−17	−4	5	200.20	124.27
−17	−4	11	−146.59	155.70
21	−1	4	−12.81	138.27
22	0	4	141.31	144.61
25	2	5	−172.08	192.68
−29	3	4	17.56	197.30
−32	3	7	0.00	183.17
19	6	1	−3.83	144.08

9	11	−2	204.69	134.57
22	12	1	90.73	202.98
10	12	−2	0.00	117.01
−32	14	−1	−16.64	180.53
−38	16	0	135.76	183.96
−14	17	−4	301.23	95.88
−29	19	−3	117.40	162.70
−39	23	−2	−7.92	169.83
15	−7	10	−12.55	153.72
−3	−7	4	1523.06	147.78
12	−6	12	43.45	158.47
19	−5	6	−28.79	135.36
−17	−5	9	35.26	137.74
19	−4	11	31.43	181.45
15	2	1	494.04	157.02
−29	2	5	−113.57	177.75
14	5	0	417.97	160.32
24	6	3	−67.62	208.00
15	6	0	−75.94	152.27
16	7	0	84.78	129.16
−33	12	0	236.92	194.66
16	14	−1	174.45	140.91
−29	15	−2	48.20	155.30
9	21	−3	−4.23	143.81
14	22	−2	−70.78	182.11
−32	26	−4	−6.21	174.85
−39	27	−3	−22.85	180.53
−8	−7	5	43.45	89.93
18	−6	9	35.66	158.21
21	−4	7	93.90	188.85
−15	−4	4	40.41	120.04
18	−3	12	−64.97	183.70
−23	−2	6	−118.99	171.15
25	0	7	−47.81	185.68
−14	0	1	1573.64	186.60
25	1	6	0.00	183.56
24	1	5	110.80	207.07
−21	1	2	122.29	137.08
−29	1	9	25.49	185.15
−23	2	2	87.95	150.81
16	3	1	216.84	157.02
−25	3	2	56.79	141.57
−31	4	4	−65.24	181.45
18	5	1	−47.01	151.61
−32	9	1	0.00	198.09
−36	10	2	−109.21	185.68
13	18	−2	26.68	147.64
−23	20	−4	130.34	125.85
−36	20	−2	5.02	196.77
−36	24	−3	42.26	172.34
4	−9	10	83.73	68.67
−8	−8	9	87.29	98.39
10	−5	3	605.50	109.35
20	−4	6	28.79	147.38

−5	−4	2	1602.82	148.17
22	−3	7	0.00	188.72
20	−3	11	166.53	189.24
15	−3	3	156.76	144.47
−10	−3	2	304.53	98.25
−21	−3	6	185.41	157.68
−22	−3	8	16.90	154.91
23	−2	7	−87.95	188.98
−24	−2	8	−20.60	157.02
24	−1	7	13.87	173.13
9	−1	1	10.83	78.31
−25	−1	6	−136.29	175.51
−26	−1	8	−111.86	173.13
23	0	5	210.24	180.40
17	4	1	213.01	144.47
23	9	2	−36.32	205.35
−10	11	−3	124.93	86.90
−15	12	−3	121.10	127.04
19	13	0	−60.62	163.49
−34	15	−1	251.84	199.54
20	17	0	−167.85	191.36
17	18	−1	0.00	197.17
−21	19	−4	79.24	125.99
−39	19	−1	21.66	180.66
−31	20	−3	91.25	195.85
−25	21	−4	59.30	136.02
13	−8	9	124.53	146.19
16	−7	8	174.45	178.68
−19	−4	6	−9.24	150.42
−20	−4	8	−93.90	149.10
5	−2	1	1192.25	132.85
22	−1	5	−80.56	160.98
24	0	6	0.00	198.36
−3	0	0	8881.10	723.43
−19	0	2	287.63	138.93
−27	0	6	−18.49	165.74
−31	3	5	142.49	182.90
−27	4	2	−71.58	151.21
23	5	3	−29.32	176.30
−35	13	0	−42.39	199.81
−31	16	−2	−21.26	167.45
−27	22	−4	18.36	155.83
11	−8	10	37.37	135.76
14	−5	4	136.42	135.89
−15	−5	5	144.08	122.55
21	−3	6	0.00	167.59
22	−2	6	197.83	194.79
23	−1	6	36.05	197.83
−28	0	8	13.21	179.34
−29	1	6	−102.22	173.00
−12	4	−1	224.37	92.05
7	5	−1	844.93	110.01
−29	5	2	0.00	174.58
−33	5	4	0.00	175.38

25	6	4	223.32	193.21
−34	10	1	68.67	196.24
15	13	−1	66.16	142.49
22	15	1	47.28	189.51
−19	18	−4	52.16	117.67
20	20	0	28.00	183.43
−14	24	−5	−42.13	85.18
−34	27	−4	−52.56	176.43
−3	−8	5	141.31	72.77
−12	−7	7	117.27	124.93
−15	−6	9	16.11	129.16
19	−5	9	−64.84	165.60
−15	−5	11	50.18	144.74
−18	−5	8	−86.10	149.63
−33	4	5	−40.01	197.04
−22	5	0	294.50	145.00
−24	6	0	−5.81	144.21
−26	7	0	222.39	151.47
−23	8	−1	76.33	146.98
−25	9	−1	−66.29	149.89
8	20	−3	166.13	130.21
17	21	−1	194.26	178.55
−33	21	−3	0.00	193.87
−38	21	−2	101.03	177.89
−29	23	−4	−98.78	182.24
17	24	−1	−126.65	179.87
−3	−9	9	167.45	37.64
−4	−9	8	211.30	55.07
−9	−8	8	−26.68	111.46
14	−7	6	575.79	134.70
16	−6	10	−118.59	162.96
14	−6	11	44.24	163.36
−14	−6	10	−16.77	132.85
−17	−5	6	0.00	136.82
16	−2	3	115.69	156.62
−17	−1	2	285.65	153.98
−30	1	8	−33.15	186.73
22	4	3	60.88	152.40
−20	4	0	48.60	158.87
−21	7	−1	523.75	171.55
22	8	2	30.64	172.34
−27	10	−1	69.60	154.51
18	12	0	64.05	151.74
0	12	−3	893.13	101.95
−36	16	−1	−34.86	188.19
12	17	−2	7.26	136.68
−33	17	−2	0.00	193.47
−38	25	−3	179.34	177.62
3	−9	6	41.34	31.03
6	−8	11	−38.83	120.57
15	−7	7	30.51	132.46
−13	−5	4	−54.67	113.57
20	−4	9	−0.13	165.08
12	−2	2	258.58	109.61

23	−1	9	3.70	181.32
−11	1	0	487.57	99.57
−31	2	6	187.26	177.62
−7	3	−1	2344.22	213.67
−31	6	2	194.53	202.05
−35	6	4	−138.53	183.56
−28	8	0	175.51	147.38
−37	14	0	0.00	189.64
13	21	−2	71.58	193.34
5	−7	12	−106.31	129.42
14	−6	5	107.23	115.95
13	−5	12	−83.86	173.00
21	−3	9	−83.46	183.96
22	−2	9	−50.18	181.72
−7	−2	1	1540.76	151.47
21	3	3	182.24	149.36
24	5	4	0.00	199.68
−19	6	−1	133.51	163.89
24	9	3	0.00	207.60
20	10	1	13.87	146.46
−29	11	−1	117.27	143.68
−36	11	1	121.63	197.30
23	12	2	0.00	205.88
14	12	−1	54.01	141.83
−22	14	−3	−18.36	129.68
−24	15	−3	143.02	140.12
19	16	0	0.00	196.90
−26	16	−3	142.76	140.64
16	17	−1	115.55	178.81
−17	24	−5	125.85	106.04
−31	24	−4	−45.96	191.88
−36	28	−4	−9.90	178.81
17	−6	8	11.89	174.45
3	−6	3	713.92	70.92
−12	−1	1	377.30	113.84
20	2	3	124.67	150.95
−18	3	0	297.14	155.96
−33	3	6	−178.41	184.49
25	5	5	−13.07	194.53
21	7	2	−24.43	158.61
−33	7	2	94.82	198.62
−30	9	0	141.97	156.76
−17	17	−4	107.50	103.93
−35	18	−2	0.92	209.32
7	19	−3	172.34	123.74
−35	22	−3	−1.45	194.26
13	24	−2	−56.65	175.24
8	−9	8	5.15	109.87
−7	−8	10	−108.16	90.07
17	−5	10	−43.05	174.72
−23	−3	7	44.64	163.23
−25	−2	7	49.52	173.66
18	0	3	214.20	129.02
19	1	3	37.77	151.47

8	2	0	49.92	78.44
−23	3	1	111.33	138.40
−25	4	1	145.40	150.95
−27	5	1	0.00	142.23
17	11	0	240.75	142.36
−31	12	−1	255.14	176.04
−20	13	−3	−48.73	125.99
21	14	1	−27.34	205.75
−28	17	−3	215.66	163.76
7	−9	7	83.33	87.82
12	−8	8	−58.77	143.42
10	−8	6	−55.33	102.35
−13	−6	5	−14.53	112.91
15	−4	4	112.12	144.61
−15	−2	2	81.09	145.27
−27	−1	7	−18.88	179.60
−21	2	1	0.00	131.40
23	4	4	139.59	185.68
8	6	−1	400.14	97.59
−29	6	1	79.63	154.64
3	8	−2	646.31	85.84
−32	10	0	−148.57	195.45
13	11	−1	−25.62	132.06
−13	11	−3	−59.43	109.61
−12	16	−4	134.04	91.65
−30	18	−3	105.91	170.23
19	19	0	−23.77	187.79
16	20	−1	96.27	190.83
−33	25	−4	92.18	182.11
−40	26	−3	−20.21	182.51
6	−8	5	199.02	90.73
14	−7	9	76.60	157.55
10	−7	11	−3.04	147.25
−15	−6	6	−15.45	124.14
18	−5	8	−32.62	171.42
15	−5	11	−56.52	172.34
−21	−4	7	0.26	155.96
10	0	1	270.59	89.54
−6	0	0	2851.86	255.67
−29	0	7	339.13	188.32
−17	5	−1	492.59	143.81
20	6	2	57.84	157.42
−13	7	−2	96.93	97.86
23	8	3	152.27	208.79
−35	8	2	135.23	184.36
19	9	1	0.00	141.70
−38	12	1	−111.59	181.98
−28	13	−2	73.43	153.45
8	23	−3	74.61	179.87
−22	25	−5	110.40	132.99
16	26	−1	−17.30	184.75
−13	−7	9	−103.27	131.40
16	−6	7	81.61	137.74
15	−6	6	221.86	132.33

9	−6	12	33.28	156.49
18	−4	10	43.58	179.47
14	−4	12	−51.11	185.41
13	−1	2	22.05	110.14
−24	−1	4	74.88	175.24
23	0	8	18.22	187.00
−26	0	4	25.75	188.72
−19	1	1	−27.47	155.57
22	3	4	−75.94	162.17
24	4	5	−10.70	197.83
−31	7	1	4.36	184.36
12	10	−1	52.56	143.81
22	11	2	0.00	226.88
−33	13	−1	−187.26	193.60
−30	14	−2	141.83	156.89
18	15	0	177.36	194.66
15	16	−1	−22.32	154.25
−9	16	−4	180.79	74.35
21	17	1	322.23	201.39
−37	19	−2	121.10	196.37
12	20	−2	138.40	171.55
16	23	−1	−6.07	173.26
−37	23	−3	0.00	192.02
11	−8	7	207.07	115.82
−8	−8	6	38.56	92.44
−3	−6	3	33.94	55.33
−13	−6	11	−26.94	136.42
15	−5	5	13.87	119.52
−19	−5	7	45.83	155.17
19	−4	8	43.58	173.92
19	−3	10	9.38	177.36
−22	−2	4	−24.04	154.51
−28	1	4	170.09	198.36
−16	2	0	223.84	125.06
16	10	0	342.30	145.93
−34	11	0	120.57	202.05
−18	12	−3	96.67	141.04
6	18	−3	−25.09	117.67
−24	19	−4	−103.67	129.82
−32	19	−3	−112.91	198.88
−26	20	−4	49.79	129.02
−20	24	−5	42.66	119.12
12	−7	10	−106.57	145.93
16	−4	11	73.95	177.62
11	−4	3	1214.57	146.72
20	−3	8	72.77	177.23
16	−3	4	134.44	133.51
21	−2	8	−0.13	179.47
20	−2	10	38.56	195.05
−24	−2	5	157.15	186.07
22	−1	8	0.00	171.94
−26	−1	5	−48.99	182.64
24	2	7	0.00	179.47
21	2	4	151.61	150.55

-30	2	4	-20.34	189.90
19	5	2	132.85	146.98
18	8	1	129.02	167.19
10	8	-1	59.16	117.27
11	9	-1	245.11	148.83
20	13	1	-112.12	218.16
10	15	-2	121.23	127.17
-28	21	-4	-78.97	164.02
-35	26	-4	0.00	177.09
3	-8	11	59.96	103.14
10	-7	5	337.81	116.87
2	-7	12	92.71	113.84
-12	-7	10	71.84	128.36
-20	-3	4	129.42	135.49
-25	-2	10	157.15	191.09
-27	-1	10	-115.69	196.37
14	0	2	1122.78	166.79
-28	0	5	11.62	183.43
23	3	5	188.32	201.00
22	7	3	53.62	169.04
24	8	4	-87.69	219.62
-33	8	1	108.69	201.39
-37	9	2	-65.50	184.62
-35	14	-1	170.23	206.28
-32	15	-2	237.97	187.79
-15	16	-4	247.88	98.39
-22	18	-4	-30.11	129.68
12	23	-2	143.29	195.19
-10	-8	7	-31.17	111.46
15	-6	9	81.88	155.44
-11	-6	4	5.94	107.89
17	-5	7	91.52	155.96
-8	-4	2	94.03	79.90
17	-3	11	-121.50	188.45
15	-3	12	-5.02	189.64
-22	-3	5	59.56	179.47
-23	-3	10	0.00	175.51
17	-2	4	8.32	128.36
-17	0	1	207.34	145.40
23	1	7	-88.22	181.85
20	1	4	-179.60	164.81
-30	1	5	-40.01	186.07
9	3	0	587.80	105.65
-10	3	-1	1134.40	126.38
-32	3	4	47.54	191.36
18	4	2	100.23	137.48
-18	8	-2	-65.11	138.53
-36	12	0	-100.50	199.94
18	18	0	0.00	215.79
-34	20	-3	-185.28	210.51
-39	20	-2	-8.06	189.64
-30	22	-4	8.32	193.73
-39	24	-3	0.00	179.47
10	-8	9	-35.66	138.27

8	−8	10	4.62	125.33
−17	−6	7	53.88	144.47
16	−4	5	91.12	131.40
−24	−3	9	−51.11	171.94
−26	−2	9	0.13	180.00
18	−1	4	−26.54	144.08
−28	−1	9	−175.91	200.86
19	0	4	37.24	155.17
15	1	2	388.92	148.44
22	2	5	61.54	204.69
17	3	2	59.82	148.83
−15	4	−1	293.84	115.82
17	7	1	242.33	148.44
−35	9	1	166.13	188.32
21	10	2	0.00	177.89
1	13	−3	147.25	88.22
14	15	−1	23.90	140.78
−34	16	−2	1.06	205.09
5	17	−3	6.34	104.06
15	19	−1	0.00	193.34
1	23	−4	−14.00	110.27
−15	23	−5	−20.87	89.41
−37	27	−4	72.24	182.77
−6	−7	4	41.73	96.93
18	−4	7	−88.35	165.60
−21	−4	10	0.00	164.15
−22	−4	9	90.73	166.66
8	−3	2	696.62	94.42
−13	−3	2	291.19	132.99
18	−2	11	−50.84	192.28
16	−2	12	133.91	199.41
22	0	7	−31.83	183.70
−30	0	9	0.00	215.92
11	1	1	110.40	100.63
−24	1	2	77.92	147.12
16	2	2	135.89	157.02
−26	2	2	97.86	150.42
−32	2	5	−21.92	185.02
−28	3	2	0.00	184.23
23	11	3	223.58	210.11
17	14	0	41.47	159.53
9	14	−2	−71.97	119.12
−37	15	−1	103.67	198.75
20	16	1	152.27	199.15
−20	17	−4	−100.76	124.67
11	19	−2	−95.35	140.12
18	21	0	169.43	182.77
7	22	−3	60.22	144.61
−32	23	−4	−104.72	193.87
13	−7	8	−73.03	166.93
−13	−7	6	−77.26	112.12
−18	−4	4	36.71	127.44
19	−3	7	220.54	189.90
20	−2	7	−235.73	201.00

21	−1	7	0.00	187.92
−22	0	2	135.63	154.64
22	1	6	56.52	215.00
21	1	5	−101.03	158.08
−30	4	2	0.00	202.98
−34	4	4	−65.90	194.13
21	6	3	−6.60	165.60
−8	6	−2	1294.99	126.91
23	7	4	−203.51	223.18
14	8	0	205.62	147.91
−26	8	−1	80.69	149.10
4	9	−2	−8.06	77.92
−16	11	−3	−100.23	129.29
−38	13	0	−192.28	199.41
−36	21	−3	0.00	182.24
15	22	−1	3.17	183.96
−18	23	−5	69.07	107.37
15	25	−1	0.00	171.68
−5	−9	7	61.54	72.24
−11	−7	5	206.68	110.80
11	−6	11	149.23	158.87
16	−5	9	0.00	166.00
17	−4	6	−160.59	140.38
−24	−3	6	−113.97	171.55
−10	−2	1	1186.17	135.49
−26	−2	6	−90.33	172.34
−28	−1	6	−53.22	172.08
21	0	6	5.68	201.13
20	0	5	90.07	146.59
−14	1	0	269.40	114.23
−34	3	5	81.48	201.00
10	4	0	1593.84	174.58
3	4	−1	2863.61	246.29
−25	5	0	171.15	152.27
−34	5	3	0.00	173.53
16	6	1	314.17	138.27
−27	6	0	−3.17	152.79
24	7	5	−93.10	187.13
−24	7	−1	80.56	155.70
−28	9	−1	182.24	155.44
−37	10	1	1.19	182.24
4	16	−3	117.14	97.99
−36	17	−2	108.03	197.17
20	19	1	−62.86	182.38
−25	25	−5	18.09	176.57
13	−6	10	−75.54	156.62
10	−5	12	139.59	171.28
−20	−5	9	−76.99	158.61
4	−4	2	2376.97	203.11
18	−3	6	54.54	138.93
−25	−3	8	−82.80	173.66
−27	−2	8	370.17	196.77
20	−1	6	−20.87	183.43
19	−1	5	123.61	141.57

-9	0	0	1249.56	136.95
-30	0	6	-36.45	180.26
12	2	1	1052.66	151.47
-23	4	0	194.79	145.00
15	5	1	233.88	158.61
-32	5	2	-89.80	205.49
13	7	0	85.71	154.12
-29	7	0	258.44	161.51
20	9	2	-122.55	156.36
-30	10	-1	25.88	160.98
8	13	-2	-132.85	125.19
2	14	-3	58.90	101.69
-25	14	-3	94.82	136.02
3	15	-3	0.00	99.84
-27	15	-3	56.92	146.19
-29	16	-3	43.32	150.95
-39	16	-1	0.00	168.64
-23	24	-5	103.01	157.55
-34	24	-4	184.49	190.17
0	-8	11	9.38	78.71
-18	-5	5	148.57	126.65
-19	-5	10	-28.13	160.98
-22	-4	6	12.81	168.77
12	-3	3	241.67	115.55
19	-2	6	-35.92	148.57
-15	-1	1	224.24	136.82
-20	-1	2	43.98	134.70
-29	-1	8	0.00	196.90
13	3	1	295.55	136.42
14	4	1	794.48	163.49
20	5	3	-103.80	159.13
11	5	0	1170.98	158.21
12	6	0	341.25	138.66
-22	6	-1	-73.69	140.38
-36	6	3	61.28	184.23
-16	7	-2	241.28	122.42
-31	8	0	-6.07	186.87
-32	11	-1	62.99	184.09
-23	13	-3	232.56	137.87
13	14	-1	53.62	136.02
-38	22	-3	-76.99	188.98
-6	-8	5	30.24	77.12
11	-7	6	228.33	111.99
7	-5	3	200.47	71.84
17	-4	9	102.74	175.24
-23	-4	8	113.70	164.68
21	0	9	9.38	183.56
-31	0	8	1.06	200.07
-32	1	6	5.94	194.39
-21	3	0	61.41	148.04
22	6	4	18.49	205.35
-34	6	2	101.03	207.20
22	10	3	151.08	214.60
5	10	-2	352.21	97.33

7	12	−2	430.92	131.27
21	13	2	87.95	215.39
16	13	0	55.99	138.80
−18	16	−4	51.90	108.69
17	17	0	−102.48	201.92
−31	17	−3	−63.65	197.04
14	18	−1	2.11	161.11
11	22	−2	20.60	193.21
0	22	−4	21.00	94.56
12	−7	7	146.46	116.21
−15	−7	7	113.18	143.29
11	−5	4	366.87	122.55
−16	−5	4	113.04	125.99
18	−3	9	−13.87	171.42
19	−2	9	−100.89	181.98
20	−1	9	125.72	179.87
4	1	0	969.72	103.67
−20	5	−1	336.36	159.00
23	6	5	230.58	194.79
−11	6	−2	497.47	97.33
−33	9	0	−130.08	210.64
−11	10	−3	135.76	90.59
6	11	−2	173.53	113.31
−21	12	−3	53.09	129.95
19	15	1	152.93	207.20
10	18	−2	−31.96	135.23
−38	18	−2	66.69	187.13
6	21	−3	30.37	129.55
11	25	−2	31.69	186.60
−36	25	−4	−189.51	196.24
5	−9	8	2.64	91.25
7	−7	11	77.12	134.57
14	−6	8	−122.95	171.81
−20	−5	6	−15.45	153.72
−21	−5	8	−54.15	161.11
−26	3	1	66.43	161.25
19	4	3	75.27	150.55
−28	4	1	328.04	161.77
−36	7	2	92.31	190.83
19	8	2	−92.97	153.72
−34	12	−1	−102.22	209.18
−33	18	−3	72.11	215.79
−27	19	−4	38.69	146.85
17	20	0	−0.13	190.56
17	23	0	−72.11	172.87
−21	23	−5	3.04	127.70
14	27	−1	54.28	190.56
−11	−8	9	−9.77	116.21
6	−6	12	−137.34	152.93
−18	−6	9	46.62	153.06
14	−5	10	122.42	167.59
−18	−2	2	41.60	125.85
−24	2	1	11.49	139.98
−13	3	−1	64.05	100.23

-30	5	1	-152.66	176.83
-23	9	-2	247.22	128.23
23	10	4	0.00	196.90
-35	10	0	111.33	208.52
21	16	2	0.00	213.15
-25	18	-4	7.13	134.70
-29	20	-4	112.52	178.02
14	21	-1	102.88	208.13
-12	23	-5	12.94	66.69
9	-8	8	0.66	123.87
11	-6	5	66.56	126.12
-16	-6	5	0.00	120.44
12	-5	11	-59.96	170.75
13	-2	3	79.76	123.74
6	-1	1	995.08	116.48
22	2	8	9.11	187.00
-19	2	0	132.72	146.98
18	3	3	122.29	143.15
23	4	7	16.77	196.24
23	5	6	15.45	185.68
21	5	4	-20.21	170.49
-32	6	1	-15.85	229.26
-8	10	-3	228.07	66.16
-36	13	-1	-54.54	204.56
-33	14	-2	68.28	194.39
-23	17	-4	-87.42	127.57
19	18	1	-127.17	210.11
-35	19	-3	-153.72	193.07
-31	21	-4	18.62	196.77
14	24	-1	-134.04	193.73
-38	26	-4	19.81	191.62
4	-9	7	0.00	69.07
11	-7	9	57.05	151.61
-17	-6	10	-0.13	155.83
11	-4	12	98.65	179.47
-22	1	1	-25.49	132.72
22	5	5	5.02	209.71
-21	8	-2	-65.24	133.38
21	9	3	-7.40	188.85
20	12	2	0.00	195.58
15	12	0	-33.15	140.91
22	13	3	65.11	196.24
-1	21	-4	90.73	85.44
-7	-9	8	-84.52	105.52
-18	-6	6	0.00	146.59
15	-5	8	104.59	184.36
15	-4	10	-111.33	177.49
-11	-4	2	108.55	103.93
14	-1	3	104.72	147.12
-30	-1	7	158.34	205.22
21	1	8	2.77	180.92
17	2	3	46.22	132.19
-18	4	-1	581.60	159.13
18	7	2	-63.92	147.64

−34	7	1	62.20	189.51
17	10	1	−0.13	149.76
−14	10	−3	223.71	113.70
−19	11	−3	−58.37	127.57
−37	11	0	72.63	185.55
−13	15	−4	71.71	98.52
−35	15	−2	−85.18	206.54
16	16	0	−52.56	158.34
−16	22	−5	−3.30	95.88
−33	22	−4	−210.11	189.24
7	−8	6	24.17	80.29
9	−7	10	0.00	136.15
−24	−4	7	180.92	169.17
9	−2	2	437.39	91.52
−25	−2	4	43.18	188.58
−27	−1	4	0.00	189.64
15	0	3	70.26	149.23
−12	0	0	463.14	101.29
−27	0	3	0.00	185.55
−29	0	4	−20.87	184.62
−32	0	7	−109.08	204.96
16	1	3	−40.94	138.40
−29	1	3	150.29	207.86
22	3	7	−67.22	188.85
20	4	4	78.18	152.53
18	14	1	−36.05	177.89
−38	14	−1	223.84	197.30
13	17	−1	21.92	137.74
−37	20	−3	−159.13	186.21
10	21	−2	−49.79	155.44
−19	22	−5	87.95	109.21
6	24	−3	0.00	167.59
−26	24	−5	−73.69	185.68
−3	−9	6	45.43	59.56
−11	−8	6	23.64	105.65
−11	−7	11	68.54	118.33
13	−6	7	125.46	124.67
13	−4	11	−29.45	177.09
12	−4	4	−25.62	125.33
16	−3	10	−162.17	194.66
−25	−1	3	−0.66	158.34
20	0	8	183.96	184.36
−20	0	1	43.98	126.65
−31	1	4	−47.15	191.49
−31	2	3	115.95	192.28
−14	6	−2	101.95	103.14
−36	8	1	313.91	200.07
22	9	4	−209.18	227.41
11	12	−1	136.15	121.23
−16	15	−4	204.30	104.86
−21	16	−4	168.38	127.84
9	17	−2	−20.60	131.27
5	20	−3	−85.97	118.19
−35	23	−4	40.28	177.49

−40	27	−4	114.36	199.68
8	−8	7	42.92	95.74
−9	−7	4	409.39	115.69
12	−6	6	146.19	115.42
−14	−6	4	99.71	117.80
−22	−5	7	−85.05	163.62
16	−4	8	26.02	188.32
12	−3	12	−18.36	191.75
−16	−3	2	192.68	140.64
−23	−3	4	24.04	173.40
−13	−2	1	442.80	115.82
−27	−2	5	234.67	179.21
19	−1	8	−101.82	178.55
−29	−1	5	−57.45	174.72
−17	1	0	16.64	144.34
−33	2	4	54.15	178.28
−33	3	3	0.00	186.07
21	4	5	464.46	198.09
−19	7	−2	290.40	149.89
20	8	3	23.24	152.79
−37	16	−2	−158.74	209.32
16	19	0	14.39	223.18
−24	23	−5	45.43	159.93
10	24	−2	0.00	196.77
5	−8	10	−28.79	114.76
−16	−7	9	−92.31	145.00
17	−3	8	243.78	185.02
−25	−3	5	−42.26	178.28
18	−2	8	−66.43	183.83
−23	−2	3	48.73	141.70
−31	0	5	−77.26	183.56
21	2	7	159.66	188.98
19	3	4	131.66	160.72
17	6	2	0.00	139.46
16	9	1	281.29	141.31
14	11	0	−165.21	145.27
−28	14	−3	−13.73	138.66
−30	15	−3	−4.23	175.64
−2	20	−4	28.53	77.39
−39	21	−3	57.97	182.64
16	22	0	5.68	191.09
−6	−9	9	14.13	79.90
7	−8	9	0.00	117.14
−13	−8	7	−74.75	128.89
12	−5	5	145.27	116.61
−14	−5	3	76.60	102.61
14	−3	11	−49.52	184.75
13	−2	12	−162.83	197.56
−27	1	2	28.39	173.00
−33	1	5	202.05	202.58
−29	2	2	41.47	195.58
−31	3	2	143.68	204.03
−35	3	4	−173.13	192.68
−35	4	3	15.32	195.19

−27	7	−1	83.86	155.44
−29	8	−1	−40.01	148.44
−31	9	−1	67.75	173.66
19	11	2	−183.30	160.98
21	12	3	−47.28	210.24
−26	13	−3	−43.84	142.49
−32	16	−3	0.00	195.85
18	17	1	183.43	222.79
13	20	−1	127.17	181.58
−37	24	−4	129.68	188.85
13	26	−1	−103.93	184.75
−10	−8	10	117.93	110.54
−17	−7	8	38.69	146.72
14	−5	7	−73.95	127.84
−21	−4	4	52.69	144.08
−23	−4	5	−106.04	191.36
15	−2	11	190.70	194.66
16	−1	11	40.01	195.85
17	0	11	−94.56	205.88
−25	0	2	−100.50	153.85
20	1	7	43.71	189.24
20	3	5	53.88	170.62
−26	4	0	−11.36	150.15
−33	4	2	−141.83	194.00
−28	5	0	239.69	148.30
−25	6	−1	0.00	146.98
−30	6	0	−141.57	173.92
−17	10	−3	245.77	138.40
−24	12	−3	102.74	135.76
−10	15	−4	44.77	80.16
−39	17	−2	−215.79	205.09
18	20	1	8.85	188.19
−22	22	−5	93.63	131.27
13	23	−1	−1.06	202.45
−9	−8	5	99.18	92.44
−14	−7	5	220.94	116.74
−16	−7	6	67.35	135.10
8	−6	11	38.30	149.49
−20	−6	7	−46.88	159.00
13	−3	4	268.08	137.87
−5	−3	1	126.38	67.62
−21	−3	3	46.75	147.12
−29	−2	9	110.93	211.17
14	−1	12	−57.05	206.94
−18	−1	1	104.20	147.91
15	0	12	−49.52	206.28
18	2	4	−39.88	139.72
−16	3	−1	290.40	130.61
16	5	2	85.97	135.10
4	5	−1	851.00	95.22
−32	7	0	−192.02	209.98
22	8	5	−77.26	206.94
21	8	4	24.17	195.19
−33	10	−1	−108.69	204.30

10	11	−1	94.03	122.68
15	15	0	1.19	137.08
−19	15	−4	−59.30	113.31
12	16	−1	0.00	130.87
8	16	−2	83.59	115.69
−34	17	−3	−163.76	204.17
−28	18	−4	−71.45	162.96
−8	−9	7	58.37	92.84
−15	−7	10	−52.43	144.34
13	−5	6	130.48	121.63
7	−5	12	272.97	168.51
−27	−3	9	21.79	192.02
10	−1	2	61.80	83.86
−23	−1	2	2.11	146.32
19	0	7	119.38	200.86
17	1	4	98.78	130.21
−8	2	−1	1246.00	129.16
−24	3	0	52.69	143.29
−23	5	−1	160.32	138.80
−35	5	2	94.29	207.60
19	7	3	153.19	150.95
15	8	1	73.03	129.02
−34	8	0	−154.78	212.88
−28	10	−2	111.72	143.29
−35	11	−1	78.18	198.49
−26	17	−4	−81.75	139.85
−3	19	−4	189.90	64.05
−30	19	−4	−49.79	185.94
−32	20	−4	58.77	189.24
−39	25	−4	20.87	194.39
3	−8	5	169.17	59.82
10	−7	8	165.47	134.83
7	−7	5	1176.53	139.06
−21	−5	5	−29.19	179.74
15	−4	7	42.26	132.72
−24	−4	10	3.30	188.45
−25	−4	9	64.71	182.38
−8	−3	1	363.83	78.71
−27	−3	6	−57.31	179.34
14	−2	4	−84.92	141.17
−29	−2	6	−87.16	191.75
−31	−1	6	47.67	190.43
20	2	9	−24.43	193.34
19	2	5	−151.21	149.49
−26	9	−2	146.59	157.28
−22	11	−3	113.70	127.04
−4	18	−4	185.94	49.26
−36	18	−3	149.23	186.73
4	19	−3	147.25	113.44
9	20	−2	41.86	130.21
−13	22	−5	75.14	74.61
10	−6	10	118.06	146.59
13	−5	9	14.53	162.83
−19	−5	4	−47.67	132.06

13	−4	5	0.00	116.35
8	−4	3	289.21	76.46
−25	−4	6	0.00	171.15
16	−3	7	−3.04	142.23
17	−2	7	0.00	156.76
18	−1	7	129.02	190.96
15	−1	4	66.82	125.72
16	0	4	42.13	127.84
−33	0	6	−112.91	200.34
19	1	6	58.50	166.66
−11	2	−1	652.65	100.89
−17	6	−2	264.91	140.38
22	7	6	−128.10	188.19
−36	9	0	−173.92	202.19
18	10	2	28.13	145.40
9	10	−1	444.25	135.76
20	11	3	58.64	190.43
−37	12	−1	20.34	193.87
19	14	2	−119.91	204.03
−24	16	−4	121.50	129.68
−34	21	−4	−170.62	188.19
5	23	−3	9.51	133.91
−29	24	−5	−2.51	188.72
−6	−9	6	140.25	81.48
3	−6	12	−43.98	140.38
−14	−4	2	210.11	134.57
−19	−4	3	38.43	134.31
−28	−3	8	−153.98	201.66
−21	−2	2	73.16	143.42
−30	−2	8	33.68	209.58
11	0	2	456.01	100.63
7	0	1	1160.95	125.33
−15	0	0	223.32	129.29
19	1	9	20.34	182.77
18	1	5	139.46	147.51
−22	2	0	36.71	127.84
−27	2	1	188.72	146.72
−29	3	1	175.38	174.45
21	4	8	55.86	185.15
−21	4	−1	269.01	138.53
−31	4	1	67.88	223.32
20	7	4	−54.01	165.74
−24	8	−2	64.84	137.87
15	18	0	0.00	190.56
−17	21	−5	85.71	101.16
−27	23	−5	−50.98	178.41
15	24	0	195.45	180.00
12	28	−1	−39.22	194.00
4	−7	11	41.47	127.84
3	−7	4	1219.32	112.78
−6	−5	2	493.64	78.58
−23	−5	9	−126.65	176.70
14	−4	6	228.20	129.55
−26	−4	8	−92.84	183.04

18	0	6	109.21	145.93
5	2	0	4.49	82.14
14	3	2	346.00	137.61
−33	5	1	82.41	218.83
18	6	3	−41.73	149.23
21	7	5	177.23	221.47
12	9	0	127.70	142.89
−36	14	−2	200.34	214.86
17	16	1	155.70	188.72
19	17	2	−93.37	200.73
−38	19	−3	−72.77	187.26
15	21	0	0.00	204.17
−20	21	−5	11.36	111.72
−36	22	−4	−59.43	185.41
9	23	−2	−33.94	182.11
9	26	−2	0.00	182.38
−18	−7	7	64.58	146.59
−22	−5	10	19.02	186.73
−23	−5	6	145.27	171.55
14	−4	9	−18.09	171.28
15	−3	6	−56.39	133.25
−16	−2	1	71.71	139.72
17	−1	6	−31.30	135.89
18	0	9	17.17	176.70
17	0	5	256.99	143.29
12	1	2	332.00	111.72
−25	1	1	−10.70	137.74
13	2	2	402.13	129.82
5	6	−1	74.75	63.26
−35	6	1	84.65	181.72
8	9	−1	−5.68	110.54
21	11	4	81.75	198.62
14	14	0	110.67	132.19
7	15	−2	−25.75	107.76
12	19	−1	0.00	143.29
−25	22	−5	−123.34	166.53
−19	−6	5	75.54	143.15
−24	−5	8	106.18	176.30
15	−3	9	0.00	167.59
16	−2	6	251.71	138.00
17	−1	9	−26.28	169.83
16	−1	5	2.51	127.70
−5	2	−1	1239.13	128.36
21	5	7	−22.85	181.98
21	6	6	93.24	192.41
−22	7	−2	−2.77	123.61
−12	9	−3	350.23	99.97
−20	10	−3	−2.91	118.06
20	14	3	0.13	197.96
11	15	−1	190.70	128.63
−22	15	−4	198.09	122.82
−38	15	−2	−254.88	194.13
17	19	1	−248.41	200.60
17	22	1	−98.39	191.22

12	22	−1	−154.51	196.64
12	25	−1	217.37	183.83
−12	−7	4	386.54	111.86
11	−5	10	−17.30	152.27
9	−5	11	3.17	152.79
8	−4	12	−26.28	171.94
16	−2	9	1.58	168.51
−7	−1	0	775.59	102.22
−23	0	1	420.35	156.10
−20	1	0	85.71	131.27
20	3	8	−93.50	178.41
−9	5	−2	1420.18	138.00
−12	5	−2	294.76	91.25
13	6	1	−16.24	139.32
17	9	2	−55.60	149.63
−14	14	−4	551.22	111.99
−17	14	−4	304.00	108.03
−38	23	−4	38.69	184.09
−14	−8	6	34.20	121.10
−14	−8	9	−13.73	132.85
−15	−8	8	49.79	136.02
9	−7	7	101.82	104.33
11	−6	8	50.71	140.64
−12	−6	3	91.91	121.23
−21	−6	6	152.66	158.47
−21	−6	9	22.05	163.49
−3	−5	2	790.39	81.75
−11	−3	1	1743.07	179.07
−19	−3	2	57.45	126.51
−14	2	−1	713.66	125.33
−19	3	−1	332.79	147.78
17	5	3	54.01	139.85
20	6	5	110.40	186.34
19	6	4	24.83	154.78
11	8	0	230.71	135.49
−15	9	−3	48.47	117.40
19	10	3	0.00	173.26
18	13	2	133.25	168.38
−29	13	−3	78.97	146.46
−31	14	−3	92.71	175.77
3	18	−3	203.64	100.50
8	19	−2	78.71	122.82
−23	21	−5	30.11	137.08
−17	−6	4	151.08	123.61
−9	−5	2	99.44	88.74
−17	−5	3	241.01	123.87
−29	−3	7	−236.65	204.43
−28	−2	4	0.92	193.47
−31	−2	7	207.20	199.94
−10	−1	0	535.24	94.42
−28	−1	3	64.18	187.26
−30	−1	4	−325.53	186.34
−30	0	3	−25.09	188.19
−32	0	4	−24.83	183.56

-32	1	3	33.68	178.94
18	2	10	9.90	202.85
-27	12	-3	-8.19	137.87
-33	15	-3	-1.72	199.68
-1	24	-4	-33.41	100.89
8	-7	6	0.00	86.90
8	-7	9	-49.79	145.00
-20	-6	10	-162.30	176.57
-22	-6	8	-10.43	163.10
-27	-4	7	-87.82	193.73
8	1	1	31.96	72.24
-34	1	4	0.13	188.98
19	2	8	3.17	183.43
-34	2	3	-18.49	186.60
20	4	7	74.35	187.92
12	5	1	394.07	130.61
-20	6	-2	245.90	143.42
-30	7	-1	79.50	177.23
-32	8	-1	31.30	203.24
21	10	5	0.00	196.11
20	10	4	59.82	199.68
-35	16	-3	290.40	189.24
14	17	0	-16.51	146.59
-29	17	-4	-103.40	180.13
-31	18	-4	90.99	195.19
4	22	-3	146.98	117.40
14	26	0	0.00	177.62
-12	-8	5	42.66	104.46
12	-4	10	93.10	163.89
-26	-3	4	-169.30	187.39
-26	-2	3	82.80	192.54
-21	-1	1	288.55	133.25
17	1	10	0.00	187.53
16	4	3	325.66	127.17
20	5	6	241.01	207.34
-15	5	-2	455.87	112.52
-28	6	-1	0.00	146.98
10	7	0	1291.82	158.61
-34	9	-1	174.58	217.50
15	11	1	136.02	153.06
-25	11	-3	164.68	136.15
13	13	0	77.65	131.40
6	14	-2	159.66	115.82
-20	14	-4	-79.10	113.84
16	15	1	122.82	157.94
-27	16	-4	54.67	139.85
-33	19	-4	-122.42	195.58
-14	21	-5	108.29	79.37
-30	23	-5	-21.13	185.28
-32	24	-5	14.39	177.89
2	-9	8	-47.28	70.12
0	-9	9	56.39	61.67
6	-8	8	0.00	100.23
6	-7	10	-0.13	124.01

−25	−5	7	48.73	180.26
10	−4	11	185.41	173.66
9	−3	3	−6.21	81.35
9	−3	12	71.05	180.40
−2	−3	1	1129.25	109.74
−28	−3	5	−28.00	178.15
2	−2	1	972.50	84.78
−30	−2	5	−199.02	180.13
−32	−1	5	0.00	189.90
16	0	10	5.68	192.02
−28	0	2	−1.58	193.73
18	1	8	30.64	184.49
−30	1	2	176.96	192.81
9	2	1	777.58	106.57
−32	2	2	191.75	195.98
6	3	0	477.53	81.09
−34	3	2	−194.66	186.21
11	4	1	305.59	114.50
−29	4	0	−259.63	169.43
18	5	4	144.74	155.57
−26	5	−1	−32.75	146.32
−31	5	0	143.68	196.64
−33	6	0	5.15	227.94
16	8	2	0.00	138.80
−9	9	−3	283.01	70.92
−18	9	−3	148.04	130.48
−31	10	−2	0.00	166.93
−36	10	−1	21.00	190.43
10	14	−1	231.50	125.19
−37	17	−3	61.80	186.73
11	18	−1	79.63	128.76
14	20	0	121.63	205.35
−35	20	−4	0.00	182.64
−28	22	−5	191.09	189.51
14	23	0	32.88	194.66
−9	−9	6	58.11	92.18
−9	−9	9	−64.71	97.33
−11	−9	7	84.92	113.18
−16	−8	7	−36.71	138.53
−17	−7	5	6.60	122.02
12	−5	8	72.77	151.61
−24	−4	4	79.76	195.85
−24	−3	3	5.28	153.72
−18	0	0	475.29	151.87
10	3	1	97.99	100.89
−27	3	0	−87.56	146.06
19	5	5	0.00	159.79
−35	7	0	−8.72	199.94
18	9	3	158.08	140.25
−29	9	−2	−20.21	142.36
19	13	3	0.00	213.94
18	19	2	37.90	194.39
−18	20	−5	−70.26	100.76
−21	20	−5	176.17	118.19

8	22	−2	0.00	136.95
8	25	−2	137.48	193.07
11	27	−1	3.43	182.38
−19	−7	6	−163.23	152.13
−19	−7	9	−55.07	157.94
−17	−4	2	245.24	120.57
−26	−4	5	−29.98	173.00
−13	−1	0	131.40	97.46
−26	−1	2	−25.22	152.00
17	0	8	26.02	180.26
19	3	7	123.48	189.64
15	3	3	76.46	131.66
−24	4	−1	41.73	124.27
9	6	0	175.38	100.10
−27	8	−2	190.04	143.15
−23	10	−3	0.00	120.04
17	12	2	119.78	144.87
−11	14	−4	231.11	89.80
−25	15	−4	80.03	131.14
16	18	1	−45.83	207.34
−39	18	−3	−140.12	183.30
16	21	1	−95.08	192.02
−26	21	−5	18.49	165.34
−37	21	−4	−167.32	182.11
4	25	−3	−62.73	159.27
2	−8	10	−5.02	94.16
−20	−7	8	11.36	154.25
−12	−5	2	−60.88	116.74
11	−3	11	111.33	176.04
−14	−3	1	856.02	138.53
10	−2	12	−29.19	190.04
−17	2	−1	302.95	133.78
−25	2	0	−25.75	135.89
7	4	0	858.66	103.40
8	5	0	801.87	107.23
−37	8	0	−50.98	181.06
20	13	4	−69.86	193.73
2	17	−3	10.17	84.78
11	21	−1	0.00	157.15
11	24	−1	113.70	193.34
−13	−8	10	0.00	126.78
10	−6	7	103.01	109.61
8	−6	5	113.57	102.61
−22	−5	4	36.98	165.74
−24	−5	5	−111.99	173.79
13	−4	8	0.00	168.11
−22	−4	3	55.20	136.29
5	−3	2	432.10	66.56
−19	−2	1	−68.67	127.04
−24	−2	2	0.00	134.83
16	−1	8	18.75	181.45
−4	−1	0	767.01	86.24
15	1	11	−54.94	202.85
17	4	4	83.46	147.64

−25	7	−2	215.39	150.15
20	9	5	214.60	201.00
19	9	4	−124.53	177.89
5	13	−2	385.62	116.21
19	16	3	−176.04	196.90
7	18	−2	0.00	115.55
−39	22	−4	22.19	188.98
4	−5	12	−64.31	154.91
14	−3	8	33.28	177.36
−30	−3	6	76.73	198.88
15	−2	8	0.00	186.34
12	−2	11	27.73	180.79
10	−2	3	56.92	89.93
−32	−2	6	23.64	200.20
13	−1	11	38.69	188.32
11	−1	12	−1.32	192.02
14	0	11	63.52	201.26
12	0	12	−121.89	204.43
13	1	12	−30.51	210.77
18	2	7	195.05	201.26
14	2	3	−100.89	135.63
−30	2	1	−141.31	219.75
−32	3	1	0.00	205.35
18	4	5	148.83	139.98
−34	4	1	157.94	183.56
−18	5	−2	459.44	140.64
15	7	2	0.00	122.16
14	10	1	340.59	132.06
12	12	0	−83.07	117.14
−23	14	−4	150.81	130.61
13	16	0	−78.84	140.78
−24	20	−5	35.39	136.15
−15	−7	4	375.19	118.72
9	−6	9	0.00	148.30
5	−6	11	46.49	134.57
−15	−6	3	−48.33	106.71
−28	−4	6	292.25	191.62
−30	−3	9	−0.40	220.41
−23	1	0	29.71	130.21
−28	1	1	102.08	178.55
18	3	9	−34.07	194.00
−22	3	−1	6.47	131.53
−36	5	1	−25.49	184.89
20	7	7	111.59	177.62
20	8	6	100.37	194.53
9	13	−1	−44.64	115.95
10	29	−1	−79.63	180.92
−7	−8	4	316.29	100.10
−18	−7	10	0.00	155.30
−28	−4	9	−153.45	204.96
11	−1	3	855.36	124.14
12	0	3	769.26	136.02
13	1	3	176.43	126.12
−23	6	−2	−23.24	127.57

17	8	3	14.66	134.83
−21	9	−3	41.47	117.40
18	12	3	0.40	161.25
−30	12	−3	0.00	163.10
−15	13	−4	52.30	105.12
−18	13	−4	−36.85	104.20
−32	13	−3	0.00	204.83
17	15	2	0.00	179.21
−15	20	−5	−18.22	85.58
13	25	0	−90.99	181.98
−4	−8	4	66.56	72.50
9	−6	6	94.16	92.84
−22	−6	5	57.97	177.89
−26	−5	6	108.69	170.49
−22	−3	2	230.18	134.44
−26	0	1	−76.20	143.42
17	1	7	0.00	180.92
16	3	4	272.05	132.59
19	5	8	−65.11	173.92
−6	5	−2	1289.05	119.65
20	12	5	−174.85	188.85
−34	14	−3	34.86	191.75
10	17	−1	75.41	124.67
−32	17	−4	−66.16	197.04
17	18	2	126.25	199.94
13	19	0	−39.88	153.98
3	21	−3	161.38	106.97
−31	22	−5	−21.39	174.58
−2	23	−4	5.02	87.82
−33	23	−5	−45.69	174.19
−13	−9	8	−30.64	124.27
0	−6	12	0.00	125.99
11	−5	7	14.00	113.70
−20	−5	3	151.87	121.36
−26	−5	9	−166.79	194.00
−29	−4	8	−5.02	205.35
−31	−3	8	−229.39	235.20
−16	−1	0	37.24	120.97
17	2	9	204.43	180.66
17	3	5	34.34	134.70
14	6	2	52.03	141.83
19	8	5	43.32	198.49
18	8	4	−126.25	150.15
16	11	2	−104.72	135.36
−28	11	−3	210.64	138.66
19	12	4	−46.88	216.32
−36	15	−3	83.73	187.53
−30	16	−4	−36.18	181.45
15	17	1	38.30	161.25
−34	18	−4	−167.19	186.60
−29	21	−5	98.78	189.11
13	22	0	0.00	199.81
15	23	1	98.25	184.36
7	27	−2	155.17	182.64

−12	−9	6	−34.86	106.97
5	−8	7	45.83	78.97
−15	−8	5	79.10	114.50
−17	−8	6	82.54	135.36
7	−6	10	126.12	135.49
−20	−6	4	0.00	135.36
9	−4	4	461.55	104.59
−24	−1	1	−0.53	133.38
16	0	7	132.59	160.32
−9	1	−1	67.48	70.78
−12	1	−1	105.25	87.42
−13	8	−3	325.53	101.69
−16	8	−3	113.97	120.31
13	9	1	−29.45	124.93
4	12	−2	209.58	97.99
−28	15	−4	132.06	148.04
1	16	−3	0.00	84.65
−38	16	−3	−56.13	198.22
−19	19	−5	222.92	106.31
−36	19	−4	−64.71	179.74
15	20	1	84.65	216.71
7	21	−2	20.60	125.06
10	26	−1	71.97	185.41
−10	−8	4	0.00	91.39
−17	−8	9	108.55	144.74
−18	−8	8	67.75	145.66
−24	−6	6	51.77	165.21
10	−5	9	−117.14	157.28
−15	−5	2	−24.04	120.70
−27	−5	8	114.36	182.77
−17	−3	1	0.00	125.33
16	1	9	201.26	171.68
15	2	4	−9.90	118.72
−20	2	−1	0.00	131.93
−29	5	−1	−9.24	146.46
19	6	7	203.51	179.87
−31	6	−1	−54.41	186.60
19	7	6	27.34	199.02
−33	7	−1	−195.19	201.66
−35	8	−1	−210.64	202.58
−26	10	−3	82.93	130.34
−21	13	−4	71.05	115.03
18	15	3	0.13	194.26
−22	19	−5	124.40	116.48
−27	20	−5	56.52	172.87
−38	20	−4	70.52	178.41
7	24	−2	−0.40	167.98
4	−8	6	55.60	61.01
1	−7	11	−6.60	105.78
−10	−7	3	272.71	108.55
9	−5	5	237.58	107.76
12	−4	7	115.95	117.93
−31	−2	4	−231.90	179.87
15	−1	7	−149.76	139.32

−31	−1	3	−9.11	183.96
−33	−1	4	−155.04	191.22
−33	0	3	58.11	174.32
16	2	5	112.38	132.33
18	4	8	37.37	176.83
−13	4	−2	116.35	94.29
−21	5	−2	185.55	131.00
16	7	3	280.10	135.10
−32	9	−2	71.05	186.34
−37	9	−1	−59.96	194.92
−34	10	−2	−4.75	196.77
−26	14	−4	17.70	137.87
19	15	4	1.72	188.32
18	18	3	−91.39	184.49
10	20	−1	56.92	134.97
10	23	−1	−91.91	185.55
−7	−9	5	62.33	77.39
−12	−9	9	−91.78	115.16
−14	−9	7	84.65	125.19
7	−7	8	60.75	106.44
−24	−6	9	−152.13	181.58
13	−3	7	−22.98	120.18
−29	−3	4	−109.48	168.25
14	−2	7	0.00	125.19
−29	−2	3	113.84	189.38
15	0	9	0.00	168.64
16	1	6	0.79	122.55
14	1	4	−19.94	126.38
−15	1	−1	284.20	117.40
−30	3	0	37.51	190.04
−27	4	−1	0.00	141.57
−32	4	0	271.78	196.24
13	5	2	291.72	134.04
−34	5	0	0.00	182.38
−36	6	0	0.00	181.32
−19	8	−3	150.95	125.85
−30	8	−2	36.71	156.76
−36	11	−2	0.00	173.66
8	12	−1	226.09	124.80
12	15	0	0.00	124.01
6	17	−2	90.46	101.55
3	24	−3	−113.70	129.68
12	27	0	0.00	174.72
−20	−7	5	−7.53	139.19
−25	−6	8	−2.64	173.13
10	−5	6	91.39	97.59
11	−4	9	−1.85	154.25
5	−4	12	−55.07	158.08
−9	−4	1	236.13	79.37
10	−3	4	1434.97	163.62
−27	−3	3	161.91	191.75
−32	−3	7	26.02	206.15
−28	2	0	140.51	147.78
16	3	10	−197.56	189.38

−10	4	−2	921.26	109.08
−16	4	−2	348.64	124.14
18	7	5	−5.55	158.34
−28	7	−2	165.60	142.36
−24	9	−3	−24.70	128.89
17	11	3	40.54	150.02
3	11	−2	504.08	89.27
16	14	2	36.98	141.57
−25	19	−5	−116.48	144.74
6	−5	11	252.10	144.47
4	−5	3	435.14	57.71
−27	−4	4	−66.82	174.06
−30	−4	7	225.69	204.03
−31	−3	5	56.39	180.66
−22	−2	1	325.53	139.32
−33	−2	5	117.67	192.02
14	−1	9	10.70	161.38
15	0	6	63.39	130.08
13	0	4	−1.32	125.33
15	1	5	104.72	128.50
17	7	4	−5.15	140.12
15	10	2	112.52	136.15
19	11	5	−36.05	190.56
−12	13	−4	92.84	91.39
−16	19	−5	287.76	94.16
14	25	1	−41.60	176.83
−18	−7	4	0.00	114.89
−22	−7	6	−275.48	159.66
−18	−6	3	220.67	122.42
8	−5	10	31.43	138.14
−28	−5	7	−151.21	192.15
−12	−4	1	573.28	115.55
12	−3	9	−57.97	161.25
13	−2	9	−0.92	166.66
11	−2	4	561.92	122.95
12	−1	4	164.28	126.91
−19	−1	0	212.75	124.01
17	3	8	−188.05	174.58
−25	3	−1	−92.57	144.61
18	5	7	−46.88	192.41
18	6	6	−26.81	183.56
−26	6	−2	244.31	136.02
12	8	1	262.80	134.04
−5	10	−3	540.92	56.52
18	11	4	130.21	170.36
−24	13	−4	−48.99	120.70
9	16	−1	−142.10	112.52
16	20	2	−13.87	191.22
12	24	0	−149.63	184.36
−4	−9	5	37.90	60.75
−10	−9	5	114.10	95.22
11	−4	6	377.17	113.97
10	−4	5	65.63	117.01
−25	−4	3	32.75	175.24

−29	−4	5	0.00	178.55
6	−2	2	158.08	62.60
−11	−2	0	115.03	85.71
14	−1	6	21.13	120.97
14	0	5	293.44	119.25
−26	1	0	−30.77	133.25
15	2	10	−62.07	190.17
12	4	2	260.82	124.67
15	6	3	124.01	133.65
−10	8	−3	267.29	72.50
19	10	6	−204.83	187.79
14	16	1	−37.51	143.95
12	18	0	−106.31	132.33
−32	21	−5	214.07	175.77
−34	22	−5	19.81	172.87
−36	23	−5	−37.37	175.77
9	28	−1	−186.21	173.66
1	−9	7	86.37	38.83
−13	−8	4	0.00	101.55
−13	−7	3	72.24	97.86
−22	−7	9	11.36	166.13
−25	−5	4	−33.15	175.51
−6	−4	1	1707.42	156.36
12	−3	6	93.50	113.70
13	−2	6	31.56	115.69
−18	1	−1	216.45	132.06
−33	2	1	−73.16	176.83
−35	3	1	−20.21	174.45
2	10	−2	414.01	71.18
−31	11	−3	29.58	180.53
−19	12	−4	187.79	106.97
−33	12	−3	125.59	197.43
−35	13	−3	−29.71	196.11
17	14	3	57.58	185.41
0	15	−3	171.94	84.39
−33	16	−4	90.07	188.72
−35	17	−4	−44.11	185.68
−20	18	−5	−55.07	103.80
2	20	−3	20.87	96.14
−30	20	−5	23.64	183.04
12	21	0	25.36	172.21
14	22	1	126.12	186.34
11	29	0	237.45	194.26
−16	−8	10	−40.28	140.91
−23	−7	8	57.97	161.64
−26	−6	7	−15.32	179.34
−27	−5	5	0.00	165.21
6	−3	12	−191.62	169.96
−8	−2	0	−55.60	68.01
−14	−2	0	112.91	111.59
14	1	10	−127.17	183.83
−31	1	1	−65.24	192.68
16	2	8	−0.92	177.62
14	3	11	−47.67	203.90

−19	4	−2	48.99	137.34
17	5	9	0.00	187.26
−22	8	−3	37.51	123.48
7	11	−1	527.85	111.59
−16	12	−4	258.84	103.40
18	14	4	−95.61	212.49
−37	14	−3	248.67	198.36
−31	15	−4	−147.91	181.19
−23	18	−5	65.11	119.65
−37	18	−4	−114.50	174.58
14	19	1	46.88	196.11
6	20	−2	63.26	112.12
−15	−9	6	120.84	126.51
−10	−6	2	194.53	100.50
−18	−5	2	−15.85	113.57
9	−4	10	−27.20	148.04
7	−4	11	−51.50	149.89
−24	0	0	38.17	144.74
−29	0	1	59.96	179.21
−23	2	−1	102.48	123.87
17	4	7	22.45	192.15
−24	5	−2	13.07	142.49
17	6	5	76.73	135.23
16	6	4	208.66	137.48
18	7	8	−12.41	188.32
0	8	−2	625.31	63.52
16	10	3	134.57	127.84
−29	10	−3	5.55	141.83
13	12	1	−24.83	120.97
−29	14	−4	−98.91	157.68
17	17	3	0.00	186.47
−28	19	−5	−3.30	175.24
9	25	−1	−149.89	192.28
6	26	−2	51.50	181.58
−18	−8	5	−16.77	131.93
8	−6	8	−1.19	110.54
−7	−6	2	115.03	71.31
−23	−5	3	62.99	146.06
−15	−4	1	127.17	126.51
13	0	10	66.16	171.02
−6	1	−1	934.60	104.99
11	3	2	527.32	111.86
17	5	6	69.99	161.77
11	7	1	456.93	139.46
14	9	2	56.65	126.65
1	9	−2	125.06	48.99
18	10	5	−60.09	197.04
−22	12	−4	371.49	121.63
15	13	2	−11.89	134.44
11	14	0	62.46	121.89
5	16	−2	52.03	95.48
9	19	−1	25.09	117.80
−3	22	−4	72.77	73.29
6	23	−2	−58.90	126.51

−16	−9	8	99.05	130.87
−20	−8	6	125.85	147.64
−24	−7	7	−125.99	164.68
−23	−6	4	72.77	173.40
−25	−6	5	−88.74	164.15
7	−2	12	−21.00	171.42
−27	−1	1	30.77	166.00
15	1	8	100.37	175.51
13	2	11	−79.50	190.43
14	5	3	234.80	127.17
−34	6	−1	−51.77	181.58
−17	7	−3	286.44	144.87
−27	9	−3	105.12	141.04
17	10	4	−6.74	158.47
−27	13	−4	−106.04	141.17
9	22	−1	−61.67	141.83
8	30	−1	−85.18	174.72
−13	−9	5	66.95	101.55
5	−7	9	107.63	125.19
7	−1	2	183.56	65.77
11	2	12	−203.11	209.45
10	2	2	470.14	98.78
16	4	9	67.62	171.42
−32	5	−1	69.07	203.24
−14	7	−3	1389.81	157.42
−36	7	−1	317.34	187.00
18	8	7	3.43	186.21
18	9	6	−83.07	186.87
−17	18	−5	146.59	94.95
−26	18	−5	20.73	145.53
11	26	0	343.36	180.40
3	−7	10	162.17	109.87
−13	−6	2	268.35	120.84
8	−3	11	36.18	161.25
−17	−2	0	262.01	128.10
8	−1	12	88.22	178.28
9	0	12	−16.51	181.85
12	1	11	130.34	191.75
10	1	12	−3.83	188.98
16	3	7	−6.74	180.92
−30	4	−1	26.81	174.72
−31	7	−2	0.53	180.92
−33	8	−2	55.60	197.17
9	9	0	47.28	106.71
−35	9	−2	214.34	179.07
−37	10	−2	−179.34	187.66
8	15	−1	92.84	104.33
15	16	2	66.03	150.02
15	22	2	13.87	181.45
2	23	−3	139.46	106.57
−9	−10	7	0.00	93.10
−15	−9	9	0.00	124.14
−21	−8	8	199.94	156.62
6	−7	7	0.00	85.97

−16	−7	3	200.20	112.12
1	−5	12	0.00	135.36
9	−2	11	−161.38	168.64
−25	−2	1	173.00	141.44
10	−1	11	−59.82	167.32
−22	−1	0	63.92	115.42
14	0	8	303.48	182.24
11	0	11	206.94	179.21
8	0	2	513.45	81.22
9	1	2	97.86	78.18
−33	3	0	0.00	176.57
−22	4	−2	0.00	117.01
−35	4	0	−252.63	186.87
16	5	5	6.07	129.68
15	5	4	2.51	126.51
17	6	8	124.14	165.60
−20	7	−3	160.19	117.93
−25	8	−3	5.02	126.78
−13	12	−4	172.08	98.25
18	13	5	−42.79	185.15
16	13	3	0.00	142.89
11	17	0	11.23	129.95
15	19	2	−94.95	206.15
−33	20	−5	344.28	175.38
−35	21	−5	−72.50	183.04
13	24	1	−87.69	182.38
2	26	−3	53.22	148.57
−6	−10	7	5.94	64.97
−9	−8	11	−14.00	37.64
−16	−8	4	18.62	110.14
−20	−8	9	32.75	152.40
−23	−7	5	195.45	164.81
−21	−6	3	−9.51	132.33
−27	−6	6	−41.20	173.79
9	−5	8	57.18	118.59
−29	−5	9	0.00	206.81
−32	−2	3	−68.94	168.25
−34	−1	3	−78.44	176.57
−13	0	−1	363.17	98.65
−31	2	0	67.22	193.07
−14	3	−2	534.72	109.74
−28	3	−1	−26.81	133.91
10	6	1	72.63	111.20
−29	6	−2	76.86	152.00
6	10	−1	134.83	89.67
−25	12	−4	91.78	123.61
17	13	4	0.00	192.81
−31	19	−5	−110.27	179.60
−37	22	−5	−9.38	178.15
11	23	0	−64.45	192.15
−21	−7	4	0.00	154.78
−30	−5	8	3.30	204.56
−18	−4	1	−101.69	111.59
−30	−3	3	266.24	177.09

−32	−3	4	10.30	180.53
13	−1	8	−5.94	170.62
−34	0	2	119.78	177.75
−29	1	0	188.98	171.81
15	3	9	−74.88	161.38
−17	3	−2	493.78	129.42
13	4	3	120.57	136.02
13	8	2	−22.98	121.23
17	9	5	10.83	170.09
15	9	3	−50.84	125.46
12	11	1	204.30	121.23
18	12	6	0.00	175.64
−1	14	−3	151.87	64.58
−21	17	−5	22.45	107.37
13	18	1	−90.99	143.68
11	20	0	−33.68	134.04
13	21	1	0.00	201.26
−22	−8	7	48.07	150.55
10	−4	8	31.03	125.33
−30	−4	4	−20.87	169.30
12	−2	8	−8.58	160.45
−32	−1	2	−47.54	171.94
−10	0	−1	188.98	74.09
−16	0	−1	410.71	127.97
15	2	7	35.00	159.27
−7	4	−2	2008.38	177.23
15	5	10	−19.55	182.64
−27	5	−2	75.54	131.66
16	9	4	58.37	130.74
−20	11	−4	101.03	107.50
−34	11	−3	−42.52	180.00
14	12	2	217.77	133.12
−36	12	−3	181.06	172.74
10	13	0	23.90	104.06
−32	14	−4	60.22	197.56
−34	15	−4	60.22	186.21
17	16	4	86.90	185.55
16	16	3	303.74	195.58
−36	16	−4	−178.55	168.77
−24	17	−5	89.93	121.50
−29	18	−5	35.00	175.24
16	19	3	91.39	179.07
8	27	−1	110.14	174.45
5	28	−2	14.39	176.83
4	−7	5	926.67	104.20
−25	−7	6	20.21	165.47
2	−6	11	81.09	118.85
−16	−6	2	65.63	104.72
−27	−6	9	197.70	200.86
−28	−6	8	90.59	188.85
−28	−4	3	13.87	171.94
11	−3	8	94.03	139.72
−23	−3	1	156.89	137.74
−30	−2	2	−86.50	186.21

-26	2	-1	59.03	126.78
17	7	7	-110.14	174.85
17	8	6	-96.93	193.34
-32	10	-3	0.00	200.34
-17	11	-4	138.66	101.16
-38	17	-4	-128.76	180.13
1	19	-3	37.11	85.84
10	28	0	19.02	161.51
-12	-10	7	16.77	108.03
-28	-5	4	-153.45	164.68
-27	0	0	-45.43	147.51
-11	3	-2	543.56	90.73
15	4	5	-30.64	126.38
14	4	4	112.12	117.80
16	5	8	172.74	165.60
-11	7	-3	1517.12	151.21
8	8	0	597.31	109.08
-30	9	-3	-258.58	171.15
-30	13	-4	-197.17	174.45
4	15	-2	194.13	105.38
-18	17	-5	132.99	98.12
8	18	-1	-151.08	119.65
5	19	-2	6.74	104.59
-7	-10	6	157.68	68.41
-10	-10	6	103.93	87.03
-11	-10	8	-0.13	95.61
-8	-9	10	36.85	48.20
-16	-9	5	-12.68	111.86
-18	-9	6	51.11	132.59
3	-8	8	41.20	78.84
-31	-5	7	-21.00	197.83
-32	-4	5	-153.85	188.98
-28	-3	2	63.65	184.89
14	1	7	21.39	137.48
-34	1	1	-74.61	190.43
14	2	9	113.57	160.32
12	3	3	244.58	119.65
-20	3	-2	136.55	114.50
9	5	1	35.39	88.35
-23	11	-4	200.47	125.99
-27	17	-5	0.00	153.85
8	24	-1	127.04	165.08
-3	25	-4	-5.55	88.61
12	26	1	47.15	167.32
-11	-9	10	-3.57	80.03
-21	-8	5	-40.67	153.85
5	-7	6	83.07	66.43
6	-6	9	-26.28	133.78
-26	-5	3	191.49	178.81
-30	-5	5	18.49	177.75
-19	0	-1	374.26	130.74
-32	0	1	-77.12	177.49
-24	1	-1	0.00	127.97
14	4	10	193.87	183.56

−25	4	−2	60.09	130.61
17	12	5	−1.06	194.39
−28	12	−4	−68.01	146.46
14	15	2	98.91	130.08
8	21	−1	−34.07	120.84
5	22	−2	−14.39	114.50
5	25	−2	65.90	142.63
−1	−8	10	−38.30	60.88
−19	−8	10	−16.24	152.93
−25	−7	9	−35.00	170.89
−26	−7	8	−43.98	176.83
7	−6	7	−41.47	91.12
−26	−6	4	−74.35	157.94
−29	−6	7	10.83	184.89
5	−4	3	254.48	54.67
−5	−2	0	−17.96	70.52
−25	−1	0	30.51	123.34
−30	−1	1	−3.04	201.26
16	8	5	83.07	138.53
14	8	3	10.30	124.01
−28	8	−3	230.58	140.51
16	12	4	144.87	149.23
15	12	3	−24.43	125.06
7	14	−1	678.00	129.16
−36	20	−5	0.00	166.66
14	21	2	35.39	185.41
10	25	0	−58.77	179.87
−19	−9	8	111.20	141.44
−11	−8	3	0.00	97.33
−19	−8	4	0.00	126.78
−23	−8	6	0.00	144.87
−4	−6	2	1131.63	109.48
−28	−6	5	24.30	170.49
−10	−5	1	282.87	88.35
−13	−5	1	−82.14	112.78
−26	−4	2	−150.55	182.24
−12	−3	0	433.29	105.91
3	−1	1	440.95	54.28
13	0	7	−3.30	125.33
13	1	9	−78.44	157.28
14	3	5	246.43	132.72
−33	4	−1	185.41	176.04
−35	5	−1	0.00	191.88
16	6	7	−77.52	185.02
12	7	2	179.60	128.10
−36	8	−2	−26.02	167.72
−6	9	−3	139.98	44.37
11	10	1	318.93	135.10
17	11	6	−87.95	181.85
17	15	5	−124.27	179.07
10	16	0	−14.92	122.29
−22	16	−5	−57.97	111.46
−32	18	−5	−12.41	164.94
−34	19	−5	−19.55	163.49

−12	20	−5	124.67	26.41
−38	21	−5	71.58	180.00
7	29	−1	190.96	170.89
9	30	0	−73.29	171.15
−13	−10	6	0.00	105.12
−8	−9	4	4.75	80.56
−11	−9	4	11.09	90.07
−20	−9	7	−33.15	140.51
4	−6	10	9.24	115.95
2	−4	12	35.00	137.08
−21	−4	1	466.97	137.87
−15	−3	0	120.44	119.78
−28	−2	1	17.43	183.96
14	2	6	−2.91	118.85
11	2	3	0.13	111.72
13	3	4	90.99	117.40
13	3	10	−197.43	175.51
15	4	8	−139.59	178.81
8	4	1	225.30	77.78
−15	6	−3	201.13	107.63
−32	6	−2	72.50	183.43
16	7	6	−23.77	178.28
−34	7	−2	129.82	180.92
15	8	4	90.07	127.57
−14	11	−4	425.76	108.42
−13	19	−5	10.96	68.94
12	23	1	0.00	200.86
−8	−8	3	448.88	98.39
−27	−7	7	−77.26	173.79
−24	−6	3	−38.43	181.32
12	0	9	−11.09	155.30
−31	3	−1	0.00	186.47
7	7	0	1651.29	162.17
13	11	2	0.00	127.57
−26	11	−4	17.04	120.04
16	15	4	−160.72	200.86
15	15	3	95.48	146.46
−25	16	−5	−59.30	129.16
12	17	1	45.43	126.91
−30	17	−5	0.13	176.57
16	18	4	71.71	182.51
15	18	3	175.11	188.98
10	22	0	230.45	156.89
11	28	1	−8.58	164.55
−14	−10	8	67.22	114.76
−18	−9	9	35.00	139.32
−14	−8	3	151.87	100.23
7	−5	9	1.98	144.87
−16	−5	1	−38.30	114.10
−32	−5	6	89.41	185.68
12	−1	7	85.58	115.03
−34	2	0	35.00	166.26
−23	3	−2	33.02	120.04
12	4	11	58.64	186.87

−30	5	−2	0.00	167.85
15	6	9	−35.92	165.08
16	8	8	6.07	167.98
−35	14	−4	64.45	167.45
−37	15	−4	113.57	167.06
−19	16	−5	140.78	102.08
10	19	0	−34.86	116.61
12	20	1	−204.83	159.93
−4	21	−4	76.07	67.09
1	22	−3	81.35	98.65
−14	−9	4	90.20	101.69
−24	−8	8	−59.03	158.61
−24	−7	4	76.33	153.06
−26	−7	5	121.36	152.53
8	−5	7	58.64	96.54
3	−5	11	4.36	123.74
−24	−5	2	4.09	146.19
−9	−3	0	641.42	94.56
−23	−2	0	169.57	138.00
11	−1	9	169.70	154.38
−22	0	−1	121.23	122.29
13	1	6	−40.54	113.44
10	1	3	575.79	111.59
−32	1	0	−34.20	178.28
−29	2	−1	−126.51	162.30
−18	10	−4	38.30	99.57
−21	10	−4	−31.96	111.20
−35	10	−3	−0.26	168.51
−37	11	−3	−8.58	160.19
−2	13	−3	23.24	47.15
−33	13	−4	97.59	187.26
−14	18	−5	204.83	81.61
1	25	−3	−28.13	111.72
8	32	0	−138.93	177.36
−14	−9	10	−59.96	115.69
−2	−7	11	152.53	55.73
−30	−6	6	112.65	184.49
8	−4	9	157.94	145.93
−18	−3	0	55.07	113.57
−26	−3	1	146.32	150.95
11	−2	7	0.00	110.27
13	2	5	52.16	114.23
12	2	4	136.42	134.97
12	2	10	0.00	163.36
−15	2	−2	541.85	119.12
14	3	8	24.04	170.62
7	3	1	1224.87	126.12
15	5	7	91.39	177.75
−33	9	−3	−24.83	183.17
16	11	5	0.00	171.81
−31	12	−4	35.26	181.45
−28	16	−5	−0.26	162.83
7	26	−1	36.85	173.53
6	31	−1	−98.65	163.36

−19	−9	5	42.66	130.61
−23	−8	9	−21.13	159.13
−11	−7	2	326.19	112.65
9	−4	7	0.00	96.93
10	−3	7	203.11	110.67
9	−3	9	−94.42	151.08
−33	−3	3	69.99	181.45
10	−2	9	−97.33	152.27
−14	−1	−1	167.32	101.82
−7	0	−1	812.97	97.86
−30	0	0	−0.26	184.09
−18	2	−2	153.32	124.40
11	3	11	−61.67	185.41
−28	4	−2	−35.66	136.29
11	6	2	162.04	116.35
15	7	5	−31.43	128.89
13	7	3	5.81	112.38
14	11	3	49.52	128.23
7	17	−1	90.20	103.01
13	23	2	−45.16	175.38
9	27	0	47.81	172.87
4	27	−2	68.54	168.77
−5	−10	8	−2.25	35.00
−21	−9	6	9.24	138.00
−25	−8	7	−57.05	154.64
−14	−7	2	304.66	98.12
5	−5	10	109.08	120.31
−7	−5	1	755.92	93.50
−31	−4	3	−28.13	164.68
6	−3	3	645.25	80.95
3	−3	12	53.22	148.96
−33	−2	2	0.13	163.49
−17	−1	−1	182.64	121.63
9	0	3	605.10	97.86
11	1	10	0.00	153.72
−27	1	−1	−96.27	133.78
9	3	12	−48.47	187.66
15	6	6	72.63	136.42
−12	6	−3	230.18	88.74
14	7	4	117.01	121.63
16	10	6	6.21	179.21
−24	10	−4	5.15	112.25
15	11	4	57.97	133.65
16	14	5	−60.75	185.55
13	14	2	0.00	120.57
3	14	−2	619.76	107.50
−8	14	−4	0.00	51.90
−15	17	−5	−52.96	82.54
4	18	−2	24.70	89.93
−37	19	−5	−119.25	170.09
−11	21	−5	−36.58	30.51
11	25	1	209.84	183.96
−11	−10	5	339.66	100.89
−16	−10	6	32.22	121.36

−17	−8	3	90.99	109.74
−22	−7	3	165.87	145.14
−28	−7	6	16.51	182.11
6	−6	6	194.26	75.01
−19	−5	1	−27.73	117.93
−3	−4	1	2839.05	239.69
−31	−3	2	64.58	171.68
−28	−1	0	88.61	165.74
12	0	6	−27.73	109.35
6	2	1	1313.21	129.02
14	5	9	85.18	157.42
6	6	0	388.13	74.88
10	9	1	414.01	121.23
−29	11	−4	−120.97	155.04
11	13	1	142.49	115.55
−23	15	−5	15.72	114.76
0	18	−3	94.95	75.54
−35	18	−5	24.04	171.68
13	20	2	11.49	203.37
7	23	−1	97.99	131.00
−8	−10	5	93.63	79.10
−22	−8	4	0.00	161.91
−24	−8	5	−84.39	151.08
−29	−5	3	−110.67	160.98
−31	−5	4	0.00	175.51
−24	−4	1	78.58	133.91
7	−2	3	379.41	71.84
8	−1	3	194.00	74.22
−11	−1	−1	36.18	74.61
4	0	1	3232.33	274.29
12	1	5	93.37	103.27
11	1	4	134.57	116.21
13	2	8	13.47	171.94
10	2	11	−55.86	173.92
8	2	12	0.00	171.94
−12	2	−2	353.53	88.61
−21	2	−2	61.01	116.35
−8	3	−2	811.12	90.86
−26	3	−2	448.35	141.57
15	7	8	5.55	161.91
6	13	−1	−55.47	108.69
−33	17	−5	−2.91	155.30
7	20	−1	43.32	116.87
−17	−9	4	15.45	108.16
−8	−7	2	123.08	83.07
−22	−6	2	130.48	122.02
−31	−6	8	−35.00	207.20
4	−4	11	0.00	129.02
−29	−4	2	220.15	164.68
−21	−3	0	27.20	120.04
4	−2	12	−51.90	149.63
−33	−1	1	0.00	157.15
10	0	10	−29.45	147.25
5	1	1	829.08	99.05

14	4	7	42.13	147.51
-7	8	-3	115.95	51.64
12	10	2	144.74	109.87
-15	10	-4	4.23	102.61
16	13	6	0.00	186.87
-9	13	-4	155.04	68.41
15	14	4	29.85	165.60
14	14	3	5.02	134.44
9	15	0	0.00	103.67
-20	15	-5	117.53	103.54
-26	15	-5	-15.32	132.46
-31	16	-5	-75.14	172.60
14	20	3	88.61	197.43
4	21	-2	83.33	103.54
9	24	0	99.71	174.72
4	24	-2	80.69	119.52
-10	-10	9	-0.13	83.20
-14	-10	5	54.67	99.44
-18	-10	7	5.94	125.59
-5	-9	4	213.54	78.58
-22	-9	8	30.11	144.34
4	-7	8	163.49	87.95
5	-6	5	352.34	82.41
-29	-6	4	9.51	154.91
6	-4	10	-29.98	124.14
11	-1	6	14.92	104.59
5	-1	12	-46.75	157.02
6	0	12	92.57	163.36
-25	0	-1	98.91	123.34
9	1	11	176.43	166.00
7	1	12	330.15	174.45
13	6	10	-10.70	172.34
-35	6	-2	39.75	168.25
-27	10	-4	66.29	124.80
15	17	4	90.59	181.85
14	17	3	-0.40	187.53
11	22	1	-35.79	181.58
6	28	-1	-84.78	163.62
8	29	0	-70.92	162.17
-13	-10	9	20.47	105.78
-17	-10	8	0.00	118.85
7	-3	10	34.07	134.17
-26	-2	0	0.00	145.66
-31	-2	1	27.73	159.66
11	0	5	398.56	107.89
-34	3	-1	0.13	158.87
13	4	9	5.68	152.13
10	5	2	219.49	109.61
-33	5	-2	-5.68	178.94
14	6	5	1.19	122.82
3	7	-1	1144.57	109.48
15	10	5	5.41	138.14
-36	13	-4	31.17	167.32
11	16	1	-57.05	119.25

−16	16	−5	252.24	87.29
11	19	1	41.34	125.85
12	25	2	−23.24	164.28
−23	−9	7	200.60	147.91
−29	−7	8	−68.14	180.92
−27	−6	3	112.52	163.23
−31	−6	5	192.28	178.02
7	−5	6	70.26	79.37
5	−3	11	0.00	133.51
10	−2	6	4.49	96.27
10	0	4	2144.01	213.54
8	0	11	1.98	150.95
12	1	8	155.96	162.30
−32	2	−1	−84.52	179.74
14	5	6	0.00	108.95
15	8	7	−23.24	169.57
−19	9	−4	111.72	97.99
−22	9	−4	125.99	115.16
−34	12	−4	32.09	175.11
−29	15	−5	35.00	171.15
9	18	0	194.13	117.14
9	21	0	7.79	125.46
10	27	1	−22.19	161.91
3	29	−2	146.46	172.60
−4	−10	6	105.12	53.09
−17	−9	10	53.75	130.08
−21	−9	9	0.00	145.40
−27	−7	4	−127.31	152.00
−27	−5	2	0.00	179.34
9	−3	6	0.00	89.14
−29	−3	1	102.88	185.15
6	−2	11	96.14	149.23
7	−1	11	55.99	150.15
−24	2	−2	178.55	133.25
−31	4	−2	−2.25	183.17
5	5	0	−18.62	62.33
13	6	4	54.28	111.33
15	9	6	31.03	167.59
−36	9	−3	−13.87	156.89
15	10	8	−31.03	162.96
14	10	4	103.14	123.74
13	10	3	−10.30	118.19
−32	11	−4	113.84	187.66
−10	12	−4	34.34	71.84
−22	−9	5	27.86	146.32
−20	−8	3	0.00	124.40
−30	−7	7	164.42	185.55
−22	−5	1	95.88	117.53
8	−4	6	71.58	83.07
−13	−4	0	439.10	118.46
−16	−4	0	45.03	122.16
−30	1	−1	121.89	184.36
13	3	7	147.51	139.59
14	6	8	102.35	155.83

14	8	9	20.73	160.85
9	8	1	−69.99	102.61
15	13	5	−287.89	192.15
12	13	2	9.77	117.14
12	22	2	21.92	185.81
−4	24	−4	33.28	74.48
0	27	−3	82.14	136.02
7	31	0	4.09	152.27
−17	−10	5	10.56	118.06
−19	−10	6	93.76	125.19
−29	−7	5	112.91	171.15
−14	−6	1	129.16	118.59
−2	−5	12	−92.84	117.53
9	−1	4	1003.27	130.87
−23	−1	−1	−3.57	120.97
11	0	8	246.29	149.89
−33	0	0	0.00	173.13
−29	3	−2	271.78	167.19
12	5	10	−18.09	168.91
−25	9	−4	1.85	115.29
15	16	5	47.67	183.17
−36	17	−5	−122.29	155.17
−38	18	−5	−79.90	187.66
−20	−9	4	21.79	155.04
−27	−8	8	2.51	165.87
−20	−7	2	23.90	118.06
−25	−7	3	−79.37	163.10
−11	−6	1	445.57	107.10
6	−5	5	343.23	87.16
6	−4	4	191.62	69.99
−27	−4	1	43.18	174.72
−24	−3	0	−13.73	117.80
−16	1	−2	344.02	120.44
−19	1	−2	4.23	108.55
12	3	9	−162.70	149.63
−30	10	−4	0.00	166.66
15	12	6	52.69	174.72
−21	14	−5	204.83	105.91
−27	14	−5	−27.47	143.02
−17	15	−5	−16.11	91.39
6	16	−1	54.15	99.31
−34	16	−5	52.30	163.36
0	21	−3	173.53	87.03
6	25	−1	−23.77	146.32
8	26	0	89.41	175.91
5	30	−1	61.14	154.12
−16	−10	9	164.42	121.36
−17	−6	1	−91.78	110.67
−25	−6	2	116.21	168.38
−6	−3	0	410.05	71.18
8	−2	4	282.35	92.31
−31	−1	0	−16.77	161.91
−28	0	−1	37.77	157.42
9	4	2	−39.62	82.67

13	5	5	58.24	121.50
−16	9	−4	112.25	102.35
14	13	4	39.22	132.46
13	13	3	0.00	117.53
2	13	−2	308.36	80.03
−32	15	−5	−8.32	165.74
12	16	2	0.13	121.50
14	19	4	167.06	170.89
10	24	1	−66.69	179.34
0	24	−3	116.08	97.20
9	29	1	142.76	156.10
−12	−9	3	98.78	89.93
−25	−8	4	−49.65	149.76
−27	−8	5	86.90	157.28
−28	−8	7	50.71	168.64
5	−6	8	77.39	91.91
7	−3	4	1157.65	125.59
−15	−2	−1	442.01	122.16
10	−1	8	35.66	131.00
11	5	3	144.74	124.40
2	6	−1	1161.08	108.42
14	7	7	208.13	176.57
−8	7	−3	176.83	57.97
14	9	5	33.41	134.97
−11	11	−4	453.50	87.16
5	12	−1	268.61	92.18
15	15	6	31.96	164.94
14	16	4	23.90	180.00
3	17	−2	127.70	89.27
13	19	3	−178.68	181.06
3	26	−2	71.18	131.40
6	33	0	−0.13	163.49
−15	−9	3	21.92	99.44
−31	−7	6	198.09	177.09
−32	−5	3	−59.03	179.21
7	−4	5	689.09	110.67
−10	−4	0	1251.15	133.25
8	−3	5	103.80	98.65
−18	−2	−1	30.51	113.84
−29	−2	0	59.43	169.17
−22	1	−2	−59.82	120.84
12	2	7	−21.26	115.42
−27	2	−2	−122.68	136.68
11	4	10	2.25	155.44
4	4	0	399.75	71.58
13	5	8	−89.27	160.98
12	5	4	−9.77	107.76
13	7	9	6.60	147.38
14	8	6	0.00	140.12
−28	9	−4	84.92	143.68
8	14	0	160.45	108.29
13	16	3	2.77	142.10
6	19	−1	0.26	104.33
6	22	−1	−7.66	109.08

−13	−11	7	47.28	99.97
−12	−10	4	361.85	100.76
−20	−10	8	−5.15	131.40
−21	−10	7	−111.46	132.33
2	−8	7	257.12	65.11
−32	−4	2	37.24	154.78
9	−2	8	−65.77	119.38
−8	−1	−1	94.16	63.92
−13	1	−2	31.56	90.07
11	2	9	77.92	149.36
−9	2	−2	807.42	94.56
−20	4	−3	42.52	92.05
10	5	11	−28.26	173.66
14	9	8	−58.64	163.23
7	10	0	1108.65	136.02
−30	14	−5	−143.55	172.74
10	21	1	−41.86	143.95
8	23	0	0.00	131.40
−10	−11	7	−14.92	88.88
−11	−11	6	−10.96	87.29
−15	−10	4	7.79	93.76
0	−7	10	40.01	89.93
−20	−6	1	184.23	117.40
6	−5	8	62.33	96.01
−25	−5	1	−38.83	157.94
−26	−1	−1	0.13	135.36
5	4	13	−0.13	187.79
8	5	12	−52.96	187.79
−28	5	−3	77.26	139.46
−20	8	−4	−54.81	103.01
−23	8	−4	31.96	113.57
13	9	4	149.76	114.10
12	9	3	46.88	107.50
−35	11	−4	38.56	150.68
−18	14	−5	255.54	100.37
11	24	2	−48.33	178.02
4	32	−1	34.47	132.19
−14	−11	6	79.90	106.84
−5	−10	5	6.60	54.28
−20	−10	5	161.91	131.00
−1	−9	8	0.00	45.83
−9	−9	3	−43.71	96.80
−25	−9	8	−35.00	147.12
−23	−8	3	−101.03	159.53
−1	−6	11	81.75	97.20
−30	−6	3	20.34	150.55
−32	−6	4	176.57	166.00
−30	−5	2	−105.91	151.61
7	−4	8	113.84	103.93
−22	−4	0	179.74	114.89
8	−3	8	83.59	111.99
−12	−2	−1	647.89	102.35
−21	−2	−1	−30.51	115.95
−23	4	−3	−4.36	112.65

−34	4	−2	−25.49	149.36
−33	10	−4	87.29	172.08
14	12	5	−110.67	161.51
−22	13	−5	171.94	110.27
−25	13	−5	−138.93	120.04
8	17	0	−71.31	110.54
10	18	1	19.02	113.18
3	20	−2	88.35	94.29
−25	20	−6	0.00	152.00
3	23	−2	233.75	102.61
7	28	0	167.45	162.44
−9	−10	4	216.32	80.95
−18	−9	3	130.61	108.55
−25	−9	5	−38.56	144.47
−26	−9	7	77.92	151.87
−29	−8	6	−115.95	168.38
−23	−7	2	134.97	160.85
−27	−3	0	156.76	170.36
10	1	9	31.30	147.78
−33	1	−1	156.89	162.70
10	3	10	88.88	148.70
8	3	2	468.95	84.78
−32	3	−2	−70.92	157.68
12	4	5	175.77	111.72
8	7	1	87.56	84.39
−26	8	−4	3.96	116.87
−12	10	−4	21.13	86.24
11	12	2	36.32	102.88
−3	12	−3	437.65	49.79
14	15	5	−61.54	174.06
−35	15	−5	−10.43	166.53
−37	16	−5	0.00	161.91
−1	17	−3	0.00	70.39
14	18	5	0.00	162.44
8	20	0	−7.13	111.99
9	26	1	96.67	163.49
5	27	−1	15.98	158.47
−16	−11	7	−101.82	117.93
−23	−9	4	46.35	145.93
−5	−7	2	316.68	62.86
−30	−7	4	110.40	160.06
−8	−6	1	124.80	76.99
11	1	7	−41.86	106.97
−25	1	−2	−31.03	117.80
12	4	8	155.04	163.36
9	4	11	132.06	168.64
13	6	7	−9.51	153.72
−17	8	−4	226.22	103.40
−31	9	−4	−69.33	167.45
14	11	6	−3.43	181.58
−28	13	−5	312.72	165.87
13	21	4	95.88	168.25
−19	−10	9	125.99	129.42
−24	−9	9	−53.88	151.21

−18	−8	2	11.36	109.21
−28	−7	3	74.61	144.61
−1	−4	12	131.80	125.33
−31	0	−1	−201.13	171.42
3	3	0	47.15	64.05
10	4	3	622.01	123.08
7	4	12	115.29	173.26
12	6	9	231.77	148.57
−9	6	−3	73.69	62.46
13	8	5	−128.63	119.65
10	8	2	30.37	115.03
9	11	1	501.70	129.02
13	12	4	29.58	116.35
−33	14	−5	−18.62	155.17
11	21	2	−79.24	164.81
−12	−11	8	33.28	95.08
−17	−11	6	0.00	115.69
−18	−10	4	24.43	107.76
−1	−7	3	213.28	36.71
−28	−6	2	−60.88	163.36
−24	−2	−1	196.90	122.55
−30	2	−2	0.00	172.34
11	4	4	0.00	123.21
−26	4	−3	3.30	118.99
13	7	6	113.57	122.82
13	8	8	29.05	150.81
12	12	3	194.26	118.46
14	13	7	−22.71	153.06
14	14	6	−81.75	172.47
11	15	2	20.60	117.40
−5	20	−4	52.56	52.96
12	21	3	−35.52	161.25
10	26	2	104.86	158.61
2	28	−2	−0.13	149.36
−8	−11	6	28.39	68.94
−15	−11	8	−104.46	108.95
−27	−9	6	−0.53	151.74
−9	−8	2	129.95	94.03
−23	−6	1	−16.77	118.33
−14	−5	0	211.30	111.86
−17	−5	0	200.47	115.69
9	0	9	1.72	141.31
−17	0	−2	505.00	135.36
−20	0	−2	110.54	109.61
9	2	10	33.94	139.06
1	5	−1	726.07	75.41
−31	5	−3	20.73	168.25
−19	13	−5	42.66	97.46
13	15	4	−0.13	141.17
13	18	4	171.15	173.13
−26	19	−6	42.52	156.36
−29	19	−6	113.31	148.17
6	30	0	0.00	146.98
−12	−11	5	36.98	85.84

−15	−11	5	−0.40	101.16
−28	−8	4	−94.56	148.57
−25	−4	0	−35.79	128.76
−32	−2	0	103.54	150.42
−29	−1	−1	−168.25	171.94
10	0	7	201.66	106.18
11	2	6	45.16	104.99
8	3	11	26.54	152.66
6	3	12	54.67	156.62
11	7	10	29.32	156.23
−29	8	−4	194.13	163.23
−31	13	−5	18.22	154.91
12	15	3	−75.01	116.35
5	15	−1	247.61	106.84
12	18	3	203.51	159.79
−22	20	−6	36.58	108.82
9	23	1	52.16	152.00
5	24	−1	−66.03	116.87
7	25	0	124.01	155.83
8	28	1	93.50	154.25
−23	−10	8	0.00	136.95
−24	−10	7	59.82	134.31
−21	−9	3	0.00	144.74
−30	−8	8	5.68	181.19
−15	−7	1	17.04	97.59
3	−6	9	101.16	115.95
−4	−5	1	894.98	91.52
−20	−5	0	111.59	111.99
−28	1	−2	−106.31	150.15
11	3	5	173.26	92.31
−21	3	−3	32.35	106.04
−21	7	−4	232.96	108.69
−24	7	−4	127.70	124.14
12	8	4	0.00	102.08
11	8	3	−126.12	127.84
−13	9	−4	48.07	95.08
−36	10	−4	−3.04	143.55
4	11	−1	310.61	80.29
−23	12	−5	20.60	101.55
−26	12	−5	0.13	132.06
−32	19	−6	0.00	140.78
4	29	−1	0.53	159.93
−19	−11	7	0.00	108.42
−23	−10	5	−87.82	136.55
−21	−8	2	87.42	122.82
−26	−8	3	0.00	148.44
−18	−7	1	36.05	93.76
−26	−7	2	−21.26	158.08
0	−3	12	150.81	124.80
−16	−3	−1	504.87	131.93
−19	−3	−1	86.63	103.93
8	−1	9	12.02	141.04
−14	0	−2	1849.65	198.09
−23	0	−2	55.86	119.91

−10	1	−2	137.87	71.45
7	2	2	597.97	84.25
5	2	12	4.36	147.64
11	3	8	108.03	155.04
−18	3	−3	90.20	115.03
12	5	7	76.73	126.38
6	9	0	114.76	81.22
13	11	5	105.78	127.84
13	11	8	−93.90	155.70
−1	26	−3	76.46	105.12
5	32	0	65.37	143.02
−21	−10	4	−52.16	151.47
−12	−7	1	332.79	111.99
1	−6	10	32.22	91.78
0	−5	11	7.13	103.27
−30	−3	0	115.03	171.02
8	1	10	−150.02	137.74
7	2	11	−49.52	144.34
2	2	0	507.77	51.50
9	3	3	573.81	106.57
−24	3	−3	−2.51	113.31
−29	4	−3	163.76	154.25
11	5	9	−86.37	139.59
7	6	1	58.64	69.86
12	9	9	−66.03	143.15
−34	9	−4	37.37	155.17
1	12	−2	291.85	66.69
−29	12	−5	−1.58	156.36
9	14	1	−29.05	102.48
−36	14	−5	54.15	142.23
−23	19	−6	−42.39	134.31
9	20	1	135.23	116.61
5	21	−1	67.09	100.50
9	28	2	18.49	155.57
−9	−11	5	59.03	78.58
−18	−11	5	53.88	110.01
−18	−11	8	−72.90	116.35
−20	−11	6	0.00	119.25
−25	−10	6	−82.80	145.93
−26	−9	4	63.79	135.63
−11	−5	0	620.69	103.54
−7	−4	0	915.45	101.55
7	−2	9	−106.97	141.83
−9	−2	−1	681.04	94.03
−27	−2	−1	242.60	154.38
9	−1	7	118.19	104.20
4	1	12	−5.68	143.42
10	3	4	366.47	119.78
−10	5	−3	675.23	89.67
−18	7	−4	86.90	94.42
−27	7	−4	53.88	123.87
13	10	6	69.86	142.76
13	14	5	87.42	161.51
2	16	−2	108.69	91.39

13	17	5	49.79	172.74
9	17	1	60.35	112.38
5	18	−1	13.07	91.65
−27	18	−6	95.08	157.02
7	22	0	18.62	109.35
10	23	2	3.96	168.38
2	25	−2	−60.09	103.27
−7	−11	7	62.33	71.31
−9	−11	8	18.75	74.35
−28	−9	5	−0.13	152.27
−28	−9	8	304.40	163.36
3	−7	7	302.55	72.77
4	−5	9	−45.43	124.93
6	−3	9	0.00	134.83
−13	−3	−1	300.44	106.97
−22	−3	−1	−77.65	118.85
1	−2	12	−19.81	128.63
3	0	12	−44.37	141.04
−33	2	−2	5.68	146.59
12	6	6	7.79	116.61
10	6	10	17.43	146.32
12	7	5	153.32	117.53
12	7	8	49.52	145.27
−32	8	−4	−167.85	162.83
10	11	2	170.23	113.31
−20	12	−5	67.75	101.42
−34	13	−5	−24.56	141.17
−30	18	−6	93.10	145.80
−1	20	−3	108.55	76.60
7	30	1	100.37	154.25
1	30	−2	0.00	152.79
4	34	0	−38.30	151.61
−22	−10	9	−8.98	134.97
−29	−9	7	−24.30	163.89
−21	−7	1	69.99	115.69
−23	−5	0	−7.66	112.78
5	−4	9	122.95	132.33
2	−1	12	122.68	136.15
7	0	10	229.79	130.87
−26	0	−2	48.33	126.51
10	1	6	−27.34	97.20
6	1	11	−137.74	143.95
−3	1	−1	525.47	57.31
−15	3	−3	79.10	110.80
9	7	2	463.14	112.52
−14	8	−4	−22.19	102.48
12	11	4	0.00	116.08
13	12	7	−28.53	153.72
13	13	6	67.75	171.68
13	16	6	76.60	151.21
7	16	0	224.11	101.82
7	19	0	155.44	104.33
12	20	4	13.60	171.68
−1	23	−3	114.23	87.03

−5	26	−4	45.16	76.33
6	27	0	69.86	157.68
3	31	−1	−94.56	146.59
−13	−10	3	73.95	92.18
−16	−10	3	137.21	100.23
−6	−9	3	101.16	77.12
−24	−9	3	37.90	141.70
−31	−7	3	−131.93	155.44
−31	−6	2	0.00	137.61
−28	−4	0	−59.43	162.96
8	−2	7	100.23	98.52
10	2	5	220.94	106.04
10	2	8	152.40	145.80
−27	3	−3	78.84	132.72
−34	5	−3	−0.13	142.10
8	10	1	11.09	100.76
11	11	3	122.68	106.18
−32	12	−5	−25.62	152.00
−14	15	−5	17.43	72.24
−24	18	−6	171.42	140.78
−33	18	−6	−70.39	136.68
10	20	2	−8.45	108.16
8	25	1	0.00	160.85
−6	−10	4	12.41	73.95
−16	−9	2	−101.42	103.14
−24	−8	2	184.75	158.08
2	−5	10	1.85	98.91
1	−4	11	112.25	108.55
−32	−1	−1	−7.26	151.21
5	0	11	−75.01	133.12
6	1	2	142.63	61.80
−31	1	−2	22.32	162.44
10	4	9	179.60	142.23
−30	7	−4	36.05	171.28
−24	11	−5	−1.19	104.72
−27	11	−5	106.04	139.19
12	14	4	0.00	121.89
10	14	2	188.45	108.55
−15	14	−5	41.34	80.43
−13	16	−5	2.25	66.43
12	17	4	0.79	153.45
11	20	3	256.86	172.60
2	22	−2	147.51	97.99
−5	23	−4	33.68	64.97
4	26	−1	42.66	127.84
6	32	1	−35.79	143.55
−13	−11	4	78.05	90.46
−22	−11	7	102.22	129.55
−15	−10	10	−23.90	98.39
−31	−5	1	−36.58	138.93
−25	−3	−1	−23.24	123.87
−18	−1	−2	64.31	102.08
−21	−1	−2	45.43	116.87
1	1	0	−2.11	6.87

11	4	7	94.69	109.87
−32	4	−3	−11.75	147.78
−22	6	−4	178.68	109.61
11	7	4	124.53	102.08
10	7	3	242.46	120.57
12	8	7	91.65	148.57
10	17	2	94.16	125.06
−28	17	−6	16.51	146.98
2	19	−2	−0.26	82.41
−16	−11	4	0.00	93.76
−21	−11	5	96.01	123.74
−19	−10	3	135.23	119.52
−24	−10	4	−133.65	135.89
−26	−10	5	20.07	141.44
−19	−9	2	−57.45	103.54
−30	−9	6	143.42	159.13
−29	−8	3	−19.94	144.74
−31	−8	4	−54.01	146.72
−29	−7	2	30.24	134.44
4	−6	7	−42.26	74.61
7	−3	7	247.61	97.99
2	−3	11	−32.09	110.80
4	−1	11	−159.66	133.12
8	2	3	121.10	77.26
−22	2	−3	79.37	108.55
8	6	11	56.79	155.96
−25	6	−4	0.00	113.31
11	8	9	83.46	146.59
12	10	5	55.20	114.10
12	10	8	−62.46	141.31
−21	11	−5	344.94	106.71
11	14	3	210.77	120.04
11	17	3	339.79	126.12
−31	17	−6	−7.53	135.10
10	25	3	113.84	153.45
9	25	2	133.91	161.38
−21	−11	8	37.64	120.70
−10	−10	3	−73.03	82.67
−26	−10	8	−106.97	143.29
−27	−10	7	−50.05	141.97
−9	−7	1	252.90	84.65
−15	−6	0	232.30	116.21
−26	−5	0	−114.23	159.27
3	−4	10	88.35	104.20
4	−3	10	35.92	108.16
3	−2	11	−97.73	116.61
−30	−2	−1	5.02	154.78
9	0	6	68.80	91.52
−11	0	−2	442.27	84.39
−29	0	−2	310.74	161.64
9	2	4	835.95	126.38
−19	2	−3	12.81	101.95
9	5	10	95.61	138.14
11	6	8	−48.60	159.27

6	6	12	−45.69	160.32
−19	6	−4	129.95	99.97
−35	8	−4	−1.98	137.34
12	9	6	61.54	117.40
10	9	10	56.26	146.59
−30	11	−5	0.00	160.32
−16	13	−5	−48.07	82.54
−25	17	−6	−56.13	144.47
12	19	5	−143.95	152.53
8	22	1	50.05	116.08
5	29	0	−78.97	148.96
−23	−11	6	−72.90	123.08
5	−5	7	62.73	80.03
6	−4	7	56.39	81.35
−15	−1	−2	238.37	108.29
−24	−1	−2	3.30	108.29
9	1	8	81.75	125.06
−25	2	−3	−14.00	108.82
−30	3	−3	83.99	172.21
3	5	13	−0.13	162.57
11	6	5	20.87	102.22
−28	6	−4	0.00	152.00
−15	7	−4	217.50	106.97
−35	12	−5	31.03	145.93
11	22	4	−48.07	144.74
6	24	0	229.39	123.48
7	27	1	−29.05	160.98
−2	28	−3	−21.66	119.65
−14	−11	9	65.11	91.91
−19	−11	4	109.61	141.17
−12	−10	10	−14.53	71.58
−29	−9	4	−54.15	143.42
−21	−6	0	26.02	119.52
−29	−6	1	84.92	131.80
0	−5	2	68.54	21.13
−17	−4	−1	169.17	110.54
−20	−4	−1	109.61	111.59
9	3	9	87.16	139.72
6	5	1	121.50	64.45
−33	7	−4	188.05	149.49
5	8	0	757.37	93.24
12	13	5	−85.05	132.99
4	14	−1	646.44	111.33
12	16	5	−42.00	171.42
−12	17	−5	72.90	59.43
−34	17	−6	41.47	137.61
−20	19	−6	31.69	92.18
−19	20	−6	−3.17	89.67
1	27	−2	0.00	117.93
−14	−12	7	43.84	96.27
−15	−12	6	−10.70	101.16
−10	−11	4	228.86	84.78
−17	−11	9	−26.68	106.71
−22	−10	3	−36.18	148.96

−28	−10	6	10.43	150.95
−6	−8	2	183.17	66.69
−16	−8	1	3.43	103.27
−31	−4	0	−29.98	152.66
9	1	5	105.25	101.95
−16	2	−3	117.14	111.99
−25	10	−5	73.82	116.08
11	11	9	100.23	146.19
−17	12	−5	128.76	89.14
8	13	1	59.82	115.69
4	23	−1	7.92	100.76
−12	−12	6	41.07	90.86
−22	−9	2	25.09	142.49
−27	−9	3	−76.60	137.21
−19	−8	1	−70.78	111.99
−27	−8	2	−10.83	147.38
−23	−4	−1	25.62	109.21
−10	−3	−1	161.77	74.09
−28	−3	−1	−24.83	155.96
5	0	2	110.54	54.01
−4	0	−1	3242.63	274.16
10	3	7	58.77	105.52
2	4	13	0.00	154.12
0	4	−1	3433.98	281.69
7	5	11	40.94	146.32
5	5	12	31.96	146.72
8	6	2	154.25	87.82
11	10	4	189.77	118.06
9	10	2	30.11	113.84
−28	10	−5	62.33	153.98
−33	11	−5	−34.47	146.19
12	12	6	9.24	149.49
12	14	7	78.44	145.80
12	15	6	69.33	161.64
−2	16	−3	42.79	67.48
−29	16	−6	−55.99	139.19
−21	18	−6	130.21	103.80
8	19	1	−57.97	114.36
9	22	2	−82.67	140.91
8	27	2	−0.40	144.87
3	28	−1	0.79	148.44
4	31	0	0.00	136.82
−17	−12	7	104.99	103.80
−18	−12	6	−48.33	108.55
−13	−8	1	80.95	107.63
−5	−7	11	15.32	69.99
−12	−6	0	402.39	113.18
−8	−5	0	737.16	94.56
−14	−4	−1	527.72	120.97
8	−1	6	15.98	87.16
−27	−1	−2	49.13	140.64
8	0	8	−44.37	108.82
−28	2	−3	194.53	154.38
8	4	10	−14.39	128.76

−31	6	−4	0.66	151.47
10	7	9	42.13	132.06
−22	10	−5	42.52	103.14
−26	16	−6	143.95	146.72
−32	16	−6	39.62	131.40
11	19	4	−78.84	166.13
−16	−12	5	−27.20	106.31
−24	−11	5	26.81	128.76
−27	−7	1	5.15	129.02
−5	−6	1	72.90	49.52
−24	−6	0	−5.68	133.65
−23	5	−4	233.48	114.76
−26	5	−4	−82.41	116.48
11	7	7	−4.23	131.66
11	9	8	−69.46	140.25
10	10	3	31.69	100.63
4	17	−1	192.41	98.12
−22	17	−6	−38.30	112.38
4	20	−1	144.34	94.42
6	21	0	41.86	103.27
6	29	1	−5.02	140.78
−13	−12	5	59.56	87.56
−22	−11	4	−66.95	131.80
−25	−11	7	−53.09	126.25
−27	−10	4	2.64	137.08
−29	−5	0	0.00	147.25
−19	−2	−2	30.11	107.23
−32	0	−2	−33.54	143.95
8	1	4	875.96	122.42
7	1	3	189.77	67.75
8	2	9	−88.22	133.78
−33	3	−3	72.37	148.70
10	5	8	0.00	143.68
−20	5	−4	50.45	99.84
9	6	3	309.42	106.97
−16	6	−4	67.48	103.01
9	8	10	−35.79	138.14
7	9	1	585.29	102.74
−31	10	−5	−11.23	141.83
−18	11	−5	350.75	98.78
11	13	4	16.11	105.65
6	15	0	0.00	103.67
11	16	4	−45.83	127.70
−37	17	−6	10.43	139.85
10	19	3	197.43	124.80
−11	−12	7	−44.50	84.65
−20	−11	9	−61.41	117.01
−24	−11	8	74.75	127.44
−29	−10	5	−78.71	149.49
−22	−2	−2	75.01	105.52
−20	1	−3	77.26	116.61
−23	1	−3	12.28	113.18
4	4	12	0.00	139.85
11	8	6	−15.72	109.61

11	9	5	−37.11	118.85
9	13	2	−5.55	101.82
6	18	0	100.10	97.06
7	24	1	−22.05	135.76
5	26	0	0.00	136.95
7	29	2	4.36	141.31
3	33	0	96.27	131.40
−19	−12	5	−48.60	115.16
−20	−12	7	−0.40	111.46
−11	−11	9	−10.96	67.48
−26	−11	6	54.01	138.40
−25	−9	2	−109.08	143.55
1	−8	6	37.11	42.79
−26	−4	−1	0.00	142.89
7	−2	6	149.23	81.88
−12	−1	−2	266.24	96.14
−5	3	−2	335.83	53.75
6	4	11	−180.40	138.66
−4	4	−2	2669.09	222.92
10	5	5	−3.96	99.18
−29	5	−4	41.07	148.83
11	12	8	85.84	135.10
10	13	3	66.95	111.72
1	15	−2	281.69	82.27
−30	15	−6	0.00	132.46
10	16	3	134.70	110.40
9	16	2	3.83	116.48
−23	16	−6	−2.77	118.33
−35	16	−6	−22.32	137.08
1	24	−2	23.24	89.41
−2	25	−3	−29.32	84.39
0	29	−2	49.26	138.40
2	30	−1	0.00	150.15
−21	−12	6	8.98	114.89
−14	−11	3	123.48	93.10
−17	−11	3	50.71	100.23
−17	−10	2	54.81	90.46
−25	−10	3	34.34	130.08
0	−8	8	−24.04	63.65
−4	−8	10	−49.26	58.24
−31	−3	−1	0.26	137.74
−16	−2	−2	243.92	120.44
−25	−2	−2	0.00	122.16
7	−1	8	−84.39	103.80
−30	−1	−2	−102.74	155.17
−26	1	−3	62.73	135.10
9	2	7	163.36	99.97
−31	2	−3	−38.03	155.44
7	3	10	−61.80	125.46
−26	9	−5	−76.46	121.36
10	10	9	15.98	129.42
−27	15	−6	−37.24	147.91
11	18	5	−27.60	164.15
8	24	2	−141.83	163.76

5	31	1	158.87	150.42
2	35	0	−102.61	133.91
−16	−12	8	2.77	96.14
−6	−11	5	62.20	58.64
−14	−10	2	178.94	96.14
−30	−10	7	134.57	159.27
−30	−8	2	−136.02	139.19
−19	−7	0	321.30	117.80
−27	−6	0	95.48	162.44
4	−1	2	160.98	50.71
−5	−1	−1	960.61	107.10
−17	1	−3	−24.17	105.78
9	6	9	8.19	131.53
−34	6	−4	−60.22	135.10
−23	9	−5	6.34	99.31
−29	9	−5	−72.63	150.95
−19	10	−5	80.82	95.35
−34	10	−5	35.00	131.00
0	11	−2	444.25	62.46
11	12	5	83.99	120.70
11	15	5	201.66	134.57
−33	15	−6	24.30	130.48
3	25	−1	126.12	107.37
−20	−11	3	76.73	139.06
−20	−10	2	−109.48	116.74
−30	−9	3	−18.49	137.08
−10	−8	1	6.74	96.54
−16	−7	0	304.27	103.54
−18	−5	−1	76.99	102.61
−21	−5	−1	241.54	115.95
7	1	9	−149.89	133.38
−6	2	−2	81.09	51.50
3	3	12	93.50	132.06
11	11	6	61.28	129.29
11	13	7	0.00	146.32
11	16	7	185.81	150.02
11	17	6	134.04	149.49
10	21	4	−63.13	164.68
4	33	1	−24.43	137.48
−9	−12	6	−45.30	69.99
−10	−12	5	18.62	74.48
−13	−12	8	−23.51	83.46
−19	−12	8	21.53	106.44
−25	−11	4	−68.80	121.76
−7	−10	3	357.36	90.20
−22	−7	0	148.96	115.82
−30	−7	1	89.14	138.00
−11	−4	−1	226.22	83.73
−13	2	−3	−43.98	94.29
5	3	11	54.41	131.00
9	4	8	−29.45	144.08
5	4	1	837.93	93.90
−24	4	−4	122.29	107.50
−17	5	−4	69.33	92.18

10	6	7	11.89	109.87
8	7	10	−84.52	132.85
10	8	8	−18.75	145.53
10	9	4	−14.79	96.14
11	14	6	260.69	160.72
7	21	1	18.88	106.84
1	21	−2	7.00	83.73
4	28	0	95.74	151.34
1	32	−1	38.43	134.04
−14	−12	4	17.17	88.08
−17	−12	4	−29.98	104.33
−22	−12	5	−163.62	124.67
−4	−4	12	65.37	99.57
−29	−4	−1	−109.74	145.40
6	−3	6	220.01	78.44
6	−2	8	14.39	94.29
−28	−2	−2	38.96	159.27
7	0	4	2220.21	205.62
6	0	3	235.60	66.56
−29	1	−3	40.01	151.47
6	2	10	−8.06	118.72
−21	4	−4	187.00	109.35
−27	4	−4	3.57	127.44
7	5	2	241.94	78.58
−32	5	−4	55.60	137.21
8	9	2	182.77	104.99
−32	9	−5	63.39	136.95
7	12	1	444.52	117.14
1	18	−2	95.22	77.52
−2	19	−3	104.20	66.69
−2	22	−3	115.69	78.18
5	23	0	−8.32	101.95
6	26	1	184.75	165.21
−23	−12	7	16.11	114.63
−5	−11	6	75.80	50.32
−11	−11	3	51.64	79.10
−15	−5	−1	191.88	115.03
−24	−5	−1	−26.15	112.91
2	2	12	−9.64	125.46
9	5	4	338.08	116.48
4	7	0	899.07	95.88
5	11	0	468.95	89.41
−28	14	−6	114.23	149.10
−31	14	−6	−113.18	136.29
−36	15	−6	66.16	141.04
10	18	4	8.32	123.87
9	21	3	−35.13	135.10
8	21	2	−14.66	111.46
8	26	3	−198.09	148.30
−1	31	−2	−74.22	148.30
−20	−12	4	5.68	128.76
−24	−12	6	9.90	117.80
−23	−11	3	77.52	128.76
−28	−11	7	−22.05	136.15

−23	−10	2	123.08	143.68
−28	−10	3	−142.76	128.36
−30	−10	4	85.05	142.63
−28	−9	2	37.90	137.48
−13	−7	0	122.16	117.67
−9	−6	0	298.19	79.76
−20	−3	−2	−62.20	108.29
−2	−2	0	466.31	47.67
−21	0	−3	127.17	105.38
−24	0	−3	27.60	105.12
−7	1	−2	155.30	57.18
−27	8	−5	75.14	140.91
−20	9	−5	1.85	93.37
7	18	1	222.92	104.46
7	26	2	3.57	162.44
−7	−11	4	59.03	64.18
−27	−11	8	33.28	133.25
−14	−9	1	101.42	93.63
−28	−8	1	4.09	131.00
−25	−7	0	37.77	152.00
−30	−6	0	67.62	134.70
5	−4	6	116.87	71.58
5	−3	8	0.00	86.10
−23	−3	−2	111.46	111.86
−13	−2	−2	501.44	115.69
6	0	9	112.52	129.55
4	2	11	5.02	124.27
9	4	5	−41.20	101.29
−30	4	−4	−22.58	148.17
8	5	3	253.95	102.35
10	7	6	416.52	119.52
10	8	5	91.78	99.44
−11	8	−4	20.73	76.73
−24	8	−5	0.00	105.91
9	9	9	0.26	123.61
10	11	8	−29.58	130.74
10	12	4	183.70	109.87
3	13	−1	234.67	82.93
−25	14	−6	96.80	136.15
−34	14	−6	67.22	138.40
10	15	4	195.19	111.72
−11	18	−5	267.56	50.71
10	20	5	−118.19	154.25
3	22	−1	49.39	94.29
−29	−11	6	16.38	140.64
−7	−9	2	385.49	85.84
1	−7	8	30.37	67.48
−17	−3	−2	218.16	103.14
−6	−2	−1	1548.15	145.14
1	1	12	−31.17	120.57
8	5	9	−60.62	127.17
4	7	12	−37.24	148.04
−30	8	−5	0.00	139.32
−10	9	−4	45.96	67.09

−35	9	−5	19.68	117.01
8	10	10	40.94	144.08
10	14	8	−47.15	128.23
9	18	3	83.20	104.86
9	23	4	−14.13	146.98
0	26	−2	129.68	97.46
2	27	−1	159.53	117.80
3	30	0	0.00	138.66
−11	−12	4	56.92	80.82
−22	−12	8	−31.03	114.89
2	−7	6	−28.00	52.43
−6	−7	1	9.11	58.64
−27	−5	−1	−9.24	149.76
−3	−3	12	0.00	98.91
−26	−3	−2	128.23	135.10
3	−2	2	318.93	50.98
−31	−2	−2	−86.10	129.82
−18	0	−3	228.99	96.67
−27	0	−3	12.81	145.00
5	1	10	66.03	116.08
−14	1	−3	121.76	106.71
−32	1	−3	33.15	132.99
8	3	8	19.15	119.12
−18	4	−4	288.82	99.31
7	6	10	−40.67	121.63
1	6	13	−81.35	147.38
−12	7	−4	254.75	89.67
6	8	1	295.95	77.52
10	9	7	100.76	129.82
8	18	2	49.52	115.55
10	19	6	9.64	137.21
5	20	0	58.90	101.03
5	28	1	23.37	158.21
−16	−13	6	−52.30	99.57
−23	−12	4	−39.75	123.08
−28	−11	4	−91.39	132.19
−2	−8	9	−25.36	61.01
−4	−6	11	0.00	76.99
4	−5	6	115.95	63.65
4	−4	8	346.92	93.10
0	0	12	−38.69	116.87
−25	3	−4	85.71	111.06
10	11	5	126.12	106.57
9	12	3	−29.71	97.20
9	12	9	−163.23	134.57
−29	13	−6	77.12	133.91
9	15	3	101.42	104.20
−20	16	−6	12.81	89.01
10	17	5	49.79	156.36
−19	17	−6	−23.64	84.12
6	23	1	10.96	106.18
−3	27	−3	46.62	94.82
6	28	2	−2.11	137.21
−26	−10	2	84.78	128.76

−2	−9	4	566.94	64.71
3	−6	6	165.21	58.37
2	−6	8	33.81	73.03
3	−5	8	14.13	76.46
−2	−2	12	−26.28	104.33
5	−1	9	0.00	117.93
−1	−1	12	−103.40	114.10
−8	0	−2	526.00	78.44
3	1	11	−15.85	119.78
−22	3	−4	31.96	100.23
9	5	7	37.51	110.67
−13	6	−4	91.65	93.63
9	7	8	22.71	141.17
−21	8	−5	3.96	94.95
−33	8	−5	176.96	126.12
10	10	6	116.74	112.65
−9	10	−4	110.27	65.37
10	12	7	0.26	155.70
−26	13	−6	34.07	139.46
−32	13	−6	−51.24	135.49
10	14	5	100.76	114.89
5	14	0	174.58	96.67
10	15	7	118.99	145.80
8	15	2	120.70	97.06
−21	15	−6	115.03	97.46
−18	18	−6	12.55	77.78
3	19	−1	108.29	83.46
−6	25	−4	62.99	64.97
2	32	0	−49.26	130.74
−17	−13	5	31.96	100.23
−26	−11	3	99.05	122.16
−30	−11	5	2.51	118.99
−17	−8	0	−9.24	104.72
−20	−8	0	39.75	101.29
−28	−7	0	167.19	135.89
−19	−6	−1	40.54	104.46
−22	−6	−1	53.88	114.63
−12	−5	−1	888.90	130.21
−3	−3	0	24.96	56.79
6	−1	4	580.28	87.69
7	0	7	159.93	94.82
−30	0	−3	0.00	138.27
−28	3	−4	41.07	157.42
−33	4	−4	270.59	134.83
10	16	6	81.75	151.87
3	16	−1	316.42	102.74
5	17	0	217.90	90.46
7	23	2	0.00	136.55
4	25	0	−8.45	104.99
−13	−13	6	52.69	94.16
−14	−13	5	127.31	92.05
−15	−13	7	−4.36	90.33
−18	−13	7	−23.24	99.97
−18	−12	3	−152.00	121.89

−26	−12	7	−115.42	123.48
−27	−12	6	6.07	131.66
−18	−11	2	0.00	95.74
−26	−9	1	−155.83	142.63
−29	−3	−2	0.00	140.78
5	−1	3	194.66	56.79
4	0	10	52.16	112.25
8	2	6	87.03	88.88
0	5	13	0.00	143.42
5	6	11	−19.81	133.38
3	6	12	64.31	136.29
−25	7	−5	141.83	116.87
−28	7	−5	−59.03	148.70
9	8	4	−6.07	102.88
−15	11	−5	41.60	75.94
−14	12	−5	88.88	73.43
10	13	6	−76.86	130.48
−35	13	−6	0.00	138.53
9	20	4	−54.67	147.64
9	22	5	5.02	139.59
8	25	4	0.00	133.25
4	30	1	−31.83	154.64
−20	−13	5	−60.35	109.61
−15	−12	3	70.12	90.73
−15	−11	2	220.94	97.46
−21	−11	2	−130.87	138.93
−11	−9	1	281.95	104.20
−23	−8	0	−13.87	123.48
−16	−6	−1	112.25	108.82
−25	−6	−1	−46.62	135.63
−30	−5	−1	88.74	128.10
−7	−3	−1	1072.07	111.33
−22	−1	−3	169.57	111.06
2	0	11	2.11	116.74
8	4	4	103.27	97.20
7	4	9	−48.73	130.74
8	8	9	−123.87	122.68
−16	10	−5	31.43	81.75
−22	14	−6	−33.41	104.20
1	29	−1	22.85	138.66
5	30	2	141.04	139.46
1	34	0	−12.41	129.68
−22	−13	6	26.41	107.89
−8	−12	7	80.56	78.31
−10	−12	8	0.00	64.58
−21	−12	3	238.63	142.49
−25	−12	8	−35.13	119.65
−21	−4	−2	−55.99	108.95
−14	−3	−2	628.35	118.46
4	−2	9	96.54	121.10
−25	−1	−3	96.93	117.01
4	3	1	2832.71	243.52
−19	3	−4	236.92	100.37
−31	3	−4	−54.41	148.83

6	4	2	1019.38	114.50
6	5	10	7.26	118.33
−14	5	−4	335.83	107.23
9	6	6	219.49	105.38
−31	7	−5	−42.39	137.08
7	9	10	9.77	124.67
9	10	8	−23.77	131.53
−30	12	−6	37.37	136.55
−13	13	−5	112.78	64.58
−6	19	−4	111.33	41.20
−17	19	−6	−30.11	71.45
−21	−13	7	23.77	107.37
−18	−12	9	60.35	96.54
−26	−12	4	135.23	126.12
−14	−8	0	244.84	107.63
−3	−7	10	−35.79	68.28
−10	−7	0	754.33	114.76
−1	−6	2	92.05	29.85
−3	−5	11	−0.13	81.09
−24	−4	−2	0.00	117.27
−9	−1	−2	48.99	66.82
−19	−1	−3	45.30	94.69
−15	0	−3	109.61	104.72
7	2	8	25.88	106.44
8	3	5	225.43	101.03
9	7	5	−56.65	93.76
−22	7	−5	91.25	95.61
7	8	2	158.34	93.76
−17	9	−5	51.90	87.69
−27	12	−6	7.13	145.27
9	13	8	−10.04	125.85
9	17	4	−38.56	110.93
8	20	3	−27.73	115.03
6	20	1	211.03	104.46
0	23	−2	94.16	86.37
2	24	−1	0.00	94.29
−1	28	−2	42.13	111.33
3	32	1	0.00	124.80
0	36	0	−32.49	124.27
−28	−12	5	55.73	132.85
−29	−10	2	17.43	124.27
−26	−8	0	121.23	151.61
−18	−4	−2	38.69	100.50
5	−3	5	554.13	94.16
1	−1	11	−9.11	102.35
−28	−1	−3	−63.26	154.51
7	4	3	1257.09	137.48
9	8	7	40.15	110.93
8	8	3	254.88	105.65
9	11	4	106.71	99.31
6	11	1	529.04	106.18
−33	12	−6	33.28	127.84
9	19	5	−43.58	147.64
−6	22	−4	84.65	53.88

7	25	3	84.65	153.32
5	25	1	−25.88	124.53
3	27	0	50.58	126.91
−15	−13	4	−49.79	93.76
−18	−13	4	28.66	101.42
−23	−13	5	−0.53	103.40
−15	−12	9	−22.58	81.75
−8	−11	3	122.29	79.76
−24	−11	2	54.94	135.89
−15	−10	1	41.47	92.44
−29	−9	1	−8.32	124.14
−28	−6	−1	−65.37	136.82
−4	−4	0	352.60	63.92
−27	−4	−2	−52.30	143.68
3	−3	9	124.14	120.70
6	−1	7	49.13	86.63
−23	2	−4	138.80	97.33
8	4	7	107.63	101.16
2	5	12	−10.43	127.04
8	6	8	134.17	147.12
−34	7	−5	0.00	131.66
8	11	9	33.54	134.70
9	14	4	168.38	99.97
4	22	0	−37.11	93.10
6	25	2	2.25	139.46
−4	29	−3	14.00	105.52
0	31	−1	−27.73	143.15
2	34	1	−3.30	124.14
−21	−13	4	−36.45	116.21
−12	−12	3	50.05	83.33
−24	−12	3	61.94	128.63
−12	−11	2	114.10	84.39
−29	−11	3	57.84	122.42
−24	−10	1	53.75	150.15
−7	−8	1	−4.49	64.58
−2	−4	11	18.22	86.50
2	−3	2	401.47	46.49
0	−2	11	23.90	97.46
−15	4	−4	89.14	98.25
4	5	11	−3.30	128.10
−26	6	−5	135.36	131.80
−29	6	−5	64.05	148.83
−18	8	−5	178.55	91.91
9	17	7	38.03	132.85
9	18	6	41.60	143.42
8	22	4	41.73	158.87
−3	24	−3	91.39	78.97
−11	−13	5	−4.89	74.88
−1	−3	11	21.39	89.41
5	−2	4	366.73	72.11
2	−2	10	0.79	90.07
9	9	6	128.50	109.87
9	10	5	170.09	106.44
4	10	0	1217.73	126.12

−24	12	−6	149.89	119.78
9	14	7	44.64	149.63
6	14	1	265.84	108.03
0	14	−2	1251.54	126.51
9	16	5	−69.86	119.78
8	17	3	77.65	118.85
−17	−13	8	−41.07	93.37
−24	−13	7	171.94	115.03
−25	−13	6	209.18	121.23
−7	−12	5	149.89	66.69
−29	−12	7	−118.85	138.80
−20	−7	−1	43.98	98.52
−23	−7	−1	26.81	111.20
−13	−6	−1	−11.09	103.67
2	−4	9	0.00	107.76
−8	−4	−1	209.58	71.05
−31	−1	−3	344.81	133.25
7	1	6	−63.39	83.59
−20	2	−4	22.58	100.10
6	3	9	0.00	125.33
5	4	10	124.80	112.52
−7	5	−3	206.54	52.82
3	6	0	488.36	68.80
−23	6	−5	119.91	101.95
8	11	3	19.94	110.67
−28	11	−6	178.02	149.49
−31	11	−6	71.31	132.06
9	13	5	6.74	102.08
−12	14	−5	117.53	60.88
7	17	2	80.16	103.01
6	27	3	−66.03	150.02
−20	−13	8	−76.99	103.54
−27	−11	2	−56.65	130.61
−18	−9	0	102.08	99.84
−21	−9	0	181.58	110.54
0	−6	9	21.53	83.86
−2	−6	10	0.00	73.29
1	−5	9	15.72	102.88
−15	−4	−2	272.05	118.72
−30	−4	−2	0.00	127.70
4	−2	3	622.67	72.24
−10	−2	−2	364.36	78.44
−23	−2	−3	56.65	106.31
−16	−1	−3	31.69	109.35
6	1	8	51.37	94.42
1	4	12	−7.40	118.06
−32	6	−5	−64.97	121.50
7	7	9	2.51	122.68
6	8	10	−1.45	120.57
7	11	2	278.52	106.71
−8	11	−4	69.73	53.62
−34	11	−6	25.75	136.68
9	12	6	−44.90	116.21
8	14	3	15.72	96.27

9	15	6	71.31	140.78
0	20	−2	57.45	73.43
4	27	1	−8.72	146.06
2	29	0	4.36	138.66
−2	30	−2	36.98	134.57
−1	33	−1	−131.14	125.46
−10	−13	6	20.47	74.09
−24	−13	4	−12.28	115.69
−8	−12	4	45.43	66.95
−29	−12	4	85.44	126.38
−17	−7	−1	206.54	96.80
4	−4	5	1069.96	116.74
1	−3	10	−85.18	94.29
−20	−2	−3	28.66	98.65
−26	−2	−3	−72.90	138.93
−16	3	−4	183.43	94.69
5	7	1	775.59	94.95
−19	7	−5	278.91	101.55
8	9	8	0.26	144.08
7	14	2	124.40	106.84
2	21	−1	−21.00	85.97
7	22	3	−1.58	130.74
5	27	2	−50.05	157.68
−12	−13	4	75.67	79.50
−26	−13	5	−173.53	129.29
−19	−12	2	−67.09	119.52
−27	−12	3	50.05	122.16
−12	−10	1	214.20	95.48
−27	−10	1	18.49	117.27
−24	−9	0	8.06	139.72
−26	−7	−1	58.50	138.80
−1	−5	10	88.08	80.43
−5	−5	0	1.19	52.96
−22	−5	−2	−97.59	101.16
−25	−5	−2	−187.39	137.08
0	−4	10	159.66	90.33
5	−2	7	16.11	78.97
7	3	4	1534.28	163.36
3	4	11	14.66	122.55
8	7	4	101.82	107.76
−25	11	−6	−0.53	129.55
8	15	8	−42.66	124.93
0	17	−2	458.78	93.10
8	19	4	−36.45	117.14
4	19	0	358.68	96.67
−16	20	−6	−12.28	62.99
8	21	5	52.16	143.55
5	22	1	94.29	104.59
7	24	4	181.32	141.83
1	26	−1	64.18	94.29
−14	−13	8	0.13	78.97
−19	−13	3	−13.73	133.12
−15	−9	0	0.00	90.20
−11	−8	0	257.12	107.50

−19	−5	−2	87.69	106.04
−29	−2	−3	107.89	136.82
−11	1	−3	515.83	90.59
7	2	5	0.00	98.12
−27	5	−5	90.59	143.29
6	11	10	19.81	124.01
8	12	8	19.02	128.89
2	12	−1	757.90	93.50
−3	15	−3	148.17	51.64
6	22	2	133.25	109.87
5	29	3	68.80	128.36
−18	−14	5	85.71	102.48
−16	−13	3	−32.88	94.42
−23	−13	8	0.26	108.03
−22	−12	2	168.51	132.72
−19	−11	1	88.35	95.61
−2	−7	2	742.84	73.56
7	3	7	61.14	91.25
7	5	8	183.17	118.33
−24	5	−5	0.00	114.23
−30	5	−5	0.00	133.51
8	6	5	7.13	94.69
8	7	7	58.37	105.65
2	8	12	−10.43	131.66
7	10	9	−22.58	115.55
−29	10	−6	58.64	127.04
−32	10	−6	62.33	117.40
−19	14	−6	−5.55	82.67
8	20	6	40.81	127.57
3	24	0	72.63	92.05
−1	25	−2	21.53	83.46
3	29	1	−32.09	147.78
1	31	0	48.33	137.61
−20	−14	6	87.03	103.14
−22	−13	3	−43.45	115.69
−22	−11	1	98.65	137.08
−9	−5	−1	−24.04	71.05
−28	−5	−2	0.00	130.08
−11	−3	−2	632.18	103.01
5	2	9	6.74	125.46
4	3	10	−12.55	112.52
0	3	12	64.45	114.89
−6	6	−3	50.84	41.73
−20	6	−5	24.83	95.35
7	13	9	31.69	117.27
−20	13	−6	−29.45	90.46
8	16	4	−21.92	99.05
4	16	0	45.30	99.44
−3	21	−3	206.41	69.60
4	29	2	−51.77	133.12
−15	−14	5	−6.34	91.12
−21	−14	5	−4.89	104.06
−27	−13	7	−54.54	117.67
−28	−13	6	0.26	127.44

−8	−9	1	96.14	76.46
−9	−8	11	27.86	37.24
−29	−7	−1	63.79	117.67
4	−3	4	471.46	69.86
−17	−2	−3	52.16	91.12
−17	2	−4	40.15	84.52
5	3	2	877.15	95.48
−33	5	−5	60.62	119.91
7	7	3	1172.04	142.49
8	10	4	45.83	94.82
−26	10	−6	−51.90	137.08
−21	12	−6	0.00	89.93
−18	15	−6	0.00	78.18
8	16	7	−100.50	136.29
8	18	5	−11.09	130.08
2	18	−1	73.95	86.50
7	19	3	154.12	104.20
−14	−14	6	112.25	90.46
−27	−13	4	29.98	127.97
−25	−12	2	25.75	117.93
−14	−7	−1	552.28	118.72
−16	−5	−2	49.39	91.78
−24	−3	−3	−3.30	113.84
6	0	6	46.75	78.44
5	0	8	−23.90	94.16
−12	0	−3	483.61	106.04
6	3	3	55.60	69.33
2	3	11	131.66	113.97
6	6	9	90.59	121.76
6	7	2	61.41	74.61
5	7	10	48.73	115.55
8	13	4	12.94	101.29
2	15	−1	353.00	91.91
5	19	1	108.95	93.10
7	23	5	−40.67	138.40
6	26	4	93.50	128.23
2	31	1	−119.25	134.04
0	33	0	57.97	121.76
−19	−14	4	41.86	112.38
−19	−14	7	59.56	95.88
−23	−14	6	46.75	104.59
−29	−13	5	−34.34	125.46
−9	−12	3	−6.47	70.78
−13	−12	2	190.96	89.93
−25	−11	1	49.26	122.42
−21	−8	−1	39.75	99.71
−24	−8	−1	−22.05	136.55
−6	−6	0	272.84	64.84
3	−5	5	364.22	71.71
4	−3	7	108.29	75.80
−21	−3	−3	60.35	101.55
−27	−3	−3	−57.71	142.63
8	8	6	−31.69	98.78
8	13	7	71.45	146.85

8	17	6	71.05	151.87
−3	18	−3	83.33	62.46
4	24	1	69.86	95.74
0	28	−1	141.17	110.80
3	31	2	−35.00	130.87
−16	−14	7	14.26	89.80
−24	−14	5	109.74	118.72
−13	−13	3	−60.62	83.59
−25	−13	3	−153.72	115.55
−22	−10	0	0.53	128.36
−18	−8	−1	0.00	97.73
−1	2	12	−32.62	110.40
−28	4	−5	−3.96	145.66
−21	5	−5	−87.03	94.95
−2	6	13	−27.86	129.16
7	8	8	3.57	129.68
8	9	5	6.07	91.25
−30	9	−6	103.40	120.57
−1	10	−2	106.57	38.30
8	15	5	22.45	105.52
−17	16	−6	5.02	71.58
6	19	2	73.16	103.93
7	21	4	−31.69	130.21
5	24	2	−1.58	114.10
−4	26	−3	−2.77	76.73
−1	35	0	−71.18	118.59
−16	−14	4	61.01	98.25
−22	−14	4	−44.24	110.27
−22	−14	7	−120.84	106.04
−3	−10	4	309.68	57.31
−19	−10	0	11.36	94.69
−23	−6	−2	79.50	112.65
−28	0	−4	0.00	130.74
3	2	1	1036.68	107.76
−25	4	−5	23.51	124.01
−31	4	−5	118.19	119.52
1	7	12	−9.38	123.74
−27	9	−6	−94.69	139.98
−33	9	−6	40.67	120.84
5	10	1	237.31	83.07
8	11	6	67.75	104.59
8	12	5	5.81	99.31
8	14	6	0.00	114.63
7	16	3	181.98	95.35
2	26	0	14.92	96.80
2	33	2	−59.56	107.89
1	33	1	−0.13	116.74
−12	−9	0	229.65	112.25
−27	−8	−1	0.00	135.89
−10	−6	−1	87.95	82.54
−20	−6	−2	57.18	98.12
−26	−6	−2	20.73	141.04
−12	−4	−2	428.41	109.61
−30	−3	−3	−156.49	130.08

−13	−1	−3	198.09	105.12
4	1	9	0.00	124.80
3	2	10	−4.09	107.63
1	8	−1	97.06	43.45
7	10	3	186.21	112.38
5	10	10	−106.71	122.68
−23	10	−6	0.00	104.06
1	23	−1	−15.85	89.27
5	26	3	104.59	150.55
0	35	1	18.88	124.40
−28	−12	2	−13.07	134.83
−13	−11	1	68.80	83.99
−13	−11	10	38.96	32.09
−28	−11	1	−17.56	115.69
−16	−10	0	45.43	95.22
−3	−8	2	710.22	76.46
3	−3	3	1158.18	104.72
−18	−3	−3	232.82	100.37
−31	0	−4	35.00	117.40
−2	1	12	−69.60	102.35
6	4	8	22.98	106.71
7	6	7	51.77	91.39
6	9	9	103.27	121.36
7	11	8	16.51	131.00
7	13	3	48.60	96.40
7	14	8	160.85	134.97
3	21	0	113.44	91.52
−2	27	−2	128.76	88.61
−1	30	−1	71.18	132.33
−12	−14	5	−16.64	81.22
−26	−14	6	123.21	118.06
−20	−13	2	14.26	130.48
6	1	5	130.48	92.97
6	2	4	555.84	97.86
6	2	7	218.69	91.91
1	2	11	29.19	110.67
−22	4	−5	0.00	93.37
7	6	4	144.34	102.48
−15	8	−5	144.34	80.29
6	10	2	243.92	99.18
6	12	9	83.33	115.69
5	13	1	3.83	89.67
−11	15	−5	78.97	48.86
6	16	2	−73.69	87.82
7	18	7	−11.36	121.89
7	20	5	−0.13	150.29
−1	22	−2	42.00	77.12
−25	−14	4	196.37	119.65
−25	−14	7	−31.17	106.57
−17	−13	2	−38.43	101.55
−19	−13	9	−12.28	90.73
−23	−13	2	18.75	112.78
−28	−13	3	144.08	123.34
−20	−12	1	−10.30	127.04

−15	−8	−1	87.82	87.56
−7	−7	0	1663.84	159.13
2	−6	5	127.17	56.13
−17	−6	−2	17.83	88.88
−29	−6	−2	−56.26	121.23
3	−4	7	154.51	68.01
4	−1	8	−5.28	83.73
7	5	5	129.82	93.76
−3	5	13	−64.18	127.04
4	6	10	−4.09	108.16
−16	7	−5	159.40	85.44
−31	8	−6	−13.34	114.50
−14	9	−5	60.75	73.56
−24	9	−6	−14.00	117.14
6	13	2	182.11	111.33
7	18	4	17.70	96.93
7	19	6	68.41	140.78
6	21	3	85.18	100.76
6	23	4	−35.79	152.27
4	26	2	61.80	134.44
3	26	1	133.78	113.44
−13	−14	4	0.00	79.63
−20	−14	3	−58.64	119.25
−27	−14	5	−77.92	122.68
−8	−13	5	0.26	63.52
−9	−13	4	207.34	76.20
−23	−12	1	45.16	125.85
−4	−10	3	577.77	81.09
−9	−10	1	361.05	95.88
−25	−4	−3	54.67	137.08
5	−1	6	54.67	72.90
−3	0	12	98.25	102.48
−29	3	−5	−174.06	131.40
−12	4	−4	147.12	87.03
5	5	9	−100.63	124.01
−11	5	−4	265.18	82.67
0	6	12	−45.03	118.59
−17	6	−5	50.05	85.84
−28	8	−6	−63.79	129.55
3	10	11	0.00	123.48
−16	17	−6	0.00	64.84
4	28	3	−94.56	134.44
1	28	0	19.81	113.18
−13	−14	7	−33.02	78.31
−17	−14	3	−89.27	122.16
−9	−13	7	31.69	64.84
−17	−12	1	104.99	94.29
−22	−9	−1	−110.80	118.33
−22	−4	−3	74.35	101.82
−28	−4	−3	117.80	133.38
−14	−2	−3	144.21	106.04
−26	−1	−4	−96.67	152.40
−13	3	−4	535.90	109.87
−26	3	−5	70.92	135.76

7	9	7	17.70	104.99
3	9	0	556.51	80.82
−13	10	−5	23.51	68.54
7	15	7	0.00	143.29
4	21	1	228.20	97.33
−2	32	−1	40.94	134.83
−18	−14	8	68.14	89.80
−21	−14	8	−54.94	99.57
−23	−14	3	29.98	109.61
−25	−9	−1	0.00	134.97
−13	−5	−2	274.16	106.71
−4	−1	12	125.85	98.12
−23	−1	−4	10.70	99.18
−29	−1	−4	−53.88	137.34
2	1	10	47.01	102.08
0	1	11	−11.36	104.06
−32	3	−5	0.00	118.72
4	6	1	1585.13	147.78
2	6	11	−3.96	116.74
7	15	4	82.67	101.69
7	17	5	5.68	104.86
−19	−15	5	162.57	104.46
−11	−14	6	64.84	76.99
−26	−13	2	−53.09	112.78
−26	−12	1	43.32	112.12
−19	−9	−1	−3.43	95.74
−11	−7	−1	749.58	118.99
−21	−7	−2	35.52	99.18
−24	−7	−2	136.95	137.48
−6	−3	12	−2.25	87.42
−5	−2	12	−32.22	80.16
3	0	9	244.05	117.01
−14	2	−4	85.71	104.59
−23	3	−5	50.45	108.29
−18	5	−5	92.57	96.27
−10	6	−4	159.79	76.46
7	7	6	660.17	115.55
6	7	8	−40.94	133.12
−25	8	−6	−39.88	129.02
7	9	4	53.22	99.57
7	16	6	−13.07	119.25
3	18	0	62.46	88.08
3	28	2	125.99	144.87
2	28	1	−10.96	138.80
−5	28	−3	16.64	84.52
−3	29	−2	38.30	104.06
3	30	3	16.51	132.59
−3	34	−1	0.00	120.84
−18	−15	6	−3.04	92.97
−21	−15	6	9.24	95.88
−22	−15	5	0.00	108.03
−11	−13	8	13.07	61.14
−14	−13	2	−44.90	85.84
−16	−13	9	11.89	76.99

−10	−12	2	86.50	81.48
−20	−11	0	−62.46	108.03
−13	−10	0	17.17	93.76
−4	−9	2	93.63	54.81
−27	−7	−2	−39.49	121.63
−19	−4	−3	59.16	101.03
6	6	3	492.85	100.89
4	9	10	−54.54	114.36
7	12	4	183.17	92.57
6	16	8	−8.32	117.53
6	18	3	284.86	108.42
1	20	−1	−40.28	78.05
6	22	5	5.02	145.93
5	25	4	0.00	149.89
0	25	−1	28.92	86.76
0	30	0	123.21	135.10
−16	−15	5	73.43	95.22
−14	−14	3	163.36	85.84
−26	−14	3	−91.91	117.53
−28	−14	7	−23.51	117.80
−10	−13	3	3.83	77.26
−28	−9	−1	16.64	123.34
−8	−8	0	367.39	84.12
1	−7	5	143.95	45.83
2	−5	7	155.57	64.97
3	−2	8	−11.36	79.50
5	2	3	429.07	72.50
5	3	8	0.00	96.67
−29	7	−6	4.75	125.06
−32	7	−6	8.19	118.33
7	8	5	47.81	86.24
5	8	9	71.84	116.74
4	12	10	61.67	118.59
7	13	6	−23.90	97.86
7	14	5	152.00	102.61
6	21	6	−98.39	135.23
5	23	3	−3.17	112.38
2	23	0	94.82	90.59
−20	−15	4	128.76	107.89
−17	−11	0	56.52	96.67
−18	−7	−2	199.28	103.01
−15	−3	−3	7.92	99.05
5	1	7	148.70	84.39
−15	1	−4	45.69	95.74
4	2	2	77.26	53.75
−27	2	−5	61.80	131.93
−30	2	−5	−1.45	115.69
6	3	6	76.07	82.41
3	5	10	6.07	105.78
2	5	0	−24.70	56.92
−1	5	12	7.26	114.63
0	9	12	−4.62	122.82
7	10	6	185.55	102.48
6	10	8	10.17	137.87

−20	10	−6	98.12	86.90
7	11	5	250.78	98.52
−19	11	−6	85.58	84.78
−18	12	−6	−66.16	81.75
6	13	8	35.66	120.70
−1	19	−2	61.14	71.05
6	20	4	−7.13	103.40
−7	24	−4	50.58	58.37
2	30	2	−117.40	136.95
−17	−15	4	−12.28	98.25
−23	−15	4	−116.74	112.65
−24	−15	6	1.72	101.29
−25	−15	5	179.74	119.12
−16	−9	−1	29.85	87.03
−14	−6	−2	104.46	100.37
−23	−5	−3	10.56	113.04
−26	−5	−3	1.19	131.00
−1	0	11	0.00	98.39
4	4	9	22.98	122.02
6	5	7	−79.50	87.56
5	6	2	428.41	77.52
−26	7	−6	118.33	137.61
−21	9	−6	32.09	89.54
5	11	9	−14.79	113.70
−12	11	−5	−9.11	61.54
3	12	0	601.67	90.20
−30	13	−7	−23.37	112.52
5	14	9	57.45	112.65
3	15	0	117.14	87.42
5	18	2	−26.54	94.95
−4	23	−3	48.20	70.39
1	30	1	14.00	139.98
−4	31	−2	−18.36	122.16
−1	32	0	−59.69	132.06
−20	−15	7	17.96	96.54
−15	−14	8	31.17	76.20
−4	−11	4	53.48	56.92
−10	−11	1	412.03	106.18
−24	−2	−4	80.16	118.59
−27	−2	−4	63.65	134.17
−24	2	−5	−15.45	125.85
1	5	11	93.24	113.57
−9	7	−4	45.03	62.86
6	15	3	83.46	90.73
4	23	2	−37.51	105.25
3	23	1	54.81	94.29
5	24	5	−34.60	127.97
4	27	4	11.62	127.17
−18	−14	2	123.74	118.46
−21	−14	2	0.00	117.14
−21	−13	1	0.00	127.17
−5	−11	3	248.01	74.09
−12	−8	−1	271.91	113.70
−29	−5	−3	−14.79	116.21

4	−2	6	198.22	70.78
−30	−2	−4	15.85	128.76
2	−1	9	63.65	116.48
−20	3	−5	62.86	87.95
−5	7	−3	240.22	39.22
6	9	3	0.00	95.08
−17	13	−6	−37.90	72.90
−26	14	−7	34.07	114.36
6	17	7	−136.42	134.31
6	19	5	12.28	114.10
−2	24	−2	103.54	80.43
1	32	2	2.25	119.12
0	32	1	0.00	123.48
−2	34	0	0.00	117.40
−17	−15	7	38.83	84.25
−23	−15	7	17.30	101.29
−24	−14	2	−6.34	107.63
−18	−13	1	33.68	101.82
−24	−13	1	94.82	115.95
−23	−10	−1	65.37	134.31
−20	−5	−3	192.68	102.88
5	1	4	618.05	91.52
6	4	5	163.76	96.40
6	5	4	336.49	98.52
−30	6	−6	44.50	120.84
−22	8	−6	64.71	100.76
6	12	3	43.32	103.27
−31	12	−7	155.44	122.95
−1	13	−2	223.32	55.73
1	17	−1	180.66	91.65
6	18	6	29.98	137.87
−1	27	−1	−7.66	86.90
0	34	2	8.32	113.70
−3	36	0	−41.34	109.74
−13	−15	5	31.96	85.71
−21	−15	3	22.98	102.35
−27	−15	6	29.71	119.38
−10	−14	4	154.25	77.39
−5	−10	2	363.70	75.80
−20	−10	−1	67.09	97.46
−26	−10	−1	−60.09	127.57
−9	−9	0	15.05	83.59
−22	−8	−2	27.47	119.12
−25	−8	−2	−5.28	130.34
1	−6	7	67.62	57.71
−21	−2	−4	92.97	94.16
−2	−1	11	−31.43	89.41
−2	4	12	−70.26	109.35
−27	6	−6	18.49	129.42
6	8	7	2.91	90.07
3	8	10	34.34	108.95
−27	13	−7	−50.58	115.16
−1	16	−2	510.42	85.84
6	17	4	−5.68	98.25

1	25	0	−18.49	90.86
−1	34	1	73.16	111.72
−14	−15	4	55.47	90.07
−18	−15	3	56.13	121.63
−28	−15	5	56.13	119.91
−14	−11	0	−18.49	86.10
−28	−8	−2	79.24	114.89
−8	−7	11	−22.71	60.88
2	−3	8	0.00	72.77
−7	−2	−2	719.86	86.37
−28	1	−5	0.00	128.63
5	6	8	−24.04	104.99
−23	7	−6	8.32	108.03
4	9	1	233.09	69.33
6	14	7	−37.24	133.12
5	15	2	70.52	94.16
4	15	1	130.87	103.93
−15	18	−6	44.77	59.30
5	20	3	72.77	99.05
5	22	4	97.59	121.89
−2	36	1	−10.96	110.14
−24	−15	3	122.29	112.65
−15	−14	2	1.19	88.08
−27	−14	2	16.38	115.69
−7	−13	6	−9.24	52.30
−11	−13	2	115.16	80.29
−27	−13	1	−33.68	113.18
−19	−8	−2	86.24	93.10
−15	−7	−2	−17.70	92.18
−8	−3	−2	496.42	84.39
−25	1	−5	104.20	131.27
4	2	8	0.00	85.97
−21	2	−5	0.00	90.46
2	4	10	26.41	102.61
4	7	9	26.28	116.35
−4	7	13	113.84	123.48
−1	8	12	1.98	116.87
5	9	2	73.43	80.03
−28	12	−7	−59.30	113.57
1	14	−1	214.07	73.82
2	20	0	47.01	79.37
3	25	2	−86.63	105.12
3	27	3	−39.22	143.81
−26	−15	7	0.00	108.69
−9	−14	5	51.11	66.56
−18	−12	0	−38.56	93.37
−17	−10	−1	61.28	94.95
−24	−6	−3	66.95	133.25
−25	−3	−4	15.98	132.46
−28	−3	−4	77.26	121.10
−6	−1	−2	84.65	54.01
0	4	11	−12.41	106.57
3	11	10	22.98	111.59
−32	11	−7	−89.93	116.87

5	12	2	80.56	99.05
4	12	1	444.39	94.03
−16	14	−6	−4.89	71.58
6	15	6	0.00	105.91
5	15	8	−28.92	128.10
6	16	5	22.58	98.65
0	22	−1	0.00	79.76
2	25	1	120.04	93.63
−11	−14	3	91.91	79.76
−15	−13	1	25.09	89.67
−27	−12	0	0.00	114.76
−13	−9	−1	107.23	94.82
−27	−6	−3	10.30	123.08
2	−4	3	648.42	63.26
−9	−4	−2	67.75	73.56
1	−2	9	134.44	110.80
−3	−2	11	36.71	85.05
4	0	7	92.05	80.56
3	3	9	28.39	122.02
−31	5	−6	−25.22	113.18
−24	6	−6	−0.13	135.63
6	8	4	−1.32	99.71
5	9	8	−37.24	123.21
6	14	4	6.07	94.69
5	19	7	13.60	134.97
5	21	5	0.00	127.70
−2	29	−1	−18.49	102.74
−23	−16	5	−14.39	111.06
−27	−15	3	−30.77	116.35
−11	−12	1	218.43	86.90
0	−7	7	411.90	64.05
−3	−7	1	1116.44	105.38
−21	−6	−3	14.92	95.61
−17	−5	−3	181.72	99.57
−10	−1	−3	375.19	90.20
−9	0	−3	418.77	81.61
5	2	6	42.79	76.73
−3	3	12	9.90	104.46
−28	5	−6	81.88	121.76
6	7	5	142.49	91.39
6	9	6	153.85	95.88
4	10	9	103.14	115.42
6	11	4	0.00	88.88
−29	11	−7	101.69	110.40
6	12	6	2.91	92.57
5	12	8	−47.67	126.91
−7	12	−4	140.51	38.96
4	13	9	0.00	109.74
4	20	2	−12.28	84.52
4	24	4	−248.80	145.14
2	29	3	0.00	128.63
−17	−16	5	−8.06	89.93
−19	−16	6	68.94	92.44
−21	−16	4	0.00	99.97

−22	−16	6	−55.86	97.99
−14	−15	7	−22.85	75.54
−10	−10	0	107.23	97.33
−4	−8	1	277.59	57.84
−22	−3	−4	96.93	102.22
−11	−2	−3	42.52	95.35
−8	1	−3	172.60	61.80
−22	1	−5	−59.16	103.54
5	4	7	134.57	88.08
1	11	11	117.53	121.10
6	13	5	36.58	94.03
5	20	6	−108.69	140.64
3	20	1	0.00	85.97
−3	26	−2	128.76	82.01
2	27	2	76.33	134.17
0	27	0	−46.35	94.69
−18	−16	4	−5.81	97.46
−12	−15	6	−12.28	78.58
−22	−15	8	−42.92	97.59
−5	−12	4	35.79	53.09
−6	−12	3	−100.37	75.27
−24	−11	−1	0.00	127.17
−23	−9	−2	−59.69	134.04
−2	−6	1	1498.89	132.19
−7	−6	11	−10.04	66.95
−6	−5	−1	121.36	57.71
−10	−5	−2	229.26	87.82
1	−4	8	47.67	71.05
−4	−3	11	−6.07	81.88
−18	−2	−4	122.55	94.69
−5	0	−2	60.35	50.71
−29	0	−5	24.70	112.12
5	5	3	411.37	80.29
−15	5	−5	−12.41	77.65
−5	6	13	−35.79	123.08
6	10	5	42.92	89.93
−33	10	−7	−28.79	114.23
−11	12	−5	50.18	57.84
5	17	3	107.89	95.61
5	19	4	−13.34	92.57
4	22	3	88.61	104.20
−3	31	−1	−35.52	125.46
−19	−15	2	−66.16	122.95
−19	−15	8	11.09	85.84
−22	−15	2	−23.77	105.91
−19	−14	1	47.28	130.87
−22	−14	1	10.83	129.16
−21	−11	−1	−20.34	117.01
−26	−9	−2	0.00	116.61
−16	−8	−2	−27.60	96.14
−5	−4	−1	617.78	78.97
3	−3	6	722.24	91.39
−12	−3	−3	267.42	100.37
−1	−2	10	−39.49	81.61

−26	0	−5	71.45	133.38
−16	4	−5	71.05	82.01
−25	5	−6	0.00	130.08
−14	6	−5	14.66	73.03
−2	7	12	3.17	110.67
−30	10	−7	0.00	107.63
−24	13	−7	−58.77	120.04
5	16	7	148.57	140.64
−4	20	−3	247.88	62.73
−5	25	−3	21.13	70.12
1	27	1	−52.16	107.50
1	31	3	−44.11	117.80
−25	−16	6	0.00	103.40
−26	−16	5	174.85	118.06
−25	−15	2	104.06	108.55
−25	−14	1	−0.79	107.63
−15	−12	0	72.63	91.39
−27	−11	−1	−31.69	111.33
−5	−9	1	−2.51	57.84
−20	−9	−2	92.97	94.69
−1	−8	7	51.64	43.98
−7	−6	−1	6.07	61.01
−6	−5	11	0.00	72.24
−5	−4	11	0.00	73.82
4	1	3	237.31	57.58
−17	3	−5	198.36	92.18
−29	4	−6	−29.32	117.40
2	7	10	14.79	100.63
−8	8	−4	46.22	53.75
−25	12	−7	76.07	122.68
−23	14	−7	0.00	120.44
4	21	7	−55.47	117.01
4	23	5	0.00	136.55
3	26	4	21.26	139.72
1	29	2	143.81	138.93
−4	33	−1	−38.43	122.68
−16	−16	6	−82.41	87.95
−18	−11	−1	−8.98	90.59
−14	−10	−1	192.41	89.80
−25	−7	−3	4.09	131.00
−11	−6	−2	128.50	101.82
−18	−6	−3	143.55	97.73
−26	−4	−4	50.32	129.16
−29	−4	−4	111.72	126.51
2	1	1	513.19	56.26
−4	2	12	−52.16	97.33
1	3	10	−28.00	98.25
−1	3	11	34.47	102.22
4	5	8	65.37	99.84
0	7	11	1.98	111.46
−13	7	−5	91.12	74.88
−19	8	−6	61.94	82.93
4	17	8	0.00	100.23
2	17	0	82.80	92.31

−2	21	−2	70.52	68.94
−1	29	0	31.69	111.99
−19	−16	3	118.06	110.14
−22	−16	3	−97.06	104.06
−27	−16	4	254.75	118.46
−11	−15	4	12.28	73.69
−12	−14	2	72.77	84.52
−13	−13	9	0.40	55.07
−25	−13	0	−1.06	113.57
−2	−9	7	88.35	35.26
−8	−7	−1	4.09	70.78
−28	−7	−3	156.36	117.80
−13	−4	−3	182.11	101.69
−23	−4	−4	82.54	119.65
0	−3	9	117.93	100.50
−4	−3	−1	2414.47	209.98
−19	−3	−4	−74.22	103.14
−23	0	−5	23.37	110.93
3	1	8	0.00	79.76
−18	2	−5	68.54	90.46
5	3	5	131.66	90.33
3	5	1	110.54	55.86
3	6	9	3.30	119.12
5	7	7	139.06	94.95
−20	7	−6	−32.75	87.42
−18	9	−6	86.50	82.01
2	10	10	−20.60	103.14
−26	11	−7	185.28	116.35
5	13	7	−62.33	119.65
2	13	10	0.00	109.48
5	14	3	100.50	95.35
−15	15	−6	12.41	65.77
5	17	6	24.83	120.31
4	17	2	101.29	88.08
5	18	5	120.31	102.08
4	22	6	37.24	128.50
1	22	0	97.86	86.10
−8	26	−4	57.58	60.75
0	29	1	0.00	128.76
−5	35	−1	70.12	112.25
−15	−16	4	0.00	88.74
−21	−16	7	−0.53	91.65
−16	−15	2	18.75	100.50
−16	−14	1	24.43	88.61
−11	−11	0	38.96	96.40
−22	−7	−3	0.00	114.36
2	2	9	0.00	117.93
5	4	4	416.12	90.59
−26	4	−6	39.75	122.55
−21	6	−6	54.01	91.91
−31	9	−7	−76.20	113.31
−17	10	−6	276.67	82.67
−7	21	−4	309.82	54.01
3	24	3	117.40	117.01

−1	24	−1	−85.97	83.99
2	28	4	97.73	118.46
−4	28	−2	125.59	87.56
0	31	2	75.41	131.00
−2	31	0	−11.36	129.82
−14	−16	5	39.62	86.50
−24	−16	7	0.00	96.54
−25	−16	3	13.60	110.40
−28	−16	6	−0.53	107.23
−12	−15	3	−33.81	78.18
−6	−10	1	26.81	65.11
−2	−3	10	80.43	78.71
−27	−1	−5	0.00	131.14
4	0	4	1197.13	120.97
−12	1	−4	99.71	96.27
4	5	2	1339.76	128.36
5	8	3	314.04	94.56
2	8	0	−9.11	49.52
−27	10	−7	103.01	115.42
4	14	8	−8.72	118.33
3	15	9	111.20	106.31
5	16	4	0.00	95.08
0	19	−1	257.65	88.08
2	22	1	97.86	88.48
3	25	5	0.00	124.01
−1	33	2	−5.28	110.27
−18	−16	7	52.03	84.39
−12	−14	8	52.30	60.75
−17	−14	9	43.84	76.86
−8	−9	10	15.45	39.62
−17	−9	−2	0.00	98.25
−12	−7	−2	310.61	111.46
0	−5	8	12.15	66.03
3	−1	7	93.63	75.41
−5	1	12	12.55	96.01
−19	1	−5	54.54	98.12
−11	2	−4	606.56	103.27
−30	3	−6	99.97	106.04
−22	5	−6	10.30	105.12
4	8	8	−71.97	111.59
3	9	9	−14.66	112.65
5	11	3	0.00	99.05
4	21	4	−66.82	98.12
−1	31	1	43.84	139.72
−3	33	0	−81.22	122.29
−2	35	2	56.39	109.48
−16	−16	3	−5.94	116.08
−10	−15	5	−10.30	70.65
−16	−15	8	−50.32	77.26
−7	−13	3	208.66	68.41
−22	−12	−1	106.84	128.89
−25	−12	−1	54.28	112.25
−24	−10	−2	18.62	121.23
−27	−10	−2	−129.02	109.35

−9	−8	−1	104.99	86.10
−14	−5	−3	150.29	92.31
−24	−1	−5	−49.65	131.80
−3	6	12	25.36	104.99
−12	8	−5	222.52	74.35
−28	9	−7	40.28	105.52
4	11	8	0.00	126.25
3	12	9	−39.22	111.86
5	14	6	−35.26	99.18
4	18	7	−0.79	141.04
4	19	3	109.35	97.59
3	24	6	−53.75	125.46
−2	33	1	−131.66	120.18
−4	35	0	−50.45	110.67
−5	37	0	34.86	104.06
−6	−13	4	114.36	60.22
−16	−13	0	94.16	90.20
−21	−10	−2	−29.85	121.50
−19	−7	−3	7.13	92.44
−1	−5	1	1059.26	96.93
−27	−5	−4	−47.81	118.46
−20	−4	−4	44.11	91.52
3	1	2	95.22	47.81
−2	2	11	45.56	100.37
4	3	7	232.82	88.88
−27	3	−6	−40.81	114.23
−32	8	−7	0.00	112.12
−16	11	−6	11.89	77.26
4	14	2	3.70	100.10
5	15	5	121.36	94.29
−3	35	1	−15.05	104.46
−4	37	1	65.24	107.89
−21	−17	5	0.00	93.10
−23	−16	2	−23.90	102.88
−20	−15	1	−106.57	124.40
−23	−15	1	−32.22	104.72
−8	−14	6	30.77	58.77
−15	−11	−1	30.24	87.16
−26	−8	−3	135.36	119.65
−24	−5	−4	21.53	136.15
−1	−4	9	56.65	95.88
−3	−4	10	58.90	75.80
−20	0	−5	−42.00	89.67
4	1	6	83.73	73.95
0	2	10	42.79	96.27
−10	3	−4	1382.94	144.21
−23	4	−6	62.07	118.46
1	6	10	−10.04	98.91
5	8	6	0.00	87.56
5	11	6	−9.90	94.29
2	11	0	69.33	60.75
5	13	4	81.09	92.71
4	19	6	−17.70	125.19
4	20	5	−12.94	108.16

2	24	2	26.68	93.76
2	27	5	0.00	101.16
−6	27	−3	61.28	74.22
−5	30	−2	−16.11	99.97
−19	−17	4	35.79	93.24
−22	−17	4	−19.15	107.37
−24	−17	5	−54.54	108.29
−20	−16	2	13.47	106.97
−26	−15	1	21.79	113.57
−12	−12	0	0.00	80.03
−19	−12	−1	131.27	103.54
−7	−11	1	507.91	96.01
−10	−9	−1	522.70	102.48
−13	−8	−2	−102.35	99.71
−23	−8	−3	−21.79	134.70
−28	−2	−5	−56.39	112.38
−4	1	−2	136.29	47.15
5	6	5	107.89	93.50
−1	6	11	−16.90	106.31
5	7	4	233.22	90.46
−29	8	−7	−2.38	106.84
3	14	1	873.85	118.99
0	24	0	206.28	97.46
−2	26	−1	109.21	83.86
−20	−17	6	42.79	88.61
−23	−17	6	26.68	97.33
−25	−17	4	0.00	111.20
−26	−16	2	29.32	106.71
−13	−15	2	45.30	83.86
−23	−14	0	68.94	112.91
−8	−13	2	15.32	77.12
−3	−11	5	92.44	34.60
−15	−6	−3	27.07	85.44
2	−4	6	126.51	59.69
−3	−2	−1	1265.01	125.59
−6	0	12	1.72	88.88
4	8	2	160.72	70.78
5	10	4	49.52	112.78
4	11	2	166.66	85.58
5	12	5	203.77	95.74
4	15	7	36.45	127.04
−4	17	−3	163.36	60.35
3	23	4	125.46	110.93
1	24	1	103.67	88.08
1	28	3	0.00	137.74
−6	32	−2	19.55	122.82
−17	−15	1	54.94	106.44
−13	−14	1	32.09	85.71
−26	−14	0	34.20	104.33
−18	−10	−2	14.66	95.35
−16	−3	−4	235.60	87.42
−25	−2	−5	−87.29	128.63
−21	−1	−5	−38.30	95.35
2	0	8	18.09	76.60

−28	2	−6	−90.59	114.23
−24	3	−6	195.05	128.50
3	4	8	314.97	96.54
2	5	9	18.49	119.25
3	8	1	362.38	65.90
5	9	5	6.21	82.27
1	9	10	−67.22	101.69
−3	9	12	−84.52	112.78
−24	10	−7	56.13	123.74
−23	11	−7	59.69	118.33
1	12	10	6.47	104.06
3	19	2	41.47	93.24
−20	−17	3	59.82	102.74
−23	−17	3	−69.20	106.31
−27	−17	5	48.47	118.19
−12	−16	4	51.37	79.24
−15	−16	7	34.60	76.73
−17	−16	2	51.77	123.48
−20	−8	−3	75.14	100.10
−1	−6	8	54.67	62.99
−4	−5	10	3.57	67.48
−21	−5	−4	22.71	99.71
1	1	9	70.12	111.99
−4	5	12	16.90	101.03
0	7	−1	924.56	86.76
3	11	1	1471.03	145.66
−22	12	−7	−23.90	117.53
4	16	3	−17.83	86.50
3	16	8	0.00	120.84
−10	16	−5	8.06	35.39
4	18	4	−56.92	93.76
−2	18	−2	183.04	75.41
1	19	0	49.26	84.92
3	20	7	4.23	116.74
3	21	3	157.94	99.97
−3	23	−2	62.73	72.24
1	26	2	0.00	98.52
−16	−17	4	44.90	90.86
−26	−17	6	−53.09	105.12
−8	−14	3	0.00	67.22
−25	−11	−2	−37.24	115.42
3	−2	5	12.41	69.07
−7	−1	12	−13.60	87.82
−3	1	11	117.14	97.20
−9	4	−4	425.10	78.97
4	6	7	−3.43	86.10
−30	7	−7	0.00	112.12
−25	9	−7	94.95	116.61
−15	12	−6	109.48	69.99
0	16	−1	384.96	86.37
2	19	1	0.00	71.84
3	22	5	−37.51	129.82
0	30	3	−105.25	121.63
−17	−17	6	−38.17	84.39

−26	−17	3	−3.04	103.54
−13	−16	3	119.78	80.29
−23	−13	−1	0.00	126.38
−26	−13	−1	69.60	101.42
−8	−12	1	187.79	85.97
−16	−12	−1	−50.98	93.50
−22	−11	−2	166.93	134.31
−11	−10	−1	471.19	110.27
−14	−9	−2	179.74	87.29
−16	−7	−3	124.14	95.22
−25	−6	−4	10.70	123.08
−2	−5	9	−9.64	79.63
−25	2	−6	115.95	128.10
−26	8	−7	82.01	112.25
4	9	7	−51.90	89.93
−1	12	11	−20.07	111.46
−21	13	−7	40.94	111.72
2	14	9	−42.79	106.84
4	16	6	19.55	95.35
4	17	5	186.47	94.16
3	21	6	0.13	130.34
2	25	4	−63.92	128.23
−1	26	0	−50.84	87.16
−3	28	−1	74.61	85.44
−7	29	−3	42.52	79.24
−1	32	3	−170.36	120.18
−15	−17	5	−142.49	88.74
−17	−17	3	11.89	108.29
−20	−16	8	−61.01	86.76
−7	−14	4	220.54	66.43
−13	−13	0	67.88	91.52
−24	−9	−3	−49.79	120.44
−7	−8	10	−48.07	51.37
−5	−6	10	11.75	62.99
−17	−4	−4	133.51	91.91
2	−2	7	194.79	73.03
−22	−2	−5	−28.26	111.33
4	4	3	362.51	69.46
2	8	9	−11.89	111.06
−11	9	−5	−36.58	65.50
2	11	9	−14.13	115.69
3	13	8	−17.30	126.65
−14	19	−6	37.64	46.35
−1	21	−1	127.04	74.88
0	26	1	246.29	90.73
0	28	2	−4.36	128.36
−11	−15	7	0.00	63.39
−20	−13	−1	0.92	126.51
−4	−12	5	97.86	43.18
−6	−9	0	298.46	73.82
−5	−8	0	713.92	84.65
−6	−7	10	0.00	57.18
1	−6	4	610.65	61.28
−26	−3	−5	46.22	112.38

-8	-2	12	-0.40	71.58
-29	1	-6	17.17	100.37
4	2	5	187.79	82.93
0	5	10	50.58	100.37
-2	5	11	72.77	106.04
-19	5	-6	0.00	81.88
-18	6	-6	57.18	82.27
-31	6	-7	-42.26	108.03
3	7	8	34.07	95.88
-7	7	13	36.45	109.21
3	10	8	13.47	113.18
2	22	7	90.46	119.25
-5	22	-3	92.71	61.28
-22	-17	7	-86.50	93.24
-11	-16	5	-18.36	77.78
-21	-16	1	3.04	111.06
-24	-16	1	22.45	103.54
-9	-14	2	120.70	74.22
-19	-11	-2	19.15	93.24
-4	-7	0	1046.06	103.40
-22	-6	-4	61.67	115.03
-16	1	-5	-33.81	85.58
-15	2	-5	139.85	81.09
-14	3	-5	178.28	78.44
4	4	6	63.26	67.48
-20	4	-6	-36.98	88.08
-27	7	-7	-14.66	101.82
4	13	3	269.80	103.67
-20	14	-7	-64.71	94.56
-14	16	-6	55.47	56.39
3	17	7	-14.00	124.14
2	23	3	-40.01	94.29
2	24	5	129.95	140.38
1	27	4	151.08	132.19
-4	30	-1	58.50	100.63
-21	-17	2	-36.45	102.48
-24	-17	2	85.97	101.16
-14	-16	2	15.32	83.99
-14	-15	1	28.66	84.12
-24	-15	0	-71.84	104.86
-12	-11	-1	131.66	87.42
-21	-9	-3	-25.36	116.21
-17	-8	-3	0.00	92.57
-18	-5	-4	46.75	87.69
-17	0	-5	221.73	92.05
-26	1	-6	0.00	118.33
3	2	7	116.74	80.29
-5	4	12	0.00	97.06
-17	7	-6	220.54	84.39
-4	8	12	74.48	106.44
4	13	6	10.17	94.95
4	15	4	-25.62	91.25
3	20	4	16.77	96.54
2	23	6	-20.34	131.53

−1	28	1	0.00	95.48
−2	28	0	19.81	89.80
−1	30	2	47.15	130.61
−22	−18	5	35.92	99.44
−23	−18	4	−149.49	109.48
−19	−17	7	77.26	84.92
−21	−15	0	1.85	114.89
−9	−13	1	182.11	85.18
−7	−10	0	847.57	113.44
−15	−10	−2	24.30	91.78
−2	−7	8	17.96	59.16
−23	−3	−5	57.18	124.14
−4	0	11	87.95	85.84
4	3	4	1175.08	125.06
−21	3	−6	0.00	103.67
−13	4	−5	32.35	71.31
−28	6	−7	6.87	101.82
0	13	−1	263.99	61.80
0	14	10	26.02	110.40
−4	14	−3	688.17	71.05
−2	32	2	48.86	118.72
−5	32	−1	78.58	122.42
−20	−18	4	20.34	99.18
−25	−18	5	0.00	104.99
−18	−17	2	112.52	111.86
−18	−16	1	−136.55	130.61
−9	−15	3	45.16	72.77
−9	−15	6	32.35	61.41
−17	−13	−1	62.20	90.07
−26	−7	−4	−38.96	107.89
−18	−1	−5	118.72	89.27
3	0	3	1332.10	122.42
3	0	6	563.24	85.58
0	0	9	214.20	111.06
1	4	9	−72.90	114.50
4	7	3	1537.59	152.40
4	7	6	264.65	83.99
0	8	10	18.09	98.25
−16	8	−6	0.00	77.39
4	10	3	249.86	95.22
4	10	6	380.07	94.56
−10	13	−5	16.90	43.71
4	14	5	255.80	99.84
3	18	3	192.28	91.78
3	18	6	3.04	106.18
−4	25	−2	−3.04	76.46
1	26	5	−10.70	125.85
−3	34	2	−34.47	112.12
−6	34	−1	−74.88	116.35
−4	36	2	77.12	102.61
−7	36	−1	10.04	104.99
−19	−18	5	−26.02	88.74
−26	−18	4	55.20	108.16
−23	−12	−2	−15.19	115.29

-8	-11	0	260.42	93.10
-3	-6	0	441.61	62.73
3	-1	4	101.55	53.48
1	-1	8	-2.11	70.52
-22	2	-6	101.69	116.21
2	3	8	41.73	82.93
1	4	0	976.72	91.25
-2	8	11	71.84	99.05
0	11	10	30.64	100.89
1	16	0	80.43	85.05
1	16	9	0.00	106.18
3	19	5	-2.38	98.78
1	21	1	121.89	85.71
0	21	0	15.45	83.99
1	25	6	-41.86	112.65
-2	30	1	33.28	132.19
-3	30	0	108.03	110.93
-21	-18	3	-6.47	99.18
-24	-18	3	10.04	106.44
-13	-17	4	14.00	80.29
-14	-17	3	-42.52	88.08
-17	-16	8	0.00	72.77
-14	-14	0	3.83	93.90
-24	-14	-1	-36.58	102.35
-25	-10	-3	0.00	111.20
-23	-7	-4	221.73	138.66
-19	-6	-4	69.20	95.61
-11	-5	-3	140.78	97.59
-10	-4	-3	900.79	119.25
-9	-3	-3	453.63	89.01
-27	0	-6	-0.92	110.54
-29	5	-7	136.95	102.88
-2	11	11	-44.90	109.21
4	12	4	259.24	92.31
3	14	7	52.96	108.29
-2	15	-2	450.72	71.84
2	16	1	137.21	86.10
-3	32	1	9.90	130.87
-4	32	0	-54.81	125.85
-6	38	1	95.08	104.99
-7	38	0	-19.55	102.08
-17	-18	4	20.87	89.27
-21	-18	6	56.52	90.73
-24	-18	6	-42.92	93.90
-14	-17	6	9.51	78.31
-8	-15	4	0.00	65.37
-10	-15	2	74.22	76.33
-13	-15	8	0.00	62.20
-21	-14	-1	95.61	126.91
-5	-13	5	6.74	48.07
-13	-12	-1	30.90	91.91
-20	-12	-2	17.70	120.70
-18	-9	-3	63.13	94.03
-8	-6	-2	203.90	73.16

1	−5	6	57.71	52.16
−19	−2	−5	40.81	90.33
−23	1	−6	90.20	130.74
4	5	5	252.10	96.93
−8	5	−4	124.67	63.13
−12	5	−5	−5.81	73.69
−23	8	−7	−161.77	122.68
−7	9	−4	57.45	48.07
−22	9	−7	108.16	116.48
4	11	5	57.45	82.01
2	15	8	−81.61	125.99
−19	15	−7	−17.17	72.11
2	19	7	−72.24	127.17
2	22	4	−33.68	93.63
0	28	5	0.00	114.23
−4	34	1	81.35	111.33
−5	34	0	0.00	119.91
−5	36	1	−52.43	104.46
−6	36	0	−5.68	102.74
−16	−11	−2	12.28	87.16
−22	−10	−3	−34.20	125.99
−9	−7	−2	248.67	90.59
−12	−6	−3	33.94	96.27
−7	−5	−2	250.12	66.29
−6	3	12	15.32	90.86
−1	4	10	−40.81	95.08
−3	4	11	24.83	98.25
3	5	7	19.68	78.97
4	6	4	229.79	90.46
−24	7	−7	8.06	116.08
−21	10	−7	62.60	113.31
1	13	9	39.35	104.59
−14	13	−6	108.29	63.79
−2	23	−1	−0.13	78.18
−18	−18	3	11.89	96.54
−27	−18	6	135.10	110.01
−22	−17	1	27.47	100.63
−25	−17	1	7.00	101.03
−15	−16	1	−103.80	100.10
−14	−14	9	5.81	55.20
−9	−12	0	199.02	97.46
−10	−8	−2	79.37	93.37
−4	−7	9	−30.64	59.16
−8	−2	−3	41.60	67.22
−5	−1	11	100.63	79.76
3	4	2	2021.19	178.15
−25	6	−7	−54.81	107.89
1	7	9	0.00	111.33
−5	7	12	−22.32	105.78
4	8	5	120.97	85.31
4	9	4	122.42	99.71
−15	9	−6	261.88	76.99
1	10	9	28.53	105.38
3	13	2	163.10	88.88

−18	−18	6	−68.01	83.59
−15	−17	2	117.67	107.63
−16	−17	7	13.73	76.07
−22	−16	0	51.24	103.14
−3	−8	8	0.00	56.79
−13	−7	−3	222.13	95.88
−20	−7	−4	23.77	98.39
1	−3	7	54.41	62.46
−13	−3	−4	159.66	91.12
−20	−3	−5	73.03	93.24
−24	0	−6	0.00	120.84
−30	4	−7	90.86	103.67
2	6	8	−31.96	90.86
3	8	7	91.91	88.88
2	12	8	−9.24	122.42
3	15	6	0.00	86.50
3	17	4	108.55	94.42
2	20	6	11.23	124.67
2	21	5	14.26	105.91
−5	27	−2	−26.02	73.16
−22	−18	2	17.43	98.52
−25	−18	2	−47.54	99.44
−12	−17	5	4.89	77.12
−19	−17	1	13.47	113.04
−10	−16	3	108.69	73.69
−18	−14	−1	0.00	105.65
−24	−13	−2	−11.09	103.54
−24	−8	−4	0.00	120.18
−6	−4	−2	938.82	101.95
−14	−4	−4	251.71	97.99
−28	−1	−6	−0.13	101.42
−26	5	−7	12.55	104.20
2	9	8	56.13	103.14
−20	11	−7	−29.32	102.74
2	18	2	48.73	81.35
2	20	3	253.69	88.22
−3	20	−2	141.31	68.14
0	23	1	105.65	86.63
−8	23	−4	10.30	48.86
−1	29	3	−94.29	137.61
−14	−13	−1	59.03	88.88
−19	−10	−3	133.25	106.04
−11	−9	−2	0.13	94.29
−5	10	12	55.47	106.97
3	15	3	−0.40	92.18
3	16	5	107.76	90.73
−1	18	−1	95.35	80.43
−7	18	−4	147.12	34.34
1	21	7	0.00	111.06
−1	23	0	10.70	81.75
1	24	4	40.81	101.95
−6	24	−3	0.00	61.80
0	25	2	−22.85	94.16
−2	31	3	−34.86	116.08

−19	−18	2	−7.66	100.23
−19	−16	0	−21.13	118.72
−6	−14	5	23.51	50.84
−10	−13	0	51.90	83.86
−17	−12	−2	35.00	88.35
−5	−8	9	24.30	50.45
−14	−8	−3	−4.09	87.95
−15	−5	−4	81.35	87.82
−1	−1	9	79.10	101.55
−7	−1	−3	174.32	69.33
−10	0	−4	1805.54	177.89
−5	4	−3	308.10	48.47
−27	4	−7	78.44	106.04
−1	7	10	−29.19	95.22
−3	7	11	−20.21	102.35
3	10	2	291.72	76.86
2	13	1	369.51	87.56
−1	13	10	−23.64	102.48
2	16	7	4.09	124.67
1	17	8	0.00	107.76
−3	33	3	4.36	113.84
−21	−19	4	85.18	102.88
−23	−19	5	15.98	99.97
−24	−19	4	109.61	103.93
−23	−18	7	−14.92	92.31
−9	−16	4	35.52	66.95
−11	−16	2	64.71	78.58
−12	−16	7	2.64	63.39
−11	−15	1	88.74	80.56
−22	−15	−1	24.70	103.93
−21	−13	−2	38.03	122.95
−23	−11	−3	18.09	110.01
−12	−10	−2	27.34	81.75
2	−3	5	111.59	61.54
−2	−1	−1	445.71	52.82
−25	−1	−6	10.70	115.42
2	1	7	−38.30	74.61
−7	2	12	137.74	89.27
3	3	6	297.93	78.97
0	3	9	138.80	111.46
2	4	1	859.72	94.03
−11	6	−5	23.24	76.99
3	7	2	3867.01	327.91
−1	10	10	−0.40	93.24
−10	10	−5	95.35	59.16
−3	13	11	−2.25	101.95
1	22	6	0.00	126.51
1	23	5	−43.84	118.46
−3	25	−1	25.49	77.39
−6	29	−2	50.45	79.37
−14	−18	4	0.00	84.92
−15	−18	3	−17.83	110.01
−10	−16	6	−13.60	67.22
−7	−10	9	38.17	32.09

-21	-8	-4	0.00	125.06
-16	-6	-4	172.08	92.44
1	-5	3	280.63	33.02
0	-2	8	-37.24	68.54
-6	-2	11	-20.47	76.86
-3	-1	10	33.41	78.44
-19	2	-6	18.88	88.08
-4	3	11	-2.38	94.29
-18	3	-6	43.18	81.75
-17	4	-6	44.50	82.01
3	12	6	194.53	93.63
-2	12	-2	376.11	52.30
-19	12	-7	90.86	92.57
0	15	9	-19.02	100.23
0	26	4	79.63	125.85
-1	27	2	0.00	95.22
-20	-19	5	26.81	86.76
-22	-19	3	0.13	101.55
-26	-19	5	-5.55	109.61
-20	-18	7	65.50	82.27
-16	-17	1	47.41	112.25
-6	-9	9	12.68	42.92
-15	-9	-3	32.75	92.97
-2	-5	0	-14.53	51.77
-20	1	-6	162.30	101.42
1	2	8	57.45	79.90
-28	3	-7	104.20	101.82
-6	6	12	7.79	97.20
0	23	7	65.77	106.57
-1	25	1	103.80	82.93
-7	31	-2	14.00	101.95
-18	-19	4	-0.79	84.92
-19	-15	-1	23.64	131.80
-11	-14	0	62.46	78.18
-15	-14	-1	0.00	87.82
-20	-11	-3	50.32	122.29
-8	-10	-1	0.00	80.16
-4	-9	8	-3.83	45.83
-7	-9	-1	353.40	82.27
-25	-9	-4	-13.87	103.80
-6	-8	-1	248.41	68.80
-26	-6	-5	57.45	103.54
-5	-3	-2	738.35	83.20
-3	2	-2	426.03	53.35
-2	3	10	130.08	92.84
-16	5	-6	152.53	83.33
3	9	6	283.27	88.88
-2	9	-2	725.15	66.16
-14	10	-6	276.27	75.41
3	13	5	-14.66	84.39
3	14	4	296.21	90.33
2	17	6	-19.68	91.25
0	18	0	343.49	95.61
2	19	4	72.63	89.67

1	22	3	112.65	88.08
−2	25	0	127.44	82.41
−1	28	4	57.31	143.95
−2	29	2	0.92	122.95
−8	33	−2	−12.15	115.42
−19	−19	3	−43.45	93.90
−15	−18	6	86.37	72.90
−16	−18	2	−65.50	116.21
−23	−18	1	−31.30	100.23
−11	−17	3	27.47	73.43
−23	−17	0	−10.56	100.10
−18	−13	−2	−56.92	111.72
−13	−11	−2	−16.64	88.08
−17	−7	−4	0.00	89.54
−16	−2	−5	34.20	88.88
−26	−2	−6	−29.32	99.84
2	−1	6	142.10	68.14
−15	−1	−5	31.03	83.73
−14	0	−5	314.70	87.16
−21	0	−6	11.36	112.91
−9	1	−4	669.02	94.69
−9	8	13	0.13	104.33
2	10	1	803.20	92.84
1	10	0	569.32	73.16
3	12	3	84.25	92.84
0	12	9	−42.66	112.12
1	14	8	47.94	119.38
2	18	5	−14.39	88.22
0	24	6	65.24	124.14
0	25	5	124.80	126.78
−4	27	−1	−6.47	74.48
−22	−19	6	100.23	89.41
−25	−19	6	−37.64	94.16
−20	−18	1	−22.05	96.93
−9	−11	−1	243.39	97.33
−17	−3	−5	5.02	83.46
2	0	2	110.80	43.58
−8	1	12	43.71	86.90
3	3	3	668.76	82.67
−23	5	−7	112.38	119.25
0	6	9	−48.47	112.91
−22	6	−7	−16.90	121.10
1	7	0	777.05	81.09
−21	7	−7	147.64	113.18
−6	9	12	104.46	106.04
1	18	7	35.39	122.82
1	20	2	61.94	81.88
−2	27	1	87.03	87.42
−3	31	2	54.54	124.80
−4	33	2	−78.44	115.95
−5	35	2	−50.58	105.91
−17	−19	5	0.00	81.22
−23	−19	2	0.53	99.57
−12	−17	2	14.79	76.73

-20	-17	0	0.00	103.93
-7	-15	5	-13.73	54.81
-22	-14	-2	57.31	109.48
-24	-12	-3	0.00	98.39
-16	-10	-3	30.64	88.61
-22	-9	-4	0.00	122.42
-5	-7	-1	566.28	76.20
-7	-3	11	31.30	72.63
-22	-1	-6	0.00	127.97
-13	1	-5	114.76	78.05
3	2	4	461.29	77.78
2	4	7	50.32	80.43
-24	4	-7	-63.13	110.80
2	7	1	1209.81	116.21
0	9	9	50.32	104.20
2	15	2	47.81	89.67
-2	15	10	-47.67	99.05
2	17	3	-16.24	83.99
-5	19	-3	237.05	58.24
-7	26	-3	223.18	67.35
-1	27	5	-6.07	118.06
-8	39	1	-55.33	108.29
-20	-19	2	111.86	95.35
-13	-18	5	23.64	78.44
-18	-17	8	83.99	76.73
-18	-8	-4	6.74	95.35
-18	-4	-5	29.71	88.08
1	5	8	89.01	84.52
-4	6	11	-45.69	99.31
-15	6	-6	156.23	78.05
-20	8	-7	89.41	105.38
2	10	7	93.76	83.46
1	11	8	-87.95	112.91
-18	13	-7	19.02	75.80
-1	17	9	3.04	98.39
0	24	3	27.20	90.20
-1	26	6	13.47	108.42
-3	27	0	65.37	79.10
-3	29	1	28.92	103.14
-5	29	-1	-0.13	85.18
-19	-19	6	-2.51	82.54
-10	-17	4	30.51	71.97
-14	-16	8	34.07	62.20
-12	-15	0	-10.70	83.99
-10	-12	-1	146.98	89.80
-14	-12	-2	68.28	89.93
-21	-12	-3	-101.29	119.78
-27	-3	-6	-36.58	97.06
-4	-2	10	0.66	74.48
-23	-2	-6	62.60	119.65
-6	0	-3	389.32	66.43
-25	3	-7	8.32	103.80
3	4	5	1127.67	134.44
-2	6	10	26.81	96.54

2	7	7	155.04	83.73
3	9	3	385.75	89.54
3	10	5	52.69	78.71
3	11	4	289.08	112.12
1	21	4	71.31	91.78
−4	22	−2	−20.34	64.71
−2	29	5	102.35	106.44
−7	37	1	0.00	97.73
−9	39	0	−70.26	99.05
−16	−19	3	0.00	98.78
−17	−18	7	18.36	78.05
−16	−15	−1	−109.35	102.88
0	−6	6	36.58	45.56
0	−4	7	199.28	63.39
−2	−2	9	−1.98	99.05
−5	2	11	26.28	91.25
−12	2	−5	183.96	80.69
−7	5	12	11.49	94.82
3	6	3	137.61	67.22
1	8	8	0.00	93.50
−19	9	−7	70.12	97.20
−2	12	10	45.30	98.78
−4	12	11	112.52	105.91
2	14	6	−33.15	87.82
1	19	6	−20.21	104.33
−2	20	−1	137.08	77.52
−1	21	8	−34.86	99.71
−4	29	0	−46.22	85.97
−4	31	1	125.33	124.01
−6	31	−1	45.30	101.16
−5	33	1	−3.43	122.55
−7	33	−1	172.47	120.18
−6	35	1	−17.56	108.42
−8	35	−1	79.24	105.12
−8	37	0	−27.73	99.57
−22	−20	4	−70.26	99.84
−15	−19	4	−50.45	85.05
−17	−18	1	−26.15	112.65
−19	−14	−2	−7.66	118.46
−17	−11	−3	7.00	92.05
−5	−10	8	32.88	31.56
−23	−10	−4	5.94	107.10
−1	2	9	29.71	108.55
−26	2	−7	−126.12	103.01
−7	6	−4	162.83	55.07
3	7	5	318.93	101.69
−2	9	10	65.50	93.76
−1	15	−1	266.63	67.09
1	20	5	−19.15	94.42
0	20	7	76.73	141.17
−5	31	0	0.00	106.71
−7	35	0	0.00	110.54
−24	−20	5	176.83	108.95
−13	−17	1	35.52	82.93

−17	−17	0	−221.33	124.67
−5	−13	2	198.22	77.39
−11	−13	−1	91.25	86.76
−19	−9	−4	−22.71	107.23
−24	−7	−5	85.18	110.14
−4	−6	−1	1738.72	157.42
−8	−4	11	−56.65	69.20
−9	0	12	77.26	84.78
3	5	4	127.84	80.56
−10	7	−5	414.41	79.10
3	8	4	5.55	85.58
−13	14	−6	24.04	54.41
2	16	4	73.03	90.20
0	16	8	30.11	114.76
−9	25	−4	17.83	52.16
−8	28	−3	40.01	68.54
−6	33	0	57.18	121.36
−21	−20	5	−15.58	88.61
−17	−19	2	34.07	101.03
−12	−18	3	26.28	79.63
−13	−17	7	−14.26	65.77
−8	−16	5	8.58	58.77
−23	−15	−2	−56.26	100.37
−15	−13	−2	23.51	85.05
−1	−3	8	27.73	66.16
−24	−3	−6	101.29	107.89
2	−2	4	496.81	64.71
1	0	7	−21.79	68.54
−27	1	−7	−7.66	97.33
−3	2	10	9.77	86.90
−8	2	−4	314.44	75.01
−1	14	9	1.19	101.55
2	15	5	40.01	91.91
1	15	1	122.68	88.61
1	15	7	35.13	100.50
−1	20	0	128.23	82.14
0	23	4	34.34	96.40
−2	28	3	0.00	130.48
−20	−20	3	40.94	97.59
−21	−19	1	−157.02	103.54
−13	−18	2	84.12	91.65
−21	−18	0	−76.07	98.52
−6	−14	2	104.33	76.73
−5	−12	1	310.48	79.24
−14	7	−6	444.52	86.63
−7	8	12	15.19	101.16
1	19	3	30.24	83.86
0	21	6	72.50	115.95
−3	30	3	35.79	127.84
−17	−16	−1	−9.11	120.31
−15	−15	9	21.79	54.67
−12	−14	−1	96.93	84.92
−6	−13	1	9.90	75.01
−22	−13	−3	−3.57	109.21

−18	−12	−3	54.67	110.54
−3	−11	2	661.23	79.90
−11	−8	−3	38.56	89.01
−10	−7	−3	422.46	111.59
−4	−2	−2	130.74	53.88
0	1	8	11.49	75.54
2	2	6	133.51	72.37
−18	10	−7	0.00	85.05
2	11	6	141.57	86.50
−13	11	−6	56.26	61.54
2	12	2	82.93	80.56
−3	17	−2	460.50	78.31
−13	17	−6	−10.70	45.03
0	22	5	0.00	99.44
−1	22	7	83.99	125.46
−9	30	−3	−20.34	77.92
−4	32	3	−13.34	118.33
−5	34	3	0.00	108.29
−16	−19	6	33.54	69.20
−21	−19	7	−150.29	90.33
−11	−18	4	54.01	75.41
−7	−15	2	0.00	65.24
−20	−15	−2	−31.30	110.27
−4	−11	1	180.13	61.28
−20	−10	−4	−88.74	128.50
−9	−6	−3	40.81	90.33
−13	−6	−4	214.34	94.56
−9	−5	11	14.00	65.37
−12	−5	−4	6.60	81.88
−25	−4	−6	90.46	102.61
−19	−1	−6	−2.25	97.46
−18	0	−6	38.17	83.73
−17	1	−6	1.45	80.43
−11	3	−5	210.24	82.80
−1	5	9	46.22	112.12
−1	11	9	9.64	103.40
2	14	3	−41.34	99.44
1	17	2	177.62	95.74
−1	24	2	149.63	87.42
−1	25	4	−30.51	97.33
−18	−20	5	−42.26	83.33
−21	−20	2	13.47	95.61
−23	−20	6	−30.11	89.80
−5	−14	3	194.00	64.84
−16	−14	−2	103.54	93.50
−4	−13	3	72.24	66.56
−24	−11	−4	54.41	99.44
−14	−7	−4	207.20	96.27
−21	−7	−5	96.40	129.55
−11	−4	−4	134.70	95.61
−5	−3	10	−10.43	71.97
2	−1	3	299.25	47.67
−10	−1	12	−71.71	83.46
−6	1	11	13.21	86.76

-22	3	-7	10.30	116.61
-8	4	12	-22.05	93.63
-21	4	-7	130.21	117.27
-5	5	11	0.00	93.24
-1	8	9	-7.13	110.93
0	13	8	146.59	129.82
-3	14	10	-11.23	104.72
-1	18	8	-5.81	102.08
-1	22	1	14.26	79.90
-5	24	-2	0.00	68.41
-17	-20	3	32.22	89.54
-18	-19	1	35.92	103.54
-14	-18	1	0.00	100.63
-18	-18	0	186.07	112.78
-13	-10	-3	42.52	89.54
-8	-5	-3	287.76	83.86
-20	-2	-6	-38.03	109.08
-23	2	-7	40.67	107.37
-20	5	-7	-16.77	110.54
-29	6	-8	-38.96	103.27
2	8	6	-50.32	82.54
-17	14	-7	161.77	75.41
1	16	6	25.49	85.05
-3	22	-1	115.29	71.31
-1	23	6	35.26	134.17
-1	24	5	-77.78	120.70
-2	24	7	-30.90	118.85
-2	27	4	14.92	124.14
-14	-17	0	2.77	95.74
-6	-15	3	45.16	61.14
-13	-15	-1	5.68	84.78
-19	-13	-3	-27.86	123.08
-15	-8	-4	20.34	85.58
-10	-6	11	157.68	63.13
-3	-3	9	13.07	89.67
-21	-3	-6	-1.06	115.82
-3	5	10	-79.37	92.71
-5	8	11	22.85	99.84
2	12	5	72.77	78.05
-2	16	9	69.20	98.52
0	17	7	38.83	117.40
1	18	4	62.07	89.41
-2	26	2	14.92	83.86
-16	-20	4	31.96	84.12
-18	-20	2	64.05	94.69
-20	-20	6	-1.45	83.99
-13	-19	3	18.62	84.25
-14	-19	2	1.98	108.16
-9	-17	5	26.54	65.90
-8	-16	2	-37.24	71.31
-23	-14	-3	71.97	100.63
-3	-12	3	126.12	57.45
-21	-11	-4	11.23	116.21
-3	-10	1	167.19	52.82

−9	−10	−2	153.59	92.84
−8	−9	−2	−56.65	90.99
−22	−8	−5	82.41	115.03
−24	1	−7	−0.79	108.42
−15	3	−6	113.18	78.71
0	4	8	181.19	81.88
−19	6	−7	29.05	97.20
0	10	8	224.64	101.42
−3	11	10	69.46	96.93
2	13	4	62.33	86.50
1	17	5	89.41	83.59
0	21	3	264.52	94.16
−2	22	0	80.82	76.46
−10	27	−4	−0.79	57.71
−3	29	4	−79.90	129.42
−23	−21	4	0.00	97.86
−22	−19	0	0.00	101.95
−6	−12	0	312.59	81.22
−10	−11	−2	133.12	87.56
−14	−11	−3	26.81	86.37
−16	−9	−4	−2.64	89.67
−7	−8	−2	54.41	72.77
−11	−7	11	−21.92	58.24
1	−4	5	345.08	63.39
−15	−4	−5	10.70	82.41
−14	−3	−5	136.42	87.29
1	−2	6	281.95	68.80
−25	0	−7	71.45	98.65
1	3	7	134.44	75.67
−3	8	10	9.90	92.31
2	9	2	953.74	108.42
1	9	7	0.00	78.44
−2	24	1	137.87	83.20
−2	25	6	6.07	112.38
−2	26	5	−20.47	130.61
−3	28	2	51.24	93.90
−7	36	2	5.94	100.50
−10	40	1	59.43	103.80
−22	−20	1	0.00	97.59
−18	−19	7	−48.60	76.86
−12	−18	6	20.07	70.65
−7	−16	3	84.92	67.62
−9	−16	1	18.36	75.54
−21	−16	−2	33.81	104.33
−17	−15	−2	142.49	113.18
−7	−13	0	198.49	100.50
−5	−11	0	518.47	82.93
−2	−10	2	362.51	54.81
−3	−5	−1	111.46	54.67
−22	−4	−6	33.41	116.61
−13	−2	−5	96.01	75.54
1	0	1	704.55	62.33
−2	1	9	54.94	103.40
0	7	8	346.53	96.27

−8	7	12	−9.51	98.65
−13	8	−6	−1.19	68.01
−17	11	−7	0.00	75.41
−6	26	−2	−10.96	71.05
−4	30	2	34.07	126.91
−5	32	2	0.00	125.85
−6	34	2	−38.17	108.03
−20	−21	4	0.00	96.67
−25	−21	5	−34.34	94.69
−12	−19	4	7.53	80.16
−22	−18	−1	13.60	96.93
−15	−17	8	−40.54	64.71
−8	−14	0	190.83	89.67
−7	−4	−3	1303.84	135.10
2	3	2	301.63	55.33
−9	3	12	66.03	87.42
1	6	7	69.46	77.52
−18	7	−7	133.25	87.69
−8	10	12	0.00	101.42
2	11	3	50.84	78.31
1	12	1	300.44	73.43
−1	15	8	170.09	125.85
−4	16	10	71.97	100.76
0	17	1	461.02	97.73
0	18	6	62.07	89.14
−6	21	−3	63.39	52.16
−3	27	6	0.00	107.10
−3	28	5	64.45	111.33
−22	−21	5	99.71	92.97
−15	−19	1	−33.94	115.16
−19	−19	0	15.32	96.27
−14	−18	7	−72.50	67.62
−9	−17	2	47.81	73.16
−14	−16	−1	−53.88	86.90
−20	−14	−3	94.42	118.72
−15	−12	−3	40.28	83.73
−17	−10	−4	10.17	91.52
−23	−9	−5	20.07	105.78
−23	−5	−6	99.57	103.27
−26	−1	−7	−15.58	105.38
−10	4	−5	159.79	82.27
−14	4	−6	260.56	82.14
2	9	5	−0.53	87.16
−1	12	−1	127.31	48.86
1	13	6	74.88	86.24
−2	13	9	−17.17	100.76
−3	18	9	−87.03	99.57
0	19	2	184.36	82.01
−1	19	7	0.00	122.55
0	20	4	22.45	88.22
−1	23	3	154.38	87.03
−3	24	0	125.85	80.16
−4	24	−1	63.26	72.90
−3	26	1	95.48	93.37

−9	38	1	−49.26	99.18
−22	−21	2	−49.92	98.65
−19	−20	1	141.17	100.76
−15	−18	0	−20.07	112.12
−9	−15	0	−56.79	76.46
−12	−13	−2	−18.49	85.05
−22	−12	−4	1.32	107.10
−6	−7	−2	43.18	65.11
−18	−7	−5	149.10	97.99
−1	−5	7	16.11	55.47
−2	−4	8	29.85	66.69
−6	−4	10	36.45	67.75
−12	−1	−5	161.91	79.24
−7	0	11	41.86	78.71
−6	4	11	−50.84	96.01
−27	5	−8	−30.11	106.71
2	6	2	1409.88	131.14
−26	6	−8	0.00	98.52
−25	7	−8	70.65	97.46
0	14	7	217.77	91.39
1	16	3	28.53	89.80
0	19	5	232.69	89.14
−7	28	−2	141.17	73.69
−18	−21	3	−11.62	95.08
−15	−20	5	23.51	79.90
−8	−17	3	40.81	66.56
−10	−17	1	36.45	77.12
−4	−10	0	1056.36	108.42
−1	−4	0	888.90	84.52
2	3	5	585.16	94.42
−7	3	−4	1189.21	118.06
2	10	4	81.09	98.78
−2	17	−1	274.03	74.88
−4	28	1	31.17	91.65
−10	34	−2	0.00	113.18
−8	36	1	−96.40	103.93
−10	38	0	−96.54	98.52
−19	−21	5	64.18	85.84
−14	−20	3	−47.94	91.12
−15	−20	2	−0.26	104.20
−17	−20	6	18.49	76.73
−10	−18	5	27.73	70.26
−18	−16	−2	−2.11	117.01
−13	−14	−2	65.24	85.18
−16	−13	−3	34.34	93.90
−18	−11	−4	0.00	117.14
−19	−8	−5	0.00	118.33
−24	−6	−6	−1.06	98.78
−1	0	8	0.00	67.09
−2	4	9	−8.98	107.76
−28	4	−8	25.75	97.46
2	6	5	221.07	91.52
−2	10	9	8.45	102.61
0	12	0	692.79	83.46

−4	13	10	54.15	95.08
−6	13	11	−5.15	104.06
1	14	2	−7.66	89.54
−1	17	0	−37.90	78.44
−2	25	3	11.23	82.67
−5	30	1	17.70	104.46
−8	30	−2	30.64	81.48
−9	32	−2	56.39	103.93
−7	34	1	−48.20	116.35
−19	−21	2	37.77	91.91
−10	−18	2	90.20	74.75
−15	−17	−1	−0.92	108.03
−22	−17	−2	0.00	89.14
−10	−16	0	9.64	76.20
−2	−11	3	73.29	45.56
0	−1	7	−21.92	64.58
2	1	4	161.25	56.92
−6	7	11	−33.94	97.86
−17	8	−7	52.16	78.18
−9	11	−5	10.17	46.88
1	14	5	106.04	90.86
1	15	4	78.44	79.37
−1	20	6	−75.41	110.67
−2	21	7	12.55	120.70
−1	22	4	38.17	84.25
−4	26	0	65.37	80.43
−5	26	−1	−20.07	74.35
−6	32	1	37.90	118.99
−9	36	0	−66.82	103.80
−10	36	−1	14.66	100.76
−21	−15	−3	0.00	106.18
−2	−9	1	728.98	76.73
−17	−2	−6	3.57	84.12
−11	0	−5	584.50	94.16
−22	0	−7	47.54	106.71
−21	1	−7	12.81	113.31
−20	2	−7	−17.83	114.76
−4	4	10	80.16	90.86
−9	6	12	66.69	91.65
−2	7	9	121.23	111.72
2	8	3	414.80	83.59
−1	12	8	−31.96	111.46
−2	17	8	−20.87	112.52
−1	21	2	103.93	80.56
−7	35	3	98.39	104.72
−16	−20	1	64.05	102.35
−16	−19	0	−61.01	115.95
−9	−18	3	31.30	68.67
−11	−18	1	−29.05	79.63
−19	−12	−4	−84.39	123.61
−20	−9	−5	106.57	124.01
−5	−6	−2	430.39	71.31
−4	−4	9	28.26	78.18
−19	−4	−6	127.84	117.67

−6	−3	−3	54.67	59.96
−18	−3	−6	−57.31	96.67
−16	−1	−6	3.17	79.24
−23	−1	−7	0.00	101.29
2	2	3	675.10	76.60
−10	2	12	−20.21	85.05
−19	3	−7	87.29	105.65
−9	8	−5	132.06	63.13
1	10	6	−13.87	80.56
−4	10	10	61.14	92.31
−3	15	9	26.94	97.20
−4	19	−2	202.32	69.20
−1	21	5	48.07	86.24
−5	28	0	87.82	80.69
−6	28	−1	−2.38	73.29
−4	29	3	3.43	128.76
−5	31	3	0.00	126.12
−6	33	3	−60.62	116.21
−8	34	0	−36.71	125.72
−9	34	−1	−79.63	115.42
−22	−22	3	51.64	99.05
−21	−21	6	0.00	83.46
−20	−20	0	−57.18	96.54
−13	−19	6	40.41	70.65
−14	−15	−2	109.21	84.52
−7	−14	7	33.68	28.66
−17	−14	−3	45.83	115.55
−6	−13	7	−15.72	15.85
−8	−13	−1	28.26	94.56
−7	−12	−1	475.29	98.39
1	1	6	203.37	71.31
−13	5	−6	142.10	73.56
2	7	4	98.25	76.86
−4	7	10	19.15	88.74
0	11	7	289.87	84.92
−2	22	6	−33.94	121.76
−3	23	7	−76.07	107.37
−2	24	4	6.34	85.97
−6	30	0	−41.20	89.01
−7	30	−1	80.29	86.63
−7	32	0	173.92	109.48
−8	32	−1	−17.83	105.52
−21	−22	4	−29.58	98.65
−20	−21	1	55.07	96.54
−19	−17	−2	−98.91	108.82
−9	−14	−1	73.16	80.29
−6	−11	−1	441.74	94.82
−3	−9	0	493.25	67.62
−1	−7	6	189.38	23.90
−20	−5	−6	−43.58	124.53
−24	−2	−7	106.31	97.59
−8	−1	11	20.34	75.80
−15	0	−6	352.07	87.42
2	4	4	187.66	68.41

2	5	3	265.57	64.05
1	9	1	21.39	49.92
−1	9	8	21.92	92.84
−9	9	12	−25.75	100.63
−16	12	−7	5.28	68.01
0	15	6	2.51	82.93
−1	16	7	35.66	99.05
0	18	3	204.56	80.95
−20	−19	−1	29.32	100.50
−5	−15	4	45.43	53.62
−21	−10	−5	19.68	108.16
−7	−5	10	−7.26	62.60
−3	0	9	151.08	99.18
−1	3	8	11.23	76.73
−18	4	−7	−108.16	96.14
−5	15	10	0.00	96.01
−2	23	5	30.24	96.93
−7	23	−3	137.74	59.56
−4	25	7	0.00	90.33
−19	−22	3	0.00	88.61
−23	−22	5	−16.11	96.40
−15	−21	3	−116.48	90.73
−16	−21	2	75.54	95.08
−16	−21	5	0.00	79.90
−19	−20	7	−2.11	77.78
−11	−19	5	150.55	77.65
−6	−16	4	135.36	59.03
−15	−16	−2	57.84	99.57
−10	−15	−1	14.92	75.80
−18	−15	−3	−130.08	121.76
−20	−13	−4	−1.06	109.48
−13	−9	−4	217.90	92.05
−12	−8	−4	41.47	81.22
−11	−7	−4	66.16	81.22
−21	−6	−6	−27.34	109.74
−25	−3	−7	−22.05	98.25
−3	−1	−2	180.40	49.13
−7	3	11	22.45	88.74
−25	4	−8	73.95	97.06
−24	5	−8	−18.62	93.24
−1	6	8	93.50	78.71
−12	12	−6	314.97	64.84
−5	16	−3	167.06	51.37
−3	24	6	−22.32	127.17
−3	26	4	−101.16	103.67
−20	−22	2	117.40	99.57
−10	−19	3	−18.09	71.31
−12	−19	1	−84.12	90.20
−15	−19	7	10.96	67.35
−12	−18	0	15.98	84.65
−4	−14	4	168.25	50.71
−11	−11	−3	49.39	81.88
−5	−10	−1	190.70	76.20
−10	−10	−3	78.44	84.39

−14	−10	−4	178.28	93.24
−14	1	−6	15.05	74.35
0	2	7	162.96	72.50
−26	3	−8	171.42	101.42
1	4	6	154.12	70.78
−23	6	−8	−5.81	94.42
0	8	7	61.54	79.37
−12	9	−6	240.22	68.01
1	11	5	40.54	77.52
−3	12	9	19.02	107.50
1	13	3	−50.98	84.92
−3	14	−2	711.68	78.05
0	16	5	268.74	100.63
0	17	4	274.42	86.24
−4	17	9	−22.58	92.18
−8	20	−4	0.00	37.51
−3	25	5	32.88	118.72
−4	28	4	31.83	122.29
−5	30	4	111.33	124.40
−9	37	2	0.00	97.46
−18	−22	4	−74.48	91.12
−17	−20	0	0.00	103.40
−16	−18	8	−13.47	64.84
−7	−17	4	72.77	60.22
−11	−16	−1	−29.71	79.37
−12	−12	−3	73.56	84.25
−9	−9	−3	76.33	99.71
−22	−7	−6	127.04	102.88
−10	−6	−4	78.31	88.74
−10	1	−5	169.43	81.09
−27	2	−8	0.00	100.10
−17	5	−7	−39.49	85.31
−16	9	−7	121.10	75.80
0	14	1	1417.94	140.91
−2	14	8	−13.34	118.06
0	16	2	421.14	96.14
−2	19	0	51.11	90.99
−3	25	2	46.09	87.95
−4	26	6	54.01	120.44
−20	−22	5	11.49	82.27
−14	−21	4	53.09	81.35
−17	−21	1	171.42	94.56
−18	−21	6	48.60	78.58
−20	−18	−2	19.55	106.97
−9	−14	8	36.32	22.32
−15	−11	−4	100.10	89.54
−22	−11	−5	−15.58	97.86
−16	−8	−5	51.11	87.29
−13	−5	−5	0.00	71.31
−11	1	12	−32.75	82.54
0	5	7	217.90	79.76
−9	5	−5	225.03	75.94
−10	5	12	−2.38	93.24
−7	6	11	−25.88	96.54

−22	7	−8	23.37	100.89
1	11	2	179.87	70.26
1	12	4	54.01	100.63
−1	17	6	−1.72	82.67
−2	18	7	105.38	111.06
−3	19	−1	371.09	85.18
−1	20	3	120.97	84.65
−4	27	5	59.43	119.12
−5	29	5	−51.90	113.31
−8	35	2	−33.28	107.50
−21	−21	0	−64.18	100.23
−12	−20	2	23.37	106.04
−16	−17	−2	156.49	122.02
−21	−14	−4	114.36	102.88
−13	−13	−3	−27.47	89.93
−1	−9	2	46.88	36.05
−8	−8	−3	248.01	95.22
−4	5	−3	405.16	47.41
1	6	1	320.91	57.58
−7	9	11	−2.91	97.33
−5	12	10	−15.19	93.76
−4	27	2	−22.05	82.27
−7	33	2	0.00	123.87
−11	39	1	72.24	96.14
−21	−22	1	23.64	98.52
−14	−20	6	45.16	68.94
−13	−19	0	14.92	111.59
−8	−18	4	15.45	63.13
−12	−17	−1	45.56	82.27
−19	−16	−3	39.62	105.38
−16	−12	−4	24.04	97.86
−17	−9	−5	102.88	110.01
−23	−8	−6	−78.97	109.48
−3	−5	8	6.74	59.43
−9	−5	−4	264.12	94.03
−2	−4	−1	777.58	87.03
−12	−4	−5	101.69	84.39
−7	0	−4	2508.37	221.33
−3	3	9	164.68	107.37
−5	3	10	−5.68	82.80
0	9	0	1340.68	122.55
−3	9	9	−20.34	105.52
0	12	6	619.23	104.99
−5	19	9	40.67	93.76
−5	29	2	25.09	90.46
−6	31	2	73.82	118.85
−17	−22	2	−30.51	92.57
−11	−20	3	−12.81	78.44
−13	−20	1	−39.09	109.61
−14	−14	−3	113.18	88.35
−4	−9	−1	131.00	59.69
0	−7	4	1911.06	158.87
−4	−5	−2	1363.79	128.63
0	−3	6	557.96	74.75

-9	-2	11	91.39	74.35
1	-1	5	146.85	65.90
-2	-1	8	0.00	63.39
-20	-1	-7	0.00	113.57
-13	2	-6	154.91	74.61
1	3	1	669.15	74.88
-3	6	9	-61.80	109.08
-12	6	-6	222.92	72.90
-10	8	12	-28.39	96.93
-21	8	-8	-18.09	96.14
-5	9	10	-45.96	90.99
-10	11	12	-51.90	97.59
-1	19	4	5.81	82.54
-5	21	-2	101.55	63.13
-8	25	-3	53.35	61.80
-22	-23	4	102.61	101.55
-16	-22	3	0.00	87.56
-22	-22	6	-6.87	93.76
-9	-16	7	-23.24	50.05
-3	-13	4	149.23	47.28
-17	-13	-4	-9.77	113.04
-18	-10	-5	17.96	117.40
-22	-3	-7	-12.68	97.59
-5	-2	-3	465.12	70.26
-21	-2	-7	0.00	107.76
-19	0	-7	23.90	113.44
-5	6	10	116.74	88.48
1	8	5	211.43	92.31
-2	11	8	27.60	108.29
-9	14	-5	-68.94	22.45
-12	15	-6	103.67	46.88
-3	16	8	-32.35	121.76
-1	18	5	65.77	81.48
-3	20	7	-46.09	123.48
-2	22	3	153.45	88.35
-3	23	1	58.64	76.20
-10	37	1	-10.04	100.10
-20	-23	3	40.94	101.03
-18	-21	0	-10.70	94.42
-13	-18	-1	-37.11	92.57
-10	-14	-2	33.81	77.65
-9	-13	-2	50.58	78.18
-8	-12	-2	25.62	93.63
-7	-7	-3	1502.06	155.44
-8	-6	10	-92.71	61.80
1	-3	4	110.54	43.32
-6	-1	10	-26.68	77.12
-18	1	-7	15.32	104.20
-8	2	11	0.00	84.52
-16	6	-7	24.70	78.05
-6	7	-4	165.47	47.15
-4	14	9	132.33	99.31
-8	14	11	-0.13	103.01
-2	19	6	-13.73	98.12

−12	39	0	62.99	96.80
−21	−23	2	110.54	101.95
−17	−22	5	97.86	81.22
−18	−22	1	24.70	92.71
−13	−21	2	−17.70	107.50
−18	−20	−1	0.00	101.69
−9	−19	4	13.87	69.46
−17	−18	−2	0.00	110.67
−20	−17	−3	38.43	97.73
−15	−15	−3	0.00	104.46
−7	−11	−2	174.32	88.22
−19	−11	−5	2.25	114.50
−2	−8	0	60.09	47.15
−2	−6	7	292.25	55.86
−18	−6	−6	43.71	125.33
−17	−5	−6	31.30	101.55
−16	−4	−6	65.50	87.29
−23	−4	−7	0.00	100.23
−11	−3	−5	226.35	81.48
−12	0	12	2.38	83.20
0	15	3	4.36	88.48
−1	18	2	104.33	89.01
−3	21	0	167.59	77.65
−9	27	−3	−3.43	63.26
−9	35	1	−75.67	108.55
−14	−20	0	50.71	118.33
−11	−15	−2	151.47	85.71
−18	−14	−4	0.00	115.55
−19	−7	−6	−83.99	119.65
−8	−4	−4	52.69	81.35
−15	−3	−6	67.88	80.82
−1	−2	7	−1.58	61.14
−25	1	−8	−111.99	98.91
−24	2	−8	−8.06	94.69
−23	3	−8	46.09	92.97
1	10	3	76.20	72.63
0	13	5	53.22	78.05
−6	14	10	−49.92	95.88
−2	21	4	27.34	83.86
−4	21	−1	328.30	77.39
−4	22	7	33.81	115.16
−3	24	3	160.59	84.52
−4	25	1	159.40	82.01
−10	29	−3	94.16	71.84
−11	31	−3	−3.83	72.90
−19	−23	4	8.72	93.76
−12	−21	3	28.79	81.75
−14	−21	1	−73.29	104.33
−16	−20	7	0.00	72.77
−4	−1	9	−13.34	94.56
−9	2	−5	133.65	75.01
−17	2	−7	51.64	91.78
−11	4	12	59.56	90.73
−22	4	−8	1.58	94.16

1	5	5	432.50	91.91
−2	8	8	0.00	85.44
1	9	4	91.91	83.73
0	9	6	5.15	73.43
−1	10	7	84.12	77.92
−2	14	−1	600.75	76.07
−2	15	7	25.62	90.20
−2	20	5	14.39	82.14
−4	26	3	−15.98	84.78
−8	33	1	60.75	121.36
−8	34	3	10.17	104.06
−11	37	0	37.51	101.03
−21	−23	5	−0.13	87.16
−7	−16	6	26.15	48.86
−12	−16	−2	−13.87	81.09
−16	−16	−3	0.00	125.59
−20	−12	−5	33.41	107.63
−6	−10	−2	192.81	77.39
−1	−8	1	74.22	38.56
−20	−8	−6	149.36	112.78
−2	2	8	−12.81	73.16
−6	4	−4	92.84	52.96
1	8	2	1797.22	162.44
−15	10	−7	14.66	68.54
0	14	4	353.00	92.44
−1	14	6	−5.55	79.10
−4	18	8	31.96	117.67
−3	21	6	−15.05	97.59
−6	23	−2	−0.13	62.60
−5	24	7	51.50	106.04
−5	27	1	85.18	79.63
−6	29	1	12.28	82.93
−6	30	3	−42.92	120.97
−7	31	1	−3.43	98.91
−7	32	3	84.92	129.82
−12	37	−1	125.46	100.89
−18	−23	2	0.00	96.93
−19	−22	6	35.39	79.10
−13	−21	5	0.26	75.80
−18	−19	−2	42.92	108.95
−7	−17	1	154.25	74.35
−10	−17	7	54.15	54.41
−6	−15	6	13.47	46.62
−10	−3	11	66.16	73.43
−14	−2	−6	99.71	86.63
−12	3	−6	90.33	73.16
−2	5	8	92.84	79.10
−8	5	11	34.47	92.84
−21	5	−8	47.54	99.18
−8	8	11	29.32	93.50
−4	11	9	61.80	101.55
−15	13	−7	−8.19	63.92
−1	16	1	240.22	84.52
−5	16	9	−22.71	95.48

−3	23	4	0.00	82.01
−4	23	0	273.89	77.12
−10	35	0	9.11	116.21
−17	−23	3	0.00	93.90
−19	−22	0	−32.75	94.82
−15	−21	6	−104.99	77.39
−10	−20	4	90.07	76.33
−8	−18	1	70.92	76.33
−8	−17	6	54.81	56.39
−13	−17	−2	−99.05	89.67
−7	−16	0	334.91	86.37
−19	−15	−4	23.37	110.14
−21	−9	−6	21.79	109.08
−6	−6	−3	303.48	79.24
−10	−2	−5	213.94	76.86
−11	7	12	−36.85	96.14
−11	10	12	72.77	104.72
−6	11	10	0.00	91.39
−3	13	8	105.38	114.50
−3	22	5	30.90	88.61
−4	23	6	0.00	125.59
−19	−23	1	115.29	97.20
−14	−22	2	36.71	90.99
−15	−21	0	21.92	103.80
−19	−21	−1	0.00	94.56
−6	−17	2	−10.43	66.43
−8	−17	0	−52.16	78.58
−17	−17	−3	0.00	113.97
−6	−15	0	94.42	89.14
−1	−10	3	392.49	49.13
−3	−8	−1	203.90	54.01
0	−5	5	706.00	78.44
−16	3	−7	52.82	80.95
−1	7	7	26.28	76.60
−15	7	−7	12.68	69.07
0	13	2	587.01	94.03
−7	16	10	64.31	96.14
−2	20	2	−3.04	77.92
−5	23	−1	36.85	67.62
−4	25	4	58.90	85.71
−11	35	−1	51.50	115.82
−11	38	2	−15.58	94.42
−21	−24	3	48.20	97.99
−15	−22	1	−34.60	95.74
−15	−20	−1	−62.73	108.69
−9	−19	1	−32.22	77.39
−7	−18	2	70.65	71.58
−5	−16	2	166.40	68.01
−4	−14	1	556.24	92.05
−12	−11	−4	−0.26	84.39
−9	−7	10	37.51	56.13
−13	−1	−6	22.85	78.31
0	0	6	1038.79	111.86
0	6	0	2821.62	237.84

0	11	1	1182.34	117.67
-1	17	3	12.55	86.10
-3	17	7	29.58	97.59
-4	24	5	-17.17	97.86
-5	25	0	-12.81	76.07
-5	25	6	55.07	120.18
-7	25	-2	49.65	67.22
-5	27	4	-59.69	103.01
-7	31	4	30.51	115.42
-9	33	0	-73.69	116.48
-11	33	-2	28.53	107.37
-13	-22	3	-13.34	84.92
-14	-18	-2	0.00	106.97
-5	-14	0	136.42	86.37
-13	-12	-4	28.13	85.97
-11	-10	-4	162.70	84.25
-5	-9	-2	5.55	68.01
-14	-9	-5	-17.04	82.80
-1	1	7	42.13	67.35
-9	1	11	4.89	84.12
-4	2	9	92.97	102.88
1	6	4	93.10	68.80
-20	6	-8	90.20	106.71
-4	8	9	42.00	108.82
-6	8	10	38.30	88.61
-1	15	5	22.85	78.58
-6	18	9	-0.13	97.33
-5	26	5	42.26	113.31
-6	27	0	70.92	75.01
-6	27	6	33.68	110.14
-6	29	4	44.50	123.21
-8	31	0	-35.13	94.95
-10	36	2	69.46	102.61
-16	-23	4	-67.75	81.35
-18	-23	5	-27.34	81.48
-8	-19	2	21.00	71.97
-4	-15	2	19.02	73.16
-14	-13	-4	-51.50	91.52
-15	-10	-5	-34.20	93.63
-13	-8	-5	15.05	83.07
-21	-5	-7	-19.28	98.25
-20	-4	-7	-68.28	106.71
-19	-3	-7	7.92	119.12
-7	-2	10	-0.53	70.52
0	3	6	871.73	104.33
-12	3	12	44.37	88.48
-1	4	7	72.37	72.63
-4	5	9	-3.96	106.18
-6	5	10	-6.87	83.99
1	7	3	208.39	65.37
-2	16	6	36.18	84.39
-3	22	2	104.59	81.75
-9	22	-4	188.98	48.99
-6	25	-1	15.85	70.92

−8	27	−2	0.00	66.29
−6	28	5	−137.08	131.40
−7	29	0	−14.92	76.33
−9	29	−2	28.66	72.24
−7	30	5	−63.79	112.52
−10	31	−2	−134.83	87.69
−10	33	−1	37.37	111.06
−13	40	1	−123.34	96.93
−20	−24	4	164.68	99.57
−11	−21	4	18.22	78.71
−10	−20	1	3.43	82.27
−10	−19	0	29.85	80.56
−11	−18	7	9.64	56.39
−18	−18	−3	7.53	99.84
−11	−16	8	0.00	43.05
−5	−14	6	25.36	33.81
−16	−11	−5	5.68	113.44
−6	−6	9	54.28	63.65
−18	−2	−7	6.34	115.03
−1	0	−1	986.76	84.12
−23	0	−8	79.50	97.33
−11	7	−6	318.00	69.99
0	10	5	16.64	82.93
−6	10	−4	127.31	35.66
−1	16	4	215.79	80.43
−9	31	−1	−10.83	82.41
−19	−24	2	0.00	99.57
−15	−23	2	85.18	92.31
−14	−22	5	76.46	78.84
−16	−22	0	129.82	96.27
−9	−20	2	106.18	76.60
−8	−16	−1	7.79	82.27
−7	−15	−1	−2.77	82.54
−11	−14	−3	280.50	97.06
−15	−14	−4	100.89	108.42
−4	−13	0	248.67	79.90
−10	−13	−3	54.54	82.93
−17	−12	−5	33.15	116.61
−22	−6	−7	0.00	96.80
−3	−4	−2	2330.61	202.32
−17	−1	−7	147.51	109.74
−24	−1	−8	−73.16	112.91
1	0	4	78.05	49.26
−6	1	−4	691.34	85.58
−22	1	−8	−5.15	95.88
−15	4	−7	−25.09	78.97
1	5	2	1432.47	130.87
−3	10	8	−7.66	91.78
−11	10	−6	51.50	55.86
−5	13	9	126.78	100.37
−4	15	8	57.31	116.21
−4	19	7	62.73	119.52
−7	20	9	−11.09	95.22
−7	27	−1	19.94	73.16

−8	29	−1	41.73	74.35
−9	34	2	0.00	125.59
−18	−24	3	33.54	96.27
−22	−24	5	−38.30	92.57
−16	−23	1	85.18	92.84
−16	−21	−1	99.71	108.42
−17	−21	7	−50.98	75.54
−15	−19	−2	−76.73	115.69
−9	−17	−1	39.22	82.01
−12	−15	−3	88.88	87.03
−6	−14	−1	14.26	90.20
−3	−13	1	187.79	67.09
−9	−8	−4	59.56	78.97
−4	−6	8	0.00	60.88
−11	−4	11	−69.46	70.52
1	−2	3	146.32	34.47
−3	−2	8	62.99	66.69
1	−1	2	372.81	48.20
−9	−1	−5	24.70	79.90
−12	0	−6	8.32	75.67
−21	2	−8	45.43	106.31
1	3	4	405.69	69.86
−1	6	−1	829.08	76.33
−19	7	−8	107.10	114.63
−1	11	6	108.69	85.31
0	12	3	32.88	78.18
−7	13	10	25.75	95.74
−2	19	3	159.00	79.76
−8	32	2	42.79	117.67
−12	38	1	11.23	92.57
−20	−23	6	75.80	86.90
−16	−22	6	45.03	78.84
−11	−20	0	−1.85	91.91
−10	−18	−1	75.41	83.86
−13	−16	−3	−119.52	93.10
−16	−15	−4	73.95	125.59
−18	−13	−5	0.00	107.23
−8	−11	−3	−36.05	84.39
−17	−8	−6	97.06	126.91
−16	−7	−6	59.96	112.78
−15	−6	−6	14.26	89.80
−5	−5	−3	483.48	77.65
−9	4	11	78.05	90.86
−8	6	−5	216.45	70.65
−12	6	12	−37.24	92.31
−9	10	11	2.11	98.91
0	11	4	373.86	100.50
−4	16	−2	336.23	64.05
−6	18	−3	0.00	51.64
−5	26	2	132.19	82.67
−7	30	2	0.00	97.73
−14	40	0	0.13	94.42
−14	−23	3	−29.32	85.84
−11	−21	1	7.53	101.29

−10	−19	6	9.77	62.20
−3	−14	2	443.06	78.58
−18	−9	−6	79.76	116.61
−5	−2	9	116.08	94.82
−16	0	−7	129.82	93.90
−20	3	−8	−76.60	102.61
−11	4	−6	447.82	81.75
−9	7	11	0.00	91.91
−12	9	12	−56.65	103.40
−2	17	5	−40.41	82.54
−3	18	6	−14.66	80.95
−5	21	7	74.22	134.57
−6	28	2	11.36	88.48
−12	−22	4	15.19	78.18
−10	−21	2	50.58	92.44
−16	−20	−2	0.00	105.25
−11	−19	−1	−79.90	91.78
−6	−18	3	26.41	66.43
−17	−16	−4	−65.50	110.93
−5	−13	−1	−33.54	78.97
−2	−12	4	32.35	41.86
−14	−5	−6	160.32	84.39
−4	−1	−3	315.49	55.86
−1	15	2	−31.30	78.05
−2	18	4	134.70	80.16
−17	−24	4	−36.85	87.03
−17	−23	0	0.00	95.35
−17	−22	−1	−47.15	95.61
−12	−21	0	0.00	110.80
−7	−19	3	132.72	66.69
−5	−17	3	48.60	60.48
−14	−17	−3	76.33	108.55
−19	−14	−5	−61.80	108.42
−7	−10	−3	307.83	96.93
−4	−8	−2	124.80	64.45
−10	−8	10	0.00	54.15
−8	−7	−4	82.80	93.63
−10	−5	−5	−1.45	78.58
1	2	2	437.52	58.37
−8	3	−5	123.61	69.99
1	4	3	359.87	59.69
−3	7	8	202.32	85.31
−8	9	−5	215.66	59.30
−5	10	9	7.26	104.99
−7	10	10	9.77	88.22
−6	15	9	24.83	99.31
−3	16	−1	301.36	67.62
−5	17	8	60.22	124.14
−3	21	3	346.92	98.52
−6	23	7	15.05	123.74
−10	24	−4	200.07	54.01
−10	35	3	0.00	106.97
−11	36	1	−26.68	106.04
−16	−24	2	76.86	97.33

−19	−24	5	0.00	84.12
−12	−22	1	−19.55	112.52
−8	−20	3	−8.98	71.71
−12	−19	7	−0.66	58.64
−3	−12	0	393.28	73.03
−10	0	11	88.61	82.27
−7	1	10	−12.41	81.88
−13	2	12	−12.28	90.73
0	7	5	1354.55	145.80
−14	8	−7	0.00	66.29
−2	9	7	67.62	78.44
−8	15	10	−74.22	101.16
−4	20	6	58.37	88.61
−7	25	7	62.99	102.74
−13	38	0	−19.94	100.23
−21	−25	4	119.25	108.69
−17	−24	1	0.00	94.69
−11	−22	2	78.58	113.57
−7	−18	5	13.60	56.79
−15	−18	−3	0.53	116.87
−6	−17	5	0.00	50.84
−12	−17	8	0.00	48.47
−18	−17	−4	−1.06	100.89
−13	−4	−6	0.66	81.35
−3	1	8	44.90	68.28
−15	1	−7	−36.32	82.14
−19	4	−8	136.42	110.93
−1	11	0	422.86	61.28
−14	11	−7	108.42	63.39
−1	12	5	16.90	73.95
−3	19	5	137.61	86.63
−4	23	3	73.95	85.18
−9	33	3	22.98	116.48
−10	34	1	9.51	128.36
−19	−25	3	−40.01	101.16
−15	−23	5	2.77	79.76
−11	−20	6	−37.11	67.75
−8	−19	5	0.13	63.52
−10	−17	−2	−84.52	83.73
−4	−16	3	218.43	61.14
−9	−16	−2	0.13	77.26
−8	−15	−2	74.75	73.03
−1	−7	0	746.94	79.90
−12	−5	11	−43.18	70.78
−1	−4	6	74.48	50.84
−2	−3	7	84.92	56.65
−8	−3	10	−0.26	69.20
1	1	3	134.04	42.66
0	3	0	9280.85	753.80
−14	5	−7	−21.26	71.45
−7	7	10	265.44	97.86
−18	8	−8	−37.64	110.01
0	10	2	4727.92	397.24
−4	12	8	−45.56	99.71

−2	13	6	133.51	84.52
−6	19	8	−57.84	105.91
−3	20	4	−28.92	81.61
−8	31	3	41.47	124.53
−15	−24	3	6.47	90.73
−13	−22	0	0.00	112.25
−9	−21	3	56.79	78.44
−11	−18	−2	−3.17	81.75
−7	−14	−2	166.00	93.90
−4	−12	−1	548.85	92.05
−2	−7	−1	1097.29	109.35
−20	−7	−7	31.30	97.99
−19	−6	−7	−74.61	106.18
−18	−5	−7	8.32	122.42
−23	−3	−8	44.24	99.44
−22	−2	−8	35.13	94.69
−11	1	−6	−38.03	72.77
−7	4	10	75.94	88.22
−5	7	9	37.11	107.50
−1	13	1	384.30	73.29
−7	17	9	23.24	95.35
−3	18	0	13.87	75.67
−5	22	6	156.36	102.88
−5	25	3	31.43	81.61
−11	26	−4	95.48	56.39
−6	27	3	16.24	75.80
−10	34	4	−18.49	104.33
−18	−24	0	47.94	100.50
−18	−23	−1	−72.11	100.89
−13	−21	−1	−70.92	121.50
−16	−19	−3	15.32	103.40
−2	−12	1	619.76	76.33
−6	−9	−3	169.83	81.22
−21	−8	−7	59.96	91.65
−3	−7	7	190.30	49.79
−17	−4	−7	54.54	121.89
−21	−1	−8	22.45	95.22
−5	1	9	0.00	96.40
−13	5	12	−10.17	93.50
0	8	1	126.38	49.92
−11	13	−6	122.29	49.65
−1	14	3	47.67	85.97
−4	16	7	57.05	93.37
−9	17	10	24.30	97.46
−4	21	5	102.88	87.69
−4	22	4	31.43	87.29
−6	24	6	14.39	128.36
−9	32	1	−10.04	105.25
−12	36	0	−40.41	108.42
−12	−23	2	101.55	97.99
−13	−23	1	16.38	97.06
−17	−23	6	45.30	81.22
−9	−20	5	63.39	71.71
−12	−19	−2	0.00	96.93

−5	−16	5	32.62	45.96
−2	−8	6	68.94	36.58
−7	−6	−4	276.67	91.12
−9	−4	−5	−13.60	72.77
−12	−3	−6	−5.55	82.93
0	−2	5	232.56	61.54
−8	0	−5	−6.74	78.05
−20	0	−8	−29.58	99.97
0	4	5	1039.06	121.63
−5	4	9	−19.94	106.71
−2	6	7	14.39	75.14
−13	8	12	12.94	99.18
−10	9	11	−34.60	97.06
−1	13	4	206.68	89.80
−2	17	2	42.92	98.25
−5	24	1	22.85	79.10
−7	26	6	−37.37	117.14
−8	28	6	14.66	110.54
−8	30	1	27.20	85.05
−12	37	2	−28.00	108.42
−15	41	1	0.40	99.05
−17	−25	2	−66.82	102.88
−10	−22	3	0.00	72.24
−12	−14	−4	15.85	83.46
−6	−13	−2	103.54	89.93
−11	−13	−4	−34.86	86.37
−15	−13	−5	9.24	123.34
−14	−12	−5	−30.51	102.08
−13	−11	−5	−35.39	86.37
−16	−3	−7	146.85	113.44
−10	3	11	137.87	89.54
−18	5	−8	92.97	114.63
0	8	4	69.99	77.39
−6	12	9	−26.54	100.37
−8	12	10	0.00	94.42
−8	19	9	−38.30	96.80
−5	23	5	19.68	86.50
−8	23	8	−13.73	106.18
−5	24	4	3.30	80.82
−6	26	1	169.96	82.80
−7	28	1	251.84	86.37
−7	28	4	−81.75	109.08
−8	30	4	36.45	121.76
−9	31	5	2.11	111.20
−18	−25	1	0.00	100.23
−18	−25	4	104.20	95.48
−14	−23	0	0.00	98.65
−14	−22	−1	108.42	103.01
−12	−21	6	41.60	68.14
−13	−20	7	−4.75	63.92
−13	−15	−4	15.72	97.06
−10	−12	−4	184.75	88.88
−12	−10	−5	125.46	85.97
−7	−7	9	1.85	58.64

−14	1	12	−60.88	90.46
−14	2	−7	0.00	73.56
−10	6	11	80.95	95.88
0	9	3	225.43	70.52
−3	11	−2	562.45	57.58
−5	14	8	56.13	128.23
−6	25	5	97.20	104.33
−6	26	4	116.61	92.44
−7	27	5	22.19	127.17
−8	29	5	93.24	124.93
−11	34	0	−8.06	119.12
−13	36	−1	34.60	111.06
−13	−20	−2	77.65	122.55
−17	−20	−3	4.09	95.88
−14	−16	−4	26.28	121.63
−3	−15	3	12.41	55.99
−16	−14	−5	34.07	118.19
−16	−10	−6	29.05	116.87
−11	−9	−5	−35.79	82.54
−11	−9	10	8.72	50.05
−2	0	7	126.78	68.41
−19	1	−8	−96.01	106.71
−2	3	−2	458.78	47.54
−2	3	7	−23.11	71.84
−1	5	6	116.08	67.22
−4	9	8	99.31	86.90
−3	11	7	111.33	81.48
−2	14	5	37.77	73.56
−3	15	6	217.11	88.88
−4	18	−1	670.08	92.44
−5	18	−2	194.00	65.24
−5	18	7	−23.24	110.67
−4	20	0	−36.98	84.78
−7	20	−3	147.91	52.96
−16	−25	3	−89.41	100.23
−20	−25	5	17.17	94.56
−14	−24	1	−56.26	95.35
−14	−24	4	99.71	82.80
−16	−24	5	−16.77	80.69
−13	−18	8	0.00	52.43
−17	−15	−5	34.73	102.61
−9	−11	−4	190.43	85.18
−15	−9	−6	−57.58	111.06
−13	−6	11	0.00	67.35
−4	−4	−3	369.37	66.95
−15	−2	−7	−3.96	97.20
−11	−1	11	66.56	79.50
−2	0	−2	118.72	42.92
−14	14	−7	211.43	63.65
−11	35	2	−12.41	107.63
−14	39	1	15.72	96.67
−20	−26	3	45.96	101.42
−13	−24	2	23.37	91.39
−11	−23	3	0.00	82.01

−15	−17	−4	3.83	127.31
−5	−12	−2	12.02	80.82
−2	−11	0	33.68	54.15
−3	−11	−1	1218.66	123.08
−14	−8	−6	0.00	94.42
−3	−7	−2	3446.13	293.31
−6	−3	9	23.64	83.33
−11	−2	−6	107.50	80.16
−10	5	−6	104.20	64.58
−2	16	3	58.24	87.95
−3	19	2	141.31	88.08
−10	32	0	17.04	93.76
−16	41	0	105.38	96.80
−15	−24	0	0.00	94.69
−15	−23	−1	0.00	104.59
−14	−21	−2	145.27	110.54
−11	−17	−3	154.91	89.01
−10	−16	−3	102.88	89.80
−18	−16	−5	59.69	99.57
−9	−15	−3	41.86	80.56
−5	−8	−3	316.02	80.82
−13	−7	−6	130.48	87.29
−1	−3	−1	715.64	77.78
−4	−3	8	68.67	67.75
−1	−1	6	250.65	64.58
−8	0	10	12.81	77.12
−1	2	6	223.98	71.31
−18	2	−8	−10.83	114.36
−1	9	5	120.44	86.90
−8	9	10	0.00	91.91
−2	10	6	−24.43	73.43
−7	14	9	26.81	102.74
−9	14	10	−2.77	95.61
−2	15	4	83.46	84.25
−6	20	7	1.32	125.59
−10	33	2	175.11	127.57
−12	34	−1	91.65	119.78
−13	34	−2	5.94	111.06
−12	−18	−3	66.03	104.86
−16	−18	−4	20.47	111.72
−8	−14	−3	128.76	83.20
−8	−10	−4	144.74	78.44
−14	4	12	−5.81	97.73
−17	6	−8	−48.99	109.87
−6	9	9	0.00	104.59
−14	10	12	0.00	109.74
−11	11	11	−14.26	105.78
−6	16	8	60.09	121.36
−4	17	6	46.88	81.22
−5	22	0	198.62	75.94
−9	30	0	0.13	79.24
−12	36	3	−0.66	106.18
−11	−22	5	−15.98	75.27
−7	−20	1	38.83	73.03

−8	−20	0	16.11	78.44
−6	−19	1	−16.77	70.92
−6	−18	0	37.11	78.71
−5	−7	8	53.75	66.29
−6	−5	−4	311.27	86.90
−9	−4	10	99.31	69.33
−21	−4	−8	39.22	101.82
−8	−3	−5	166.40	79.50
−10	2	−6	0.00	67.48
−4	6	8	−50.18	81.61
−13	6	−7	66.16	71.18
−10	8	−6	−15.19	58.37
−2	15	1	417.05	80.82
−4	21	2	131.27	83.86
−7	22	7	39.09	119.38
−9	31	2	0.00	109.74
−13	37	1	22.19	113.04
−11	32	−1	65.90	92.71
−8	28	0	−2.11	76.33
−7	26	0	157.68	84.25
−6	24	0	50.58	73.03
−12	30	−3	−12.68	73.43
−9	26	7	26.54	109.87
−8	24	7	29.32	114.63
−5	20	−1	208.66	81.61
−8	22	−3	61.94	54.94
−3	16	5	0.00	74.88
−1	12	2	179.87	69.33
−8	6	10	46.09	90.86
−13	9	−7	1.06	61.54
−14	7	12	22.58	103.01
−14	−1	−7	31.03	87.82
−12	−6	−6	−61.54	83.33
−4	−15	5	15.19	42.52
−5	−17	0	75.14	79.37
−5	−18	1	125.99	71.05
−20	−3	−8	59.16	99.97
−18	−8	−7	−98.78	107.37
−19	−9	−7	−7.53	101.95
−8	−21	1	−20.21	76.07
−9	−21	0	−121.50	86.90
−13	−19	−3	−75.14	132.99
−13	−22	6	−45.83	73.16
−15	−25	1	−22.85	97.59
−18	−24	6	−24.70	85.97
−19	−26	4	42.39	107.50
−15	39	0	−27.60	113.04
−11	34	3	6.60	117.67
−8	29	2	28.53	90.99
−12	32	−2	0.00	94.03
−3	18	3	52.43	85.84
−5	19	6	67.22	86.24
−7	18	8	0.00	121.10
−7	15	−4	111.59	26.81

−8	16	9	62.99	101.16
−10	16	10	41.86	101.29
0	5	4	622.54	85.44
−3	8	7	−31.96	76.99
−5	2	−4	450.86	67.22
−11	8	11	23.11	99.57
−11	2	11	10.30	91.12
−13	3	−7	101.82	75.67
−7	−13	−3	−32.75	85.18
−19	−2	−8	−32.62	107.10
−17	−7	−7	−125.33	125.59
−7	−18	−1	6.60	80.29
−7	−20	4	−6.21	67.09
−8	−19	−1	−5.41	88.61
−9	−20	−1	−11.36	84.39
−14	−21	7	37.64	70.39
−12	−24	3	38.30	83.33
−17	−19	−4	0.00	104.20
−14	−25	2	−69.20	97.06
−17	−26	3	8.19	102.08
−7	27	2	115.69	83.73
−3	17	4	242.20	89.27
−6	20	−2	90.46	63.92
−2	13	0	128.63	53.88
0	7	2	7693.87	632.57
−5	11	8	7.13	97.06
−6	6	9	−7.40	110.80
−4	3	8	10.83	74.88
−4	0	8	334.38	80.16
−17	3	−8	190.56	121.76
−4	−11	−2	165.87	80.16
−12	−10	10	18.49	51.64
−16	−6	−7	47.81	122.55
−6	−17	−1	15.45	72.63
−6	−19	4	0.00	59.16
−8	−21	4	−40.54	74.09
−9	−22	1	75.14	96.93
−10	−22	0	1.45	105.25
−14	−20	−3	−9.24	112.52
−15	−25	4	−42.92	87.16
−16	−24	−1	−21.00	103.40
−16	−25	0	0.00	103.14
−17	−25	5	7.13	87.82
−12	35	1	−129.82	132.46
−10	32	3	136.95	125.59
−10	30	−1	−28.66	77.26
−11	28	−3	54.81	67.22
−9	24	−3	−2.51	60.09
−4	18	5	108.16	87.42
−1	10	4	30.51	82.54
−9	11	10	−66.95	95.61
−2	−3	−2	340.19	55.33
−6	0	9	−49.79	98.39
−11	5	11	75.27	96.27

−7	−9	−4	120.70	96.40
−14	−19	8	−12.41	61.41
−10	29	6	29.58	109.08
−11	30	−2	123.74	81.61
−4	20	3	0.00	76.99
−6	22	−1	30.24	65.63
−10	26	−3	52.30	63.13
−6	21	6	29.71	91.12
−8	20	8	0.00	103.27
−1	11	3	123.08	68.14
−11	21	−5	121.23	30.90
−9	18	9	118.46	100.76
−7	11	9	73.82	107.63
−6	3	9	32.35	104.86
−7	4	−5	829.87	100.37
−5	−1	−4	664.40	84.12
0	−8	2	547.26	53.22
−10	−1	−6	55.20	73.56
−12	−2	11	37.11	82.80
−18	−1	−8	−14.26	105.12
−4	−16	0	86.50	87.29
−15	−5	−7	7.79	113.44
−5	−18	4	125.19	59.69
−6	−20	2	65.77	71.71
−7	−21	2	169.43	75.67
−9	−22	4	1.06	78.18
−10	−23	1	−10.04	111.99
−11	−23	0	−2.77	119.65
−12	−23	5	16.90	75.94
−16	−23	−2	0.00	100.76
−14	38	2	131.80	102.48
−9	30	3	41.20	105.38
−9	27	6	69.73	126.78
−8	25	6	0.00	122.82
−7	23	6	90.59	113.04
−3	17	1	636.14	102.08
−3	12	6	212.62	88.35
0	6	3	45.30	52.43
−5	5	−4	632.18	71.18
−7	1	−5	70.92	75.67
−1	−11	1	58.50	46.75
−11	−5	−6	192.28	89.93
−6	−12	−3	51.64	91.39
−5	−16	−1	0.00	80.03
−13	−14	−5	−1.32	113.97
−8	−22	2	−16.51	79.90
−11	−22	−1	0.00	124.01
−15	−21	−3	26.15	106.44
−13	−25	3	9.77	91.12
−16	−26	1	−110.67	106.97
−14	37	0	69.07	107.10
−11	33	1	−4.75	124.27
−10	31	4	125.99	127.97
−8	26	−1	22.58	73.03

−10	28	−2	1.98	67.75
−5	22	3	110.93	80.56
−7	24	−1	138.27	70.12
−4	19	4	42.39	82.80
−5	20	5	59.03	90.99
−7	22	−2	161.38	62.60
−10	20	9	−8.32	97.33
−2	11	5	−14.66	81.48
−1	6	5	355.77	89.41
0	−7	1	1091.88	97.59
−4	−7	−3	1225.79	126.38
−15	3	12	−54.54	105.12
−13	0	−7	132.85	85.44
−2	−14	3	−27.60	57.31
−5	−19	2	−25.09	69.99
−12	−13	−5	93.50	90.73
−8	−18	−2	9.51	85.05
−9	−19	−2	20.21	90.73
−14	−15	−5	40.94	120.31
−14	−23	6	27.86	77.78
−15	−26	2	−123.87	104.99
−10	30	5	71.58	118.33
−7	26	3	142.63	85.97
−6	24	3	84.78	81.88
−6	13	8	5.68	110.27
0	5	1	764.24	87.69
−15	9	12	−20.21	107.63
−16	7	−8	95.08	109.48
−2	−10	−1	683.42	82.14
−8	−6	−5	18.88	67.09
−17	0	−8	−13.07	114.63
−14	−4	−7	89.93	104.86
−11	−12	−5	0.00	93.90
−7	−17	−2	159.66	92.97
−11	−16	−4	3.96	90.33
−16	−13	−6	0.00	114.36
−10	−20	−2	−75.94	95.61
−15	−16	−5	−33.15	114.76
−9	−23	2	189.51	109.48
−10	−23	4	0.00	81.48
−11	−24	1	1.06	104.99
−12	−23	−1	−3.96	118.85
−12	−24	0	−78.05	108.95
−16	40	1	−17.83	101.95
−9	29	4	28.13	125.46
−9	28	5	−5.15	124.53
−8	26	5	−36.18	108.82
−7	24	5	75.94	95.74
−9	26	−2	31.30	71.31
−5	21	4	80.16	89.14
−6	22	5	51.24	86.76
−8	24	−2	50.32	66.03
−1	10	1	25.75	48.86
−10	13	10	40.41	102.48

0	2	4	533.66	73.29
-3	5	7	105.38	75.14
-9	8	10	0.00	94.03
-13	12	-7	7.40	59.56
-12	10	11	-23.37	111.99
-15	6	12	20.21	107.63
-16	4	-8	95.22	117.53
-8	-8	9	-8.19	57.97
-4	-18	2	50.71	66.16
-10	-15	-4	0.13	88.61
-12	-17	-4	-15.85	109.87
-13	-18	-4	36.32	127.44
-17	-14	-6	51.24	115.82
-11	-21	-2	-14.66	125.85
-16	-17	-5	-94.95	115.55
-15	-22	7	31.03	76.60
-16	-26	4	21.00	89.27
-18	-27	3	0.00	106.31
-20	-27	4	-13.07	111.86
-13	36	2	-47.15	112.91
-10	31	1	75.27	88.48
-8	27	4	0.00	108.16
-7	25	4	24.96	85.18
-6	23	4	-0.40	92.71
-2	14	2	117.67	74.48
-6	17	7	-32.88	93.76
-5	8	8	4.62	84.12
-3	-4	7	168.11	59.96
-7	-2	-5	171.81	82.80
-9	-1	10	4.75	80.16
-6	-8	-4	0.00	87.16
-3	-15	0	161.11	80.56
-4	-15	-1	167.32	91.52
-4	-17	4	185.55	59.03
-10	-11	-5	288.03	102.08
-6	-16	-2	47.54	83.07
-9	-14	-4	154.12	90.86
-13	-10	-6	-21.66	104.59
-20	-6	-8	75.94	104.86
-13	-24	5	13.73	81.88
-13	35	0	-45.03	121.89
-15	37	-1	-85.97	106.71
-4	14	6	271.39	94.03
-1	8	0	746.41	77.65
-4	10	7	69.33	79.76
-8	13	9	1.58	104.99
-10	11	-6	45.56	52.56
0	-3	0	9999.00	812.04
-3	0	-3	1136.25	106.31
-10	-4	-6	356.43	90.86
-5	-11	-3	78.97	89.14
-19	-5	-8	9.64	107.76
-14	-19	-4	103.27	119.38
-10	-24	2	22.71	102.61

−12	−22	−2	33.41	129.55
−12	−25	1	−46.22	101.55
−13	−24	−1	0.00	112.25
−14	−26	3	−7.00	99.44
−18	−26	5	144.74	100.50
−7	19	7	24.70	112.25
−2	12	4	62.86	80.69
−8	12	−5	204.17	41.20
−7	8	9	−40.15	109.35
−7	7	−5	1405.00	133.38
−9	5	10	82.67	93.50
−1	−6	−1	202.98	51.77
−12	4	−7	110.27	73.95
−1	−10	0	375.71	58.50
−12	1	11	−9.24	92.44
−3	−10	−2	218.03	67.62
−10	−5	10	0.00	70.39
−12	−9	−6	32.49	88.35
−18	−4	−8	54.81	104.06
−13	−11	10	41.20	49.79
−17	−10	−7	0.00	104.86
−18	−11	−7	3.83	102.35
−15	−20	8	0.00	67.09
−13	−25	0	37.51	98.65
−12	34	2	1.06	128.50
−9	29	1	0.00	89.93
−2	13	3	69.46	78.05
−7	15	8	3.96	136.29
−12	18	−6	−15.45	28.79
−11	15	10	0.00	105.12
−3	2	7	29.32	66.95
−12	7	11	56.52	101.55
−9	3	−6	20.47	67.35
−9	2	10	50.32	89.01
−2	−6	−2	1303.44	122.82
−16	1	−8	−36.58	117.40
−13	−3	−7	61.01	96.67
−9	−10	−5	174.19	85.71
−5	−15	−2	122.95	90.07
−8	−13	−4	167.32	86.76
−16	−9	−7	120.31	109.35
−15	−20	−4	−50.84	109.35
−15	38	1	78.58	106.84
−8	27	1	−27.73	80.29
−8	21	7	58.77	129.68
−12	23	−5	27.86	38.56
−3	13	5	99.84	78.05
−5	13	−3	339.26	42.39
−2	4	6	160.45	69.86
0	−1	4	118.99	47.41
−3	−1	7	78.97	64.18
−12	7	−7	79.10	69.07
−3	−3	−3	641.29	77.52
−12	4	11	−21.13	98.91

−12	1	−7	68.67	76.20
−13	−3	11	−10.17	81.75
−15	−8	−7	113.97	125.06
−22	−1	−9	−58.90	111.86
−9	−18	−3	−19.94	84.52
−10	−19	−3	36.85	98.91
−11	−25	2	27.47	97.46
−15	−24	6	−0.13	83.07
−13	35	3	71.45	125.19
−12	33	0	100.23	114.36
−7	25	1	163.89	83.86
−6	23	1	15.05	71.97
−10	25	7	69.07	113.70
−9	23	7	35.66	123.61
−5	16	6	88.35	84.39
−3	13	−1	145.00	47.94
−9	15	9	0.00	101.82
−5	5	8	2.25	75.54
−13	12	11	0.00	116.21
−9	0	−6	203.51	76.07
−11	−8	−6	82.41	89.67
−3	−17	2	97.46	65.37
−17	−3	−8	4.23	108.29
−8	−17	−3	34.34	87.95
−11	−20	−3	118.99	134.57
−13	−26	1	−48.33	104.59
−14	−25	−1	113.44	110.01
−14	−26	0	119.25	102.35
−17	40	0	−12.94	101.29
−11	32	2	−9.51	128.23
−14	35	−1	−118.46	127.17
−3	16	2	95.08	78.18
−8	17	8	−91.65	133.38
−1	7	4	48.86	72.37
−3	9	6	−8.85	74.09
−12	17	10	35.26	107.37
−1	3	5	42.79	68.67
−10	10	10	14.92	102.22
−7	5	9	32.62	112.38
−9	6	−6	2.64	59.43
−1	−6	5	138.27	47.54
−5	−4	8	79.10	73.03
−7	−5	−5	152.79	87.29
−3	−14	−1	865.79	114.10
−7	−12	−4	117.01	85.97
−21	0	−9	46.09	109.35
−7	−22	3	−23.90	77.26
−12	−21	−3	83.59	123.21
−12	−25	4	−3.04	86.37
−14	−24	−2	−158.47	108.69
−14	−25	5	0.13	85.44
−17	−27	4	14.39	101.16
−5	12	7	103.80	85.31
−6	10	8	52.56	92.31

−2	−5	6	3.96	46.09
−16	8	12	115.16	118.46
−16	5	12	−17.30	113.44
−2	−15	1	158.74	70.92
−14	−7	−7	164.02	134.83
−7	−16	−3	125.19	97.46
−6	−21	3	99.97	70.92
−8	−23	3	36.98	83.20
−16	−23	7	0.00	76.60
−15	−27	3	220.01	112.12
−16	39	2	−11.49	103.01
−15	35	−2	−79.50	121.50
−6	18	6	−5.41	85.84
−3	14	4	349.70	100.50
−9	19	8	23.51	117.67
−10	17	9	0.00	104.06
0	3	3	1338.57	121.89
−8	10	9	−32.22	107.23
−7	−1	9	56.26	97.06
−15	5	−8	146.72	115.42
−2	−14	0	64.97	69.73
−8	−9	−5	18.36	89.27
−4	−14	−2	140.64	91.39
−16	−2	−8	136.15	120.84
−5	−20	3	14.00	66.82
−13	−22	−3	−57.71	125.99
−12	−26	2	86.90	95.22
−15	−26	−1	−34.86	117.01
−17	−28	2	64.18	110.14
−14	36	1	−7.00	120.04
−12	33	3	19.02	136.02
−10	30	2	−40.81	96.01
−11	31	0	−43.71	87.42
−3	15	3	54.28	85.18
−4	15	5	46.49	80.56
−1	9	2	2007.33	180.40
−2	8	5	296.08	89.54
0	4	2	3012.58	254.35
−4	7	7	228.20	83.46
−5	2	8	70.12	77.12
−7	2	9	17.17	106.18
−4	−8	7	67.35	45.30
−5	−7	−4	44.37	90.59
−9	−3	−6	−2.77	79.50
−4	−10	−3	299.65	87.42
−12	−2	−7	61.41	82.80
−10	−7	−6	279.44	97.20
−9	−24	3	−1.98	88.48
−14	−27	1	31.03	108.03
−11	28	6	54.67	121.10
−4	18	2	119.91	89.27
−4	17	0	353.53	78.84
−7	20	6	−3.04	92.05
−11	19	9	119.25	110.93

−2	1	6	129.82	66.82
−13	9	11	2.64	110.40
−5	−1	8	107.89	74.88
−15	8	−8	0.00	108.03
−15	2	−8	−80.03	121.89
−13	−6	−7	108.29	111.20
−6	−15	−3	38.43	82.01
−19	−8	−8	4.23	117.27
−12	−16	−5	131.40	133.91
−14	−14	−6	−51.77	128.63
−7	−22	0	11.23	89.01
−13	−17	−5	−14.13	137.61
−15	−15	−6	0.00	121.23
−14	−23	−3	17.43	107.37
−15	−27	0	−64.71	112.25
−18	41	1	80.56	114.76
−11	31	3	23.51	122.68
−12	32	4	0.00	141.17
−13	33	−1	−15.58	111.46
−9	28	2	−3.70	87.95
−12	31	5	−53.75	119.38
−10	26	6	104.46	132.46
−9	24	6	83.99	117.93
−8	22	6	16.90	106.31
−2	12	1	711.41	85.31
−1	8	3	217.64	61.80
−11	12	10	53.88	103.67
−10	7	10	6.60	99.44
−3	−6	−3	106.04	59.03
−6	−8	8	−9.24	71.71
−6	−11	−4	120.04	81.35
−3	−16	4	273.76	55.33
−18	−7	−8	6.34	112.78
−11	−15	−5	0.00	111.72
−6	−21	0	83.33	82.67
−7	−21	−1	153.85	88.74
−8	−23	0	0.00	103.01
−14	−18	−5	0.00	113.84
−13	−26	4	0.00	92.18
−16	−25	6	57.71	94.82
−16	38	0	32.88	110.67
−10	29	0	26.28	81.09
−5	17	5	59.69	94.42
−4	13	−2	85.58	40.28
−7	12	8	50.84	106.84
−12	10	−7	86.63	63.13
−2	−2	6	129.16	59.43
−10	−2	10	−40.94	82.80
−13	0	11	−36.85	95.22
−4	−19	3	107.89	65.77
−17	−6	−8	21.53	105.65
−10	−14	−5	78.71	101.82
−21	−3	−9	59.96	117.67
−6	−20	−1	69.33	84.65

−9	−23	−1	5.68	117.27
−9	−24	0	67.75	123.08
−15	−19	−5	160.45	101.03
−10	−25	3	−13.34	87.56
−13	−27	2	−48.60	108.82
−15	−26	5	−153.85	97.73
−16	−28	3	48.20	113.97
−18	−28	4	−51.50	118.46
−15	37	2	81.61	121.50
−13	34	1	137.87	127.44
−8	26	2	21.79	83.73
−11	29	5	0.00	135.36
−6	22	2	−18.88	78.97
−4	17	3	141.31	95.74
−4	16	4	127.70	89.14
−4	11	6	348.24	87.82
0	−6	0	3007.03	252.37
−13	6	11	87.42	109.48
−15	−1	−8	103.01	124.27
−2	−16	2	242.73	77.26
−14	−4	11	−11.49	84.65
−6	−22	1	−92.97	82.93
−10	−18	−4	46.09	112.65
−16	−12	−7	50.58	94.42
−7	−23	1	73.16	97.59
−11	−19	−4	0.00	145.14
−10	−24	−1	41.07	124.40
−11	30	4	10.70	132.33
−14	33	−2	30.24	113.18
−9	27	0	127.31	81.75
−10	27	5	79.76	127.17
−5	19	0	207.86	87.82
−6	19	5	16.24	86.63
−9	12	9	10.56	109.08
−1	−3	5	475.02	72.50
−6	−1	−5	127.17	78.58
−1	−9	−1	7.26	52.69
−13	3	11	−67.75	102.35
−2	−9	−2	986.23	107.37
−2	−13	−1	418.24	82.80
−11	−6	10	−13.21	73.69
−5	−14	−3	134.31	85.97
−9	−13	−5	133.38	90.20
−20	−2	−9	−9.77	112.78
−5	−19	−1	17.43	83.86
−9	−17	−4	0.00	99.31
−15	−11	−7	−33.28	117.53
−8	−21	−2	57.31	91.78
−8	−24	1	−70.39	119.38
−12	−20	−4	−31.03	122.55
−10	−25	0	145.53	116.35
−15	−28	1	73.03	113.97
−12	31	−1	0.00	90.86
−9	25	5	3.96	99.71

−7	16	7	77.39	92.71
−12	14	10	139.59	113.57
−6	7	8	4.36	84.92
−8	7	9	−3.30	115.82
−14	11	11	−12.94	122.68
−6	2	−5	172.47	74.75
−10	1	10	−28.53	90.07
−11	2	−7	79.50	78.05
−17	7	12	0.00	119.91
−9	−6	−6	223.32	87.16
−3	−13	−2	144.74	85.18
−3	−14	5	128.76	37.90
−12	−5	−7	8.19	107.63
−16	−5	−8	−54.54	117.27
−11	−11	−6	3.17	96.93
−5	−21	1	5.41	78.84
−7	−20	−2	−44.11	91.91
−9	−22	−2	0.00	119.52
−10	−23	−2	74.09	132.19
−9	−25	1	0.00	115.95
−13	−21	−4	−82.14	111.33
−11	−25	−1	5.94	111.20
−11	−26	3	11.75	92.84
−9	27	3	46.49	92.18
−10	28	4	42.26	110.40
−8	25	0	75.80	77.52
−7	23	0	201.26	77.12
−6	21	0	15.85	80.69
−8	23	5	50.71	95.88
−5	19	3	160.32	94.42
−7	21	5	70.39	87.56
−5	18	4	146.19	79.24
−4	15	−1	249.99	57.45
−11	5	−7	−32.22	72.50
−11	−1	−7	−0.13	81.09
−17	4	12	−9.38	120.84
−9	−9	9	12.41	57.84
−8	−16	−4	−20.47	89.80
−14	−10	−7	20.34	134.17
−6	−19	−2	−72.24	89.27
−11	−26	0	−42.92	108.82
−14	−27	4	0.00	95.35
−17	39	1	24.70	110.67
−14	35	2	7.53	126.78
−15	36	0	50.45	132.19
−17	38	−1	−96.67	114.23
−12	32	1	−68.01	100.37
−8	25	3	41.07	99.18
−9	26	4	70.65	97.99
−6	21	3	93.76	79.90
−6	20	4	−11.09	81.35
−8	18	7	−13.07	112.52
−2	9	4	11.49	75.41
−3	10	5	279.04	90.33

−8	14	8	7.79	131.53
−5	9	7	76.46	81.75
−4	4	7	270.86	81.22
−11	9	10	−29.32	103.54
−4	0	−4	1364.06	129.29
−6	−4	−5	189.64	89.54
−8	−5	9	25.36	71.31
−19	−1	−9	22.32	109.61
−4	−18	−1	27.20	82.41
−4	−20	1	14.13	74.88
−19	41	0	66.03	110.40
−15	36	3	115.16	126.51
−7	23	3	236.13	90.59
−8	24	4	−13.47	90.07
−7	22	4	57.84	92.31
−12	26	7	0.00	133.78
−9	20	7	100.76	129.02
−10	14	9	112.12	113.84
−13	16	10	0.00	113.70
−8	1	−6	40.41	68.80
−14	3	−8	101.16	115.29
−1	−13	3	173.66	52.56
−5	−10	−4	29.45	103.27
−15	−4	−8	−16.38	119.78
−8	−12	−5	81.48	100.50
−10	−10	−6	88.08	99.31
−7	−15	−4	36.05	90.07
−13	−9	−7	−101.42	131.27
−9	−21	6	4.75	67.35
−10	−26	1	62.60	110.54
−12	−26	−1	4.49	115.55
−16	−27	5	−3.57	101.42
−17	−29	3	0.00	121.50
−11	29	−1	8.19	77.52
−13	31	−2	−0.26	85.31
−14	31	−3	−9.51	83.20
−11	24	7	−70.12	128.89
−10	22	7	84.78	147.64
−3	14	1	1622.24	153.85
−5	13	6	37.90	86.50
−1	4	4	489.95	76.07
−9	9	−6	−4.89	56.26
−15	13	11	1.85	122.16
−14	6	−8	61.28	108.42
−8	−2	−6	50.84	74.75
−3	−9	−3	287.23	83.73
−1	−14	1	225.16	66.43
−3	−18	3	155.57	64.05
−5	−18	−2	0.00	90.07
−8	−20	6	21.26	64.18
−9	−23	5	56.92	81.09
−10	−22	6	14.53	72.90
−12	−25	−2	56.13	115.03
−12	−27	0	18.09	109.61

−2	11	2	1006.30	106.84
−9	16	8	172.21	138.93
−11	16	9	−120.44	109.35
−2	5	5	641.68	99.97
−8	4	9	85.31	114.36
−14	8	11	66.03	117.27
0	−10	1	151.21	43.05
−1	−13	0	148.96	59.16
−14	0	−8	324.21	132.72
−11	−4	−7	26.94	86.90
−4	−13	−3	102.35	96.67
−3	−18	0	159.53	79.24
−7	−21	5	−22.58	72.63
−8	−20	−3	12.02	94.56
−9	−21	−3	−35.66	118.72
−7	−24	2	−109.61	92.71
−10	−22	−3	−73.69	141.70
−11	−27	1	−29.58	107.76
−13	33	2	28.39	141.70
−11	30	1	70.26	90.73
−2	10	0	53.22	42.39
−2	10	3	273.23	73.03
−10	18	8	46.22	125.33
0	0	3	333.19	47.28
−6	4	8	94.69	85.18
−18	9	12	−10.43	127.31
−4	−6	−4	202.19	82.14
−14	−1	11	0.00	98.91
−17	−9	−8	−126.78	114.76
−6	−23	2	90.73	80.29
−8	−25	2	93.10	112.25
−10	−24	5	103.01	85.84
−11	−23	−3	148.44	135.63
−11	−23	6	75.14	77.39
−13	−26	−2	109.61	114.50
−13	−27	−1	42.39	116.61
−15	−28	4	−30.11	105.52
−15	−29	2	−57.84	120.84
−14	34	0	−35.92	126.91
−14	34	3	−83.73	139.98
−10	27	−1	−24.17	78.18
−13	29	6	131.66	123.74
−5	17	−1	502.10	81.09
−6	15	6	159.13	89.54
−12	18	9	8.32	107.50
0	2	1	1187.49	102.74
−7	9	8	27.20	93.90
−9	9	9	0.13	113.97
−4	1	7	96.54	70.26
−8	4	−6	−6.87	63.13
−9	−9	−6	124.67	91.39
−18	0	−9	173.00	113.44
−3	−17	−1	0.00	84.78
−6	−14	−4	44.11	86.50

−12	−8	−7	0.13	119.38
−15	−5	11	58.50	95.08
−3	−19	1	107.10	74.22
−16	−8	−8	−58.90	118.72
−7	−19	−3	139.59	90.99
−5	−22	2	−21.00	75.27
−13	−16	−6	−17.83	122.95
−14	−17	−6	53.09	118.99
−12	−24	−3	136.68	113.84
−13	−28	0	106.57	118.19
−16	37	1	29.58	121.76
−16	36	−1	−75.67	127.70
−14	32	5	−5.28	124.14
−11	20	8	−142.63	120.18
−4	12	5	173.26	82.54
−13	20	9	−50.32	111.99
−1	7	1	272.05	56.26
−6	11	7	66.29	85.97
−12	11	10	−62.20	113.31
−11	6	10	−47.67	104.72
−8	1	9	71.84	109.74
−14	5	11	−34.60	110.80
−8	−2	9	98.65	94.69
−8	−5	−6	151.87	85.05
−14	−3	−8	−35.92	132.59
−7	−11	−5	286.70	92.71
−4	−17	−2	110.40	91.52
−19	−4	−9	−54.94	123.21
−6	−20	5	−2.91	66.29
−12	−15	−6	−34.60	143.15
−11	−18	−5	127.84	141.17
−12	−19	−5	−29.71	129.55
−13	−20	−5	35.00	114.76
−9	−26	2	−64.84	110.67
−13	−28	3	108.29	116.21
−14	33	4	34.20	116.35
−10	28	1	138.53	89.54
−12	29	−2	78.58	79.50
−13	29	−3	53.48	77.52
−12	27	6	41.60	139.98
−13	27	−4	11.75	66.03
−5	8	−4	106.97	37.11
−6	5	−5	268.35	66.56
−11	8	−7	140.64	69.20
0	−9	0	1089.77	103.67
−4	−5	7	35.00	58.37
−14	2	11	35.13	108.82
−18	6	12	46.75	130.08
−11	−3	10	85.71	87.69
−6	−18	−3	84.78	91.25
−10	−17	−5	−14.53	141.31
−15	−14	−7	−9.38	116.74
−11	−25	5	−27.60	89.01
−12	−28	1	67.75	116.87

−17	38	2	129.16	115.03
−18	39	0	0.00	111.86
−12	31	2	2.51	105.12
−9	25	−1	−71.58	76.33
−4	16	1	447.69	80.69
−7	17	6	309.15	97.86
−3	11	4	3.17	84.39
−4	3	−4	396.98	58.90
−15	10	11	33.41	128.76
−6	−5	8	41.07	75.14
−2	−12	−2	64.18	73.43
−15	−7	−8	55.73	121.63
−4	−21	2	81.22	75.01
−11	−14	−6	−26.54	128.89
−14	−13	−7	0.00	119.65
−7	−23	4	30.24	84.12
−8	−24	4	−31.03	86.37
−12	−24	6	−44.90	86.90
−10	−27	2	98.25	106.18
−17	−28	5	−28.39	115.95
−13	32	3	43.32	137.87
−9	26	1	0.00	88.35
−13	30	5	214.86	154.51
−11	25	6	19.81	135.10
−6	19	−1	29.05	70.26
−8	19	6	89.01	89.54
−5	15	−2	465.91	66.95
−8	17	−4	19.15	31.17
−2	8	−1	1499.55	129.02
−11	16	−6	96.27	30.90
−3	3	6	567.33	88.22
−13	13	10	−9.90	115.55
−6	1	8	125.19	83.99
−4	−2	7	−14.26	62.73
−11	3	10	−51.37	105.52
−10	0	−7	107.76	84.92
−1	−12	−1	186.34	62.33
−1	−15	2	231.11	68.14
−11	−7	−7	43.98	106.18
−2	−17	0	206.28	90.33
−12	−7	10	−46.35	77.78
−18	−3	−9	−4.09	114.63
−9	−16	−5	23.24	102.88
−6	−22	4	0.92	77.26
−13	32	0	−33.54	104.59
−13	31	4	−36.32	142.10
−8	23	−1	99.71	68.67
−7	21	−1	562.18	96.54
−10	23	6	0.00	108.29
−9	21	6	−97.73	97.46
−3	13	2	318.93	75.54
−7	13	7	8.19	81.48
−8	11	8	−6.60	106.18
−5	6	7	39.49	80.29

−10	11	9	26.68	120.44
−1	−5	−2	1301.99	122.42
0	−11	2	445.57	55.60
−11	0	10	15.45	96.67
−4	−9	−4	248.27	96.40
−17	1	−9	22.32	110.40
−5	−17	−3	165.34	88.22
−13	−12	−7	35.39	131.93
−9	−20	−4	−13.47	129.82
−10	−20	7	−9.24	57.18
−10	−21	−4	−4.62	142.23
−11	−21	7	10.56	64.18
−11	−22	−4	0.53	125.85
−13	−29	1	16.51	122.82
−14	−29	3	−57.71	120.18
−16	−29	4	34.34	116.48
−15	35	1	−39.22	141.44
−11	29	2	45.43	91.78
−8	24	1	120.97	80.69
−11	27	−2	27.86	74.35
−5	18	1	−10.04	84.65
−3	12	3	221.99	79.90
−5	14	5	−19.94	80.69
−6	15	−3	148.83	44.90
−1	5	3	347.32	61.41
−10	3	−7	200.20	83.20
−6	−2	8	52.96	78.58
−13	1	−8	22.05	118.99
−10	−3	−7	122.55	85.18
−3	−12	−3	117.67	89.80
−8	−8	−6	10.96	94.69
−3	−16	−2	85.71	95.61
−2	−18	1	195.58	78.18
−8	−15	−5	18.88	93.63
−12	−23	−4	−31.30	116.35
−10	−26	4	22.85	92.71
−12	−26	5	−102.74	97.46
−11	−28	2	7.13	113.84
−19	40	1	11.23	117.53
−15	34	−1	101.29	131.14
−12	28	5	−26.68	139.46
−7	22	1	26.68	81.22
−12	25	−4	42.52	60.62
0	1	2	308.63	50.05
−14	15	10	47.41	126.12
−9	6	9	0.53	115.95
−12	8	10	28.66	109.61
−16	12	11	117.53	133.91
−2	−5	−3	148.96	58.77
−13	4	−8	78.31	105.25
−13	−2	−8	54.67	129.29
−2	−16	−1	41.07	90.07
−2	−17	3	27.20	55.33
−14	−6	−8	22.05	139.85

−3	−20	2	63.52	73.03
−12	−11	−7	95.88	141.44
−5	−21	4	84.65	70.92
−8	−19	−4	−42.13	106.71
−7	−24	−1	193.47	118.06
−8	−25	−1	−15.98	129.68
−12	−22	7	28.53	70.78
−13	−25	6	42.79	92.44
−21	42	0	−6.87	124.40
−16	36	2	−24.43	132.59
−17	37	3	0.00	120.70
−12	29	4	22.32	120.44
−12	27	−3	13.07	67.62
−4	13	4	112.38	92.44
−1	6	2	1534.28	138.66
−1	−2	−2	444.91	61.41
−15	7	11	−46.22	120.70
−19	8	12	0.00	135.23
−1	−11	4	47.67	20.07
−3	−9	6	60.75	31.43
−2	−15	4	11.49	39.88
−17	−2	−9	−76.86	119.25
−5	−19	5	0.00	58.11
−16	−11	−8	29.32	120.70
−6	−24	0	114.76	104.46
−7	−25	0	78.05	131.40
−9	−26	−1	−3.96	122.29
−19	39	−1	31.56	107.50
−10	27	2	77.78	88.08
−11	26	5	38.43	113.04
−6	16	5	−0.53	83.46
−3	7	5	1081.84	128.76
−9	13	8	49.39	129.16
−7	10	−5	270.46	46.88
−11	13	9	48.86	114.10
−15	17	10	40.01	125.85
−7	6	8	3.70	89.01
−2	−2	−3	769.12	82.01
−5	−3	−5	145.93	87.16
−1	−8	−2	1895.34	171.55
−15	−2	11	125.46	104.20
−7	−18	−4	−11.89	96.01
−5	−22	−1	89.93	92.05
−5	−23	0	7.13	85.97
−7	−23	−2	16.90	123.34
−8	−24	−2	27.47	147.64
−8	−26	0	29.71	122.42
−11	−27	4	0.00	96.93
−14	−30	1	−10.96	118.99
−17	37	0	44.11	126.38
−12	30	0	−5.15	87.95
−11	28	3	−13.21	95.48
−10	25	−2	88.74	73.69
−10	24	5	−29.98	107.10

−11	23	−4	23.64	55.99
−13	25	7	24.83	140.78
−4	15	2	118.99	77.39
−6	17	−2	176.70	64.58
−7	18	5	385.22	97.99
−9	17	7	−35.13	105.91
−5	10	6	149.76	83.46
−12	13	−7	27.20	58.50
−7	−1	−6	5.81	72.24
−19	5	12	8.85	141.44
−10	−6	−7	88.74	96.14
−7	−14	−5	281.16	103.93
−18	−6	−9	28.92	121.76
−15	−10	−8	74.75	124.27
−6	−22	−2	69.46	98.91
−9	−27	0	13.60	113.70
−10	−26	−2	28.00	117.80
−10	−27	−1	36.85	116.48
−13	−27	5	−2.25	101.69
−15	−30	3	55.73	133.78
−14	33	1	153.85	129.95
−11	27	4	−30.77	107.23
−9	22	5	79.24	98.12
−8	20	5	66.43	103.54
−12	23	7	117.80	157.42
−4	14	3	306.38	90.07
−3	12	0	416.65	62.33
−10	19	7	46.09	120.70
−2	6	4	118.33	67.88
−12	15	9	46.88	118.72
−1	1	4	52.16	50.84
−5	0	−5	613.29	96.40
−2	−8	−3	232.56	68.01
−5	−6	−5	104.72	86.10
−7	−4	−6	453.10	92.97
−15	4	11	−32.62	115.29
−13	−5	−8	−14.92	138.14
−11	−10	−7	44.50	140.78
−6	−18	6	22.98	59.56
−7	−25	3	66.95	91.52
−13	−23	7	6.07	83.20
−17	−30	4	0.00	133.38
−16	35	3	137.48	130.08
−16	34	−2	37.37	128.76
−9	23	−2	143.95	66.03
−11	25	−3	83.07	68.28
−9	19	−4	−38.17	24.70
−11	21	7	89.14	137.48
−10	15	8	60.09	140.38
−6	8	7	264.91	92.44
−8	7	−6	−58.24	55.99
−3	0	6	859.19	100.50
−13	10	10	−18.09	111.86
−13	7	−8	17.30	87.56

−15	1	11	0.00	110.67
−9	−6	9	0.00	74.09
−10	−10	9	0.00	44.24
−6	−17	−4	106.04	94.69
−4	−21	−1	122.29	97.33
−5	−21	−2	60.35	101.55
−6	−24	3	−2.91	90.59
−12	−18	−6	100.10	129.42
−8	−26	3	24.17	96.40
−10	−28	0	40.28	117.27
−11	−27	−2	−139.85	120.18
−11	−28	−1	41.07	115.29
−14	−26	6	55.20	102.88
−15	34	2	13.34	155.04
−14	32	−1	92.57	101.16
−10	26	3	118.59	96.27
−8	23	2	0.00	82.41
−10	25	4	16.24	97.20
−5	17	2	−123.21	90.20
−7	19	−2	202.32	73.29
−10	21	−4	165.47	51.24
−11	17	8	1.85	142.36
−13	17	9	0.00	116.08
−10	8	9	98.78	119.78
0	−3	3	1137.71	99.05
−10	6	−7	3.57	70.92
−9	3	9	−55.73	118.59
−16	9	11	8.98	133.65
−4	−12	−4	167.19	90.07
−7	−9	8	29.58	52.69
−1	−16	0	−21.53	81.22
−16	−1	−9	48.60	116.87
−8	−11	−6	36.85	99.31
−17	−5	−9	39.62	118.72
−14	−9	−8	88.48	120.97
−4	−20	4	0.79	64.84
−11	−17	−6	−30.90	140.78
−6	−25	1	144.34	123.74
−8	−23	−3	−107.89	156.23
−7	−26	1	5.02	124.01
−9	−24	−3	−132.85	147.91
−8	−27	1	−59.16	116.61
−9	−27	3	3.30	99.97
−12	−28	4	−36.18	105.38
−13	−30	2	−53.35	131.66
−18	38	1	152.79	134.70
−19	39	2	−27.20	125.59
−11	28	0	28.00	83.59
−7	21	2	244.84	94.82
−9	23	4	115.42	95.08
−6	19	2	287.76	107.37
−8	21	−2	−20.47	65.77
−5	16	3	−2.51	91.78
−6	17	4	128.10	88.88

−13	21	8	−18.09	124.01
−12	19	8	−55.33	134.70
−14	19	9	−7.53	121.89
−5	3	7	74.61	76.60
0	−5	−1	609.20	76.99
−7	2	−6	−2.25	68.54
−3	−5	−4	100.37	71.58
0	−12	0	518.87	71.71
0	−13	1	285.78	61.41
−7	−7	−6	461.69	93.76
−2	−15	−2	4.75	99.71
−5	−23	3	−19.41	82.01
−13	−15	−7	87.56	118.33
−5	−24	1	0.00	94.16
−7	−22	−3	66.03	117.01
−10	−20	−5	22.05	148.17
−11	−21	−5	−48.33	128.10
−10	−25	−3	6.74	123.48
−11	−29	0	46.62	119.38
−9	24	3	28.92	93.24
−14	28	6	283.80	149.63
−8	21	4	144.21	94.29
−7	19	4	250.26	92.97
−7	17	−3	459.57	67.88
−6	12	6	152.66	85.05
−4	5	6	68.80	70.39
−8	8	8	11.09	100.37
0	−8	−1	79.37	49.39
−1	−11	−2	224.24	69.73
−12	−1	−8	17.96	123.48
−12	−4	10	5.68	87.69
−6	−13	−5	−0.13	97.59
−10	−9	−7	−13.34	122.16
−2	−19	2	0.79	62.99
−3	−20	−1	159.00	87.16
−4	−20	−2	91.91	93.24
−10	−16	−6	70.78	141.83
−6	−21	−3	0.00	96.80
−9	−19	−5	31.03	144.47
−9	−28	1	41.47	110.54
−11	−26	−3	0.00	115.55
−14	−24	7	117.14	97.33
−14	−28	5	38.43	108.69
−20	40	0	−28.92	118.59
−16	35	0	−89.93	150.15
−13	31	1	0.00	96.40
−15	31	5	−87.95	135.49
−8	22	3	43.58	99.84
−10	23	−3	−28.39	62.99
−6	18	3	−26.41	97.06
−2	9	1	1208.75	114.89
−14	12	10	−28.26	120.84
−7	3	8	182.24	92.57
−9	0	9	0.00	107.37

−12	2	−8	4.49	105.12
−9	−2	−7	57.45	85.84
−20	7	12	67.62	150.68
−1	−15	−1	126.25	88.22
−3	−15	−3	10.43	96.14
−5	−16	−4	18.49	89.80
−13	−8	−8	−38.03	131.93
−13	−8	10	−31.96	84.25
−12	−14	−7	157.68	143.81
−4	−23	1	55.47	82.67
−18	37	−1	58.37	128.36
−15	33	3	−89.41	158.87
−10	26	0	8.32	81.61
−7	20	3	161.91	85.71
−13	26	6	97.86	149.63
−4	9	5	177.75	90.99
−1	5	0	869.89	80.95
−17	11	11	0.00	136.15
−9	1	−7	113.70	80.95
−12	2	10	66.95	106.71
−3	−8	−4	54.81	85.58
−2	−11	−3	42.26	80.56
−12	−4	−8	35.00	136.15
−16	−4	−9	9.38	120.70
−9	−15	−6	5.94	126.78
−4	−22	3	111.20	73.95
−8	−18	−5	15.72	127.44
−10	−29	1	−39.22	118.72
−12	−30	0	92.71	127.84
−13	−29	4	−50.58	111.99
−21	41	1	14.39	124.01
−14	32	2	−19.55	119.38
−15	32	4	−36.58	163.62
−9	21	−3	40.15	56.52
−8	19	−3	67.88	55.73
−7	14	6	814.02	121.23
−7	10	7	299.25	97.06
−11	10	9	16.51	120.04
−3	−3	6	256.33	62.20
−16	6	11	−26.54	130.48
−12	−1	10	8.72	96.93
−9	−5	−7	0.00	85.44
−11	−13	−7	54.94	143.42
−5	−20	−3	0.00	92.44
−11	−29	3	−95.88	115.95
−14	−31	2	−106.04	139.46
−13	30	−1	98.52	85.71
−15	32	−2	0.00	106.57
−12	24	6	−79.63	139.06
−4	14	0	1129.39	113.31
−2	7	3	223.58	61.54
−15	14	10	0.00	128.89
−7	−10	−6	369.77	106.04
−16	−3	11	12.94	109.21

−3	−19	−2	−30.51	82.41
−3	−22	1	−61.28	83.59
−8	−22	−4	132.46	140.12
−9	−23	−4	−25.62	136.95
−10	−24	−4	0.00	127.84
−17	36	1	45.30	146.46
−18	37	2	−9.64	138.00
−12	29	1	−20.73	94.42
−9	24	0	−93.50	80.03
−14	29	5	−120.44	143.95
−11	22	6	−26.81	108.82
−8	16	6	2.11	90.33
−10	14	−6	223.05	44.50
−9	10	8	−31.83	113.97
−11	11	−7	277.33	69.20
−16	16	10	75.14	133.51
−13	7	10	0.00	113.04
−3	−6	6	0.00	47.28
−15	0	−9	−52.96	122.02
−4	−18	5	−44.90	51.90
−7	−17	−5	103.40	107.76
−14	−12	−8	28.26	132.59
−11	−30	1	−93.63	130.87
−15	−29	5	0.26	121.63
−10	20	6	201.92	110.80
−9	18	6	111.33	102.74
−3	8	4	85.84	76.46
−5	3	−5	467.50	80.56
−2	−1	5	11.09	65.11
−10	5	9	−62.86	121.63
−12	5	−8	193.21	107.76
0	−11	−1	1418.60	134.70
−16	3	11	−71.71	127.31
−5	−9	7	−20.21	47.28
−5	−12	−5	81.88	92.18
−9	−8	−7	7.13	100.89
−4	−15	−4	178.68	92.57
−12	−7	−8	−19.68	140.51
−2	−19	−1	−20.73	81.75
−4	−19	−3	227.54	99.05
−16	−7	−9	76.20	132.19
−8	−18	7	−9.24	29.85
−7	−21	−4	−138.93	135.89
−6	−26	2	−4.23	100.50
−7	−27	2	−34.07	113.18
−15	33	0	−72.77	117.53
−14	30	4	68.54	155.04
−16	32	−3	81.09	96.54
−15	26	7	0.00	128.23
−2	8	2	1292.08	123.61
−5	11	5	99.18	95.74
−8	12	7	141.57	94.56
−12	12	9	69.73	125.06
−5	0	7	165.87	75.54

−7	0	8	380.20	95.61
−3	−11	−4	235.46	96.93
−16	0	11	−21.13	115.82
−15	−3	−9	−89.80	126.91
−10	−12	−7	100.37	155.57
−5	−25	2	−43.18	94.95
−8	−28	2	−91.91	114.89
−12	−30	3	28.92	129.16
−14	−30	4	14.00	122.68
−19	38	0	−79.90	129.95
−13	30	2	28.13	99.18
−8	22	0	126.65	83.86
−13	27	5	48.33	131.93
−6	−3	−6	7.92	76.60
−3	−19	4	26.02	64.05
−3	−21	3	141.70	71.58
−13	−11	−8	−1.72	130.34
−6	−20	−4	14.79	109.35
−12	−31	1	260.29	141.83
−17	35	−1	0.26	141.04
−18	36	3	−75.94	140.38
−11	27	1	13.07	95.74
−12	28	−1	0.00	77.92
−7	20	0	87.82	85.71
−6	18	0	279.18	83.86
−14	24	7	41.60	139.98
−10	12	8	−12.02	127.97
−3	4	5	490.34	89.67
−13	14	9	3.96	129.02
−6	5	7	0.00	79.37
−9	4	−7	0.00	75.27
−14	9	10	−56.65	123.21
−4	−5	−5	212.49	92.18
−17	8	11	189.90	138.40
−6	−6	−6	190.30	87.56
−1	−14	−2	57.97	84.25
−2	−14	−3	125.85	102.88
−1	−16	3	4.23	59.03
−6	−16	−5	4.49	96.54
−11	−20	−6	−17.04	134.17
−6	−26	−1	−44.50	133.78
−7	−27	−1	18.75	131.14
−9	−29	2	−43.18	118.85
−20	39	1	−45.03	123.61
−13	28	4	25.49	111.46
−12	25	5	68.01	110.93
−7	5	−6	61.54	64.18
−1	−2	4	94.29	43.98
−8	5	8	−29.85	92.05
−13	4	10	0.00	115.16
−11	0	−8	145.14	114.63
−7	−6	8	−68.14	92.57
−11	−3	−8	185.28	133.51
−1	−18	2	64.05	69.60

−2	−18	−2	−39.09	80.69
−15	−6	−9	−148.30	127.84
−2	−21	1	87.69	80.69
−4	−24	2	70.26	87.56
−10	−19	−6	−40.67	133.38
−12	−17	−7	28.26	119.52
−5	−25	−1	−17.43	119.91
−6	−25	−2	62.20	146.59
−8	−27	−2	−76.60	133.51
−8	−28	−1	22.05	123.87
−16	34	1	0.00	146.06
−17	35	2	−25.62	159.53
−14	30	−2	70.78	88.74
−13	22	7	−52.96	150.68
−3	11	1	363.43	63.52
−12	20	7	0.00	137.61
−6	13	5	149.63	85.05
−16	20	9	−11.09	123.21
−11	14	8	127.97	143.68
−15	18	9	−77.52	124.40
−14	16	9	85.18	120.44
0	−2	−1	1325.23	113.57
−3	1	−4	172.74	55.86
−7	−3	8	199.54	90.73
0	−15	0	166.79	74.22
−9	−11	−7	−7.66	132.33
−3	−18	−3	218.43	95.48
−12	−10	−8	112.38	136.55
−5	−19	−4	0.00	99.71
−10	−23	−5	−13.21	127.57
−9	−28	−2	−62.73	123.61
−9	−29	−1	0.13	121.10
−13	−31	3	−20.07	136.02
−22	41	0	69.86	135.49
−12	28	2	43.18	93.90
−16	29	6	−80.69	140.38
−11	23	5	10.70	100.89
−11	18	7	20.47	112.12
−15	22	8	54.67	126.25
−4	10	4	243.12	87.69
−1	2	3	275.61	50.45
−4	2	6	107.63	68.80
−11	7	9	46.75	120.70
−4	−2	−5	225.69	83.46
−6	0	−6	55.60	72.24
−5	−3	7	16.77	67.48
−10	2	9	−77.65	111.72
−8	−4	−7	30.90	86.50
−6	−9	−6	220.28	99.57
−11	−6	−8	12.28	142.49
−9	−18	−6	−39.22	141.97
−11	−16	−7	0.00	126.38
−5	−24	−2	71.45	114.36
−5	−26	0	100.37	144.74

-9	-22	-5	-60.48	138.93
-6	-27	0	-47.28	133.78
-8	-26	-3	56.52	131.66
-7	-28	0	-95.74	123.08
-8	-29	0	88.74	123.74
-15	-31	4	50.98	133.51
-14	31	0	121.10	97.99
-10	25	1	106.44	89.67
-17	32	5	-21.13	135.63
-7	15	5	51.50	83.59
-3	9	3	158.74	64.84
-14	20	8	-76.33	134.17
-13	18	8	15.32	135.36
-12	16	8	-45.96	141.70
-15	11	10	-9.51	128.63
-18	10	11	0.00	137.08
-8	-1	-7	104.72	88.74
-14	1	-9	2.38	123.87
-14	-2	-9	-9.24	123.87
-3	-14	-4	233.88	89.14
-13	-5	10	38.43	94.82
-1	-18	-1	8.32	94.95
-1	-19	0	48.20	83.73
-17	-4	11	-27.47	116.35
-8	-21	-5	32.88	152.27
-7	-25	-3	-137.61	145.00
-9	-27	-3	-137.08	129.95
-10	-30	-1	96.67	139.85
-17	34	3	14.66	152.27
-11	26	-1	208.13	85.18
-12	27	3	105.52	97.33
-12	26	4	-4.49	94.69
-10	21	5	145.66	100.10
-9	19	5	191.88	99.97
-8	17	5	10.56	88.22
-2	3	4	167.59	59.69
-10	9	-7	-0.79	66.95
-1	-7	-3	235.60	62.73
-11	3	-8	-8.19	103.67
-5	-6	7	30.11	58.11
-13	1	10	0.00	112.25
-17	5	11	59.82	132.85
0	-14	-1	1279.41	135.23
-4	-11	-5	34.73	81.75
-8	-7	-7	33.54	99.57
0	-16	1	407.01	81.48
-10	-7	9	48.47	78.18
-5	-15	-5	147.12	98.25
-14	-9	10	18.36	87.82
-8	-17	-6	7.40	140.91
-10	-15	-7	0.00	136.68
-3	-23	2	246.16	85.84
-4	-23	-2	-35.92	101.03
-4	-25	0	93.90	100.10

−6	−24	−3	−54.81	141.17
−9	−30	0	−139.19	128.10
−11	−31	2	18.88	139.32
−18	36	0	19.41	147.38
−18	35	−2	93.90	143.55
−17	33	4	343.75	143.68
−15	30	−3	90.46	91.12
−15	27	6	−160.59	158.74
−6	8	−5	189.51	55.86
−16	13	10	0.00	134.17
−19	12	11	0.13	135.49
−1	−10	−3	73.82	74.22
−14	−5	−9	0.79	128.23
−4	−18	−4	−2.51	95.22
−15	−9	−9	57.71	133.65
−7	−20	−5	−0.13	146.19
−5	−23	−3	34.60	118.33
−8	−25	5	−23.24	89.93
−9	−26	5	−83.99	94.82
−14	−32	3	89.01	158.61
−20	38	−1	117.40	131.40
−20	38	2	−89.80	124.93
−16	33	−1	−3.57	120.97
−11	24	4	34.73	97.20
−4	13	1	330.15	65.11
−3	10	2	3785.53	321.30
−1	1	−2	375.19	48.47
−7	7	7	301.50	83.20
−9	7	8	82.01	102.88
−12	8	−8	−40.67	86.90
−13	−2	10	−23.11	101.42
−6	−12	−6	−33.68	95.22
−11	−9	−8	−7.26	141.44
−10	−27	5	−54.54	101.42
−10	−31	0	17.30	134.31
−19	37	1	−2.38	135.10
−15	32	1	−115.03	108.55
−16	33	2	139.72	150.95
−11	25	3	111.06	99.97
−16	30	5	68.14	156.49
−5	12	4	162.04	92.18
−9	12	−6	189.11	51.37
−17	15	10	−68.41	142.23
−12	9	9	0.00	125.85
−2	−4	5	66.29	55.86
0	−7	−2	7926.83	652.25
−2	−7	−4	9.90	82.93
0	−10	−2	4256.99	359.73
−10	−1	9	0.00	103.40
−17	2	11	−134.44	135.23
−8	−10	−7	0.00	115.95
−17	−1	11	−81.88	126.65
−1	−20	1	66.82	71.97
−2	−20	3	3.83	70.12

−11	−11	9	−14.66	64.18
−3	−22	−2	−18.75	99.84
−3	−24	0	−61.94	94.69
−6	−25	4	−9.38	90.46
−7	−24	5	−3.96	85.05
−8	−25	−4	57.05	130.34
−6	−28	1	−6.07	125.99
−9	−26	−4	109.08	132.33
−9	−28	4	−44.90	104.86
−12	−32	2	0.00	139.06
−13	28	−2	0.92	81.61
−10	22	4	209.05	97.33
−14	25	6	0.00	150.81
−3	6	−3	721.58	64.71
−1	−4	−3	317.21	64.18
−2	−10	−4	7.26	90.86
−10	−4	9	93.37	91.91
−1	−17	−2	52.43	103.54
−2	−17	−3	−5.15	89.01
−7	−16	−6	0.40	142.89
−9	−14	−7	−64.45	161.25
−6	−19	−5	49.39	126.25
−12	−13	−8	−0.13	124.53
−4	−22	−3	148.96	101.16
−7	−24	−4	99.18	146.46
−5	−27	1	0.00	134.31
−7	−29	1	5.02	117.14
−11	−28	5	95.74	107.10
−13	29	0	48.07	90.86
−16	31	4	28.79	155.83
−10	24	−1	192.94	78.97
−10	24	2	60.48	87.69
−5	15	1	425.24	77.92
−6	14	4	24.17	96.14
−4	11	3	1427.98	146.85
−7	11	6	446.76	99.31
−4	6	−4	130.48	40.54
−4	6	5	29.32	83.99
−6	2	7	10.17	75.27
−8	2	−7	213.54	83.99
−8	2	8	39.35	92.57
−18	7	11	25.49	139.06
−10	−2	−8	106.84	117.27
−10	−5	−8	−1.06	137.21
−5	−17	6	13.87	43.45
−14	−8	−9	−178.28	136.02
−2	−22	−1	188.98	92.84
−8	−30	1	−68.01	123.87
−10	−29	4	0.13	110.93
−21	39	0	28.92	124.14
−20	37	3	−26.41	120.70
−16	32	3	18.88	147.25
−10	23	3	93.10	90.33
−7	19	1	365.41	93.63

−6	17	1	357.62	91.65
−9	20	4	112.38	89.93
−13	23	6	111.99	127.97
−13	11	9	13.60	124.53
−1	−13	−3	−97.86	90.20
−4	−14	−5	239.16	104.46
−2	−22	2	139.72	81.35
−6	−23	−4	191.49	148.04
−4	−26	1	134.04	124.01
−7	−28	3	10.70	104.20
−9	−31	1	171.42	136.95
−22	40	1	0.00	124.80
−9	22	2	76.60	85.05
−15	28	5	95.48	148.30
−8	18	4	94.95	89.93
−7	16	4	109.74	95.35
−16	25	7	80.03	138.53
−4	12	2	108.82	64.18
−8	13	6	199.68	92.31
−8	9	7	−14.92	89.14
−10	9	8	14.39	111.86
−11	4	9	−34.07	119.12
−15	8	10	79.10	126.65
−5	−5	−6	48.60	82.41
0	−13	−2	350.23	85.71
−5	−8	−6	68.67	89.93
−13	−1	−9	85.31	129.82
−13	−4	−9	−8.19	129.95
−3	−17	−4	88.48	96.14
−11	−12	−8	111.99	129.02
−2	−23	0	88.61	88.48
−5	−24	4	73.95	87.42
−6	−27	3	0.00	98.39
−9	−24	6	0.00	81.22
−10	−25	6	−23.77	90.99
−11	−30	4	3.17	114.50
−12	−29	5	64.05	115.95
−24	42	0	13.07	135.76
−17	34	0	2.77	142.63
−14	30	1	2.64	96.40
−15	31	2	−96.67	113.04
−9	21	3	−43.71	82.93
−12	21	6	−0.13	104.86
−5	13	3	202.05	82.93
−1	4	1	715.64	76.33
−1	3	2	461.69	61.54
−14	13	9	73.82	121.36
−4	1	−5	413.22	82.01
1	−8	0	702.96	75.14
1	−11	0	333.85	56.92
0	−12	3	644.06	69.33
−2	−13	−4	135.63	91.65
0	−18	0	371.75	91.78
−10	−8	−8	141.44	152.40

−8	−13	−7	28.26	141.31
−2	−21	−2	−12.02	94.69
−5	−18	−5	129.16	106.84
−3	−21	−3	170.09	108.55
−5	−22	−4	66.69	134.83
−3	−25	1	118.99	95.74
−9	−21	−6	−24.17	131.40
−5	−26	3	2.51	97.86
−13	−21	8	34.20	67.75
−11	−26	6	−109.21	98.25
−9	−30	3	24.17	113.04
−10	−32	1	−61.41	145.00
−19	36	2	−74.09	141.57
−14	28	−3	166.53	80.82
−9	22	−1	88.48	78.44
−8	20	2	0.00	93.90
−11	19	6	29.32	96.40
−10	17	6	378.49	103.40
−9	15	6	124.67	99.71
−17	19	9	−97.73	127.84
−15	15	9	−6.07	120.44
−5	4	6	11.09	73.16
−9	7	−7	34.47	73.29
−6	3	−6	239.56	71.84
0	−6	3	257.78	28.00
1	−9	1	61.41	37.51
−10	1	−8	0.00	104.20
−19	9	11	−77.26	148.83
−14	3	10	19.41	119.78
−7	−6	−7	8.19	93.76
0	−17	−1	319.98	98.39
−13	−7	−9	−2.38	132.99
−6	−15	−6	24.30	110.67
−8	−20	−6	−40.28	141.57
−18	35	1	1.06	152.13
−19	36	−1	−0.26	143.15
−15	31	−1	26.15	94.69
−17	33	−2	0.00	129.68
−12	27	0	48.20	85.97
−12	26	−2	128.50	81.09
−15	29	4	67.35	134.44
−15	28	−4	25.88	79.37
−14	26	5	141.83	125.33
−8	19	3	54.67	97.73
−5	14	2	19.81	67.62
−6	15	3	151.08	94.29
−15	23	7	57.18	148.70
−17	23	8	6.74	125.46
−9	11	7	191.75	94.82
−16	17	9	−62.07	124.14
−11	11	8	190.17	131.40
0	−4	−2	3326.75	279.57
−4	−1	6	201.39	64.97
−11	6	−8	17.04	90.59

−3	−7	−5	327.91	103.67
−7	−3	−7	406.48	96.54
−18	4	11	−9.38	129.68
−5	−11	−6	122.29	101.55
−7	−9	−7	−45.83	103.27
−8	−23	6	0.00	78.44
−8	−24	−5	76.20	137.48
−12	−27	6	5.15	105.91
−7	18	2	158.87	92.71
−6	16	2	312.72	90.33
−7	17	3	189.38	96.14
−14	21	7	0.00	148.44
−16	21	8	−107.10	131.00
−5	8	5	150.29	88.74
−16	10	10	−1.32	133.38
−5	−2	−6	−16.77	75.67
−20	11	11	66.43	150.81
1	−12	1	61.01	46.62
0	−17	2	540.39	90.99
−2	−18	4	95.61	58.24
−10	−11	−8	−120.18	133.65
−1	−21	−1	65.24	83.86
−4	−21	−4	113.97	112.12
−9	−17	−7	93.50	137.21
−4	−25	3	37.77	91.12
−6	−28	−2	39.09	134.83
−6	−29	−1	−156.10	147.12
−7	−29	−2	−25.88	132.19
−8	−30	−2	−29.19	133.91
−10	−31	3	−99.31	125.06
−12	−31	4	76.33	124.80
−13	−30	5	9.77	126.78
−19	35	3	66.16	139.46
−19	34	4	0.13	133.25
−8	20	−1	237.58	80.16
−10	13	7	59.69	95.22
−3	5	4	544.09	81.48
−12	13	8	144.34	138.80
−2	−7	5	124.40	49.13
−8	−1	8	16.24	90.07
−1	−16	−3	−91.52	94.82
−18	1	11	42.39	132.72
−14	−6	10	17.70	97.86
−18	−2	11	35.92	127.97
−7	−19	−6	−25.09	156.49
−4	−23	4	−29.85	79.24
−7	−23	−5	81.88	141.44
−5	−28	−1	0.00	138.66
−7	−28	−3	42.26	131.93
−7	−30	−1	16.24	124.67
−22	39	2	35.92	112.25
−13	24	5	59.69	105.91
−17	28	6	12.02	141.04
−5	14	−1	74.75	51.24

−13	19	7	−15.98	132.72
−15	19	8	95.88	144.21
−14	17	8	−19.15	139.85
−13	15	8	−25.75	149.36
−9	4	8	78.18	102.08
−17	12	10	86.37	141.04
−12	6	9	58.37	124.27
−3	−4	−5	916.24	120.84
−14	0	10	165.87	114.89
0	−19	1	140.78	79.37
−4	−17	−5	186.07	100.10
−2	−20	−3	100.63	99.97
−1	−22	0	60.88	83.20
−13	−10	−9	10.83	135.76
−15	−10	10	−36.45	90.07
−2	−24	1	104.99	85.71
−11	−15	−8	44.11	132.06
−4	−27	−1	13.21	147.51
−12	−20	8	0.00	54.15
−6	−27	−3	15.32	147.12
−6	−29	2	−35.92	115.42
−8	−31	−1	−1.98	146.72
−13	−28	6	−29.71	101.03
−11	−32	3	25.22	138.27
−20	37	0	90.86	138.93
−13	28	1	71.84	92.57
−14	29	2	50.98	94.56
−14	27	4	−90.59	117.27
−18	31	5	43.58	143.81
−7	18	−1	459.44	83.59
−2	7	0	0.00	47.15
−8	10	−6	−36.18	52.16
−7	4	7	196.11	83.07
−18	14	10	105.52	139.85
1	−10	−1	97.73	53.35
−11	1	9	153.85	117.53
1	−14	0	248.01	66.82
−9	−4	−8	92.84	126.25
0	−16	−2	356.04	100.89
−3	−13	−5	−38.96	93.63
−2	−16	−4	64.97	96.67
−7	−12	−7	246.82	134.57
−1	−21	2	28.79	73.16
−5	−22	5	−47.01	73.56
−6	−22	−5	2.11	149.36
−5	−26	−3	−15.85	146.59
−5	−28	2	−57.45	107.63
−7	−30	2	−80.29	120.31
−13	−32	4	28.92	137.08
−21	38	1	0.00	132.46
−22	39	−1	14.39	123.74
−11	25	0	93.10	83.73
−14	28	3	14.26	101.29
−6	16	−1	31.56	60.48

−2	4	3	705.87	78.71
−6	−1	7	0.00	73.95
−7	0	−7	549.64	101.42
−15	5	10	20.47	126.91
−19	6	11	71.45	145.27
−12	−3	−9	−14.26	131.53
−9	−7	−8	24.96	148.30
−12	−6	−9	26.15	127.70
−8	−16	−7	−2.11	147.25
−5	−29	0	0.00	123.21
−6	−30	0	45.03	126.12
−7	−31	0	8.98	123.08
−16	32	0	1.19	106.84
−18	34	2	21.39	157.15
−11	24	−2	155.17	74.22
−12	22	5	135.10	96.93
−16	26	6	151.21	160.59
−6	10	5	228.07	96.80
0	−2	2	84.25	30.11
−2	−1	−4	83.07	54.67
1	−13	−1	297.01	67.22
−1	−19	3	80.43	64.05
−3	−17	5	73.95	46.35
−3	−20	−4	83.07	96.40
−6	−18	−6	0.00	147.25
−3	−24	3	−1.72	83.20
−3	−25	−2	36.85	119.78
−3	−26	−1	176.04	127.97
−4	−25	−3	−58.90	139.46
−4	−27	2	22.85	101.16
−4	−28	0	99.18	142.63
−7	−27	−4	108.69	133.25
−9	−32	2	−176.96	144.74
−23	40	0	128.63	136.55
−17	33	1	41.07	130.48
−20	36	−2	−200.34	136.55
−14	29	−1	5.02	88.22
−18	32	4	−77.65	164.55
−13	26	−3	57.84	74.48
−5	6	−5	346.13	66.95
−6	6	6	44.64	75.27
−13	8	9	119.52	126.25
−10	4	−8	171.68	97.59
−9	−1	−8	−10.04	106.97
−12	0	−9	54.81	133.78
−9	−10	−8	−3.30	151.87
−9	−14	8	0.00	42.13
−10	−14	−8	−0.13	125.72
−5	−21	−5	−55.47	146.98
−7	−22	6	−67.09	71.31
−6	−26	−4	−29.98	134.17
−8	−32	0	84.39	135.36
−12	−33	3	−56.79	154.25
−16	31	−2	−11.23	94.03

−18	33	3	87.95	155.04
−13	25	4	16.11	103.40
−11	20	5	8.32	94.95
−18	26	7	0.00	130.61
−7	12	5	159.93	88.48
−1	−1	−3	199.68	46.22
−8	5	−7	42.13	73.56
−8	−4	8	−32.09	98.78
1	−15	1	187.26	67.35
−1	−14	4	9.51	40.41
−11	−8	9	−7.26	81.22
−12	−9	−9	68.54	130.21
−18	34	−1	0.00	139.85
−12	26	1	205.35	93.50
−17	29	5	0.00	156.76
−15	24	6	0.13	135.49
−10	18	5	114.89	98.12
−10	6	8	117.40	108.42
1	−7	−1	243.78	55.07
0	−9	−3	139.46	66.95
−1	−9	−4	−6.60	85.44
−4	−7	−6	195.85	89.54
0	−12	−3	175.24	87.95
−20	8	11	10.43	147.64
−1	−12	−4	138.14	95.74
−4	−10	−6	329.62	102.88
−6	−8	−7	14.13	97.06
−8	−7	8	−1.98	99.31
−3	−16	−5	55.86	102.35
0	−20	−1	49.65	79.50
−7	−15	−7	57.97	156.89
−1	−23	1	−18.09	85.31
−2	−24	−2	−35.52	100.23
−3	−24	−3	8.98	122.82
−3	−26	2	−25.49	97.59
−3	−27	0	43.18	129.42
−5	−25	−4	69.20	161.25
−10	−33	2	−42.39	145.66
−21	37	2	0.00	133.65
−10	23	0	122.82	87.03
−13	26	3	21.13	97.86
−14	26	−4	17.70	68.54
−9	16	5	85.71	86.76
−8	14	5	−24.96	79.24
−4	7	4	1264.22	133.12
−5	1	−6	19.55	69.07
−14	10	9	−51.90	122.55
−6	−5	−7	81.35	90.86
−19	3	11	−1.06	134.70
−1	−19	−3	47.94	90.59
−5	−17	−6	−4.49	124.27
−4	−20	−5	35.39	119.52
−12	23	4	−30.64	97.33
−17	24	7	−16.24	137.08

−8	6	7	254.61	90.86
−21	10	11	−0.13	154.78
−15	2	10	−0.40	122.82
1	−16	−1	118.99	87.29
1	−17	0	−3.96	83.86
−11	−5	9	0.00	89.67
−6	−11	−7	−3.70	108.82
−2	−19	−4	−13.73	95.74
−9	−13	−8	50.18	131.14
−3	−22	4	69.73	70.92
−4	−24	−4	−44.24	146.06
−7	−22	−6	−80.95	142.63
−11	−34	2	−97.33	153.19
−13	−34	3	−3.57	166.26
−19	35	0	96.67	151.47
−20	36	1	62.60	152.66
−17	32	2	43.32	127.44
−21	36	3	0.00	146.85
−17	30	4	71.31	144.87
−14	22	6	−3.43	124.80
−3	8	−2	804.12	69.99
−18	18	9	48.20	125.46
−17	16	9	−14.39	119.91
−2	0	4	529.17	68.67
−16	14	9	−0.13	119.78
−15	12	9	15.19	124.93
−3	−1	−5	169.04	77.92
−1	−6	−4	54.41	71.71
−4	−4	−6	63.26	78.18
−4	−4	6	28.79	54.15
−17	9	10	−162.70	135.10
−12	3	9	1.58	118.85
0	−15	−3	192.68	105.12
0	−15	3	61.01	57.71
0	−19	−2	199.28	91.39
−19	0	11	9.77	133.25
−12	−12	−9	−6.60	129.02
−2	−26	0	−23.24	101.42
−5	−30	1	−79.37	115.42
−6	−31	1	22.05	123.08
−15	30	0	−23.77	90.99
−10	22	−2	80.43	67.22
−16	27	5	−113.97	144.08
−19	29	6	72.77	141.04
−18	22	8	27.86	129.95
−7	8	−6	0.66	53.48
−11	8	8	122.42	114.10
−9	1	8	40.67	96.27
−8	−6	−8	−20.73	133.78
−1	−15	−4	62.46	92.44
−11	−5	−9	0.00	130.61
−11	−8	−9	43.58	127.57
−2	−23	−3	17.17	108.95
−4	−29	1	−21.66	130.61

−7	−32	1	−83.07	136.42
−21	37	−1	70.39	131.27
−16	31	1	6.74	104.46
−13	27	−1	85.44	84.78
−11	24	1	40.67	78.71
−9	21	0	277.86	89.93
−12	24	3	33.54	97.73
−20	32	5	−156.49	132.06
−11	21	4	184.36	100.89
−13	20	6	1.06	101.95
−16	22	7	12.28	157.94
1	−5	0	978.70	89.93
−5	1	6	75.01	68.14
1	−6	1	999.83	89.54
0	−6	−3	17.04	54.01
−9	2	−8	96.14	96.93
−18	11	10	0.00	141.44
−6	−2	−7	86.63	85.84
−6	−4	7	80.03	66.29
1	−12	−2	207.20	66.95
−11	−2	−9	105.78	128.36
−2	−12	−5	216.18	87.69
−8	−9	−8	−16.77	142.49
−6	−14	−7	−10.83	141.04
−15	−7	10	29.19	98.78
−1	−23	−2	−89.80	103.54
−12	−12	9	−15.72	71.84
−2	−23	3	−13.07	73.69
−2	−25	2	70.65	87.69
−6	−21	−6	−3.04	142.10
−6	−25	−5	−43.45	138.40
−12	−25	7	116.08	93.63
−8	−33	1	135.63	141.04
−23	39	1	−22.71	130.21
−17	31	−3	4.49	97.33
−5	9	4	515.70	89.01
−2	5	2	414.28	62.46
−17	20	8	135.36	130.87
−9	8	7	178.55	89.41
−19	13	10	−141.57	142.89
1	−9	−2	1887.81	170.75
−20	5	11	−14.39	139.85
−3	−19	−5	89.01	100.89
−3	−23	−4	70.65	121.10
−3	−28	1	6.07	138.27
−11	−24	7	−72.37	84.65
−6	−31	−2	−58.64	132.72
−22	38	0	−10.83	132.19
−12	24	−3	29.98	70.12
−10	19	4	36.18	86.76
−3	9	0	447.16	60.75
−12	18	6	63.52	102.61
−15	20	7	−21.92	142.76
−2	6	1	792.10	86.90

−3	6	3	669.95	79.37
−12	10	8	5.28	125.85
−7	3	−7	279.31	82.67
−20	15	10	120.97	141.04
−2	−6	−5	300.70	97.99
−8	−3	−8	−53.48	108.69
1	−15	−2	44.50	83.46
−15	−1	10	−28.92	113.18
0	−20	2	89.01	68.54
−4	−16	−6	23.77	114.76
−8	−12	−8	78.71	148.17
−11	−11	−9	−81.22	132.59
−4	−21	5	−19.81	64.97
−9	−16	−8	−29.45	137.21
−1	−25	0	95.61	92.18
−5	−24	−5	183.56	151.74
−5	−30	−2	−132.06	139.85
−9	−34	1	−106.18	145.40
−25	41	0	129.95	131.93
−17	32	−1	6.60	103.93
−19	34	−2	28.26	139.98
−20	35	2	−76.86	150.81
−15	29	−2	0.00	81.48
−16	28	4	6.87	115.55
−15	25	5	−46.09	119.91
−18	27	6	47.01	145.93
−11	16	6	344.15	101.95
−10	14	6	185.15	103.93
−9	12	6	149.76	90.46
−16	18	8	47.01	131.14
−3	4	−4	426.95	57.71
−15	16	8	124.40	160.59
−13	12	8	23.77	138.66
−7	1	7	0.00	74.35
−13	5	9	−1.98	118.19
−16	4	10	16.64	122.95
−2	−15	−5	111.20	96.14
−15	−4	10	0.79	101.82
0	−22	1	75.14	79.76
−5	−20	−6	12.15	153.72
−4	−29	−2	111.99	141.57
−5	−31	−1	3.04	124.27
−7	−29	4	126.65	101.16
−6	−32	−1	−80.69	129.02
−8	−30	4	1.85	107.76
−16	30	2	165.47	110.54
−20	33	4	17.70	142.76
−11	23	2	135.76	87.69
−8	19	0	267.56	85.18
−9	20	−2	105.78	76.73
−11	22	3	62.60	88.88
−14	24	−5	203.37	59.56
−9	17	4	54.94	87.42
−6	11	4	373.20	96.67

−10	10	7	164.02	95.74
−14	14	8	201.26	152.66
−1	−1	3	247.75	42.66
−21	7	11	−187.66	155.57
0	−18	−3	137.74	89.01
−1	−18	−4	−25.36	97.46
−1	−22	−3	91.91	106.71
−4	−28	−3	10.56	136.15
−4	−30	−1	−25.49	128.76
−9	−31	4	−5.15	111.33
−20	34	3	17.83	150.55
−16	29	3	5.81	105.52
−19	30	5	0.00	147.64
−8	15	4	217.77	97.73
−7	13	4	232.82	94.82
−11	12	7	−13.07	86.63
−3	−2	5	540.92	80.03
1	−10	2	100.50	34.34
−11	1	−9	−22.32	143.29
−5	−10	−7	104.46	101.69
1	−19	−1	−15.05	93.90
−20	2	11	−2.38	135.76
0	−23	−1	257.78	94.69
−6	−17	−7	131.93	149.23
−2	−22	−4	81.61	107.63
−4	−23	−5	42.79	154.51
−6	−21	6	−33.41	64.31
−2	−27	1	63.26	116.48
−6	−28	4	73.03	94.29
−7	−31	3	98.91	109.08
−18	33	0	0.13	120.44
−19	34	1	−128.36	152.53
−23	38	2	−76.99	127.31
−14	28	0	23.51	88.61
−14	23	5	107.50	101.16
−12	14	7	5.55	93.76
−3	−9	−6	230.71	92.71
−5	−7	−7	232.69	95.35
−3	−12	−6	149.23	96.27
−10	−7	−9	−4.75	127.17
1	−20	0	137.74	88.22
0	−22	−2	218.30	98.65
−8	−15	−8	−1.72	125.85
−3	−27	−3	157.68	152.27
−3	−29	−1	−79.63	134.44
−5	−28	−4	21.13	129.02
−10	−23	7	18.62	70.92
−5	−29	3	−19.41	103.01
−8	−28	5	−80.29	98.25
−9	−29	5	0.00	94.42
−8	−32	3	53.62	119.38
−10	−32	4	−48.73	119.52
−15	29	1	65.11	95.35
−12	25	−1	4.62	76.46

−13	24	−4	37.11	63.13
−10	20	3	−19.68	88.08
−17	25	6	28.13	148.70
−4	4	−5	1165.17	119.38
−5	5	5	652.38	99.31
−4	−1	−6	62.20	71.58
−10	3	8	31.96	102.08
−14	7	9	269.80	126.78
−17	6	10	73.56	131.80
−6	−7	7	49.92	57.45
−22	9	11	12.28	155.70
−10	−4	−9	249.60	137.61
1	−18	−2	87.69	97.99
−5	−13	−7	35.66	116.35
−2	−18	−5	45.16	95.08
−1	−24	2	22.98	78.31
−10	−30	5	61.41	111.59
−9	−33	3	42.00	125.06
−10	21	2	238.77	94.42
−15	26	4	−106.44	106.04
−4	11	0	901.32	90.46
−19	25	7	−9.51	133.25
−4	8	3	425.24	74.48
−6	6	−6	325.00	65.37
−8	0	−8	32.09	98.39
−9	−2	8	0.40	98.91
−7	−8	−8	17.83	138.27
−10	−10	−9	151.34	134.04
−20	−1	11	−49.79	133.25
−4	−19	−6	79.50	139.59
0	−24	0	101.55	92.05
−2	−28	−1	49.52	140.25
−4	−27	−4	87.42	148.70
−4	−28	3	15.19	96.14
−7	−27	5	−135.10	95.35
−4	−31	0	−130.61	126.38
−5	−32	0	−15.05	129.95
−6	−33	0	−14.53	130.87
−20	35	−1	−49.65	142.10
−22	37	1	78.58	142.10
−5	13	0	596.92	74.35
−20	19	9	59.69	139.59
−6	3	6	252.10	76.46
−10	7	−8	221.86	84.52
−3	−6	−6	−1.06	82.93
0	−11	−4	208.52	93.50
−4	−7	6	390.50	62.20
1	−16	2	261.08	72.63
−6	−10	7	13.73	52.16
−7	−11	−8	−0.13	145.27
−3	−22	−5	117.40	142.49
−1	−26	1	−55.33	96.01
−2	−26	−3	−83.46	138.40
−7	−34	0	−71.58	150.02

−11	−31	5	−1.58	116.08
−10	−34	3	102.48	141.97
−11	−33	4	92.31	133.25
−25	40	1	6.07	126.38
−19	33	2	284.33	162.83
−15	28	2	62.07	92.44
−19	31	4	−5.81	154.91
−8	18	−2	32.75	67.35
−18	28	5	21.26	146.59
−13	21	5	25.09	92.97
−3	7	2	2270.13	197.04
−19	17	9	81.75	125.06
−6	1	−7	45.96	78.31
−15	9	9	−9.64	122.55
−5	−4	−7	−17.83	85.31
−7	−5	−8	139.19	115.95
0	−14	−4	598.90	113.18
−16	1	10	52.43	122.55
−21	4	11	61.14	140.78
0	−21	−3	338.74	102.48
−1	−22	3	50.32	71.97
−2	−21	4	116.35	64.58
−10	−13	−9	61.54	130.61
−3	−30	0	73.16	134.83
−5	−31	2	−31.69	116.35
−7	−33	2	29.45	132.33
−21	36	0	−36.45	145.40
−16	29	−3	123.34	86.37
−15	27	3	0.00	93.10
−11	22	−3	137.74	62.99
−9	19	2	333.32	100.89
−16	23	6	61.94	140.51
−5	10	3	721.71	92.71
−18	15	9	−80.43	123.74
−17	13	9	−80.56	123.21
1	−6	−2	1308.33	122.29
−2	−3	−5	609.86	99.44
−8	3	7	107.63	80.16
−16	11	9	151.74	121.10
1	−11	−3	69.99	71.31
−10	−1	−9	156.36	140.25
1	−14	−3	0.00	92.31
−12	−3	9	−53.75	96.01
−5	−16	−7	50.45	146.46
−1	−21	−4	−3.43	98.91
−1	−26	−2	−13.47	111.20
−5	−23	−6	0.00	140.91
−3	−26	−4	0.00	145.40
−4	−30	2	−44.37	108.95
−8	−34	2	−162.04	145.00
−23	38	−1	−13.47	123.74
−24	39	0	−39.62	121.23
−16	30	−1	50.58	90.07
−13	26	0	58.24	84.78

−14	27	−2	−17.70	78.71
−8	18	1	31.30	81.09
−8	16	3	381.00	100.50
−12	19	5	277.46	97.73
−3	8	1	35.13	52.30
−18	23	7	109.21	141.83
−19	21	8	7.40	121.23
−6	7	5	24.04	86.24
−11	5	8	1.58	103.27
2	−13	0	72.77	53.09
−1	−11	−5	128.89	87.16
−1	−14	−5	−13.21	88.61
0	−18	3	57.97	55.07
−7	−14	−8	−4.23	135.36
−1	−27	−1	−13.21	119.12
−3	−27	3	0.00	96.14
−4	−26	4	4.23	88.35
−2	−29	0	0.00	138.27
−6	−26	5	18.75	85.58
−11	−35	3	61.80	155.44
−18	32	−2	87.69	114.10
−22	36	2	156.23	140.78
−14	27	1	54.54	93.76
−22	34	4	153.98	143.42
−14	24	4	197.43	96.14
−7	14	3	1638.75	167.06
−6	12	3	404.11	81.35
0	−1	−2	304.93	49.92
−3	2	4	113.44	53.88
−21	14	10	26.41	147.25
0	−8	−4	32.62	83.73
−20	12	10	0.00	140.91
2	−12	−1	641.16	81.48
−13	2	9	32.09	117.93
2	−15	−1	232.69	77.39
0	−17	−4	369.90	98.25
−1	−17	4	2.38	46.09
1	−21	1	20.21	73.95
1	−22	−1	78.05	86.76
−3	−18	−6	49.52	123.74
−12	−9	9	12.02	81.48
−16	−8	10	10.70	97.06
−1	−25	−3	−20.87	112.65
−3	−29	2	−31.17	101.03
−9	−35	2	−120.70	151.21
−18	32	1	−29.32	111.99
−11	23	−1	194.00	77.12
−7	16	−2	332.66	65.37
−8	17	2	85.84	88.61
−20	28	6	0.00	135.63
−15	21	6	24.83	104.06
−11	14	−7	152.53	51.50
−10	12	−7	115.42	56.79
0	−3	−3	961.93	95.61

−9	5	−8	157.15	88.61
2	−16	0	111.86	74.35
1	−17	−3	66.29	94.16
−22	6	11	168.91	146.98
−9	−9	−9	141.83	128.50
−16	−2	10	−12.28	107.89
−2	−21	−5	0.00	115.29
−4	−22	−6	−49.79	145.80
−2	−25	−4	0.00	152.93
−4	−26	−5	139.72	143.68
−17	31	0	53.35	96.54
−22	35	3	61.80	136.02
−18	29	4	62.60	134.70
−21	31	5	181.58	141.44
−7	16	1	321.70	79.10
−17	26	5	59.43	126.51
−11	17	5	38.69	89.14
−4	9	2	1453.20	136.02
−17	21	7	49.52	156.49
−7	9	5	73.69	97.59
−18	19	8	8.19	124.01
1	−4	−1	725.15	78.97
−12	7	8	65.50	116.61
−7	−2	−8	−83.46	97.06
−4	−9	−7	−68.67	96.80
−9	−5	8	9.64	100.89
−17	3	10	−0.13	127.70
−9	−6	−9	0.26	129.29
−4	−12	−7	0.00	105.78
−1	−17	−5	190.30	108.95
−12	−6	9	−21.39	89.01
−21	1	11	−18.49	140.25
−14	25	3	−14.66	100.89
−7	15	2	126.12	77.39
−4	10	1	1267.52	122.02
−10	15	5	12.55	81.09
−9	10	−7	−29.19	57.18
−17	17	8	156.36	135.76
−7	5	6	0.92	72.37
−5	−2	6	55.73	58.50
−7	−2	7	65.24	70.52
−23	8	11	91.52	154.12
−12	−8	−10	−48.73	138.40
−9	−12	−9	−5.94	117.01
−16	−5	10	69.99	103.27
1	−23	0	61.14	85.58
0	−23	2	16.24	76.73
−7	−17	−8	139.32	126.78
0	−25	−2	−19.81	101.29
0	−25	1	−35.26	82.80
0	−26	−1	14.00	95.08
−1	−28	0	−7.26	124.80
−9	−22	7	7.53	59.96
−4	−31	−3	257.65	133.38

−4	−32	−2	−5.55	129.82
−4	−32	1	5.28	114.63
−5	−33	1	6.74	125.72
−6	−34	1	−96.93	141.44
−18	31	2	−62.86	111.20
−13	22	4	−21.26	86.90
−6	14	1	142.76	60.09
−5	12	1	618.97	78.05
−6	13	2	31.17	63.52
−4	10	−2	1065.20	93.63
−5	11	2	489.95	75.80
−14	19	6	39.22	99.97
−9	13	5	−3.96	80.03
−8	11	5	114.23	88.22
−2	4	−3	781.40	71.45
−5	4	−6	99.05	61.28
−9	5	7	−4.09	82.67
−3	−3	−6	284.46	79.50
1	−8	−3	148.83	62.33
2	−10	0	0.66	43.45
−2	−11	−6	185.28	91.52
2	−18	−1	33.54	91.65
−6	−10	−8	85.44	146.98
−4	−15	−7	177.09	144.47
0	−20	−4	54.81	97.86
−3	−25	−5	−71.71	144.21
−2	−28	2	12.28	97.46
−3	−31	−2	−17.96	125.72
−10	−28	6	11.09	96.14
−11	−29	6	0.00	102.74
−7	−35	1	63.13	138.93
−21	35	−2	31.83	141.17
−21	35	1	−0.53	146.46
−18	30	3	−31.83	115.82
−6	14	−2	134.83	45.96
−19	26	6	126.65	151.61
−3	2	−5	40.67	63.13
−16	15	8	88.61	141.70
−13	9	8	0.00	124.27
−10	0	8	254.88	106.71
−14	4	9	54.01	113.18
2	−14	−2	81.22	76.20
−9	−3	−9	188.72	140.12
−6	−7	−8	−8.32	126.38
−4	−10	6	227.14	44.24
1	−19	2	104.99	68.80
1	−20	−3	77.92	91.39
−6	−13	−8	213.94	145.80
−9	−11	8	0.00	60.35
0	−24	−3	216.98	98.65
−1	−24	−4	173.66	120.44
−2	−26	3	23.51	85.05
−5	−25	5	32.75	80.43
−3	−31	1	54.54	113.18

−9	−27	6	61.01	92.05
−5	−34	−1	−28.00	137.61
−19	33	−1	101.95	124.53
−24	38	1	30.24	125.06
−12	24	0	−23.77	77.12
−13	17	6	81.22	92.57
−2	2	−4	482.68	64.05
−15	13	8	2.64	139.06
−14	11	8	93.90	134.83
−5	−1	−7	63.52	83.59
−4	−6	−7	52.03	88.74
−18	5	10	115.82	131.27
2	−17	−2	−37.77	90.46
2	−19	0	10.30	93.76
−3	−21	−6	122.82	145.00
−3	−25	4	0.00	83.46
−2	−30	−2	84.52	134.83
−4	−33	−1	0.00	124.67
−20	34	0	69.60	144.21
−13	25	1	133.25	84.52
−10	21	−1	307.04	87.82
−21	32	4	23.11	140.78
−10	20	−3	0.00	57.45
−12	22	−4	193.21	60.88
−12	20	4	16.11	86.10
−16	24	5	30.24	108.55
−5	12	−2	338.21	47.01
−8	8	−7	61.94	64.45
−8	7	6	48.99	78.05
−10	7	7	186.07	86.10
−8	3	−8	−21.92	89.41
2	−9	−1	931.43	94.42
−2	−8	−6	91.39	82.41
2	−14	1	599.69	76.86
−9	−8	8	−15.85	84.25
−2	−17	−6	7.40	113.31
−22	3	11	49.79	141.17
−1	−20	−5	37.24	96.40
0	−27	0	−49.79	99.84
−2	−29	−3	−120.18	143.29
−2	−30	1	−45.69	123.21
−3	−32	−1	15.98	118.19
−15	28	−1	64.71	83.46
−17	30	1	0.00	90.86
−19	32	−3	−128.50	113.18
−21	34	2	53.35	162.83
−13	25	−2	37.51	75.67
−13	23	3	15.45	99.71
−17	27	4	14.13	106.18
−13	22	−5	42.92	49.65
−20	29	5	55.86	164.55
−12	15	6	91.91	99.71
−14	15	7	0.26	106.31
−21	18	9	51.24	133.65

-4	0	5	1082.90	116.35
2	-11	-2	762.92	92.71
-15	6	9	7.79	117.93
-4	-18	-7	108.69	150.29
-6	-16	-8	59.69	127.70
-3	-20	5	4.23	52.96
-2	-24	-5	-102.22	146.72
-3	-29	-4	68.54	124.53
-2	-31	-1	74.48	122.82
-22	36	-1	66.29	135.76
-23	37	0	-35.66	130.48
-26	40	0	30.24	122.55
-21	33	3	-3.96	139.98
-13	24	2	67.35	89.54
-20	24	7	39.49	129.68
-11	13	6	10.70	94.69
-21	22	8	132.59	132.46
-4	4	4	1368.28	132.06
-13	13	7	78.05	101.03
-11	9	7	136.55	85.44
-20	16	9	56.79	118.72
-1	-5	-5	473.83	94.42
-19	7	10	-15.85	131.53
-8	-8	-9	-23.24	130.34
-17	0	10	86.10	118.33
-8	-11	-9	-71.45	121.36
1	-24	-2	-63.39	102.22
-13	-13	9	22.19	67.22
-5	-22	-7	-75.27	123.34
-1	-27	2	66.03	90.07
-4	-25	-6	-46.49	135.10
-1	-29	-2	-203.90	149.36
-8	-26	6	1.45	82.41
-25	39	-1	-48.86	123.08
-16	29	0	74.48	85.05
-24	37	2	38.03	127.97
-15	27	-3	29.19	74.09
-24	36	3	23.77	128.89
-18	24	6	34.73	142.89
-12	11	7	-8.32	87.95
-16	8	9	66.95	114.50
-6	-4	-8	70.26	101.42
1	-13	-4	288.03	98.91
1	-16	-4	104.99	94.95
2	-20	-2	-25.49	81.48
-23	5	11	0.26	141.44
2	-21	-1	-14.66	80.29
-11	-10	-10	-102.35	128.36
-1	-28	-3	-10.04	150.55
-2	-28	-4	-20.21	128.10
-17	30	-2	91.91	86.10
-17	29	2	82.54	95.61
-17	28	3	42.26	106.97
-11	18	4	173.40	92.05

−15	22	5	−25.62	99.44
0	−1	1	873.45	73.16
−7	6	−7	−8.19	66.43
−19	14	9	−85.84	118.85
−18	12	9	11.75	117.80
−17	10	9	104.86	118.19
−22	13	10	88.74	141.97
0	−13	−5	111.86	86.24
0	−16	−5	212.88	109.08
−2	−20	−6	101.95	145.93
−8	−14	−9	−27.86	124.53
−22	0	11	−46.88	147.51
1	−24	1	−59.69	81.09
0	−23	−4	0.00	99.84
−1	−29	1	0.00	132.46
−1	−30	−1	−62.33	136.15
−4	−34	0	−24.96	128.50
−5	−35	0	40.15	140.78
−20	20	8	64.31	128.63
−2	1	3	230.84	44.64
−4	2	−6	80.95	62.99
2	−11	1	643.93	69.20
−9	0	−9	46.22	134.70
−11	2	8	50.18	102.22
−8	−5	−9	−76.20	133.91
−3	−11	−7	103.93	95.88
−3	−14	−7	67.88	119.91
−5	−12	−8	−84.78	148.17
−24	7	11	−30.64	141.70
0	−21	3	179.34	68.28
1	−23	−3	106.18	96.40
−1	−23	−5	−50.98	127.84
−6	−19	−8	−57.97	124.93
−17	−9	10	24.43	97.46
−3	−24	−6	−18.88	124.93
−3	−33	0	65.50	116.87
−20	33	1	93.50	134.70
−11	22	0	213.81	92.31
−20	30	4	−26.15	152.27
−12	21	3	41.07	82.41
−16	25	4	−96.14	95.88
−19	27	5	1.72	145.40
−19	22	7	−86.10	143.55
−7	1	−8	−10.43	86.10
−8	0	7	70.26	71.45
1	−10	−4	−3.43	84.65
−1	−8	4	575.39	55.07
2	−16	−3	73.29	98.91
−5	−9	−8	9.24	130.87
1	−19	−4	−10.04	92.31
−17	−3	10	−31.03	108.03
−1	−25	3	46.75	78.31
−3	−28	−5	25.49	122.02
−5	−32	3	40.28	105.78

−6	−33	3	−50.05	109.08
−7	−34	3	−22.45	114.50
−9	19	−1	405.30	84.39
−10	16	4	39.88	88.22
−17	22	6	−75.01	128.10
−5	6	4	675.76	91.91
−2	−5	−6	91.78	73.95
−4	−3	−7	165.74	86.24
2	−13	−3	5.81	85.18
−18	2	10	−20.60	118.19
2	−20	1	78.44	74.48
0	−19	−5	145.66	101.29
−3	−17	−7	69.33	148.30
−5	−15	−8	−59.96	132.99
1	−26	0	−24.96	88.48
0	−27	−3	0.00	138.53
−1	−27	−4	−76.86	145.93
−2	−32	0	219.62	126.12
−7	−32	4	−26.41	105.52
−8	−33	4	67.09	108.69
−8	−35	3	−67.88	120.97
−23	36	1	−38.96	136.82
−26	39	1	73.16	119.12
−12	22	2	91.12	80.69
−14	20	5	81.88	104.59
−21	27	6	−20.34	141.17
−19	18	8	−58.24	128.36
−6	0	6	245.37	70.52
−3	−5	5	209.71	59.82
−3	−8	−7	319.19	99.71
−10	−3	8	42.92	105.91
−14	1	9	54.28	104.59
−1	−13	−6	14.26	93.50
2	−19	−3	136.95	92.57
−13	−4	9	−1.32	88.61
1	−22	2	20.87	71.45
−23	2	11	20.73	144.08
−17	−6	10	−13.34	88.48
0	−29	−1	−109.87	146.59
−4	−31	3	−4.23	101.16
−6	−31	4	−46.62	101.69
−5	−34	2	−51.24	130.34
−6	−35	2	76.86	129.68
−9	−34	4	−34.20	115.16
−18	31	−1	−14.26	93.10
−19	32	0	−45.96	108.55
−14	26	−1	111.72	81.35
−16	28	1	−14.92	87.16
−20	32	2	0.00	137.87
−23	33	4	−50.98	129.42
−9	14	4	152.66	104.33
−2	0	−5	267.29	74.75
−12	4	8	0.00	110.27
−7	−5	7	45.83	61.01

−10	−9	−10	67.88	134.17
2	−23	−2	18.22	88.74
−1	−20	4	10.30	56.65
0	−26	2	108.69	84.12
−2	−24	4	−13.07	74.09
0	−28	1	−12.55	113.31
−2	−27	−5	11.09	123.48
−1	−31	0	40.94	121.89
−7	−25	6	−19.41	73.56
−7	−36	2	−65.63	136.82
−9	−36	3	−54.41	131.27
−20	33	−2	39.22	130.74
−28	41	0	54.54	126.78
−16	27	−4	4.23	73.95
−16	26	3	141.44	92.84
−9	18	−3	4.23	59.82
−11	19	3	109.87	92.84
−22	30	5	−25.22	132.33
−8	12	4	163.76	88.48
−6	8	4	501.17	86.76
−6	4	−7	−43.98	70.26
−18	16	8	53.22	125.06
2	−8	−2	1465.48	134.97
−1	−10	−6	132.06	86.76
−5	−6	−8	25.88	105.78
−7	−10	−9	1.85	123.48
−7	−13	−9	−16.51	119.65
1	−22	−4	−55.33	99.71
2	−24	−1	172.34	94.56
−5	−18	−8	73.29	122.95
−2	−23	−6	116.08	137.61
−5	−30	4	48.60	94.29
−15	27	0	51.24	85.18
−23	35	2	136.82	138.53
−12	23	−2	204.69	71.31
−23	34	3	158.74	137.08
−16	20	6	33.68	102.61
−7	10	4	1540.89	159.79
1	−5	−3	306.12	57.97
−8	−2	−9	28.13	141.57
−19	4	10	43.45	123.08
−1	−19	−6	13.34	115.55
0	−22	−5	71.97	113.84
1	−27	−2	47.67	107.63
0	−26	−4	17.96	141.04
−3	−30	3	150.42	99.18
−2	−32	−3	−1.98	129.16
−3	−32	2	−69.99	104.33
−8	−37	2	−118.99	154.64
−22	35	0	23.11	143.55
−23	36	−2	−6.47	123.21
−25	38	0	−47.94	120.57
−27	40	−1	−33.28	118.06
−19	28	4	27.20	117.01

−15	23	4	51.90	91.12
−18	25	5	85.97	121.50
−13	18	5	−6.21	90.86
−22	25	7	4.49	119.52
−1	0	−4	102.35	49.39
−17	14	8	135.36	131.40
−22	17	9	−59.69	121.23
−6	−1	−8	−29.05	89.80
−13	6	8	18.09	124.27
2	−10	−3	402.52	83.59
−15	3	9	22.32	105.38
3	−17	−1	676.42	103.40
−7	−7	−9	270.99	131.66
2	−22	−3	0.00	85.84
−13	−7	9	−7.00	80.16
−24	4	11	−21.66	139.19
−13	−10	9	−19.81	75.80
1	−26	−3	19.94	116.74
1	−28	−1	41.20	110.01
−1	−26	−5	68.28	140.25
−2	−33	−2	11.36	119.78
−10	−37	3	−96.80	147.38
−21	34	−1	98.12	137.34
−24	37	−1	21.53	124.01
−10	20	0	289.35	87.29
−11	20	2	45.69	92.71
−20	25	6	107.23	145.80
−3	0	−6	548.98	87.16
−16	12	8	175.64	136.68
−14	8	8	9.77	126.38
0	−7	−5	1482.38	159.66
−9	2	7	24.43	76.33
3	−14	−1	1301.59	129.82
−20	6	10	−8.85	126.12
0	−30	0	120.18	132.59
−2	−31	2	0.00	97.20
−4	−29	4	33.94	90.73
−4	−35	1	−34.73	127.57
−8	−31	5	−28.26	97.86
−5	−36	1	64.58	130.74
−9	−32	5	41.07	103.40
−16	28	−2	−49.65	80.82
−18	30	−3	74.22	89.27
−8	17	−1	82.54	67.48
−11	20	−4	65.90	49.26
−10	17	3	249.99	98.12
−15	18	6	62.20	96.01
−15	10	8	20.60	132.06
−3	−5	−7	13.87	83.20
3	−16	−2	80.69	82.27
−21	8	10	−8.85	134.97
−2	−13	−7	188.19	101.42
−4	−11	−8	−4.89	127.17
3	−20	−1	49.39	94.69

−2	−16	−7	55.73	123.48
−4	−14	−8	9.77	133.65
−18	−1	10	93.50	112.91
−25	6	11	61.14	139.46
2	−25	0	31.56	84.39
−1	−32	−2	−22.98	119.38
−3	−34	1	−20.21	119.38
−3	−35	−1	0.00	122.55
−19	31	1	−55.47	100.50
−14	25	−3	252.10	74.22
−12	16	5	136.82	87.29
−1	0	2	363.43	49.26
−22	21	8	−35.00	117.14
−5	2	−7	55.33	72.90
−7	2	6	131.40	69.46
1	−7	−4	119.91	71.84
−21	15	9	14.79	116.61
−5	−5	6	105.38	55.47
−16	5	9	−26.02	105.91
3	−15	0	387.07	70.92
−22	10	10	−29.98	135.76
2	−15	−4	97.46	90.46
1	−15	−5	41.34	90.33
3	−18	0	54.94	77.65
3	−19	−2	186.47	97.59
1	−18	−5	76.20	92.71
2	−23	1	41.60	76.86
−4	−17	−8	242.20	127.44
−1	−22	−6	28.66	137.21
1	−27	1	−1.32	87.29
−2	−34	−1	18.36	119.38
−7	−30	5	−31.56	90.99
−19	29	3	−14.53	115.03
−22	31	4	−74.09	145.40
−21	23	7	2.25	134.83
−3	3	3	1104.43	104.59
−16	16	7	96.93	113.84
1	−3	−2	441.08	61.01
−20	13	9	26.68	113.70
−1	−7	−6	−36.58	79.10
−2	−10	−7	15.85	86.37
2	−18	−4	77.39	83.07
−9	−11	−10	0.00	127.70
−2	−19	−7	63.92	129.68
1	−25	−4	−12.81	115.69
−3	−23	−7	22.98	123.48
−2	−26	−6	15.85	121.50
0	−30	−3	57.71	129.68
0	−31	−2	53.09	132.72
−1	−30	−4	−3.17	119.52
−2	−29	3	−27.34	91.39
−2	−33	1	−128.23	112.12
−22	34	1	199.41	144.74
−25	37	1	−70.52	123.61

−15	26	1	150.42	89.41
−19	30	2	61.67	96.54
−15	24	3	−104.33	91.25
−14	21	4	258.18	91.12
−21	28	5	67.09	143.55
−17	23	5	−5.15	107.10
−23	28	6	19.81	136.15
−11	14	5	183.83	84.65
−14	16	6	147.38	94.82
−10	4	7	90.99	79.37
−19	11	9	−30.64	111.86
−18	9	9	38.03	114.10
3	−13	−2	271.78	71.97
−17	7	9	117.14	110.80
1	−12	−5	0.00	83.46
−7	−4	−9	54.67	131.00
−11	−1	8	−68.01	110.27
−14	−2	9	79.37	85.97
−9	−8	−10	7.92	121.63
−6	−12	−9	132.85	120.97
2	−26	−2	−13.47	90.73
0	−25	−5	−34.20	141.83
−1	−30	2	−57.45	97.73
−1	−33	−1	14.53	115.42
−18	30	0	80.56	93.63
−10	19	1	375.45	94.42
−10	18	2	120.97	86.50
−9	15	3	106.97	90.46
−19	23	6	349.70	141.83
−15	14	7	−51.24	99.18
−21	19	8	−3.30	124.01
−1	−2	−5	38.03	72.24
2	−7	0	−23.24	45.56
−5	−3	−8	93.37	88.88
−4	−8	−8	30.90	112.65
−6	−9	−9	47.41	121.50
−10	−6	8	−0.26	99.84
3	−21	0	134.17	78.71
0	−18	−6	2.38	96.80
2	−21	−4	71.45	98.12
1	−21	−5	−58.37	98.91
−6	−15	−9	129.68	122.29
−18	−4	10	−7.13	100.37
2	−25	−3	−29.98	93.24
0	−24	3	26.54	68.28
2	−27	−1	144.47	93.63
1	−29	0	60.22	121.89
−1	−32	1	−3.83	106.57
−6	−29	5	−81.75	86.50
−17	29	−1	117.14	83.86
−21	33	−3	0.00	119.78
−13	24	−1	114.23	74.22
−15	25	2	5.02	88.08
−25	35	3	82.27	128.36

−18	26	4	18.49	94.56
−10	12	5	62.33	88.61
−7	6	5	62.46	77.52
−14	12	7	42.26	89.80
−11	6	7	−9.77	82.27
2	−12	−4	22.45	92.97
2	−18	2	133.65	71.31
−19	1	10	36.18	113.04
3	−23	−1	110.40	84.65
1	−25	2	63.92	72.11
−4	−20	−8	19.94	117.93
0	−29	−4	13.21	136.02
0	−32	−1	23.24	116.35
−27	39	0	79.10	121.63
−14	25	0	19.55	75.54
−22	33	2	32.75	140.38
−25	36	2	45.43	135.49
−9	10	5	57.05	87.29
−13	14	6	9.51	84.65
−20	21	7	0.00	119.52
−8	8	5	22.58	87.42
−9	8	−8	−15.98	71.97
−13	10	7	119.52	87.56
−2	−2	−6	153.72	71.05
−8	4	6	107.89	70.92
−12	8	7	199.15	83.99
0	−12	−6	441.88	99.84
3	−18	−3	57.71	84.78
1	−17	3	53.35	55.86
−25	3	11	−12.81	138.00
−2	−22	−7	43.98	119.38
−10	−15	8	2.77	31.17
−18	−7	10	−89.14	101.55
−1	−25	−6	−40.67	129.02
1	−29	−3	12.55	132.46
1	−30	−2	−159.13	136.42
−8	−21	7	21.66	26.41
−1	−29	−5	0.00	118.99
−3	−36	0	190.04	122.68
−19	31	−2	−45.43	95.22
−11	21	−2	183.43	70.12
−7	15	−1	23.51	50.45
−13	19	4	172.60	86.50
−8	13	3	330.55	85.44
2	−6	−1	831.46	88.48
−4	0	−7	116.61	76.33
3	−11	−1	364.49	62.99
3	−15	−3	52.43	89.41
−7	−8	7	−31.17	58.11
0	−21	−6	124.01	141.17
−2	−35	0	34.73	125.99
−21	33	0	−1.19	115.82
−24	36	0	28.66	128.50
−8	16	−3	203.11	53.48

−16	21	5	−61.54	98.65
−4	5	3	347.06	59.30
−12	12	6	33.68	85.18
−8	6	−8	29.32	73.56
−9	6	6	20.34	74.48
−20	17	8	26.15	124.14
−8	1	−9	0.00	125.33
−2	−7	−7	77.92	86.37
−8	−3	7	219.35	71.58
−3	−13	−8	29.45	141.70
−20	3	10	6.07	113.57
3	−21	−3	191.62	91.52
−3	−16	−8	69.46	132.46
2	−24	−4	−14.92	93.90
−1	−28	3	−29.19	87.16
0	−31	1	1.06	113.70
−20	32	−1	58.90	102.88
−26	38	−1	27.73	114.10
−18	27	3	9.51	87.29
−12	20	−5	14.39	33.02
−14	22	3	92.44	86.24
−21	29	4	97.99	133.25
−9	16	2	334.77	82.67
−20	26	5	−18.62	126.78
−7	11	3	1381.89	138.14
−22	26	6	0.00	127.31
−18	21	6	29.05	114.10
0	−2	−4	577.63	74.88
−10	8	6	−5.02	76.73
3	−12	0	498.00	66.69
−12	1	8	79.50	111.59
−6	−6	−9	−24.56	132.19
−1	−15	−7	89.41	105.78
−8	−10	−10	9.77	119.91
1	−24	−5	0.00	122.02
1	−28	−4	33.81	139.19
0	−29	2	−45.03	90.86
1	−31	−1	75.54	125.99
−5	−28	5	−13.07	75.80
−1	−34	0	50.18	106.71
−22	34	−2	−12.15	131.00
−23	35	−1	−6.21	136.29
−18	29	1	58.77	89.93
−9	17	1	100.63	77.12
−24	32	4	0.00	125.85
−6	9	3	52.43	59.96
−5	7	3	477.53	69.73
−7	4	−8	233.48	80.43
−19	15	8	−35.52	117.93
−23	16	9	39.09	121.63
−4	−5	−8	10.43	93.63
1	−14	3	98.78	47.54
−21	5	10	−12.15	118.46
2	−21	2	1.32	61.28

−1	−18	−7	−10.17	131.27
−5	−14	−9	5.41	117.80
−14	−5	9	61.80	86.90
3	−24	0	83.86	80.82
3	−25	−2	0.00	92.57
−3	−19	−8	119.52	120.97
2	−26	1	131.93	77.39
−1	−23	4	−22.19	59.69
2	−28	0	43.32	95.61
−3	−23	5	−5.15	64.05
0	−28	−5	0.00	120.84
−27	38	1	−50.18	122.29
−14	24	1	191.62	81.22
−15	25	−4	−11.23	63.13
−17	24	4	150.29	94.29
−23	29	5	−140.38	135.36
−23	24	7	−30.51	129.02
−3	−1	4	379.41	58.37
2	−7	−3	310.87	66.56
0	−9	−6	−0.53	80.16
−1	−12	−7	46.62	87.82
−3	−10	−8	6.74	118.06
−24	11	10	−24.04	129.42
−23	9	10	99.31	129.42
−22	7	10	2.11	125.46
−5	−11	−9	−46.62	125.33
1	−20	3	35.13	58.24
−19	−2	10	15.85	106.18
0	−24	−6	130.08	139.19
2	−28	−3	106.44	135.49
0	−33	0	−6.60	108.82
−1	−35	−2	−12.68	124.80
−15	26	−2	98.78	76.73
−14	23	2	71.97	82.27
−12	17	4	−51.24	87.03
−18	13	8	−67.22	121.10
−7	−1	−9	38.03	128.23
3	−12	−3	309.68	83.59
3	−16	1	233.09	64.97
2	−17	−5	−8.19	88.61
3	−24	−3	55.60	88.35
0	−33	−3	86.10	116.87
−5	−35	3	−71.45	101.16
−5	−37	2	−82.14	118.85
−6	−36	3	−75.27	108.03
−17	28	−3	248.54	80.82
−21	32	1	−1.06	110.80
−24	35	1	−14.13	132.72
−27	37	2	17.96	116.48
−21	30	3	83.46	116.08
−15	19	5	23.77	89.93
−17	19	6	170.23	97.73
0	−4	−5	899.07	113.97
−3	−2	−7	145.27	79.63

3	−10	−2	3611.34	306.91
2	−9	−4	110.80	82.01
−4	−3	5	1110.10	113.44
−22	14	9	−133.51	115.95
−13	3	8	38.03	113.31
3	−17	−4	142.23	82.01
−16	2	9	74.09	102.35
2	−20	−5	71.18	89.14
2	−30	−1	74.22	140.78
1	−30	1	105.78	120.97
−2	−27	4	−4.75	79.37
0	−34	−2	112.12	119.52
−4	−34	3	−43.32	95.61
−4	−36	2	42.39	118.33
−6	−38	2	−9.38	131.53
−7	−37	3	−38.56	114.50
−17	28	0	35.26	82.54
−29	40	0	3.43	115.03
−12	22	−1	62.99	72.90
−13	23	−3	67.48	62.07
−21	31	2	1.06	122.55
−8	14	2	237.05	76.60
−19	24	5	27.34	103.54
−21	24	6	24.56	135.10
−18	17	7	−3.96	124.93
−23	20	8	−39.09	111.86
−6	2	−8	58.77	79.90
−1	−4	−6	174.58	74.88
−17	11	8	0.00	124.01
−21	12	9	−64.45	109.48
2	−14	−5	230.58	85.58
1	−17	−6	33.41	89.93
3	−20	−4	16.24	87.95
−2	−16	5	82.01	22.32
3	−22	1	77.92	70.26
1	−20	−6	−8.06	104.59
−10	−12	8	56.52	58.37
2	−27	−4	39.35	135.49
1	−27	−5	−29.58	135.23
−3	−35	2	97.06	110.80
−8	−38	3	−92.57	121.50
−16	27	−1	19.55	70.26
−13	23	0	100.63	82.27
−24	34	2	138.27	140.12
−13	20	3	184.75	84.92
−16	9	8	88.35	122.02
−15	7	8	36.85	120.44
−6	−3	6	−7.00	57.31
−14	5	8	−44.90	118.59
−5	−8	−9	−24.43	122.16
−17	4	9	0.00	100.89
4	−18	−2	161.25	97.99
4	−19	−1	36.85	84.25
−11	−4	8	42.52	105.25

2	−23	−5	−15.19	103.14
−14	−8	9	−6.21	79.10
1	−32	0	−29.58	114.63
−3	−33	3	0.00	93.76
−1	−36	−1	61.14	124.40
−7	−35	4	113.31	103.14
−17	25	3	−7.00	88.08
−8	15	1	−38.69	64.31
−20	27	4	52.43	103.67
−2	2	2	143.02	44.90
−22	22	7	34.47	128.36
−1	−9	−7	128.23	83.59
−9	−1	7	0.00	66.16
−20	10	9	48.86	105.65
3	−14	−4	249.86	97.59
−19	8	9	−83.20	107.37
−18	6	9	47.67	101.82
4	−21	−2	75.54	78.97
−2	−15	−8	0.00	139.72
4	−22	−1	20.34	78.44
−7	−12	−10	22.58	123.34
3	−23	−4	−5.94	95.48
−20	0	10	26.41	106.97
−19	−5	10	65.11	100.37
−1	−24	−7	−26.02	116.08
−14	−11	9	38.69	73.69
1	−28	2	42.26	82.80
1	−33	−2	101.29	114.10
−9	−30	6	−2.64	83.46
−3	−37	1	−19.68	124.67
−6	−34	4	47.41	96.40
−4	−38	1	97.99	122.02
−8	−36	4	0.00	96.93
−26	37	0	209.05	120.84
−16	22	4	259.24	100.23
−14	17	5	234.94	93.76
−24	27	6	−70.39	131.66
−16	17	6	−44.50	88.48
−17	15	7	−57.45	108.42
−22	18	8	−32.09	118.85
−6	−3	−9	29.19	128.89
−3	−7	−8	76.07	95.08
4	−16	−1	448.61	88.88
−2	−18	−8	−110.54	119.65
2	−24	2	97.20	73.43
1	−23	−6	91.78	136.42
3	−27	−3	−15.45	106.84
3	−27	0	13.34	82.41
3	−28	−2	−22.05	103.40
0	−27	−6	56.92	114.23
0	−27	3	−27.34	80.29
−19	−8	10	6.34	92.97
1	−31	−4	150.55	124.93
−4	−27	5	0.00	69.86

0	−35	−1	−75.41	113.18
−5	−33	4	106.97	94.29
−20	31	−3	52.16	108.16
−20	31	0	45.03	93.10
−28	39	−1	−86.90	109.74
−17	27	1	21.92	92.57
−10	19	−2	298.85	75.01
−10	18	−4	73.56	41.47
−23	30	4	8.32	143.95
−7	12	2	246.95	66.03
−22	27	5	31.17	142.76
−5	0	−8	26.28	78.05
4	−15	−2	160.72	78.84
−2	−12	−8	54.67	125.59
−7	−9	−10	7.26	110.54
0	−17	−7	−29.98	124.67
−4	−13	−9	22.71	106.71
−7	−11	7	79.76	59.43
−4	−16	−9	0.00	108.69
1	−23	3	−36.71	66.82
−1	−33	2	−13.07	92.05
−10	−26	7	−7.40	63.26
−23	34	0	0.00	128.63
−17	26	2	101.55	90.33
−13	21	2	0.00	82.54
−7	14	0	1482.12	137.48
−25	30	5	83.59	117.01
2	−8	1	1177.06	103.67
−2	−4	−7	82.14	78.18
2	−12	2	222.13	42.13
0	−14	−7	294.89	104.33
−21	2	10	−0.92	107.10
4	−24	−2	109.48	92.84
−2	−21	−8	0.00	108.16
3	−29	−1	106.97	105.91
2	−31	−3	88.08	125.33
−2	−32	3	34.34	91.12
−1	−35	1	−76.99	110.14
−8	−29	6	58.64	76.73
−19	30	−1	158.08	88.88
−12	18	3	84.39	93.90
−18	22	5	−5.94	86.90
−10	13	4	489.68	102.61
−20	22	6	56.65	129.82
2	−5	−2	413.35	59.16
−4	1	4	485.46	68.01
−16	13	7	135.76	97.06
3	−13	1	135.63	49.26
1	−11	−6	104.86	80.29
4	−17	−3	206.54	97.99
4	−20	0	37.77	82.41
4	−23	−3	135.89	87.69
3	−25	1	−26.81	76.46
3	−26	−4	73.43	106.44

2	−26	−5	25.49	130.21
2	−29	1	−10.43	104.59
2	−31	0	28.00	121.89
2	−32	−2	−78.71	118.06
1	−34	−1	21.66	104.06
−4	−32	4	−120.18	89.93
−25	36	−1	−178.02	120.18
−26	36	1	22.98	126.38
−20	28	3	12.41	95.61
−26	34	3	0.00	102.88
−6	10	2	916.77	99.05
−13	15	5	33.28	83.99
−15	15	6	94.95	94.82
−21	16	8	38.83	108.29
−10	1	7	−90.99	73.03
4	−17	0	321.96	78.05
−4	−10	−9	105.12	124.14
−25	10	10	−11.62	120.84
−24	8	10	−19.41	120.04
−22	4	10	−54.41	111.99
4	−25	−1	159.40	89.80
−2	−19	5	−1.45	40.28
0	−23	−7	8.58	117.67
1	−26	−6	−155.57	119.78
2	−30	−4	66.43	131.40
1	−30	−5	−19.94	116.35
−2	−38	0	10.83	111.72
−21	32	−2	16.64	110.01
−22	33	−1	−67.62	122.29
−24	35	−2	−31.17	123.48
−20	30	1	79.24	90.33
−23	31	3	31.96	149.23
−7	14	−3	1287.20	116.87
−7	13	1	3.17	54.54
−15	11	7	−35.92	85.71
1	−6	−5	464.72	89.67
0	−6	−6	264.78	79.63
−4	−2	−8	50.71	80.29
−24	15	9	92.05	115.42
−5	−5	−9	−27.34	127.84
−5	−8	6	77.65	46.49
3	−19	−5	76.46	90.20
−23	6	10	−8.98	115.69
0	−34	1	8.98	100.63
−1	−37	0	138.14	113.04
−18	28	−4	0.53	72.63
−23	33	1	233.35	135.23
−20	29	2	42.26	88.08
−26	35	2	−9.38	115.42
−16	23	3	53.75	90.20
−19	25	4	0.00	91.39
−15	20	4	77.78	90.20
−9	11	4	805.57	113.44
−23	25	6	0.00	127.31

−3	4	2	145.93	50.71
3	−9	−3	210.77	62.73
−2	−9	−8	61.41	104.06
−12	−2	8	26.15	103.01
2	−19	−6	0.00	95.35
3	−22	−5	78.97	90.86
−20	−3	10	25.62	100.50
2	−33	−1	23.51	105.65
0	−32	2	69.73	92.44
−14	24	−2	171.68	69.07
−16	26	0	−12.55	79.37
−12	21	0	95.22	87.29
−23	32	2	−21.79	136.82
−5	8	2	966.69	97.20
−4	6	2	73.69	46.88
−24	23	7	98.52	111.99
−14	9	7	47.54	78.05
−5	−1	5	454.82	75.67
−20	14	8	1.85	115.95
3	−11	−4	4.62	82.41
−7	−1	6	−15.45	59.69
−11	3	7	83.86	74.09
4	−13	−1	232.43	61.41
4	−14	−3	438.84	91.39
−23	13	9	−6.21	109.74
0	−11	−7	190.43	93.37
3	−16	−5	−76.07	82.27
−8	−6	7	3.30	57.18
−6	−11	−10	2.11	110.93
−16	−1	9	12.94	89.93
−1	−17	−8	27.20	122.42
0	−19	4	111.99	49.26
2	−22	−6	−21.00	121.10
4	−26	−3	175.64	91.52
4	−27	−2	0.00	93.10
3	−30	−3	2.25	135.10
−1	−26	4	41.47	71.97
3	−31	−2	−12.15	124.93
−3	−31	4	30.77	82.93
0	−36	0	0.79	107.37
−28	38	0	36.32	107.76
−30	40	−1	0.00	109.74
−11	20	−1	321.70	83.59
−22	28	4	34.20	119.38
−21	25	5	133.38	118.85
−8	9	4	722.64	105.65
−12	13	5	174.32	85.71
−19	20	6	26.15	106.18
−14	13	6	−60.62	90.99
−5	3	4	489.68	72.63
−20	18	7	31.30	124.53
−13	7	7	98.25	81.09
−1	−6	−7	1.58	76.86
−12	5	7	42.39	67.35

4	−19	−4	−43.05	87.16
4	−22	−4	13.87	90.46
−3	−15	−9	56.52	114.36
−1	−20	−8	170.09	120.04
−3	−18	−9	−50.84	114.23
−7	−14	7	11.36	39.09
2	−29	−5	−33.02	117.53
1	−33	1	11.62	99.18
−7	−28	6	10.70	67.35
−7	−33	5	8.58	89.14
−15	25	−1	27.47	76.60
−12	19	2	298.19	96.67
−6	12	0	186.34	50.05
−25	31	4	55.73	132.59
−11	16	3	221.60	97.99
−24	28	5	21.26	127.57
−17	20	5	−46.22	92.57
−5	7	−6	57.84	48.73
−4	5	−6	124.40	55.47
−24	19	8	−78.71	105.65
−1	−14	−8	−10.04	124.67
−15	−6	9	−15.05	78.05
3	−25	−5	0.00	124.27
2	−27	2	−62.46	75.67
3	−29	−4	137.61	136.15
3	−30	0	82.01	112.12
−5	−23	6	15.85	49.79
−16	26	−3	173.92	70.12
−16	25	1	31.17	79.10
−12	20	1	221.86	90.86
−16	24	2	27.86	82.27
−4	9	−1	785.90	75.67
−6	11	1	148.44	55.20
−7	7	4	2678.86	236.39
1	−2	−3	193.47	44.64
−6	5	4	644.06	83.86
3	−8	−1	100.10	47.81
−3	−4	−8	277.99	90.46
−19	12	8	51.50	113.04
4	−12	−2	233.35	64.31
−4	−7	−9	78.71	123.61
−22	11	9	−22.71	110.01
4	−16	−4	73.29	82.41
−13	0	8	−2.11	105.38
1	−16	−7	73.29	103.67
0	−16	4	117.93	36.98
1	−19	−7	101.42	130.74
−11	−7	8	47.81	80.29
4	−26	0	53.88	79.10
−21	−1	10	−62.73	105.12
2	−25	−6	68.67	125.72
4	−28	−1	67.75	98.91
−20	−6	10	53.75	97.59
1	−35	0	−56.13	101.03

1	−36	−2	0.00	107.89
−19	29	0	−22.71	78.71
−25	35	0	−188.98	132.59
−19	26	3	113.18	89.27
−3	3	−6	710.62	85.84
−11	11	5	874.38	113.04
−1	−4	3	251.44	34.34
2	−13	−6	65.77	88.35
−3	−8	5	135.10	42.79
−21	9	9	57.05	96.14
−6	−8	−10	−23.77	102.74
−3	−12	−9	74.75	116.74
4	−25	−4	53.09	93.63
3	−28	1	26.02	80.56
1	−26	3	21.66	72.11
3	−32	−1	7.13	118.59
1	−31	2	−38.17	90.33
−15	−15	9	0.13	64.18
2	−34	−3	58.64	112.38
−6	−32	5	65.37	82.93
−22	32	0	63.65	107.89
−12	21	−3	−12.02	56.39
−14	23	−4	20.60	58.77
−28	37	1	−47.81	116.21
−14	18	4	130.21	81.61
−22	23	6	0.00	142.49
−19	16	7	−22.05	122.82
1	−8	−6	−18.75	70.92
−8	1	6	−30.51	64.31
−18	10	8	−19.28	115.42
4	−14	0	1151.70	113.18
3	−13	−5	27.47	79.24
−20	7	9	22.71	100.76
−19	5	9	−25.49	98.78
5	−20	−2	−15.32	88.48
−18	3	9	91.52	97.86
5	−23	−2	72.63	80.56
−3	−26	5	63.26	64.97
−27	37	−1	13.21	112.65
−28	36	2	−14.00	107.10
−22	29	3	0.00	101.82
−18	23	4	143.95	89.01
−6	9	−6	21.00	41.86
−3	5	−5	524.55	70.65
−18	18	6	2.91	94.42
−12	9	6	53.75	76.20
−6	1	5	448.61	80.82
3	−9	0	403.71	56.13
2	−8	−5	212.75	85.31
−23	17	8	−63.52	103.14
−17	8	8	42.00	117.80
−1	−11	−8	69.73	111.06
−14	2	8	69.46	112.25
5	−21	−1	1.98	83.59

3	−20	2	138.14	63.92
−5	−13	−10	−21.13	110.27
−22	1	10	−34.86	106.04
0	−22	4	24.96	51.37
1	−25	−7	106.44	108.42
4	−29	−3	−23.11	119.78
3	−28	−5	23.77	120.97
2	−32	1	103.27	104.72
0	−30	3	59.43	82.67
−2	−30	4	19.94	76.46
2	−35	−2	−43.71	101.82
−3	−38	2	75.80	110.27
−4	−39	2	−157.02	121.50
−30	39	0	33.94	111.06
−9	17	−2	−3.83	60.09
−15	21	3	160.06	80.95
−10	14	3	699.26	102.48
−20	23	5	5.02	96.67
−16	18	5	1.85	82.41
−25	26	6	−50.05	121.50
−1	1	1	1033.25	88.08
−5	5	−7	88.08	63.13
−2	1	−6	172.47	66.69
−10	9	5	43.84	80.95
−11	7	6	577.77	94.29
−6	0	−9	0.00	116.61
−9	3	6	27.20	68.80
−16	6	8	−8.72	119.12
−15	4	8	14.00	114.76
1	−13	−7	50.32	84.39
5	−22	−3	173.53	89.27
4	−21	1	214.86	74.61
−25	7	10	112.12	115.16
5	−24	−1	99.18	82.80
−23	3	10	−1.85	106.71
2	−28	−6	17.04	106.04
2	−34	0	58.77	101.29
−9	−25	7	−10.43	48.99
1	−37	−1	−49.79	106.31
−2	−37	2	−29.71	106.18
−5	−38	3	−6.07	97.86
−18	28	−1	13.47	76.86
−26	36	−2	−21.26	108.82
−19	28	1	10.30	88.48
−25	34	1	68.14	137.34
−25	33	2	−14.66	135.76
−5	10	0	−14.39	42.39
−5	9	1	724.22	81.35
−4	7	−5	827.89	85.44
−2	3	−5	101.69	58.77
−6	7	−7	26.54	55.47
−4	3	−7	148.44	66.82
−9	7	5	82.14	82.54
−10	5	6	107.89	69.33

0	−8	−7	20.73	83.33
−2	−6	−8	134.31	86.63
5	−17	−2	−1.06	79.50
5	−18	−1	12.15	83.59
5	−19	−3	93.10	83.59
4	−21	−5	117.80	89.27
3	−21	−6	−12.15	101.29
0	−19	−8	109.87	117.01
−2	−17	−9	−51.77	106.31
−24	5	10	−57.97	109.48
3	−23	2	46.75	65.63
5	−26	−2	93.63	91.65
4	−28	−4	0.00	121.50
−15	−9	9	−29.71	73.82
−5	−31	5	14.39	77.39
−2	−39	1	103.40	109.87
−4	−37	3	32.62	96.80
−6	−39	3	−42.13	102.48
−17	27	−2	48.99	73.43
−19	29	−3	43.32	79.24
−24	34	−1	5.28	125.85
−22	31	1	27.73	95.08
−11	19	0	322.76	81.22
−22	30	2	−33.68	102.61
−11	17	2	220.28	80.82
−21	26	4	7.92	102.22
−23	26	5	80.82	127.04
−8	5	5	235.73	81.22
3	−7	−2	2083.00	181.98
−18	14	7	36.05	106.71
−25	14	9	−98.78	119.38
−3	−9	−9	−29.58	115.55
3	−18	−6	−9.38	85.18
−5	−10	−10	21.00	103.67
0	−16	−8	48.60	122.82
5	−25	−3	−12.68	84.12
4	−24	1	14.26	71.71
−16	−4	9	55.47	83.99
0	−22	−8	125.59	112.91
−2	−22	5	−9.90	53.09
4	−29	0	68.01	90.86
−21	−4	10	−10.56	96.80
3	−32	−4	25.75	106.97
3	−34	−2	−55.47	102.35
−1	−38	1	0.00	111.86
−3	−36	3	31.96	89.93
−21	31	−1	49.13	88.61
−22	32	−3	−8.32	113.04
−15	24	0	26.94	73.43
−24	29	4	178.15	139.59
−3	7	−1	2856.74	236.39
−13	16	4	94.03	82.01
−26	29	5	−1.72	111.06
−3	1	−7	−88.61	66.95

−5	−2	−9	−15.72	113.84
−22	15	8	−1.06	105.25
4	−13	−4	187.79	91.78
−9	−4	7	22.71	60.35
4	−18	−5	223.84	88.35
3	−17	2	577.90	80.43
−2	−14	−9	4.49	106.84
4	−24	−5	−64.45	103.80
3	−24	−6	37.64	130.48
4	−31	−1	38.83	117.14
−15	−12	9	−22.45	70.12
2	−36	−1	0.00	102.88
−1	−36	2	46.88	99.44
−23	33	−2	26.41	114.10
−15	22	2	25.09	76.33
−1	1	−5	37.24	58.11
−21	21	6	78.05	133.51
−17	16	6	−28.92	83.20
−1	−1	−6	266.37	69.99
−22	19	7	82.93	117.67
4	−11	−3	1363.66	136.82
3	−33	0	10.56	103.40
−6	−27	6	0.00	60.48
−2	−35	3	0.00	86.37
−20	30	−2	−27.20	80.69
−27	36	0	121.23	108.69
−10	18	−1	27.60	66.82
−11	18	1	377.30	87.03
−18	24	3	39.35	86.63
−17	21	4	194.39	86.90
−4	7	1	2613.49	223.05
−7	9	−7	60.35	51.90
−17	12	7	27.86	88.35
2	−10	−6	7.26	73.29
5	−16	−3	0.66	91.91
−24	12	9	15.45	108.03
5	−21	−4	50.05	86.24
2	−18	−7	0.00	113.57
−12	−5	8	71.97	90.86
5	−24	−4	26.02	86.76
−11	−10	8	8.32	62.46
5	−28	−3	60.62	101.29
4	−27	−5	−1.32	125.46
4	−32	−3	0.13	107.50
3	−31	−5	146.46	110.67
2	−30	2	−49.65	84.12
−29	38	−1	−70.65	109.74
−9	12	3	581.07	92.18
−15	16	5	40.54	83.46
−24	24	6	73.56	120.44
2	−4	−3	746.28	80.29
−4	−4	−9	106.97	120.57
−21	13	8	50.71	102.74
0	−13	−8	34.73	123.87

2	−24	−7	32.09	107.37
3	−26	2	176.96	70.12
5	−29	−2	−37.24	90.07
3	−27	−6	42.26	118.46
−22	−2	10	−18.88	99.44
4	−31	−4	45.96	106.44
3	−31	1	113.18	115.69
0	−39	0	54.54	108.16
−5	−36	4	0.66	88.35
−6	−37	4	−61.67	94.42
−13	22	−2	106.97	64.31
−14	23	−1	−14.13	66.69
−14	19	3	222.92	85.44
−3	5	1	1289.05	121.36
−19	21	5	−28.53	85.18
−2	3	1	534.05	56.92
−2	−1	−7	31.83	71.05
3	−8	−4	153.19	72.24
−25	18	8	101.69	100.23
1	−10	−7	64.45	81.09
−1	−8	−8	83.20	90.20
−2	−11	−9	−83.73	109.48
−23	10	9	−51.50	104.46
5	−19	0	116.35	77.26
−4	−12	−10	105.52	103.14
2	−19	3	135.49	52.69
−17	−2	9	−52.03	87.82
5	−25	0	46.88	75.54
2	−22	3	69.99	59.03
4	−27	1	70.12	72.24
4	−33	−2	1.32	108.29
3	−35	−1	0.00	105.91
−4	−30	5	−15.85	73.16
1	−36	1	59.82	104.20
−18	27	0	−11.89	76.73
−24	33	0	69.60	129.29
−27	35	1	−10.70	119.12
−21	27	3	−23.64	98.91
−24	30	3	−3.70	130.08
−4	8	0	378.22	54.94
0	−1	−5	87.42	66.29
−16	14	6	96.27	81.48
−21	17	7	34.34	120.70
−16	10	7	28.26	84.25
2	−15	−7	137.48	88.61
−1	−19	−9	−75.14	105.12
5	−27	−4	17.83	106.44
0	−25	4	−43.32	62.86
1	−29	3	−21.79	74.61
−1	−34	3	133.25	85.97
1	−38	0	8.58	107.76
−4	−35	4	0.00	83.07
−27	34	2	32.22	118.33
−10	15	2	388.00	84.25

−12	14	4	98.39	88.22
0	−3	−6	721.05	97.20
5	−14	−2	−7.26	70.26
5	−15	−1	212.35	66.69
−6	−6	6	124.27	53.22
−10	−2	7	211.56	68.28
−22	8	9	−16.51	101.03
1	−18	−8	−25.75	110.54
1	−21	−8	88.48	108.55
−23	0	10	−34.86	99.57
−17	26	−4	22.58	64.05
−21	30	0	141.44	88.61
−15	23	−5	8.32	50.18
−11	18	−5	299.38	45.03
−18	25	2	22.98	83.73
−5	9	−5	142.49	39.88
−20	24	4	56.79	83.33
−26	30	4	−43.45	125.33
−2	5	−1	1258.01	107.76
−22	24	5	23.90	106.97
−25	27	5	0.00	130.61
−6	5	−8	46.09	64.58
−20	19	6	78.84	97.99
−5	3	−8	62.07	68.01
−1	−3	−7	81.48	71.45
−20	11	8	−0.13	107.23
−1	−16	−9	−45.69	108.82
−8	−9	7	−27.20	58.50
−18	0	9	53.35	90.46
−26	6	10	69.86	114.63
−25	4	10	0.00	108.29
−24	2	10	22.58	106.18
5	−30	−1	−71.58	102.08
4	−30	−5	72.37	109.61
4	−32	0	−32.75	113.31
1	−34	2	46.62	84.52
−26	35	−1	−10.17	119.52
−18	26	1	13.07	84.65
−24	32	1	12.94	124.93
−24	31	2	14.79	131.93
−23	27	4	18.36	112.78
−8	10	3	131.40	62.60
−14	14	5	477.27	95.08
−4	1	−8	81.48	71.84
4	−10	−1	1472.74	134.44
−15	8	7	140.25	79.76
−3	−6	−9	4.09	117.53
−21	6	9	0.00	94.95
6	−22	−2	53.75	77.92
4	−20	−6	14.13	98.12
−13	−3	8	−51.37	98.39
−20	4	9	0.92	90.59
5	−23	−5	29.58	94.03
6	−25	−2	−12.02	84.78

4	−23	−6	−71.58	118.33
5	−28	0	148.96	79.63
−16	−7	9	−3.57	75.41
5	−31	−3	−44.11	121.23
4	−34	−1	43.71	101.42
2	−35	1	−24.96	91.91
2	−37	0	40.67	101.16
−3	−34	4	28.66	80.29
−15	24	−3	48.47	63.26
−21	29	1	0.00	86.63
−29	37	0	0.00	96.93
−31	39	−1	18.75	97.59
−9	16	−4	86.37	41.60
−7	11	−6	146.98	35.79
−16	19	4	231.11	80.43
−7	7	−8	201.26	68.01
−24	20	7	46.88	108.95
−24	16	8	−4.49	108.42
5	−15	−4	24.17	89.54
0	−10	−8	230.05	104.86
−19	9	8	−4.36	104.06
4	−15	1	168.51	55.60
−11	0	7	262.01	72.11
1	−15	−8	−97.59	125.85
6	−21	−3	115.82	82.01
5	−20	−5	157.42	90.46
6	−24	−3	148.17	87.69
4	−26	−6	102.08	122.82
2	−25	3	8.06	61.14
−11	−13	8	42.52	55.73
5	−30	−4	−75.67	124.53
5	−32	−2	9.11	118.19
−22	−5	10	0.00	94.82
3	−37	−2	37.11	110.93
−20	29	−4	34.73	77.26
−6	12	−3	389.58	46.62
−10	16	1	56.92	66.29
−14	20	2	106.44	89.93
−18	19	5	62.07	90.33
−23	22	6	146.46	123.34
−26	25	6	41.07	108.69
1	−3	−5	54.01	69.33
−3	−1	−8	116.21	72.90
−20	15	7	−127.17	118.72
−2	−6	4	321.44	45.56
−14	6	7	83.33	78.71
3	−12	−6	114.50	78.84
6	−19	−2	180.40	91.65
−4	−9	−10	0.00	94.16
−26	13	9	−40.15	101.95
−1	−13	−9	−7.40	106.97
6	−23	−1	−35.13	75.67
3	−20	−7	74.88	119.12
−3	−14	−10	36.45	104.99

3	−23	−7	−10.17	99.31
6	−27	−3	−21.79	86.63
5	−26	−5	−6.47	117.67
−11	−16	8	0.00	46.35
4	−35	−3	55.73	103.93
0	−33	3	29.45	81.09
−17	26	−1	46.22	75.01
−23	32	−1	28.79	101.95
−14	22	0	191.62	81.09
−17	22	3	124.93	82.67
−13	17	3	65.11	94.82
−3	6	0	37.64	43.71
−11	12	4	108.03	89.54
1	−5	−6	149.89	69.46
4	−9	−2	1327.61	126.12
5	−13	−3	564.30	95.22
−13	4	7	−1.19	72.37
2	−12	−7	182.64	83.07
−12	2	7	134.44	73.16
−18	7	8	0.00	108.16
−14	−1	8	−53.88	102.48
6	−28	−2	−80.16	86.37
3	−26	−7	158.87	104.20
4	−30	1	71.05	97.86
3	−29	2	28.13	76.73
−20	29	−1	38.96	78.97
−25	34	−2	−10.56	114.23
−11	19	−3	5.68	55.33
−8	15	−2	123.08	50.58
−29	36	1	32.09	109.61
−29	35	2	27.07	104.46
−8	11	−7	127.17	45.56
−7	8	3	198.22	57.45
−19	17	6	221.47	89.93
3	−6	−3	621.88	78.05
0	−5	−7	146.06	76.07
4	−10	−4	156.10	82.93
4	−12	−5	110.54	70.52
−2	−8	−9	−4.09	114.36
5	−16	0	519.79	77.12
6	−20	−1	173.79	87.42
3	−17	−7	134.83	107.76
6	−23	−4	28.53	87.03
4	−29	−6	−5.28	99.05
4	−36	−2	98.12	100.37
3	−36	0	0.00	93.10
−16	25	−2	4.89	67.62
−24	33	−3	26.68	115.42
−14	21	1	402.65	94.16
−26	31	3	−90.46	132.99
−9	13	2	29.58	64.05
−28	31	4	−119.12	106.18
−13	12	5	203.11	79.37
−2	−2	3	627.29	62.73

−2	−3	−8	160.45	76.73
−23	14	8	35.52	94.42
6	−18	−3	−36.71	89.67
5	−17	−5	135.76	82.14
−17	5	8	−66.56	110.54
−16	3	8	52.96	105.38
−15	1	8	3.17	101.16
−25	11	9	105.65	105.12
0	−18	−9	141.17	103.67
6	−26	−4	60.88	94.42
5	−29	−5	155.96	114.89
−23	−3	10	46.49	96.14
3	−34	1	99.05	92.44
2	−33	2	−11.36	83.86
−3	−29	5	−6.34	64.71
−2	−33	4	47.01	76.07
−3	−41	2	−0.13	97.86
−26	34	0	1.19	122.29
−9	16	−1	402.26	68.80
−20	25	3	13.60	80.43
−23	28	3	−6.74	99.97
−19	22	4	58.37	87.82
−21	22	5	−36.98	90.33
−27	28	5	72.77	107.89
−23	18	7	−20.60	113.18
−19	13	7	0.00	103.67
4	−11	0	852.32	85.97
3	−10	1	1101.79	99.31
1	−11	3	427.22	45.16
6	−20	−4	10.30	81.61
2	−16	3	60.35	51.50
2	−20	−8	58.24	110.27
2	−23	−8	31.56	97.73
−17	−5	9	−36.58	79.76
6	−30	−3	−12.41	127.17
5	−31	0	0.13	106.18
−16	−10	9	68.41	72.77
5	−33	−4	20.34	98.78
5	−33	−1	−13.07	111.20
5	−34	−3	−34.07	88.22
3	−38	−1	82.93	99.44
−2	−40	2	−28.92	106.18
−18	27	−3	11.89	65.50
−21	30	−3	40.28	89.14
−22	31	−2	−55.86	92.84
−31	38	0	65.50	97.33
−24	25	5	76.73	121.50
2	−5	−5	376.37	86.24
1	−12	−8	101.42	107.89
−7	−4	6	290.14	61.54
−3	−11	−10	54.28	99.44
5	−23	1	40.54	64.31
6	−29	−1	−13.47	84.92
−24	−1	10	−26.54	95.48

0	−40	1	6.60	102.88
−1	−39	2	74.61	98.65
−13	21	−4	17.96	51.11
−28	36	−1	−62.33	103.67
−26	33	1	108.69	131.14
−17	23	2	33.02	81.09
−26	32	2	−38.03	126.78
−22	25	4	36.32	95.61
−25	28	4	3.96	118.46
−15	17	4	14.53	71.71
−6	6	3	277.20	59.03
−10	10	4	2246.09	207.86
−17	17	5	107.10	80.56
−22	20	6	0.13	117.80
2	−7	−6	47.94	70.12
−26	21	7	−44.64	99.44
1	−7	−7	21.00	77.26
−1	−5	−8	150.02	81.88
−26	17	8	131.66	96.14
−4	−6	5	127.04	50.45
2	−17	−8	−31.96	116.87
0	−15	−9	−34.07	101.29
−24	9	9	67.22	105.91
5	−26	1	63.39	71.45
−27	5	10	65.11	108.82
6	−29	−4	−61.67	120.31
−26	3	10	−63.13	106.31
−25	1	10	3.43	100.10
2	−28	3	18.75	68.54
0	−28	4	0.00	66.03
−16	−13	9	0.00	67.22
5	−35	−2	−42.00	104.72
4	−35	0	45.16	94.56
−4	−40	3	99.57	95.22
−13	21	−1	127.17	77.65
−17	25	0	94.16	74.35
−23	31	0	−48.33	90.07
−12	10	5	162.04	75.67
−25	23	6	−62.86	111.06
−13	8	6	117.40	74.09
4	−14	−6	404.90	96.80
−22	12	8	52.43	99.31
3	−14	−7	105.52	80.43
−1	−10	−9	125.19	112.25
−5	−11	6	103.67	37.77
5	−22	−6	−12.02	99.18
4	−22	2	18.49	59.43
5	−25	−6	0.00	118.06
−5	−26	6	15.98	50.05
4	−37	−1	−20.34	97.20
1	−39	1	6.74	102.48
0	−38	2	51.37	93.63
−3	−39	3	0.00	87.03
−12	20	−2	6.34	66.56

−20	28	0	4.62	74.48
−20	26	2	0.00	79.90
−12	15	3	123.61	83.73
−1	3	−4	106.18	41.60
−2	4	0	500.51	47.01
0	1	−4	107.63	46.35
−18	15	6	37.37	84.92
6	−16	−2	222.52	80.43
6	−22	−5	−24.17	80.43
−23	7	9	35.39	101.03
6	−25	−5	59.30	106.18
4	−25	−7	−13.87	116.87
4	−25	2	52.43	68.41
−18	−3	9	0.00	82.41
5	−28	−6	51.50	104.20
−2	−38	3	−59.16	84.52
−23	30	1	26.02	87.82
−13	18	2	181.98	89.67
−16	20	3	230.97	83.73
−28	32	3	70.39	110.80
−5	4	3	254.09	51.90
−3	0	3	1171.78	105.52
−12	6	6	122.29	72.63
−18	11	7	172.21	95.48
6	−17	−4	95.35	77.78
6	−17	−1	464.85	84.52
5	−19	−6	0.00	82.54
6	−21	0	62.73	78.71
−9	−7	7	55.20	59.96
6	−27	0	96.14	75.67
6	−28	−5	−68.28	119.78
4	−33	1	−55.33	100.23
−1	−32	4	79.76	72.24
−7	−31	6	−25.36	66.82
−20	27	1	−39.88	79.37
−9	14	1	−26.28	56.92
1	−1	−4	141.97	51.24
−4	2	3	678.00	71.97
4	−8	−3	394.86	67.62
−4	−1	−9	17.70	106.18
−22	16	7	108.95	121.63
5	−12	−4	118.99	81.61
0	−7	−8	216.84	85.71
5	−14	−5	44.77	82.80
−8	−2	6	15.58	57.45
−21	10	8	56.39	98.12
4	−19	−7	−18.22	111.72
−2	−13	−10	−87.69	98.78
−27	12	9	−0.13	94.95
7	−23	−3	104.06	92.31
7	−24	−2	72.11	84.52
7	−26	−3	157.42	82.93
1	−20	−9	7.00	87.82
7	−27	−2	11.75	79.63

−19	−1	9	−5.15	91.78
5	−29	1	42.13	72.63
6	−32	−4	47.94	101.03
6	−32	−1	−4.49	113.44
3	−32	2	13.60	78.58
1	−37	2	−71.58	87.95
−5	−34	5	0.00	74.35
−25	33	−1	21.79	113.31
−27	35	−2	−41.47	103.93
−28	35	0	−16.64	104.72
−9	15	0	284.72	59.82
−8	11	2	587.67	76.86
−20	20	5	3.96	81.09
−9	8	4	760.01	99.31
−11	8	5	211.69	82.93
3	−7	−5	815.34	109.08
−3	−3	−9	58.24	106.04
5	−12	−1	640.10	78.44
2	−9	−7	11.49	70.26
−11	4	6	330.28	76.86
6	−15	−3	218.56	87.69
−25	15	8	6.21	92.84
2	−14	−8	0.00	112.78
6	−19	−5	56.52	79.90
3	−22	−8	−47.81	94.16
−20	1	9	1.85	90.59
6	−34	−2	77.78	101.29
−1	−37	3	66.95	81.88
−30	37	−1	−59.03	92.44
−28	33	2	66.95	118.99
−25	29	3	48.47	124.53
−18	20	4	154.91	87.16
−27	29	4	164.94	121.36
−23	23	5	121.50	104.72
−26	26	5	70.65	120.97
−16	15	5	190.30	82.93
−21	18	6	26.28	97.99
5	−11	−2	378.62	68.41
3	−9	−6	−11.89	67.75
−25	19	7	70.26	100.76
−17	9	7	−19.68	81.75
−9	0	6	68.94	62.73
−3	−8	−10	0.00	90.99
0	−12	−9	−34.86	102.61
7	−21	−2	275.22	86.24
4	−19	2	142.10	63.26
1	−17	−9	−11.36	97.99
7	−25	−4	12.28	82.27
−13	−6	8	−44.50	76.46
7	−29	−3	10.83	97.46
4	−28	2	−2.91	69.07
−17	−8	9	−8.32	72.90
−24	−4	10	−56.13	99.18
5	−34	0	−40.01	96.93

5	−36	−1	24.43	92.97
−13	20	0	130.48	77.65
−13	19	1	94.29	79.76
−28	34	1	0.79	108.55
−19	23	3	−27.07	82.54
−22	26	3	18.75	82.80
−2	5	−4	463.67	53.62
−14	15	4	12.15	76.99
−10	2	6	−53.88	63.39
−20	8	8	92.05	99.18
7	−20	−3	99.44	78.97
7	−22	−4	189.51	84.12
3	−19	−8	131.27	115.03
−26	10	9	23.64	101.42
7	−25	−1	112.65	82.27
7	−28	−4	−101.16	108.29
6	−30	0	−60.22	88.22
6	−31	−5	68.54	98.39
3	−39	0	29.71	100.23
−16	24	−1	19.28	66.69
−22	30	−1	9.77	78.31
−21	23	4	83.07	80.03
−24	26	4	30.37	104.99
2	−3	−4	120.97	54.54
−24	21	6	3.70	115.29
−27	24	6	−50.98	101.82
−2	−5	−9	25.62	110.01
4	−16	−7	70.39	83.59
−25	−2	10	−107.50	98.12
3	−37	1	19.02	92.57
0	−36	3	94.42	80.03
−4	−33	5	−1.98	69.73
−1	2	0	−8.72	5.55
1	−1	−1	1119.48	94.82
−8	6	4	434.08	76.20
−10	6	5	117.40	75.14
−21	14	7	131.40	118.06
1	−9	−8	97.20	86.50
−24	13	8	32.49	90.33
7	−22	−1	−43.98	71.58
−1	−15	−10	−59.43	99.18
6	−24	−6	36.98	117.93
1	−21	4	11.09	45.16
6	−27	−6	104.46	108.03
7	−32	−3	12.81	115.16
−27	2	10	51.90	98.39
−26	0	10	18.75	99.71
2	−31	3	−27.07	73.95
5	−38	−2	21.92	103.14
−4	−38	4	109.08	75.54
−19	27	−1	41.86	74.48
−24	32	−2	59.16	106.18
−25	32	0	72.24	110.54
−16	21	2	42.13	76.07

−25	30	2	0.00	108.55
−11	13	3	17.83	77.78
−5	−4	5	206.81	57.84
−16	7	7	181.19	81.61
−2	−10	−10	169.96	96.14
−19	6	8	−21.39	96.80
−10	−5	7	74.75	60.75
−25	8	9	−68.54	101.95
5	−24	−7	−7.66	107.37
−8	−12	7	16.64	63.92
5	−27	−7	−26.28	98.52
1	−24	4	23.24	52.69
−12	−11	8	33.15	60.22
7	−31	−4	67.88	118.99
6	−36	−3	−46.88	92.05
−2	−28	5	0.00	57.71
4	−38	0	−36.85	99.97
−14	22	−3	59.82	55.07
−16	24	−4	8.85	56.13
−25	31	1	205.09	107.76
−30	36	0	−80.56	96.14
−32	38	−1	76.73	92.71
−15	18	3	6.07	78.05
4	−9	−5	159.79	88.61
4	−11	−6	170.75	77.65
3	−11	−7	188.72	82.01
−1	−7	−9	90.46	111.20
−27	16	8	−59.43	91.91
6	−18	0	232.03	72.63
5	−17	1	384.56	72.11
3	−16	−8	−61.41	115.03
1	−14	−9	−18.36	100.76
6	−21	−6	−41.47	85.44
7	−24	−5	4.49	85.44
−14	−4	8	52.30	83.86
7	−27	−5	−31.03	118.46
3	−24	3	128.23	59.03
5	−32	1	28.92	104.06
6	−35	−1	102.08	93.90
−6	−30	6	11.49	59.16
−3	−37	4	−27.47	78.18
−15	23	−2	70.92	62.20
−29	36	−2	−57.18	90.33
−8	14	−1	218.43	52.56
−19	24	2	25.22	80.95
−22	27	2	8.32	78.31
−12	16	2	499.06	98.65
−30	34	2	42.52	101.95
−7	9	2	807.69	85.84
−29	30	4	48.07	104.86
−19	18	5	51.37	78.18
−28	27	5	0.00	99.44
5	−10	−3	660.97	83.73
−24	17	7	−11.89	100.89

−27	20	7	148.83	93.10
6	−14	−4	24.17	91.52
6	−16	−5	67.35	76.60
−15	5	7	123.61	73.95
7	−18	−2	233.22	87.82
7	−19	−4	70.39	75.80
−18	4	8	5.15	91.25
2	−19	−9	−0.13	96.14
7	−31	−1	145.14	99.18
−18	−6	9	−11.75	74.35
7	−33	−2	−115.82	110.67
4	−31	2	26.15	75.41
−17	−11	9	38.56	69.73
0	−31	4	58.11	68.67
4	−36	1	93.24	84.78
−8	−24	7	−19.15	22.98
−19	27	−4	31.43	65.90
−23	31	−3	−80.43	103.80
−16	23	0	233.48	73.82
−22	29	0	10.43	75.14
−27	34	−1	60.35	109.21
−30	35	1	−57.05	99.97
−27	30	3	34.07	118.85
−15	13	5	31.03	73.82
−7	4	4	1422.43	136.82
−20	16	6	41.20	83.99
5	−13	0	573.67	70.12
2	−11	−8	29.45	93.76
−23	11	8	24.83	91.91
−17	2	8	3.96	92.57
−15	−2	8	44.37	86.63
4	−24	−8	70.26	86.10
7	−30	−5	121.50	111.06
6	−33	0	15.19	99.84
6	−37	−2	58.11	98.25
1	−35	3	0.66	73.43
−21	29	−2	−1.85	74.48
−7	13	−2	86.24	38.69
−12	18	−6	3.04	24.17
−22	28	1	6.21	75.94
−8	12	1	182.24	54.81
−17	18	4	54.28	77.39
−13	13	4	37.11	85.71
−22	21	5	−3.83	86.63
−25	24	5	−24.96	116.48
−8	2	5	292.38	76.99
−6	−2	5	227.94	65.50
−20	12	7	43.84	106.71
7	−17	−3	309.29	87.82
−14	3	7	214.20	73.69
−1	−12	−10	2.38	89.27
7	−21	−5	300.97	86.37
−11	−3	7	270.86	68.28
−16	0	8	−0.13	89.41

4	−21	−8	−41.60	100.37
3	−21	3	146.19	53.75
3	−27	3	−10.56	60.62
7	−34	−4	−26.68	95.61
5	−37	0	0.00	85.31
3	−35	2	116.35	80.69
−2	−36	4	−21.26	73.16
−1	−42	2	−24.96	100.10
−18	26	−2	0.00	67.88
−12	19	−1	284.33	75.80
−19	26	0	109.87	79.24
−19	25	1	0.00	80.16
−26	27	4	15.32	120.70
−6	2	4	99.84	54.81
−23	19	6	37.77	120.44
−26	22	6	0.00	112.91
6	−13	−2	106.57	60.35
−7	0	5	1424.67	139.72
0	−9	−9	−19.55	106.18
7	−19	−1	405.82	93.10
−13	1	7	109.35	69.60
5	−18	−7	−7.53	94.29
8	−25	−3	115.55	80.29
8	−26	−2	91.91	77.26
6	−25	1	−33.02	66.56
−23	4	9	16.38	92.84
8	−28	−3	13.60	82.67
−19	−4	9	67.09	82.67
1	−27	4	34.34	60.09
7	−35	−3	17.43	90.33
−17	−14	9	23.90	65.90
5	−39	−1	8.85	91.65
−3	−32	5	22.32	63.65
−17	25	−3	55.07	62.60
−20	28	−3	8.98	68.94
−32	37	0	−36.32	90.86
−18	21	3	380.07	88.74
−24	27	3	64.58	87.82
2	−3	−1	548.45	56.52
−5	0	4	590.05	71.58
4	−13	−7	54.41	77.52
−26	14	8	55.33	95.08
−12	−1	7	164.15	65.24
2	−16	−9	29.32	95.22
8	−27	−4	3.04	97.99
7	−26	0	138.14	75.27
−22	2	9	37.37	88.88
8	−29	−2	26.81	80.82
6	−28	1	23.77	66.03
−4	−22	6	0.00	33.02
2	−41	1	0.53	92.18
0	−41	2	52.96	95.48
−10	17	−3	242.46	57.58
−27	33	0	−146.72	113.44

−8	13	0	509.36	68.01
−27	31	2	−9.11	116.74
−21	24	3	73.29	84.39
−10	11	3	491.40	81.88
−20	21	4	69.86	75.67
−23	24	4	−47.01	89.93
−1	0	−7	−1.58	62.46
4	−7	−4	1161.34	120.57
−15	9	6	182.11	77.12
6	−14	−1	175.64	59.16
5	−13	−6	−57.71	76.73
4	−12	1	171.55	42.92
−22	9	8	38.17	87.82
8	−22	−3	122.82	77.65
4	−18	−8	43.18	108.29
8	−23	−2	90.73	72.37
8	−24	−4	−25.36	80.29
7	−26	−6	−26.15	109.48
−21	0	9	22.05	87.42
8	−30	−4	−36.18	111.06
7	−29	0	37.90	75.41
−20	−2	9	−13.73	85.44
8	−31	−3	0.00	111.99
7	−34	−1	104.20	98.52
−28	1	10	0.00	95.22
−27	−1	10	−4.62	99.31
−24	31	−1	17.70	88.48
−31	37	−2	0.00	84.12
−12	17	1	431.18	87.56
−27	32	1	89.93	117.14
−29	31	3	31.30	108.69
−2	2	−7	213.15	63.65
0	−2	−7	94.42	66.95
−23	15	7	32.35	114.10
−19	10	7	−15.32	94.56
3	−13	−8	14.53	99.18
6	−22	1	89.14	63.26
6	−26	−7	−6.07	96.27
7	−29	−6	152.79	100.10
7	−36	−2	37.64	96.40
5	−35	1	−10.17	83.73
1	−40	2	52.56	88.22
−26	33	−2	28.39	107.37
−18	16	5	96.93	83.73
1	−4	−7	89.14	69.73
−14	11	5	216.98	74.88
6	−12	−3	401.33	78.58
−26	18	7	−11.49	100.10
1	−11	−9	20.47	103.01
7	−18	−5	326.32	84.12
0	−14	−10	97.99	92.84
7	−23	−6	43.98	100.23
8	−32	−2	−49.65	105.52
6	−38	−1	6.07	87.82

−1	−35	4	16.90	69.73
3	−40	1	33.81	93.76
−2	−41	3	18.62	82.67
−11	18	−2	335.96	70.12
−14	21	−5	217.37	50.18
−29	35	−1	−54.28	94.29
−15	19	2	102.22	82.54
−14	16	3	78.58	86.50
−6	7	2	73.56	45.30
0	0	−6	1007.36	106.18
−2	0	−8	56.39	66.16
−27	25	5	21.92	110.80
−1	−2	−8	25.49	69.33
7	−16	−4	196.90	84.78
8	−21	−4	96.93	79.37
8	−26	−5	−63.13	103.93
5	−24	2	23.64	57.97
8	−29	−5	−36.58	107.23
5	−27	2	43.32	60.48
−13	−9	8	−17.43	61.54
8	−33	−4	−82.67	94.69
6	−31	1	1.58	92.84
−12	−14	8	−7.79	51.77
3	−30	3	44.24	67.75
−18	−9	9	−5.68	66.16
6	−36	0	−4.49	85.18
2	−34	3	8.32	72.77
−12	−20	8	8.58	34.86
−1	−40	3	−46.22	78.05
−9	13	−7	26.54	31.17
−28	28	4	0.00	115.29
1	−2	−6	263.59	66.16
−3	2	−8	26.15	61.80
−12	11	4	158.21	79.63
0	−4	−8	324.21	78.31
−28	23	6	−27.60	95.74
−14	7	6	380.20	74.75
−25	12	8	0.13	87.03
6	−20	−7	90.86	111.06
−21	7	8	−0.40	87.82
−6	−9	6	143.15	46.49
8	−24	−1	−6.47	72.11
1	−18	4	31.03	37.24
5	−23	−8	10.70	87.69
8	−30	−1	105.52	86.90
7	−32	0	37.37	99.97
−1	−24	5	50.71	54.15
4	−34	2	−7.13	75.14
2	−39	2	3.17	82.67
−15	22	−1	−38.83	64.84
−21	28	−1	101.29	72.63
−11	14	2	685.53	89.80
−29	32	2	−31.30	107.37
−1	2	−6	199.15	59.03

−3	4	−7	104.46	56.92
−16	16	4	249.73	77.39
3	−5	−1	1181.02	112.65
2	−4	−6	123.87	65.90
−21	19	5	16.11	76.20
−24	22	5	46.22	92.84
5	−9	−4	456.53	83.20
2	−6	−7	7.53	69.73
−22	17	6	87.16	97.06
−25	20	6	12.15	108.82
6	−13	−5	232.56	75.14
5	−15	−7	62.86	77.65
4	−16	2	251.44	57.58
7	−20	−6	34.20	78.31
−28	15	8	65.77	89.54
8	−23	−5	−9.77	86.63
3	−18	−9	−15.45	92.84
−1	−21	5	0.00	40.15
−25	32	−3	−8.32	103.40
−24	30	0	18.62	90.59
−24	29	1	92.84	80.82
−29	34	0	−58.11	105.65
−18	22	2	18.22	75.80
−21	25	2	55.60	84.12
−26	28	3	141.31	106.04
2	−2	−2	117.27	42.39
1	−6	−8	−2.77	73.43
6	−15	−6	133.12	82.67
−22	13	7	−37.11	105.38
−1	−9	−10	−29.58	86.24
−18	8	7	−8.06	84.52
8	−19	−3	−8.72	78.71
4	−15	−8	0.00	105.78
2	−13	−9	−70.65	99.18
8	−20	−2	6.60	83.99
5	−20	−8	−37.51	97.59
1	−16	−10	−10.96	92.71
8	−32	−5	−19.28	88.48
5	−30	2	110.93	71.58
8	−35	−2	4.89	89.93
−5	−29	6	−35.00	49.92
0	−39	3	24.56	74.09
−18	25	−1	42.39	68.80
−29	33	1	0.00	110.27
1	0	−2	444.91	52.30
−25	25	4	105.91	99.05
−28	19	7	9.77	84.65
7	−20	0	78.97	81.61
−20	5	8	36.71	86.63
−25	5	9	0.26	89.27
8	−33	−1	52.56	108.95
7	−37	−1	−40.81	88.61
5	−40	0	149.76	94.82
−23	30	−2	153.32	84.12

−15	20	1	247.09	91.65
−31	36	−1	0.00	88.88
−3	7	−4	1121.46	96.93
−7	10	1	977.65	95.88
−17	19	3	26.81	71.18
−23	25	3	53.88	76.60
−4	4	−8	316.02	67.35
−9	9	3	671.00	86.63
−19	19	4	140.51	77.52
3	−6	−6	59.30	64.45
−29	26	5	111.72	102.22
−13	9	5	81.35	73.82
3	−8	−7	58.77	71.31
7	−15	−2	66.82	67.48
−13	5	6	0.00	66.29
−25	16	7	−7.00	96.01
5	−21	2	93.50	57.18
9	−27	−3	−20.47	78.05
−9	−10	7	−32.35	62.86
9	−30	−3	120.97	108.29
−19	−7	9	−26.54	72.90
−12	−17	8	−20.47	45.56
−2	−31	5	−9.64	55.86
0	−34	4	−29.45	65.37
3	−38	2	−21.26	76.46
−12	19	−4	146.85	46.75
−17	24	−5	69.99	54.28
−15	21	0	35.66	76.99
−21	27	0	10.43	71.97
−21	26	1	15.45	79.10
−22	22	4	105.38	82.41
3	−4	−2	179.60	46.62
−30	29	4	−28.66	93.50
−17	14	5	143.42	77.26
2	−8	−8	1.45	73.03
6	−15	0	267.29	61.54
0	−11	−10	27.73	87.82
−24	10	8	25.49	83.07
3	−18	3	52.69	46.75
9	−26	−4	−8.19	80.03
7	−25	−7	85.44	101.16
9	−28	−2	39.88	73.82
9	−29	−4	0.00	103.27
8	−28	−6	35.79	102.48
−24	3	9	29.45	86.10
7	−35	0	2.64	87.03
6	−34	1	28.53	83.99
8	−37	−3	42.39	92.71
−18	−12	9	−4.49	64.97
−26	32	−1	1.45	101.82
−28	34	−2	−27.86	93.24
−2	4	−6	104.33	48.99
−20	22	3	75.27	75.80
−31	33	2	31.43	100.50

-3	0	-9	125.19	95.61
-2	-2	-9	135.49	98.65
-1	-4	-9	-3.96	100.89
7	-14	-3	1479.48	153.06
2	-9	2	639.31	58.37
7	-16	-1	337.94	70.39
6	-17	-7	72.37	84.78
-17	6	7	57.71	80.29
8	-20	-5	43.05	76.07
8	-21	-1	69.46	86.50
9	-24	-3	59.16	73.95
4	-20	-9	83.99	84.52
-19	3	8	48.47	86.63
8	-25	-6	-56.52	107.89
-28	8	9	-2.11	93.90
-23	1	9	6.74	86.76
9	-32	-4	-50.58	95.48
9	-33	-3	0.00	99.44
-20	-5	9	-59.96	78.18
5	-38	1	42.79	84.78
1	-38	3	-33.02	72.63
-18	24	0	105.78	69.46
-31	35	0	-10.30	88.08
-28	29	3	119.25	110.01
2	-4	0	427.48	41.34
-11	9	4	772.69	103.93
-26	23	5	169.04	110.14
4	-8	-6	8.85	64.45
6	-11	-4	301.36	79.37
4	-10	-7	0.13	68.41
-27	21	6	-19.41	97.20
8	-18	-4	152.53	74.09
3	-15	-9	-6.07	98.12
-27	13	8	5.15	82.41
9	-25	-2	-0.13	77.12
-22	-1	9	-17.17	81.09
-21	-3	9	13.87	80.03
-1	-27	5	-18.75	50.71
-4	-25	6	4.49	35.66
6	-39	0	35.26	87.56
-14	21	-2	262.27	66.03
-33	37	-1	54.15	84.92
-31	34	1	-94.56	94.56
-4	6	-7	15.45	49.92
-13	14	3	247.22	84.52
-5	5	2	144.61	44.50
4	-7	-1	2246.49	193.60
-20	17	5	13.21	71.05
0	-6	-9	-49.39	99.57
-24	18	6	86.37	103.67
3	-10	-8	196.51	87.29
7	-15	-5	109.21	69.99
-12	3	6	171.42	66.82
-21	11	7	98.25	101.82

5	−17	−8	−25.09	101.69
9	−23	−4	263.46	86.24
−18	1	8	−23.11	82.67
9	−28	−5	−16.64	110.14
8	−28	0	−57.31	68.28
−15	−5	8	−5.94	64.84
9	−31	−5	40.01	92.18
5	−33	2	1.19	72.11
8	−38	−2	102.22	92.71
−21	28	−4	22.05	69.46
−22	29	−3	84.39	77.92
−26	31	0	0.00	99.18
−11	15	1	245.77	66.82
−26	30	1	−28.66	91.12
−14	17	2	544.36	93.76
−15	14	4	55.07	77.26
−27	26	4	−20.47	105.52
4	−6	−2	88.08	46.09
−23	20	5	22.05	85.84
−12	7	5	100.76	69.46
1	−13	−10	21.92	87.16
−16	4	7	20.07	74.75
8	−22	−6	66.43	87.16
−7	−7	6	402.92	60.75
−23	8	8	0.00	80.03
9	−25	−5	29.19	90.07
6	−22	−8	−92.71	91.78
8	−25	0	19.68	63.65
−17	−1	8	−2.51	82.27
7	−27	1	50.45	64.58
8	−31	0	−15.58	81.75
9	−34	−2	92.97	103.27
9	−35	−4	9.77	84.78
8	−36	−1	33.94	81.88
4	−37	2	67.09	75.54
−17	24	−2	126.65	67.88
−11	17	−1	375.45	69.73
−7	11	0	855.36	86.10
−5	6	−8	86.90	55.33
2	−2	−5	271.39	66.43
5	−10	−6	47.67	67.62
1	−8	−9	−2.25	100.63
−17	10	6	284.06	77.52
−24	14	7	26.68	95.35
−27	17	7	27.34	86.63
−27	6	9	26.94	93.63
4	−26	3	25.75	53.48
7	−30	1	55.20	71.58
9	−36	−3	−92.57	85.31
7	−40	−1	76.73	93.76
−13	20	−3	43.71	52.56
−27	33	−3	76.07	91.39
−23	26	2	185.02	78.58
−33	36	0	−27.73	85.97

−10	12	2	337.55	65.77
3	−4	−5	415.20	77.78
5	−12	−7	98.91	74.48
5	−14	1	−15.19	44.37
−11	1	6	8.85	59.03
9	−21	−3	−58.24	69.99
4	−17	−9	−74.09	96.93
0	−13	4	0.00	13.07
7	−24	1	56.79	61.14
−10	−8	7	27.07	60.35
9	−29	−1	43.71	71.84
2	−37	3	78.05	71.45
1	−43	2	33.81	85.84
−8	14	−4	9.77	30.51
−23	29	−1	71.71	71.31
−11	16	0	484.53	73.69
−28	33	−1	11.89	103.80
−30	35	−2	−48.47	87.69
0	2	−2	122.29	28.00
1	0	−5	99.31	60.35
−25	26	3	53.35	85.97
−30	30	3	43.58	104.06
−8	7	3	197.96	55.86
−24	23	4	−7.66	79.76
4	−6	−5	128.89	72.24
−16	12	5	115.42	70.92
7	−13	−4	125.19	77.52
−29	22	6	6.74	87.03
4	−12	−8	−24.43	87.03
8	−17	−2	59.43	76.86
7	−19	−7	64.84	95.74
9	−22	−2	99.05	73.16
−26	11	8	50.05	81.75
8	−27	−7	65.24	89.93
9	−32	−1	−68.80	97.06
4	−29	3	4.49	63.65
−13	−12	8	−19.68	53.75
−19	−10	9	44.24	66.69
7	−38	0	20.60	78.71
6	−37	1	−57.84	78.84
−3	−35	5	0.00	62.60
−15	22	−4	83.86	53.75
−18	25	−4	12.81	55.07
−19	26	−3	69.07	61.94
−17	20	2	123.61	70.52
−20	23	2	4.23	80.95
−18	17	4	74.88	79.37
5	−8	−2	826.44	85.31
−28	24	5	−31.17	100.37
8	−16	−3	259.90	81.09
2	−10	−9	−2.38	99.97
−10	−1	6	70.92	57.18
−20	9	7	85.05	98.12
9	−22	−5	−42.79	76.99

6	−19	−8	18.75	96.27
2	−15	−10	31.56	85.58
−8	−5	6	2.64	50.84
−22	6	8	−77.65	81.35
9	−27	−6	−65.90	102.22
10	−29	−3	−3.70	82.41
9	−30	−6	−0.40	98.91
−26	4	9	−36.98	85.71
2	−26	4	53.88	54.67
8	−34	0	−97.73	89.54
7	−33	1	199.68	97.73
1	−33	4	71.05	61.14
4	−42	1	27.07	86.76
2	−42	2	27.20	83.99
−25	31	−2	−7.40	96.54
−23	27	1	9.11	77.52
−16	17	3	−28.53	78.31
−21	20	4	14.92	75.67
−29	27	4	31.56	100.50
3	−6	0	28.39	42.26
−10	7	4	1751.53	165.08
8	−17	−5	41.20	71.18
9	−20	−4	43.71	76.60
8	−19	−6	49.26	74.61
−9	−3	6	175.24	58.11
10	−28	−4	13.47	94.56
4	−23	3	69.86	52.69
10	−31	−4	−15.05	114.89
10	−32	−3	−117.40	111.20
9	−37	−2	70.78	87.95
−1	−38	4	9.77	65.11
−16	23	−3	301.10	65.37
−6	11	−5	24.96	14.79
−23	28	0	110.01	70.39
−28	32	0	5.28	111.06
−28	31	1	−54.01	108.69
−22	23	3	89.54	74.61
5	−8	−5	196.37	78.05
5	−9	−1	710.22	76.73
6	−12	−6	185.28	74.22
−26	19	6	58.37	110.27
−16	8	6	101.42	68.94
−29	18	7	67.62	83.20
8	−22	0	213.28	74.35
−14	0	7	262.80	72.11
10	−26	−3	130.08	84.65
7	−24	−8	−5.41	96.27
−11	−6	7	−14.92	62.86
6	−26	2	58.50	59.69
6	−29	2	62.60	61.14
−25	2	9	31.30	82.93
8	−39	−1	−6.60	80.82
−20	26	−1	62.46	69.73
−14	18	1	601.54	96.80

−32	36	−2	102.74	84.78
−3	6	−6	237.71	48.33
0	2	−5	303.61	59.43
−6	8	1	1320.48	121.36
−19	20	3	0.00	66.82
3	−3	−3	1123.18	104.20
−19	15	5	13.21	77.92
−25	21	5	0.40	93.76
6	−14	−7	−8.58	71.05
5	−14	−8	77.78	98.39
3	−12	−9	47.28	97.06
8	−18	−1	12.15	74.35
7	−17	0	1038.66	111.59
−23	12	7	45.30	98.52
−26	15	7	−40.67	93.10
9	−23	−1	0.40	69.99
5	−19	−9	29.32	86.63
10	−25	−4	26.15	74.22
9	−24	−6	−4.23	101.03
−13	−2	7	67.75	64.58
−12	−4	7	6.47	62.33
10	−30	−5	0.00	96.01
10	−30	−2	−51.50	79.63
2	−23	4	16.51	42.26
10	−34	−4	−52.56	81.61
9	−35	−1	−52.16	87.42
−20	−8	9	−4.36	69.99
5	−36	2	63.65	71.31
5	−41	1	41.07	84.39
3	−41	2	43.84	76.20
−14	20	−1	60.75	72.50
−30	31	2	88.74	100.76
4	−5	−3	374.66	57.97
−4	3	2	198.22	46.49
−14	12	4	−1.32	78.05
−22	18	5	29.58	71.31
−30	25	5	−41.20	92.44
3	−17	−10	124.40	83.99
−25	9	8	26.54	79.37
−28	12	8	38.30	84.12
10	−27	−5	140.51	102.08
10	−27	−2	18.49	71.58
−21	4	8	10.56	77.12
10	−33	−5	24.70	82.27
10	−33	−2	39.22	106.97
−24	0	9	5.28	78.05
2	−29	4	−3.04	58.77
4	−32	3	0.00	64.84
−1	−30	5	57.18	51.37
−19	−13	9	−28.39	61.67
3	−36	3	0.00	67.22
0	−42	3	−9.90	66.43
−17	23	−1	0.00	62.99
−5	10	−3	539.60	49.39

−30	34	−1	0.00	93.63
−25	27	2	20.73	84.39
−12	12	3	906.73	106.84
−27	27	3	−93.50	94.82
−31	28	4	−7.26	90.46
8	−15	−4	106.44	82.41
−19	7	7	12.28	83.07
8	−21	−7	54.15	99.44
5	−18	2	240.88	60.75
−23	−2	9	−24.30	77.39
6	−32	2	15.72	68.54
−22	−4	9	−23.37	75.54
−21	−6	9	14.92	71.97
8	−37	0	9.51	79.50
0	−37	4	94.56	65.63
−10	16	−2	155.57	52.82
−6	11	−2	531.02	55.99
−20	25	0	86.76	69.73
5	−7	−3	451.52	68.67
−26	24	4	13.07	90.73
6	−10	−2	1034.57	103.93
−15	10	5	79.10	66.43
7	−14	−6	768.73	103.27
1	−10	−10	−12.15	78.18
4	−14	−9	116.48	90.33
7	−21	−8	64.18	92.31
7	−21	1	424.97	81.88
−3	−11	5	91.65	21.92
−28	5	9	23.11	83.33
−14	−10	8	26.02	58.37
7	−36	1	73.69	77.26
−10	16	−5	108.16	19.41
−22	28	−2	97.59	69.20
−24	30	−3	−1.85	91.91
−25	30	−1	0.00	80.43
−30	33	0	100.50	99.05
−30	32	1	−10.70	94.95
4	−8	0	370.70	52.16
−28	20	6	3.57	92.44
7	−16	−7	49.52	75.14
9	−19	−5	226.62	81.75
6	−16	−8	30.90	104.46
2	−13	3	4.36	28.13
10	−23	−3	71.58	71.18
10	−24	−5	113.04	74.22
6	−23	2	1.06	55.07
−20	2	8	12.81	76.07
9	−30	0	100.23	72.63
10	−36	−2	60.48	87.56
7	−41	0	−38.03	83.07
4	−40	2	−66.16	71.45
−2	−34	5	−12.68	55.99
1	−41	3	−19.15	68.28
−17	22	0	108.16	74.48

−27	32	−2	0.00	96.80
−5	8	−7	72.50	43.84
−13	15	2	547.13	92.18
−32	32	2	−101.82	90.73
−7	5	3	483.08	66.69
−9	5	4	628.21	88.22
6	−11	−1	114.36	45.30
−27	22	5	64.05	103.14
−19	11	6	63.92	71.58
9	−18	−3	−44.24	82.41
−15	6	6	234.67	71.71
9	−19	−2	165.60	82.54
9	−21	−6	0.40	75.27
10	−24	−2	68.80	71.84
9	−26	−7	−0.92	94.95
−24	7	8	16.38	77.12
9	−27	0	22.58	69.46
10	−29	−6	1.32	92.97
9	−38	−1	121.89	85.18
6	−40	1	4.75	80.69
−10	13	1	198.75	54.41
−25	28	1	52.96	75.80
−32	35	−1	0.00	79.76
−22	24	2	74.48	73.43
−9	10	2	101.16	53.35
−24	24	3	30.11	76.07
−17	15	4	37.77	75.01
−23	21	4	49.26	76.07
2	−12	−10	−17.56	80.43
10	−22	−4	253.82	78.84
−22	10	7	−1.72	103.40
−28	16	7	0.00	79.63
−18	5	7	−29.71	80.29
6	−21	−9	14.00	77.12
−19	0	8	−48.07	74.48
10	−31	−1	38.30	85.31
8	−29	1	17.83	63.92
9	−33	0	−2.91	91.91
−27	3	9	−26.02	83.59
−4	−28	6	336.10	40.01
−23	29	−4	27.20	70.12
−25	29	0	−18.22	77.39
−29	28	3	2.38	106.31
−20	18	4	140.12	73.43
6	−9	−3	22.05	51.77
5	−16	−9	39.88	87.16
−25	13	7	58.50	86.50
10	−26	−6	42.52	101.95
−27	10	8	7.79	81.35
11	−30	−4	104.06	107.10
11	−31	−3	69.33	104.72
11	−33	−4	42.00	86.37
−15	−8	8	−5.55	59.03
8	−32	1	12.81	87.82

−13	−15	8	−13.73	47.41
−20	−11	9	18.49	64.58
2	−40	3	0.00	66.03
−9	15	−3	143.15	48.86
−16	18	2	97.86	84.12
−19	21	2	83.33	67.75
−32	34	0	49.39	87.16
−27	28	2	2.25	98.12
−32	33	1	33.81	94.03
−15	15	3	76.73	81.48
2	−5	−8	13.34	66.29
−28	25	4	91.25	111.59
7	−12	−2	270.86	63.92
−18	13	5	68.41	76.07
−24	19	5	−57.31	80.16
9	−17	−4	88.48	72.90
−30	21	6	−27.73	84.25
6	−16	1	96.14	53.09
8	−23	−8	−22.98	84.92
11	−28	−3	62.46	78.31
−18	−2	8	13.60	73.43
11	−34	−3	24.70	90.59
10	−34	−1	23.90	100.89
−26	1	9	−8.98	79.76
6	−35	2	49.13	68.01
2	−32	4	123.21	59.69
−5	−32	6	−12.41	49.39
−34	36	−1	−125.46	82.27
−21	21	3	46.62	79.37
3	−5	−7	153.32	67.62
1	−3	−8	−5.02	64.71
3	−7	−8	120.18	68.67
−9	1	5	103.27	66.43
8	−18	−7	30.51	84.65
7	−18	−8	0.00	97.06
3	−14	−10	80.82	79.24
11	−27	−4	−50.18	76.60
11	−29	−5	−15.98	103.80
8	−26	1	12.94	62.20
−23	5	8	0.00	76.07
11	−32	−5	16.77	89.01
10	−39	−2	46.09	78.71
4	−35	3	32.09	62.99
8	−40	0	−47.41	78.71
5	−39	2	−10.43	68.28
−19	25	−2	18.22	61.67
−1	4	−5	294.63	55.20
−18	18	3	138.27	72.24
2	−3	−7	−39.22	61.94
0	−1	−8	−8.85	61.67
−31	29	3	−28.66	88.35
4	−7	−7	177.23	67.35
−13	10	4	396.32	88.35
7	−11	−3	1026.11	110.14

-21	16	5	21.66	70.52
-29	23	5	22.98	99.05
3	-11	2	526.40	54.01
-14	4	6	284.72	71.97
9	-20	-1	354.98	83.73
10	-25	-1	16.11	68.41
4	-20	3	184.89	47.28
-9	-13	7	3.57	51.90
9	-36	0	14.53	79.37
-21	-9	9	54.28	66.95
7	-39	1	-24.17	78.97
-13	19	-2	150.15	62.20
-21	27	-3	-38.96	63.52
-22	27	-1	80.43	65.90
-27	31	-1	-5.41	98.65
-29	33	-2	-35.39	90.86
-34	35	0	0.00	79.90
-3	1	2	384.17	48.60
1	-5	-9	-70.92	91.91
5	-10	0	-11.09	40.41
4	-9	-8	46.49	74.48
8	-14	-5	41.60	67.35
-14	8	5	2.38	59.56
10	-21	-5	191.22	78.44
8	-19	0	217.77	70.65
6	-18	-9	-22.19	83.59
10	-23	-6	68.80	97.99
-21	8	7	-12.15	96.01
-17	3	7	99.71	73.43
11	-29	-2	13.73	75.67
-29	11	8	14.53	77.92
-29	4	9	-26.15	81.22
11	-37	-3	-1.98	83.07
-25	-1	9	0.00	74.35
10	-37	-1	-67.62	76.86
8	-35	1	-20.47	78.58
9	-41	-1	11.62	79.50
-16	22	-2	-10.43	57.58
-10	15	-1	214.86	54.28
-26	31	-3	1.58	93.10
-10	14	0	1153.95	112.52
-6	9	0	445.97	57.31
-27	29	1	-92.18	89.54
1	-1	-7	18.22	57.58
-11	10	3	620.95	86.63
-26	25	3	-54.67	82.80
0	-3	-9	64.18	88.48
5	-9	-7	83.73	67.48
-30	26	4	110.80	99.97
2	-7	-9	129.29	95.74
-8	3	4	119.12	59.03
7	-13	-1	39.35	56.39
-18	9	6	58.90	66.82
10	-20	-3	-67.09	72.24

4	−16	−10	90.86	83.46
11	−25	−3	48.20	74.09
11	−26	−5	57.05	91.25
10	−28	−7	0.00	80.56
−26	8	8	14.79	76.46
5	−28	3	−17.43	56.79
11	−35	−2	−4.49	83.46
−24	−3	9	78.84	73.56
−22	−7	9	−33.15	66.95
3	−39	3	5.55	64.58
−19	25	−5	−14.66	55.07
−22	26	0	9.90	73.82
−27	30	0	−55.99	89.54
−25	22	4	−22.19	79.10
5	−11	−8	0.00	80.69
3	−9	−9	−43.71	92.97
−8	−1	5	175.38	62.46
10	−21	−2	129.82	77.78
11	−24	−4	−8.32	71.58
−24	11	7	67.22	88.88
−27	14	7	30.11	88.88
−22	3	8	5.81	70.65
11	−31	−6	−10.30	81.22
−23	−5	9	14.66	71.18
−1	−33	5	37.77	51.37
3	−44	2	−5.28	76.86
−24	29	−2	−29.45	69.60
−13	16	1	232.69	72.11
−24	25	2	70.92	72.24
−1	1	−8	2.25	57.58
−1	−1	−9	69.46	88.88
−6	3	3	691.21	74.88
6	−11	−7	53.62	67.75
8	−14	−2	193.07	65.11
−26	20	5	−24.56	94.16
−31	24	5	−49.52	86.10
10	−19	−4	258.31	73.16
−21	12	6	−9.51	70.92
9	−20	−7	−8.06	88.88
8	−20	−8	−58.50	95.48
11	−26	−2	72.11	74.09
9	−25	−8	79.50	80.69
11	−28	−6	76.60	95.35
2	−20	4	8.98	37.37
7	−28	2	20.34	54.81
7	−31	2	36.85	64.97
5	−31	3	0.00	60.75
−8	−18	7	90.20	25.09
−28	2	9	−40.81	77.78
−13	−18	8	−9.24	44.11
−13	−21	8	−139.98	32.75
−20	26	−4	−29.98	58.37
−6	10	−1	1413.85	122.95
−19	22	1	40.81	66.69

−5	6	1	1236.62	115.03
6	−8	−4	358.68	73.95
−16	13	4	162.17	68.54
8	−13	−3	425.37	83.20
9	−16	−5	40.94	68.94
4	−11	−9	1.45	94.82
−13	2	6	172.87	64.71
8	−23	1	29.19	55.73
−16	1	7	28.79	72.90
−1	−18	5	−41.34	15.45
11	−38	−2	−7.26	76.46
9	−39	0	−85.05	76.07
6	−38	2	−0.13	64.05
6	−43	1	−23.24	76.20
4	−43	2	−34.34	75.41
−19	24	−1	−1.19	61.41
−16	19	1	149.36	85.05
−29	32	−1	−10.56	96.93
−31	34	−2	0.00	79.10
0	1	−7	134.17	57.05
4	−5	−6	18.88	59.82
5	−7	−6	37.24	60.75
−22	19	4	3.43	70.78
−17	11	5	12.68	69.07
6	−13	−8	−57.84	82.27
7	−20	−9	42.79	75.94
5	−18	−10	−48.07	79.24
12	−32	−4	−17.43	92.57
5	−25	3	−0.40	48.99
12	−33	−3	42.79	94.56
10	−32	0	22.32	81.22
12	−35	−4	37.90	80.03
−14	−13	8	−8.32	52.16
10	−40	−1	46.22	74.88
−18	24	−3	168.64	63.79
−19	23	0	289.87	71.97
−12	13	2	181.98	63.13
−29	30	1	16.51	99.31
3	−3	−6	532.73	78.05
−23	22	3	51.77	76.60
−28	26	3	−1.58	94.82
6	−9	−6	34.73	62.33
−19	16	4	201.53	76.86
7	−13	−7	−1.06	65.50
−23	17	5	23.64	70.92
5	−13	−9	100.63	88.35
−7	−3	5	230.58	58.77
11	−23	−5	−9.38	75.67
−4	−9	5	0.92	33.02
11	−25	−6	15.05	94.95
6	−20	2	38.17	58.11
−20	6	7	0.00	86.24
−29	15	7	12.15	75.80
12	−29	−4	0.00	88.48

12	−30	−3	197.17	96.93
12	−31	−5	−40.81	89.27
11	−30	−1	53.48	71.45
10	−29	0	−22.32	63.92
−25	6	8	16.64	71.45
−28	9	8	4.62	81.88
−21	1	8	−35.39	73.69
11	−33	−1	51.77	93.37
7	−34	2	−12.68	66.03
−27	0	9	2.91	74.88
8	−38	1	9.77	69.46
2	−35	4	−2.91	55.99
8	−43	0	−94.69	80.03
−13	18	−1	−11.36	63.52
−29	31	0	149.10	101.82
3	−2	−4	104.99	48.07
−21	22	2	0.00	73.16
−8	8	2	607.88	70.92
7	−10	−4	1269.50	131.40
−27	23	4	27.60	92.84
6	−12	0	213.41	45.56
−20	14	5	37.90	73.82
10	−20	−6	37.51	77.65
−17	7	6	357.75	73.69
10	−22	−1	72.90	69.99
−15	−1	7	21.53	68.41
10	−35	0	72.37	82.80
4	−38	3	70.39	63.52
−12	18	−3	51.24	53.48
−16	22	−5	9.38	45.03
−16	21	−1	−10.83	62.99
−24	28	−1	40.94	71.18
−28	32	−3	48.99	87.16
−24	26	1	0.00	69.60
−33	35	−2	42.52	76.46
−14	13	3	74.75	73.29
7	−11	−6	361.85	76.60
−12	8	4	136.68	73.03
8	−15	−1	60.88	54.94
2	−9	−10	−85.84	73.03
−28	21	5	92.31	97.20
7	−15	−8	−59.03	93.50
11	−22	−3	49.13	78.18
7	−18	1	589.12	80.82
9	−22	−8	0.00	85.31
−23	9	7	31.17	89.41
−26	12	7	38.83	80.16
12	−28	−5	39.09	98.91
7	−25	2	44.37	55.73
−10	−11	7	47.54	63.79
9	−31	1	1.98	68.80
11	−36	−1	0.00	78.44
3	−28	4	11.62	52.43
−26	−2	9	0.00	71.71

0	−29	5	−3.17	45.16
7	−42	1	−76.86	77.39
5	−42	2	21.39	68.28
−13	19	−5	6.21	39.35
−4	8	−6	55.07	36.05
−16	20	0	0.00	77.39
−9	11	1	688.70	76.99
−31	33	−1	9.24	86.90
2	−1	−6	251.18	61.28
−2	3	−8	32.62	55.60
−26	26	2	80.03	76.46
−31	31	1	−63.26	96.67
−20	19	3	65.77	70.78
9	−15	−3	168.11	77.12
−7	1	4	138.14	55.60
9	−16	−2	223.18	77.78
8	−15	−7	32.09	69.33
10	−18	−5	107.37	68.41
3	−11	−10	33.28	72.24
6	−15	−9	29.58	84.52
−6	−5	5	83.33	50.45
10	−22	−7	−80.43	99.84
9	−21	0	53.88	73.03
−5	−7	5	112.65	41.86
−12	0	6	360.92	68.14
12	−26	−4	−28.53	72.90
12	−27	−3	−49.39	78.31
11	−27	−1	−67.35	70.26
−20	−1	8	30.51	71.45
12	−34	−2	−2.11	99.05
−22	−10	9	−12.28	64.84
−15	21	−3	42.13	52.69
−17	23	−4	15.72	52.56
−26	30	−2	31.03	88.22
−24	27	0	1.85	71.31
−15	16	2	418.50	86.37
−18	19	2	83.46	70.26
−35	36	−2	−14.13	75.41
−17	16	3	−38.56	78.18
−30	27	3	93.37	98.39
8	−12	−4	168.25	74.75
11	−21	−4	128.63	72.90
11	−23	−2	194.00	73.03
8	−22	−9	−9.51	73.95
11	−27	−7	5.28	80.43
10	−26	0	90.59	65.90
−14	−3	7	153.72	68.67
12	−30	−6	47.01	84.39
9	−28	1	0.00	59.30
−24	4	8	1.32	70.65
9	−34	1	17.30	80.95
−15	−11	8	−3.70	55.33
3	−31	4	29.85	55.33
−25	−4	9	35.92	69.86

−11	17	−4	33.68	40.28
−21	26	−2	154.78	63.65
−23	28	−3	24.96	66.43
−31	32	0	1.85	95.22
−10	8	3	155.83	58.24
−29	24	4	66.69	103.54
8	−17	−8	−0.40	98.12
4	−13	−10	16.11	77.78
−20	10	6	113.18	66.82
−23	13	6	70.12	81.09
−19	4	7	0.00	86.10
−11	−9	7	0.00	66.56
−27	7	8	40.41	77.52
12	−37	−2	0.00	77.12
10	−38	0	−66.16	71.84
−29	1	9	−29.05	74.75
−24	−6	9	0.00	69.73
−23	−8	9	−46.09	64.58
9	−42	0	0.40	73.29
1	−39	4	0.00	57.18
−1	3	−7	0.53	49.52
−33	34	−1	52.96	76.86
−33	32	1	105.12	86.10
−25	23	3	−16.64	68.54
−5	1	3	346.00	50.98
−24	20	4	−35.52	69.73
−25	18	5	0.00	80.16
−30	22	5	0.00	90.07
4	−13	2	71.18	35.39
11	−22	−6	55.20	74.88
12	−25	−5	−23.64	77.52
12	−28	−2	39.62	67.88
−13	−5	7	0.00	63.79
−12	−7	7	16.51	67.62
3	−25	4	0.00	44.64
0	−23	5	24.70	41.60
11	−39	−1	−40.41	73.69
−4	−31	6	−30.24	41.60
−30	33	−3	149.89	79.63
2	0	−4	552.28	65.24
−33	33	0	−32.88	80.82
−32	28	3	41.47	85.84
9	−14	−4	260.16	87.29
7	−14	0	1525.70	139.98
9	−17	−7	0.00	77.39
7	−17	−9	59.82	80.56
−11	−2	6	127.84	56.39
10	−24	−8	52.96	77.26
12	−27	−6	−47.54	102.74
−28	13	7	29.05	75.14
−6	−12	6	−14.66	34.60
13	−34	−4	83.86	89.27
−18	−5	8	−24.56	65.63
−16	−9	8	−3.17	57.18

9	−37	1	29.85	67.88
8	−41	1	−5.41	73.03
6	−41	2	9.24	61.41
3	−42	3	−33.54	63.79
−14	20	−4	−0.40	46.09
−26	27	1	43.18	69.99
−35	35	−1	54.67	72.37
−28	27	2	0.00	83.20
−15	11	4	24.17	70.78
9	−15	−6	241.54	78.84
−31	25	4	16.51	84.65
10	−17	−3	635.61	103.01
−16	9	5	61.14	72.11
9	−17	−1	13.47	65.50
11	−20	−5	0.66	69.33
5	−15	−10	−22.19	72.63
−16	5	6	33.54	62.99
13	−31	−4	0.00	94.82
13	−33	−5	80.43	82.01
−23	2	8	−35.79	68.80
−17	−7	8	8.19	59.82
−28	−1	9	0.00	70.52
5	−37	3	0.00	58.64
−26	29	−1	99.05	77.52
1	1	−6	218.69	54.67
−23	23	2	55.73	68.80
−35	34	0	−26.41	73.82
−2	−1	2	454.95	49.39
−21	17	4	284.46	77.52
−22	15	5	70.52	71.45
10	−18	−2	82.93	81.22
9	−19	−8	−34.20	98.12
12	−24	−3	65.24	74.22
3	−15	3	140.64	40.01
−22	7	7	17.56	88.08
−25	10	7	−33.54	79.24
13	−30	−5	13.07	96.14
13	−32	−3	−1.32	91.78
12	−40	−2	140.64	76.46
0	−32	5	46.49	46.88
−21	25	−1	159.93	67.48
−21	24	0	22.58	70.78
−28	31	−2	4.09	89.67
−26	28	0	−54.67	72.37
−18	14	4	177.49	72.24
−19	12	5	202.71	76.73
12	−23	−4	−21.39	74.48
8	−19	−9	4.09	78.97
11	−24	−1	25.88	65.50
−10	−4	6	314.70	60.48
9	−25	1	10.56	59.43
−18	2	7	−90.46	83.46
5	−22	3	28.00	45.16
12	−32	−1	−23.77	80.16

−26	5	8	−42.13	73.95
−29	8	8	62.73	76.60
12	−35	−1	−28.92	85.84
13	−38	−3	−64.58	73.82
−14	−16	8	20.21	47.01
−27	−3	9	28.13	69.46
10	−41	0	−90.86	76.07
3	−34	4	16.38	53.75
−9	14	−2	34.34	42.66
−18	23	−2	9.24	59.56
−22	27	−4	77.26	66.82
0	3	−3	1175.48	101.29
−12	14	1	592.16	80.43
6	−7	−5	171.94	78.31
7	−9	−5	124.01	82.01
−22	20	3	−11.89	71.58
−30	28	2	86.24	101.16
−27	24	3	−46.88	77.26
−11	6	4	467.89	80.03
−26	21	4	5.68	76.07
10	−16	−4	120.18	78.05
−27	19	5	−3.96	95.48
10	−19	−7	56.65	90.33
6	−17	−10	−53.35	78.18
−25	14	6	−8.85	93.90
13	−28	−4	−50.45	80.69
13	−29	−3	48.86	81.75
−30	14	7	−7.66	71.18
11	−31	0	80.95	71.84
8	−30	2	24.83	61.54
11	−34	0	65.24	87.03
8	−33	2	15.45	54.15
11	−42	−1	−7.53	73.69
2	−38	4	−4.62	54.67
−1	−36	5	−12.68	51.11
5	−5	−5	568.26	87.03
−28	28	1	27.86	84.12
−19	8	6	75.80	68.28
12	−24	−6	−31.56	92.71
12	−25	−2	145.53	66.82
−22	0	8	15.05	68.80
6	−30	3	−18.09	57.31
−26	−5	9	0.00	66.43
4	−41	3	−17.96	58.37
−2	6	−5	147.91	42.00
−6	10	−7	29.19	35.00
−25	29	−3	−23.11	79.10
−9	12	0	467.50	62.99
−3	5	−8	33.54	49.52
−15	17	1	248.01	75.67
−11	11	2	549.77	77.65
−13	11	3	−2.64	67.35
11	−19	−3	2.51	71.84
10	−21	−8	77.12	85.05

-22	11	6	-3.30	72.24
10	-23	0	43.45	61.01
13	-27	-5	11.75	91.25
7	-22	2	2.64	51.37
-9	-6	6	159.79	50.58
13	-29	-6	37.64	83.73
-7	-10	6	205.88	46.62
13	-36	-2	84.52	80.16
12	-38	-1	-23.24	75.94
11	-37	0	-7.00	72.11
6	-33	3	3.04	59.03
-25	-7	9	-0.92	66.43
7	-40	2	15.45	62.07
-24	-9	9	-12.55	66.29
-20	25	-3	7.66	60.09
-23	27	-2	18.75	62.86
-28	30	-1	16.24	91.12
-30	32	-2	0.00	84.39
-20	20	2	117.53	68.94
4	-6	-8	104.86	64.05
5	-8	-8	-4.09	66.95
-32	29	2	-6.34	90.20
6	-10	-8	9.90	72.24
8	-16	0	546.73	73.95
12	-22	-5	96.27	70.39
9	-21	-9	1.85	70.78
-15	3	6	297.80	69.86
12	-26	-7	48.20	81.22
-8	-8	6	53.75	45.03
-27	11	7	-18.09	73.29
11	-28	0	130.34	65.50
10	-33	1	70.78	86.50
8	-36	2	26.68	63.79
5	-45	2	48.86	67.22
4	-3	-5	275.61	68.28
-28	29	0	-46.49	82.67
-7	6	2	106.44	44.77
-25	24	2	1.45	76.73
-19	17	3	60.22	67.75
3	-6	-9	0.00	88.61
4	-8	-9	13.21	90.33
-29	25	3	117.53	92.05
5	-10	-9	-20.07	89.80
-28	22	4	-28.92	90.99
11	-20	-2	79.63	74.88
10	-19	-1	398.96	81.09
-29	20	5	0.00	91.78
11	-21	-7	-0.92	89.80
-21	5	7	-81.22	89.01
-24	8	7	38.30	80.56
13	-30	-2	22.32	71.58
-17	0	7	62.73	80.95
8	-27	2	49.65	54.81
6	-27	3	54.28	48.60

−25	3	8	45.03	71.84
−28	6	8	89.14	75.27
13	−39	−2	2.91	71.05
−29	−2	9	−21.13	69.86
8	−44	1	8.72	69.46
−15	20	−2	30.11	62.46
−18	22	−1	89.93	62.73
−18	21	0	161.77	70.92
3	−4	−8	−4.49	62.60
−30	29	1	−31.69	106.57
−16	14	3	52.69	77.26
7	−12	−8	159.27	78.05
−23	18	4	85.71	73.56
6	−12	−9	3.17	86.63
11	−18	−4	104.46	68.41
−24	16	5	43.71	70.39
−11	2	5	−27.07	60.48
13	−25	−4	8.19	68.28
−27	15	6	−3.17	93.50
13	−26	−3	−0.13	68.80
14	−33	−4	47.41	83.59
10	−30	1	0.00	61.28
14	−36	−4	−34.07	74.09
10	−36	1	0.00	68.54
−12	17	−2	24.70	55.73
−14	14	2	1258.15	133.65
−17	17	2	623.33	99.57
6	−8	−7	136.15	63.92
−9	6	3	45.43	51.37
8	−14	−8	36.45	85.18
14	−32	−5	−51.90	81.75
14	−34	−3	2.11	85.58
12	−41	−1	32.22	76.99
11	−40	0	−49.13	72.24
−15	−14	8	−4.36	49.52
−14	−19	8	10.17	43.18
5	−40	3	0.00	59.16
6	−44	2	0.00	65.37
−2	5	−7	103.67	48.33
−23	26	−1	139.98	68.28
−5	7	0	6.60	44.37
−23	25	0	−2.38	70.92
−30	31	−1	9.77	93.24
−32	33	−2	12.28	76.20
−4	4	1	4011.75	332.53
5	−6	−7	104.59	64.97
−24	21	3	24.70	71.45
8	−12	−7	44.77	65.63
10	−15	−5	3.30	64.31
−4	−1	3	446.76	53.48
−14	9	4	341.11	82.93
−31	26	3	97.73	96.93
7	−14	−9	82.01	82.54
−21	13	5	89.80	72.37

-31	21	5	9.77	80.95
11	-23	-8	128.50	81.22
13	-26	-6	-52.43	97.06
12	-26	-1	69.46	66.95
14	-30	-4	-72.90	104.99
-29	12	7	-18.36	69.07
14	-37	-3	75.94	75.27
-20	-4	8	12.15	63.52
-9	-16	7	5.94	40.01
6	-36	3	-21.66	57.18
-28	-4	9	0.13	69.20
1	2	-4	192.81	41.86
0	3	-6	535.90	68.28
-12	15	0	449.67	68.80
-30	30	0	0.00	94.42
-32	30	1	-15.45	94.69
-20	15	4	96.01	69.20
4	-10	-10	-36.98	69.33
-30	23	4	38.30	99.84
-18	10	5	121.10	67.75
13	-24	-5	12.15	66.43
-18	6	6	35.00	61.41
-24	12	6	-70.65	89.67
14	-31	-3	11.49	88.48
-16	-2	7	34.34	75.54
-30	7	8	124.27	76.60
-8	-21	7	0.00	26.02
-27	-6	9	9.11	66.43
3	-37	4	-4.09	55.73
-15	19	-1	4.36	68.67
-27	30	-3	-14.00	87.56
4	-4	-7	122.82	61.94
2	-2	-8	-16.38	58.11
-34	34	-2	16.38	70.12
1	-2	-9	-35.13	80.03
-27	25	2	3.70	76.07
9	-14	-7	34.47	66.43
-17	12	4	-5.28	62.86
-33	27	3	88.88	82.67
9	-16	-8	56.52	90.59
5	-12	-10	-17.43	70.92
12	-20	-4	37.11	64.84
8	-16	-9	0.00	75.41
12	-21	-6	9.38	71.45
12	-23	-7	-1.72	100.50
-21	9	6	22.98	66.43
-14	1	6	60.62	58.50
-29	16	6	82.80	80.43
13	-27	-2	-29.98	68.94
14	-29	-5	37.11	96.01
13	-28	-7	22.85	73.16
14	-31	-6	-65.50	79.63
-20	3	7	107.76	86.63
3	-22	4	30.64	36.71

13	−34	−1	−19.02	88.22
−24	1	8	−33.28	67.35
−27	4	8	−40.94	73.29
10	−39	1	12.94	68.28
8	−39	2	14.66	59.56
9	−43	1	20.60	69.86
0	−35	5	44.50	48.33
−24	28	−4	40.15	77.92
−25	28	−2	28.13	69.20
3	−1	−5	34.34	54.81
−8	9	1	30.24	44.11
−20	21	1	26.41	63.26
−32	32	−1	−2.51	83.33
−32	31	0	42.39	90.33
−36	35	−2	−29.32	72.37
−34	31	1	30.90	80.43
11	−17	−5	101.03	77.26
−10	4	4	199.28	66.29
6	−14	−10	1.19	69.86
−32	24	4	92.31	87.29
9	−18	0	79.63	63.79
−26	17	5	−3.70	80.56
10	−27	1	20.34	61.01
−26	9	7	42.26	78.58
−31	13	7	−36.98	71.31
12	−33	0	70.52	87.03
13	−37	−1	−1.72	72.11
−19	−6	8	−9.38	59.96
−16	−12	8	18.22	51.50
7	−43	2	−18.49	63.65
−19	24	−4	3.43	51.90
−12	16	−1	457.06	70.52
−22	21	2	36.98	71.05
−25	19	4	54.54	70.52
12	−22	−2	40.67	66.82
11	−21	−1	428.67	91.25
9	−22	1	72.63	62.33
−31	17	6	42.52	72.63
−23	6	7	−22.19	79.37
13	−31	−1	73.16	72.63
14	−35	−2	68.67	83.59
12	−36	0	19.15	77.12
−18	−8	8	26.41	59.56
−17	−10	8	−4.62	53.75
−3	−27	6	17.96	28.92
−17	22	−3	136.02	56.92
−20	24	−2	14.66	61.54
−34	33	−1	8.32	74.35
−34	32	0	57.18	76.07
−21	18	3	216.84	80.82
−26	22	3	39.35	71.31
4	−9	1	702.30	67.62
10	−16	−7	10.96	73.43
10	−18	−8	0.00	89.67

9	−18	−9	−28.79	75.27
7	−16	−10	64.71	72.77
−10	0	5	73.29	59.82
14	−27	−4	43.84	70.78
12	−25	−8	0.00	70.65
14	−28	−3	−14.26	68.41
−15	−4	7	38.83	77.78
14	−38	−2	88.48	75.54
13	−40	−1	−49.92	75.94
4	−33	4	−13.34	50.84
−29	31	−3	−4.75	83.86
3	−2	−7	−31.69	56.92
7	−9	−2	622.27	73.56
−36	34	−1	0.00	71.31
−29	26	2	−0.13	84.52
5	−11	1	58.11	34.60
4	−17	3	79.37	44.24
−26	13	6	12.94	88.48
14	−28	−6	0.00	86.50
8	−24	2	−2.38	52.56
12	−30	0	66.03	64.58
15	−34	−5	−18.49	76.60
15	−35	−4	−21.26	75.01
9	−32	2	23.77	61.67
−29	5	8	53.35	72.50
12	−39	0	0.00	68.94
6	−39	3	−2.91	57.18
−29	−5	9	−27.47	69.33
−18	23	−5	57.31	49.79
−22	26	−3	40.15	60.35
−25	27	−1	98.39	65.24
1	0	−8	21.92	55.99
−25	26	0	−5.81	68.67
7	−8	−6	33.68	59.82
8	−10	−6	−1.19	64.84
8	−10	−3	115.82	65.37
8	−11	−2	519.92	71.18
12	−19	−5	218.83	74.09
13	−22	−4	158.47	72.11
−23	14	5	0.26	66.69
13	−23	−6	113.31	82.80
13	−23	−3	51.37	69.33
−28	18	5	86.63	93.76
14	−26	−5	21.53	84.39
15	−32	−4	−1.45	89.14
6	−24	3	28.79	48.07
−28	10	7	81.88	70.39
4	−27	4	25.09	47.81
9	−35	2	54.94	63.52
−20	22	0	53.35	65.24
−17	18	1	303.74	85.44
7	−8	−3	210.37	57.05
6	−7	−2	164.68	50.84
9	−12	−6	42.39	67.62

9	−12	−3	513.59	84.65
−12	9	3	613.03	82.93
9	−13	−2	50.71	58.90
−31	27	2	−17.30	96.54
11	−18	−7	143.95	80.16
−27	20	4	25.09	83.99
11	−20	−8	0.00	87.69
−4	−5	4	470.40	58.90
10	−20	−9	14.66	70.39
8	−18	−10	−15.19	72.50
6	−17	2	195.71	57.45
15	−31	−5	−69.99	83.46
−19	1	7	−0.40	83.73
−14	−6	7	1.72	73.29
11	−35	1	−48.47	76.07
1	−28	5	4.89	41.07
10	−42	1	0.00	69.33
−20	23	−1	5.55	61.80
−27	29	−2	−8.06	80.16
6	−6	−6	0.00	56.92
−19	18	2	194.00	78.05
−18	15	3	49.65	75.01
10	−14	−6	237.97	72.37
3	−7	1	2488.16	206.28
−28	23	3	21.92	80.69
−22	16	4	192.81	73.03
6	−13	1	0.53	40.94
10	−20	0	301.50	79.63
−17	4	6	54.94	62.73
−23	10	6	−20.34	74.09
−13	−1	6	55.33	58.77
14	−29	−2	1.85	70.78
15	−33	−3	−9.77	101.42
9	−29	2	93.10	56.65
11	−32	1	165.34	76.73
7	−32	3	−3.83	57.58
−22	−3	8	−4.62	64.71
1	−31	5	−21.53	43.84
−15	−17	8	−51.50	47.28
8	−42	2	53.88	60.22
−5	9	−2	1413.05	119.91
−4	7	−8	131.66	46.88
−26	29	−4	−2.91	85.44
−11	12	1	79.24	54.41
−31	32	−3	33.81	76.46
6	−6	−3	254.61	55.33
−10	9	2	57.31	50.45
−24	22	2	45.96	74.75
−15	12	3	174.32	78.18
10	−14	−3	686.85	98.12
−33	28	2	16.51	87.95
−20	11	5	34.34	70.26
13	−24	−2	92.31	73.03
12	−23	−1	225.69	67.48

−30	19	5	54.54	85.84
−20	7	6	−42.26	64.84
−28	14	6	48.47	82.54
−22	4	7	−136.55	85.84
−25	7	7	96.80	74.35
−30	11	7	−25.62	70.92
11	−38	1	19.02	66.29
12	−42	0	−8.19	71.18
−3	−30	6	0.00	31.96
5	−43	3	−59.56	59.96
−8	13	−3	257.12	45.43
−14	19	−3	73.82	52.69
−14	15	1	290.14	76.46
−22	22	1	−1.98	67.09
−27	27	0	30.77	67.88
11	−16	−6	199.15	73.29
10	−15	−2	289.74	74.48
−13	7	4	98.65	74.09
13	−21	−5	67.75	74.61
12	−20	−7	144.74	88.08
−29	21	4	21.39	91.52
15	−29	−4	5.68	82.27
−13	−8	7	55.73	71.45
7	−29	3	11.62	54.54
−11	−12	7	24.70	53.09
4	−36	4	0.00	52.16
−5	8	−1	100.10	34.20
5	−4	−6	87.29	57.71
−27	28	−1	37.37	71.58
−33	33	−3	39.88	65.63
8	−9	−4	577.50	86.76
−16	15	2	291.85	81.48
9	−11	−4	858.92	109.87
−29	27	1	1.06	78.84
−8	4	3	380.07	58.77
−23	19	3	120.44	71.31
−19	13	4	132.19	74.09
−30	24	3	−21.66	99.31
12	−22	−8	−25.49	84.12
−25	15	5	−23.11	75.14
14	−25	−6	51.24	90.59
11	−22	−9	−32.62	68.28
−32	20	5	40.94	78.44
14	−27	−7	5.02	80.95
15	−30	−6	0.00	78.18
15	−30	−3	65.90	82.14
12	−27	0	29.71	69.99
14	−36	−1	123.74	81.09
−12	−10	7	6.60	65.90
−25	0	8	0.00	74.09
3	−40	4	20.87	52.30
2	0	−7	59.96	54.28
−29	30	−2	15.45	94.16
5	−5	−2	96.40	44.90

−13	12	2	372.68	71.97
10	−13	−4	295.29	86.63
7	−10	−1	1087.13	104.06
11	−16	−3	149.23	76.86
−16	10	4	102.88	76.33
7	−15	1	52.30	45.69
14	−24	−4	111.06	72.50
14	−25	−3	98.52	70.12
−9	−2	5	−1.19	54.54
15	−28	−5	72.77	95.22
10	−24	1	174.45	61.41
−30	15	6	60.62	80.16
11	−29	1	−2.38	57.97
14	−33	−1	2.38	87.16
−18	−1	7	38.83	84.12
15	−37	−2	69.33	75.27
14	−39	−1	46.22	69.60
−21	−5	8	16.11	63.92
−16	21	−4	−29.71	50.84
−17	21	−2	204.83	60.09
2	1	−5	41.20	53.62
−6	4	2	164.68	45.69
−31	28	1	20.34	98.78
8	−12	−1	283.93	55.99
−3	−3	3	649.87	63.52
−9	2	4	55.86	55.99
−24	17	4	88.35	68.67
−32	25	3	−84.78	98.91
−31	22	4	−24.56	89.93
1	−15	4	−13.21	21.00
−25	11	6	−10.04	101.95
15	−34	−2	−27.60	97.33
−27	8	7	23.51	69.33
13	−35	0	−12.15	79.50
15	−40	−2	−31.17	71.31
13	−38	0	26.28	68.01
−30	4	8	0.00	72.90
7	−46	2	0.00	64.05
−11	16	−3	12.68	51.11
−22	25	−2	3.30	59.56
−24	27	−3	6.34	66.29
0	2	−8	59.82	51.77
−29	28	0	−33.81	84.39
6	−8	−1	1430.75	130.87
−26	23	2	128.76	70.39
11	−15	−4	−12.28	80.56
−1	−3	2	123.34	25.75
9	−14	−1	22.85	51.64
11	−17	−2	152.53	72.11
−34	26	3	26.54	81.61
14	−23	−5	23.51	67.88
13	−22	−7	−19.55	90.86
−33	23	4	22.85	77.12
−12	−3	6	134.83	59.30

−32	16	6	−43.71	76.46
16	−33	−5	67.75	81.75
16	−34	−4	21.53	75.94
13	−32	0	14.79	73.95
−20	−7	8	−1.72	64.05
7	−38	3	10.70	53.48
−16	−15	8	−54.67	50.71
9	−41	2	−57.71	60.62
1	−34	5	11.23	44.11
10	−45	1	45.16	68.67
6	−42	3	−14.66	54.15
−14	19	−6	14.92	36.45
−21	25	−4	36.85	58.11
5	−4	−3	270.06	50.98
−22	23	0	22.45	66.16
−29	29	−1	−63.79	93.76
−31	31	−2	−12.28	81.09
8	−11	−8	−28.66	73.82
6	−9	−9	−31.96	85.31
7	−11	−9	7.53	87.16
−33	29	1	0.00	85.05
8	−13	−9	0.00	81.35
12	−18	−3	−5.55	80.69
−13	3	5	76.07	59.43
13	−24	−8	1.06	73.16
11	−22	0	113.31	69.73
−27	16	5	64.31	90.86
14	−26	−2	110.01	68.28
7	−19	2	187.39	61.80
−16	2	6	−37.90	62.07
−21	2	7	−40.15	77.26
−24	5	7	7.53	78.31
16	−38	−3	−36.45	69.33
−24	−2	8	−17.43	68.54
−27	1	8	107.37	69.99
14	−42	−1	−0.26	72.90
−15	−20	8	−61.54	46.35
−17	20	−1	−48.86	69.46
−22	24	−1	71.18	65.11
−24	23	1	−20.21	69.20
7	−9	−8	−5.28	69.20
5	−7	−9	24.43	87.16
−21	19	2	24.43	68.80
−31	29	0	18.22	93.10
9	−13	−8	106.71	82.67
−20	16	3	172.74	67.62
12	−17	−4	21.53	67.62
−25	20	3	−28.53	73.16
−35	30	1	−64.97	80.82
9	−15	−9	104.99	78.31
−3	−7	4	1446.07	126.25
−22	12	5	18.09	72.11
15	−27	−6	26.94	95.22
13	−25	−1	−38.03	69.46

-22	8	6	50.32	72.11
15	-31	-2	105.52	87.42
14	-30	-1	3.83	66.29
9	-26	2	61.67	56.92
16	-35	-3	21.13	82.41
-29	9	7	43.05	69.60
1	-25	5	33.68	44.90
13	-41	0	21.00	69.73
-19	-9	8	0.00	55.86
-17	-13	8	-0.79	52.82
-19	23	-3	111.86	60.35
4	-2	-6	124.53	57.18
-8	10	0	12.28	42.26
-31	30	-1	-31.17	92.57
-33	32	-2	-1.85	73.95
-35	33	-2	58.37	68.94
-28	24	2	82.01	73.29
10	-15	-8	-34.07	90.73
6	-11	-10	-24.04	69.60
10	-16	-1	50.05	63.39
7	-13	-10	71.45	70.78
12	-19	-2	299.91	85.71
15	-26	-4	50.71	73.03
5	-19	3	140.25	46.88
-19	5	6	9.64	63.13
16	-31	-4	39.35	94.56
-27	12	6	19.41	84.39
-17	-3	7	24.96	81.61
10	-34	2	31.43	63.39
-18	-11	8	-25.62	55.60
8	-45	2	7.13	61.01
-15	20	-5	61.67	42.92
-1	5	-6	41.60	40.15
-19	19	1	215.52	75.27
-33	30	0	42.00	84.52
-37	34	-2	-20.07	69.07
10	-17	-9	37.51	74.48
8	-15	-10	15.58	68.54
-26	18	4	-54.41	72.11
8	-17	1	-4.36	52.96
15	-27	-3	21.92	68.80
-29	17	5	21.39	91.78
16	-30	-5	-7.66	89.67
-31	10	7	12.15	73.95
12	-37	1	-33.81	67.75
10	-37	2	45.69	62.20
4	-39	4	-11.75	50.71
-14	16	0	120.84	64.31
9	-10	-5	10.30	78.44
4	-5	-9	-42.13	84.92
-33	31	-1	92.84	79.63
-35	32	-1	0.00	75.80
-35	31	0	58.24	81.48
13	-19	-4	227.41	74.09

11	−17	−8	42.00	93.76
13	−20	−3	149.89	69.60
−21	14	4	240.09	71.71
15	−25	−5	35.13	75.01
16	−32	−3	−54.67	92.05
−11	−5	6	132.85	53.88
7	−26	3	83.33	50.18
4	−24	4	55.86	39.88
12	−34	1	−68.41	82.93
−23	−4	8	−7.40	65.24
−29	2	8	−47.41	72.77
−8	−24	7	3.83	18.36
11	−44	1	19.81	67.75
−3	7	−7	250.26	48.33
−14	18	−2	84.52	63.26
−26	28	−3	0.00	77.52
5	−5	−8	30.11	63.79
5	−6	−1	888.51	90.07
9	−11	−7	136.82	65.90
10	−13	−7	65.11	66.43
−11	7	3	1575.62	147.64
−17	13	3	181.98	79.76
−30	25	2	14.26	90.99
−27	21	3	−43.05	74.48
14	−22	−6	55.99	76.73
11	−19	−9	16.11	71.71
9	−17	−10	−51.50	75.67
−8	−4	5	174.45	53.48
−31	18	5	58.64	84.12
11	−26	1	167.45	63.26
13	−29	0	14.92	62.73
−29	13	6	−5.68	83.07
−26	6	7	1.58	66.29
10	−31	2	−2.51	60.22
15	−38	−1	6.47	69.99
5	−32	4	6.60	51.90
−26	−1	8	−8.19	66.95
−11	13	0	112.38	52.69
−24	26	−2	−14.13	62.07
8	−8	−5	17.43	73.29
−24	24	0	90.33	73.29
8	−9	−7	75.67	66.16
12	−16	−5	155.83	69.86
11	−15	−7	46.35	69.60
12	−19	−8	12.28	86.90
11	−18	−1	475.02	87.56
−12	5	4	138.53	69.33
−28	19	4	16.51	82.41
−24	13	5	61.28	71.97
16	−29	−6	0.00	83.99
17	−36	−4	−16.24	80.16
16	−36	−2	−27.47	79.76
15	−35	−1	52.30	88.08
16	−39	−2	−31.69	70.92

12	−40	1	−11.62	68.54
5	−35	4	−101.42	52.82
7	−41	3	−2.77	54.81
−20	24	−5	27.34	56.13
1	2	−7	0.26	52.82
−1	4	−2	128.10	17.83
−14	17	−1	737.16	93.50
4	−3	−2	179.07	46.49
−7	7	1	1008.55	98.78
−18	16	2	206.02	87.16
−23	20	2	35.00	71.97
−14	10	3	741.92	97.99
−32	26	2	−6.07	96.67
−18	11	4	173.13	66.43
−34	27	2	21.39	83.59
13	−21	−2	−10.43	72.63
10	−19	−10	−23.11	70.52
15	−28	−2	−5.02	71.84
−33	19	5	16.90	74.22
−15	0	6	68.67	58.90
−24	9	6	25.22	87.82
−31	14	6	−29.05	72.63
12	−31	1	−0.13	62.86
−20	0	7	10.17	79.76
−23	3	7	68.14	77.39
−16	−5	7	55.33	78.97
15	−41	−1	24.43	75.27
8	−34	3	−5.28	56.52
−22	−6	8	62.60	65.63
10	−40	2	65.63	58.90
9	−44	2	28.92	59.69
−3	−33	6	−14.79	36.45
−13	18	−4	−1.98	44.50
−24	25	−1	18.49	64.18
7	−7	−7	745.22	95.22
3	−3	−9	214.07	83.73
12	−17	−7	−11.23	76.46
−22	17	3	96.93	73.95
14	−21	−4	501.70	94.69
−29	22	3	−9.24	81.09
14	−22	−3	38.03	73.16
12	−21	−9	38.30	76.33
16	−28	−4	36.32	78.84
14	−27	−1	33.41	69.99
−28	7	7	13.47	67.35
14	−37	0	0.00	72.50
5	−29	4	21.53	50.58
−9	−19	7	−6.21	35.00
−16	−18	8	23.11	47.15
−23	26	−4	62.99	66.29
5	−3	−4	446.10	66.82
7	−6	−5	163.10	75.54
−28	29	−3	−66.43	87.95
−16	16	1	385.62	84.65

13	−18	−5	0.00	69.60
−15	8	4	154.78	84.78
13	−21	−8	24.30	80.03
−8	0	4	5.02	57.05
15	−24	−6	14.00	90.99
−30	20	4	0.26	94.42
16	−27	−5	14.92	91.25
−12	1	5	−13.73	56.65
16	−29	−3	67.88	80.03
8	−21	2	56.26	54.94
17	−32	−5	−17.70	84.78
17	−33	−4	101.29	82.67
15	−32	−1	19.15	80.43
14	−34	0	17.43	84.65
8	−31	3	−2.64	54.81
14	−40	0	34.20	70.26
−25	−3	8	−5.41	66.56
−28	0	8	48.07	72.11
8	−37	3	−13.34	56.39
1	−37	5	−14.13	47.41
−19	22	−2	29.58	60.48
4	−2	−3	647.23	69.07
4	−3	−8	33.28	61.01
−21	20	1	36.71	65.77
−9	7	2	767.54	83.07
13	−19	−7	−12.15	86.63
12	−20	−1	195.45	82.80
−23	15	4	122.82	70.52
−31	23	3	−5.28	93.76
−32	21	4	24.04	96.67
−26	14	5	0.13	79.50
−18	3	6	7.79	60.22
−21	6	6	−38.56	69.33
−10	−7	6	208.00	53.35
−21	−8	8	−36.71	63.92
12	−43	1	23.77	66.82
0	4	−4	1199.11	106.31
−8	12	−2	82.14	38.17
−11	15	−2	94.16	54.15
3	0	−6	792.50	88.88
−26	27	−2	12.02	67.09
−26	25	0	−34.47	69.46
−15	13	2	340.19	80.16
−7	2	3	220.67	50.71
14	−20	−5	75.14	69.20
−33	24	3	81.88	86.37
−34	22	4	0.00	69.33
17	−31	−6	28.92	73.29
−26	10	6	23.37	89.54
17	−34	−3	−47.94	86.50
10	−28	2	104.99	55.47
−15	−7	7	−12.15	75.41
−30	8	7	5.02	68.80
−30	1	8	44.50	70.78

−1	4	−8	15.32	50.18
−19	21	−1	−13.73	61.67
6	−5	−7	78.58	64.05
−26	26	−1	23.24	68.80
−30	30	−3	75.94	84.92
−25	21	2	−6.60	76.73
15	−23	−4	−2.25	73.03
15	−24	−3	28.00	69.99
14	−23	−8	−4.09	77.52
14	−23	−2	139.85	68.94
−2	−9	4	588.86	59.03
16	−28	−7	−49.65	73.43
6	−21	3	48.47	44.24
−19	−2	7	34.34	78.05
−25	4	7	13.73	70.78
14	−43	0	144.08	69.99
−20	−10	8	−32.75	58.77
5	−38	4	63.79	52.03
−17	−16	8	−6.34	51.11
−21	24	−3	84.78	61.67
−10	10	1	152.66	51.90
−13	13	1	376.77	68.54
−3	2	1	562.98	56.65
−12	10	2	470.80	71.58
−24	18	3	0.00	71.58
14	−21	−7	127.57	92.71
16	−26	−6	−59.96	96.67
−15	4	5	99.31	62.73
17	−30	−4	70.26	92.05
−7	−6	5	118.59	45.69
−28	15	5	41.47	91.91
14	−31	0	−69.86	67.75
−22	1	7	−1.06	72.63
−24	−5	8	55.47	64.31
−27	−2	8	50.98	71.05
−19	−12	8	−39.49	57.45
8	−40	3	11.49	54.81
−18	−14	8	2.38	47.15
10	−43	2	29.19	59.56
−11	16	−6	36.18	27.73
−16	20	−3	55.60	54.81
−18	22	−4	21.53	54.01
2	−1	−9	6.60	66.95
−28	28	−2	120.70	80.69
10	−11	−6	56.79	64.71
−32	31	−3	3.70	76.20
11	−13	−6	149.49	71.84
−28	26	0	1.72	69.99
−34	32	−3	−29.98	70.39
12	−15	−6	23.77	72.50
−36	33	−3	115.03	68.14
8	−13	0	570.77	71.31
−19	14	3	217.11	73.43
−34	28	1	−19.81	85.44

15	−22	−5	33.02	72.90
−25	16	4	95.22	74.22
17	−29	−5	27.07	97.59
16	−30	−2	22.05	73.82
12	−28	1	19.81	59.96
−28	11	6	46.49	81.48
−9	−9	6	44.50	46.35
17	−38	−2	−36.58	77.12
13	−36	1	−20.21	72.63
11	−36	2	70.52	61.28
13	−39	1	33.41	64.84
7	−44	3	−27.86	57.45
1	3	−5	348.11	59.16
6	−4	−5	39.88	68.14
−20	17	2	98.39	75.54
7	−11	0	814.02	82.41
−27	22	2	−81.61	70.12
9	−15	0	218.43	57.71
−20	12	4	83.20	72.24
13	−22	−1	20.21	74.22
15	−25	−8	0.00	71.45
17	−31	−3	−11.09	89.27
−30	16	5	6.74	87.29
2	−17	4	−3.30	24.96
18	−34	−5	−4.89	82.27
−14	−2	6	57.71	55.33
18	−35	−4	−59.03	75.54
16	−37	−1	0.00	76.86
−5	−17	6	9.11	29.85
−27	5	7	−15.58	67.22
−14	−9	7	24.70	68.01
17	−41	−2	−59.96	78.05
16	−40	−1	12.02	68.01
2	−30	5	18.75	42.26
2	−33	5	−31.69	43.18
−10	15	−4	72.63	44.11
−12	17	−5	96.01	39.49
−25	27	−4	111.20	80.95
9	−9	−6	149.49	67.48
−28	27	−1	1.45	74.88
−23	21	1	100.23	71.05
−30	27	0	−8.98	89.80
13	−17	−6	25.49	68.14
15	−23	−7	3.57	93.24
16	−25	−4	15.98	72.63
9	−23	2	143.81	54.41
−32	17	5	−6.34	80.29
−23	7	6	7.40	80.82
17	−35	−2	42.92	84.92
−30	12	6	−32.75	74.88
13	−33	1	66.29	83.07
8	−28	3	55.07	51.24
0	−20	5	189.51	18.49
11	−33	2	27.34	63.13

−10	−17	7	−42.66	42.26
−29	−1	8	−8.85	67.35
12	−46	1	−3.43	66.03
9	−47	2	29.32	62.99
−22	25	−5	0.00	59.96
5	−3	−7	97.20	60.35
3	−1	−8	−10.04	59.03
−16	17	0	15.05	70.92
4	−4	−1	3808.24	316.68
−21	21	0	161.11	60.62
−30	29	−2	78.18	90.86
9	−12	−9	−11.62	85.58
10	−14	−9	73.82	79.90
−32	28	0	6.74	92.71
10	−17	0	896.43	100.37
−26	19	3	165.60	76.86
16	−26	−3	94.56	71.18
15	−25	−2	44.90	68.14
−27	17	4	−28.26	73.56
17	−28	−6	54.54	85.31
16	−34	−1	−18.22	89.93
−32	13	6	−37.77	75.01
18	−39	−3	−36.85	63.92
15	−39	0	8.19	66.95
16	−43	−1	37.11	68.14
−23	−7	8	−39.75	65.77
−7	12	−4	90.73	31.96
−4	5	0	862.89	81.22
8	−10	−9	−19.02	88.88
−30	28	−1	18.88	89.67
−32	30	−2	22.98	82.67
−34	31	−2	5.55	75.01
8	−12	−10	−15.19	69.86
−36	32	−2	68.67	71.97
11	−16	−9	34.86	74.61
9	−14	−10	62.20	72.50
−34	29	0	−78.97	82.67
−29	23	2	8.85	77.78
−36	30	0	15.72	74.75
16	−24	−5	5.02	74.88
−20	4	6	31.83	65.24
−8	−11	6	33.41	42.92
15	−36	0	2.51	82.14
−18	−4	7	−4.62	73.16
−29	6	7	0.00	69.73
−26	−4	8	21.79	66.43
−21	23	−2	36.45	64.45
8	−7	−6	58.50	61.94
−18	17	1	327.25	87.29
11	−14	−8	151.08	90.99
6	−9	0	509.10	62.33
−5	2	2	389.84	53.22
−32	29	−1	78.97	93.63
−16	11	3	211.17	89.01

−36	31	−1	7.79	71.05
12	−18	−9	0.00	75.01
10	−16	−10	36.18	69.99
−2	−5	3	370.30	42.92
−31	24	2	77.52	89.80
−11	3	4	51.77	64.84
−17	9	4	81.09	66.43
−11	−1	5	8.98	55.73
18	−31	−5	51.24	82.54
18	−32	−4	−17.83	88.88
5	−26	4	7.79	44.37
−24	2	7	23.77	71.58
−31	7	7	3.17	71.05
−11	−15	7	−6.87	45.03
15	−42	0	−3.57	67.09
−5	10	−6	29.19	24.04
−23	25	−3	−54.81	61.41
−21	22	−1	37.64	61.41
−27	28	−4	10.70	86.90
10	−12	−8	164.15	82.93
12	−16	−8	28.79	89.14
−34	30	−1	−11.09	82.41
−10	5	3	725.41	83.86
11	−18	−10	102.08	72.77
11	−19	0	251.05	76.07
−28	20	3	−24.04	73.95
−33	25	2	−57.31	98.91
−35	26	2	49.26	84.25
14	−24	−1	234.01	75.54
−29	18	4	−13.47	87.82
11	−23	1	236.13	65.77
17	−32	−2	0.00	87.95
−25	8	6	−36.32	91.39
−21	−1	7	119.78	77.78
9	−36	3	0.00	57.97
6	−34	4	−38.96	50.98
11	−42	2	−61.94	60.48
−22	−9	8	−14.39	61.94
2	−36	5	−28.66	46.88
8	−43	3	0.00	54.01
10	−46	2	−77.52	62.46
5	−41	4	−14.79	52.69
4	−1	−4	412.82	61.80
−16	19	−2	174.06	70.39
9	−10	−8	0.00	72.77
7	−8	−9	51.24	88.61
11	−13	−3	48.07	82.80
12	−15	−3	65.11	82.80
−25	22	1	0.00	69.46
−22	18	2	−26.81	70.92
13	−18	−8	−53.22	91.12
−13	8	3	356.04	77.26
−21	15	3	43.18	73.43
13	−20	−9	2.11	73.95

−14	6	4	71.84	78.58
−22	13	4	228.86	79.50
17	−27	−4	0.00	76.07
−31	19	4	−56.79	97.20
14	−28	0	142.63	68.54
7	−23	3	111.72	48.73
15	−33	0	0.00	85.18
11	−30	2	23.37	61.54
−7	−13	6	−15.32	40.54
−6	−15	6	17.70	38.56
9	−33	3	3.17	58.77
−28	−3	8	−5.41	66.69
−16	18	−1	55.20	71.84
10	−11	−3	602.99	89.01
12	−14	−4	56.79	83.46
−17	14	2	769.65	110.54
13	−17	−3	147.78	89.67
17	−26	−5	41.34	86.37
−30	21	3	24.04	86.76
−32	22	3	0.00	92.97
17	−28	−3	51.11	70.65
16	−27	−2	58.11	76.60
18	−30	−6	−1.72	84.65
−33	20	4	−71.58	81.48
−6	−8	5	35.13	41.47
18	−33	−3	0.00	92.84
16	−31	−1	30.64	71.71
13	−30	1	42.26	62.20
−13	−4	6	−1.06	55.20
−26	3	7	22.19	69.07
9	−39	3	12.81	56.26
−25	−6	8	0.00	64.97
13	−45	1	−15.72	66.43
−21	−11	8	−30.24	59.69
0	4	−7	152.40	52.16
8	−8	−8	17.30	69.20
−29	29	−4	58.50	81.48
11	−12	−4	99.97	86.37
−23	22	0	91.12	73.95
13	−16	−4	169.96	73.16
14	−20	−8	−13.47	81.75
16	−23	−6	28.26	88.35
14	−22	−9	−3.17	75.01
−34	23	3	150.95	84.12
−27	9	6	23.51	81.75
18	−40	−2	0.00	73.29
17	−39	−1	−26.28	69.46
−17	−6	7	3.57	72.37
6	−37	4	56.92	53.22
−30	−2	8	0.00	70.52
−18	−17	8	57.97	52.43
−17	21	−5	183.04	51.90
5	−2	−5	523.36	81.48
7	−5	−6	18.49	61.01

−13	14	0	198.49	64.45
6	−6	−9	0.40	87.82
11	−14	−2	609.07	89.54
14	−18	−4	223.58	82.41
12	−16	−2	378.75	88.88
−27	23	1	−46.75	77.78
14	−19	−3	148.17	78.44
12	−21	0	198.09	76.07
−14	2	5	21.39	58.64
−17	5	5	73.69	66.82
10	−25	2	−13.34	57.84
−22	5	6	0.00	75.41
18	−37	−2	−20.07	80.56
14	−38	1	−61.28	67.75
17	−42	−1	15.32	68.80
−20	−13	8	−40.15	57.97
−19	−15	8	−19.94	55.73
2	2	−6	40.54	50.32
−23	24	−2	64.97	64.45
−25	26	−3	−42.79	66.56
10	−10	−4	2077.06	194.53
12	−14	−7	8.58	66.95
10	−12	−2	364.22	72.11
13	−16	−7	0.00	74.75
15	−22	−8	2.77	82.41
15	−26	−1	23.37	71.31
19	−33	−5	−6.60	80.43
19	−34	−4	−4.49	78.58
19	−38	−3	0.00	72.90
17	−36	−1	−27.07	81.88
−29	10	6	52.03	79.37
−23	0	7	4.89	70.12
−28	4	7	23.51	74.48
14	−41	1	79.50	70.78
−24	−8	8	−30.90	64.58
−27	−5	8	−44.11	68.41
11	−45	2	15.98	59.56
−13	17	−3	78.97	62.86
4	−1	−7	77.26	57.97
−20	23	−4	16.90	56.52
3	−1	−2	498.53	58.50
9	−9	−3	507.38	75.94
−23	23	−1	137.08	69.46
11	−12	−7	32.75	67.75
−15	14	1	148.30	71.45
14	−18	−7	17.30	83.73
15	−20	−4	60.48	77.39
13	−18	−2	214.20	81.88
−24	19	2	22.58	76.20
−29	24	1	68.54	82.54
15	−21	−3	38.96	69.73
−23	16	3	98.25	78.05
17	−25	−6	49.52	98.25
18	−28	−5	0.53	93.24

-24	14	4	219.49	74.88
18	-29	-4	76.86	89.01
12	-25	1	43.71	60.35
-31	11	6	5.94	70.52
14	-35	1	5.94	80.03
12	-35	2	80.95	67.09
-20	-3	7	12.41	73.69
-30	5	7	-18.22	74.88
12	-38	2	-23.37	61.01
2	1	-8	23.11	56.92
-18	21	-3	198.88	61.14
7	-6	-8	-40.94	65.50
10	-10	-7	172.47	67.48
-20	18	1	82.14	78.71
-25	23	0	-25.49	67.88
15	-20	-7	0.00	92.84
16	-24	-8	-23.64	74.22
-19	10	4	262.01	78.05
17	-29	-2	18.09	69.73
3	-19	4	82.01	33.41
-16	-1	6	-34.20	60.35
-19	2	6	-39.49	68.28
9	-30	3	3.30	58.11
16	-38	0	9.64	75.27
16	-41	0	0.00	68.28
9	-42	3	-2.77	57.58
-29	-4	8	-44.77	68.80
-3	8	-5	116.08	31.96
9	-10	-2	38.69	53.75
14	-17	-5	201.79	76.99
-14	11	2	760.28	96.67
14	-20	-2	80.82	81.48
-18	12	3	0.00	90.46
13	-23	0	96.40	69.07
18	-30	-3	53.62	83.86
15	-30	0	76.07	63.65
19	-35	-3	89.14	86.24
-16	-8	7	-53.35	67.62
12	-41	2	-11.09	59.96
14	-44	1	25.49	68.80
-23	-10	8	33.41	64.71
6	-40	4	-16.77	51.77
5	-4	-9	0.00	83.46
-10	11	0	311.93	55.60
11	-11	-5	808.35	100.89
-27	27	-3	62.33	79.24
5	-7	0	-17.70	47.28
15	-19	-5	95.48	75.14
16	-22	-4	117.40	73.29
-6	0	3	78.31	43.32
-26	20	2	59.56	71.71
16	-23	-3	33.68	68.67
-26	15	4	58.77	72.11
-10	-3	5	65.37	54.81

17	−33	−1	129.42	90.33
−24	6	6	36.45	88.48
16	−35	0	−87.03	87.95
−25	1	7	−42.13	70.12
16	−44	0	5.55	69.86
−26	−7	8	45.69	66.56
−15	19	−4	69.86	50.84
−16	20	−6	46.75	45.16
−7	8	0	582.65	67.62
−25	25	−2	28.53	67.09
−25	24	−1	5.15	68.94
−8	5	2	868.83	89.41
−27	24	0	−44.11	75.14
−37	32	−3	−39.75	72.50
16	−22	−7	−39.22	93.37
−25	17	3	65.77	76.99
18	−27	−6	33.02	91.12
16	−28	−1	26.81	74.61
−1	−11	4	92.57	21.66
8	−25	3	95.48	52.69
−12	−6	6	61.54	53.62
−9	−22	7	−9.51	31.69
−2	6	−8	30.11	46.62
−13	15	−1	308.49	67.75
9	−8	−7	75.94	65.50
8	−7	−3	231.90	57.84
−29	28	−3	0.00	88.88
−11	8	2	549.51	74.22
−35	31	−3	9.38	71.58
16	−21	−5	−16.24	70.92
15	−22	−2	26.15	70.78
−28	21	2	84.78	72.11
19	−30	−5	64.71	97.46
19	−31	−4	−2.11	90.59
−28	16	4	92.31	77.26
−19	6	5	58.50	69.20
−5	−10	5	9.77	37.37
20	−36	−4	0.00	73.29
14	−32	1	11.09	76.20
19	−39	−2	−60.62	75.94
12	−32	2	39.49	62.99
−2	−32	6	26.94	26.15
−22	−12	8	26.54	57.58
6	−3	−6	181.98	61.80
−18	20	−2	−11.75	63.65
10	−9	−5	6.87	72.77
−18	19	−1	77.52	78.97
−12	11	1	112.25	57.71
−31	29	−3	−2.38	82.80
−22	19	1	52.30	68.41
−33	30	−3	103.93	76.73
−29	25	0	31.03	75.80
−31	26	0	−1.98	90.20
17	−24	−7	−20.87	97.86

17	−24	−4	106.84	73.56
−35	28	0	8.45	89.54
−10	1	4	19.28	66.29
−16	7	4	192.94	73.82
−27	18	3	−67.22	73.82
−30	17	4	−6.60	104.20
−32	18	4	77.78	92.05
−34	19	4	−13.21	81.09
11	−27	2	−1.85	57.97
−26	7	6	91.78	89.67
6	−28	4	1.72	47.54
18	−41	−1	−16.77	69.99
−19	−5	7	−14.66	71.84
−22	−2	7	−22.71	71.31
−27	2	7	5.15	68.54
12	−44	2	−51.64	59.82
−28	−6	8	−9.38	65.63
−21	−14	8	−5.41	57.18
−13	16	−2	173.00	64.05
6	−4	−8	−39.22	64.71
−22	24	−4	16.90	61.80
8	−8	−2	566.67	70.12
−27	26	−2	48.73	71.97
−27	25	−1	1.06	67.62
11	−15	−1	230.45	63.26
12	−17	−1	455.08	85.97
17	−23	−5	−22.98	73.16
−33	27	0	0.13	96.14
−37	31	−2	0.00	71.18
−37	30	−1	16.64	73.03
17	−25	−3	14.66	80.29
−30	22	2	−10.17	79.37
−32	23	2	−1.58	99.44
19	−29	−6	58.90	83.73
−21	11	4	170.89	80.43
−34	24	2	−41.34	91.25
19	−32	−3	59.03	96.40
18	−31	−2	−9.51	79.37
13	−27	1	19.02	62.33
−21	3	6	−33.94	75.01
18	−38	−1	−46.49	73.29
10	−38	3	−5.41	57.84
−2	−29	6	−1.98	17.30
−25	−9	8	−0.13	63.39
−30	−5	8	−38.43	69.07
−20	−16	8	−17.83	57.31
−6	5	1	879.66	91.25
14	−16	−6	142.76	76.46
−29	27	−2	62.20	84.52
12	−15	−9	48.73	78.71
10	−13	−10	43.84	73.29
10	−13	−1	113.44	63.52
13	−17	−9	−12.28	76.60
11	−15	−10	79.10	73.56

12	−17	−10	0.00	74.35
−35	30	−2	−17.96	76.86
13	−19	−10	59.56	77.65
13	−19	−1	147.91	83.99
−15	9	3	126.25	75.41
−35	29	−1	−12.55	81.35
16	−24	−2	−38.96	75.54
−13	4	4	188.58	78.44
−29	19	3	60.62	80.82
−35	22	3	−13.87	85.84
19	−36	−2	88.74	82.54
−28	8	6	0.00	83.33
15	−40	1	5.55	66.95
−29	3	7	0.00	73.95
−31	4	7	−23.90	72.11
−2	7	−6	24.17	33.02
−1	5	−3	230.18	26.41
8	−6	−7	175.77	66.43
13	−14	−6	−15.05	74.48
11	−13	−9	10.70	82.54
15	−18	−6	61.14	73.16
−29	26	−1	−15.58	83.20
−31	28	−2	86.24	96.01
−31	27	−1	−59.30	94.16
−33	29	−2	67.09	83.07
14	−19	−9	123.08	78.97
−33	28	−1	91.91	94.29
−20	13	3	319.06	77.26
18	−26	−4	0.00	75.41
−31	20	3	8.98	95.88
−33	21	3	53.48	94.16
−13	0	5	63.79	58.50
−16	3	5	−2.25	65.24
16	−32	0	−34.73	78.44
−15	−3	6	5.02	59.82
−18	0	6	110.01	67.22
−30	9	6	12.15	71.97
15	−37	1	−9.64	70.78
−32	10	6	−3.30	70.78
7	−36	4	1.72	51.64
3	−32	5	−12.68	39.75
3	−35	5	−22.32	41.34
9	−45	3	−17.70	58.37
4	−2	−9	108.03	81.75
−20	22	−3	241.41	68.14
12	−12	−6	35.66	69.46
10	−11	−9	48.20	87.16
−9	8	1	97.20	49.92
−17	15	1	196.64	83.07
−24	20	1	−36.71	68.94
−21	16	2	117.27	71.31
15	−21	−9	8.72	73.69
18	−25	−5	0.00	84.25
14	−21	−1	448.74	92.71

20	−32	−5	0.00	80.56
20	−33	−4	47.67	93.50
18	−35	−1	149.36	92.57
17	−40	0	−10.96	68.54
−24	−1	7	−32.49	70.92
15	−43	1	63.26	71.58
10	−41	3	43.58	56.39
−24	−11	8	5.55	64.58
−27	−8	8	−32.88	66.43
−19	22	−5	68.67	55.20
9	−7	−5	69.20	77.39
13	−15	−8	−6.07	91.12
14	−17	−8	67.62	95.35
15	−19	−8	−42.13	85.05
−9	3	3	135.63	52.03
−12	6	3	342.70	72.24
18	−27	−3	54.28	74.61
−21	7	5	311.40	75.54
−11	−8	6	25.88	50.45
−23	4	6	62.20	90.73
17	−37	0	−20.07	75.94
13	−37	2	−56.26	65.63
17	−43	0	−22.45	72.90
7	−33	4	105.38	54.28
−14	−12	7	40.94	59.03
13	−40	2	−30.64	61.41
−29	−7	8	62.20	68.14
4	0	−5	965.10	103.67
3	1	−7	69.46	57.58
−15	15	0	105.91	71.58
12	−13	−8	93.37	92.57
9	−11	−1	589.52	74.61
17	−22	−6	58.11	82.80
17	−26	−2	103.93	79.76
−6	−4	4	226.88	56.79
20	−31	−6	−16.64	76.99
−23	12	4	102.35	72.50
15	−27	0	2.64	68.54
10	−32	3	−43.32	62.20
−18	−7	7	1.32	67.75
−21	−4	7	0.00	70.12
7	−39	4	−15.05	51.90
12	−47	2	0.00	60.48
−23	−13	8	0.00	60.75
−10	14	−3	313.91	55.86
11	−10	−6	59.69	69.33
−24	25	−4	22.05	66.69
9	−9	−9	53.35	88.74
−16	12	2	325.79	82.54
16	−21	−8	7.13	80.82
−26	21	1	−14.66	78.84
19	−27	−5	0.00	94.82
15	−23	−1	129.02	69.73
19	−28	−4	13.34	89.93

20	−34	−3	−15.05	97.99
14	−29	1	10.04	62.07
9	−27	3	26.02	51.11
15	−34	1	36.18	87.29
20	−41	−2	27.34	72.77
−26	0	7	19.15	69.99
13	−43	2	−76.99	63.39
−26	−10	8	42.13	66.82
−22	−15	8	95.88	64.97
3	1	−4	471.06	60.75
7	−5	−3	405.96	63.92
−20	21	−2	37.24	64.31
11	−11	−8	70.78	84.39
−20	20	−1	153.98	70.12
18	−24	−6	65.24	92.84
17	−23	−8	−28.13	75.67
−22	14	3	−1.72	67.22
8	−18	2	82.80	57.84
12	−29	2	8.85	60.75
13	−34	2	0.00	57.45
19	−43	−1	84.92	73.03
−13	−14	7	0.00	52.56
15	−46	1	0.00	68.01
5	−2	−8	21.26	61.01
−23	17	2	0.00	70.65
−28	22	1	29.45	71.31
9	−16	1	240.22	59.82
−30	23	1	41.86	80.69
10	−18	1	5.02	61.54
−32	24	1	53.62	96.01
−34	25	1	109.08	98.52
19	−29	−3	−34.07	78.31
−25	13	4	4.49	70.65
21	−35	−4	−1.06	78.05
−9	−5	5	38.17	50.32
−23	8	5	3.83	67.22
−4	−12	5	131.14	25.09
20	−38	−2	40.54	75.14
−25	5	6	−17.30	93.24
19	−40	−1	124.14	73.43
−28	1	7	0.26	74.88
−12	−16	7	−12.94	50.84
−30	2	7	4.23	74.22
−11	−18	7	−0.40	45.03
10	−44	3	0.00	57.45
3	−38	5	15.19	48.99
−28	−9	8	11.62	68.80
1	3	−8	36.85	53.48
7	−4	−7	283.93	69.60
−26	26	−4	48.07	82.80
18	−28	−2	141.17	78.31
9	−20	2	71.05	65.37
−27	14	4	−12.81	78.05
18	−32	−1	31.17	83.99

−18	4	5	200.07	72.77
17	−34	0	22.32	88.22
−20	1	6	−5.94	76.07
1	−22	5	−50.05	17.30
−27	6	6	−44.24	82.27
−25	−12	8	31.56	67.48
5	−1	−6	174.19	61.14
−15	18	−3	90.86	61.14
10	−8	−6	78.97	71.58
10	−9	−8	−12.02	76.20
8	−7	−9	38.69	90.86
7	−6	−2	194.00	49.92
3	−2	−1	527.45	55.07
−22	23	−3	58.11	64.45
15	−17	−7	−11.09	83.07
16	−19	−7	0.00	94.16
8	−14	1	210.11	53.35
19	−26	−6	78.44	95.61
18	−25	−8	62.07	75.01
−17	10	3	177.62	87.03
20	−29	−5	47.41	103.14
16	−25	−1	−6.87	71.05
−24	15	3	50.32	76.99
20	−30	−4	18.09	103.14
−29	15	4	0.00	89.01
−33	17	4	−5.94	83.46
19	−37	−1	60.22	81.75
−29	7	6	31.43	74.61
−33	9	6	−2.38	69.20
−23	−3	7	101.82	71.97
−4	9	−7	23.11	35.92
−17	20	−4	61.94	57.31
14	−15	−7	17.83	78.58
8	−9	−1	121.36	49.13
−28	27	−4	37.90	91.65
−19	16	1	150.68	85.97
−32	29	−4	124.14	78.44
17	−21	−7	−10.43	96.01
−25	18	2	21.79	76.07
−35	23	2	155.17	91.25
16	−29	0	40.94	68.54
−31	16	4	−14.00	97.99
−25	9	5	5.55	76.60
−14	−5	6	83.20	59.56
−17	−2	6	4.09	64.58
−10	−10	6	79.24	51.11
16	−39	1	49.79	68.01
−31	8	6	47.67	74.61
3	−29	5	34.20	41.86
13	−46	2	−15.58	62.73
7	−42	4	128.36	58.50
−27	−11	8	−50.98	70.78
8	−5	−5	144.34	76.07
7	−4	−4	1301.33	127.57

−10	13	−2	192.41	48.99
−15	16	−1	179.21	72.77
13	−13	−7	90.86	73.56
−22	21	−1	5.81	66.56
15	−17	−4	43.58	74.88
−30	28	−4	−71.58	86.10
16	−19	−4	178.28	76.99
15	−18	−3	46.09	77.26
−38	31	−3	−63.39	76.60
−27	19	2	−61.67	74.48
−36	27	0	88.61	90.46
12	−22	1	106.18	69.33
−15	5	4	83.86	79.10
−26	16	3	179.60	77.39
21	−36	−3	0.00	84.39
20	−35	−2	26.54	85.18
5	−23	4	68.67	41.73
7	−30	4	36.32	55.33
18	−42	0	17.30	73.69
11	−37	3	−23.64	54.81
−20	−6	7	−59.82	69.46
11	−40	3	−58.50	60.88
0	5	−5	561.66	67.75
−14	18	−5	236.13	52.30
−21	23	−5	23.37	61.80
−22	22	−2	84.92	65.37
14	−15	−4	21.92	75.27
14	−16	−3	179.47	84.65
−14	12	1	650.00	89.27
17	−21	−4	155.70	82.93
16	−20	−3	275.08	74.88
−13	9	2	47.67	69.60
−26	22	0	0.53	72.90
18	−23	−7	12.28	96.80
20	−28	−6	11.09	86.76
−29	20	2	115.42	82.14
−31	21	2	52.69	86.63
20	−31	−3	−86.24	95.35
−20	9	4	141.44	75.94
−33	22	2	86.76	102.22
10	−22	2	74.09	56.65
−12	−2	5	142.89	59.56
−15	1	5	117.93	66.03
−34	20	3	28.92	94.56
−27	10	5	−22.98	94.03
−22	2	6	15.19	88.61
−33	13	5	1.19	80.56
18	−39	0	−52.30	73.43
−25	−2	7	5.55	71.58
16	−45	1	52.03	70.78
12	−11	−7	148.04	68.28
−24	24	−3	31.43	66.69
13	−14	−3	212.09	87.82
−18	13	2	173.53	88.08

−36	30	−3	−16.11	72.63
−34	26	0	42.92	101.16
−38	30	−2	61.14	72.50
21	−31	−5	47.01	89.27
17	−27	−1	26.15	71.97
19	−30	−2	19.28	81.09
−28	17	3	40.28	74.61
−30	18	3	−3.57	91.78
−32	19	3	9.64	101.29
13	−31	2	−17.30	63.13
−29	11	5	93.63	101.69
−31	12	5	−0.13	89.41
16	−36	1	0.00	78.44
−27	−1	7	−0.79	73.95
−9	−25	7	−40.15	23.24
1	4	−6	98.25	48.86
−1	6	−7	2.51	45.30
−7	9	−1	1123.71	102.74
−15	17	−2	70.26	67.35
13	−13	−4	100.37	88.22
−12	12	0	687.51	83.86
15	−16	−5	193.47	80.03
−17	16	0	34.47	76.60
16	−18	−5	−0.40	75.67
17	−20	−5	67.09	76.07
−4	0	2	3182.67	264.39
18	−23	−4	130.87	80.03
17	−22	−3	−2.38	74.48
−28	23	0	49.79	72.11
19	−25	−7	1.32	93.63
−30	24	0	82.54	78.05
−32	25	0	74.48	102.22
−36	28	−1	1.45	85.84
−12	2	4	83.99	71.58
21	−32	−4	3.04	91.91
19	−34	−1	−67.48	96.67
−20	5	5	121.63	69.60
21	−40	−2	0.00	72.11
10	−29	3	40.15	58.90
11	−34	3	32.88	62.60
−16	−11	7	−9.11	62.99
14	−42	2	24.70	61.14
−29	0	7	34.34	75.94
−12	16	−4	172.87	52.30
9	−6	−6	16.24	65.24
7	−5	−9	0.00	89.93
−26	25	−3	−23.64	70.26
−24	22	−1	83.33	73.69
15	−18	−9	−29.71	85.71
13	−16	−10	−7.53	75.54
18	−22	−5	0.13	73.56
14	−18	−10	−35.00	74.48
−21	17	1	230.84	81.75
11	−16	0	424.44	78.97

7	−12	1	611.71	67.62
−34	29	−3	35.13	78.18
12	−18	0	328.04	76.73
13	−20	0	71.58	84.25
−36	29	−2	−34.34	79.63
−19	11	3	37.11	75.67
21	−30	−6	62.07	84.12
13	−24	1	6.47	59.43
17	−31	0	52.56	72.90
18	−36	0	−25.88	83.07
20	−42	−1	34.34	78.18
14	−36	2	0.26	65.63
−22	−5	7	0.00	68.01
11	−43	3	−12.02	60.09
12	−12	−3	867.77	115.82
−24	23	−2	96.27	69.73
14	−16	−9	3.70	80.95
12	−14	−10	5.94	78.58
14	−17	−2	528.77	98.78
16	−20	−9	35.00	75.41
15	−19	−2	162.83	96.93
−32	28	−3	−93.37	86.90
19	−25	−4	−19.81	78.71
18	−24	−3	56.39	78.31
−14	7	3	1478.03	148.96
−34	27	−1	104.06	95.88
−22	10	4	234.14	76.86
11	−24	2	0.53	57.05
20	−39	−1	−40.28	75.54
−24	3	6	86.37	93.90
8	−35	4	−12.15	53.88
8	−38	4	43.71	56.13
11	−9	−7	92.44	71.18
12	−11	−4	86.90	87.69
−23	24	−5	39.09	67.09
13	−14	−9	86.76	84.78
4	−5	0	707.05	70.78
13	−15	−2	321.30	83.33
−28	26	−3	47.28	84.92
−26	23	−1	68.01	71.97
−30	27	−3	−1.98	91.39
19	−24	−5	−3.70	88.61
17	−22	−9	0.00	79.76
16	−21	−2	79.24	82.41
−32	26	−1	85.58	108.55
−34	28	−2	−11.09	83.86
5	−12	2	219.09	39.75
−5	−2	3	254.88	48.07
14	−22	0	97.33	75.54
22	−33	−5	−13.87	80.29
21	−33	−3	0.00	94.03
21	−37	−2	52.82	80.95
−19	−1	6	−12.41	74.09
−26	4	6	64.97	84.92

14	−45	2	−19.68	62.46
6	−2	−7	−8.98	64.84
4	0	−8	241.54	66.43
−4	6	−1	735.32	72.50
13	−12	−5	133.78	72.11
−26	24	−2	−13.87	68.41
10	−14	0	1125.42	112.78
−10	6	2	1170.19	115.16
−28	24	−1	51.24	74.09
−30	25	−1	−60.09	84.78
−32	27	−2	−5.55	98.25
−20	14	2	149.23	90.33
19	−26	−3	126.38	77.39
22	−34	−4	0.00	79.90
20	−32	−2	167.85	94.29
−22	6	5	−5.28	68.67
−13	−7	6	−1.58	58.50
−9	−12	6	−1.45	50.98
−28	5	6	2.91	79.76
−30	6	6	−42.52	74.09
−32	7	6	−5.94	76.07
−24	−4	7	50.84	66.69
12	−12	−9	0.00	81.75
16	−17	−6	−59.96	79.76
17	−19	−6	0.00	81.61
16	−18	−8	2.64	90.46
17	−20	−8	188.45	89.27
−28	25	−2	28.26	73.43
−30	26	−2	59.16	93.50
−23	18	1	60.62	69.46
20	−26	−5	−64.97	96.01
17	−23	−2	13.21	73.95
20	−27	−4	66.82	86.50
−17	6	4	148.44	72.24
−35	24	1	126.12	98.39
14	−26	1	149.63	69.20
−24	11	4	12.81	79.24
−8	−7	5	121.36	50.45
16	−33	1	132.19	90.07
−34	16	4	−11.75	87.56
6	−25	4	8.32	43.84
−16	−4	6	20.34	67.48
17	−41	1	5.15	73.29
−15	−13	7	48.20	60.35
4	−34	5	1.58	45.56
8	−41	4	86.90	57.58
8	−5	−8	−27.47	69.99
6	−3	−3	771.24	81.88
−19	21	−4	0.00	59.82
15	−15	−6	227.67	81.09
15	−16	−8	65.77	97.99
12	−13	−2	293.97	72.77
−19	17	0	91.65	88.48
18	−22	−8	5.02	82.93

-7	3	2	2056.32	178.55
15	-24	0	37.24	70.92
-21	12	3	-0.79	67.09
6	-18	3	40.15	49.92
7	-20	3	111.59	47.67
12	-26	2	87.03	62.60
-26	12	4	31.17	75.41
-17	2	5	3.83	67.62
20	-36	-1	95.88	85.58
-3	-14	5	60.88	31.69
-32	15	4	0.00	92.71
19	-41	0	2.51	73.69
19	-44	0	-28.39	78.97
17	-44	1	0.00	74.75
4	-37	5	16.64	48.73
11	-46	3	0.00	55.20
7	-3	-5	960.48	111.72
-7	11	-3	1451.88	126.12
-17	19	-3	80.69	59.56
11	-10	-3	433.56	85.44
14	-14	-8	283.01	105.91
-17	17	-1	319.46	85.18
-25	25	-5	158.34	85.44
-11	9	1	696.89	81.88
19	-23	-6	183.96	102.35
-25	19	1	-57.58	86.10
18	-25	-2	102.88	84.12
-11	4	3	1519.49	143.42
-33	23	1	11.89	102.35
-36	22	2	12.41	87.82
18	-33	0	-56.79	98.65
-28	13	4	-40.67	82.14
-30	14	4	0.00	102.22
-24	7	5	113.70	76.60
14	-33	2	0.00	66.82
17	-38	1	0.00	72.90
-34	12	5	73.95	81.48
-30	-1	7	-3.30	78.71
2	3	-7	96.80	56.65
12	-10	-5	254.88	79.10
14	-13	-6	41.60	76.99
11	-10	-9	-45.56	92.44
7	-7	-1	966.29	96.80
-16	13	1	326.72	78.18
20	-25	-6	-24.04	97.86
19	-24	-8	18.62	78.71
21	-28	-5	47.67	103.27
-22	15	2	73.95	79.10
-27	20	1	-67.35	82.67
21	-29	-4	59.30	99.57
20	-28	-3	-22.45	78.31
-29	21	1	61.67	76.99
-31	22	1	26.02	88.61
-5	-6	4	272.84	55.20

19	−31	−1	−0.13	69.99
22	−35	−3	29.58	83.86
−35	19	3	−87.16	93.50
19	−38	0	−34.20	77.52
11	−31	3	45.69	64.05
−21	0	6	29.45	89.01
−32	11	5	−17.30	80.95
12	−39	3	4.49	62.07
−18	−10	7	−4.75	66.16
14	−48	2	−8.58	67.88
10	−7	−7	276.27	73.82
6	−3	−9	134.70	89.80
−15	10	2	436.07	84.25
−8	1	3	172.60	52.56
−1	−7	3	122.42	26.28
−23	13	3	−22.85	81.88
5	−16	3	137.74	48.07
−37	26	0	−31.96	85.97
23	−36	−4	0.00	72.63
−34	21	2	138.93	102.74
8	−22	3	−6.47	48.99
22	−39	−2	83.86	81.09
−26	8	5	3.17	91.52
−28	9	5	40.54	97.86
−30	10	5	129.95	90.86
15	−41	2	−45.30	65.63
−14	−15	7	0.00	56.52
17	−47	1	42.39	73.82
12	−42	3	33.02	62.33
−10	−23	7	−8.85	43.45
8	−4	−6	10.83	62.73
6	−4	−2	126.38	47.28
13	−12	−8	167.32	96.27
−17	18	−2	110.93	77.26
9	−12	0	520.06	68.14
19	−27	−2	−7.79	77.12
−16	8	3	243.92	83.20
15	−28	1	76.07	67.62
−11	−4	5	77.39	55.47
−14	−1	5	78.97	63.52
−33	18	3	0.66	104.06
21	−41	−1	0.00	80.16
2	−24	5	6.34	32.88
15	−38	2	8.45	67.22
15	−44	2	−24.56	65.77
4	1	−6	170.89	55.73
3	2	−5	453.63	71.84
6	−2	−4	171.28	59.96
−7	10	−2	3791.60	312.85
11	−11	−2	370.56	71.05
−9	9	0	1134.54	107.89
21	−27	−6	44.24	91.52
22	−30	−5	−36.32	90.73
−24	16	2	5.41	72.50

21	−30	−3	0.00	102.74
−25	14	3	170.49	81.61
−19	7	4	243.26	76.20
−32	20	2	42.00	99.05
−31	17	3	−5.02	97.59
−8	−14	6	0.00	52.96
−23	1	6	2.64	90.33
−13	−17	7	−14.92	54.28
−13	17	−6	3.43	40.67
13	−11	−6	17.30	71.31
−21	22	−4	76.60	62.99
17	−18	−7	125.06	100.23
18	−20	−7	−26.28	100.10
14	−18	−1	758.56	109.61
6	−10	1	1373.57	121.50
15	−20	−1	234.80	92.97
16	−22	−1	125.06	75.14
−35	29	−4	−10.43	71.05
22	−31	−4	−4.62	94.69
−26	17	2	37.77	80.29
−28	18	2	30.24	76.86
−35	25	0	−4.36	98.39
−14	3	4	382.98	89.41
−30	19	2	130.34	84.39
−27	15	3	44.24	75.27
−29	16	3	−37.37	80.56
13	−28	2	−26.02	60.09
−19	3	5	159.66	75.54
17	−35	1	−7.79	89.41
−18	−3	6	52.56	75.01
−25	2	6	−30.37	85.84
−31	5	6	2.91	76.07
4	−31	5	−24.17	44.64
−11	−21	7	−11.89	47.01
12	−10	−8	75.41	86.50
10	−8	−9	9.38	94.82
16	−16	−7	54.28	90.99
−14	13	0	199.54	62.20
−19	18	−1	62.86	80.43
19	−22	−7	69.20	95.08
22	−29	−6	96.54	88.08
−37	29	−3	−52.16	77.52
20	−29	−2	36.85	77.39
−37	27	−1	29.85	80.69
17	−28	0	18.75	77.65
20	−33	−1	31.69	98.78
9	−24	3	−23.11	52.30
19	−35	0	−140.51	95.22
21	−38	−1	31.83	78.71
7	−27	4	40.15	50.45
−12	−9	6	24.70	60.22
−27	3	6	−40.81	81.75
−29	4	6	−0.53	80.29
−25	−5	7	15.45	79.50

−12	−19	7	13.87	50.98
−29	−3	7	2.38	79.50
12	−45	3	−1.85	62.07
−31	−2	7	−83.46	79.24
2	2	−3	803.06	75.94
11	−8	−5	138.93	76.20
−16	19	−5	185.68	56.92
10	−8	−3	118.85	65.63
−19	20	−3	−6.87	63.39
13	−16	−1	254.35	76.20
20	−24	−7	10.30	90.07
17	−24	−1	141.57	75.27
−33	24	0	−121.76	106.57
22	−36	−2	8.32	85.05
−35	15	4	−69.99	82.27
−15	−6	6	56.92	67.35
20	−43	0	−50.84	76.86
−17	−12	7	58.50	61.80
−20	−9	7	16.11	69.46
−27	−4	7	−73.03	78.44
15	−47	2	31.03	68.94
0	5	−8	0.40	51.90
7	−3	−8	171.94	72.50
−12	15	−3	80.03	61.54
15	−14	−7	−45.69	77.39
16	−19	−10	15.98	69.86
−18	14	1	690.94	102.08
21	−26	−7	60.22	83.73
−33	28	−4	−4.62	79.76
−31	23	0	−19.55	85.31
23	−32	−5	0.00	87.03
−37	28	−2	81.22	83.46
22	−32	−3	46.22	110.40
−21	8	4	55.07	74.61
16	−30	1	83.33	67.48
15	−35	2	94.95	72.50
18	−40	1	86.37	75.14
9	−37	4	0.00	56.26
9	−40	4	7.40	55.99
−19	19	−2	152.53	68.01
−23	23	−4	40.67	66.69
15	−17	−10	9.24	76.46
−8	6	1	1471.82	134.17
−17	11	2	180.53	94.95
−35	28	−3	−39.62	78.31
18	−26	−1	61.41	77.92
−35	26	−1	−114.76	100.23
23	−33	−4	90.33	85.31
−21	4	5	107.23	69.86
−33	14	4	73.95	89.54
20	−40	0	0.00	74.88
12	−33	3	46.49	63.65
18	−46	1	0.00	74.09
5	0	−7	126.25	62.07

9	−5	−7	16.51	68.94
12	−9	−6	155.70	79.24
−12	14	−2	112.65	58.90
17	−18	−4	78.71	76.99
16	−17	−9	−83.73	82.27
14	−15	−10	−92.97	81.75
18	−20	−4	39.49	80.29
17	−19	−9	−10.70	83.46
12	−14	−1	742.58	99.31
19	−22	−4	63.52	83.99
18	−21	−9	−12.02	79.37
−31	27	−4	90.59	86.24
−18	9	3	31.43	87.03
21	−31	−2	16.51	95.61
−8	−3	4	45.96	62.07
18	−30	0	15.19	74.61
−36	23	1	23.77	89.93
−23	9	4	104.86	77.26
14	−30	2	68.41	64.97
10	−26	3	96.14	55.99
22	−43	−1	−4.62	75.14
−20	−2	6	68.01	92.31
−7	−16	6	29.98	55.60
−33	10	5	49.39	86.76
−22	−8	7	0.92	72.24
14	−12	−7	77.52	74.35
18	−19	−5	49.92	77.12
−25	24	−4	−28.00	76.33
19	−21	−5	−5.28	76.46
−21	19	−1	12.28	70.39
−27	25	−4	95.48	91.91
−29	26	−4	−6.60	94.69
20	−24	−4	−4.49	80.03
−12	7	2	338.87	68.28
−33	25	−1	19.81	111.99
−35	27	−2	0.00	93.90
21	−35	−1	63.79	93.24
−7	−9	5	14.26	46.09
−16	0	5	267.69	75.67
−23	5	5	32.09	75.14
−31	13	4	−5.02	99.97
−31	9	5	16.11	90.73
−16	−14	7	29.05	62.46
11	−8	−8	−52.43	80.29
9	−6	−9	62.46	98.25
2	1	−2	421.14	50.18
10	−9	−2	58.90	62.99
17	−17	−5	231.64	84.92
16	−16	−4	221.47	80.56
15	−15	−9	0.00	86.10
−21	21	−3	256.46	74.75
17	−19	−3	40.54	71.84
8	−10	0	20.47	46.62
−2	0	1	1291.29	108.95

20	−23	−5	−168.64	89.80
18	−21	−3	141.57	79.24
19	−23	−3	98.39	80.69
−20	15	1	204.17	94.16
−33	27	−3	34.47	90.20
−13	5	3	484.27	84.65
19	−28	−1	26.54	74.88
23	−34	−3	−55.07	91.39
−34	22	1	−58.90	111.06
−25	10	4	−38.30	82.27
−27	11	4	117.01	86.24
−29	12	4	−8.58	97.59
22	−40	−1	12.94	78.84
18	−37	1	−34.20	78.97
−25	6	5	29.19	82.41
−29	8	5	−69.46	92.57
9	−34	4	1.06	57.45
16	−43	2	−39.09	69.46
13	−41	3	2.11	62.20
−19	−11	7	−34.86	69.73
−24	−7	7	0.00	74.35
9	−43	4	34.60	60.48
−30	−4	7	−42.92	79.63
3	2	−8	48.07	59.82
−14	17	−4	239.16	56.92
16	−17	−3	0.00	87.95
−13	10	1	131.93	59.56
−23	20	−1	79.24	76.99
21	−25	−5	31.43	99.71
21	−26	−4	27.20	85.84
−31	24	−1	−61.54	92.57
−33	26	−2	94.82	103.67
24	−35	−4	−61.01	87.95
−16	4	4	243.78	77.78
23	−38	−2	−33.02	84.78
17	−32	1	90.99	82.67
−35	20	2	32.75	97.20
20	−37	0	35.00	83.59
−34	17	3	−8.72	99.57
−27	7	5	82.54	102.88
−22	−1	6	−7.26	93.90
13	−38	3	59.43	66.43
−32	4	6	−4.49	80.69
−26	−6	7	18.22	76.86
−28	−5	7	−27.86	82.41
−1	−34	6	−19.55	33.02
14	−13	−9	−7.26	85.71
−6	6	0	2334.71	201.53
18	−19	−8	−3.57	89.01
−21	20	−2	45.43	67.48
19	−21	−8	−64.18	89.14
20	−23	−8	22.58	85.84
20	−25	−3	4.62	77.39
−31	26	−3	55.47	99.84

−29	23	−1	−65.11	84.12
−19	12	2	348.91	98.39
4	−14	3	659.25	67.09
−20	10	3	0.00	74.35
22	−33	−2	−10.30	101.42
−32	21	1	32.35	91.25
−17	−5	6	−40.67	78.84
−24	0	6	70.65	87.82
−30	3	6	0.00	78.05
16	−46	2	3.96	71.84
13	−44	3	29.58	63.52
7	−2	−6	56.13	62.86
−1	6	−4	572.22	57.31
10	−6	−5	131.27	79.10
15	−14	−4	14.26	79.63
17	−17	−8	99.57	97.59
15	−15	−3	135.76	90.20
−5	3	1	1463.63	136.15
−16	14	0	191.49	81.09
−25	21	−1	144.61	75.41
22	−27	−5	28.66	105.25
−27	22	−1	66.95	76.73
22	−28	−4	−70.39	103.40
−22	16	1	−1.19	78.44
−31	25	−2	64.71	99.84
19	−32	0	55.07	83.86
−13	−3	5	79.76	64.31
−32	16	3	−0.13	102.61
8	−29	4	24.56	57.05
−11	−11	6	0.00	60.48
21	−45	0	−26.68	77.78
−26	1	6	−14.66	82.93
−28	2	6	−47.28	82.14
5	−36	5	14.39	51.24
−10	−26	7	−9.77	44.37
6	−1	−5	340.98	75.67
13	−10	−7	46.49	72.24
18	−18	−6	59.16	84.92
11	−12	−1	85.44	58.50
−23	21	−2	10.56	73.82
−25	23	−3	14.66	69.46
21	−24	−6	94.69	106.57
−27	24	−3	86.24	76.33
−29	25	−3	−50.98	91.91
21	−27	−3	−18.49	81.75
−30	20	1	−83.46	86.90
−22	11	3	63.92	77.92
−33	19	2	128.23	106.31
15	−32	2	33.41	70.65
11	−28	3	31.03	57.05
−2	−16	5	18.09	26.94
21	−42	0	−61.54	80.03
16	−37	2	45.83	73.16
19	−42	1	−24.96	79.76

3	−26	5	24.96	43.84
19	−45	1	−2.64	77.52
−15	−16	7	−33.94	62.73
−21	−10	7	0.00	74.35
11	−7	−6	711.28	97.46
−18	20	−5	−19.15	57.84
16	−15	−8	73.29	99.57
17	−20	−2	35.66	80.03
−25	22	−2	−3.17	72.11
22	−26	−6	−27.73	104.33
18	−22	−2	−8.98	75.27
−27	23	−2	41.20	73.29
−29	24	−2	−18.49	82.01
23	−29	−5	5.94	98.65
−24	17	1	64.71	76.99
−21	13	2	−45.16	84.39
−26	18	1	0.00	77.39
−28	19	1	0.00	79.50
24	−36	−3	−30.24	86.10
−36	24	0	−30.51	97.06
22	−37	−1	−12.94	85.58
−30	15	3	5.41	89.01
−10	−6	5	5.94	56.79
−14	−8	6	66.16	70.26
−6	−18	6	23.11	50.58
−34	9	5	−5.28	85.05
5	−39	5	33.94	54.54
2	3	−4	494.57	60.75
9	−6	−3	7.53	59.82
13	−11	−9	129.55	86.90
17	−16	−6	−14.66	78.44
16	−18	−2	84.52	90.46
19	−24	−2	30.37	86.10
4	−10	2	1007.89	89.27
23	−30	−4	10.96	101.16
22	−29	−3	78.97	97.46
12	−19	1	183.04	72.50
14	−23	1	−8.32	66.03
−24	12	3	42.39	79.63
−38	26	−1	53.62	87.95
−18	5	4	83.46	70.92
−26	13	3	0.00	77.12
−31	18	2	−24.04	86.76
−28	14	3	42.13	85.05
24	−40	−2	18.88	81.61
−34	13	4	34.07	95.35
13	−35	3	26.41	65.11
−18	−13	7	0.00	65.37
−23	−9	7	−73.69	78.05
16	−49	2	−20.87	75.67
−29	−6	7	10.30	79.90
10	−6	−8	84.65	79.37
14	−12	−4	−26.94	93.50
14	−13	−3	202.58	88.61

6	−5	−1	920.07	95.48
23	−28	−6	−34.07	98.39
−23	14	2	208.39	76.73
−29	17	2	0.00	83.20
18	−34	1	92.44	95.48
21	−39	0	−13.21	84.12
23	−42	−1	−14.53	82.41
19	−39	1	−143.95	79.24
−19	−4	6	−3.43	92.97
−14	−18	7	−45.43	58.37
−25	−8	7	−101.03	81.35
−27	−7	7	−61.67	82.41
8	−3	−7	36.45	65.77
6	−1	−8	55.99	67.88
9	−5	−4	513.45	84.52
16	−14	−6	198.49	82.80
15	−13	−8	0.00	106.44
20	−26	−2	58.64	79.37
15	−21	0	56.65	85.58
24	−31	−5	−4.75	94.69
16	−23	0	247.35	76.99
−10	2	3	271.78	61.54
−15	6	3	166.13	83.46
15	−25	1	74.61	71.05
−25	15	2	96.93	83.20
−38	28	−3	0.00	80.95
21	−32	−1	0.00	92.84
−27	16	2	−55.47	83.46
−38	27	−2	22.58	84.39
20	−34	0	50.18	96.14
−37	22	1	25.62	94.82
−20	2	5	−54.01	75.54
−32	12	4	45.96	99.97
−32	8	5	−5.41	88.35
5	−33	5	39.88	48.73
10	−39	4	23.37	61.14
−11	−24	7	41.34	51.90
8	−4	−9	−23.64	92.84
20	−21	−7	−7.66	99.84
15	−16	−2	311.14	93.90
−11	10	0	294.89	57.84
21	−23	−7	−34.34	97.99
−18	15	0	133.78	84.65
14	−19	0	31.03	83.20
−9	4	2	1352.97	125.85
24	−30	−6	−58.37	83.59
11	−17	1	474.50	79.10
−14	8	2	114.76	69.46
24	−32	−4	187.26	97.06
23	−31	−3	−24.96	109.74
−36	28	−4	−13.21	80.82
−20	6	4	89.80	85.31
12	−30	3	16.24	62.73
−30	7	5	−7.00	96.93

17	-42	2	-7.26	70.92
-31	2	6	-47.28	84.78
10	-42	4	-17.04	62.73
-13	-20	7	-19.55	57.97
12	-8	-7	121.76	73.56
14	-11	-5	266.24	80.29
-14	16	-3	95.74	71.58
-9	10	-1	15.19	43.45
-20	21	-5	58.90	63.13
19	-19	-7	0.00	102.08
22	-25	-7	-71.71	88.48
-15	11	1	204.69	76.86
21	-28	-2	35.00	78.18
11	-21	2	84.52	67.75
16	-27	1	25.88	70.26
12	-23	2	73.95	59.43
-36	25	-1	-96.27	96.27
-22	3	5	46.22	77.39
-30	11	4	20.73	106.31
-21	-3	6	51.77	98.12
17	-45	2	-18.75	73.43
-20	-12	7	0.00	75.80
-12	-22	7	19.68	54.67
-9	13	-4	111.86	48.86
-11	15	-5	87.16	40.54
12	-9	-9	8.72	94.82
18	-17	-7	11.62	96.93
-14	15	-2	34.73	67.62
17	-18	-10	44.64	84.65
23	-27	-7	-22.05	85.84
-35	21	1	-15.45	109.61
23	-39	-1	101.03	87.69
-36	19	2	-10.30	96.54
16	-34	2	65.63	73.95
-35	16	3	41.47	95.61
-24	4	5	74.61	86.63
-26	5	5	0.00	99.18
9	-31	4	-25.36	59.96
-28	6	5	34.73	99.84
14	-40	3	61.67	68.01
10	-36	4	0.00	57.58
-29	1	6	-22.05	79.24
14	-43	3	-30.77	67.35
-17	-15	7	-57.18	66.16
-30	-7	7	-102.22	86.90
15	-12	-6	14.39	75.67
14	-11	-8	25.22	96.40
-15	18	-6	-15.72	48.20
9	-7	-2	657.93	80.03
-16	18	-4	72.90	58.90
16	-16	-10	-10.43	84.78
-3	3	0	9677.70	786.03
19	-20	-9	15.32	82.67
13	-17	0	193.21	77.78

-20	16	0	0.00	89.80
0	-5	2	57.84	16.38
-34	27	-4	-61.54	83.46
22	-30	-2	52.82	88.88
25	-34	-4	17.04	87.82
24	-33	-3	60.62	99.18
10	-19	2	239.56	73.16
-36	27	-3	4.89	82.41
-36	26	-2	-35.13	94.56
22	-34	-1	80.16	104.72
13	-25	2	48.33	66.16
24	-37	-2	-70.39	86.10
-22	7	4	114.63	80.03
-24	8	4	0.00	76.73
-15	-2	5	-2.25	66.56
-26	9	4	16.24	81.09
-28	10	4	-37.51	93.90
22	-44	0	0.00	81.35
17	-39	2	-32.75	72.63
-10	-13	6	9.77	66.56
-16	-7	6	87.29	85.05
-23	-2	6	51.37	88.61
-4	-22	6	-11.09	32.62
-25	-1	6	-4.23	83.20
-27	0	6	62.07	82.80
-22	-11	7	93.76	77.92
-28	-8	7	52.56	83.59
13	-10	-4	528.38	102.74
17	-15	-7	4.49	94.29
13	-11	-3	25.62	80.56
18	-18	-9	-38.03	85.71
14	-14	-2	1477.10	154.91
10	-10	-1	290.01	60.62
-22	22	-5	-117.27	73.43
-28	25	-5	-23.64	98.12
-34	24	-1	7.26	111.99
17	-29	1	2.11	70.52
-4	-8	4	95.61	48.60
21	-36	0	24.70	90.73
19	-36	1	63.39	89.01
-33	15	3	0.00	106.71
20	-44	1	-37.11	82.93
20	-47	1	20.73	82.80
17	-48	2	22.71	77.65
-24	-10	7	12.15	78.84
-26	-9	7	-36.18	82.80
10	-5	-6	82.41	69.99
5	0	-4	681.43	81.61
-24	23	-5	-26.68	78.84
-26	24	-5	50.84	97.33
-22	17	0	83.20	75.41
-4	-4	3	318.53	47.01
-7	-1	3	216.71	53.88
-17	7	3	415.73	96.54

−10	−2	4	37.77	62.33
14	−27	2	26.15	66.43
−33	20	1	0.00	105.91
−34	18	2	55.99	107.50
22	−41	0	−109.74	81.48
20	−41	1	52.96	82.54
−13	−10	6	−0.40	73.16
14	−46	3	19.55	69.86
9	−4	−5	215.52	80.82
17	−16	−9	−33.68	87.16
−10	7	1	1053.85	105.52
17	−21	−1	150.68	82.27
18	−23	−1	83.59	81.75
10	−15	1	207.60	61.67
−24	18	0	112.65	80.16
−26	19	0	223.45	91.52
−34	26	−3	141.57	91.65
19	−29	0	−84.12	76.33
−6	−11	5	19.02	45.30
13	−32	3	16.11	69.60
4	−28	5	−34.20	44.50
−33	7	5	16.24	84.52
−32	1	6	1.85	85.84
−16	−17	7	−36.71	66.29
−19	−14	7	4.62	72.50
3	3	−6	288.03	59.16
1	5	−7	−6.07	51.64
9	−4	−8	105.78	76.73
5	−2	−2	391.16	58.37
21	−22	−5	−14.53	84.12
20	−21	−4	−26.94	87.03
7	−8	0	769.65	82.27
22	−24	−5	34.20	102.74
21	−23	−4	155.30	88.22
22	−25	−4	9.11	84.92
−17	12	1	877.94	117.80
19	−25	−1	−4.23	79.37
−32	26	−4	80.56	92.97
−16	9	2	466.97	101.16
23	−32	−2	−13.21	103.93
−32	23	−1	76.46	105.65
−34	25	−2	8.19	110.27
25	−35	−3	−1.19	90.99
−31	14	3	83.86	112.12
−29	−9	7	−50.45	85.05
4	2	−7	−34.20	58.90
13	−9	−8	86.24	92.71
13	−9	−5	−14.66	75.14
11	−7	−9	−24.83	98.39
16	−13	−7	18.88	84.12
20	−20	−8	155.96	90.99
20	−20	−5	26.02	88.22
19	−19	−4	154.51	85.97
21	−22	−8	42.13	85.71

22	−24	−8	0.00	85.58
23	−26	−5	48.47	103.01
16	−19	−1	300.70	101.82
12	−15	0	411.11	75.27
5	−8	1	99.18	38.43
20	−27	−1	25.49	78.18
−31	19	1	56.65	92.44
23	−36	−1	6.07	97.20
18	−31	1	16.51	79.37
25	−39	−2	−21.13	85.84
−32	17	2	0.00	96.80
24	−41	−1	−35.52	92.44
−12	−5	5	92.31	65.90
−17	−1	5	179.60	75.94
17	−36	2	−14.53	76.73
−18	−6	6	−67.62	97.59
−30	0	6	−30.51	86.76
−21	−13	7	10.43	77.39
0	6	−6	25.49	41.34
11	−6	−7	5.81	71.45
14	−10	−6	299.91	90.33
16	−14	−9	17.43	92.97
19	−18	−8	−48.07	97.59
19	−18	−5	87.42	80.82
−18	19	−4	33.68	65.90
23	−27	−4	−6.74	99.05
−6	1	2	1576.41	138.93
−30	25	−4	−3.17	104.20
−30	22	−1	65.24	83.86
20	−31	0	−34.73	85.44
−19	8	3	−15.85	80.29
−7	−5	4	164.81	63.52
15	−29	2	14.13	66.29
22	−38	0	−29.19	84.78
−29	13	3	33.94	91.12
−31	6	5	8.45	92.18
18	−44	2	21.79	76.73
−15	−19	7	12.28	64.97
−27	−10	7	0.00	85.31
18	−17	−4	42.13	82.14
13	−12	−2	238.50	77.92
−16	17	−3	−31.17	70.39
−16	16	−2	396.45	83.33
19	−20	−3	29.71	76.73
23	−25	−6	7.40	103.40
20	−22	−3	17.56	80.56
21	−24	−3	22.05	79.10
24	−28	−5	52.03	104.86
22	−26	−3	32.22	84.12
24	−29	−4	43.45	108.95
−32	25	−3	12.15	105.78
−32	24	−2	−65.37	106.04
−12	3	3	270.33	73.03
−29	18	1	11.09	79.10

−9	−8	5	16.64	54.41
20	−38	1	−6.34	81.48
10	−33	4	20.34	64.58
−20	−5	6	24.04	95.88
−28	−1	6	19.81	84.25
6	−38	5	0.00	54.15
−23	−12	7	−41.73	80.29
−25	−11	7	85.44	82.67
6	0	−6	334.91	67.22
15	−17	−1	310.61	91.78
24	−27	−6	−78.97	95.22
25	−30	−5	−12.94	97.20
−19	13	1	−14.00	89.67
21	−29	−1	−6.60	77.52
−18	10	2	65.50	82.93
26	−37	−3	15.19	86.63
−21	9	3	67.88	74.61
−25	11	3	167.45	84.92
−30	16	2	33.68	84.52
−27	12	3	11.49	80.95
−19	0	5	296.87	82.67
18	−41	2	25.36	75.67
−29	5	5	64.84	94.42
15	−42	3	−12.41	70.92
−26	−2	6	138.66	86.90
18	−47	2	−26.94	82.67
11	−41	4	72.24	64.31
−18	−16	7	0.00	71.71
18	−16	−8	−0.40	99.71
20	−19	−6	58.64	90.20
18	−18	−3	125.46	80.16
−13	11	0	31.83	60.62
−20	17	−1	−41.86	82.93
−28	24	−4	125.46	103.93
23	−28	−3	−73.56	95.48
−28	21	−1	277.99	83.46
−27	17	1	−41.73	84.65
−23	10	3	105.65	80.03
−37	24	−1	95.61	99.71
19	−33	1	83.99	99.31
23	−43	0	15.32	84.78
−27	4	5	33.94	111.33
−9	−15	6	−26.28	69.20
21	−46	1	29.58	84.12
−22	−4	6	0.00	89.80
11	−38	4	9.38	64.31
−24	−3	6	1.45	84.52
15	−45	3	−40.15	70.78
−14	−21	7	2.77	63.79
−12	−25	7	−38.17	58.50
−28	−11	7	0.00	84.12
7	−1	−7	−15.58	66.69
15	−11	−7	−25.49	76.46
8	−4	−3	390.64	65.37

15	−12	−9	62.86	91.52
12	−9	−3	509.49	85.44
−9	11	−2	853.25	86.63
−17	19	−6	−64.71	56.92
−20	20	−4	131.27	69.33
25	−29	−6	−32.35	94.82
25	−31	−4	−29.05	98.25
−26	20	−1	57.45	81.61
−30	24	−3	0.00	102.61
−21	14	1	621.22	110.54
−30	23	−2	−35.52	89.41
−23	15	1	133.78	79.37
−25	16	1	28.00	89.54
−37	27	−4	63.92	86.63
21	−33	0	−3.57	99.44
−28	15	2	11.23	82.01
24	−38	−1	43.98	92.44
16	−31	2	−14.92	71.84
−36	20	1	−14.66	100.23
14	−34	3	−65.63	71.97
−21	1	5	43.84	76.07
−25	3	5	−11.09	102.48
−15	−9	6	−1.06	89.93
6	−35	5	−14.53	54.94
−13	−23	7	0.00	60.09
2	4	−8	32.62	60.09
8	−3	−4	117.01	68.67
19	−17	−6	127.31	87.16
17	−15	−4	96.54	75.80
−22	21	−4	24.70	70.65
−24	22	−4	−73.95	71.97
−26	23	−4	85.84	79.90
−24	19	−1	72.90	82.93
26	−32	−5	−0.26	92.84
24	−30	−3	45.83	106.44
−11	5	2	337.15	69.33
25	−36	−2	5.68	92.31
−26	14	2	121.36	90.59
−34	14	3	17.17	98.12
−23	2	5	0.00	82.14
15	−39	3	−25.62	72.50
−31	−1	6	−15.45	90.59
−20	−15	7	−43.05	77.39
−26	−12	7	−60.35	85.18
5	1	−8	42.92	64.71
17	−14	−8	74.35	108.55
−9	12	−3	227.81	51.37
17	−16	−3	−0.66	87.56
22	−22	−7	31.17	97.20
−18	18	−3	225.03	67.75
23	−24	−7	−74.48	105.91
14	−15	−1	128.63	81.09
−18	17	−2	125.33	82.80
24	−26	−7	31.30	91.91

11	-13	0	82.67	58.77
-28	23	-3	0.00	85.44
-28	22	-2	0.00	77.78
26	-33	-4	35.00	93.76
-22	12	2	82.27	86.10
-24	13	2	115.03	84.65
-37	25	-2	-46.22	92.44
23	-40	0	14.13	89.93
-35	17	2	-36.85	109.48
18	-38	2	-20.87	76.60
-12	-12	6	83.99	81.75
-17	-18	7	15.05	70.92
-22	-14	7	0.00	79.24
-24	-13	7	-2.77	79.24
13	-8	-6	27.34	75.41
12	-7	-8	107.76	87.95
21	-20	-7	0.00	111.59
20	-23	-2	95.48	84.78
21	-25	-2	98.25	84.25
9	-13	1	158.87	51.64
-3	-2	2	373.86	47.01
25	-32	-3	75.94	104.06
23	-33	-1	29.58	109.48
-37	26	-3	-103.40	89.67
-35	23	-1	17.56	110.01
-14	-4	5	214.60	75.01
21	-40	1	25.22	81.48
5	-30	5	3.43	48.33
-32	5	5	103.54	87.29
5	1	-5	405.56	77.26
2	4	-5	80.69	57.05
12	-7	-5	132.59	80.03
10	-5	-9	-0.66	101.03
18	-15	-6	61.01	83.99
18	-17	-10	0.00	89.67
9	-8	-1	116.87	52.69
21	-21	-9	34.07	79.50
-11	11	-1	1223.68	117.93
19	-21	-2	0.00	80.16
-20	18	-2	4.36	71.45
-26	22	-3	124.01	74.61
22	-27	-2	18.75	82.01
-26	21	-2	90.20	76.99
-31	20	0	-80.03	89.67
22	-35	0	29.19	101.95
20	-35	1	-46.49	98.12
-34	19	1	34.60	114.76
17	-33	2	0.00	78.18
7	-24	4	57.58	47.28
-32	13	3	0.00	120.57
-17	-8	6	-1.85	95.22
-29	-2	6	0.00	90.20
-27	-13	7	86.24	88.48
9	-3	-6	64.71	66.82

−2	8	−7	108.03	42.52
14	−10	−9	59.30	95.74
−4	8	−3	361.32	37.11
20	−18	−7	1.06	106.57
20	−19	−9	53.09	98.52
−19	20	−6	63.92	68.41
18	−19	−2	0.00	82.54
−20	19	−3	69.60	70.39
−22	19	−2	45.16	80.95
−24	21	−3	54.01	77.92
−12	8	1	303.87	66.29
−24	20	−2	46.22	77.78
23	−29	−2	0.00	90.73
−35	26	−4	−33.02	89.67
−14	4	3	664.27	100.76
26	−38	−2	−3.57	87.95
10	−23	3	1.72	53.75
11	−25	3	30.90	60.88
25	−40	−1	−50.58	86.63
19	−46	2	−33.02	84.78
−16	−20	7	0.00	61.94
−19	−17	7	−9.11	74.48
10	−4	−7	136.95	71.58
8	−2	−8	28.39	73.03
14	−9	−7	6.47	79.50
16	−12	−8	0.00	110.80
16	−13	−4	157.94	83.07
12	−10	−2	312.59	75.54
−8	7	0	701.77	78.31
−22	20	−3	210.11	79.10
−15	12	0	302.95	76.33
26	−34	−3	−42.26	96.40
12	−27	3	12.02	62.46
6	−22	4	83.73	43.05
−33	16	2	−1.98	116.35
8	−26	4	4.49	49.13
19	−43	2	81.35	82.54
11	−35	4	0.00	64.84
−30	4	5	45.30	91.65
−27	−3	6	−10.17	84.78
−21	−16	7	8.58	78.97
−25	−14	7	66.56	85.18
8	−5	−2	854.96	90.86
−13	16	−5	−22.45	50.71
16	−14	−3	−103.14	98.52
24	−31	−2	1.19	104.46
−29	19	0	0.66	81.61
−35	25	−3	9.64	101.03
24	−35	−1	37.24	98.78
−33	22	−1	32.88	115.29
−35	24	−2	−5.55	108.29
9	−21	3	−42.13	52.43
−32	18	1	−38.96	103.80
−30	12	3	78.44	101.03

15	−36	3	0.00	64.18
22	−45	1	0.00	89.54
−19	−7	6	0.00	91.78
−25	−4	6	46.62	87.95
19	−49	2	−6.34	90.73
−23	−15	7	31.83	84.12
−28	−14	7	20.73	90.20
17	−13	−6	81.88	79.50
16	−12	−5	175.64	84.92
19	−16	−7	117.27	105.12
19	−17	−9	−3.57	92.31
18	−24	0	36.32	77.39
16	−24	1	44.77	72.90
23	−37	0	−13.87	91.12
−36	21	0	−11.36	108.03
24	−42	0	12.41	89.14
−16	−3	5	103.80	71.58
−8	−17	6	18.49	69.46
−28	3	5	−62.73	97.20
−21	−6	6	0.00	89.93
16	−44	3	−25.49	76.07
−23	−5	6	0.00	87.03
−15	−22	7	0.00	69.07
8	−2	−5	326.85	85.31
23	−23	−8	10.96	93.24
17	−17	−2	428.41	103.54
13	−13	−1	247.48	71.31
17	−22	0	99.18	77.78
−13	6	2	51.64	62.07
17	−26	1	29.71	75.14
−9	0	3	262.54	63.26
21	−37	1	16.77	90.46
13	−29	3	−23.24	64.97
−31	15	2	12.81	98.25
18	−35	2	−17.43	80.69
−28	11	3	−48.20	85.84
−11	−7	5	222.92	66.69
−37	19	1	49.92	102.88
9	−28	4	22.98	57.05
−35	13	3	−8.72	105.52
16	−41	3	44.11	76.07
−14	−11	6	1.58	87.29
−33	4	5	16.77	88.74
−30	−3	6	159.53	96.40
−18	−19	7	31.17	77.78
−14	−24	7	0.00	66.69
−26	−15	7	0.00	89.41
22	−21	−8	16.51	90.86
23	−23	−5	101.95	106.04
24	−25	−5	−11.36	108.29
−7	4	1	2726.27	233.09
−17	13	0	239.95	89.27
25	−33	−2	−88.22	115.29
20	−28	0	−0.66	79.50

−33	25	−4	−62.07	97.99
27	−36	−3	−1.72	92.84
−27	18	0	36.05	85.84
18	−28	1	178.02	77.65
−16	5	3	24.17	84.78
−30	17	1	64.97	87.42
−38	23	−1	21.92	96.67
26	−42	−1	−21.39	94.16
22	−42	1	−30.77	91.25
−26	2	5	−14.92	107.89
12	−40	4	0.00	66.29
16	−47	3	−19.94	78.97
12	−43	4	0.53	70.39
−24	−16	7	17.56	88.22
11	−6	−4	131.27	80.82
18	−14	−7	22.45	95.74
11	−7	−3	1373.57	139.46
18	−15	−9	31.17	90.99
21	−19	−8	13.34	94.95
22	−21	−5	−77.92	90.07
22	−22	−4	30.77	83.33
3	−3	0	7928.68	644.59
23	−24	−4	0.13	86.63
25	−27	−5	0.53	108.16
24	−26	−4	98.12	111.06
16	−20	0	67.62	91.65
−25	17	0	144.21	85.18
−31	21	−1	72.63	94.03
−33	23	−2	87.56	114.10
25	−37	−1	−106.71	96.67
−5	−13	5	35.79	46.49
−36	16	2	0.00	103.67
−20	−18	7	−65.77	82.14
−22	−17	7	−16.51	85.18
11	−5	−8	14.92	84.39
15	−10	−8	−13.21	103.14
13	−8	−9	103.01	98.52
15	−11	−4	−49.65	97.99
−11	14	−4	209.84	61.01
25	−26	−6	40.54	98.91
26	−29	−5	7.79	101.16
25	−28	−4	−7.53	111.86
−14	9	1	77.78	64.84
−19	14	0	208.00	92.97
−29	24	−5	0.00	101.69
14	−20	1	113.18	85.44
21	−30	0	7.92	82.01
−33	24	−3	−25.09	111.86
19	−30	1	24.83	80.03
−18	6	3	−21.92	91.52
−9	−4	4	173.53	72.24
−29	14	2	182.77	88.88
−26	10	3	229.65	89.01
−1	−18	5	−33.02	11.36

−20	−1	5	37.90	76.07
−22	0	5	−6.47	83.73
−24	1	5	10.30	100.23
−11	−14	6	46.88	82.54
−28	−4	6	−25.62	92.44
7	−40	5	0.00	60.09
−17	−21	7	40.01	76.07
12	−6	−6	199.81	79.10
21	−19	−5	0.00	83.86
21	−20	−4	81.22	88.48
16	−15	−2	294.50	94.42
−6	7	−1	218.96	44.64
26	−28	−6	0.00	106.44
27	−31	−5	0.00	101.95
26	−30	−4	135.10	114.76
20	−24	−1	−21.39	82.80
21	−26	−1	9.64	84.39
−21	15	0	3.17	95.74
−23	16	0	115.29	82.41
8	−19	3	11.09	54.67
−28	16	1	−23.11	84.52
−38	26	−4	−59.82	93.63
−34	20	0	−19.15	117.01
−38	24	−2	−35.39	99.18
24	−39	0	86.50	92.05
5	−20	4	55.99	37.24
−24	9	3	47.67	91.91
14	−31	3	0.00	69.46
−8	−10	5	96.80	58.11
10	−30	4	28.13	64.97
−33	12	3	74.35	120.31
6	−32	5	0.00	49.92
−31	3	5	60.48	94.03
13	−7	−7	47.67	77.39
16	−11	−6	71.31	83.07
20	−17	−8	−40.81	103.67
15	−12	−3	171.15	90.59
22	−20	−6	172.87	111.06
22	−23	−3	46.22	88.22
10	−11	0	377.03	62.20
−13	12	−1	320.12	67.62
23	−25	−3	16.11	89.01
24	−27	−3	−55.60	97.99
19	−22	−1	177.23	81.09
22	−28	−1	0.00	86.37
−31	24	−4	47.54	107.10
−29	20	−1	65.90	83.33
22	−32	0	21.00	101.03
−20	7	3	49.52	76.86
−38	25	−3	−30.51	94.95
−22	8	3	62.07	83.99
−35	18	1	2.51	117.93
16	−38	3	58.50	77.39
20	−45	2	71.45	88.35

12	−37	4	30.90	68.80
−33	8	4	−7.66	95.22
−16	−10	6	−9.38	92.84
7	−37	5	3.04	59.43
−26	−5	6	35.13	90.46
−16	−23	7	−1.72	69.20
−19	−20	7	99.44	83.46
−23	−18	7	0.00	90.99
15	−10	−5	35.92	76.33
17	−13	−9	61.54	97.46
20	−18	−4	43.45	87.82
15	−18	0	96.67	84.65
25	−29	−3	71.05	116.48
−27	23	−5	75.14	106.84
27	−32	−4	39.75	97.73
−15	7	2	132.33	74.88
−31	22	−2	−88.35	98.91
14	−24	2	102.74	69.33
15	−26	2	53.75	69.46
20	−32	1	49.39	93.37
−27	13	2	4.09	90.33
−36	22	−1	46.75	103.67
22	−39	1	62.86	86.24
19	−37	2	15.32	82.67
25	−44	0	6.87	93.63
−34	15	2	46.22	114.63
23	−47	1	25.49	93.90
20	−48	2	0.00	92.05
−21	−19	7	−5.15	84.65
11	−5	−5	43.84	85.71
17	−12	−7	−30.77	90.99
2	0	−1	1411.60	118.85
−15	17	−5	−21.00	58.50
−11	12	−2	201.79	55.73
25	−25	−7	−14.53	97.59
21	−21	−3	338.47	95.88
18	−20	−1	−35.66	92.71
13	−18	1	63.79	76.46
−8	2	2	1425.73	129.16
−16	10	1	87.16	76.99
−31	23	−3	7.53	116.61
−26	15	1	66.03	82.93
16	−28	2	94.69	74.09
26	−39	−1	29.98	96.40
20	−42	2	0.00	81.75
−18	−9	6	74.88	92.05
−29	2	5	0.00	95.48
−24	−6	6	40.15	90.33
19	−15	−8	−12.15	109.35
21	−18	−6	33.02	96.14
5	−3	−1	1251.54	120.84
24	−23	−7	−9.77	104.72
26	−31	−3	5.02	113.44
−27	19	−1	−50.32	83.99

13	−22	2	2.64	62.46
27	−37	−2	−5.81	95.08
23	−34	0	−56.65	115.42
−25	12	2	41.20	90.46
−32	19	0	0.00	102.35
15	−33	3	0.00	76.07
−13	−6	5	4.23	70.65
−31	11	3	19.68	115.82
23	−44	1	78.18	84.65
−20	−8	6	6.87	91.25
−22	−7	6	−36.45	87.82
11	−8	−2	637.33	84.12
23	−21	−7	5.41	101.55
20	−19	−3	65.50	88.74
8	−11	1	256.20	50.71
−25	22	−5	8.06	95.22
−29	23	−4	70.78	107.37
−18	11	1	505.40	101.16
24	−32	−1	−97.46	110.93
−17	8	2	106.04	85.31
−24	14	1	210.64	87.69
21	−34	1	89.93	108.55
17	−30	2	64.05	77.12
−33	17	1	45.96	115.42
−37	20	0	13.34	113.97
−31	7	4	61.14	114.23
17	−43	3	0.00	78.97
17	−46	3	35.26	80.29
6	1	−7	−28.13	64.45
14	−8	−8	43.98	101.82
19	−16	−4	150.55	97.99
22	−20	−9	103.80	95.61
−11	13	−3	34.86	59.69
12	−11	−1	77.39	65.90
27	−33	−3	0.00	102.48
7	−13	2	33.54	46.09
−20	12	1	213.15	101.16
−29	21	−2	14.66	84.25
−22	13	1	0.40	92.97
−11	1	3	191.22	66.82
−19	9	2	348.11	100.50
−21	10	2	251.84	93.10
−36	25	−4	37.77	94.16
−23	11	2	288.16	86.63
−34	21	−1	45.56	122.68
−36	23	−2	0.00	105.12
25	−41	0	0.00	92.97
−32	14	2	−24.56	107.23
−13	−13	6	10.56	88.22
−27	1	5	−19.15	98.91
−32	2	5	−71.45	99.31
9	−2	−7	31.96	70.12
12	−6	−9	−110.93	110.14
16	−11	−9	−19.02	96.54

20	−16	−6	129.29	86.63
22	−19	−7	32.22	112.91
15	−13	−2	550.43	101.55
−17	18	−5	72.63	65.50
17	−18	−1	270.86	96.40
23	−26	−2	194.39	89.27
−25	18	−1	80.29	81.22
−29	22	−3	−113.84	96.14
25	−34	−1	0.79	109.21
−36	24	−3	60.35	100.89
−6	−7	4	131.14	62.33
27	−41	−1	−29.05	96.27
23	−41	1	−12.68	91.91
−29	10	3	0.00	101.82
13	−42	4	−48.07	75.54
−27	−6	6	72.77	97.86
15	−9	−6	122.55	83.07
18	−13	−8	35.79	110.40
21	−18	−9	−35.00	100.76
6	−6	0	2415.53	208.39
22	−24	−2	119.25	86.50
14	−16	0	60.35	77.78
−10	8	0	55.86	55.07
−23	21	−5	22.85	77.78
24	−28	−2	28.66	89.93
28	−35	−3	46.62	98.78
12	−20	2	79.76	75.80
3	−12	3	388.66	40.41
−6	−3	3	157.55	48.20
28	−39	−2	−30.11	91.12
24	−36	0	−65.90	103.01
−30	18	0	62.86	85.97
18	−32	2	89.80	81.88
20	−39	2	17.96	83.07
−15	−5	5	72.90	73.95
17	−40	3	7.00	79.90
−29	6	4	−28.53	124.40
−34	11	3	7.26	105.25
−25	0	5	−77.52	111.59
−34	7	4	−0.13	96.14
−30	−6	6	79.37	102.88
8	−1	−6	0.00	64.18
7	0	−8	331.74	80.82
5	2	−6	42.13	59.56
3	4	−7	271.25	65.90
−1	7	−5	102.48	43.45
14	−10	−3	756.45	110.54
19	−17	−3	56.92	85.31
−19	19	−5	263.59	74.61
21	−22	−2	−25.22	96.40
−21	20	−5	−25.36	72.37
25	−30	−2	0.00	115.95
−27	22	−4	−59.30	90.73
−23	17	−1	−2.11	71.58

-27	20	-2	75.41	82.54
22	-36	1	-23.51	100.23
-11	-3	4	233.88	82.14
-31	16	1	-87.42	91.91
16	-35	3	0.00	77.52
21	-47	2	127.17	94.95
-10	-16	6	8.06	80.03
-30	1	5	-68.14	96.67
-25	-7	6	107.76	96.54
16	-10	-7	30.11	84.12
7	-1	-4	105.65	64.31
4	1	-3	307.31	50.05
21	-17	-7	-5.28	115.29
-13	15	-4	3.83	64.05
26	-36	-1	11.75	105.12
-35	19	0	-54.01	126.78
-30	13	2	-63.52	90.86
-27	9	3	-19.02	86.50
-36	17	1	-147.91	113.57
-17	-4	5	175.11	78.84
21	-44	2	-2.11	92.57
-23	-1	5	-68.28	106.84
13	-39	4	-3.57	71.31
7	-34	5	87.29	57.84
-15	-12	6	25.62	88.61
10	-3	-8	179.07	89.14
4	3	-8	322.63	73.43
14	-8	-5	-20.34	75.67
-3	9	-6	41.07	23.51
18	-14	-4	295.02	86.63
24	-22	-8	51.24	97.59
8	-6	-1	1343.85	125.85
20	-20	-2	23.51	84.92
-13	13	-2	255.01	70.78
12	-16	1	345.34	78.18
26	-32	-2	-20.21	119.91
-27	21	-3	-16.90	80.16
7	-17	3	330.68	63.79
-34	24	-4	51.50	99.05
-28	17	0	-8.06	86.63
-32	20	-1	30.11	112.65
-34	22	-2	20.07	120.44
-3	-10	4	209.05	51.90
26	-43	0	83.46	97.73
-35	14	2	-19.94	107.37
24	-46	1	0.00	97.59
12	-34	4	-0.13	70.52
-19	-3	5	38.03	80.95
-27	5	4	-63.39	106.71
-21	-2	5	60.22	90.07
-23	-8	6	63.13	91.39
12	-5	-7	125.06	79.50
11	-4	-6	590.18	94.03
4	2	-4	160.45	56.26

19	−14	−6	61.01	86.76
20	−16	−9	102.22	96.27
23	−20	−8	0.00	103.80
26	−26	−5	170.49	116.21
16	−16	−1	452.97	106.97
27	−28	−5	−32.22	108.95
−9	5	1	779.95	86.24
−25	21	−4	47.54	81.61
−25	19	−2	0.26	87.56
−32	24	−5	−122.82	101.95
−13	2	3	0.00	78.84
25	−38	0	3.30	91.91
19	−34	2	0.00	85.44
−37	21	−1	−20.07	110.67
−10	−9	5	192.94	65.37
−32	10	3	−19.68	121.63
−32	6	4	64.31	104.86
−17	−11	6	−19.81	91.39
−28	−7	6	−3.30	103.01
10	−4	−4	156.36	82.01
18	−13	−5	0.00	83.99
−10	14	−6	222.92	43.32
18	−15	−3	−29.98	90.33
25	−24	−5	16.77	117.53
25	−25	−4	−65.50	110.14
26	−27	−4	−22.58	122.55
28	−30	−5	−38.17	107.10
27	−34	−2	0.00	104.72
−34	23	−3	23.90	114.76
−29	15	1	72.77	91.25
−25	8	3	62.99	87.95
24	−43	1	0.13	99.44
−28	0	5	−49.39	97.99
−19	−10	6	24.43	91.65
−21	−9	6	97.06	94.03
17	−11	−8	−7.00	112.65
15	−9	−9	0.00	101.16
20	−15	−7	−10.56	113.97
10	−5	−3	716.17	91.12
24	−22	−5	−19.02	119.91
24	−23	−4	−65.77	94.29
27	−27	−6	228.73	104.06
27	−29	−4	73.16	117.53
−25	20	−3	−4.23	79.37
27	−38	−1	78.84	100.37
23	−38	1	10.43	93.24
−28	12	2	−0.13	88.48
−25	4	4	35.13	88.08
18	−45	3	44.50	83.33
−6	−21	6	0.00	50.84
8	−39	5	66.43	64.97
22	−18	−8	15.05	99.97
19	−18	−2	0.00	90.33
−13	14	−3	0.00	67.48

28	−31	−4	108.95	104.46
−23	20	−4	−5.41	77.39
20	−25	0	1.98	84.92
−23	18	−2	0.40	78.97
22	−29	0	88.48	86.24
11	−18	2	224.90	77.92
−10	3	2	3735.35	314.97
−26	16	0	90.46	90.99
−13	−2	4	163.23	78.58
−33	18	0	86.37	110.67
−23	7	3	171.55	91.65
−34	16	1	−66.03	124.80
−35	10	3	74.75	104.20
−31	0	5	0.00	96.27
−29	−8	6	−35.66	102.22
13	−6	−8	26.54	100.10
19	−14	−9	−4.75	105.38
24	−21	−6	85.44	119.91
23	−20	−5	−38.96	99.84
14	−11	−2	648.16	98.78
23	−21	−4	14.92	91.65
−15	16	−4	48.60	59.69
13	−14	0	167.45	67.09
−15	14	−2	45.96	74.61
−12	9	0	579.75	78.84
29	−33	−4	102.48	101.82
19	−27	1	61.01	82.93
−30	19	−1	27.07	89.54
−32	21	−2	20.73	115.29
−15	3	3	35.79	86.37
26	−40	0	10.70	94.42
−37	22	−2	−66.82	114.50
20	−36	2	0.00	85.44
−33	13	2	−28.26	121.63
18	−42	3	−18.09	82.54
−30	5	4	0.00	111.06
−12	−15	6	−11.89	87.03
−26	−1	5	72.90	105.91
18	−12	−6	−14.13	94.16
25	−26	−3	3.96	108.82
26	−28	−3	61.94	118.46
−21	19	−4	83.73	76.86
19	−23	0	548.98	97.99
−21	17	−2	88.35	82.93
18	−25	1	124.80	80.43
23	−31	0	46.35	104.06
20	−29	1	15.85	78.58
−32	23	−4	−72.50	114.63
−17	4	3	238.77	98.91
−27	14	1	198.22	90.46
−21	6	3	52.03	86.63
−26	11	2	70.39	92.18
−35	20	−1	−67.75	127.84
−23	3	4	−34.34	88.88

-30	9	3	56.65	105.52
13	-36	4	-14.66	73.43
15	-8	-7	-44.77	83.59
14	-7	-6	801.48	116.87
10	-3	-5	302.16	86.76
7	0	-5	1366.83	141.57
2	5	-6	17.04	50.45
21	-16	-8	-2.77	102.35
17	-12	-4	42.39	85.31
26	-24	-7	103.80	100.50
24	-24	-3	266.10	98.65
9	-9	0	1172.17	112.91
-17	17	-4	190.17	69.73
-19	18	-4	7.26	73.03
-17	15	-2	8.06	83.99
27	-30	-3	0.00	119.12
22	-25	-1	-29.85	95.61
-19	16	-2	86.50	89.01
23	-27	-1	-49.52	90.33
24	-29	-1	-65.50	94.29
-30	23	-5	30.77	107.50
-24	15	0	-62.46	85.18
21	-31	1	68.41	94.16
-32	22	-3	-11.36	122.16
28	-40	-1	5.02	99.71
-37	24	-4	34.34	104.06
-19	5	3	43.32	83.46
-37	23	-3	53.88	98.52
24	-40	1	93.24	97.20
-15	-1	4	66.43	74.61
-7	-12	5	144.21	58.37
-21	2	4	-22.32	84.52
-37	16	1	50.84	108.29
-36	13	2	40.81	108.82
22	-46	2	0.00	96.67
22	-49	2	19.41	101.55
-33	5	4	46.62	105.65
14	-44	4	-39.88	81.61
19	-13	-7	30.77	102.74
13	-7	-4	121.63	88.08
23	-19	-6	296.48	124.01
7	-3	-2	2378.42	204.56
25	-22	-7	17.17	111.46
22	-19	-4	30.11	99.05
23	-22	-3	38.56	91.25
15	-14	-1	171.42	79.10
-15	15	-3	25.49	74.48
18	-21	0	93.63	88.08
-21	18	-3	152.00	79.24
28	-32	-3	92.84	110.54
17	-23	1	4.09	67.62
29	-38	-2	-18.36	99.97
24	-33	0	23.24	118.59
13	-26	3	-13.21	65.50

14	−28	3	81.09	68.28
−17	0	4	316.29	84.39
−19	1	4	43.58	87.16
25	−45	1	−7.66	103.80
−12	−8	5	42.39	70.52
−24	−2	5	227.94	117.01
14	−41	4	0.00	81.35
−29	−1	5	−98.12	106.84
−27	−9	6	78.71	106.71
17	−11	−5	73.16	89.01
17	−13	−3	1.06	97.73
18	−16	−2	179.21	104.46
11	−9	−1	647.76	84.39
21	−23	−1	151.61	89.67
−14	10	0	865.53	100.50
−22	14	0	76.99	89.80
−28	18	−1	161.51	94.82
22	−33	1	14.39	112.25
−25	13	1	32.49	89.80
−24	10	2	160.45	89.01
−31	17	0	91.39	99.57
15	−30	3	−8.58	73.56
21	−38	2	0.00	90.59
−32	15	1	22.45	109.35
−36	18	0	47.94	122.42
−31	12	2	−3.04	99.44
22	−43	2	0.00	95.74
−28	4	4	−110.01	120.44
−33	9	3	−26.15	122.82
8	−36	5	346.79	44.11
−14	−14	6	15.45	88.08
−32	−1	5	43.84	105.12
18	−12	−9	27.34	102.48
13	−8	−3	391.96	90.20
24	−20	−7	−18.22	116.08
−17	16	−3	−67.62	76.86
−19	17	−3	135.23	73.16
29	−34	−3	−1.98	103.27
−11	6	1	59.43	55.86
26	−33	−1	65.11	126.65
−12	4	2	138.93	62.46
25	−35	0	−92.57	109.74
−30	20	−2	−0.66	92.57
−35	24	−5	0.00	100.23
12	−24	3	−0.66	65.24
−8	−6	4	−41.47	70.92
27	−42	0	9.51	101.16
−38	20	−1	−1.45	109.74
−28	8	3	59.43	96.93
18	−39	3	−21.79	82.54
−22	−3	5	73.16	109.21
−20	−11	6	−28.00	93.50
16	−9	−8	−48.47	113.70
14	−7	−9	−43.05	106.31

22	-17	-6	112.65	106.31
23	-19	-9	75.01	106.71
10	-6	-2	1119.61	114.23
22	-20	-3	1.58	93.10
-8	8	-1	120.31	46.75
20	-21	-1	46.22	84.78
11	-14	1	1499.55	141.17
-4	1	1	898.68	87.69
-16	11	0	711.94	101.03
-20	13	0	88.08	92.18
-30	22	-4	-88.35	119.52
-3	-6	3	52.30	38.69
4	-18	4	68.94	23.90
-35	21	-2	13.47	126.51
16	-32	3	-74.61	81.22
-14	-7	5	145.27	75.67
-16	-13	6	5.28	89.41
-18	-12	6	-24.56	94.03
-28	-10	6	97.33	107.76
13	-6	-5	212.75	80.69
20	-14	-8	-148.83	113.70
21	-17	-4	89.01	92.97
4	0	-2	3002.28	250.65
-5	4	0	693.72	74.61
-18	12	0	214.60	88.48
-28	22	-5	15.98	112.91
27	-35	-1	-43.18	109.61
-30	21	-3	-23.77	102.08
-8	-2	3	152.66	59.96
-23	12	1	121.89	90.07
23	-35	1	-43.84	107.63
-22	9	2	0.00	88.35
-33	19	-1	174.58	134.83
-35	15	1	9.11	124.40
-20	-4	5	-9.90	94.56
19	-44	3	111.59	91.25
-31	4	4	11.09	105.52
19	-47	3	0.00	95.22
-5	-23	6	-32.49	38.43
-27	-2	5	-73.69	102.48
-25	-10	6	-15.45	103.01
4	3	-5	972.89	106.31
23	-18	-7	0.00	130.08
24	-25	-2	0.00	90.73
25	-27	-2	-7.13	95.35
17	-19	0	71.31	92.05
26	-29	-2	69.60	114.63
-5	-1	2	1412.79	125.06
17	-27	2	72.24	77.39
26	-37	0	-68.28	106.57
18	-29	2	-13.47	79.90
-35	23	-4	-47.54	104.59
25	-42	1	-35.92	102.22
-26	7	3	148.04	93.90

−16	−6	5	51.11	80.56
−34	12	2	−24.43	124.40
−18	−5	5	83.20	83.33
−26	3	4	97.73	103.14
14	−38	4	0.00	76.86
−34	4	4	66.95	94.16
−30	−2	5	−87.56	103.01
18	−11	−7	97.06	101.29
17	−10	−6	259.76	91.39
22	−17	−9	157.81	108.82
27	−31	−2	−129.42	126.25
−14	5	2	6.21	72.37
19	−31	2	37.64	87.95
−20	8	2	34.47	97.99
−29	16	0	0.00	92.31
−35	22	−3	0.00	110.67
−30	14	1	−33.81	91.65
−34	17	0	99.57	128.76
22	−40	2	39.49	95.08
−29	11	2	89.54	97.86
−31	8	3	−106.57	130.61
23	−48	2	36.32	106.57
−29	−11	6	71.05	108.55
11	−3	−7	272.57	83.86
8	0	−7	43.45	71.97
0	7	−7	419.03	63.39
25	−21	−8	−32.88	107.76
21	−18	−3	146.32	96.14
29	−29	−5	74.48	111.86
23	−23	−2	40.01	96.27
19	−19	−1	344.15	101.16
−22	20	−6	17.70	84.52
28	−33	−2	3.17	117.27
−13	7	1	15.05	60.35
28	−37	−1	−42.92	110.01
16	−25	2	90.73	77.26
−28	19	−2	0.00	86.10
−16	6	2	211.69	90.99
11	−22	3	37.37	61.01
−18	7	2	408.46	98.78
24	−37	1	68.28	99.18
17	−34	3	8.72	85.97
10	−27	4	67.35	59.96
−38	21	−2	39.49	106.44
11	−29	4	−5.41	67.09
−23	−11	6	−94.69	106.71
−26	−11	6	13.34	109.61
9	−1	−8	34.86	83.86
21	−15	−6	45.30	98.91
17	−14	−2	379.94	105.38
−12	15	−6	84.65	51.90
27	−25	−5	114.76	125.46
28	−27	−5	30.90	113.97
15	−19	1	142.23	89.27

−19	10	1	382.71	103.54
−33	23	−5	6.07	105.38
20	−33	2	1.32	87.82
−36	19	−1	−79.10	120.04
23	−45	2	85.31	104.20
19	−41	3	−29.98	89.01
−24	2	4	−1.85	89.41
−34	8	3	19.41	111.72
−25	−3	5	13.60	112.38
12	−4	−8	44.11	99.71
17	−10	−9	117.40	105.78
22	−16	−7	2.25	123.21
24	−19	−8	27.07	104.72
20	−15	−4	74.88	92.05
13	−9	−2	19.94	68.14
27	−26	−4	−18.09	125.19
28	−28	−4	12.41	127.84
12	−12	0	457.99	73.43
29	−30	−4	−38.17	123.08
−26	21	−5	−33.68	106.31
−15	8	1	61.41	69.20
−28	21	−4	145.40	105.12
15	−23	2	169.43	71.18
−17	9	1	35.00	86.37
−28	20	−3	6.34	87.42
27	−39	0	−9.77	108.03
−33	20	−2	0.00	130.74
−38	23	−4	−8.98	106.04
−38	22	−3	26.15	112.78
−24	6	3	50.45	96.40
12	−31	4	−39.09	73.95
−37	17	0	11.62	120.04
−29	3	4	135.10	116.87
15	−43	4	−13.73	86.90
−8	−20	6	−27.34	67.62
−21	−12	6	0.00	99.84
14	−6	−7	71.58	82.27
10	−2	−6	25.62	74.48
19	−12	−8	64.31	129.68
16	−9	−5	24.17	83.59
21	−15	−9	36.98	106.04
20	−14	−5	26.81	98.78
16	−11	−3	173.26	110.80
26	−23	−5	34.60	124.27
22	−21	−2	−11.75	91.25
−20	19	−6	−11.75	78.44
30	−32	−4	36.32	113.31
29	−39	−1	35.52	106.84
−27	15	0	0.00	92.84
−10	−5	4	−20.60	75.01
−28	13	1	−6.34	90.46
9	−25	4	−46.35	54.01
−27	10	2	−8.98	90.86
18	−36	3	−167.98	87.42

−33	14	1	114.10	128.36
−9	−11	5	−20.73	62.60
−32	11	2	20.07	127.44
6	−29	5	23.64	49.92
−32	3	4	0.00	104.59
−28	−3	5	−31.30	105.12
−31	−3	5	26.54	110.67
−27	−12	6	63.52	111.99
13	−5	−6	56.39	81.88
6	2	−8	−35.13	75.67
26	−24	−4	−6.47	115.42
14	−12	−1	594.54	87.56
16	−17	0	54.54	87.82
4	−6	1	741.39	73.82
30	−37	−2	0.00	111.59
25	−39	1	−64.31	101.55
21	−35	2	1.58	96.27
−22	1	4	−14.39	87.16
−29	7	3	110.67	106.04
−23	−4	5	59.16	121.10
9	−38	5	−5.15	70.26
−24	−12	6	53.48	105.25
15	−7	−8	−13.21	115.03
7	1	−6	29.98	67.75
1	4	−3	298.59	39.62
26	−22	−6	0.92	120.18
25	−21	−5	56.13	129.68
7	−9	1	1243.22	113.70
−26	18	−2	3.96	97.86
−10	−1	3	115.03	67.48
−33	22	−4	−124.40	118.85
−33	21	−3	0.00	127.57
−32	16	0	0.00	117.27
−22	5	3	104.72	94.42
23	−42	2	29.58	105.91
−36	14	1	37.11	124.80
−35	11	2	−105.52	119.78
20	−46	3	−24.56	97.33
−4	−25	6	14.92	50.71
−28	−13	6	−30.90	119.25
5	3	−7	0.00	63.92
23	−17	−8	47.94	111.72
25	−22	−4	51.90	100.89
−14	16	−6	52.43	58.11
18	−17	−1	180.53	103.40
27	−27	−3	57.71	126.65
−18	18	−6	0.00	69.73
28	−29	−3	−54.41	132.99
−10	9	−1	29.71	47.01
29	−31	−3	−64.97	120.18
23	−28	0	0.00	77.65
24	−30	0	24.83	101.55
28	−41	0	−49.52	111.20
−36	20	−2	110.40	120.97

7	−31	5	−9.11	55.86
15	−40	4	51.90	85.31
−27	2	4	−33.02	127.70
−32	7	3	−105.52	132.33
21	−14	−7	19.55	123.61
20	−13	−6	−7.53	90.99
1	5	−4	614.08	66.03
25	−20	−6	−40.81	125.06
27	−23	−7	0.00	108.69
21	−19	−2	8.85	82.41
26	−25	−3	−31.03	113.18
−16	17	−6	0.00	63.52
30	−33	−3	39.88	111.33
−24	20	−5	38.30	93.37
−26	20	−4	−23.51	86.90
25	−32	0	−45.96	124.14
−36	23	−5	80.69	111.86
22	−37	2	51.50	101.29
−20	4	3	28.92	85.71
−25	9	2	−30.11	94.03
−20	0	4	33.02	86.50
−21	−5	5	12.02	116.87
−26	−4	5	−1.72	103.01
−22	−13	6	−50.84	106.97
−25	−13	6	20.07	114.36
17	−9	−7	−29.45	94.82
12	−5	−4	134.70	94.29
9	−2	−4	−7.26	73.03
24	−20	−4	−23.64	95.48
25	−28	−1	−29.45	103.27
26	−30	−1	58.77	114.50
14	−21	2	174.85	79.10
−26	19	−3	106.71	86.63
6	−15	3	150.42	49.79
−31	22	−5	32.35	112.65
−25	14	0	123.74	92.71
−31	19	−2	−2.91	102.22
−26	12	1	216.18	97.73
26	−41	1	34.60	108.69
−12	−4	4	164.94	77.65
19	−38	3	67.22	95.61
−18	−1	4	16.64	84.39
−35	16	0	40.54	132.33
−30	10	2	0.00	101.69
−11	−10	5	228.60	73.29
−27	6	3	71.84	98.12
24	−47	2	0.00	113.04
20	−43	3	0.00	94.03
−30	2	4	54.94	111.59
−33	2	4	−32.22	104.33
−29	−4	5	17.70	109.48
16	−8	−6	34.07	86.76
20	−13	−9	−49.26	108.69
19	−13	−4	177.62	94.42

26	−21	−7	35.79	116.35
25	−23	−3	−53.62	101.55
24	−26	−1	0.00	98.39
21	−24	0	99.97	93.10
14	−17	1	618.84	100.76
6	−11	2	489.95	57.58
21	−28	1	93.76	86.24
−24	17	−2	103.80	89.41
26	−34	0	−38.30	120.31
22	−30	1	23.37	93.37
10	−20	3	−17.56	56.65
−12	0	3	70.92	77.78
−18	3	3	53.09	89.01
−36	21	−3	0.00	119.65
−14	−3	4	413.22	82.27
−16	−2	4	26.15	80.03
−31	13	1	76.33	109.74
27	−46	1	−8.98	113.57
14	−35	4	−5.28	80.56
−19	−6	5	28.39	98.52
18	−10	−8	−69.86	123.61
16	−8	−9	113.57	113.84
19	−12	−5	205.22	94.82
12	−6	−3	357.75	83.46
16	−12	−2	341.77	95.61
3	−8	2	640.89	57.05
28	−34	−1	−73.95	115.95
20	−26	1	144.34	91.39
23	−32	1	3.17	113.57
−14	1	3	0.00	84.65
29	−43	0	23.37	111.72
−5	−9	4	251.44	66.95
−16	2	3	186.21	104.46
−36	22	−4	77.52	113.44
8	−23	4	2.77	53.22
24	−44	2	−24.43	113.84
−25	1	4	25.09	108.69
8	−33	5	−74.35	62.86
−10	−19	6	47.67	77.39
−20	−14	6	−45.56	103.40
−26	−14	6	−56.13	119.65
22	−15	−8	63.52	115.55
24	−18	−6	50.58	137.08
23	−24	−1	−49.26	91.78
−12	10	−1	668.76	83.59
−22	19	−5	−21.26	81.75
−18	13	−1	−26.54	89.67
27	−36	0	99.44	112.25
24	−34	1	−51.64	120.57
−31	21	−4	0.00	124.67
−31	20	−3	−43.45	119.12
−23	8	2	128.63	87.69
−30	15	0	−118.06	94.16
23	−39	2	6.74	99.97

−34	13	1	−82.67	134.04
−13	−9	5	52.82	78.05
−15	−8	5	4.49	81.48
−33	10	2	0.00	131.80
−17	−7	5	66.03	92.84
−36	10	2	−8.58	112.25
16	−45	4	−0.26	95.88
−24	−5	5	32.75	112.91
−30	−5	5	0.92	118.85
−23	−14	6	−74.75	111.33
−27	−15	6	−89.93	123.48
12	−4	−5	173.53	83.86
25	−19	−7	0.00	120.04
24	−18	−9	0.00	115.82
23	−18	−4	68.80	98.65
19	−14	−3	374.92	97.86
24	−21	−3	−14.53	100.76
20	−17	−2	49.26	96.27
20	−22	0	78.31	85.18
10	−12	1	704.02	82.67
−14	11	−1	1272.28	129.55
−16	12	−1	566.28	95.35
19	−24	1	−5.68	84.65
−24	19	−4	−14.79	85.84
−24	18	−3	166.40	91.65
29	−36	−1	161.77	116.35
−23	13	0	114.36	101.03
−24	11	1	50.98	84.65
−30	6	3	−12.55	135.76
−7	−22	6	0.13	68.41
−18	−15	6	10.56	100.50
9	−1	−5	208.92	87.56
10	−7	−1	1329.19	126.25
17	−15	−1	190.83	94.56
15	−15	0	41.73	80.95
27	−28	−2	3.17	127.31
28	−30	−2	−31.17	130.34
29	−32	−2	−101.95	125.59
−22	16	−2	72.50	83.46
−7	0	2	8240.34	674.30
25	−36	1	155.70	117.67
−34	19	−2	−29.45	135.63
27	−43	1	0.00	115.29
20	−40	3	0.79	95.88
−6	−14	5	0.00	58.50
−25	5	3	11.49	98.25
16	−42	4	−10.56	94.16
−28	1	4	−50.84	126.78
−3	−27	6	34.73	49.13
−12	−18	6	101.29	85.31
−27	−5	5	75.54	109.87
20	−12	−7	119.25	119.78
19	−11	−6	−63.52	90.86
15	−7	−5	32.09	81.35

6	1	−4	120.70	64.18
15	−9	−3	170.89	94.16
−8	12	−5	123.87	43.98
30	−28	−5	53.75	122.02
26	−26	−2	227.41	116.21
22	−22	−1	120.84	93.24
30	−34	−2	0.00	116.48
30	−38	−1	0.00	118.19
28	−38	0	163.89	109.48
−29	18	−2	−19.81	92.44
−34	22	−5	43.05	114.36
−28	9	2	−51.77	95.74
15	−37	4	0.00	85.84
−23	0	4	46.35	92.97
21	−48	3	29.05	109.08
−31	1	4	110.67	118.19
−14	−17	6	1.85	90.99
−16	−16	6	−16.38	95.74
−24	−15	6	13.47	116.21
19	−11	−9	27.20	114.76
23	−16	−6	27.47	112.78
26	−20	−8	12.15	117.80
29	−26	−5	65.24	125.19
7	−4	−1	2682.69	230.84
−7	5	0	−8.98	56.13
13	−19	2	149.76	85.71
−29	21	−5	19.41	129.16
16	−29	3	49.13	79.50
17	−31	3	39.49	88.61
−21	7	2	475.82	111.59
−29	12	1	40.81	95.88
−33	15	0	−6.87	126.65
−37	19	−2	−61.54	124.80
25	−49	2	11.36	128.36
21	−45	3	14.92	104.46
−22	−6	5	4.23	119.91
10	−40	5	26.81	82.14
−21	−15	6	122.55	110.54
13	−4	−7	0.00	85.05
21	−13	−8	−51.37	119.38
24	−17	−7	−31.83	129.02
23	−16	−9	70.65	115.03
23	−19	−3	30.11	102.61
29	−27	−4	0.00	129.55
30	−29	−4	28.79	126.38
25	−24	−2	171.81	100.37
31	−31	−4	−8.32	122.68
−20	18	−5	−5.15	78.18
−6	2	1	914.92	95.22
−21	12	0	27.86	112.91
26	−38	1	−47.15	110.80
−22	10	1	189.11	97.46
−28	14	0	59.69	98.39
−36	15	0	55.99	134.17

9	−35	5	59.16	70.78
−25	−16	6	28.13	120.44
14	−5	−8	−23.24	115.69
11	−2	−8	55.60	99.57
10	−1	−7	−28.92	79.63
4	4	−6	70.39	59.03
3	5	−8	−38.83	68.67
28	−24	−5	7.79	129.42
13	−10	−1	126.38	70.52
19	−20	0	20.34	95.74
−22	18	−4	20.60	83.99
9	−14	2	38.96	54.94
−20	15	−2	7.40	94.69
−22	17	−3	143.81	86.90
20	−30	2	4.09	90.99
29	−40	0	62.86	119.65
15	−27	3	22.58	72.37
−34	21	−4	172.60	124.93
−34	20	−3	0.00	129.55
18	−33	3	58.37	91.65
−23	4	3	145.53	96.14
25	−46	2	67.62	119.65
−31	9	2	88.74	119.65
−28	5	3	0.00	108.95
−34	9	2	−33.02	127.17
−34	5	3	−35.26	119.12
−25	−6	5	−11.49	113.70
−28	−6	5	0.00	115.29
−26	−17	6	−61.94	131.80
16	−7	−7	82.80	94.95
22	−15	−5	−71.18	103.40
22	−16	−4	124.14	99.18
28	−23	−6	−7.66	136.55
28	−25	−4	69.20	141.31
21	−20	−1	25.75	87.95
8	−7	0	686.72	79.63
19	−28	2	8.98	82.27
−29	20	−4	−58.37	133.51
−29	19	−3	143.42	103.27
21	−32	2	109.61	100.23
−19	6	2	142.10	106.71
−37	22	−5	−50.58	127.57
28	−45	1	220.41	125.19
−32	12	1	−20.60	120.70
−21	−1	4	15.98	88.74
−26	0	4	193.21	130.08
−20	−7	5	28.13	124.01
−32	0	4	−17.56	115.55
−19	−16	6	0.13	110.40
−22	−16	6	−13.21	119.38
17	−8	−8	0.00	131.14
18	−10	−5	38.56	92.05
25	−18	−8	130.74	118.99
27	−22	−5	6.34	142.89

12	−7	−2	296.08	73.03
9	−4	−2	1435.64	134.31
19	−15	−2	159.00	108.69
−6	10	−4	140.38	39.75
24	−22	−2	89.27	96.01
11	−10	0	453.23	72.37
−19	11	0	256.73	99.97
−5	−5	3	671.79	75.27
22	−34	2	158.08	111.20
19	−35	3	0.00	95.22
−37	20	−3	150.81	120.04
−26	8	2	9.11	106.71
21	−42	3	29.85	104.59
16	−39	4	−13.47	92.97
−35	12	1	0.00	131.93
−29	0	4	15.85	122.29
−9	−21	6	−20.34	79.50
15	−6	−6	234.14	93.24
12	−3	−6	174.32	85.97
18	−12	−3	4.23	104.72
27	−23	−4	−22.71	130.48
−6	9	−3	144.61	37.90
30	−30	−3	90.99	140.64
31	−32	−3	112.91	119.91
−18	17	−5	114.76	78.44
−25	20	−6	−76.73	127.84
18	−26	2	52.03	83.86
−9	1	2	2120.37	186.87
−27	17	−2	8.06	91.52
14	−25	3	247.88	78.44
−20	9	1	292.12	111.86
30	−42	0	0.00	123.48
27	−40	1	2.51	116.08
−32	18	−2	−18.75	130.21
−27	11	1	10.30	98.65
−37	21	−4	131.93	122.02
−17	−17	6	0.00	106.18
−29	−7	5	42.26	129.42
−23	−17	6	−20.73	126.78
8	1	−8	66.16	87.42
23	−15	−7	129.82	137.34
22	−14	−6	−5.41	114.10
27	−21	−6	0.00	129.82
22	−17	−3	107.37	103.27
29	−28	−3	−4.36	133.91
13	−15	1	429.33	82.93
−20	17	−4	100.50	82.93
−18	14	−2	97.20	92.31
−27	20	−5	0.00	123.08
−17	5	2	−3.17	91.78
23	−36	2	104.72	111.86
−7	−8	4	118.19	74.35
−31	14	0	141.44	114.50
25	−43	2	0.00	121.36

−21	3	3	334.51	98.39
−3	−17	5	100.89	48.73
−8	−13	5	41.47	65.50
−18	−8	5	−95.88	112.52
17	−44	4	0.00	104.99
19	−10	−7	25.62	115.69
22	−14	−9	108.42	121.23
6	2	−5	332.79	80.95
28	−22	−7	−8.45	126.25
26	−20	−5	0.13	121.63
15	−10	−2	377.03	93.10
28	−26	−3	−56.65	133.12
17	−20	1	−148.04	92.57
−9	6	0	521.64	70.12
25	−29	0	56.39	113.31
−20	16	−3	51.77	81.09
26	−31	0	69.73	130.21
−17	10	0	990.46	123.74
−32	21	−5	48.86	119.52
−26	13	0	279.44	110.14
−19	−2	4	70.92	92.18
−26	4	3	46.09	104.59
22	−47	3	−27.07	117.40
−6	−24	6	43.18	71.45
−23	−7	5	110.40	121.50
−11	−20	6	−2.77	88.35
−15	−18	6	21.92	97.06
−26	−7	5	31.43	117.14
−20	−17	6	−51.37	119.78
−24	−18	6	71.58	132.99
−25	−19	6	−26.54	142.49
26	−21	−4	98.91	115.16
23	−20	−2	0.00	96.54
16	−13	−1	293.57	90.46
20	−18	−1	0.00	98.65
18	−18	0	350.36	110.27
−16	16	−5	169.57	73.29
27	−29	−1	22.45	123.61
−16	13	−2	118.33	85.18
27	−33	0	0.00	129.82
17	−24	2	104.72	82.01
9	−18	3	23.90	66.43
−11	2	2	912.54	101.29
−15	4	2	189.64	85.31
28	−42	1	25.09	125.46
−35	18	−2	−84.52	149.76
−34	14	0	32.88	149.63
−29	8	2	0.53	103.01
26	−48	2	60.22	140.91
−37	14	0	78.18	133.78
−24	−1	4	−40.15	112.65
10	−37	5	9.51	79.76
−35	8	2	101.55	136.55
−13	−19	6	60.35	95.08

20	−11	−8	2.25	129.95
18	−9	−9	−77.65	119.78
18	−9	−6	131.14	95.08
9	0	−6	46.62	74.61
7	2	−7	124.93	77.52
24	−16	−8	112.38	119.78
26	−19	−6	116.35	142.89
21	−14	−4	328.30	108.16
27	−24	−3	161.77	117.93
−10	13	−5	45.16	50.05
−10	10	−2	4443.59	369.51
26	−27	−1	−9.77	108.55
29	−33	−1	−67.09	130.61
28	−35	0	−48.07	125.19
−23	14	−1	0.92	89.80
−27	18	−3	100.89	102.48
−13	3	2	222.13	71.97
−18	8	1	0.00	93.50
−32	19	−3	29.19	137.48
24	−38	2	60.09	111.33
−24	7	2	30.77	104.06
−33	16	−1	155.04	129.42
−30	11	1	−64.58	111.46
−17	−3	4	73.69	88.61
−32	8	2	81.48	152.40
−16	−9	5	−7.92	94.82
−32	4	3	−80.95	126.51
−30	−1	4	−87.29	121.89
21	−13	−5	49.39	102.88
2	6	−7	144.87	61.67
27	−20	−7	−83.99	124.40
1	6	−5	94.56	54.28
14	−13	0	170.36	74.09
−14	15	−5	3.96	64.45
−14	12	−2	104.33	74.48
−11	7	0	886.39	95.74
30	−35	−1	−20.21	125.85
−8	3	1	152.27	54.41
−15	9	0	124.67	73.29
24	−31	1	−0.26	120.18
−27	19	−4	41.20	102.35
−32	20	−4	0.00	132.72
7	−21	4	251.31	54.41
−19	2	3	163.36	88.35
−36	16	−1	11.62	138.93
22	−44	3	34.34	113.84
−10	−12	5	144.08	73.69
17	−41	4	0.00	93.24
−14	−10	5	126.91	85.71
−36	11	1	−49.13	133.65
−27	−1	4	−84.92	134.31
−18	−18	6	75.01	115.42
−21	−18	6	−169.70	129.29
14	−6	−4	−41.34	93.10

-12	14	-5	17.17	55.33
25	-25	-1	89.67	103.01
-12	11	-2	148.17	62.20
23	-25	0	-93.76	97.20
-18	16	-4	114.76	76.73
-18	15	-3	5.81	84.52
-13	8	0	586.22	81.48
31	-37	-1	65.63	134.57
23	-29	1	36.32	104.99
29	-37	0	66.16	119.52
25	-33	1	73.43	129.82
-16	7	1	352.34	88.74
13	-23	3	25.62	69.46
-35	21	-5	-109.48	125.72
-25	10	1	9.11	91.91
-9	-7	4	177.75	79.37
13	-30	4	0.00	80.29
21	-39	3	-56.79	105.78
14	-32	4	201.26	89.41
26	-45	2	-64.84	132.33
-33	11	1	1.32	138.00
-12	-11	5	7.00	76.07
-21	-8	5	104.72	129.55
-27	-8	5	77.78	125.59
-22	-19	6	-107.37	131.14
22	-13	-7	81.75	145.53
11	-3	-4	132.19	85.05
25	-19	-4	27.73	108.29
26	-22	-3	102.61	103.67
5	-4	0	762.12	79.37
29	-29	-2	-0.53	139.98
30	-31	-2	34.47	149.49
31	-33	-2	53.88	126.78
-23	19	-6	61.28	110.40
22	-27	1	117.27	95.61
26	-35	1	-12.68	129.55
-25	16	-2	0.00	92.44
-28	15	-1	88.48	100.50
-30	17	-2	-58.64	111.99
25	-40	2	-28.66	120.97
-35	19	-3	-44.64	141.31
-15	-4	4	150.42	82.93
-24	-8	5	0.00	122.55
-23	-20	6	56.39	143.68
21	-12	-9	0.00	124.27
14	-5	-5	49.65	84.52
21	-15	-3	75.01	92.31
18	-13	-2	231.64	102.35
31	-27	-5	15.72	127.70
22	-18	-2	0.00	98.12
-25	19	-5	-13.60	108.95
30	-39	0	-98.12	126.91
-7	-4	3	1741.75	157.94
-24	12	0	1.32	91.78

−35	20	−4	−33.54	126.65
12	−28	4	−12.28	71.97
−17	1	3	54.54	97.33
−29	13	0	−14.26	98.91
−11	−6	4	53.48	75.54
−13	−5	4	56.39	75.01
−24	3	3	157.94	101.29
−22	−2	4	179.34	100.50
−8	−23	6	0.00	80.95
−31	−2	4	104.46	128.63
−16	−19	6	44.50	108.29
23	−14	−8	93.10	135.76
21	−12	−6	−79.90	111.86
26	−18	−7	−24.17	128.76
25	−17	−9	116.87	130.61
25	−17	−6	75.14	141.97
14	−7	−3	1318.23	148.83
6	−1	−2	1492.69	133.51
32	−30	−4	−5.41	129.68
28	−27	−2	42.92	125.85
24	−23	−1	0.00	99.31
22	−23	0	77.78	89.80
16	−22	2	83.99	78.58
−10	4	1	1261.71	122.29
−14	6	1	223.05	71.31
−21	13	−1	138.93	107.63
27	−37	1	24.17	123.21
−2	−12	4	−0.79	48.33
−22	6	2	−52.82	100.89
−27	7	2	−45.16	115.55
−33	3	3	163.76	137.87
−28	−9	5	−0.40	136.15
−19	−19	6	96.67	125.33
16	−6	−8	−2.77	133.91
15	−5	−7	76.73	95.74
17	−8	−5	−0.40	88.74
30	−25	−5	0.00	136.15
31	−28	−4	57.18	139.72
−16	15	−4	55.20	71.45
16	−18	1	110.14	93.50
−16	14	−3	72.50	89.54
21	−25	1	261.08	98.52
−12	5	1	685.13	85.84
−30	20	−5	−122.02	139.72
22	−41	3	−10.83	115.42
−32	13	0	31.43	118.19
16	−36	4	−3.57	93.24
−35	13	0	155.83	152.93
−33	7	2	−2.38	138.14
−25	−2	4	−1.72	132.99
−19	−9	5	−52.30	131.53
−28	−2	4	−11.62	136.29
−14	−20	6	23.11	105.65
−29	−10	5	64.58	150.15

13	-3	-8	0.00	116.48
11	-2	-5	9.51	84.92
17	-10	-3	459.04	108.69
30	-24	-6	-38.96	144.74
25	-20	-3	14.39	102.61
30	-26	-4	-8.58	141.44
19	-16	-1	226.22	110.80
27	-25	-2	0.53	111.06
-25	17	-3	89.14	96.93
31	-41	0	-82.54	136.42
28	-39	1	0.00	122.95
-30	18	-3	181.32	117.14
-23	9	1	139.98	95.74
-15	0	3	19.41	99.05
26	-42	2	-50.71	134.04
-31	15	-1	43.05	125.19
-33	17	-2	77.39	146.19
-28	10	1	55.99	99.71
-36	17	-2	-90.33	139.59
23	-46	3	38.96	133.65
-30	7	2	130.87	120.04
-30	3	3	-24.43	129.82
11	-39	5	0.00	90.86
-5	-26	6	0.00	72.50
-10	-22	6	0.00	89.41
-12	-21	6	31.69	97.33
-25	-9	5	64.84	120.70
-32	-3	4	-10.17	132.59
-20	-20	6	40.41	129.55
19	-9	-8	-20.47	145.27
18	-8	-7	60.62	112.12
12	-2	-7	123.74	90.33
27	-19	-8	17.43	133.25
24	-16	-5	52.96	114.36
24	-17	-4	179.74	103.54
-1	8	-6	13.21	37.77
29	-23	-5	7.79	142.10
-8	10	-3	249.60	49.65
17	-16	0	12.41	93.63
-25	18	-4	79.76	96.14
-28	20	-6	46.22	131.40
-30	19	-4	-33.02	139.72
-9	-3	3	478.33	80.29
11	-26	4	46.09	66.29
27	-47	2	56.65	148.57
-37	15	-1	-48.07	143.81
-20	-3	4	119.78	98.39
-34	10	1	10.96	147.25
18	-43	4	32.75	115.42
-22	-9	5	52.96	121.23
-17	-20	6	-57.71	126.25
-21	-21	6	-17.04	135.89
-22	-22	6	79.10	157.68
20	-11	-5	96.27	104.99

26	−23	−2	72.50	102.88
4	−1	−1	876.09	86.90
−8	11	−4	11.49	51.77
−14	14	−4	563.50	89.14
−14	13	−3	104.99	77.65
−21	18	−6	2.64	94.03
−23	15	−2	173.40	92.97
−22	11	0	91.91	101.69
−26	14	−1	79.76	98.25
−11	−2	3	45.96	78.18
−13	−1	3	40.28	85.31
−34	15	−1	−19.15	151.21
−22	2	3	186.07	101.55
17	−38	4	177.62	103.40
−31	10	1	−26.54	120.84
25	−16	−7	55.60	137.08
24	−15	−9	−22.85	144.47
5	4	−8	14.00	81.35
29	−22	−6	92.84	135.10
29	−24	−4	60.48	142.63
31	−29	−3	101.29	150.81
23	−21	−1	−0.66	92.84
32	−31	−3	82.27	134.83
21	−21	0	103.01	93.24
−19	12	−1	350.23	108.95
22	−31	2	−63.39	115.95
23	−33	2	6.07	129.29
29	−41	1	110.54	141.17
−28	16	−2	3.43	103.67
19	−32	3	0.00	105.25
−33	20	−5	22.98	137.34
−20	5	2	40.81	103.67
−27	12	0	−32.88	106.18
−17	−10	5	87.95	126.12
21	−11	−7	163.10	144.08
17	−7	−6	382.84	111.99
14	−4	−6	368.32	99.05
24	−15	−6	0.00	141.31
8	0	−4	−26.68	73.82
21	−16	−2	17.83	92.44
12	−8	−1	184.62	69.86
30	−27	−3	−188.45	153.32
−10	11	−3	99.18	54.81
−12	12	−3	127.84	71.58
9	−10	1	51.24	46.75
−23	18	−5	80.16	95.35
21	−29	2	−53.22	94.16
12	−21	3	30.11	65.90
24	−35	2	−19.02	129.02
18	−30	3	183.04	99.05
20	−34	3	0.00	107.76
−33	18	−3	60.75	160.45
−36	18	−3	−7.13	140.38
−25	6	2	−5.55	115.42

23	−43	3	0.00	126.38
-5	−16	5	35.13	59.82
−34	6	2	0.00	137.34
−29	−3	4	54.67	140.25
−15	−21	6	−33.68	117.14
−26	−10	5	124.93	139.85
−18	−21	6	8.85	133.51
22	−12	−8	−98.52	140.91
20	−10	−9	−5.94	118.85
10	0	−8	296.87	109.74
26	−17	−8	52.82	132.99
6	3	−6	32.88	68.67
28	−21	−5	−38.43	148.04
20	−13	−3	312.59	101.42
24	−18	−3	−42.66	107.89
15	−11	−1	263.07	85.18
−10	12	−4	75.54	64.31
−12	13	−4	61.94	72.11
12	−13	1	348.38	72.37
−33	19	−4	74.61	146.85
21	−36	3	−12.81	113.70
−36	20	−5	0.00	133.78
27	−44	2	−16.24	141.17
−23	−3	4	16.24	116.48
−26	−3	4	55.33	143.29
−31	2	3	−64.45	144.21
−19	−22	6	77.26	142.36
20	−10	−6	186.07	109.21
29	−21	−7	−21.13	134.57
28	−20	−6	−64.45	141.44
28	−22	−4	21.00	139.32
14	−8	−2	141.83	75.41
29	−25	−3	111.86	145.66
25	−21	−2	92.44	106.04
31	−34	−1	26.02	133.51
27	−30	0	228.20	148.96
28	−32	0	0.00	145.27
29	−34	0	13.21	135.63
15	−20	2	78.97	83.07
17	−28	3	104.72	87.56
25	−37	2	−2.64	123.08
−21	8	1	94.56	96.01
−26	9	1	158.74	111.59
−36	19	−4	28.66	132.59
−28	6	2	0.00	107.23
−36	12	0	46.35	149.49
−31	6	2	49.65	145.66
−15	−11	5	−87.03	108.29
−20	−10	5	−107.76	132.19
−7	−25	6	0.00	90.20
−23	−10	5	17.17	128.36
−20	−23	6	55.73	158.87
1	3	−2	132.85	30.37
29	−30	−1	70.92	145.93

32	−36	−1	−9.77	137.74
30	−36	0	−66.29	135.76
−2	−4	2	168.51	34.20
20	−27	2	6.47	89.80
−23	16	−3	110.54	93.50
−28	19	−5	2.91	140.25
30	−43	1	−21.39	148.96
−29	14	−1	−60.09	104.59
−31	16	−2	−55.33	124.40
−30	12	0	107.10	110.40
−20	1	3	33.68	90.07
−33	12	0	215.00	152.93
18	−40	4	0.00	110.54
24	−48	3	−26.41	149.36
−35	9	1	−44.37	153.19
−30	−4	4	0.13	136.42
−13	−22	6	−26.94	113.70
−28	−12	5	−110.40	156.36
9	1	−7	106.31	84.52
23	−14	−5	−32.62	106.57
23	−15	−4	−65.63	107.37
3	4	−4	1323.25	122.68
17	−11	−2	262.80	105.38
22	−19	−1	155.96	97.59
−11	14	−7	98.65	57.84
28	−28	−1	−51.77	139.46
−17	11	−1	440.29	96.54
−21	14	−2	218.69	99.84
−20	10	0	286.57	105.52
−28	17	−3	162.83	108.42
−18	4	2	155.30	108.55
−37	16	−2	−58.37	143.15
9	−32	5	0.00	74.61
19	−45	4	0.00	133.25
12	−41	5	30.11	108.95
−4	−28	6	−48.60	77.26
−9	−24	6	147.25	103.01
−11	−23	6	47.41	107.89
−16	−22	6	0.00	132.06
24	−14	−7	0.00	152.79
23	−13	−9	221.73	142.49
11	−1	−6	88.35	86.10
8	1	−5	197.43	87.03
11	−5	−2	495.10	79.90
28	−23	−3	82.01	122.68
18	−14	−1	458.38	111.46
9	−5	−1	765.82	87.95
−9	13	−7	−4.09	44.50
13	−11	0	106.97	64.45
20	−19	0	0.00	101.55
−3	4	−1	742.98	69.99
19	−21	1	−66.16	89.01
−19	17	−6	30.24	85.31
31	−38	0	−84.65	139.46

−26	19	−6	0.00	141.57
−28	18	−4	0.00	113.70
−24	13	−1	140.78	98.52
26	−39	2	24.96	131.14
−32	14	−1	−5.15	127.84
−34	16	−2	121.23	157.68
−29	9	1	34.34	104.33
24	−45	3	18.36	138.27
−35	14	−1	−46.49	171.55
−7	−15	5	23.64	70.92
−32	9	1	140.78	152.53
10	−34	5	−0.66	79.76
−13	−12	5	19.94	97.59
−32	1	3	1.85	138.66
28	−19	−7	−8.45	137.87
27	−20	−4	0.00	124.40
31	−30	−2	99.97	159.00
27	−26	−1	21.00	123.87
32	−32	−2	39.35	140.25
33	−34	−2	24.83	135.89
25	−26	0	50.18	103.80
15	−16	1	194.92	87.82
25	−30	1	0.00	127.70
26	−32	1	0.00	141.97
19	−25	2	−66.29	91.25
27	−34	1	18.88	138.00
16	−26	3	185.41	84.65
10	−24	4	73.43	64.45
−25	11	0	0.92	105.38
28	−46	2	0.00	155.04
−23	5	2	24.30	103.93
−16	−5	4	39.49	93.10
−11	−13	5	0.00	84.52
−27	−4	4	11.09	136.68
−24	−11	5	126.38	134.31
−31	−5	4	124.93	147.91
−17	−23	6	−78.44	142.10
−18	−24	6	−95.61	155.17
25	−15	−8	−44.24	139.06
23	−13	−6	−115.16	127.70
27	−18	−6	−45.56	159.40
16	−7	−4	190.43	108.82
23	−16	−3	206.15	112.52
30	−28	−2	23.77	148.96
10	−8	0	11.62	51.24
32	−40	0	−35.92	152.27
28	−36	1	102.61	136.55
−26	15	−2	160.19	112.91
−19	7	1	363.17	111.06
23	−40	3	55.07	125.19
8	−30	5	64.84	68.41
−9	−14	5	11.49	76.33
−21	−4	4	−46.75	111.86
−18	−11	5	−63.13	137.61

−14	−23	6	8.06	128.23
18	−7	−8	99.84	151.47
16	−6	−5	59.43	92.18
4	5	−7	16.11	68.14
32	−26	−5	51.24	141.44
20	−14	−2	93.76	109.61
3	3	−3	641.82	67.35
24	−19	−2	107.76	103.27
16	−14	0	45.16	90.59
24	−28	1	167.59	109.35
11	−15	2	153.72	71.05
−21	17	−5	116.61	94.42
29	−38	1	−22.19	134.04
−31	19	−5	0.00	144.87
−16	3	2	93.37	94.95
−31	17	−3	−52.56	144.34
−18	0	3	246.82	94.95
−37	17	−3	0.00	153.19
19	−42	4	0.00	125.59
11	−36	5	−52.16	92.05
−32	5	2	68.67	161.77
−24	−4	4	139.59	148.44
−29	1	3	−68.94	154.25
−21	−11	5	1.98	128.23
−33	0	3	86.90	154.51
17	−6	−7	70.65	115.82
15	−4	−8	−35.00	135.36
19	−9	−5	13.47	90.86
13	−4	−4	140.64	96.54
27	−21	−3	55.20	118.33
32	−27	−4	133.51	151.47
29	−26	−2	142.36	144.34
26	−24	−1	48.20	118.06
24	−24	0	173.92	105.91
8	−12	2	81.22	49.13
−15	10	−1	222.52	80.43
−18	9	0	94.03	91.39
−24	8	1	−15.58	97.20
−34	17	−3	104.46	163.89
−14	−6	4	150.15	89.54
−26	5	2	74.88	103.54
26	−16	−9	51.77	153.85
21	−10	−8	135.76	149.10
20	−9	−7	5.81	137.08
26	−17	−5	134.31	154.51
31	−24	−5	−14.53	163.36
−34	19	−5	129.82	148.04
−37	19	−5	83.20	148.83
−31	18	−4	−93.24	166.40
−37	18	−4	−79.37	149.10
16	−8	−3	271.12	104.99
−21	15	−3	122.42	85.58
−19	13	−2	128.10	103.01
−28	11	0	−48.86	115.03

−34	11	0	−25.36	166.40
−29	5	2	−2.11	129.02
−4	−11	4	103.67	63.79
−28	−5	4	99.71	145.00
−26	−13	5	−191.22	165.87
−3	−30	6	35.52	80.56
−6	−27	6	−57.97	95.74
−12	−24	6	0.00	122.42
−15	−24	6	185.94	143.95
14	−3	−7	288.29	101.16
27	−17	−7	144.61	157.55
31	−23	−6	28.92	159.79
13	−3	−5	102.35	85.31
−26	18	−5	−7.66	120.84
26	−18	−4	−20.60	114.10
31	−25	−4	0.00	152.79
−21	16	−4	0.00	90.33
−34	18	−4	132.99	155.30
19	−11	−3	56.79	106.31
−29	15	−2	98.12	111.33
21	−17	−1	313.38	106.18
−27	13	−1	33.81	118.72
−31	11	0	−18.22	125.06
23	−26	1	93.24	102.88
30	−40	1	−0.66	152.13
−33	8	1	−32.88	170.89
28	−43	2	−46.88	158.74
8	−16	3	196.77	61.80
24	−42	3	12.55	136.02
25	−47	3	0.00	159.27
−16	−12	5	0.00	118.72
−27	−14	5	28.39	170.09
−16	−25	6	−1.32	151.34
26	−16	−6	−33.54	157.02
−29	19	−6	10.96	146.46
22	−12	−5	100.50	116.35
13	−5	−3	427.75	95.22
33	−30	−3	69.99	156.76
−26	16	−3	88.22	103.80
28	−24	−2	160.19	128.76
−35	15	−2	136.95	167.45
−36	13	−1	15.85	153.06
6	−7	1	291.85	50.84
−17	6	1	443.72	103.27
−27	8	1	32.35	114.36
18	−23	2	−0.26	87.29
−14	2	2	118.19	81.22
−21	4	2	135.89	93.37
15	−24	3	56.13	82.93
−16	−1	3	3.96	96.27
−30	0	3	0.00	149.49
15	−31	4	−13.87	95.22
17	−35	4	−30.90	106.84
−19	−5	4	28.53	98.39

−22	−12	5	−56.52	138.00
−8	−26	6	−9.64	104.06
−10	−25	6	84.39	116.74
24	−13	−8	−52.03	143.95
28	−18	−8	−89.14	159.13
7	3	−8	65.24	93.10
23	−12	−7	−0.13	159.40
19	−8	−6	2.25	111.06
−17	16	−6	19.81	81.88
30	−22	−5	0.00	155.96
32	−28	−3	60.22	156.89
−32	15	−2	16.64	161.11
25	−22	−1	−83.59	109.74
−13	9	−1	267.95	68.14
−30	13	−1	85.71	113.70
−33	13	−1	0.00	151.74
19	−17	0	124.93	106.31
−23	10	0	160.45	104.46
18	−19	1	27.20	104.06
31	−42	1	−105.65	162.83
−30	8	1	−35.39	124.14
−33	4	2	183.96	155.83
18	−37	4	102.35	116.74
−12	−7	4	339.13	86.90
−25	−5	4	−47.81	142.10
−29	−6	4	234.67	151.74
12	−38	5	65.24	103.80
−19	−12	5	0.00	138.93
−13	−25	6	−49.39	140.51
12	−1	−8	68.67	126.38
16	−5	−6	78.58	101.82
−24	18	−6	−10.56	141.04
3	5	−5	168.64	64.45
30	−23	−4	−7.13	160.32
−26	17	−4	0.40	105.12
26	−19	−3	156.10	120.44
23	−17	−2	−54.01	111.20
−24	14	−2	136.82	106.71
23	−22	0	35.39	102.35
11	−19	3	64.58	71.97
20	−44	4	0.00	147.51
−22	−5	4	29.32	127.04
22	−11	−6	7.00	120.44
30	−21	−6	173.92	154.64
−19	16	−5	68.28	91.78
22	−14	−3	−27.34	114.10
31	−26	−3	0.00	157.68
−16	8	0	461.42	95.74
22	−24	1	169.96	106.04
24	−32	2	25.09	148.44
25	−34	2	−24.70	144.21
29	−45	2	0.00	170.75
25	−44	3	−63.13	151.21
−27	0	3	−49.79	152.66

14	−29	4	0.26	90.07
−6	−10	4	106.04	70.39
−10	−8	4	175.51	78.58
−30	−7	4	−5.28	156.76
−2	−32	6	−56.92	83.59
−14	−26	6	64.18	153.19
−32	19	−6	−50.32	146.19
8	−2	−2	1480.93	134.97
27	−22	−2	−33.02	117.80
32	−33	−1	−21.92	150.02
33	−35	−1	−28.92	158.21
29	−31	0	49.39	155.70
30	−33	0	0.00	151.74
31	−35	0	15.98	149.89
−35	10	0	35.79	160.19
−22	7	1	55.47	103.01
−34	7	1	−58.77	172.74
23	−30	2	21.39	126.51
26	−36	2	−86.10	137.34
−12	1	2	248.01	73.69
−24	4	2	109.74	109.08
−30	4	2	67.09	161.91
−34	3	2	0.00	145.40
21	−33	3	48.99	115.03
22	−35	3	60.75	124.14
−31	−1	3	−10.17	144.34
−8	−9	4	79.63	71.58
19	−39	4	196.64	125.72
−14	−13	5	170.75	130.34
−11	−26	6	−89.14	133.65
25	−14	−9	13.87	164.02
26	−15	−7	3.57	146.85
8	2	−6	7.79	78.05
25	−15	−5	−111.86	124.40
−29	18	−5	56.39	158.74
25	−16	−4	89.27	108.95
10	−1	−4	42.39	85.05
−19	14	−3	43.45	89.27
−29	16	−3	23.37	128.50
−35	16	−3	0.00	168.91
−17	12	−2	19.94	92.44
17	−12	−1	424.97	102.88
14	−9	−1	−11.09	71.31
30	−29	−1	−51.11	161.64
−11	8	−1	177.36	55.60
32	−37	0	6.34	149.63
27	−38	2	22.98	145.66
−27	4	2	−10.17	120.31
20	−31	3	0.13	111.20
23	−37	3	111.72	131.00
−14	−2	3	139.85	99.18
−17	−6	4	14.53	95.35
−26	−6	4	−24.56	145.27
−2	−19	5	−5.55	52.69

-24	-14	5	-51.64	161.11
-25	-15	5	-143.81	178.41
-5	-29	6	0.00	100.10
-1	-34	6	57.71	73.03
27	-16	-8	-28.53	155.70
11	0	-7	434.22	107.23
30	-20	-7	105.52	163.49
13	-2	-6	195.71	95.22
29	-21	-4	6.07	156.23
30	-24	-3	46.09	160.06
-32	16	-3	-0.26	157.55
16	-9	-2	468.29	112.65
34	-33	-2	20.21	151.61
28	-29	0	0.26	143.29
33	-39	0	0.00	158.74
-26	10	0	7.00	112.38
-32	10	0	-139.59	145.93
-15	5	1	207.07	79.10
-4	-3	2	2070.85	175.77
13	-40	5	-73.03	120.31
-17	-13	5	48.47	139.59
-20	-13	5	119.12	140.78
-26	-16	5	-77.12	180.00
-9	-27	6	-43.84	126.12
25	-14	-6	16.90	154.38
29	-19	-6	36.05	162.44
-35	18	-5	-139.06	157.15
-19	15	-4	57.97	85.58
-29	17	-4	0.00	135.23
19	-12	-2	199.15	118.46
33	-31	-2	-2.77	172.60
-36	14	-2	-62.33	162.44
24	-20	-1	18.62	107.76
-20	11	-1	280.76	113.84
-25	12	-1	-10.56	112.91
-29	10	0	2.77	115.69
-31	7	1	-109.08	151.47
22	-28	2	-16.90	101.69
-10	0	2	5517.77	458.52
28	-40	2	35.52	160.59
-19	3	2	147.51	111.20
19	-29	3	106.97	102.48
-32	-2	3	39.88	153.45
-12	-14	5	0.00	109.61
-7	-28	6	68.01	113.04
-12	-27	6	112.78	149.49
20	-8	-8	135.49	153.72
1	7	-6	17.83	49.92
-15	15	-6	38.17	81.09
10	0	-5	153.19	90.07
-24	17	-5	0.40	106.44
-32	18	-5	-3.96	155.44
18	-8	-4	37.77	95.61
-32	17	-4	-59.03	170.49

−35	17	−4	57.71	167.45
25	−17	−3	132.19	114.63
−24	15	−3	36.45	101.95
32	−29	−2	−78.44	161.11
−27	14	−2	4.49	117.01
20	−15	−1	118.85	117.01
29	−27	−1	−72.11	148.04
−5	5	−1	646.83	72.63
−9	7	−1	1020.57	100.63
−34	12	−1	117.53	173.00
22	−20	0	132.33	100.76
7	−5	0	7.13	59.56
−14	7	0	1519.89	147.91
−21	9	0	272.31	116.87
−36	9	0	−47.41	159.13
14	−14	1	1018.06	117.80
28	−33	1	−21.00	153.06
29	−35	1	96.14	146.85
−25	7	1	36.32	107.10
−28	7	1	130.08	115.16
−35	6	1	−71.97	157.28
17	−21	2	72.63	84.92
30	−47	2	−99.31	206.02
24	−39	3	74.75	138.14
26	−46	3	−46.75	172.60
−28	−1	3	10.04	165.87
13	−27	4	136.82	83.07
20	−41	4	96.80	139.72
−20	−6	4	−49.79	113.97
−23	−6	4	−2.64	152.27
−27	−7	4	−43.18	152.40
23	−11	−8	147.51	156.23
17	−5	−8	87.16	164.42
19	−7	−7	−54.81	130.34
18	−7	−5	426.82	112.52
13	−6	−2	101.55	73.56
26	−20	−2	1.85	119.25
−33	14	−2	271.12	176.70
27	−31	1	−118.19	155.30
30	−37	1	−49.13	150.02
−6	−2	2	1488.59	132.19
−8	−1	2	2128.82	185.55
−31	3	2	59.82	164.68
14	−22	3	41.07	76.20
−12	−3	3	105.78	89.27
6	−19	4	116.08	42.00
−21	−14	5	19.55	147.64
22	−10	−7	106.31	166.79
6	4	−7	0.00	75.94
−27	18	−6	−48.86	169.17
21	−10	−5	259.90	113.04
28	−18	−5	201.79	170.09
−24	16	−4	23.51	104.86
29	−22	−3	206.02	152.40

22	−15	−2	−2.38	99.84
31	−27	−2	139.85	163.49
−22	13	−2	−95.35	103.14
−30	14	−2	72.11	124.40
−7	6	−1	295.55	52.82
−28	12	−1	−4.62	117.80
−31	12	−1	193.60	140.38
15	−12	0	333.06	85.05
11	−11	1	1204.00	119.91
31	−39	1	47.54	164.02
29	−42	2	0.00	176.17
25	−41	3	−0.40	146.06
9	−22	4	158.74	63.65
−10	−28	6	47.28	144.08
16	−4	−7	−49.26	116.08
29	−18	−7	−2.91	157.68
−22	17	−6	49.13	119.52
−17	15	−5	77.78	86.37
15	−5	−4	19.41	104.99
28	−19	−4	66.29	141.31
−15	11	−2	153.45	82.93
11	−6	−1	57.71	57.31
28	−25	−1	120.31	125.06
17	−17	1	401.99	117.40
26	−29	1	18.75	133.25
−13	4	1	338.87	79.76
−20	6	1	95.74	118.99
21	−26	2	127.44	100.76
5	−13	3	244.97	43.71
18	−27	3	48.73	90.33
−25	−1	3	94.29	134.57
−15	−7	4	136.68	98.52
−28	−8	4	155.30	162.30
−10	−15	5	95.61	99.84
−22	−15	5	−181.98	176.30
−4	−31	6	0.00	107.23
−11	−29	6	−203.24	162.96
28	−17	−6	24.56	165.60
24	−13	−5	130.87	118.99
5	3	−4	137.21	63.65
33	−26	−4	−28.39	157.28
18	−9	−3	32.62	108.95
−17	13	−3	199.41	97.46
−36	15	−3	−35.52	176.30
6	−2	−1	865.53	93.37
18	−15	0	116.08	106.57
26	−25	0	−3.30	112.52
−33	9	0	−110.40	174.32
32	−41	1	45.96	174.72
−22	3	2	62.46	104.20
−28	3	2	0.00	132.85
−29	−2	3	27.20	164.68
−24	−7	4	55.60	157.28
−29	−9	4	5.81	172.47

−4	−18	5	7.00	68.14
−15	−14	5	−76.60	146.98
−18	−14	5	−45.30	143.42
−23	−16	5	104.86	178.68
−25	−18	5	−57.58	206.68
−8	−29	6	40.94	134.97
26	−14	−8	76.73	162.04
14	−2	−8	0.00	148.96
9	2	−8	92.18	108.69
25	−13	−7	0.00	182.11
21	−9	−6	11.23	116.61
−36	18	−6	236.26	177.49
15	−4	−5	122.95	93.63
32	−23	−5	−94.29	162.17
21	−12	−3	0.00	97.99
−27	15	−3	26.68	124.27
30	−25	−2	251.05	166.93
23	−18	−1	−108.03	104.99
25	−27	1	6.34	112.65
−32	6	1	234.94	174.98
30	−44	2	−6.60	184.62
−17	2	2	98.52	104.06
−25	3	2	−11.89	117.53
−32	2	2	94.16	164.28
−10	−4	3	325.53	86.63
21	−43	4	−19.15	157.42
−8	−16	5	−28.26	88.48
14	−42	5	−37.77	123.74
−24	−17	5	−84.12	189.51
−6	−30	6	50.84	123.21
18	−6	−6	−67.22	108.42
32	−22	−6	1.85	163.76
−30	18	−6	0.00	157.68
−33	18	−6	113.31	156.23
15	−6	−3	86.63	105.38
34	−29	−3	−195.45	158.61
−35	11	−1	84.92	176.83
12	−9	0	523.09	78.58
−12	6	0	340.59	68.80
−24	9	0	−27.60	105.12
26	−43	3	150.55	162.17
27	−48	3	31.69	197.17
−6	−17	5	36.58	78.44
−9	−30	6	−19.02	150.15
24	−12	−6	17.30	151.47
−27	17	−5	64.97	146.19
−36	17	−5	89.54	167.98
32	−24	−4	−165.87	169.83
−17	14	−4	381.92	92.18
−36	16	−4	−62.99	163.23
28	−20	−3	−13.87	130.34
−30	15	−3	18.75	140.51
−33	15	−3	48.99	174.45
25	−18	−2	148.96	111.72

27	−23	−1	−40.41	115.95
−18	10	−1	23.37	94.16
−23	11	−1	103.27	101.16
−30	9	0	67.35	137.34
−23	6	1	7.53	102.08
−30	−3	3	164.28	166.13
−18	−7	4	13.34	104.72
−21	−7	4	228.60	143.55
−3	−33	6	−60.48	115.16
−27	16	−4	104.20	119.78
24	−15	−3	−34.34	127.04
33	−27	−3	8.19	173.40
34	−34	−1	−12.94	160.85
21	−18	0	117.93	104.99
25	−23	0	−26.28	114.10
−19	8	0	227.67	114.89
−27	9	0	110.14	116.35
20	−20	1	8.85	95.35
−11	3	1	301.10	64.18
−29	6	1	−42.39	126.38
20	−24	2	166.13	102.35
−26	−2	3	88.74	155.57
−13	−8	4	72.24	91.12
−25	−8	4	−11.49	161.77
−19	−15	5	0.00	149.49
−7	−31	6	50.45	145.14
28	−16	−7	0.00	162.83
−13	14	−6	−3.70	74.61
27	−16	−5	16.38	155.96
−30	17	−5	−180.92	176.17
−33	17	−5	164.68	164.81
27	−17	−4	−85.05	128.10
−33	16	−4	−0.40	170.62
5	2	−3	335.04	56.52
−22	14	−3	4.49	94.82
29	−23	−2	69.07	147.78
−25	13	−2	132.06	109.35
−32	11	−1	267.03	155.57
32	−34	0	36.58	155.30
33	−36	0	22.45	159.93
−34	8	0	77.39	171.94
24	−25	1	70.52	115.29
−26	6	1	9.38	115.82
−33	5	1	−156.23	189.24
27	−35	2	−23.64	149.49
31	−46	2	−122.95	206.68
−29	2	2	61.01	164.94
17	−25	3	28.92	91.25
−31	−4	3	−107.89	165.74
19	−36	4	−3.17	126.38
12	−35	5	107.89	101.69
−13	−15	5	42.66	140.25
−5	−32	6	−40.81	129.95
29	−17	−8	83.07	187.39

13	−1	−7	63.92	101.82
10	1	−6	133.65	86.90
5	4	−5	262.01	78.71
−22	16	−5	141.31	103.40
31	−22	−4	0.00	179.74
−30	16	−4	7.26	161.91
32	−30	−1	0.00	170.49
31	−32	0	−25.49	174.72
34	−38	0	42.39	175.77
−18	5	1	51.24	114.63
16	−19	2	104.20	98.25
5	−9	2	1273.20	110.54
26	−33	2	−157.15	159.27
28	−37	2	−107.50	157.81
27	−45	3	−158.34	182.90
−23	−2	3	−21.66	115.03
12	−25	4	−20.07	71.84
17	−32	4	−39.22	113.97
20	−38	4	0.00	132.85
22	−45	4	96.40	176.04
−26	−9	4	−126.91	161.38
11	−33	5	25.49	91.25
13	−37	5	−63.79	113.31
−16	−15	5	62.33	143.95
−24	−20	5	−15.32	214.73
22	−9	−8	−0.79	160.98
27	−15	−6	102.08	183.70
15	−3	−6	213.28	110.01
31	−20	−6	0.00	166.00
12	−2	−4	0.92	95.61
32	−25	−3	−23.11	170.75
35	−32	−2	−77.39	169.04
−20	12	−2	64.58	112.25
19	−13	−1	39.35	106.31
−26	11	−1	217.50	117.53
−29	11	−1	115.42	122.16
−36	10	−1	65.90	180.13
30	−30	0	−107.10	177.09
−10	5	0	29.71	55.60
25	−31	2	−60.48	155.44
29	−39	2	−83.33	166.00
−20	2	2	3.04	98.12
23	−34	3	53.35	136.29
24	−36	3	75.41	142.49
19	−6	−8	109.08	171.42
21	−8	−7	58.77	161.51
−25	17	−6	104.59	159.53
−15	14	−5	53.88	76.46
−22	15	−4	167.85	105.65
21	−13	−2	74.88	114.10
18	−10	−2	154.51	108.55
10	−3	−2	3708.54	313.51
16	−10	−1	121.36	92.18
26	−21	−1	−48.86	112.78

31	−28	−1	−28.66	169.83
10	−13	2	212.35	60.62
−15	1	2	183.83	103.40
−26	2	2	55.86	123.21
−27	−3	3	31.69	166.93
−22	−8	4	−16.90	162.96
−27	−10	4	−29.58	175.11
−21	−17	5	−85.18	181.32
−22	−18	5	49.26	185.94
−23	−19	5	−94.16	202.05
25	−12	−8	109.21	167.72
24	−11	−7	−36.85	172.21
−20	16	−6	72.90	103.80
20	−8	−5	0.00	119.78
12	−1	−5	0.00	87.95
20	−9	−4	108.16	103.27
27	−18	−3	30.77	125.72
−15	12	−3	97.99	91.91
34	−30	−2	27.86	169.57
30	−34	1	25.62	159.27
31	−36	1	−7.13	158.08
−9	2	1	1218.92	118.59
−30	5	1	0.00	147.51
−34	4	1	−148.04	172.21
24	−29	2	169.17	132.33
−23	2	2	56.13	115.69
−30	1	2	0.00	179.74
22	−32	3	35.13	128.50
16	−30	4	57.97	111.33
21	−40	4	−14.66	144.61
−11	−9	4	110.14	82.01
−16	−8	4	47.81	101.69
−28	−11	4	69.07	193.73
14	−39	5	−30.77	123.87
31	−19	−7	−137.61	178.02
8	3	−7	213.67	88.61
3	6	−6	22.85	59.43
23	−11	−5	15.05	126.25
30	−19	−5	−34.20	193.34
31	−23	−3	−78.05	175.11
−34	14	−3	0.13	177.75
28	−21	−2	41.60	127.84
3	2	−2	437.39	52.56
22	−16	−1	103.67	107.10
−16	9	−1	417.71	91.39
24	−21	0	135.36	119.52
−22	8	0	304.66	115.55
−31	8	0	36.32	141.70
−35	7	0	−6.60	170.62
29	−32	1	68.14	178.81
32	−38	1	0.00	168.77
13	−20	3	4.49	76.73
26	−40	3	−41.60	156.10
−19	−8	4	20.47	126.12

−11	−16	5	32.22	133.65
−17	−16	5	−21.00	149.49
18	−5	−7	−14.13	133.38
−34	17	−6	35.66	165.60
30	−20	−4	154.25	185.55
−25	14	−3	133.91	112.25
24	−16	−2	−55.86	113.04
15	−7	−2	121.50	86.76
33	−28	−2	−31.83	170.75
30	−26	−1	133.78	160.45
8	−8	1	100.23	47.54
33	−40	1	−45.83	184.23
19	−22	2	66.82	94.95
31	−43	2	63.13	198.49
10	−17	3	306.91	80.43
21	−30	3	160.85	120.97
28	−47	3	34.60	202.32
10	−31	5	44.50	76.99
28	−15	−8	0.00	177.89
16	−3	−8	51.11	161.11
11	1	−8	−2.38	122.55
27	−14	−7	71.84	167.32
30	−18	−6	82.27	172.34
−28	17	−6	−19.55	169.96
26	−14	−5	−20.21	141.44
26	−15	−4	67.35	123.48
17	−6	−4	28.39	110.27
−15	13	−4	68.41	83.99
20	−10	−3	0.53	102.61
−31	14	−3	29.71	157.15
−21	10	−1	374.66	126.51
−33	10	−1	203.24	174.06
−17	7	0	1126.88	138.66
−28	8	0	38.03	124.93
16	−15	1	120.70	92.71
28	−30	1	−59.43	167.72
−21	5	1	66.82	109.21
−27	5	1	106.57	120.84
−24	−3	3	−115.42	135.63
−28	−4	3	23.24	181.32
22	−42	4	−3.17	158.74
−23	−9	4	−8.06	163.76
−14	−16	5	127.44	147.64
−23	−22	5	225.16	236.65
23	−10	−6	90.07	142.76
−31	17	−6	−98.12	164.68
17	−5	−5	68.67	102.74
−25	16	−5	−116.61	122.55
−34	16	−5	73.56	167.59
−34	15	−4	9.77	174.85
23	−13	−3	100.37	116.35
−28	14	−3	−57.71	120.57
32	−26	−2	0.26	180.79
17	−13	0	375.58	109.74

28	−26	0	57.05	131.40
−8	4	0	294.36	62.99
−25	8	0	58.24	113.31
19	−18	1	−14.13	113.18
−24	5	1	−81.35	113.70
23	−27	2	8.32	107.50
27	−42	3	58.24	168.64
20	−7	−6	3.83	117.27
34	−25	−4	0.00	171.02
7	2	−4	84.25	69.46
−25	15	−4	0.00	108.03
−20	13	−3	55.99	92.97
25	−19	−1	−30.64	108.95
13	−7	−1	33.28	69.73
20	−16	0	17.17	117.27
−7	1	1	330.68	60.35
−16	4	1	458.52	96.67
−31	4	1	0.00	177.36
32	−45	2	0.00	208.52
−27	1	2	−90.46	132.06
16	−23	3	214.34	90.46
−21	−3	3	124.80	118.06
−29	−5	3	51.24	170.23
−9	−10	4	138.14	80.56
−24	−10	4	−16.38	162.83
15	−41	5	−61.67	143.55
−22	−21	5	208.26	212.09
26	−13	−6	38.69	170.75
−11	13	−6	103.27	68.41
17	−7	−3	292.78	112.25
30	−21	−3	60.88	167.06
−36	11	−2	0.00	174.85
29	−24	−1	191.36	133.51
−30	10	−1	217.64	147.51
9	−6	0	347.72	64.45
−32	7	0	0.00	181.72
13	−12	1	232.69	72.77
27	−28	1	124.14	140.38
−13	0	2	431.84	91.78
−18	1	2	131.93	115.16
−32	−1	2	−19.81	178.81
20	−28	3	−35.66	106.31
−30	−6	3	110.67	170.09
15	−28	4	−4.49	93.37
−9	−17	5	−96.14	113.31
−19	−18	5	171.81	173.40
−21	−20	5	48.60	200.86
30	−17	−7	4.36	179.87
−16	15	−7	30.37	99.05
29	−17	−5	227.14	198.09
−20	15	−5	131.14	98.39
−28	16	−5	85.18	165.21
−31	16	−5	85.71	176.43
−28	15	−4	111.99	133.51

−31	15	−4	216.45	196.37
26	−16	−3	221.33	138.93
35	−28	−3	66.69	176.30
−35	13	−3	0.00	188.05
27	−19	−2	176.43	121.76
−24	10	−1	360.53	124.53
28	−44	3	−14.00	187.53
29	−49	3	−26.68	225.82
23	−44	4	−47.54	183.30
−14	−9	4	244.71	105.12
−20	−9	4	109.61	157.94
−25	−11	4	178.02	172.08
−20	−19	5	7.26	186.21
15	−2	−7	36.85	116.61
12	0	−6	359.07	103.27
29	−18	−4	35.92	158.47
31	−24	−2	18.09	178.15
8	−3	−1	142.63	57.84
−27	10	−1	62.20	118.33
−34	9	−1	180.92	183.83
23	−19	0	220.67	112.52
14	−10	0	1094.92	118.99
27	−24	0	−12.02	116.74
34	−35	0	−20.21	170.36
35	−37	0	28.39	178.94
−21	1	2	119.65	103.54
−24	1	2	62.07	116.48
−25	−4	3	−12.02	171.55
−17	−9	4	72.63	109.74
−26	−12	4	−65.77	184.62
−27	−13	4	15.32	198.62
−15	−17	5	30.77	150.68
24	−10	−8	196.90	173.66
29	−16	−6	10.56	167.72
17	−4	−6	52.96	108.29
7	3	−5	368.98	90.73
33	−23	−4	94.69	179.87
−20	14	−4	170.49	98.52
34	−26	−3	−137.87	176.96
−13	11	−3	27.73	77.65
34	−31	−1	−49.52	175.38
33	−33	0	70.12	171.55
−6	3	0	−7.66	57.18
22	−21	1	48.86	99.97
−1	−2	1	486.38	45.96
−5	0	1	839.25	91.65
−32	3	1	−50.98	205.35
22	−25	2	36.98	106.71
29	−36	2	−46.35	164.94
−28	0	2	66.95	170.75
6	−26	5	68.14	33.54
−12	−17	5	−9.64	147.25
−22	−24	5	−145.40	256.59
27	−13	−8	6.74	176.43

21	−7	−8	126.51	173.53
23	−9	−7	24.83	176.70
−23	16	−6	8.45	135.23
−35	16	−6	83.33	187.00
−13	13	−5	43.18	73.82
14	−3	−4	276.67	111.46
7	1	−3	192.54	59.96
−14	8	−1	152.00	71.71
−20	7	0	103.93	114.36
−29	7	0	−35.00	130.08
−33	6	0	−51.77	191.22
26	−26	1	273.10	128.36
−28	4	1	111.06	130.48
28	−34	2	94.56	162.96
30	−38	2	−62.46	172.34
−7	−11	4	185.41	75.14
−7	−18	5	0.00	100.50
26	−12	−7	−30.24	171.81
10	2	−7	99.84	92.57
−18	15	−6	−7.79	98.25
−35	15	−5	−180.40	175.11
22	−10	−4	295.95	120.70
−35	14	−4	41.60	177.36
29	−19	−3	29.58	139.06
−32	13	−3	−59.69	192.41
23	−14	−2	130.48	104.20
20	−11	−2	150.68	125.19
−33	11	−2	48.60	177.09
21	−14	−1	537.88	132.33
28	−22	−1	−9.64	136.29
33	−29	−1	103.54	170.75
32	−31	0	0.26	168.64
−15	6	0	323.95	83.86
−19	4	1	30.64	113.18
27	−32	2	0.26	166.26
31	−40	2	0.40	192.54
29	−46	3	39.62	215.79
−26	−5	3	171.42	172.60
11	−23	4	106.71	71.31
20	−35	4	−62.73	135.23
21	−37	4	43.18	138.66
−21	−10	4	44.24	167.85
−21	−23	5	111.46	228.07
20	−6	−7	−0.40	154.91
5	5	−6	95.35	69.20
25	−12	−5	−15.98	135.89
22	−9	−5	−57.84	115.16
32	−20	−5	−27.20	177.89
14	−2	−5	151.08	94.29
−23	13	−3	14.79	106.04
−36	12	−3	−41.47	179.74
12	−4	−2	98.12	70.65
18	−11	−1	391.03	114.50
−19	9	−1	405.43	109.74

-31	9	-1	-132.19	149.10
-35	8	-1	56.13	186.07
-3	-1	1	679.98	70.39
-33	2	1	107.10	175.11
18	-20	2	3.04	89.14
19	-26	3	215.26	103.80
25	-35	3	-5.81	144.21
26	-37	3	118.06	157.28
-16	-4	3	0.00	82.67
-22	-4	3	17.17	123.87
22	-39	4	-14.53	149.76
24	-46	4	-74.61	203.37
18	-4	-8	62.33	170.23
13	0	-8	-48.60	152.53
29	-15	-7	58.24	181.72
-32	16	-6	-30.51	166.79
32	-21	-4	32.09	184.75
-13	12	-4	216.98	87.42
33	-24	-3	72.24	189.24
30	-22	-2	-74.35	146.46
35	-29	-2	0.00	168.64
24	-17	-1	63.39	113.44
26	-22	0	46.49	112.91
31	-29	0	0.00	173.00
-23	7	0	427.35	114.50
-26	7	0	278.52	121.36
32	-35	1	146.19	166.93
33	-37	1	124.93	175.77
34	-39	1	15.05	181.98
-14	3	1	1797.48	175.24
-25	4	1	214.20	122.82
32	-42	2	-454.42	216.98
-11	-1	2	279.31	76.07
27	-39	3	-4.75	164.94
19	-33	4	-11.62	129.29
1	-15	4	-28.53	22.45
1	-22	5	78.05	31.17
-17	-19	5	99.97	166.53
-20	-22	5	-92.18	203.37
32	-19	-6	-42.13	182.11
-26	16	-6	39.88	171.02
28	-15	-5	53.62	182.64
19	-7	-4	269.93	110.01
-26	13	-3	6.21	120.31
-29	13	-3	37.90	123.21
26	-17	-2	76.07	128.63
17	-8	-2	25.36	102.74
32	-27	-1	97.46	183.04
-34	5	0	274.03	185.28
31	-33	1	18.09	169.96
-22	4	1	0.00	99.97
26	-30	2	173.79	165.21
-16	0	2	494.96	113.70
-25	0	2	1.85	127.84

24	−33	3	−23.77	139.46
−19	−4	3	207.34	111.59
−27	−6	3	171.55	172.08
−5	−12	4	8.06	66.69
23	−41	4	−107.50	166.79
−12	−10	4	195.19	97.33
−22	−11	4	143.55	168.64
−5	−19	5	50.58	89.80
−18	−20	5	117.14	183.56
−19	−21	5	−6.87	194.00
25	−11	−6	0.13	171.94
22	−8	−6	105.12	146.85
−29	16	−6	37.90	170.75
19	−6	−5	108.16	109.74
−23	15	−5	−11.36	109.61
−32	15	−5	6.07	187.00
28	−16	−4	2.11	139.72
−23	14	−4	55.99	109.87
−32	14	−4	7.53	196.64
22	−11	−3	185.55	115.29
−4	2	0	405.96	42.92
−29	3	1	−8.19	144.47
12	−14	2	53.88	72.50
33	−44	2	−64.97	216.45
−30	−2	2	−124.67	179.34
28	−41	3	52.56	178.41
30	−48	3	85.44	237.84
−28	−7	3	51.11	176.30
14	−26	4	43.05	83.99
−18	−10	4	28.53	135.36
−13	−18	5	53.62	145.66
28	−14	−6	122.55	185.94
9	1	−4	100.23	80.56
25	−14	−3	16.11	121.10
−18	12	−3	13.87	107.10
34	−27	−2	145.66	189.64
5	1	−2	1455.58	129.29
−28	9	−1	55.33	133.65
−30	6	0	−26.41	139.06
18	−16	1	42.39	102.08
30	−31	1	19.81	180.26
21	−23	2	95.88	102.74
23	−31	3	−112.52	133.25
−29	−8	3	−144.34	185.28
8	−20	4	8.85	54.41
18	−31	4	55.86	117.14
−15	−10	4	−30.11	105.12
−23	−12	4	−82.14	165.21
14	−36	5	−32.75	114.89
15	−38	5	−58.24	125.99
−10	−18	5	65.90	143.55
−20	−25	5	61.28	244.18
−29	14	−4	−22.58	147.64
19	−8	−3	−1.45	112.78

32	−22	−3	5.55	187.66
−34	10	−2	−253.29	196.77
27	−20	−1	−12.02	124.40
−22	9	−1	245.63	114.76
22	−17	0	152.53	104.72
19	−14	0	63.13	105.91
21	−19	1	−7.79	93.90
10	−9	1	42.13	49.65
25	−28	2	−6.60	129.29
−19	0	2	99.44	103.80
−22	0	2	73.56	121.10
−31	−3	2	−71.05	182.90
15	−21	3	73.16	81.75
7	−14	3	1258.94	120.04
29	−43	3	−99.84	193.47
−23	−5	3	5.55	146.98
24	−43	4	−59.56	177.75
−24	−13	4	307.44	185.41
−25	−14	4	64.18	202.45
−3	−20	5	37.24	78.71
17	−3	−7	35.13	132.19
14	−1	−6	101.16	104.99
31	−18	−5	57.31	192.15
−18	14	−5	208.52	98.78
−26	15	−5	−18.88	137.34
−29	15	−5	185.94	176.43
−26	14	−4	47.54	115.29
28	−17	−3	55.86	125.59
−33	12	−3	−71.18	190.17
29	−20	−2	20.73	134.31
15	−8	−1	51.24	73.82
31	−25	−1	89.41	182.64
−25	9	−1	19.55	114.76
−32	8	−1	122.68	183.70
29	−29	1	−65.77	167.19
−3	−13	4	150.68	61.41
13	−34	5	−31.43	103.80
−1	−21	5	−5.94	65.77
16	−40	5	33.81	143.02
26	−11	−8	0.00	185.81
23	−8	−8	22.98	167.06
31	−17	−6	13.21	171.02
19	−5	−6	58.50	111.46
−9	12	−6	226.09	60.75
9	2	−5	5.28	86.37
31	−19	−4	4.09	185.81
−11	10	−3	87.56	67.22
33	−25	−2	245.24	185.41
11	−7	0	883.49	94.56
15	−13	1	303.21	86.76
−26	3	1	38.96	116.21
−30	2	1	66.95	175.64
7	−10	2	3392.65	282.35
−26	−1	2	16.77	147.91

18	−24	3	143.95	94.95
12	−18	3	40.28	82.80
−1	−14	4	51.90	51.11
−19	−11	4	−1.72	169.70
−19	−24	5	113.18	216.98
29	−14	−8	0.00	189.77
25	−10	−7	−69.07	173.92
12	1	−7	44.24	99.31
−14	14	−7	226.48	92.84
35	−24	−4	−19.68	184.09
−18	13	−4	79.10	87.56
10	−4	−1	1425.33	133.25
−12	7	−1	540.26	78.31
25	−20	0	0.66	115.29
16	−11	0	353.00	87.29
29	−25	0	31.30	140.64
36	−36	0	−107.50	196.77
−2	1	0	−5.68	6.21
−18	6	0	348.38	115.95
−27	6	0	71.58	134.31
−31	5	0	−171.68	178.15
22	−29	3	83.46	121.89
30	−45	3	9.38	216.58
−24	−6	3	52.43	168.77
28	−13	−7	4.75	169.96
7	4	−6	334.11	85.05
−21	15	−6	−31.17	115.82
−33	15	−6	19.68	186.73
16	−4	−4	144.74	101.03
−33	13	−4	−22.85	177.89
9	0	−3	121.89	66.95
−35	9	−2	0.00	180.13
35	−34	0	−41.07	174.45
−13	5	0	603.52	87.03
−17	3	1	838.98	126.65
−9	−2	2	1066.00	108.82
−14	−5	3	86.76	90.73
−20	−5	3	124.80	113.57
17	−29	4	15.58	107.23
25	−45	4	−53.48	195.45
−15	−20	5	155.17	156.76
−18	−23	5	0.00	199.68
15	−1	−8	41.73	165.34
22	−7	−7	116.87	174.19
27	−13	−5	63.26	161.91
24	−10	−5	29.32	124.14
16	−3	−5	3.17	100.23
−11	12	−5	8.32	64.97
24	−11	−4	−8.45	128.10
−30	12	−3	143.42	151.47
22	−12	−2	4.09	107.23
−31	10	−2	−50.58	168.91
−17	8	−1	59.56	87.56
−33	7	−1	212.75	206.02

34	−32	0	36.45	179.60
28	−27	1	−34.60	146.19
−12	2	1	812.57	98.12
−23	3	1	20.73	106.71
−31	1	1	−86.50	189.64
30	−35	2	154.12	167.19
31	−37	2	186.34	170.89
32	−39	2	53.48	189.90
−17	−5	3	82.27	106.31
−10	−11	4	263.46	92.57
17	−42	5	18.75	154.12
−8	−19	5	−55.33	135.36
−11	−19	5	25.22	139.85
−16	−21	5	0.00	174.85
−17	−22	5	51.24	186.21
20	−5	−8	55.86	171.55
−19	15	−7	−80.43	122.55
−16	14	−6	7.40	91.25
34	−21	−5	27.20	184.49
−33	14	−5	0.00	189.51
31	−20	−3	−9.51	164.94
−21	12	−3	31.43	96.01
−34	11	−3	0.00	201.53
25	−15	−2	11.23	115.55
14	−5	−2	−34.07	81.88
23	−15	−1	263.99	104.33
30	−23	−1	21.53	138.53
35	−30	−1	−111.20	181.19
−29	8	−1	24.70	132.59
−21	6	0	26.54	116.74
−24	6	0	77.52	110.14
−14	−1	2	45.56	96.40
−23	−1	2	−38.83	118.46
−25	−7	3	23.37	169.17
−16	−11	4	6.21	117.14
−20	−12	4	0.00	158.61
12	−32	5	7.79	96.67
31	−16	−7	−65.37	181.72
34	−22	−4	−14.79	187.39
35	−25	−3	−22.71	178.68
32	−23	−2	55.47	185.81
20	−12	−1	205.75	118.85
33	−30	0	−27.86	171.28
−32	4	0	0.00	192.94
−32	0	1	7.00	195.85
29	−33	2	53.22	165.60
31	−47	3	147.25	235.99
−25	−17	4	146.59	213.54
27	−12	−6	61.67	178.55
24	−9	−6	−23.24	161.11
−30	15	−6	−189.77	178.94
30	−16	−5	51.24	183.56
21	−7	−5	277.06	120.97
30	−17	−4	−4.75	174.98

-11	11	-4	339.93	80.82
-24	12	-3	30.64	117.93
-27	12	-3	19.68	115.82
28	-18	-2	169.70	131.14
19	-9	-2	210.64	121.36
26	-18	-1	83.86	118.06
28	-23	0	23.11	127.17
34	-36	1	0.00	175.77
35	-38	1	-106.84	187.53
-27	2	1	89.41	127.57
20	-21	2	24.17	96.01
34	-43	2	-106.18	211.69
-28	-3	2	-177.36	190.17
21	-27	3	43.71	105.78
27	-36	3	85.05	154.25
-26	-8	3	220.41	172.87
22	-36	4	16.64	137.48
23	-38	4	1.72	145.66
-13	-11	4	-17.96	99.57
-21	-13	4	105.25	164.02
-18	-26	5	64.58	227.14
30	-15	-6	131.14	175.11
-24	15	-6	71.84	149.89
-27	15	-6	69.20	179.87
11	0	-4	386.81	103.01
-30	13	-4	70.52	175.64
27	-15	-3	90.20	123.21
24	-12	-3	114.76	115.42
7	0	-2	8579.48	702.04
-28	10	-2	0.00	117.27
34	-28	-1	-30.90	173.26
-34	6	-1	74.22	175.77
-28	5	0	-22.58	128.23
33	-34	1	-13.34	167.19
28	-31	2	170.09	171.02
-20	-1	2	41.20	110.27
26	-34	3	0.26	148.57
29	-40	3	-77.65	180.53
-21	-6	3	-42.39	123.34
-27	-9	3	-7.66	169.17
-28	-10	3	-19.94	186.47
24	-40	4	3.17	154.91
-22	-14	4	-65.77	164.15
-23	-15	4	-134.17	176.83
19	-4	-7	0.00	149.49
16	-2	-6	-73.82	107.10
-34	14	-6	-51.77	191.09
-21	14	-5	31.30	104.99
-30	14	-5	174.06	185.55
-21	13	-4	-3.17	99.44
-34	12	-4	-8.72	174.85
-16	11	-3	-4.23	90.46
-35	10	-3	32.22	181.45
36	-28	-2	252.24	181.45

−32	9	−2	−47.28	178.68
−26	8	−1	16.38	113.70
−33	3	0	−19.41	177.62
27	−25	1	−52.82	138.53
32	−32	1	92.57	174.98
−17	−1	2	164.94	110.40
−29	−4	2	166.79	173.13
30	−42	3	1.32	194.53
13	−24	4	132.33	78.31
−17	−25	5	0.00	198.62
33	−19	−5	54.67	174.58
11	1	−5	−34.60	84.12
21	−9	−3	−88.48	102.08
34	−23	−3	33.94	183.43
29	−21	−1	27.20	124.27
−20	8	−1	329.36	118.72
−23	8	−1	76.20	107.10
21	−15	0	141.04	114.76
20	−17	1	20.34	107.23
23	−24	2	5.02	110.67
14	−15	2	78.44	87.56
−30	−5	2	47.41	171.68
25	−42	4	103.93	175.64
−17	−12	4	−21.79	144.61
28	−12	−8	0.00	181.32
25	−9	−8	−39.35	180.26
14	0	−7	328.30	117.80
21	−6	−6	32.88	126.78
9	3	−6	293.31	89.93
−27	14	−5	−59.30	151.08
−34	13	−5	−51.77	183.43
33	−20	−4	67.48	176.04
−24	13	−4	−34.07	113.04
−27	13	−4	18.49	123.21
30	−18	−3	13.60	153.06
−31	11	−3	52.96	187.66
31	−21	−2	12.28	166.13
−25	10	−2	0.00	112.25
17	−9	−1	117.40	94.69
33	−26	−1	185.81	196.37
−30	7	−1	129.02	141.31
24	−18	0	88.74	90.20
23	−20	1	106.84	107.10
−28	1	1	−33.15	160.98
27	−29	2	−76.46	157.28
−24	−2	2	−2.11	121.50
25	−32	3	43.58	135.23
16	−27	4	−13.87	92.71
−6	−20	5	64.84	119.65
−13	−21	5	−17.17	142.89
−16	−24	5	31.30	185.15
27	−11	−7	90.86	176.04
1	8	−7	103.54	54.67
−22	15	−7	232.96	164.55

33	−18	−6	−13.47	170.23
−24	14	−5	29.85	111.86
18	−5	−4	40.41	98.52
11	−1	−3	81.48	73.29
35	−26	−2	−15.58	169.70
13	−8	0	654.10	86.76
12	−10	1	719.60	89.41
31	−30	1	0.00	172.87
−24	2	1	43.32	113.84
−7	−3	2	3674.99	307.70
17	−22	3	193.60	92.57
31	−44	3	−135.76	215.00
−12	−6	3	35.92	102.88
−18	−6	3	89.54	107.76
−22	−7	3	−9.90	137.21
20	−32	4	129.68	125.85
−8	−12	4	57.84	82.27
−9	−20	5	−0.26	133.65
−14	−22	5	5.28	164.02
−15	−23	5	−86.10	174.45
−17	−28	5	15.98	240.09
17	−2	−8	−64.71	167.85
30	−14	−7	−61.14	180.00
18	−4	−5	232.56	111.86
−16	13	−5	−68.28	90.73
12	−5	−1	613.69	88.48
−10	6	−1	1424.28	131.93
27	−21	0	45.03	120.18
18	−12	0	333.45	108.29
31	−26	0	−51.77	174.98
−16	5	0	396.84	92.44
−25	5	0	76.20	115.69
−29	4	0	37.90	139.19
17	−14	1	624.65	113.70
−15	2	1	454.03	97.86
26	−44	4	−26.81	183.96
−24	−19	4	64.18	216.84
22	−6	−8	101.82	166.26
24	−8	−7	27.73	174.19
3	7	−7	269.80	70.39
29	−14	−5	−45.69	171.81
26	−11	−5	17.96	144.61
26	−12	−4	−58.77	122.29
−16	12	−4	73.29	89.14
−35	11	−4	0.00	172.47
−33	8	−2	−40.15	188.98
−11	4	0	854.57	94.82
−29	0	1	−127.84	182.38
9	−11	2	942.39	95.08
20	−25	3	30.77	95.74
14	−19	3	106.18	77.52
32	−46	3	0.00	228.73
−15	−6	3	14.39	93.50
−14	−12	4	17.43	107.89

−18	−13	4	−36.71	162.44
16	−37	5	−35.39	119.52
17	−39	5	−57.71	133.65
−31	14	−6	−122.68	167.59
−31	12	−4	−91.91	183.96
33	−21	−3	0.00	186.21
−9	9	−3	231.50	55.99
−28	11	−3	93.24	126.91
27	−16	−2	137.34	126.65
24	−13	−2	45.96	105.12
16	−6	−2	198.62	98.52
−29	9	−2	−36.58	142.76
25	−16	−1	74.48	111.99
32	−24	−1	215.13	173.66
−31	6	−1	120.04	178.55
30	−28	1	167.45	167.19
−10	1	1	82.14	55.07
−21	2	1	88.35	107.37
−12	−2	2	83.86	78.71
9	−15	3	71.31	65.37
−23	−8	3	−76.60	167.98
−11	−12	4	100.23	95.22
−23	−18	4	0.00	195.71
11	−30	5	0.00	85.18
−16	−27	5	7.40	205.35
−1	9	−7	22.32	40.15
23	−8	−5	94.82	120.97
−31	13	−5	−10.56	179.07
23	−9	−4	128.89	115.95
13	−1	−4	205.88	107.89
−32	10	−3	−49.39	183.30
34	−24	−2	52.16	180.92
22	−13	−1	69.99	111.86
37	−31	−1	−130.21	192.81
−15	7	−1	23.64	74.61
−27	7	−1	231.24	126.91
36	−33	0	32.88	171.94
−19	5	0	240.62	117.14
−22	5	0	194.92	103.67
−18	2	1	116.48	115.69
−30	−1	1	−112.38	180.79
26	−27	2	−66.69	128.23
32	−36	2	67.48	164.28
33	−38	2	79.37	179.74
34	−40	2	65.63	195.85
−21	−2	2	127.84	113.84
10	−21	4	125.33	64.58
27	−46	4	285.52	202.32
−19	−14	4	33.68	157.94
15	−35	5	−54.81	108.69
18	−41	5	93.76	146.72
−12	13	−7	6.74	76.07
29	−13	−6	62.07	166.93
26	−10	−6	82.27	166.93

−19	14	−6	50.32	97.99
32	−17	−5	−159.13	176.30
32	−18	−4	−6.74	177.36
−19	11	−3	78.84	104.33
30	−19	−2	72.77	129.16
21	−10	−2	166.00	115.16
9	−1	−2	2051.96	181.19
−34	7	−2	96.40	184.75
28	−19	−1	82.54	124.14
−30	3	0	100.23	161.64
−25	1	1	0.00	120.04
31	−34	2	114.50	162.70
35	−42	2	159.27	203.77
−26	−4	2	−14.13	164.81
−24	−9	3	−61.28	172.60
−27	−12	3	0.00	181.72
19	−30	4	3.04	113.44
−20	−15	4	−150.15	162.44
−21	−16	4	−90.46	168.64
21	−5	−7	42.13	163.10
5	6	−7	82.41	74.75
32	−16	−6	85.44	173.53
18	−3	−6	27.47	107.37
11	2	−6	147.12	92.18
13	0	−5	47.41	87.03
29	−16	−3	107.23	131.27
26	−13	−3	−42.00	119.65
36	−29	−1	88.22	167.72
−32	5	−1	−78.71	184.49
30	−24	0	10.30	133.12
35	−31	0	217.11	169.30
4	−2	0	443.20	45.69
36	−37	1	0.00	187.92
−31	−2	1	139.46	170.23
22	−22	2	114.10	104.06
−19	−7	3	238.24	113.97
−25	−10	3	−99.97	158.87
−26	−11	3	52.82	174.32
−4	−21	5	0.00	101.69
−15	−26	5	−52.82	185.81
16	−1	−7	4.75	119.25
−17	14	−7	64.84	106.97
−14	13	−6	18.88	83.86
−28	14	−6	250.12	175.77
23	−10	−3	76.20	114.10
−22	11	−3	14.92	110.54
−25	11	−3	1.85	111.86
−26	4	0	46.88	121.76
−31	2	0	0.26	182.38
35	−35	1	−192.15	170.36
30	−32	2	0.00	160.59
−15	−2	2	177.36	104.33
−18	−2	2	61.80	95.35
−27	−5	2	−29.19	167.19

23	−28	3	−149.63	121.63
29	−37	3	32.88	161.91
30	−39	3	−94.16	177.89
30	−13	−8	71.18	187.53
27	−10	−8	−122.55	179.47
23	−7	−6	1.58	144.34
−7	11	−6	158.61	50.58
−9	11	−5	14.92	62.86
−28	12	−4	−31.69	135.89
36	−24	−3	−89.41	160.06
13	−2	−3	54.81	90.33
−26	9	−2	0.26	115.82
19	−10	−1	352.60	107.10
−24	7	−1	114.36	108.29
29	−26	1	299.78	153.85
−28	−6	2	−57.31	164.68
28	−35	3	−47.67	149.49
31	−41	3	−29.05	188.98
24	−37	4	140.38	140.38
25	−39	4	25.75	148.57
−6	−13	4	110.67	69.46
−15	−13	4	38.30	116.21
−23	−21	4	−156.10	217.37
8	−27	5	−10.96	54.67
−7	−21	5	105.65	128.50
−11	−22	5	120.57	133.12
−14	−25	5	10.17	171.55
−15	−29	5	−6.74	215.66
19	−3	−8	25.75	159.00
29	−12	−7	156.62	171.81
−22	14	−6	60.88	117.14
−25	14	−6	−11.09	159.00
−32	13	−6	−1.85	161.38
−28	13	−5	−96.80	160.72
35	−21	−4	290.27	168.51
20	−6	−4	43.32	103.40
−32	11	−4	7.40	188.58
32	−19	−3	−84.25	188.05
−33	9	−3	8.98	178.15
33	−22	−2	−176.70	176.04
−30	8	−2	5.15	137.87
31	−22	−1	−31.03	143.42
−18	7	−1	486.64	112.65
−33	4	−1	−17.96	182.90
26	−19	0	113.57	122.68
23	−16	0	203.11	105.65
−32	1	0	−110.67	172.34
22	−18	1	82.01	97.06
34	−33	1	−302.02	163.23
25	−25	2	0.00	113.57
16	−16	2	294.89	101.16
−22	−3	2	−23.90	113.57
−29	−7	2	−10.96	162.70
32	−43	3	−63.92	192.94

26	−41	4	−42.79	162.96
14	−33	5	−14.26	100.37
−12	−23	5	−100.10	143.15
−13	−24	5	26.41	160.19
32	−15	−7	0.00	182.38
7	5	−7	43.84	81.48
−29	14	−7	−11.62	163.76
20	−5	−5	88.48	111.20
−19	13	−5	87.95	97.86
−32	12	−5	−48.60	180.00
−19	12	−4	245.90	93.50
−14	10	−3	215.52	84.78
−29	10	−3	49.52	136.55
35	−27	−1	0.00	162.30
14	−6	−1	246.95	75.67
−21	7	−1	524.81	119.12
−28	6	−1	11.75	116.21
15	−9	0	285.38	85.44
25	−21	1	246.56	115.29
14	−11	1	1490.04	144.21
−26	0	1	194.53	121.76
27	−33	3	24.30	144.08
−10	−7	3	440.69	100.89
−16	−7	3	304.93	108.42
−20	−8	3	0.00	120.57
15	−25	4	59.03	84.92
24	−7	−8	−4.36	159.27
26	−9	−7	25.75	167.06
28	−12	−5	48.73	159.40
−9	10	−4	−16.77	62.73
−25	12	−4	−37.51	110.67
37	−27	−2	−36.05	168.91
−20	9	−2	70.52	120.04
−23	9	−2	21.79	98.12
20	−13	0	−10.43	107.23
19	−15	1	56.79	108.82
33	−31	1	−75.80	161.51
−22	1	1	17.96	100.37
29	−30	2	2.91	156.76
−5	−4	2	1467.07	129.95
19	−23	3	199.15	97.99
27	−43	4	−98.25	169.96
−22	−20	4	23.37	196.64
31	−15	−5	152.13	177.23
−25	13	−5	0.00	120.31
28	−13	−4	−2.91	132.19
15	−2	−4	63.92	99.31
−22	12	−4	184.36	106.97
26	−14	−2	100.89	118.59
18	−7	−2	297.27	108.16
11	−2	−2	1013.17	108.82
29	−22	0	0.00	122.95
33	−27	0	−7.79	176.43
6	−3	0	25.09	60.62

-23	4	0	180.40	111.99
-27	3	0	3.30	128.76
11	-12	2	204.43	64.58
33	-45	3	-115.03	211.30
-13	-7	3	124.80	84.65
18	-28	4	116.08	101.55
22	-33	4	16.38	124.53
-12	-13	4	0.00	91.39
-16	-14	4	113.04	153.19
-14	-28	5	13.60	188.19
13	1	-6	99.18	95.08
25	-9	-5	-34.47	130.48
15	-1	-5	22.58	93.24
-22	13	-5	-17.43	103.54
25	-10	-4	13.07	124.14
-33	10	-4	-60.22	167.32
35	-22	-3	117.53	168.51
29	-17	-2	16.51	127.04
-31	7	-2	-19.41	167.06
27	-17	-1	-2.77	114.63
-14	4	0	1131.50	122.82
-13	1	1	422.60	81.61
-27	-1	1	-139.98	149.76
22	-26	3	233.62	105.52
16	-20	3	35.26	76.60
-21	-9	3	-87.56	146.19
28	-45	4	26.54	174.58
-9	-13	4	271.25	92.05
-21	-19	4	-23.11	186.07
23	-6	-7	-21.53	162.83
-20	14	-7	28.79	125.46
-26	14	-7	86.37	162.17
31	-14	-6	40.01	159.66
28	-11	-6	43.98	163.76
20	-4	-6	-105.65	114.10
34	-18	-5	5.02	156.23
24	-14	-1	150.02	106.71
34	-25	-1	0.92	167.45
-29	5	-1	-20.73	129.29
32	-29	1	146.06	169.04
-19	1	1	8.72	109.35
26	-31	3	1.85	133.38
11	-16	3	31.83	73.69
-26	-14	3	231.24	178.94
-17	-15	4	-63.26	154.12
-20	-18	4	187.79	166.00
-22	-23	4	11.09	210.24
-2	-22	5	0.00	86.37
18	-2	-7	21.79	122.95
9	4	-7	67.09	85.84
-29	13	-6	154.25	161.38
-33	12	-6	25.36	161.64
-33	11	-5	0.00	164.02
34	-19	-4	64.31	171.55

−29	11	−4	−28.66	148.70
28	−14	−3	18.49	120.31
32	−20	−2	28.53	157.15
23	−11	−2	114.50	95.61
36	−25	−2	20.73	156.36
−27	8	−2	105.91	116.35
30	−20	−1	−34.07	120.18
−9	3	0	486.91	70.92
−20	4	0	−29.32	117.93
−8	0	1	162.44	55.07
−28	−2	1	−122.02	163.62
28	−28	2	107.23	149.36
34	−37	2	63.13	169.96
35	−39	2	−46.49	174.32
−10	−3	2	396.84	74.35
−19	−3	2	172.34	101.42
−22	−10	3	62.33	162.17
−25	−13	3	23.51	162.70
−18	−16	4	80.82	148.70
−19	−17	4	−116.21	148.96
−13	−27	5	−28.79	164.68
−23	14	−7	−130.34	162.30
25	−8	−6	0.00	170.89
−14	12	−5	47.54	74.88
31	−17	−3	38.30	174.98
15	−3	−3	−37.77	93.37
−26	10	−3	146.72	117.53
−30	9	−3	−104.86	152.53
−8	5	−1	68.14	50.32
−25	6	−1	−33.54	113.04
32	−25	0	−30.37	157.42
37	−32	0	65.37	168.64
−17	4	0	244.31	100.50
−28	2	0	13.21	133.12
−16	1	1	259.63	101.03
21	−20	2	112.52	101.42
−24	−5	2	−57.58	130.87
−23	−11	3	−8.19	154.38
−24	−12	3	101.03	163.23
12	−22	4	277.72	74.75
18	−38	5	−36.18	122.55
19	−40	5	136.82	132.99
29	−11	−8	−62.46	170.89
−29	12	−5	0.13	164.15
−14	11	−4	94.56	89.93
−34	9	−4	136.95	169.17
25	−11	−3	54.94	116.87
−32	6	−2	−7.13	172.08
21	−11	−1	312.06	119.38
−13	6	−1	29.71	62.60
8	−4	0	355.90	64.58
5	−5	1	693.32	75.27
3	−4	1	583.18	59.16
−23	0	1	58.50	105.91

−29	−3	1	34.60	167.32
24	−23	2	36.45	101.29
33	−35	2	−83.86	156.36
−17	−8	3	18.62	101.95
21	−31	4	−95.48	117.01
−5	−22	5	116.74	117.01
−9	−23	5	−44.11	119.91
−12	−26	5	−49.65	148.96
−13	−30	5	−50.84	182.11
31	−13	−7	32.62	157.15
−30	13	−7	−63.52	152.93
22	−6	−5	62.20	109.74
22	−7	−4	43.05	105.52
16	−7	−1	349.04	95.88
−30	4	−1	0.13	153.59
25	−17	0	27.73	99.84
31	−27	1	94.56	161.51
37	−36	1	−68.80	175.11
32	−33	2	42.79	147.64
−16	−3	2	93.63	108.29
−25	−6	2	−7.26	155.17
25	−29	3	−121.76	122.42
−13	−14	4	−55.33	105.65
−21	−22	4	−112.91	186.73
13	−31	5	3.43	83.20
17	−36	5	−41.07	107.37
20	−42	5	82.80	140.64
−10	−24	5	0.00	126.65
−11	−25	5	121.50	137.87
26	−8	−8	6.74	149.23
28	−10	−7	73.03	156.89
15	0	−6	132.72	99.57
−17	13	−6	0.00	88.61
34	−20	−3	−34.34	166.13
−17	10	−3	254.88	108.82
−23	10	−3	−64.71	102.88
35	−23	−2	114.10	158.08
13	−3	−2	252.76	78.84
−33	5	−2	−70.92	174.06
33	−23	−1	0.00	159.27
28	−20	0	91.52	122.68
17	−10	0	734.39	111.46
36	−30	0	0.00	157.02
−24	3	0	99.44	115.29
−29	1	0	41.34	148.04
24	−19	1	38.17	103.40
16	−12	1	459.97	94.16
36	−34	1	−53.09	154.25
−13	−3	2	219.62	93.10
−26	−7	2	−47.54	155.57
−28	−9	2	59.96	155.30
32	−40	3	−1.06	167.98
33	−42	3	−19.41	175.24
−4	−14	4	200.73	62.07

11	3	−7	266.76	95.35
−26	13	−6	−52.16	155.57
30	−13	−5	0.00	163.36
17	−3	−4	302.42	97.73
−26	11	−4	1.19	108.55
−24	8	−2	−27.86	114.36
37	−28	−1	−77.65	160.32
−22	6	−1	5.55	105.78
22	−14	0	299.51	115.42
27	−22	1	140.78	112.65
27	−26	2	−23.51	129.42
−20	−4	2	17.70	113.18
−27	−8	2	−55.73	161.64
30	−36	3	0.00	141.44
26	−38	4	−48.86	136.82
27	−40	4	75.94	145.40
33	−16	−5	−32.75	158.87
17	−2	−5	32.22	99.31
30	−14	−4	25.09	165.74
−30	10	−4	−127.44	168.77
−7	8	−3	123.61	45.83
−20	10	−3	18.62	91.25
20	−8	−2	−82.27	114.50
−28	7	−2	393.28	123.34
−31	3	−1	−135.89	171.42
−30	0	0	0.00	162.83
21	−16	1	−11.75	108.16
35	−32	1	25.49	153.98
7	−6	1	285.78	53.88
−24	−1	1	2.25	117.27
31	−31	2	77.92	143.29
13	−13	2	405.96	79.90
29	−34	3	−129.55	140.91
34	−44	3	3.43	184.62
−18	−9	3	42.52	103.14
17	−26	4	148.30	86.63
25	−36	4	67.75	131.00
28	−42	4	81.35	152.93
−20	−21	4	69.07	173.13
−12	−29	5	28.13	156.76
20	−3	−7	0.00	150.68
22	−5	−6	78.58	134.04
−30	12	−6	116.21	152.93
27	−10	−5	3.43	143.95
−26	12	−5	140.78	133.38
27	−11	−4	21.13	128.10
31	−18	−2	1.19	129.82
28	−15	−2	137.74	114.50
−15	8	−2	50.84	79.24
−26	5	−1	99.57	111.59
−32	2	−1	−0.26	158.08
31	−23	0	69.46	135.76
−31	−1	0	0.00	154.51
−20	0	1	207.47	101.29

21	−24	3	171.42	92.31
−8	−8	3	187.00	81.22
−14	−8	3	38.30	88.74
−14	−15	4	0.00	112.25
16	−34	5	−69.20	99.05
25	−7	−7	1.06	149.49
33	−15	−6	−16.90	150.55
30	−12	−6	−76.73	161.38
−20	13	−6	−41.34	98.91
−23	13	−6	0.00	125.19
−30	11	−5	−40.54	168.38
−27	9	−3	0.00	115.55
25	−12	−2	66.69	110.27
29	−18	−1	107.89	113.70
26	−15	−1	151.87	110.14
−16	6	−1	91.52	81.35
−19	6	−1	80.29	112.52
10	−5	0	71.31	56.39
30	−25	1	−6.34	134.57
18	−21	3	282.21	89.27
13	−17	3	168.11	83.46
−25	−16	3	−45.56	166.00
20	−29	4	80.56	106.18
29	−44	4	−42.66	158.87
−10	−14	4	170.36	88.35
10	−28	5	−17.04	69.73
0	−23	5	93.63	70.92
−15	13	−7	25.36	89.14
−27	13	−7	−175.77	149.36
−31	12	−7	−33.68	153.06
−17	11	−4	56.13	83.07
−23	11	−4	64.97	104.99
30	−15	−3	5.02	138.14
36	−26	−1	26.94	151.61
−21	3	0	10.17	106.57
−25	2	0	−12.68	110.54
34	−30	1	−6.87	146.19
−25	−2	1	74.48	115.55
24	−27	3	−4.23	104.06
28	−32	3	57.05	138.93
−11	−8	3	0.00	86.10
−19	−10	3	69.33	118.85
−7	−14	4	244.58	81.35
−15	−16	4	14.53	140.91
−20	−24	4	−1.19	178.02
−11	−28	5	56.52	145.53
31	−12	−8	−95.08	165.34
13	2	−7	140.12	98.65
−10	12	−7	88.08	64.84
27	−9	−6	−60.48	155.70
−12	12	−6	268.35	79.37
−17	12	−5	103.14	88.61
36	−20	−4	−6.74	146.19
6	4	−4	217.37	70.26

33	−18	−3	23.90	158.21
27	−12	−3	123.74	110.27
34	−21	−2	5.94	164.42
−18	8	−2	73.43	106.04
−29	6	−2	−100.10	125.46
32	−21	−1	31.83	146.46
5	0	−1	772.82	85.71
9	−7	1	976.99	97.73
30	−29	2	−116.48	146.19
−24	−15	3	−12.41	155.57
30	−46	4	0.00	165.34
−18	−19	4	−15.98	139.19
17	−1	−6	−90.07	97.46
24	−7	−5	150.15	117.67
−23	12	−5	−60.22	101.55
24	−8	−4	−25.62	108.03
4	5	−4	475.16	70.78
−20	11	−4	119.38	96.40
−31	9	−4	−13.87	175.77
−12	9	−3	297.01	77.65
15	−4	−2	100.63	88.48
23	−12	−1	282.35	110.01
18	−8	−1	121.50	98.39
7	−1	−1	260.42	57.71
−27	4	−1	38.43	111.72
34	−26	0	53.09	164.81
−11	0	1	1482.38	140.38
26	−24	2	55.86	102.88
23	−21	2	34.60	99.71
36	−38	2	−27.60	155.83
−17	−4	2	246.56	99.71
−20	−11	3	−9.90	139.59
−23	−14	3	53.35	141.83
14	−23	4	67.62	75.01
−16	−17	4	59.16	137.34
−17	−18	4	16.77	138.66
−11	−31	5	79.63	159.79
19	−3	−5	211.30	103.01
−20	12	−5	13.73	95.61
19	−4	−4	82.01	94.16
8	3	−4	54.67	77.52
−33	6	−3	59.56	158.34
27	−18	0	4.89	106.44
19	−11	0	200.60	103.40
−12	3	0	430.25	73.29
18	−13	1	75.94	99.44
−17	0	1	324.87	106.71
−26	−3	1	−28.79	127.70
35	−36	2	11.36	151.87
−21	−12	3	−63.26	146.06
−22	−13	3	−14.39	139.46
23	−32	4	31.56	116.61
−3	−23	5	0.00	95.74
−7	−24	5	66.29	107.10

−10	−27	5	1.58	128.76
30	−11	−7	−12.55	150.15
−24	13	−7	−20.07	153.45
−31	11	−6	105.78	151.34
6	5	−5	7.92	68.41
−31	10	−5	−152.93	153.45
−27	10	−4	3.83	116.74
36	−21	−3	−1.19	148.83
22	−9	−2	39.35	98.39
−25	7	−2	1.98	110.67
35	−24	−1	7.92	150.15
−23	5	−1	31.69	104.06
30	−21	0	0.66	115.95
12	−6	0	333.59	67.88
−18	3	0	8.98	100.89
−26	1	0	28.13	106.31
26	−20	1	25.22	104.06
33	−28	1	−47.54	152.93
−27	−4	1	0.00	159.53
15	−14	2	96.01	85.05
−3	−5	2	777.18	74.22
−22	−6	2	−40.01	108.42
27	−30	3	70.65	142.23
−15	−9	3	50.05	95.61
−19	−23	4	−5.81	159.40
20	−39	5	−71.45	113.97
21	−41	5	−48.60	122.95
−8	−25	5	83.20	114.89
−9	−26	5	60.88	122.55
22	−4	−7	27.86	149.23
−32	11	−7	130.48	149.36
−27	12	−6	−157.81	159.27
32	−14	−5	0.00	155.04
8	4	−5	244.05	73.82
−32	8	−4	0.00	144.87
−24	9	−3	21.26	108.55
37	−24	−2	0.00	144.74
−30	5	−2	−45.43	141.97
9	−2	−1	1125.16	110.80
24	−15	0	0.00	107.37
38	−31	0	90.86	147.25
23	−17	1	−4.89	102.08
−14	0	1	1614.45	160.72
−21	−1	1	118.46	102.61
−29	−6	1	91.52	146.46
34	−34	2	18.09	139.85
−8	−4	2	136.02	60.22
−27	−11	2	−37.64	139.59
−11	−15	4	−22.58	88.74
15	−32	5	29.05	83.07
27	−8	−7	84.92	141.31
15	1	−7	5.15	108.29
−18	13	−7	100.37	102.35
24	−6	−6	94.69	138.80

35	−17	−5	−19.55	155.04
29	−11	−5	24.70	149.76
4	6	−5	125.19	65.50
−27	11	−5	−108.16	140.78
32	−15	−4	178.81	160.72
29	−12	−4	−46.75	136.68
10	2	−4	5.55	81.22
30	−16	−2	3.96	114.23
3	1	−1	825.91	80.95
−28	3	−1	71.05	123.48
−15	3	0	358.94	85.84
11	−8	1	363.70	63.52
29	−27	2	48.33	141.04
−23	−7	2	−42.39	123.61
−26	−10	2	−71.31	136.42
32	−37	3	137.61	143.42
33	−39	3	−0.40	148.30
34	−41	3	17.04	154.64
35	−43	3	0.00	161.77
−19	−26	4	−108.03	169.96
19	−37	5	49.39	105.12
22	−43	5	−79.24	127.44
−21	13	−7	110.27	137.48
32	−13	−6	−13.07	137.34
33	−19	−2	57.71	151.21
−31	4	−2	−99.84	155.96
33	−24	0	−92.05	148.17
−22	2	0	33.68	100.63
−27	0	0	−71.58	118.99
33	−32	2	35.00	133.38
−14	−4	2	146.46	100.10
−24	−8	2	−236.13	147.51
−25	−9	2	179.87	145.14
23	−25	3	100.76	94.56
15	−18	3	−41.60	80.16
−24	−18	3	−114.10	158.87
19	−27	4	89.80	89.54
28	−39	4	−150.29	131.66
29	−41	4	61.80	136.15
−18	−22	4	−11.36	144.34
−10	−30	5	−24.56	137.87
−28	12	−7	−8.19	136.02
19	−2	−6	−4.89	100.37
−32	10	−6	5.28	136.95
10	3	−5	22.85	73.03
−7	10	−5	298.59	61.94
−32	9	−5	0.00	142.10
−33	7	−4	97.86	159.40
27	−13	−2	83.07	108.16
17	−5	−2	41.47	97.33
31	−19	−1	7.26	121.76
28	−16	−1	−14.79	108.16
11	−3	−1	291.99	63.92
−7	2	0	85.84	59.43

37	−33	1	64.31	138.80
−6	−1	1	329.10	58.64
20	−22	3	92.71	86.37
31	−35	3	73.16	131.14
27	−37	4	0.00	120.84
30	−43	4	27.07	140.51
29	−10	−6	23.77	139.32
2	6	−4	440.56	58.24
32	−16	−3	33.54	153.85
8	2	−3	354.32	68.41
6	3	−3	222.26	54.67
−21	9	−3	70.39	85.84
−22	7	−2	−5.02	91.25
−32	3	−2	−9.64	164.28
38	−27	−1	21.39	140.25
20	−9	−1	197.04	118.46
−11	5	−1	76.60	53.22
−29	2	−1	−33.02	131.53
−28	−1	0	33.68	137.74
32	−26	1	−98.39	151.21
−22	−2	1	206.54	104.72
−11	−4	2	177.89	82.54
−18	−5	2	−3.30	98.12
−16	−10	3	107.23	92.84
−23	−17	3	163.76	144.87
22	−30	4	43.32	105.65
−2	−15	4	20.60	52.16
18	−35	5	6.74	91.25
−24	12	−6	124.53	124.80
26	−8	−5	109.35	129.82
21	−4	−5	137.48	105.65
−12	11	−5	−29.32	66.69
26	−9	−4	9.24	105.25
21	−5	−4	0.13	96.40
12	1	−4	216.32	95.48
−12	10	−4	119.65	79.76
−24	10	−4	91.39	101.29
−28	9	−4	13.34	124.14
35	−19	−3	−59.03	147.64
29	−13	−3	−41.07	115.55
−15	9	−3	41.60	85.84
36	−22	−2	22.71	151.61
−26	6	−2	15.45	108.69
34	−22	−1	−116.48	159.40
25	−13	−1	36.98	103.93
−20	5	−1	153.72	111.33
−30	1	−1	276.40	153.72
21	−12	0	171.42	112.25
14	−7	0	1377.53	137.48
−29	−2	0	−149.23	151.87
−30	−3	0	−61.94	155.70
36	−31	1	−125.19	138.66
26	−28	3	103.93	123.61
30	−33	3	27.20	125.06

−12	−9	3	0.66	79.24
16	−24	4	0.00	73.16
31	−45	4	0.00	145.40
−12	−16	4	74.88	103.54
−9	−29	5	93.50	121.50
17	0	−7	35.52	119.65
12	2	−5	164.02	79.76
−33	8	−5	−82.93	138.80
−7	9	−4	3.96	50.98
10	1	−3	114.76	70.65
−24	4	−1	−7.79	97.99
−31	0	−1	0.00	141.57
20	−14	1	132.46	109.48
13	−9	1	246.43	67.48
−18	−1	1	−51.24	105.78
32	−30	2	24.70	132.06
−8	−15	4	107.63	77.92
−18	−25	4	0.00	149.23
−18	−28	4	174.85	165.08
2	−24	5	9.77	48.47
32	−12	−7	7.26	133.12
24	−5	−7	10.56	137.61
−15	12	−6	115.55	82.67
−28	11	−6	−74.09	141.83
−24	11	−5	53.22	101.82
−28	10	−5	−153.06	148.83
−18	9	−3	128.10	98.12
−25	8	−3	45.69	99.84
24	−10	−2	72.24	110.40
29	−19	0	−4.75	113.18
36	−27	0	−129.42	141.70
28	−25	2	21.13	118.72
17	−15	2	22.32	95.22
−17	−11	3	36.58	94.42
−22	−16	3	−2.25	127.44
−13	−17	4	0.00	128.10
−16	−20	4	−3.30	121.76
−9	−32	5	−147.25	134.44
26	−7	−6	40.15	143.29
34	−15	−5	115.55	131.93
4	4	−3	358.15	53.88
13	−4	−1	207.73	67.22
−6	4	−1	655.82	76.33
−17	5	−1	466.97	97.33
32	−22	0	106.44	134.17
26	−16	0	37.11	99.18
−23	1	0	49.26	97.73
28	−21	1	219.62	113.18
−23	−3	1	61.80	104.59
37	−37	2	0.00	135.76
−6	−9	3	126.65	68.94
−21	−15	3	165.87	130.08
25	−33	4	0.00	94.16
−14	−18	4	15.85	121.23

−15	−19	4	−14.53	121.36
29	−9	−7	84.65	129.42
−25	12	−7	−11.62	136.82
−29	11	−7	30.37	127.84
21	−3	−6	78.44	110.80
−21	12	−6	87.95	99.97
31	−12	−5	0.00	139.19
34	−16	−4	17.70	151.08
31	−13	−4	87.42	143.42
14	0	−4	584.77	106.18
12	0	−3	85.31	77.78
19	−6	−2	152.53	106.57
−13	7	−2	74.61	65.77
−19	7	−2	214.86	111.72
−27	5	−2	16.38	106.97
37	−25	−1	−92.05	131.00
−14	5	−1	−1.19	72.37
−19	2	0	0.00	109.87
25	−18	1	13.73	97.46
35	−29	1	−40.81	128.63
29	−31	3	−137.61	124.80
17	−19	3	73.95	74.22
−9	−9	3	−42.26	85.18
−18	−12	3	59.03	113.44
−20	−14	3	1.19	128.10
−5	−15	4	75.01	66.29
−5	−25	5	−20.87	91.78
34	−14	−6	72.63	138.53
−18	12	−6	−6.47	86.63
14	1	−5	18.22	82.41
2	7	−5	48.60	53.09
−21	10	−4	186.34	93.10
−29	8	−4	29.32	142.76
−31	5	−3	−42.52	148.70
32	−17	−2	46.35	136.15
−25	3	−1	11.62	99.18
16	−8	0	413.35	86.76
−24	−4	1	44.77	103.40
31	−28	2	61.67	135.89
36	−35	2	0.00	129.68
−26	−13	2	48.33	136.95
−19	−13	3	13.21	138.14
9	−19	4	99.31	45.43
−17	−24	4	0.00	133.38
17	−33	5	−92.71	85.84
22	−40	5	12.81	104.20
−1	−24	5	55.86	82.27
−6	−26	5	62.86	94.82
−7	−27	5	−0.13	101.16
31	−11	−6	−118.59	127.70
8	5	−6	32.75	67.35
−21	11	−5	238.11	94.03
23	−6	−4	−15.05	105.52
35	−20	−2	−26.28	144.87

29	−14	−2	23.77	107.37
−16	7	−2	309.95	92.05
33	−20	−1	0.00	138.00
30	−17	−1	−0.53	108.82
22	−10	−1	77.52	105.78
35	−25	0	22.98	133.65
15	−10	1	123.74	68.28
−25	−5	1	−40.41	124.80
−27	−7	1	−79.50	131.14
−28	−8	1	−142.49	133.25
−15	−5	2	13.34	94.69
−20	−7	2	33.81	94.42
25	−26	3	−94.69	100.23
35	−40	3	0.00	139.19
36	−42	3	29.45	139.98
21	−28	4	−13.21	95.74
7	−18	4	98.12	35.92
−17	−30	4	20.07	156.76
21	−38	5	22.85	97.33
23	−42	5	22.71	106.04
19	−1	−7	−64.58	124.40
10	4	−6	512.00	91.91
6	6	−6	169.17	62.73
−29	10	−6	−128.50	134.17
28	−9	−5	59.56	136.55
23	−5	−5	36.18	97.86
−29	9	−5	33.02	153.19
28	−10	−4	31.96	123.74
−15	10	−4	15.45	87.95
34	−17	−3	−5.68	134.04
−32	4	−3	−19.15	139.72
−23	6	−2	13.47	93.24
15	−5	−1	116.08	76.07
−24	0	0	0.00	102.35
−15	−1	1	248.80	100.23
35	−33	2	−139.85	125.33
−25	−12	2	−3.83	128.50
22	−23	3	29.32	87.95
−13	−10	3	1.19	85.18
30	−40	4	−41.86	114.10
−17	−27	4	27.34	140.64
−8	−31	5	−8.19	114.89
−15	11	−5	29.32	75.01
16	−1	−4	196.77	85.44
−18	10	−4	101.95	81.88
−25	9	−4	11.75	96.14
−30	7	−4	1.06	144.21
31	−14	−3	66.16	129.82
14	−1	−3	63.26	95.88
−28	4	−2	81.61	110.67
27	−14	−1	77.52	100.23
−21	4	−1	276.14	115.42
23	−13	0	48.07	94.95
34	−27	1	3.43	127.44

22	−15	1	0.13	94.42
−19	−2	1	−10.56	92.71
19	−16	2	88.88	96.54
−21	−8	2	59.82	103.40
−24	−11	2	−78.05	127.97
33	−36	3	20.07	121.36
−22	−19	3	−51.24	134.83
24	−31	4	37.90	103.01
18	−25	4	−15.85	76.60
29	−38	4	23.51	111.99
11	−20	4	82.41	54.81
31	−42	4	−17.96	121.10
−9	−16	4	−4.09	77.26
−16	−23	4	−17.17	127.97
26	−6	−7	0.13	122.29
−13	12	−7	89.80	70.52
−22	12	−7	59.30	127.31
−30	10	−7	31.83	123.21
12	3	−6	87.42	78.18
−25	11	−6	−15.72	127.44
16	0	−5	287.36	89.14
−18	11	−5	362.11	91.91
37	−20	−3	−23.37	130.21
−22	8	−3	67.88	92.71
38	−23	−2	25.49	127.97
26	−11	−2	38.96	98.12
36	−23	−1	−20.60	131.80
−26	2	−1	53.09	104.99
−16	2	0	126.12	85.71
−25	−1	0	74.22	101.03
−9	−1	1	60.09	50.71
27	−23	2	−1.45	91.91
−22	−9	2	−118.99	123.87
−23	−10	2	48.73	136.29
28	−29	3	46.49	125.85
32	−44	4	−111.59	124.14
20	−36	5	−29.58	86.50
28	−8	−6	7.79	127.44
−25	10	−5	−82.67	104.59
28	−11	−3	31.96	100.23
21	−7	−2	90.59	99.05
−29	3	−2	−35.39	128.50
31	−20	0	−4.36	108.42
−10	2	0	−3.30	57.84
38	−32	1	114.23	131.27
−12	−1	1	484.00	78.31
30	−26	2	−146.85	128.36
24	−20	2	−10.70	92.71
34	−31	2	−60.75	122.29
32	−34	3	94.95	117.01
−1	−10	3	393.41	52.82
28	−36	4	73.95	106.18
31	−10	−7	−19.68	115.95
23	−4	−6	102.08	125.19

−30	9	−6	−62.33	121.36
−30	8	−5	−33.54	139.98
−31	6	−4	−64.05	145.53
28	−17	0	62.73	104.86
18	−9	0	176.43	91.39
−20	1	0	36.58	98.52
−26	−2	0	61.67	108.69
30	−22	1	−16.24	113.18
−12	−5	2	4.62	87.56
19	−20	3	27.20	78.44
−21	−18	3	0.13	122.29
33	−46	4	0.00	129.82
−16	−26	4	−29.05	123.87
−7	−30	5	42.52	100.37
21	−2	−7	−15.05	123.08
−26	11	−7	10.56	118.72
−31	9	−7	0.00	121.23
14	2	−6	78.31	82.54
−5	10	−6	32.62	31.96
33	−13	−5	−30.51	121.10
36	−17	−4	−57.05	122.29
33	−14	−4	237.84	131.27
16	−2	−3	42.13	97.20
−30	2	−2	13.21	137.08
−31	1	−2	−81.22	130.21
17	−6	−1	825.25	123.34
−27	1	−1	67.35	103.67
34	−23	0	0.13	143.95
−13	2	0	78.18	65.90
−27	−3	0	110.67	143.81
−28	−4	0	59.16	136.42
−29	−5	0	203.51	129.16
27	−19	1	2.64	99.18
17	−11	1	371.49	88.61
−14	−11	3	210.24	89.01
−10	−17	4	31.30	84.65
−16	−29	4	−33.54	132.19
−19	12	−7	−11.49	102.88
4	7	−6	486.25	67.35
25	−6	−5	26.94	109.48
25	−7	−4	137.08	99.71
18	−2	−4	73.69	91.39
−32	5	−4	−13.07	141.31
−10	8	−3	216.58	60.35
34	−18	−2	−49.39	141.04
−24	5	−2	85.71	95.48
24	−11	−1	−8.32	87.03
−28	0	−1	77.65	128.63
33	−25	1	−123.48	117.67
37	−30	1	14.26	119.65
−20	−3	1	101.03	91.12
10	−10	2	4463.40	370.70
−6	−5	2	522.96	66.56
31	−32	3	0.00	112.65

-20	-17	3	-76.73	110.40
27	-34	4	-52.82	96.27
13	-21	4	48.73	61.67
-14	-21	4	-68.54	112.12
-7	-33	5	155.04	112.78
-16	12	-7	12.81	83.59
33	-12	-6	36.05	117.67
-10	11	-6	79.50	60.75
30	-10	-5	-42.66	130.74
18	-1	-5	169.43	90.86
-31	7	-5	-54.94	131.40
30	-11	-4	-10.43	130.21
-26	8	-4	-5.68	95.61
31	-15	-2	101.16	112.91
10	0	-2	4892.46	407.54
-20	6	-2	104.33	105.25
32	-18	-1	-16.51	121.76
-22	3	-1	143.29	91.39
-29	-1	-1	-52.56	145.14
-30	-2	-1	25.09	134.31
33	-29	2	183.30	125.59
38	-36	2	34.86	125.72
8	-9	2	1723.00	149.89
-25	-15	2	50.45	123.87
24	-24	3	51.11	83.99
10	-14	3	256.07	63.39
-10	-10	3	-11.62	73.56
-11	-18	4	0.00	102.48
19	-34	5	20.47	82.01
-6	-29	5	80.03	93.76
16	1	-6	-18.09	82.01
-22	11	-6	-73.82	96.80
-31	8	-6	16.51	120.04
-22	9	-4	140.51	91.25
36	-18	-3	-73.69	126.78
33	-15	-3	0.00	130.61
-19	8	-3	103.80	96.27
37	-21	-2	0.00	118.06
8	1	-2	2221.13	192.28
35	-21	-1	0.00	127.04
29	-15	-1	114.10	101.42
-18	4	-1	704.68	117.40
37	-26	0	20.73	124.27
25	-14	0	47.15	92.57
24	-16	1	-52.96	91.65
21	-17	2	117.40	82.67
12	-11	2	183.70	60.35
-9	-5	2	27.73	62.07
27	-27	3	-15.85	114.76
8	-13	3	326.19	57.31
-15	-12	3	78.97	83.99
-19	-16	3	-31.69	114.63
23	-29	4	15.19	92.44
-6	-16	4	103.80	69.20

−12	−19	4	33.41	114.36
−13	−20	4	170.09	110.93
16	−31	5	0.00	74.75
24	−41	5	9.38	95.88
28	−7	−7	32.62	113.70
−26	10	−6	−10.17	136.02
−32	6	−5	88.48	124.80
18	−3	−3	22.98	91.25
2	5	−3	466.84	51.64
−28	5	−3	29.98	109.61
28	−12	−2	−31.17	93.10
23	−8	−2	22.98	84.25
12	−1	−2	138.14	69.46
−3	5	−2	290.27	33.28
−8	6	−2	1295.39	117.67
20	−10	0	124.14	97.20
−21	0	0	225.56	93.10
−16	−2	1	78.71	105.25
−21	−4	1	236.79	97.33
−27	−10	1	100.89	114.76
37	−34	2	0.00	124.40
−24	−14	2	−121.63	113.97
36	−39	3	103.80	119.91
12	−15	3	53.62	69.73
37	−41	3	−66.95	120.70
20	−26	4	28.66	80.56
−15	−25	4	−30.37	112.65
23	−39	5	−6.60	89.93
25	−43	5	7.79	97.73
30	−9	−6	−56.79	118.85
25	−5	−6	113.04	123.34
−22	10	−5	84.52	90.20
−26	9	−5	−57.18	114.50
30	−12	−3	204.43	113.57
38	−24	−1	20.47	112.25
19	−7	−1	343.36	108.95
36	−28	1	−3.17	123.08
19	−12	1	421.41	110.93
29	−24	2	50.05	106.31
30	−30	3	−23.37	113.57
35	−37	3	22.19	118.19
−16	−13	3	5.81	94.16
−17	−14	3	42.52	112.65
−18	−15	3	−52.82	122.82
26	−32	4	−22.58	96.27
15	−22	4	45.96	64.71
32	−41	4	−31.96	106.57
−15	−31	4	−109.08	127.84
−3	−26	5	32.22	78.44
−5	−28	5	0.00	85.31
23	−3	−7	122.55	117.27
−27	10	−7	111.72	119.52
20	−3	−4	51.90	86.50
−16	8	−3	394.60	96.14

14	−2	−2	203.51	82.54
−25	4	−2	−1.45	102.22
33	−21	0	97.73	123.87
−22	−5	1	71.84	93.50
−26	−9	1	−48.20	126.78
26	−21	2	99.71	95.35
14	−12	2	115.55	70.52
21	−21	3	414.41	93.10
31	−39	4	−101.95	102.61
33	−43	4	−140.64	113.18
−15	−28	4	0.00	113.31
22	−37	5	60.22	83.20
−4	−27	5	28.26	80.56
−6	−32	5	−104.06	99.97
33	−11	−7	10.30	115.82
−8	11	−7	71.05	51.50
−23	11	−7	11.09	120.84
18	0	−6	97.99	85.31
20	−2	−5	23.11	87.95
27	−8	−4	−47.41	98.52
−27	7	−4	97.99	112.65
−13	8	−3	315.10	79.37
−29	4	−3	−87.29	125.72
6	2	−2	1538.91	135.63
26	−12	−1	48.33	97.20
−23	2	−1	39.75	92.18
30	−18	0	4.49	96.40
−17	1	0	−56.26	92.84
29	−20	1	85.31	102.35
−23	−6	1	145.27	105.65
−25	−8	1	43.58	126.65
32	−27	2	−9.64	121.89
36	−32	2	0.00	106.71
−18	−8	2	15.32	88.35
−23	−13	2	−34.60	117.67
34	−35	3	−103.14	113.57
14	−16	3	140.38	78.44
−20	−20	3	6.74	115.03
30	−37	4	49.79	100.63
34	−45	4	−107.23	113.84
0	−16	4	195.05	44.90
−19	11	−6	152.79	84.12
35	−14	−5	5.41	111.46
27	−7	−5	−46.09	121.10
35	−15	−4	14.00	124.93
−5	7	−3	236.26	38.69
−30	3	−3	42.13	128.76
−17	6	−2	119.12	88.35
−9	4	−1	742.45	82.14
−15	4	−1	218.96	78.18
36	−24	0	83.86	117.93
−22	−1	0	68.80	90.86
6	−8	2	1241.77	108.82
−22	−12	2	−24.96	128.10

−7	−10	3	263.46	80.82
−14	−24	4	−4.09	105.12
−27	9	−6	27.73	121.36
−19	9	−4	157.15	82.80
20	−4	−3	23.51	85.44
−31	2	−3	−51.50	127.04
36	−19	−2	−9.64	120.57
33	−16	−2	150.68	130.48
16	−3	−2	156.62	93.50
−26	3	−2	65.37	94.42
27	−15	0	29.98	88.74
35	−26	1	0.00	114.89
16	−13	2	68.67	81.22
−13	−6	2	245.90	96.67
−19	−9	2	−13.34	91.39
−21	−11	2	15.58	122.82
−3	−16	4	253.69	58.77
1	−25	5	49.26	58.11
12	4	−7	49.65	78.18
10	5	−7	−8.19	69.20
32	−11	−5	−8.19	111.86
−27	8	−5	−114.50	127.44
32	−12	−4	−0.26	123.34
−28	6	−4	−47.28	120.04
25	−9	−2	78.44	94.16
−21	5	−2	146.85	87.82
34	−19	−1	−32.75	125.46
21	−8	−1	74.48	108.69
−12	4	−1	222.13	65.63
22	−11	0	52.82	103.27
26	−17	1	80.16	88.35
39	−31	1	13.34	116.21
21	−13	1	0.00	98.12
−17	−3	1	5.02	96.40
−20	−10	2	0.00	99.97
−24	−17	2	−49.79	116.61
26	−25	3	9.51	94.16
33	−33	3	76.86	101.95
16	−17	3	96.40	79.24
−4	−10	3	802.67	92.05
−11	−11	3	9.77	73.29
25	−30	4	39.88	90.07
17	−23	4	−11.23	70.26
−7	−17	4	63.52	70.39
21	−35	5	−73.03	78.97
18	−32	5	30.90	73.56
30	−8	−7	−37.11	105.52
14	3	−7	127.97	85.84
−28	9	−7	−16.38	104.86
27	−6	−6	−111.33	118.72
20	−1	−6	93.50	96.93
−13	11	−6	94.69	69.86
22	−3	−5	37.11	89.67
−10	10	−5	36.32	60.62

−19	10	−5	51.64	80.69
22	−4	−4	91.39	87.82
−10	9	−4	49.79	64.71
−23	8	−4	32.22	92.97
35	−16	−3	−118.59	131.40
30	−13	−2	−129.42	97.33
37	−22	−1	8.45	117.53
31	−16	−1	−53.09	99.31
−19	3	−1	199.41	111.99
−24	1	−1	−107.76	98.78
−23	−2	0	12.02	99.18
−28	−7	0	71.05	121.76
−4	−2	1	934.86	92.18
−13	−2	1	503.42	86.63
35	−30	2	59.69	107.50
29	−28	3	−11.49	113.57
−19	−19	3	−7.00	103.67
−20	−23	3	−6.07	113.31
22	−27	4	−84.12	85.44
−14	−27	4	−13.73	104.86
−14	−30	4	0.00	109.35
4	−25	5	14.39	29.71
−5	−31	5	0.26	84.65
25	−4	−7	54.81	110.93
8	6	−7	13.21	61.01
32	−10	−6	−8.72	106.04
−16	11	−6	116.87	74.48
−23	10	−6	73.56	100.10
32	−13	−3	26.68	121.76
18	−4	−2	57.31	99.05
−11	6	−2	539.07	72.77
−14	6	−2	87.16	69.07
−27	2	−2	−21.79	94.69
39	−27	0	−36.45	105.65
−27	−6	0	−59.56	125.46
23	−22	3	130.87	81.48
6	−12	3	342.70	45.43
11	−27	5	59.43	47.15
−5	−34	5	−26.15	91.25
−23	9	−5	40.41	83.20
−28	7	−5	8.98	133.38
−29	5	−4	−2.51	127.84
−28	1	−2	−24.43	117.27
−25	0	−1	0.00	95.35
−5	1	0	653.44	71.05
−24	−3	0	−5.02	93.24
−25	−4	0	−33.15	99.84
−26	−5	0	−23.77	120.57
38	−29	1	−37.77	106.44
31	−25	2	−17.30	117.53
28	−22	2	−13.07	91.25
28	−33	4	−38.56	90.59
25	−40	5	28.79	80.95
13	−28	5	13.60	62.46

26	−42	5	−32.09	87.16
16	2	−7	86.90	97.46
−20	11	−7	0.00	101.42
−29	8	−7	0.00	98.91
−28	8	−6	−33.68	116.74
29	−8	−5	84.78	120.44
29	−9	−4	64.18	123.61
−16	9	−4	188.85	86.37
−30	4	−4	0.00	123.21
−29	0	−2	46.35	124.01
−30	−1	−2	14.66	118.19
28	−13	−1	−31.96	95.74
−29	−4	−1	−5.41	114.10
35	−22	0	125.99	119.91
32	−19	0	−39.22	114.50
−14	1	0	225.96	77.26
−18	0	0	385.75	115.55
−26	−12	1	−16.51	106.04
18	−14	2	136.68	93.50
−1	−6	2	148.30	29.32
−23	−16	2	−48.07	110.01
32	−31	3	77.92	101.55
−12	−12	3	73.16	80.82
−18	−18	3	106.84	100.37
−8	−18	4	−0.13	72.63
−12	−22	4	16.90	92.84
−24	10	−7	18.49	114.50
−16	10	−5	38.96	73.16
−13	9	−4	114.50	77.26
−25	5	−3	5.28	89.27
23	−9	−1	−2.11	88.22
−26	−1	−1	77.39	96.54
−27	−2	−1	17.17	110.93
−28	−3	−1	98.65	125.85
31	−21	1	86.10	100.23
−18	−4	1	−33.28	79.76
34	−28	2	14.26	106.57
18	−18	3	195.85	74.75
33	−40	4	116.48	94.69
34	−42	4	47.67	98.78
35	−44	4	−27.73	100.76
24	−38	5	56.52	80.16
−4	−30	5	62.86	78.05
22	−2	−6	−64.84	104.46
24	−4	−5	0.00	94.56
−29	6	−5	45.69	118.46
24	−5	−4	−32.49	89.27
35	−17	−2	0.00	114.63
27	−10	−2	−10.70	90.86
20	−5	−2	121.89	93.63
−22	4	−2	10.83	85.18
38	−25	0	−26.02	101.82
29	−16	0	43.32	94.29
24	−12	0	0.00	75.27

13	−5	0	694.24	88.88
11	−4	0	978.18	101.55
28	−18	1	89.80	89.80
23	−14	1	19.02	88.88
−10	−2	1	937.90	101.55
38	−33	2	123.74	104.72
−10	−6	2	204.83	78.71
36	−36	3	0.00	101.55
−17	−17	3	14.26	104.86
19	−24	4	−15.85	71.05
32	−38	4	0.13	90.99
−9	−19	4	8.19	85.97
−11	−21	4	45.56	97.20
−13	−26	4	0.00	96.80
−13	−32	4	14.79	104.99
20	−33	5	−12.15	72.63
15	−29	5	41.07	55.99
32	−9	−7	−70.39	102.88
27	−5	−7	32.62	101.82
18	1	−7	106.57	110.14
−30	7	−7	20.21	102.22
29	−7	−6	83.07	106.84
−29	7	−6	100.76	107.37
34	−12	−5	50.58	101.82
−13	10	−5	113.97	67.22
34	−13	−4	10.17	108.42
−24	7	−4	73.29	89.67
38	−20	−2	115.55	109.21
36	−20	−1	13.21	110.67
15	−6	0	280.50	75.27
−25	−11	1	54.67	103.93
−22	−15	2	10.04	100.89
28	−26	3	−10.43	106.97
−13	−13	3	−20.87	77.39
−19	−25	3	58.77	106.04
36	−46	4	−55.99	101.82
−10	−20	4	60.35	99.97
−13	−29	4	−12.41	98.39
6	7	−7	72.11	55.07
−30	5	−5	50.18	115.82
37	−17	−3	−41.60	104.33
32	−14	−2	44.11	114.50
33	−17	−1	107.50	115.95
−20	2	−1	46.49	93.63
37	−27	1	20.07	101.03
−19	−5	1	149.36	86.50
20	−15	2	83.33	88.08
−23	−19	2	50.58	102.08
−14	−14	3	20.47	81.88
−16	−16	3	−108.16	111.33
27	−31	4	−28.53	86.90
24	−28	4	−21.92	87.03
31	−36	4	9.24	85.84
23	−36	5	26.28	75.54

9	−26	5	23.24	27.60
−3	−29	5	7.13	71.05
−4	−33	5	28.53	77.52
−17	11	−7	33.81	80.03
34	−11	−6	63.52	100.76
−24	9	−6	−67.22	104.20
−30	6	−6	−45.43	101.69
−31	4	−5	0.00	105.65
−20	8	−4	−54.67	80.69
34	−14	−3	13.47	111.06
24	−6	−3	160.98	91.78
−26	4	−3	48.47	91.39
−18	5	−2	302.95	98.39
9	−3	0	470.80	67.62
−11	1	0	389.58	67.75
−19	−1	0	48.33	90.86
12	−7	1	661.10	80.16
−7	−2	1	1424.28	129.02
−24	−10	1	−61.67	117.53
37	−31	2	10.70	96.80
−21	−14	2	−40.28	101.55
31	−29	3	74.35	101.55
25	−23	3	−15.85	80.82
35	−34	3	−11.62	97.20
20	−19	3	49.79	75.67
−8	−11	3	67.22	71.97
−15	−15	3	45.83	91.52
−18	−21	3	0.00	96.01
−2	−28	5	57.45	67.22
−1	−27	5	19.94	64.18
33	−26	2	23.64	118.72
−12	−25	4	126.65	92.84
30	−23	2	−11.62	104.46
30	−14	−1	8.19	90.33
31	−10	−4	−14.26	121.36
25	−10	−1	37.64	86.76
31	−9	−5	−69.07	104.33
−23	−9	1	41.60	112.91
14	−8	1	167.98	64.45
17	−7	0	769.39	109.08
−20	−6	1	−15.19	87.42
24	−3	−6	92.44	111.06
−14	−3	1	1038.79	127.17
20	0	−7	21.13	109.35
−16	3	−1	302.68	89.93
−17	7	−3	170.23	93.24
−24	8	−5	19.81	89.54
−20	10	−6	76.99	80.03
21	−25	4	114.76	73.56
34	−20	0	−20.47	108.82
−4	−17	4	135.63	61.80
25	−15	1	6.60	83.86
26	−13	0	253.03	95.61
−20	−13	2	43.45	109.87

16	−9	1	297.27	87.69
−21	−7	1	185.15	89.01
26	−6	−4	−6.21	90.07
22	−6	−2	0.66	80.56
10	−6	1	1568.75	141.70
−8	1	0	808.08	87.56
−23	3	−2	125.72	91.52
−27	3	−3	163.49	104.72
−25	9	−7	−10.30	106.97
−11	11	−7	195.85	61.41
28	−43	5	−3.43	81.48
27	−41	5	−12.41	78.84
34	−32	3	65.50	91.65
37	−23	0	−70.12	115.69
33	−22	1	61.54	118.99
29	−11	−2	45.83	86.24
−16	−9	2	0.79	80.29
26	−5	−5	−8.19	114.50
14	−3	−1	1672.68	160.45
12	−2	−1	933.80	101.95
15	1	−4	94.03	75.94
−21	1	−1	−31.56	82.41
13	2	−4	125.72	79.63
−28	2	−3	70.65	113.31
−25	6	−4	−45.96	88.88
−20	9	−5	−8.06	80.16
−14	11	−7	324.08	74.88
26	−39	5	10.70	78.97
22	−34	5	0.00	71.45
−12	−31	4	−27.20	92.71
36	−29	2	70.92	95.61
−12	−28	4	3.83	89.67
−18	−27	3	55.47	99.84
−18	−24	3	−63.26	99.44
−17	−20	3	32.49	91.65
30	−19	1	107.89	95.88
−22	−18	2	−64.05	96.27
31	−17	0	−14.66	91.78
22	−16	2	65.90	80.43
−19	−12	2	−46.88	96.80
−17	−10	2	−65.24	82.14
31	−8	−6	−102.88	97.20
19	−8	0	408.60	96.80
29	−6	−7	−100.50	101.69
−7	−6	2	132.46	55.60
16	−4	−1	373.07	88.88
−20	−2	0	55.99	79.50
−30	0	−3	−11.49	112.91
−29	1	−3	90.86	121.63
13	3	−5	62.20	73.43
11	3	−4	309.68	85.71
36	−43	4	−26.41	90.59
35	−41	4	12.68	89.54
−3	−35	5	−8.45	68.67

-3	-32	5	0.00	69.73
26	-29	4	-19.68	76.20
30	-27	3	70.92	103.67
22	-20	3	205.49	79.24
37	-18	-2	47.81	99.05
36	-14	-4	117.80	97.86
-25	-14	1	-47.41	97.99
-18	-11	2	-18.75	84.12
18	-10	1	186.07	89.41
-26	-8	0	12.68	112.78
26	-7	-3	146.85	89.41
22	-1	-7	17.43	102.74
10	-1	-1	-10.70	50.18
17	0	-4	238.24	79.10
-15	0	0	98.52	84.92
15	2	-5	-40.01	76.60
-24	2	-2	221.07	97.86
-21	10	-7	0.00	104.33
37	-45	4	18.36	96.93
34	-39	4	-26.41	86.63
29	-32	4	-63.13	83.73
27	-24	3	96.80	87.56
38	-21	-1	-65.24	102.08
35	-18	-1	104.33	112.91
34	-15	-2	64.84	122.02
24	-7	-2	109.48	97.73
18	-5	-1	-19.02	89.93
26	-4	-6	-52.16	105.52
7	-2	0	-7.92	57.31
17	1	-5	179.74	81.61
11	4	-5	14.66	64.05
-26	5	-4	-8.85	92.71
-25	7	-5	184.36	96.67
-25	8	-6	-109.61	111.33
33	-37	4	48.33	87.69
25	-37	5	29.45	72.77
33	-30	3	176.04	91.91
39	-28	1	108.03	95.48
23	-26	4	38.83	78.71
-21	-17	2	33.54	94.29
36	-15	-3	-102.74	100.63
33	-11	-4	177.89	104.99
27	-11	-1	-92.71	94.82
21	-9	0	210.90	103.93
28	-7	-4	-32.35	101.29
-11	-7	2	349.70	86.90
-25	-7	0	-25.88	117.93
-4	-6	2	1053.58	97.59
-28	-6	-1	-52.56	110.67
-21	-3	0	43.98	89.67
-29	-3	-2	2.11	109.21
19	-1	-4	3.30	79.24
15	0	-3	53.09	87.42
-22	0	-1	24.56	80.82

13	1	−3	20.73	79.10
−25	1	−2	−4.36	88.88
9	4	−4	123.61	75.80
−14	7	−3	1345.70	137.48
−26	8	−7	139.72	100.10
37	−35	3	46.22	92.18
−11	−33	4	5.68	87.03
39	−32	2	−25.36	95.48
32	−24	2	0.00	107.23
−10	−23	4	120.31	86.76
14	−20	4	43.71	57.05
−16	−19	3	−5.68	96.40
−5	−18	4	75.54	59.69
24	−17	2	59.69	89.14
27	−16	1	50.58	94.29
32	−15	−1	−20.60	99.31
28	−14	0	137.87	89.01
−24	−13	1	35.66	95.74
−9	−12	3	53.09	67.62
20	−11	1	267.16	105.12
33	−10	−5	107.50	96.80
28	−6	−5	69.86	109.35
−24	−6	0	4.75	98.39
8	−5	1	100.37	48.07
−23	−5	0	−50.71	87.29
−15	−4	1	234.01	94.69
−22	−4	0	56.79	91.39
−28	−2	−2	−48.47	119.78
17	−1	−3	16.24	85.58
19	0	−5	258.71	82.27
−26	0	−2	94.03	95.61
11	2	−3	54.94	73.16
−13	3	−1	66.16	64.71
−19	4	−2	134.57	100.63
−27	4	−4	142.63	104.99
−15	5	−2	383.90	85.97
−21	7	−4	175.38	82.80
−28	7	−8	100.10	94.56
−17	8	−4	72.37	80.56
−17	10	−6	170.23	74.09
35	−27	2	−57.84	96.67
−11	−27	4	70.78	86.90
−17	−23	3	30.51	90.73
36	−21	0	−26.81	104.99
29	−21	2	26.15	87.82
16	−21	4	38.17	62.46
31	−12	−2	−127.31	99.05
20	−6	−1	86.50	104.20
−27	−5	−1	28.92	114.63
24	−2	−7	−64.31	97.06
−23	−1	−1	38.30	92.97
−27	−1	−2	34.73	97.86
−17	2	−1	251.58	90.59
−4	3	−1	732.41	73.69

9	5	−5	62.99	61.54
−26	6	−5	60.48	112.91
−2	−31	5	7.92	66.56
−11	−30	4	52.69	84.65
−17	−26	3	0.00	93.24
39	−24	0	−58.64	91.52
24	−21	3	91.12	78.58
32	−20	1	39.88	97.86
12	−19	4	157.81	52.69
33	−18	0	5.02	102.74
−5	−11	3	172.34	69.07
33	−9	−6	15.19	89.54
28	−8	−3	129.16	93.63
31	−7	−7	−68.94	92.05
−26	−4	−1	82.27	104.72
−25	−3	−1	104.59	89.54
21	−2	−4	112.65	80.95
19	−2	−3	107.76	77.12
−24	−2	−1	−11.49	88.88
−28	3	−4	−108.16	108.16
−8	7	−3	81.88	45.16
−26	7	−6	270.99	123.21
−27	7	−7	32.22	95.61
−5	9	−5	194.92	45.30
−21	9	−6	−89.01	78.84
29	−42	5	71.71	76.07
24	−35	5	53.48	70.12
36	−33	3	48.99	86.76
21	−32	5	−30.64	69.20
38	−26	1	−40.15	93.10
18	−22	4	9.77	64.31
−9	−22	4	80.56	87.69
−15	−18	3	−61.28	101.16
−20	−16	2	−39.49	93.37
−23	−12	1	138.40	101.55
23	−10	0	−27.86	87.69
26	−8	−2	−62.60	86.76
28	−5	−6	−8.85	94.82
−11	−3	1	1733.30	160.32
21	−1	−5	59.30	78.97
8	0	−1	133.12	52.03
15	3	−6	−29.71	70.12
−11	7	−3	1267.39	122.68
−5	8	−4	83.46	38.69
−21	8	−5	−9.77	76.73
−17	9	−5	28.92	73.43
28	−40	5	−17.56	73.95
−2	−34	5	0.00	67.88
38	−30	2	−3.30	87.95
28	−30	4	115.82	82.93
32	−28	3	−60.75	93.50
3	−26	5	20.34	48.73
29	−25	3	−57.71	101.55
−21	−20	2	16.51	88.08

−6	−19	4	10.17	64.58
−1	−17	4	86.24	45.03
36	−16	−2	−5.28	99.05
−10	−13	3	−24.17	67.88
29	−12	−1	30.51	86.90
22	−12	1	194.79	91.39
22	−7	−1	78.31	86.63
−16	−5	1	165.47	88.61
−16	−1	0	−16.51	95.22
17	2	−6	79.90	73.43
13	4	−6	−0.79	68.67
−23	4	−3	0.79	92.05
−27	5	−5	18.49	117.01
−27	6	−6	33.54	114.76
4	8	−7	110.54	45.56
25	−27	4	−38.56	79.90
−16	−22	3	16.77	85.71
−8	−21	4	−35.26	88.35
−7	−20	4	57.45	72.63
37	−19	−1	−59.03	99.97
29	−17	1	−3.04	82.27
−14	−17	3	106.57	96.54
30	−15	0	5.15	81.35
15	−15	3	−9.11	79.24
−22	−11	1	29.45	104.72
−26	−11	0	−2.38	88.88
30	−8	−4	−10.96	113.70
30	−7	−5	56.65	98.65
26	−3	−7	32.22	89.54
21	−3	−3	280.10	88.48
9	3	−3	328.04	70.52
−28	4	−5	−48.20	111.33
−28	6	−7	0.00	94.95
−22	9	−7	−31.83	101.42
−18	10	−7	−41.60	76.99
37	−42	4	61.67	88.22
36	−40	4	−33.81	85.97
27	−38	5	0.00	71.18
31	−33	4	−31.43	82.14
−1	−30	5	−46.49	61.54
−10	−26	4	13.73	81.09
20	−23	4	−8.85	64.71
34	−16	−1	65.50	112.12
17	−16	3	−1.19	74.61
−13	−16	3	21.92	76.99
−24	−16	1	−6.87	93.24
−19	−15	2	−155.44	108.42
−11	−14	3	29.32	71.97
35	−12	−4	7.92	95.88
17	−12	2	−31.96	78.84
35	−11	−5	−24.56	92.18
15	−11	2	176.96	74.75
4	−7	2	1838.95	153.19
23	−3	−4	39.35	83.46

−12	0	0	749.71	89.14
19	1	−6	48.73	80.56
−30	2	−5	49.13	97.86
−10	3	−1	1269.24	119.78
−20	3	−2	105.65	87.16
−29	3	−5	−109.74	105.25
11	5	−6	142.63	64.71
7	5	−4	147.91	64.45
−28	5	−6	−56.92	103.01
−29	5	−7	−14.92	88.88
−22	6	−4	120.44	89.54
−8	10	−6	2.91	46.62
−14	10	−6	398.03	77.65
35	−38	4	40.15	85.44
35	−31	3	0.00	85.31
34	−25	2	57.45	96.27
31	−22	2	14.13	103.27
26	−22	3	61.01	73.69
−12	−15	3	64.45	72.50
13	−14	3	−32.62	68.54
33	−13	−2	55.47	110.54
25	−11	0	−97.86	83.99
13	−10	2	259.50	66.82
−21	−10	1	155.04	94.03
30	−9	−3	51.37	94.42
33	−8	−7	−58.64	85.97
−17	−6	1	87.42	76.60
23	−2	−5	29.85	86.76
−24	3	−3	132.72	92.18
−29	4	−6	−48.60	92.57
−12	5	−2	230.45	61.94
39	−36	3	−13.73	88.48
23	−33	5	4.62	67.62
−10	−32	4	6.74	79.76
37	−28	2	7.53	88.61
16	−28	5	2.11	55.73
−16	−28	3	−6.74	87.95
−16	−25	3	−17.96	92.18
38	−22	0	17.30	87.82
−20	−19	2	86.37	87.95
10	−18	4	85.97	42.00
19	−17	3	77.52	68.54
−18	−14	2	39.22	95.22
24	−13	1	18.75	79.90
19	−13	2	123.61	93.90
28	−9	−2	−15.58	81.88
24	−8	−1	57.31	80.56
−18	−7	1	84.52	87.82
30	−6	−6	−18.88	87.16
23	−4	−3	133.65	88.48
17	−2	−2	110.80	99.05
15	−1	−2	57.58	75.27
21	0	−6	41.86	91.52
−18	1	−1	227.41	101.42

−14	8	−4	45.56	83.59
26	−36	5	−12.28	69.73
0	−29	5	62.07	58.11
−10	−29	4	−5.28	79.90
14	−27	5	0.00	48.86
34	−21	1	21.26	111.59
35	−19	0	−30.90	104.99
−19	−8	1	66.29	82.80
25	−4	−4	96.40	83.33
−17	−2	0	190.56	101.55
−29	−2	−3	20.07	98.91
−25	2	−3	29.71	85.58
7	6	−5	146.59	56.92
−22	7	−5	−21.26	79.90
−22	8	−6	63.26	90.86
−24	8	−8	25.49	86.76
−11	10	−6	35.92	58.50
34	−36	4	−38.43	83.07
−1	−36	5	−9.64	65.24
30	−31	4	28.13	79.50
18	−29	5	−3.96	62.86
22	−24	4	−56.26	68.67
−15	−21	3	−19.02	85.58
−23	−15	1	−26.94	90.33
31	−13	−1	−16.38	86.76
−17	−13	2	88.48	82.54
32	−9	−4	−47.54	106.31
11	−9	2	964.71	97.86
−27	−8	−1	3.83	97.06
−8	−7	2	113.70	61.94
28	−4	−7	−21.92	83.59
25	−3	−5	−27.34	101.03
19	−3	−2	303.34	98.91
−28	−1	−3	−73.43	110.93
−27	0	−3	142.36	111.06
−26	1	−3	−117.93	96.54
−21	2	−2	−106.44	86.24
−7	3	−1	2393.74	205.22
−14	9	−5	89.67	66.43
31	−43	5	19.41	72.24
38	−34	3	58.11	85.58
−1	−33	5	−16.24	61.80
34	−29	3	−36.71	85.97
27	−28	4	58.37	77.39
1	−28	5	−60.09	55.47
31	−26	3	11.36	101.29
−20	−22	2	16.38	88.08
31	−18	1	−46.75	93.37
21	−18	3	208.26	76.46
38	−17	−2	105.38	96.67
32	−16	0	−46.75	98.91
21	−14	2	191.88	83.20
−2	−11	3	38.03	48.33
−14	−10	2	122.02	76.99

−24	−9	0	43.84	113.84
32	−8	−5	−91.12	97.59
−28	−5	−2	63.52	97.73
23	−1	−6	−29.58	100.50
−16	4	−2	324.61	91.12
−6	5	−2	1211.53	108.29
−23	5	−4	157.68	86.50
9	6	−6	221.73	62.99
−18	7	−4	90.86	73.69
−23	8	−7	−11.23	106.97
30	−41	5	−30.90	70.65
−9	−34	4	42.92	78.18
36	−17	−1	70.78	109.87
26	−14	1	211.30	89.93
27	−12	0	60.48	81.75
−16	−12	2	−3.04	79.24
−15	−11	2	39.09	76.73
26	−9	−1	−16.38	87.69
25	−5	−3	30.90	83.33
21	−4	−2	255.67	85.18
−12	−4	1	795.67	103.54
−19	0	−1	285.38	97.33
−14	2	−1	613.42	90.73
−9	5	−2	1462.84	131.80
−18	9	−6	109.61	74.22
29	−39	5	−114.89	71.18
−15	−30	3	−28.00	85.05
36	−26	2	37.51	89.67
33	−23	2	71.84	102.08
28	−23	3	6.60	85.71
−19	−18	2	−28.53	87.16
35	−14	−2	−13.73	103.01
−22	−14	1	4.23	92.44
−6	−12	3	−8.72	71.05
32	−10	−3	−54.94	105.91
30	−10	−2	47.54	87.82
−23	−8	0	−67.22	95.08
−26	−7	−1	201.79	109.61
27	−5	−4	19.02	97.99
−27	−4	−2	−21.00	114.76
−8	−3	1	435.93	65.63
−18	−3	0	155.04	86.24
−22	1	−2	−42.66	87.42
7	4	−3	1652.88	149.36
−25	7	−8	35.13	85.31
−11	8	−4	29.71	65.11
−15	10	−7	110.54	66.82
38	−41	4	16.38	83.86
25	−34	5	1.85	66.29
37	−32	3	28.00	89.67
12	−26	5	16.24	44.77
24	−25	4	27.86	73.43
−15	−24	3	26.02	82.14
−14	−20	3	74.75	89.14

23	−19	3	128.63	77.39
−23	−18	1	−39.35	88.74
23	−15	2	75.67	76.20
32	−7	−6	−18.62	86.37
23	−5	−2	6.34	80.43
27	−4	−5	40.28	99.71
6	−4	1	849.28	87.82
25	−2	−6	109.08	101.82
5	−1	0	869.75	86.90
−24	4	−4	87.82	82.54
−23	6	−5	35.79	79.90
−23	7	−6	59.56	93.10
−18	8	−5	55.73	75.54
37	−39	4	17.30	79.10
28	−37	5	43.18	67.35
−9	−31	4	−19.68	76.73
39	−29	2	39.62	89.27
−9	−28	4	−70.12	79.10
−15	−27	3	−52.03	87.03
−8	−24	4	7.92	79.24
36	−22	1	−86.90	97.99
37	−20	0	54.67	97.46
33	−14	−1	−2.91	106.44
−25	−13	0	93.37	88.35
−22	−7	0	−23.51	84.25
−21	−6	0	129.02	82.01
−25	−6	−1	−45.96	104.72
−20	−5	0	76.33	85.18
−19	−4	0	178.81	80.03
−26	−3	−2	−32.88	101.29
−25	−2	−2	−8.45	84.39
−20	−1	−1	19.15	81.48
−24	−1	−2	94.42	82.14
−29	−1	−4	37.64	97.33
−23	0	−2	151.47	85.97
6	1	−1	442.80	61.67
17	3	−7	36.85	91.91
15	4	−7	−108.95	86.37
−26	6	−8	19.55	85.31
−24	7	−7	71.71	97.99
−8	8	−4	−7.92	54.01
−11	9	−5	11.36	64.18
−19	9	−7	−22.58	82.67
29	−29	4	−47.15	81.61
−2	−18	4	50.98	52.43
28	−15	1	84.92	82.41
29	−13	0	−7.79	83.33
34	−10	−4	11.36	90.59
28	−10	−1	8.58	83.73
34	−9	−5	7.26	83.07
27	−6	−3	29.05	80.16
18	−6	0	375.58	95.74
16	−5	0	550.69	90.99
−24	−5	−1	28.13	85.97

−13	−1	0	119.12	67.88
−9	0	0	1323.25	123.74
−28	0	−4	111.20	105.65
−25	3	−4	−152.53	88.48
−8	9	−5	216.84	60.35
36	−37	4	23.11	81.09
32	−32	4	−31.43	80.16
0	−32	5	42.13	59.96
33	−27	3	62.07	88.48
−19	−21	2	105.78	84.92
33	−19	1	120.70	100.50
−13	−19	3	−26.81	96.54
34	−17	0	2.64	107.10
2	−17	4	82.67	32.75
−18	−17	2	0.00	91.12
−21	−13	1	76.20	100.89
−23	−4	−1	10.30	82.14
−22	−3	−1	17.70	80.82
−21	−2	−1	56.92	84.12
21	1	−7	17.30	92.71
−20	4	−3	181.32	85.58
13	5	−7	−30.90	71.18
−24	5	−5	−102.61	85.31
−27	5	−8	−1.58	83.86
0	−35	5	10.17	60.75
36	−30	3	−17.43	81.88
30	−24	3	−68.28	96.67
−19	−24	2	11.49	83.20
25	−20	3	−1.85	76.46
34	−11	−3	15.19	106.97
32	−11	−2	−31.30	105.38
1	−11	3	453.37	47.15
17	−8	1	31.96	78.58
20	−7	0	162.04	91.91
29	−6	−4	42.52	104.72
25	−6	−2	0.13	84.52
29	−5	−5	−47.94	101.82
−13	−5	1	−35.39	79.63
14	−4	0	1360.76	136.42
27	−3	−6	0.00	88.61
−17	3	−2	413.48	100.50
−24	6	−6	89.27	107.23
−25	6	−7	−54.28	96.01
32	−42	5	79.76	70.92
27	−35	5	−14.92	66.69
26	−26	4	−88.35	77.65
−7	−23	4	−0.13	82.80
−14	−23	3	−26.81	80.95
38	−18	−1	85.05	93.24
−22	−17	1	−64.58	86.90
37	−15	−2	−7.66	93.24
−7	−13	3	137.08	64.58
−24	−12	0	27.34	89.01
19	−9	1	506.98	100.76

34	−8	−6	0.00	85.18
9	−8	2	1799.86	156.89
−9	−8	2	6.34	72.90
15	−7	1	−44.90	66.82
−25	4	−5	0.00	95.08
−19	6	−4	83.73	78.58
31	−40	5	−2.77	67.75
35	−35	4	0.00	81.88
24	−32	5	−1.98	66.95
38	−27	2	0.00	80.82
35	−24	2	17.70	96.80
−12	−18	3	66.03	82.01
−17	−16	2	−13.60	97.99
−20	−12	1	53.88	94.56
21	−10	1	348.24	100.76
22	−8	0	89.93	93.37
29	−7	−3	70.26	95.22
−25	5	−6	−76.07	106.04
−26	5	−7	2.64	90.20
5	6	−4	290.53	59.96
−12	10	−7	54.54	59.69
39	−33	3	−32.75	81.35
−8	−33	4	57.58	73.43
−14	−29	3	44.90	80.03
−8	−27	4	0.66	74.75
−14	−26	3	−28.00	82.80
−6	−22	4	32.49	79.76
39	−21	0	0.00	82.54
32	−21	2	82.54	98.52
19	−21	4	70.92	65.63
17	−20	4	74.22	62.60
−3	−19	4	53.75	54.67
27	−17	2	7.66	84.25
30	−16	1	129.42	84.25
35	−15	−1	4.49	106.57
31	−14	0	80.82	91.25
36	−11	−4	88.22	83.20
30	−11	−1	1.06	76.07
−26	−10	−1	14.26	103.67
27	−7	−2	−15.85	76.60
19	−4	−1	101.42	95.48
−28	−4	−3	0.00	102.08
17	−3	−1	1090.03	129.68
12	−3	0	468.68	71.45
−15	1	−1	274.95	83.07
−28	1	−5	0.00	92.71
−27	2	−5	50.58	101.29
−21	3	−3	115.82	78.05
−26	3	−5	63.52	107.89
−27	4	−7	5.28	83.99
11	6	−7	61.67	64.71
7	7	−6	576.45	74.48
−19	8	−6	−33.94	70.52
−21	8	−8	29.98	88.61

−6	10	−7	51.24	42.26
30	−38	5	30.11	67.09
31	−30	4	−47.94	79.76
−8	−30	4	−29.58	75.41
38	−23	1	44.11	84.39
27	−21	3	47.54	78.44
−18	−20	2	0.00	85.84
−11	−17	3	55.99	73.43
23	−11	1	104.99	79.90
−19	−11	1	51.64	82.67
24	−9	0	−0.79	84.25
31	−7	−4	−59.16	98.65
13	−6	1	26.81	62.86
−14	−6	1	166.26	89.54
29	−4	−6	18.36	86.63
18	1	−4	19.94	73.43
−28	2	−6	−70.52	88.88
−27	3	−6	4.75	100.10
−13	4	−2	39.09	68.14
−26	4	−6	80.82	102.61
−15	7	−4	108.69	82.27
−19	7	−5	183.70	78.18
−15	9	−6	73.03	67.62
34	−33	4	5.02	78.58
1	−31	5	−5.15	54.01
35	−28	3	0.00	80.56
28	−27	4	−35.52	79.76
32	−25	3	−0.13	91.52
21	−22	4	−1.85	64.18
−5	−21	4	26.41	62.60
35	−20	1	−32.49	100.10
−4	−20	4	8.98	55.60
15	−19	4	138.00	58.24
36	−18	0	−25.09	96.01
−21	−16	1	5.02	87.16
−16	−15	2	−12.28	84.39
−8	−14	3	−0.66	64.58
36	−12	−3	0.00	87.69
34	−12	−2	17.04	100.37
−5	−7	2	306.25	54.67
−27	−7	−2	75.01	97.59
31	−6	−5	43.05	87.56
21	−5	−1	10.70	87.82
−5	−3	1	87.69	44.24
−27	−3	−3	−124.14	112.65
15	−2	−1	316.68	83.20
−14	−2	0	105.52	78.18
20	0	−4	105.38	79.24
16	2	−4	41.86	70.12
−22	2	−3	13.47	82.41
−12	6	−3	312.98	71.97
−20	8	−7	18.22	91.39
1	−37	5	3.57	59.96
−18	−23	2	10.43	84.39

−13	−22	3	1.98	83.07
−10	−16	3	136.29	68.94
−9	−15	3	5.15	66.69
−25	−9	−1	14.39	103.40
31	−8	−3	147.38	103.93
−15	−7	1	32.22	78.71
−26	−2	−3	−19.02	102.74
22	−1	−4	24.83	75.01
20	1	−5	−44.11	73.29
18	2	−5	2.77	71.84
−11	2	−1	682.23	82.14
−18	2	−2	102.88	96.54
−20	5	−4	45.96	77.12
5	7	−5	132.06	52.69
−15	8	−5	75.01	65.90
−9	10	−7	39.35	50.84
38	−38	4	−36.05	78.05
29	−36	5	64.05	69.46
−7	−35	4	0.00	75.14
26	−33	5	24.43	66.16
38	−31	3	22.32	85.05
19	−28	5	−26.28	64.05
17	−27	5	33.02	57.84
23	−23	4	−30.77	66.03
−24	−15	0	28.13	85.71
32	−12	−1	0.00	93.90
25	−12	1	−52.56	86.50
26	−10	0	−15.32	87.69
−17	−9	1	46.75	78.71
29	−8	−2	−23.24	91.52
−16	−8	1	−57.45	76.20
20	−1	−3	4.75	76.73
−25	−1	−3	2.91	85.05
18	0	−3	179.21	78.71
−6	0	0	2650.46	227.67
−23	1	−3	56.52	91.78
16	3	−5	191.49	71.84
14	3	−4	523.36	83.99
−17	9	−8	14.79	92.31
34	−43	5	−16.11	71.45
−13	−31	3	−33.41	79.63
21	−29	5	2.51	65.24
37	−25	2	−20.34	84.25
29	−22	3	18.62	83.46
29	−18	2	70.65	73.29
32	−17	1	7.26	93.24
37	−16	−1	−41.07	93.50
33	−15	0	123.87	103.14
−15	−14	2	107.37	73.16
−22	−10	0	−50.84	95.88
−26	−6	−2	3.17	104.33
31	−5	−6	−0.26	86.63
−9	−4	1	368.32	66.43
24	−2	−4	−15.05	75.41

13	−1	−1	421.80	73.56
22	0	−5	31.43	80.56
−24	0	−3	19.68	81.22
16	1	−3	−2.64	77.39
−22	7	−8	47.01	86.76
−16	9	−7	118.59	71.97
33	−41	5	−56.65	68.41
37	−36	4	−136.29	82.67
1	−34	5	30.90	58.11
−13	−25	3	−89.27	80.56
34	−22	2	0.00	103.14
−17	−19	2	14.26	84.78
−21	−19	1	0.00	80.16
8	−17	4	−0.66	34.07
20	−16	3	56.79	70.52
18	−15	3	13.34	71.97
33	−8	−4	−33.81	95.48
−24	−8	−1	63.26	101.69
33	−7	−5	0.00	80.29
25	−7	−1	41.34	87.03
22	−2	−3	−0.13	76.33
−16	0	−1	305.72	90.07
14	4	−5	264.52	75.94
−20	7	−6	28.13	73.03
32	−39	5	33.81	68.14
33	−31	4	−82.67	79.24
23	−30	5	74.88	69.99
30	−28	4	−57.18	84.39
−13	−28	3	27.86	79.37
15	−26	5	−5.81	49.79
25	−24	4	0.00	75.01
37	−21	1	−14.13	90.59
38	−19	0	0.00	87.03
13	−18	4	92.44	50.98
22	−17	3	115.42	73.29
−20	−15	1	−49.39	108.03
36	−13	−2	−88.22	97.86
27	−13	1	19.02	79.76
−14	−13	2	0.00	71.71
20	−12	2	54.15	89.93
28	−11	0	0.00	81.09
18	−11	2	209.05	87.29
−21	−9	0	28.79	84.78
11	−5	1	84.92	52.03
−25	−5	−2	0.00	105.12
24	−3	−3	108.42	80.16
−15	−3	0	67.22	87.82
−28	−3	−4	−4.23	95.61
10	−2	0	31.69	51.77
24	−1	−5	8.72	89.93
−19	1	−2	−7.00	82.01
14	2	−3	216.58	86.76
−21	4	−4	218.43	85.84
−20	6	−5	21.53	76.46

-21	7	-7	18.09	96.67
-6	-37	4	-59.82	69.20
28	-34	5	34.86	67.75
-7	-32	4	-5.41	72.37
2	-30	5	34.73	51.37
37	-29	3	16.64	84.65
-7	-29	4	82.93	73.43
34	-26	3	19.02	84.52
-12	-21	3	12.55	89.80
-23	-14	0	-52.96	93.76
22	-13	2	-2.51	73.69
-3	-12	3	105.91	57.45
-13	-12	2	127.70	73.56
-12	-11	2	227.01	71.45
16	-10	2	125.33	76.86
33	-9	-3	-56.26	99.18
31	-9	-2	39.62	105.38
26	-3	-4	134.97	86.50
12	4	-4	180.13	71.18
-23	6	-8	13.34	82.67
9	7	-7	94.69	57.58
-12	-33	3	-63.26	80.16
6	-26	5	-15.98	28.13
-17	-22	2	-16.51	80.95
24	-18	3	200.73	78.05
34	-13	-1	-0.13	100.76
27	-8	-1	-2.91	80.43
-20	-8	0	0.00	86.37
-23	-7	-1	42.66	85.71
-24	-4	-2	-19.81	82.14
26	-2	-5	40.54	94.69
-27	-2	-4	7.79	107.23
-10	-1	0	498.93	72.63
-17	-1	-1	100.23	94.42
-20	0	-2	119.78	87.16
20	2	-6	-39.22	86.63
18	3	-6	20.60	74.61
5	5	-3	681.57	74.09
31	-37	5	50.98	67.88
-6	-25	4	26.81	75.54
-17	-25	2	-63.26	84.25
31	-23	3	0.00	95.35
34	-18	1	-68.41	100.37
35	-16	0	20.73	103.93
24	-14	2	71.71	77.52
-19	-14	1	0.00	111.72
-25	-12	-1	-1.19	86.10
33	-6	-6	-49.52	81.75
26	-4	-3	-15.85	83.33
-16	-4	0	-25.09	91.25
-23	-3	-2	120.70	84.39
-22	-2	-2	12.94	77.65
-21	-1	-2	1.06	85.44
11	0	-1	1562.15	145.27

22	1	−6	115.69	97.33
−22	3	−4	0.00	82.14
−24	5	−8	−25.09	79.90
−12	9	−6	153.45	63.79
39	−26	2	66.82	81.09
27	−25	4	0.00	75.67
36	−23	2	−0.13	95.48
−16	−18	2	0.00	97.46
29	−14	1	54.01	78.97
30	−12	0	27.07	85.05
35	−9	−4	−14.26	80.16
35	−8	−5	−53.09	85.97
−19	−7	0	143.15	82.54
−18	−6	0	195.85	80.82
−22	−6	−1	83.73	78.31
−17	−5	0	50.58	89.27
28	−4	−4	−38.83	101.55
−26	−1	−4	−74.75	107.50
24	0	−6	48.99	95.48
−25	0	−4	0.00	90.20
−14	3	−2	527.32	88.35
16	4	−6	37.51	68.41
12	5	−5	160.32	68.14
−21	5	−5	30.64	75.01
−16	6	−4	67.88	77.92
−21	6	−6	41.86	76.86
−22	6	−7	−69.99	97.20
32	−29	4	−53.35	78.05
3	−29	5	−10.17	48.73
−11	−20	3	−26.41	90.46
26	−19	3	81.75	76.86
−20	−18	1	68.14	88.08
26	−15	2	95.35	78.58
38	−14	−2	29.71	81.09
−22	−13	0	0.00	87.16
35	−10	−3	−4.36	88.88
33	−10	−2	193.07	107.23
29	−9	−1	7.92	81.61
14	−9	2	−16.90	62.20
−27	−6	−3	57.31	96.80
−21	−5	−1	−17.30	82.41
28	−3	−5	4.89	95.35
−18	−2	−1	124.80	89.93
12	3	−3	59.82	78.05
−26	3	−8	18.62	82.67
−25	4	−8	0.00	81.22
−9	6	−3	144.74	52.03
−12	7	−4	89.27	73.03
−16	8	−6	21.92	71.18
−18	8	−8	−51.64	96.40
35	−42	5	−44.50	70.39
39	−37	4	−1.85	75.67
−11	−35	3	−7.79	74.22
−6	−34	4	−28.66	74.35

35	−32	4	0.00	77.92
−12	−30	3	64.31	80.43
−12	−24	3	−57.18	78.05
39	−22	1	−7.13	89.41
−26	−9	−2	−0.79	93.90
28	−5	−3	16.11	90.20
−20	−4	−1	110.67	90.99
−2	−3	1	1392.85	122.55
−19	−3	−1	32.22	78.31
−10	4	−2	954.80	97.46
−22	4	−5	6.60	83.07
34	−40	5	−94.82	71.45
2	−36	5	11.49	57.58
30	−35	5	−3.30	68.01
2	−33	5	12.55	53.22
36	−27	3	7.13	79.37
−12	−27	3	73.03	75.14
−20	−21	1	−49.92	81.75
36	−14	−1	0.00	97.86
−18	−13	1	22.98	83.73
−24	−11	−1	−42.00	97.33
30	−5	−4	−47.81	102.88
−10	−5	1	245.50	71.05
26	−1	−6	0.00	88.22
−27	−1	−5	−48.33	95.61
−3	0	0	9660.13	784.84
−12	1	−1	53.09	61.80
14	5	−6	94.69	66.29
−22	5	−6	26.28	82.80
−23	5	−7	34.60	98.78
−16	7	−5	66.03	68.01
−17	8	−7	30.37	75.01
38	−35	4	−10.04	79.50
39	−30	3	67.48	79.10
−6	−28	4	10.83	69.46
29	−26	4	35.52	87.56
10	−25	5	−14.00	27.86
33	−24	3	−78.58	90.07
−5	−24	4	0.00	82.01
−16	−21	2	0.00	82.14
28	−20	3	15.05	79.63
36	−19	1	2.91	89.01
37	−17	0	−83.99	91.52
−15	−17	2	−41.86	90.59
28	−16	2	9.77	78.84
31	−15	1	36.98	83.33
32	−13	0	71.05	94.56
7	−7	2	7162.33	584.90
−26	−5	−3	−46.62	102.22
30	−4	−5	−19.28	85.97
−26	0	−5	−45.03	117.01
−18	3	−3	92.31	86.24
−23	3	−5	−86.37	82.54
−24	4	−7	0.00	90.73

−12	8	−5	170.09	66.95
−14	9	−8	−14.00	71.05
33	−38	5	−37.37	67.48
−6	−31	4	67.75	72.63
4	−28	5	16.38	44.77
13	−25	5	0.00	42.66
−10	−19	3	3.57	74.22
31	−10	−1	56.79	88.74
−6	−8	2	154.12	56.26
−25	−8	−2	199.54	106.97
30	−6	−3	87.16	97.06
28	−2	−6	−23.90	85.05
−27	0	−6	1.72	87.16
−25	1	−5	13.34	106.84
−8	2	−1	1212.72	114.36
−24	2	−5	−3.83	98.91
−26	2	−7	16.77	80.95
−25	3	−7	−72.90	91.12
−23	4	−6	70.78	96.67
10	5	−4	67.09	69.20
−13	9	−7	−53.75	64.05
22	−28	5	−9.90	64.58
20	−27	5	−37.90	63.65
38	−24	2	33.28	86.10
−16	−24	2	−10.83	82.01
22	−21	4	17.70	65.24
20	−20	4	71.97	66.03
1	−18	4	53.48	39.88
−19	−17	1	74.75	90.86
−17	−12	1	76.33	78.44
37	−11	−3	69.33	83.73
35	−11	−2	−37.77	97.06
23	−7	0	64.71	81.22
21	−6	0	7.66	92.31
19	−5	0	151.74	93.10
−25	−4	−3	−80.43	111.99
−26	1	−6	−0.40	98.91
−25	2	−6	105.12	100.63
−24	3	−6	−14.79	102.74
−5	−36	4	0.00	73.16
29	−33	5	27.20	68.01
−11	−32	3	−0.53	75.80
34	−30	4	6.21	76.33
−11	−23	3	−20.21	83.20
24	−22	4	−32.62	69.20
35	−21	2	−3.70	94.03
18	−19	4	−18.49	64.45
11	−17	4	82.80	48.47
−22	−16	0	12.41	83.73
−4	−13	3	86.24	66.69
−23	−10	−1	−15.98	104.33
22	−9	1	36.71	85.84
20	−8	1	171.68	95.61
12	−8	2	149.49	57.58

32	−6	−4	−52.30	88.88
9	−4	1	816.14	86.90
−15	2	−2	319.19	86.76
−19	2	−3	42.00	74.75
−17	5	−4	28.79	74.75
10	6	−5	26.15	59.43
−19	7	−8	32.09	91.78
3	−38	5	44.77	58.24
32	−36	5	36.45	67.48
37	−33	4	−23.51	75.14
24	−29	5	72.63	70.26
38	−28	3	−44.24	80.95
31	−27	4	−16.90	82.67
18	−26	5	15.45	58.77
30	−21	3	0.00	92.44
−9	−18	3	−1.06	69.20
30	−17	2	−42.52	86.63
33	−16	1	−38.03	100.50
−14	−16	2	30.37	77.92
38	−15	−1	48.07	89.67
34	−14	0	53.48	101.16
25	−8	0	111.46	81.09
18	−7	1	432.63	93.37
−11	−6	1	347.32	82.67
32	−5	−5	146.72	85.84
17	−4	0	373.47	85.31
30	−3	−6	37.37	82.41
−24	−3	−3	−20.87	90.73
−11	−2	0	101.03	58.37
4	2	−1	867.38	86.90
12	6	−6	−24.83	60.35
5	8	−6	100.23	46.75
36	−41	5	68.80	70.12
35	−25	3	121.23	86.63
−4	−23	4	19.15	73.82
38	−20	1	−18.36	96.54
−19	−20	1	−1.19	79.90
33	−11	−1	31.83	99.18
−20	−11	0	−11.49	83.59
32	−7	−3	−28.66	96.67
−2	−7	2	625.57	62.20
−24	−7	−2	17.96	101.16
−23	−2	−3	−15.72	79.50
8	−1	0	704.81	82.67
−20	1	−3	62.33	80.29
10	4	−3	512.00	82.27
−17	7	−6	99.05	75.14
−9	9	−6	17.17	54.28
26	−30	5	0.13	69.46
−11	−29	3	−51.11	80.69
−11	−26	3	−5.94	77.65
26	−23	4	−23.11	69.99
−19	−23	1	1.06	81.61
−15	−20	2	21.39	89.14

39	−18	0	96.40	87.69
26	−11	1	57.84	82.41
27	−9	0	−30.11	84.78
16	−6	1	51.50	74.61
22	−4	−1	−35.00	77.26
−22	−1	−3	46.22	79.24
−21	0	−3	27.86	81.61
9	1	−1	−17.17	51.90
−17	6	−5	50.05	72.24
−18	7	−7	11.36	78.44
2	9	−7	81.61	29.32
35	−39	5	−16.64	68.41
−10	−34	3	8.85	78.05
−5	−33	4	48.86	73.82
−15	−29	2	0.00	80.43
16	−18	4	−25.88	57.05
−8	−17	3	112.38	68.14
−18	−16	1	0.00	100.89
−13	−15	2	21.00	71.97
−5	−14	3	153.32	62.46
37	−12	−2	14.26	84.78
−15	−10	1	48.07	78.31
−22	−9	−1	5.55	88.22
34	−7	−4	62.46	90.59
−12	−7	1	311.66	82.67
−23	−6	−2	−21.39	86.50
24	−5	−1	78.58	80.03
−26	−4	−4	−14.00	106.57
20	−3	−1	31.69	87.82
15	−3	0	407.01	80.69
−13	0	−1	245.90	75.94
31	−34	5	0.13	68.54
3	−32	5	8.85	52.03
36	−31	4	−72.90	78.84
33	−28	4	46.22	80.69
16	−25	5	−18.75	50.58
28	−24	4	−17.83	92.57
37	−22	2	−84.12	87.69
32	−22	3	−59.96	96.93
−3	−22	4	18.75	59.82
32	−18	2	−2.38	95.74
35	−17	1	−71.97	97.86
23	−16	3	149.76	76.86
21	−15	3	154.91	71.18
−21	−15	0	98.65	100.23
28	−12	1	22.05	84.25
−25	−11	−2	−6.74	90.99
29	−10	0	36.85	77.65
−19	−10	0	120.97	84.39
−7	−9	2	230.31	66.56
−14	−9	1	−6.87	73.95
34	−8	−3	11.09	89.01
−13	−8	1	123.48	89.54
−26	−8	−3	−82.67	95.08

34	−6	−5	−17.17	82.27
32	−4	−6	0.00	82.93
−6	−4	1	1759.71	153.59
18	−2	−1	163.89	100.76
−16	1	−2	327.51	93.10
−18	4	−4	140.25	75.67
0	6	−3	282.48	28.92
−6	6	−3	22.05	37.51
−20	6	−8	18.62	87.69
7	8	−7	28.92	48.47
34	−37	5	−72.24	70.78
3	−35	5	−0.13	55.86
−5	−30	4	−38.43	71.71
−15	−26	2	145.14	84.25
−15	−23	2	9.90	78.31
−10	−22	3	33.02	90.20
0	−19	4	135.23	47.15
25	−17	3	3.04	74.75
−7	−16	3	186.73	66.43
36	−15	0	−12.02	96.67
−6	−15	3	58.11	58.24
35	−12	−1	20.34	107.63
26	−6	−1	0.00	81.09
23	0	−4	95.08	75.54
21	1	−4	−7.79	72.37
−9	7	−4	75.01	59.56
39	−34	4	72.37	75.54
40	−29	3	−69.99	83.86
37	−26	3	10.17	82.54
−2	−21	4	46.88	54.67
−12	−14	2	91.78	73.29
−23	−13	−1	32.22	89.80
23	−12	2	216.32	78.18
21	−11	2	51.11	80.29
−21	−8	−1	62.33	81.09
−22	−5	−2	66.82	83.07
−12	−3	0	493.91	83.07
−25	−3	−4	−41.73	106.18
25	−1	−4	38.03	83.20
19	2	−4	126.51	73.16
−13	6	−4	161.91	80.16
−13	8	−6	−47.81	66.16
−9	−36	3	−2.64	79.10
−1	−20	4	8.19	50.32
−14	−19	2	−31.96	95.08
−18	−19	1	−1.72	87.16
27	−18	3	−15.32	77.52
−21	−18	0	−5.68	87.03
−17	−15	1	43.71	85.31
30	−13	1	47.41	80.69
25	−13	2	44.24	82.93
31	−11	0	53.35	84.52
19	−10	2	225.69	89.27
−18	−9	0	47.94	80.43

36	−8	−4	32.49	78.84
28	−7	−1	64.71	81.22
34	−5	−6	36.18	82.41
14	−5	1	−45.16	63.39
27	−2	−4	−33.15	95.61
25	−2	−3	2.91	76.46
−24	−2	−4	44.50	94.42
23	−1	−3	66.03	79.24
16	−1	−1	250.65	87.82
21	0	−3	438.31	86.63
−17	0	−2	527.32	107.63
23	1	−5	17.83	88.88
21	2	−5	94.82	80.16
−19	3	−4	119.52	76.73
−18	5	−5	91.91	76.99
−21	5	−8	−76.73	89.27
−18	6	−6	28.26	71.97
−19	6	−7	12.41	84.12
−15	8	−8	−39.35	87.29
38	−42	5	−27.60	72.24
−10	−31	3	−51.64	80.29
30	−25	4	−33.41	90.59
34	−23	3	56.26	90.46
−11	−13	2	118.72	70.26
−20	−7	−1	4.75	80.03
−25	−7	−3	0.00	107.76
−21	−4	−2	57.71	81.35
4	−3	1	642.21	66.69
−7	−1	0	699.66	78.44
−14	−1	−1	340.19	86.10
25	0	−5	9.11	96.01
19	1	−3	60.62	74.61
19	3	−5	84.25	72.11
17	3	−4	76.60	72.77
−11	3	−2	421.67	73.82
−22	4	−8	−27.07	84.78
−9	8	−5	184.75	67.09
37	−40	5	−44.77	72.24
33	−35	5	92.97	73.16
−4	−35	4	58.11	71.84
30	−32	5	15.85	72.63
35	−29	4	32.75	77.65
−10	−25	3	−19.41	79.24
−18	−22	1	0.13	82.27
37	−18	1	−58.11	88.88
14	−17	4	207.60	56.65
27	−14	2	20.47	78.71
−20	−14	0	95.48	92.57
37	−13	−1	−8.32	93.90
−24	−10	−2	43.71	100.76
36	−9	−3	23.11	85.58
34	−9	−2	0.00	89.54
30	−8	−1	154.25	92.57
−17	−8	0	76.46	83.20

−13	−4	0	424.71	85.31
27	−3	−3	−48.20	84.78
−20	−3	−2	55.99	85.44
13	−2	0	138.27	62.20
−19	−2	−2	66.29	76.20
27	−1	−5	−7.13	96.27
−18	−1	−2	74.75	83.86
−23	−1	−4	−64.97	94.42
−20	2	−4	−21.66	77.65
−24	2	−8	0.00	82.54
−23	3	−8	−47.41	83.59
−7	4	−2	1873.81	161.77
8	6	−4	0.00	65.24
−13	7	−5	152.13	69.20
−14	8	−7	24.30	64.45
−10	9	−7	−10.30	55.73
38	−32	4	0.00	80.69
23	−27	5	−5.55	68.80
−4	−26	4	23.90	74.48
39	−23	2	11.49	82.80
29	−19	3	0.00	84.25
38	−16	0	−77.52	87.29
32	−14	1	117.67	94.42
−10	−12	2	−7.66	67.75
−22	−12	−1	−5.15	106.57
17	−9	2	102.08	82.41
−19	−6	−1	−14.92	82.27
29	−3	−4	75.14	105.78
−25	−2	−5	28.00	105.65
−26	−2	−6	0.00	90.73
17	2	−3	141.17	69.86
17	4	−5	177.75	71.84
−20	5	−7	−22.45	97.86
10	7	−6	304.40	64.05
−6	9	−6	0.00	39.88
36	−38	5	−49.39	74.75
25	−28	5	67.35	71.05
39	−27	3	9.38	82.80
32	−26	4	16.11	83.99
21	−26	5	11.36	67.35
−14	−22	2	−0.26	86.63
−9	−21	3	−9.90	86.50
29	−15	2	108.69	85.84
33	−12	0	21.00	98.78
10	−7	2	3121.66	262.80
−16	−7	0	228.20	80.82
−15	−6	0	−4.09	88.22
−24	−6	−3	85.84	107.89
−14	−5	0	175.24	99.18
29	−4	−3	−51.11	98.25
29	−2	−5	56.92	87.82
−15	−2	−1	179.74	90.99
−19	4	−5	72.77	77.12
−19	5	−6	157.55	77.52

4	−37	5	0.00	56.92
−4	−32	4	31.30	72.37
36	−24	3	26.81	83.33
23	−20	4	51.37	66.82
−13	−18	2	32.09	83.99
−16	−14	1	27.20	74.48
36	−10	−2	−94.42	93.37
32	−9	−1	73.43	106.04
−18	−5	−1	37.51	84.92
31	−4	−4	57.71	92.97
−16	−3	−1	178.41	97.46
−24	−1	−5	−7.00	109.74
−25	−1	−6	17.96	98.78
14	0	−1	1626.07	158.87
−25	0	−7	0.00	90.07
23	2	−6	38.83	97.99
15	4	−4	73.95	68.14
−21	4	−7	−2.38	97.20
3	7	−4	1470.63	130.21
−3	−37	4	75.80	74.09
32	−33	5	76.99	75.01
−9	−33	3	−8.32	80.82
4	−31	5	0.00	51.24
−4	−29	4	0.00	63.65
19	−25	5	1.72	57.71
−14	−25	2	−2.91	79.76
25	−21	4	88.08	72.77
31	−20	3	25.49	107.50
39	−19	1	−7.13	85.97
21	−19	4	77.92	70.26
−17	−18	1	64.97	98.39
−20	−17	0	10.04	91.25
34	−15	1	−31.17	105.25
−23	−9	−2	82.27	103.27
31	−5	−3	−91.25	101.55
−17	−4	−1	232.82	87.56
−23	0	−5	156.10	96.01
25	1	−6	−1.06	89.80
−9	1	−1	20.60	49.79
−24	1	−7	−75.80	90.20
−23	2	−7	−44.37	99.18
21	3	−6	58.24	93.50
−15	3	−3	24.96	82.01
−20	3	−5	0.00	74.75
−22	3	−7	0.00	107.23
−20	4	−6	13.47	79.37
−10	5	−3	605.63	75.54
8	7	−5	167.72	55.86
35	−36	5	79.37	72.77
37	−30	4	−76.99	82.93
27	−22	4	−91.12	86.76
31	−16	2	−52.69	93.10
35	−13	0	77.65	102.61
−23	−5	−3	−37.90	90.59

31	-3	-5	102.35	89.67
27	0	-6	28.13	83.99
19	0	-2	203.77	76.20
3	0	0	9687.47	786.95
-24	0	-6	0.00	106.44
-22	1	-5	19.02	80.29
-5	2	-1	1125.95	109.48
-21	2	-5	73.69	78.44
19	4	-6	151.87	90.86
8	5	-3	387.60	69.60
-16	7	-8	28.79	92.57
39	-41	5	-67.88	82.14
34	-27	4	-27.60	79.37
14	-24	5	47.28	44.77
-20	-20	0	0.00	86.63
19	-18	4	36.05	64.45
-15	-13	1	23.64	82.14
-21	-11	-1	193.73	97.46
34	-10	-1	-22.45	100.37
15	-8	2	70.92	66.43
-25	-6	-4	-53.48	102.74
33	-5	-4	0.00	83.59
-7	-5	1	813.63	86.37
12	-4	1	218.96	58.37
-23	1	-6	28.26	106.57
-22	2	-6	-18.09	98.12
15	3	-3	-16.90	76.99
-21	3	-6	0.00	84.25
15	5	-5	40.54	65.90
-14	5	-4	231.11	87.56
4	-34	5	-66.43	55.47
-9	-30	3	-59.43	84.12
-3	-25	4	46.88	82.54
-9	-24	3	50.45	84.52
-17	-24	1	-73.03	85.05
29	-23	4	-34.07	94.16
38	-21	2	52.43	85.18
33	-21	3	25.49	98.78
-17	-21	1	14.79	84.25
-8	-20	3	-24.83	69.99
25	-9	1	0.79	84.92
23	-8	1	129.55	83.73
26	-7	0	155.96	83.20
33	-6	-3	-69.99	91.65
24	-6	0	67.35	82.67
22	-5	0	101.29	83.99
33	-4	-5	77.26	93.50
29	-1	-6	1.58	83.07
38	-39	5	65.63	81.35
-8	-35	3	86.90	81.61
34	-34	5	-7.40	73.16
-13	-30	2	14.00	86.76
38	-25	3	56.39	86.10
33	-17	2	4.23	103.80

−12	−17	2	40.81	74.48
36	−16	1	−30.51	93.50
0	−12	3	681.17	70.65
−18	−12	0	68.54	80.56
27	−10	1	−12.68	84.52
−22	−8	−2	132.33	91.65
21	−7	1	991.78	130.08
20	−4	0	−3.43	93.90
−22	−4	−3	−27.07	81.88
17	1	−2	98.78	88.61
−16	2	−3	19.02	99.05
17	5	−6	−54.01	76.73
−14	7	−6	518.47	84.92
3	8	−5	189.24	44.50
−3	9	−6	14.00	25.62
5	−39	5	−1.32	59.43
−9	−27	3	35.39	76.99
−13	−21	2	36.32	95.48
37	−14	0	22.58	91.12
28	−8	0	43.71	80.56
35	−6	−4	−29.58	82.67
7	−3	1	2720.59	230.58
31	−2	−6	51.24	81.75
11	−1	0	493.12	71.71
−12	2	−2	184.49	67.09
−15	7	−7	120.84	69.99
37	−37	5	0.00	78.97
−3	−34	4	−41.73	75.54
39	−31	4	−131.14	86.50
36	−28	4	93.37	83.46
31	−24	4	−4.09	88.08
17	−24	5	22.05	53.35
−16	−17	1	−21.66	95.88
26	−16	3	151.21	79.76
−19	−16	0	−80.69	99.31
−23	−12	−2	14.79	102.35
36	−11	−1	98.78	101.16
29	−11	1	−23.24	77.65
−20	−10	−1	30.90	81.61
−24	−9	−3	−17.96	107.23
19	−6	1	84.78	96.80
−24	−5	−4	5.94	104.06
−21	−3	−3	40.15	80.69
7	2	−1	1463.76	131.66
13	5	−4	30.24	64.58
−3	6	−3	596.92	55.33
−14	6	−5	−1.98	65.50
−7	−37	3	18.62	81.61
−13	−27	2	22.32	82.67
−13	−24	2	−24.17	82.93
35	−22	3	8.72	95.35
35	−18	2	−90.59	107.37
28	−17	3	6.87	83.59
17	−17	4	264.91	65.24

30	−9	0	9.24	80.16
35	−7	−3	−4.62	86.37
−21	−7	−2	26.81	86.63
18	−3	0	60.22	87.56
−20	−2	−3	63.13	86.76
12	1	−1	520.58	79.63
−17	6	−8	16.11	93.90
24	−26	5	94.42	74.48
−7	−19	3	89.01	68.14
38	−17	1	132.59	88.88
−21	−14	−1	5.55	101.69
31	−12	1	−82.14	90.86
−17	−11	0	95.88	80.16
27	−5	−1	98.12	82.27
25	−4	−1	236.79	86.90
33	−3	−6	110.80	85.31
23	−3	−1	92.97	76.46
−8	−2	0	38.69	49.65
−19	−1	−3	51.64	83.86
−18	0	−3	98.12	75.27
13	4	−3	153.19	79.50
−6	7	−4	108.55	46.75
23	−40	6	−16.64	54.94
−6	−39	3	0.00	85.71
36	−35	5	22.45	79.63
33	−32	5	−36.32	74.88
−8	−32	3	−12.41	81.09
−12	−32	2	0.00	86.76
−3	−31	4	−32.75	70.65
5	−30	5	−38.03	48.60
26	−27	5	75.54	72.77
40	−26	3	13.34	85.18
33	−25	4	13.87	85.71
22	−25	5	57.58	68.94
11	−24	5	37.90	28.39
−16	−20	1	0.00	89.93
−19	−19	0	−13.87	86.90
30	−18	3	136.68	95.08
12	−16	4	203.77	55.20
26	−12	2	42.13	84.25
24	−11	2	61.01	71.05
−13	−11	1	40.15	68.28
32	−10	0	16.77	102.88
22	−10	2	41.86	77.26
−3	−8	2	578.82	63.26
−8	−6	1	193.21	58.50
−23	−4	−4	25.36	97.99
21	−2	−1	66.69	78.18
15	2	−2	44.90	84.12
−15	4	−4	0.00	82.54
13	6	−5	27.20	65.37
−10	8	−6	−33.68	56.92
−12	8	−8	49.13	70.92
−7	9	−7	71.84	49.52

28	−28	5	0.00	72.63
−16	−26	1	83.46	85.84
−2	−24	4	35.66	70.52
−8	−23	3	56.52	91.39
28	−13	2	62.60	82.27
38	−12	−1	53.75	89.41
−19	−9	−1	12.68	82.80
−23	−8	−3	46.62	109.61
13	−7	2	116.35	57.45
29	−6	−1	0.00	82.93
−20	−6	−2	65.24	87.69
17	−5	1	544.49	95.08
6	0	0	2918.28	248.01
−10	0	−1	202.98	55.86
−18	5	−8	0.00	100.89
15	6	−6	39.88	68.41
−6	8	−5	234.01	56.39
39	−38	5	17.17	84.52
−2	−36	4	16.24	73.56
38	−29	4	−10.96	84.65
37	−23	3	42.52	90.46
−16	−23	1	21.79	84.92
−12	−20	2	37.77	93.76
−21	−17	−1	0.00	88.61
9	−16	4	132.99	42.79
−15	−16	1	92.97	81.35
−18	−15	0	−59.82	101.55
30	−14	2	0.00	83.73
33	−13	1	78.05	101.55
−22	−11	−2	−21.13	109.61
−12	−10	1	87.16	84.52
20	−9	2	54.41	87.95
31	−7	−1	37.77	94.69
−22	−3	−4	17.56	84.78
19	−1	−1	−52.03	86.90
26	0	−4	36.05	99.84
−23	0	−8	25.09	89.54
24	1	−4	−46.88	89.80
−13	1	−2	272.71	74.09
−10	6	−4	102.22	64.84
−15	6	−6	113.31	73.43
−16	6	−7	89.41	75.54
26	−40	6	24.30	62.73
24	−39	6	0.00	57.18
21	−39	6	−16.51	45.16
5	−36	5	−20.73	58.90
30	−29	5	−12.41	77.12
20	−24	5	9.77	63.13
26	−20	4	29.98	76.46
32	−19	3	0.00	98.91
24	−19	4	97.86	69.20
−6	−18	3	63.79	66.69
34	−11	0	12.02	106.57
−16	−10	0	76.73	82.93

−9	−7	1	342.43	70.52
16	−2	0	216.05	79.63
28	−1	−4	−18.49	103.14
26	−1	−3	−22.71	84.65
24	0	−3	66.56	81.61
22	2	−4	−12.28	78.05
−19	4	−8	22.71	96.67
−15	5	−5	−62.33	68.41
3	6	−3	69.46	35.79
8	8	−6	31.56	52.03
−11	8	−7	74.09	62.33
20	−40	6	−41.47	16.90
35	−33	5	37.37	80.56
35	−26	4	12.15	82.80
−8	−26	3	0.40	82.67
28	−21	4	−14.92	91.78
−10	−15	2	121.63	73.03
−20	−13	−1	33.02	115.69
−11	−9	1	326.72	89.80
−10	−8	1	58.11	73.82
−18	−8	−1	82.14	85.58
−19	−5	−2	58.77	82.67
−24	−3	−6	0.00	102.22
30	−2	−4	−69.33	100.50
28	−2	−3	−19.28	100.50
−21	−2	−4	−17.43	79.24
−24	−2	−7	0.00	88.48
26	1	−5	60.48	101.69
22	1	−3	18.49	80.29
−22	1	−8	22.85	85.84
−21	2	−8	−19.28	90.33
−16	3	−4	157.94	82.54
−20	3	−8	16.77	89.93
22	−38	6	82.54	55.07
38	−36	5	−74.35	83.33
−7	−34	3	3.83	82.41
−12	−23	2	75.27	87.56
22	−18	4	−24.17	70.52
15	−16	4	38.96	56.39
32	−15	2	95.48	100.50
35	−14	1	−37.24	104.46
33	−8	−1	128.89	104.72
−24	−8	−4	−5.02	102.22
−22	−7	−3	48.99	91.12
30	−3	−3	5.68	104.72
−23	−3	−5	21.13	107.76
28	0	−5	−58.37	94.69
−14	0	−2	1580.90	157.94
24	2	−5	−14.92	98.65
20	3	−4	15.05	72.24
−10	7	−5	241.01	73.56
−1	−38	4	17.56	75.94
5	−33	5	10.30	53.35
−12	−26	2	26.54	82.67

39	−24	3	0.00	84.52
30	−22	4	−66.29	97.33
34	−20	3	43.98	100.89
−18	−18	0	68.54	95.48
36	−12	0	−82.80	102.74
−15	−9	0	15.98	74.61
18	−8	2	62.07	78.97
−18	−4	−2	46.49	78.71
32	−3	−4	17.83	92.18
10	−3	1	1090.56	104.46
−9	−3	0	518.87	70.26
30	−1	−5	15.72	87.42
−20	−1	−4	27.60	90.99
−23	−1	−7	−51.77	93.90
17	0	−1	379.81	95.22
−17	2	−4	0.00	73.03
22	3	−5	52.30	92.31
−17	5	−7	−38.43	79.24
27	−39	6	−62.60	66.56
25	−38	6	0.00	62.07
−2	−33	4	1.72	72.77
40	−30	4	0.00	84.65
37	−27	4	95.08	85.31
15	−23	5	28.66	47.15
−1	−23	4	0.00	58.90
−15	−19	1	0.00	104.72
20	−17	4	6.87	67.09
−5	−17	3	7.92	61.94
34	−16	2	−6.21	106.04
−14	−15	1	31.30	75.01
−22	−14	−2	−35.52	106.84
−1	−13	3	126.91	51.50
−21	−10	−2	−22.58	93.63
35	−9	−1	24.56	99.44
−17	−7	−1	157.15	82.93
32	−4	−3	−22.71	102.22
15	−4	1	152.53	67.09
−17	−3	−2	131.66	82.14
−22	−2	−5	38.30	108.42
−23	−2	−6	−77.12	109.08
−15	−1	−2	351.81	97.06
20	2	−3	187.79	78.18
−16	4	−5	97.86	73.16
−16	5	−6	82.67	72.37
11	6	−4	70.26	61.94
23	−37	6	1.06	56.26
37	−34	5	0.00	83.33
−2	−27	4	0.40	74.61
32	−23	4	0.00	95.35
−7	−22	3	20.47	80.43
−18	−21	0	−1.85	88.61
−11	−19	2	95.61	80.29
37	−15	1	−24.30	98.12
−9	−14	2	31.30	66.29

−23	−11	−3	0.00	101.16
28	−3	−2	93.37	91.52
32	−2	−5	−48.07	86.37
26	−2	−2	38.43	79.24
−16	−2	−2	0.00	88.88
24	−1	−2	25.62	78.18
−11	−1	−1	35.39	57.84
−22	0	−7	−27.47	98.25
13	3	−2	75.14	76.86
−8	3	−2	742.84	77.12
−18	4	−7	−20.87	85.44
6	7	−4	112.12	58.50
29	−40	6	0.00	66.03
−6	−36	3	27.73	83.99
34	−31	5	−69.07	83.99
−11	−31	2	−0.79	90.46
−15	−25	1	101.03	93.63
18	−23	5	33.28	56.39
−15	−22	1	−32.62	91.78
36	−21	3	−61.67	90.73
38	−13	0	−7.79	92.31
−19	−12	−1	−52.16	92.71
16	−11	3	31.43	76.99
−4	−9	2	66.16	47.67
−14	−8	0	218.43	88.61
−23	−7	−4	0.13	109.08
8	−6	2	1292.08	115.29
−21	−6	−3	−42.66	88.61
34	−4	−4	−37.37	90.99
30	−4	−2	−74.75	111.99
−21	−1	−5	43.45	84.12
−22	−1	−6	30.51	116.48
22	0	−2	190.30	83.46
−21	1	−7	−33.68	106.97
−20	2	−7	34.60	100.89
−19	3	−7	39.62	96.54
18	4	−4	57.71	72.90
13	7	−6	5.15	62.86
−13	7	−8	−1.85	81.09
6	−38	5	54.81	57.18
40	−37	5	4.89	92.31
−7	−31	3	−54.67	86.76
−2	−30	4	−37.90	70.78
6	−29	5	−8.45	33.81
27	−26	5	0.00	71.97
25	−25	5	−4.89	74.22
−20	−19	−1	−8.32	87.82
36	−17	2	0.00	97.33
−4	−16	3	118.99	55.73
28	−9	1	−9.77	81.61
26	−8	1	14.66	82.14
24	−7	1	239.03	90.07
−24	−7	−5	0.00	97.46
27	−6	0	−7.00	80.43

−16	−6	−1	34.60	80.16
34	−5	−3	−88.88	91.12
25	−5	0	93.50	85.84
−3	−4	1	2508.90	210.11
−10	−4	0	1147.35	116.35
34	−3	−5	0.00	95.48
14	−1	0	166.40	69.20
−20	0	−5	−16.90	78.44
−21	0	−6	23.51	107.89
28	1	−6	−1.06	86.10
26	2	−6	−1.32	85.84
10	2	−1	896.03	93.76
24	3	−6	157.28	92.84
18	3	−3	284.33	82.93
−17	3	−5	150.29	77.39
−17	4	−6	−51.24	77.12
11	5	−3	119.52	74.61
11	7	−5	292.38	68.28
5	9	−7	−14.39	16.64
26	−37	6	0.00	62.99
29	−27	5	0.00	77.39
34	−24	4	−48.47	87.82
23	−24	5	97.20	74.09
29	−16	3	−69.60	83.73
−3	−15	3	72.24	58.77
−2	−14	3	63.79	58.77
−8	−13	2	9.24	69.07
37	−10	−1	−14.26	90.07
30	−10	1	67.48	83.59
−20	−9	−2	70.78	82.54
29	−7	0	3.04	82.01
−13	−7	0	66.95	87.16
32	−5	−2	182.51	105.25
−15	−5	−1	58.11	89.27
23	−4	0	268.35	86.90
−12	−2	−1	774.14	99.18
9	0	0	1160.42	110.14
−19	1	−5	−36.18	79.76
−20	1	−6	72.77	95.88
−18	2	−5	53.88	77.52
−18	3	−6	93.37	75.80
−4	4	−2	2720.32	225.03
28	−38	6	−1.72	68.14
−5	−38	3	6.21	87.42
39	−28	4	−3.04	87.16
31	−28	5	−16.51	78.84
−7	−28	3	66.03	82.41
−11	−28	2	62.99	89.14
−7	−25	3	0.00	89.54
38	−22	3	0.00	85.31
−13	−14	1	94.69	81.88
31	−8	0	30.24	97.20
16	−7	2	235.33	80.03
36	−6	−3	14.26	87.56

22	−6	1	15.45	79.76
−12	−6	0	501.83	92.44
−11	−5	0	880.71	105.65
−20	−5	−3	−14.13	78.58
−14	−4	−1	447.95	92.97
−13	−3	−1	169.83	76.73
20	1	−2	126.12	73.03
−19	2	−6	102.61	80.56
22	4	−6	26.41	99.18
18	5	−5	65.37	71.45
30	−39	6	−1.06	69.99
24	−36	6	6.60	59.82
21	−36	6	−71.58	21.53
39	−35	5	−11.23	88.48
−1	−35	4	−46.09	78.44
−10	−33	2	142.89	92.44
36	−32	5	−35.26	85.05
0	−22	4	49.39	53.62
−11	−22	2	7.79	100.89
31	−17	3	15.58	108.29
−17	−17	0	−31.03	102.22
18	−16	4	40.94	63.65
−16	−13	0	29.05	77.65
−21	−13	−2	29.58	109.87
−7	−12	2	114.10	71.45
32	−11	1	−73.56	102.08
−5	−10	2	265.18	61.67
−22	−10	−3	−76.33	120.04
34	−6	−2	−11.09	105.52
−22	−6	−4	65.90	108.03
21	−3	0	140.78	78.58
−21	0	−9	−48.47	90.59
15	1	−1	200.60	84.39
−7	5	−3	247.22	48.73
−11	7	−6	214.86	64.31
6	8	−5	−19.94	45.43
−4	−40	3	32.09	93.90
19	−38	6	101.16	23.77
36	−25	4	−32.88	90.20
−11	−25	2	90.20	88.61
−6	−21	3	68.67	69.07
38	−18	2	−5.02	89.01
−14	−18	1	0.00	88.35
−19	−15	−1	0.00	104.86
29	−12	2	−13.21	78.97
21	−12	3	21.39	75.14
27	−11	2	13.47	84.65
−18	−11	−1	−15.32	85.97
25	−10	2	68.41	74.35
11	−10	3	166.66	55.73
33	−9	0	0.00	111.33
11	−6	2	497.08	69.07
5	−6	2	1172.44	101.82
−23	−6	−5	0.00	103.93

20	−5	1	190.17	95.22
−4	−1	0	775.59	76.20
−20	1	−9	−26.41	91.52
16	5	−4	7.79	69.73
−11	5	−4	310.61	76.86
−12	7	−7	165.21	69.20
7	−40	5	0.00	55.86
−6	−33	3	−35.13	86.50
33	−29	5	52.69	83.33
−14	−27	1	0.26	91.65
21	−23	5	10.96	64.05
−17	−23	0	149.49	90.59
−17	−20	0	0.00	92.71
27	−19	4	0.00	80.43
33	−18	3	0.00	110.80
−10	−18	2	22.19	70.52
31	−13	2	−35.13	93.50
34	−12	1	0.00	104.46
−19	−8	−2	6.21	82.01
28	−4	−1	118.85	94.69
−19	−4	−3	85.31	81.22
26	−3	−1	178.15	89.80
20	5	−6	−38.96	96.67
6	6	−3	445.31	63.26
−14	6	−8	−77.39	90.07
32	−40	6	52.03	75.54
38	−33	5	0.00	87.42
29	−20	4	15.05	97.33
25	−18	4	14.00	71.18
−21	−16	−2	−0.66	103.01
35	−10	0	−30.64	105.25
−23	−10	−4	−67.35	105.65
23	−9	2	31.56	72.50
36	−7	−2	−31.69	94.82
30	−5	−1	119.65	94.82
13	−3	1	163.76	60.62
24	−2	−1	2.38	82.54
18	2	−2	68.01	74.88
16	4	−3	20.87	68.41
29	−37	6	0.00	60.22
27	−36	6	13.47	67.75
22	−35	6	−13.60	56.52
−9	−35	2	0.00	91.25
38	−26	4	−54.15	89.54
−1	−26	4	8.58	82.54
−14	−24	1	91.39	89.27
31	−21	4	16.38	97.86
−14	−21	1	−1.19	99.84
35	−19	3	0.13	95.48
−19	−18	−1	112.38	97.46
23	−17	4	26.02	70.39
33	−14	2	104.59	107.37
36	−13	1	5.81	102.74
32	−6	−1	48.33	118.99

−21	−5	−4	5.68	89.80
−23	−5	−6	−7.92	97.86
−18	−3	−3	150.81	79.63
19	−2	0	50.58	96.01
−22	−2	−8	0.00	89.54
−6	1	−1	847.04	87.42
−13	2	−3	60.62	73.56
−11	6	−5	28.00	70.39
−9	8	−8	64.97	61.94
0	−37	4	−23.64	78.31
25	−35	6	9.38	63.52
6	−35	5	0.00	56.79
1	−21	4	−47.15	48.86
13	−15	4	120.18	58.11
−22	−13	−3	35.26	104.06
−15	−12	0	−33.15	81.09
−20	−12	−2	25.22	102.48
−21	−9	−3	33.02	99.71
21	−8	2	11.36	82.14
22	−1	−1	77.52	79.50
16	6	−5	83.86	70.78
−4	9	−7	25.75	39.75
31	−38	6	−59.96	75.67
6	−32	5	−2.64	52.56
33	−22	4	22.45	90.07
16	−22	5	44.37	52.43
−16	−16	0	−43.45	87.56
35	−15	2	−66.43	105.38
37	−11	0	71.58	98.52
19	−11	3	−6.21	74.48
14	−10	3	473.44	82.41
−17	−10	−1	11.62	79.37
34	−7	−1	57.45	107.50
−18	−7	−2	164.55	86.76
18	−4	1	961.14	124.67
−17	−2	−3	7.26	73.16
−21	−1	−8	9.77	89.54
−14	1	−3	−38.83	87.03
11	4	−2	252.63	70.12
−15	5	−8	−1.98	97.46
18	6	−6	25.36	92.84
−7	8	−6	42.92	48.73
−5	−35	3	−30.64	88.08
−6	−30	3	76.20	80.29
30	−26	5	13.60	81.22
28	−25	5	−20.73	79.50
26	−24	5	73.16	77.78
−6	−24	3	0.00	82.54
19	−22	5	81.61	58.90
−10	−21	2	−46.75	89.14
37	−20	3	0.00	89.01
−13	−17	1	−1.72	77.65
38	−14	1	5.68	94.56
−20	−4	−4	0.00	78.58

−22	−4	−6	21.13	108.03
−22	−3	−7	0.00	96.14
29	−1	−3	−65.11	105.38
−16	−1	−3	−51.90	87.42
29	0	−4	−83.59	98.65
27	0	−3	52.16	104.20
12	0	0	698.07	83.99
−15	0	−3	25.36	84.65
27	1	−4	−53.22	105.12
−9	2	−2	741.26	82.14
33	−39	6	−10.17	78.44
1	−39	4	−60.88	82.93
40	−34	5	−45.96	92.31
23	−34	6	2.11	59.56
37	−31	5	0.00	92.97
−13	−29	1	74.35	92.97
40	−27	4	0.00	91.78
−6	−27	3	47.67	84.78
35	−23	4	−41.86	90.46
−5	−20	3	28.13	64.18
−9	−17	2	54.81	72.50
21	−16	4	−33.81	70.52
16	−15	4	79.10	61.54
−18	−14	−1	−26.68	92.18
−22	−9	−4	46.62	114.76
36	−8	−1	121.63	101.55
14	−6	2	169.83	63.52
31	−2	−3	33.28	99.57
31	−1	−4	63.65	91.65
20	0	−1	0.00	77.65
−20	0	−8	−29.32	94.56
25	1	−3	−43.84	85.97
25	2	−4	13.47	99.31
−12	4	−4	45.96	70.26
−13	6	−7	98.25	71.84
11	8	−6	21.26	59.43
28	−35	6	−62.20	73.95
32	−27	5	68.54	85.05
−10	−27	2	3.83	87.56
−16	−25	0	100.63	94.56
−10	−24	2	58.11	92.31
24	−23	5	11.36	72.77
−16	−19	0	−37.37	112.25
37	−16	2	185.28	104.86
−20	−15	−2	−17.83	104.46
−11	−12	1	131.66	73.95
−20	−8	−3	145.14	86.50
19	−7	2	108.42	90.73
33	−3	−3	−7.53	94.03
33	−2	−4	54.54	90.73
17	−1	0	32.88	82.14
29	1	−5	−7.00	90.07
−19	1	−8	−43.58	96.54
27	2	−5	0.00	96.67

13	2	−1	435.01	76.20
23	3	−4	−44.77	87.03
16	3	−2	213.15	86.37
−16	4	−8	−2.91	100.50
14	6	−4	53.75	68.14
−12	6	−6	127.17	67.75
9	7	−4	138.14	67.09
−8	8	−7	45.96	56.79
3	9	−6	−69.33	21.79
−4	−37	3	−25.49	89.01
30	−36	6	10.96	74.61
26	−34	6	−8.06	68.01
−9	−32	2	−27.47	92.84
−16	−22	0	19.81	90.33
39	−21	3	0.00	89.80
2	−20	4	94.82	45.03
−14	−11	0	−4.49	79.24
−16	−9	−1	72.63	78.18
−17	−6	−2	38.56	80.82
−19	−3	−4	40.28	87.69
−21	−2	−7	−16.90	99.44
31	0	−5	−37.64	90.20
23	2	−3	24.56	79.24
−18	2	−8	47.94	98.65
25	3	−5	77.92	103.27
−17	3	−8	86.24	108.69
32	−37	6	3.83	77.26
34	−28	5	−42.26	89.01
−13	−26	1	14.26	95.61
37	−24	4	−80.82	89.67
−13	−20	1	0.13	102.35
32	−16	3	97.99	104.86
−21	−12	−3	−41.47	110.14
−19	−11	−2	0.00	86.90
31	−9	1	0.00	92.31
29	−8	1	1.72	85.05
27	−7	1	−54.54	85.71
35	−4	−3	−41.86	92.05
35	−3	−4	33.68	92.44
31	−3	−2	21.26	106.84
−21	−3	−6	110.93	114.63
29	−2	−2	−3.43	99.57
33	−1	−5	−15.32	93.37
27	−1	−2	13.34	90.59
14	5	−3	115.29	72.37
−12	5	−5	156.10	72.24
19	−35	6	0.00	46.22
20	−34	6	−5.15	51.50
0	−34	4	−22.05	77.39
39	−32	5	−33.41	94.16
−13	−23	1	−15.19	94.03
22	−22	5	62.20	69.86
−18	−20	−1	131.66	94.82
39	−17	2	−3.17	102.74

10	−15	4	7.53	49.52
24	−12	3	80.16	73.16
17	−10	3	277.99	84.92
30	−6	0	−6.07	97.06
25	−6	1	0.00	88.08
28	−5	0	49.39	85.97
33	−4	−2	50.71	100.63
26	−4	0	−31.43	81.48
−20	−3	−5	−12.94	94.42
−20	−1	−7	5.94	108.95
25	0	−2	−14.53	84.39
23	4	−5	−82.67	103.27
21	4	−4	52.56	76.33
−14	5	−7	−23.64	68.54
9	6	−3	160.59	65.90
−3	−39	3	33.28	96.67
7	−37	5	−26.28	64.18
24	−33	6	−4.75	63.65
−5	−32	3	−70.26	89.27
0	−25	4	3.70	71.05
34	−17	3	−48.73	100.37
−12	−16	1	−8.85	73.16
19	−15	4	50.05	64.84
−17	−13	−1	62.20	81.35
−10	−11	1	332.40	84.12
33	−10	1	101.55	105.78
−21	−8	−4	−48.33	111.33
−22	−8	−5	−4.89	110.80
32	−7	0	−52.43	111.99
−4	−5	1	793.95	77.26
−16	−5	−2	249.07	83.73
16	−3	1	228.07	77.12
−18	−2	−4	85.18	85.31
18	1	−1	−38.17	87.03
21	3	−3	111.72	79.37
5	3	−1	94.16	39.88
−13	3	−4	284.72	80.56
−7	6	−4	107.63	49.79
16	7	−6	−11.49	79.50
14	7	−5	172.08	69.07
−2	−41	3	36.85	101.16
18	−39	6	0.00	16.51
34	−38	6	0.00	79.76
39	−25	4	130.74	96.67
32	−20	4	−123.74	99.84
30	−19	4	89.41	101.16
3	−19	4	169.83	40.41
28	−18	4	44.90	90.73
26	−17	4	10.43	77.65
−8	−16	2	49.79	66.95
35	−11	1	17.43	104.86
30	−11	2	0.00	86.10
28	−10	2	98.25	79.24
34	−8	0	−21.39	112.91

−15	−8	−1	56.13	76.33
−19	−7	−3	74.88	86.50
35	−5	−2	76.46	95.08
23	−5	1	53.35	84.39
24	−3	0	92.57	85.84
8	−2	1	1198.32	111.72
−20	−2	−6	20.07	103.93
−19	0	−7	−8.06	111.06
23	1	−2	47.81	85.44
−10	1	−2	87.03	53.35
−13	5	−6	64.71	70.39
9	8	−5	10.56	54.41
29	−34	6	32.49	75.67
−8	−34	2	119.78	97.46
21	−33	6	0.00	56.92
−9	−29	2	−62.60	92.05
13	−22	5	46.62	42.26
−9	−20	2	6.07	76.33
−4	−19	3	98.52	63.26
36	−18	3	−36.45	96.93
32	−12	2	−15.72	115.03
22	−11	3	0.00	69.86
26	−9	2	132.06	91.39
−19	−2	−5	36.32	78.58
−20	−2	−9	22.32	94.16
−14	2	−4	52.82	82.41
−15	4	−7	−61.14	76.20
21	5	−5	−74.75	96.67
−7	7	−5	1133.61	112.12
−10	7	−8	0.00	68.41
−3	8	−5	134.70	38.03
31	−35	6	39.62	78.71
27	−33	6	0.00	70.78
38	−30	5	0.00	99.71
31	−25	5	−31.83	85.05
29	−24	5	29.58	87.16
−5	−23	3	69.73	76.20
34	−21	4	66.16	96.27
4	−18	4	113.70	29.58
24	−16	4	2.77	74.35
−19	−14	−2	88.88	109.87
34	−13	2	−30.90	107.37
37	−12	1	−39.35	97.99
−20	−11	−3	0.00	108.82
−9	−10	1	484.93	84.52
−18	−10	−2	19.41	85.84
36	−9	0	−66.95	105.12
−22	−7	−6	9.51	100.37
17	−6	2	81.22	83.99
11	−2	1	725.41	81.61
−19	−1	−6	97.33	92.57
−18	1	−7	42.00	102.08
−17	2	−7	58.90	90.59
8	3	−1	427.22	58.11

−16	3	−7	−24.56	81.22
−13	4	−5	−11.62	66.95
36	−39	6	0.00	87.82
33	−36	6	0.00	80.69
0	−31	4	−37.37	72.37
−5	−29	3	−90.59	85.58
0	−28	4	185.68	79.50
−12	−28	1	23.11	93.63
−15	−24	0	−2.64	88.74
27	−23	5	74.75	80.69
17	−21	5	116.87	56.52
−15	−18	0	−5.68	103.14
24	−8	2	72.50	77.52
−20	−7	−4	−7.53	94.69
31	−4	−1	111.86	111.06
21	−4	1	186.47	83.46
29	−3	−1	95.88	98.91
27	−2	−1	83.07	85.05
22	−2	0	65.50	78.97
−18	−1	−5	31.96	80.16
−19	−1	−9	−30.64	93.24
15	0	0	120.57	78.05
−7	0	−1	882.17	90.46
−11	0	−2	557.96	77.65
21	2	−2	37.90	78.31
19	4	−3	96.93	84.39
14	4	−2	236.26	82.27
19	5	−4	−22.98	76.46
−3	7	−4	1107.20	96.54
6	9	−6	174.98	48.86
1	−36	4	−5.28	79.37
−7	−36	2	88.35	99.31
−4	−34	3	−2.11	90.46
25	−32	6	25.49	68.41
22	−32	6	−59.82	61.41
33	−26	5	147.91	90.20
−5	−26	3	6.87	91.65
−9	−26	2	4.89	93.63
−9	−23	2	−44.11	104.59
36	−22	4	−66.03	94.16
20	−21	5	115.03	64.71
−15	−21	0	−35.39	101.42
38	−19	3	11.62	93.50
−19	−17	−2	−79.37	103.93
−17	−16	−1	33.15	112.12
36	−14	2	79.37	100.10
−12	−9	0	30.77	84.39
−21	−7	−5	−138.27	121.10
−18	−6	−3	41.20	90.73
33	−5	−1	97.20	120.97
−14	−3	−2	638.78	105.12
−5	−2	0	5.81	50.98
−18	0	−6	23.24	82.67
−14	4	−6	364.49	82.14

6	5	−2	590.84	66.95
8	−39	5	0.00	66.95
35	−37	6	−5.15	85.44
35	−27	5	−4.75	90.86
25	−22	5	−20.47	75.80
−12	−19	1	104.33	85.18
−7	−15	2	60.35	62.86
−14	−14	0	104.59	80.03
−16	−12	−1	22.05	82.80
−21	−11	−4	0.00	105.52
−8	−9	1	88.35	69.60
35	−6	−1	100.10	99.84
−5	−6	1	104.33	40.54
−13	−2	−2	386.01	89.93
25	−1	−1	32.09	80.16
−12	−1	−2	212.75	70.39
−17	0	−5	253.95	87.16
−18	0	−9	−3.04	92.71
−17	1	−6	−5.28	75.01
−14	3	−5	149.23	71.84
−15	3	−6	−4.23	72.11
40	−31	5	−26.41	96.93
−12	−25	1	−34.60	92.31
−17	−22	−1	−0.40	96.80
−17	−19	−1	3.70	104.06
38	−15	2	−38.56	94.16
22	−15	4	131.40	72.77
−11	−15	1	138.00	79.37
14	−14	4	522.30	77.26
−20	−14	−3	5.41	110.14
20	−10	3	−8.85	72.11
12	−9	3	479.12	73.43
22	−7	2	121.10	75.67
−21	−6	−6	59.03	111.99
−21	−5	−7	−37.11	98.12
5	−2	1	1247.05	114.36
−16	1	−5	−1.85	75.94
−17	1	−9	−30.77	92.71
16	2	−1	147.91	85.71
−16	2	−6	36.58	75.27
19	6	−5	8.72	84.65
7	−34	5	−7.53	56.39
30	−33	6	19.15	76.99
38	−23	4	10.43	99.57
−12	−22	1	−110.27	106.18
17	−14	4	284.33	66.16
−17	−9	−2	91.65	85.71
−7	−8	1	43.05	53.75
−6	−7	1	33.15	46.75
−13	−6	−1	104.72	85.97
12	−5	2	278.91	61.41
9	−5	2	1302.12	117.67
−20	−3	−8	−39.22	93.24
20	−1	0	144.74	83.07

23	0	−1	247.09	85.71
7	1	0	749.45	79.24
12	7	−4	303.08	68.14
37	−38	6	−74.48	92.18
2	−38	4	10.70	83.59
−3	−36	3	57.84	93.10
32	−34	6	0.00	80.43
28	−32	6	−9.11	73.56
7	−31	5	31.43	51.24
−11	−30	1	164.28	102.74
37	−28	5	−12.28	96.27
23	−21	5	−42.79	73.16
−3	−18	3	143.02	60.48
29	−13	3	7.13	85.58
−18	−13	−2	58.37	98.25
−19	−10	−3	0.00	96.40
15	−9	3	136.15	71.84
−11	−8	0	204.96	83.07
−19	−6	−4	13.60	86.37
−17	−5	−3	103.93	84.52
19	−3	1	264.25	92.18
14	−2	1	725.81	93.37
19	3	−2	66.16	72.90
11	3	−1	1532.70	143.42
12	6	−3	55.47	75.67
−11	6	−8	−1.72	75.14
−9	7	−7	146.32	63.52
34	−35	6	−65.37	84.52
23	−31	6	0.00	66.43
1	−24	4	17.56	57.97
−8	−19	2	10.83	67.75
27	−12	3	3.96	78.18
32	−1	−3	18.22	102.74
32	0	−4	80.82	94.03
30	0	−3	64.97	105.91
30	1	−4	−7.00	94.95
28	1	−3	0.00	116.08
10	1	0	540.00	68.94
28	2	−4	−103.54	100.10
17	5	−3	153.06	73.82
9	5	−2	40.54	55.33
17	6	−4	−0.66	72.63
14	8	−6	55.47	71.45
26	−31	6	−15.45	71.84
−4	−31	3	102.74	88.61
−14	−26	0	−42.66	95.08
14	−21	5	219.35	50.45
31	−18	4	3.57	106.44
29	−17	4	−10.17	97.86
−14	−17	0	−48.33	87.42
35	−16	3	−17.04	97.33
−20	−10	−4	0.00	115.03
−21	−10	−5	8.32	111.59
32	−8	1	5.68	106.04

30	−7	1	108.42	96.93
20	−6	2	98.25	83.20
−12	−5	−1	487.70	92.44
−20	−4	−7	7.26	102.88
34	−2	−3	−19.94	90.46
−19	−2	−8	1.06	102.35
34	−1	−4	76.20	94.95
−8	−1	−1	32.35	46.88
−8	7	−6	2.25	53.48
3	−40	4	−14.53	87.42
−2	−38	3	17.83	95.48
36	−36	6	79.63	88.74
1	−33	4	81.61	76.07
−8	−28	2	13.73	92.05
32	−24	5	0.00	89.41
30	−23	5	64.84	94.69
−4	−22	3	40.28	66.29
33	−19	4	50.18	100.50
27	−16	4	11.36	82.01
−16	−15	−1	−5.15	92.44
20	−14	4	138.93	70.12
−6	−14	2	91.52	71.71
−13	−13	0	104.46	79.10
25	−11	3	93.76	80.29
−15	−11	−1	88.22	85.71
34	−9	1	0.00	114.36
1	−7	2	116.87	19.02
−10	−7	0	961.40	110.27
28	−6	1	15.19	81.22
15	−5	2	371.62	75.01
−20	−5	−6	100.63	118.19
−16	−4	−3	141.44	74.75
26	2	−3	−24.70	97.46
26	3	−4	52.03	107.10
12	8	−5	68.80	64.84
−7	−33	2	30.90	99.31
8	−27	5	−19.02	33.81
34	−25	5	−0.13	90.07
−14	−23	0	−49.13	100.10
28	−22	5	−64.58	89.93
35	−20	4	88.48	95.61
−14	−20	0	54.67	106.04
−11	−18	1	−22.45	74.22
37	−17	3	40.41	100.63
−18	−16	−2	0.00	114.50
36	−10	1	52.03	98.39
18	−9	3	50.58	84.12
9	−9	3	195.32	47.41
−16	−8	−2	−45.03	82.54
33	−6	0	89.93	118.99
31	−5	0	−16.38	106.31
26	−5	1	117.27	87.95
−18	−5	−4	150.55	85.44
29	−4	0	45.43	92.71

−11	−4	−1	318.27	74.61
−6	−3	0	442.14	59.43
32	−2	−2	−33.94	110.40
−9	−2	−1	679.32	78.97
30	−1	−2	−63.26	109.74
−18	−1	−8	−18.49	107.10
28	0	−2	−2.38	100.23
32	1	−5	0.00	98.78
30	2	−5	−22.98	92.44
−2	2	−1	979.36	82.27
28	3	−5	−19.55	95.61
2	3	−1	1246.79	110.01
−1	−40	3	23.90	100.10
31	−32	6	124.40	82.54
−10	−32	1	5.15	103.67
−4	−28	3	78.31	90.46
−11	−27	1	−65.24	92.05
−8	−25	2	−67.88	100.37
−8	−22	2	0.00	83.59
21	−20	5	−30.64	64.05
18	−20	5	51.37	58.37
−18	−19	−2	0.00	100.89
−19	−13	−3	83.59	119.52
33	−11	2	86.90	123.48
31	−10	2	−34.60	98.52
29	−9	2	−22.71	83.46
−18	−9	−3	71.18	81.61
35	−7	0	−84.52	106.57
34	−3	−2	45.03	95.61
27	−3	0	12.28	83.86
−10	−3	−1	68.54	60.48
18	0	0	564.16	102.22
24	3	−3	−111.33	86.24
24	4	−4	0.00	104.59
−12	5	−8	−57.45	84.78
17	7	−5	40.67	73.95
9	9	−6	93.76	56.13
38	−37	6	−11.62	93.50
33	−33	6	107.23	85.84
19	−32	6	−14.26	50.32
29	−31	6	82.80	78.31
24	−30	6	0.00	69.99
36	−26	5	137.61	97.73
−4	−25	3	60.48	94.56
37	−21	4	11.62	95.22
26	−21	5	0.13	72.11
−16	−21	−1	48.33	100.50
39	−18	3	−69.86	102.08
25	−15	4	4.89	77.26
11	−14	4	255.54	60.62
35	−12	2	−28.66	118.72
−17	−12	−2	15.19	83.59
23	−10	3	39.75	77.78
27	−8	2	−21.53	89.14

-9	-6	0	234.41	64.45
24	-4	1	-22.45	83.46
-15	-3	-3	53.75	78.71
-19	-3	-7	57.31	122.68
-17	0	-8	-0.13	108.69
26	1	-2	-6.87	92.97
13	1	0	126.65	62.33
26	4	-5	42.52	94.82
17	4	-2	257.25	74.75
-8	5	-4	136.82	56.79
19	7	-6	0.00	97.86
-6	-35	2	31.83	101.69
35	-34	6	46.22	89.54
20	-31	6	17.83	56.65
27	-30	6	130.74	77.39
1	-30	4	74.35	70.39
-13	-28	0	22.32	106.71
1	-27	4	-16.51	84.92
-11	-24	1	86.37	108.55
-11	-21	1	-17.04	97.73
-2	-17	3	12.02	52.56
-20	-13	-4	-16.64	108.55
37	-8	0	127.17	100.89
-8	-5	0	757.77	83.07
-7	-4	0	875.04	87.03
-19	-4	-6	237.45	114.36
25	-2	0	-94.82	88.48
17	-2	1	303.87	84.39
14	3	-1	900.92	107.10
-3	-33	3	74.35	91.12
38	-27	5	4.75	98.78
39	-22	4	21.79	93.63
-7	-18	2	-4.49	68.28
-19	-16	-3	38.30	103.93
37	-13	2	3.70	98.91
-19	-9	-4	4.75	104.59
-20	-9	-5	150.42	120.18
25	-7	2	1.06	87.29
-17	-4	-4	131.00	94.16
4	1	0	886.66	82.93
-16	1	-8	112.38	108.55
24	2	-2	-116.08	79.24
-5	3	-2	330.02	45.69
22	4	-3	150.42	79.37
-13	4	-8	29.05	91.52
24	5	-5	-22.58	104.20
12	5	-2	124.01	73.69
-8	6	-5	115.03	66.03
-10	6	-7	11.49	63.92
40	-38	6	-56.52	102.61
8	-36	5	-22.19	63.26
37	-35	6	91.52	91.78
2	-35	4	72.37	77.92
21	-30	6	0.00	61.67

−7	−30	2	29.32	91.65
24	−20	5	−42.00	74.22
23	−14	4	58.64	75.54
−5	−13	2	185.41	70.65
−12	−12	0	0.13	74.61
−14	−10	−1	151.87	75.01
−15	−7	−2	12.28	79.50
18	−5	2	387.86	99.84
−14	−2	−3	−35.00	84.39
−18	−2	−7	−26.41	113.57
−10	2	−3	51.50	59.43
−15	2	−8	−73.56	106.44
−14	3	−8	138.00	103.01
22	5	−4	30.37	86.63
−5	−37	2	−62.60	104.33
18	−33	6	−17.83	44.11
−10	−29	1	1.45	95.88
−15	−14	−1	32.62	84.92
−20	−8	−6	14.66	107.76
−20	−7	−7	−24.04	100.10
22	−3	1	101.42	80.56
−18	−3	−9	−33.94	101.82
−18	−3	−6	28.26	95.48
23	−1	0	192.94	85.71
15	6	−3	145.27	67.48
15	7	−4	82.27	69.46
32	−31	6	−49.52	84.25
25	−29	6	0.92	74.88
−13	−25	0	1.32	97.20
15	−20	5	71.31	51.24
−17	−15	−2	11.09	104.46
32	−13	3	−62.86	117.93
18	−13	4	39.49	62.60
15	−13	4	129.29	65.11
−18	−12	−3	96.80	97.73
21	−9	3	25.22	72.50
−17	−8	−3	41.07	80.95
23	−6	2	−2.64	74.88
−13	−1	−3	172.08	82.54
−12	0	−3	798.18	102.74
−11	1	−3	623.59	84.65
7	8	−4	212.49	61.67
−4	−39	2	0.00	107.37
39	−36	6	97.99	102.74
34	−32	6	39.09	88.61
30	−30	6	82.93	81.75
33	−23	5	68.14	91.65
2	−23	4	16.77	50.05
31	−22	5	166.79	96.93
−3	−21	3	0.00	62.33
−13	−19	0	57.71	96.93
−17	−18	−2	−46.49	108.03
32	−17	4	73.29	108.29
−10	−17	1	4.89	78.05

30	−12	3	−6.87	98.25
−20	−12	−5	8.45	107.23
6	−5	2	1323.12	115.42
−19	−5	−8	9.24	97.59
−16	−3	−4	164.68	83.07
−17	−1	−7	0.00	108.16
22	3	−2	22.32	78.18
22	6	−5	−19.02	105.12
−9	6	−6	−22.98	58.90
4	8	−4	167.19	47.15
−2	−35	3	0.00	91.52
36	−33	6	−141.83	93.24
28	−29	6	0.00	70.26
22	−29	6	70.39	66.69
−7	−27	2	0.00	92.18
35	−24	5	30.77	91.12
−15	−23	−1	33.02	97.46
−13	−22	0	−107.89	110.27
29	−21	5	−25.09	99.18
34	−18	4	34.07	98.65
−15	−17	−1	−4.62	95.22
30	−16	4	97.73	104.06
−19	−12	−4	99.97	126.12
28	−11	3	137.61	84.78
−16	−11	−2	0.00	81.22
−19	−8	−5	−83.46	119.78
−14	−6	−2	15.45	83.20
−17	−3	−5	109.21	82.67
−17	−2	−9	−45.69	93.90
16	1	0	61.80	79.37
20	5	−3	30.64	75.94
−11	5	−7	69.86	70.26
3	−37	4	3.43	81.88
−3	−30	3	29.71	83.46
−10	−26	1	108.82	98.25
37	−25	5	38.43	96.54
−7	−21	2	181.58	75.41
−15	−20	−1	17.04	111.06
36	−19	4	−47.41	94.95
19	−19	5	256.99	68.80
38	−16	3	106.57	97.59
−1	−16	3	47.41	53.75
28	−15	4	−21.00	92.44
−13	−9	−1	43.71	77.26
16	−8	3	219.22	77.92
13	−8	3	290.27	67.48
−18	−8	−4	−64.45	88.61
−17	−2	−6	196.51	90.20
12	−1	1	85.05	54.15
9	−1	1	57.45	45.43
−16	0	−7	16.77	93.37
−9	4	−4	458.38	72.37
20	6	−4	67.48	79.10
7	7	−3	1543.53	141.97

17	8	−6	130.74	102.35
15	8	−5	18.88	68.80
−5	8	−7	53.09	48.33
38	−34	6	12.02	101.82
−9	−31	1	20.34	101.69
−7	−24	2	96.40	101.03
38	−20	4	−67.22	102.48
27	−20	5	0.00	90.59
22	−19	5	63.26	68.94
−18	−15	−3	−10.96	114.76
21	−13	4	−43.98	71.84
−11	−11	0	122.68	80.43
26	−10	3	15.05	82.80
35	−8	1	−13.34	109.48
33	−7	1	20.34	111.86
−19	−7	−6	1.72	122.16
31	−6	1	3.04	100.50
−19	−6	−7	−2.38	106.71
20	−2	1	85.71	80.82
−15	−2	−4	56.52	74.75
−16	−1	−9	−16.24	95.22
15	5	−2	65.50	77.78
10	8	−4	163.76	60.22
12	9	−6	49.65	64.18
9	−38	5	−4.89	67.88
−1	−37	3	14.13	95.35
2	−32	4	136.15	78.44
39	−26	5	−44.90	101.29
−10	−23	1	42.26	106.18
−10	−20	1	19.28	75.94
−18	−18	−3	−12.28	106.04
−19	−15	−4	−12.28	107.63
26	−14	4	13.73	82.54
37	−9	1	10.17	99.31
32	−9	2	23.90	107.23
−16	−7	−3	59.82	83.86
29	−5	1	83.46	90.33
21	−5	2	54.54	74.88
13	−4	2	83.86	59.96
−18	−4	−8	−45.03	104.33
−16	−2	−5	124.53	82.80
33	0	−3	143.02	107.10
33	1	−4	16.38	98.91
31	1	−3	−9.90	96.01
−15	1	−7	−42.39	80.69
31	2	−4	−58.50	95.74
−12	4	−7	134.31	69.86
−9	5	−5	109.21	67.75
−4	8	−6	42.66	38.96
8	−33	5	−24.56	57.58
26	−28	6	0.00	76.07
23	−28	6	−11.23	69.86
−12	−27	0	−22.19	96.93
−3	−24	3	7.40	73.16

−6	−17	2	6.87	64.58
−12	−15	0	0.92	77.78
−14	−13	−1	105.38	83.86
−8	−12	1	284.59	81.48
36	−11	2	−29.19	102.48
34	−10	2	42.66	106.84
30	−8	2	5.81	98.12
19	−8	3	−25.49	75.67
34	−5	0	0.00	108.16
32	−4	0	23.90	112.38
27	−4	1	41.20	84.92
30	−3	0	28.92	106.04
15	−1	1	322.89	76.86
−16	−1	−6	66.16	75.80
−15	0	−9	−44.24	98.39
29	2	−3	108.55	111.99
−14	2	−7	63.92	77.78
29	3	−4	108.95	99.71
−13	3	−7	89.67	71.18
10	7	−3	309.29	76.86
−7	7	−8	213.28	66.69
7	9	−5	62.07	49.92
4	−39	4	−15.45	86.63
40	−35	6	−28.26	108.55
35	−31	6	−32.62	90.07
33	−30	6	43.05	86.50
31	−29	6	−127.70	85.31
25	−19	5	2.64	76.86
−16	−14	−2	27.47	90.86
−17	−11	−3	12.55	89.14
24	−9	3	108.16	78.58
28	−7	2	143.02	84.52
−18	−7	−5	−25.75	95.48
36	−6	0	−5.15	100.10
−13	−5	−2	123.87	88.74
16	−4	2	282.35	80.16
10	−4	2	918.62	92.71
35	−2	−2	13.60	100.63
33	−1	−2	−136.95	101.42
31	0	−2	74.22	106.84
27	3	−3	−12.15	107.10
−10	3	−4	1309.52	132.19
9	4	−1	256.33	57.71
−10	5	−6	34.47	61.67
20	7	−5	−155.96	103.54
0	−39	3	67.88	96.67
−5	−34	2	0.00	102.35
8	−30	5	−10.56	47.94
16	−19	5	3.17	54.28
12	−13	4	158.21	60.88
−3	−11	2	567.47	67.22
−19	−11	−5	93.37	115.29
−15	−10	−2	44.50	83.33
−17	−7	−4	34.07	87.29

28	−2	0	77.65	83.73
−15	−1	−5	85.31	77.26
−15	0	−6	39.09	77.65
29	1	−2	114.76	112.25
−14	1	−9	−0.13	97.46
27	4	−4	35.00	104.06
−4	5	−3	369.90	43.18
4	7	−3	1268.97	113.70
5	−41	4	118.59	92.84
1	−41	3	13.07	103.80
−8	−33	1	−55.86	104.20
37	−32	6	−14.13	97.59
−2	−32	3	−21.13	88.22
29	−28	6	−24.17	79.76
2	−26	4	19.55	72.37
−14	−25	−1	−58.64	97.20
−12	−24	0	−45.83	106.04
−16	−20	−2	34.20	105.52
−16	−17	−2	−5.94	114.63
−9	−16	1	95.88	72.37
−18	−11	−4	26.02	105.12
−12	−8	−1	132.99	81.61
−18	−6	−6	−41.60	115.55
−18	−5	−7	−70.39	124.14
25	−3	1	28.13	87.56
−17	−3	−8	54.94	103.80
19	1	0	81.61	80.03
−12	1	−4	206.28	82.14
−11	2	−4	886.79	112.25
31	3	−5	−64.18	101.82
12	4	−1	538.54	83.20
18	6	−3	149.23	74.09
10	−40	5	−14.92	71.58
2	−29	4	−56.92	78.05
−6	−29	2	0.00	89.54
−9	−28	1	−31.03	96.93
36	−23	5	0.00	97.33
34	−22	5	−14.53	90.33
32	−21	5	−85.44	93.37
−12	−18	0	180.53	85.18
−14	−16	−1	51.37	87.29
10	−8	3	255.14	51.24
26	−6	2	−14.66	79.63
−15	−6	−3	71.71	79.37
−12	−4	−2	337.02	81.88
26	−1	0	−81.61	80.03
−14	0	−5	388.79	82.54
−14	1	−6	0.00	76.46
27	2	−2	−84.78	113.84
11	2	0	96.14	61.01
−6	2	−2	131.27	41.47
29	4	−5	1.32	97.20
25	4	−3	32.62	105.78
−10	4	−5	81.88	71.58

−11	4	−6	369.24	73.56
18	7	−4	−33.68	73.95
10	9	−5	122.95	59.16
4	9	−5	29.32	38.03
−1	9	−7	59.82	28.79
39	−33	6	0.00	102.88
−11	−29	0	−29.85	106.57
−14	−22	−1	0.00	103.40
−12	−21	0	61.80	108.55
30	−20	5	40.15	100.10
−2	−20	3	42.66	60.75
20	−18	5	0.00	61.80
35	−17	4	−27.47	105.91
33	−16	4	68.67	103.14
0	−15	3	86.50	52.16
−17	−14	−3	103.14	122.29
35	−13	3	−68.54	104.86
33	−12	3	77.26	114.50
19	−12	4	196.77	68.67
31	−11	3	111.06	114.23
−10	−10	0	253.56	86.24
35	−3	−1	0.00	94.16
33	−2	−1	70.92	98.65
18	−1	1	178.55	89.67
−13	2	−6	71.45	73.29
−12	3	−6	158.21	72.90
25	5	−4	186.73	110.54
13	7	−3	9.11	64.58
13	8	−4	86.63	65.24
−4	−36	2	0.00	102.61
38	−24	5	−29.98	99.57
−6	−20	2	90.33	71.58
37	−18	4	69.07	101.69
23	−18	5	87.03	72.50
31	−15	4	101.55	112.52
−18	−14	−4	119.38	120.97
16	−12	4	370.56	75.41
−7	−11	1	478.33	83.59
−2	−10	2	278.38	45.69
22	−8	3	−37.51	79.76
−17	−5	−9	27.20	104.20
19	−4	2	186.73	86.90
−16	−2	−8	−15.85	109.48
31	−1	−1	−67.35	116.08
−13	1	−5	101.55	71.05
8	2	0	55.73	43.84
−11	3	−5	124.53	72.77
6	4	−1	1819.54	157.15
27	5	−5	48.73	96.14
3	−34	4	13.87	75.94
34	−29	6	61.80	88.22
19	−29	6	52.96	55.99
−6	−26	2	−4.62	105.25
28	−19	5	0.00	103.14

−17	−17	−3	84.52	112.91
29	−14	4	0.13	100.10
−13	−12	−1	−7.79	80.43
29	−10	3	25.75	90.73
−16	−10	−3	11.09	78.84
0	−8	2	569.84	54.01
−18	−7	−8	−2.64	108.16
−16	−6	−4	159.40	89.93
24	−5	2	29.98	85.18
−17	−4	−7	117.53	115.29
23	−2	1	−27.34	79.50
29	0	−1	124.93	121.63
24	0	0	207.60	89.27
14	2	0	112.12	70.26
−12	2	−5	185.55	69.07
25	3	−2	0.00	87.42
18	5	−2	148.83	75.14
10	6	−2	237.31	66.16
−3	−38	2	−0.13	105.38
36	−30	6	−43.71	96.54
32	−28	6	−59.03	85.84
20	−28	6	0.00	60.22
−9	−25	1	29.32	105.38
3	−22	4	67.88	48.07
−9	−19	1	16.64	75.54
−5	−16	2	158.61	65.24
−11	−14	0	35.66	77.65
−15	−13	−2	−27.34	85.44
22	−12	4	207.47	76.73
−18	−10	−5	32.49	118.46
−1	−9	2	70.52	31.83
−14	−9	−2	−0.92	78.44
−11	−7	−1	394.60	90.73
−17	−5	−6	−3.96	97.59
−11	−3	−2	510.02	83.59
6	−1	1	980.16	95.61
23	5	−3	102.48	85.58
−8	6	−8	−15.05	66.43
18	8	−5	9.38	84.78
15	9	−6	0.00	85.84
−1	−34	3	54.94	96.01
38	−31	6	49.79	101.03
−5	−31	2	58.77	93.76
−8	−30	1	3.30	97.33
−2	−29	3	−56.52	89.80
30	−27	6	0.00	84.39
−6	−23	2	26.15	81.22
−9	−22	1	111.33	90.59
27	−13	4	38.03	83.46
−17	−10	−4	50.18	92.97
27	−9	3	9.51	77.12
−14	−5	−3	176.43	85.05
−15	−1	−8	−33.81	114.76
27	1	−1	99.84	104.46

25	6	−5	−3.43	97.99
23	6	−4	99.44	106.84
7	6	−2	112.38	48.60
−2	−40	2	58.77	112.12
18	−30	6	−13.21	48.60
−11	−26	0	130.21	98.91
26	−18	5	43.45	91.12
17	−18	5	72.63	60.88
−18	−9	−6	50.05	119.91
−18	−8	−7	−37.51	113.04
36	−7	1	38.69	106.31
17	−7	3	353.79	91.12
14	−7	3	1371.45	136.15
34	−6	1	48.60	105.91
32	−5	1	16.51	120.97
−16	−4	−9	18.75	95.35
−10	−2	−2	323.55	63.79
−7	1	−2	174.72	46.22
40	−32	6	−50.32	114.63
−2	−23	3	97.33	64.97
−15	−16	−2	−51.90	94.69
37	−10	2	3.70	99.84
35	−9	2	174.45	106.84
−9	−9	0	42.66	74.48
33	−8	2	3.96	114.89
31	−7	2	124.01	114.23
30	−4	1	34.07	111.59
−16	−3	−7	134.44	112.65
17	2	0	131.40	87.03
23	4	−2	21.39	76.60
13	6	−2	217.24	78.71
13	9	−5	71.84	64.97
4	−36	4	−15.45	71.31
9	−35	5	−5.55	61.67
−2	−26	3	−35.92	93.76
−13	−24	−1	−46.09	99.57
35	−21	5	9.11	91.78
33	−20	5	−101.69	98.12
−15	−19	−2	21.79	125.59
−13	−15	−1	16.90	80.16
−16	−13	−3	0.00	85.97
−18	−13	−5	129.29	109.61
13	−12	4	203.37	65.90
25	−8	3	−15.98	80.56
20	−7	3	219.75	78.18
−17	−6	−8	41.73	97.86
−15	−5	−4	17.43	72.63
35	−4	0	65.24	106.31
22	−4	2	25.09	74.22
33	−3	0	15.32	103.54
−9	−1	−2	55.07	52.96
−8	0	−2	574.47	69.46
−14	0	−8	161.38	109.74
22	1	0	4.49	80.16

25	2	−1	137.87	86.50
16	7	−3	184.62	75.14
−6	7	−7	43.18	54.01
0	−36	3	69.20	94.56
−11	−23	0	35.00	112.91
37	−22	5	0.00	97.73
31	−19	5	135.10	99.71
−17	−13	−4	−24.43	121.50
25	−12	4	3.70	76.46
−6	−10	1	35.92	57.71
29	−6	2	98.91	101.55
−10	−6	−1	101.16	68.14
−16	−4	−6	44.90	86.76
28	−3	1	52.16	88.74
14	−3	2	598.10	84.65
31	−2	0	−4.09	125.72
13	0	1	405.56	73.16
34	1	−3	0.00	105.91
32	2	−3	−4.23	95.35
−9	5	−8	122.95	73.16
21	6	−3	96.67	79.24
23	7	−5	47.01	104.33
16	8	−4	−10.70	70.78
−7	−32	1	−6.21	101.95
3	−31	4	0.00	72.24
37	−29	6	−41.34	103.54
35	−28	6	−45.69	99.05
33	−27	6	−68.14	90.99
39	−23	5	43.32	101.16
−13	−21	−1	31.30	123.08
−11	−20	0	−14.92	83.99
21	−17	5	154.38	67.75
1	−14	3	200.07	47.81
−12	−11	−1	311.14	85.84
−15	−9	−3	−31.56	83.99
−17	−9	−5	46.88	105.12
−13	−8	−2	−7.26	79.63
−13	−4	−3	154.38	87.56
−15	−3	−9	0.00	96.80
34	0	−2	4.09	101.95
−13	1	−8	97.33	105.52
32	3	−4	1.98	100.50
30	3	−3	0.00	102.74
21	7	−4	93.63	92.31
−5	−28	2	0.00	94.56
−8	−27	1	21.66	99.05
−16	−19	−3	9.51	102.48
29	−18	5	0.00	100.89
−13	−18	−1	70.12	87.95
24	−17	5	44.90	73.43
36	−16	4	−95.35	99.44
−16	−16	−3	−66.56	114.89
−17	−16	−4	0.66	111.20
34	−15	4	0.00	97.46

32	−14	4	−35.92	116.35
34	−11	3	31.17	103.67
−16	−9	−4	24.17	82.14
27	−5	2	−14.13	79.76
7	−4	2	1929.94	165.34
17	−3	2	711.15	102.74
−15	−2	−7	−7.53	92.18
29	−1	0	76.20	106.31
16	0	1	683.02	104.20
32	1	−2	−65.37	99.97
−12	2	−8	27.73	92.71
30	4	−4	0.00	104.20
−10	4	−8	26.54	79.37
1	−38	3	178.81	99.97
−1	−31	3	125.06	84.25
39	−30	6	0.00	104.59
−10	−28	0	33.41	95.22
9	−26	5	33.41	27.34
26	−25	6	37.90	79.90
−1	−19	3	56.39	54.28
38	−17	4	6.34	103.67
−10	−13	0	50.84	75.27
36	−12	3	−17.96	100.50
−14	−12	−2	161.38	83.73
20	−11	4	64.45	69.20
17	−11	4	99.97	66.16
32	−10	3	123.87	119.12
−17	−8	−6	0.00	116.74
−17	−7	−7	165.60	114.50
26	−2	1	19.55	80.16
10	0	1	211.69	51.50
30	2	−2	26.81	103.01
23	3	−1	237.05	87.82
−11	3	−8	20.34	84.92
28	4	−3	−115.16	107.89
21	5	−2	149.49	79.76
16	6	−2	3.70	70.78
−4	7	−5	774.14	79.63
5	−38	4	19.02	84.78
−5	−19	2	35.00	68.14
−8	−18	1	8.58	71.05
18	−17	5	89.54	62.99
30	−13	4	−152.53	112.38
30	−9	3	59.69	101.42
−8	−8	0	391.83	73.69
23	−7	3	67.62	79.50
11	−7	3	1563.60	142.36
−16	−5	−8	−1.19	104.20
−14	−4	−4	252.24	83.46
−15	−4	−5	0.00	78.18
11	−3	2	613.03	74.88
−15	−3	−6	3.57	76.60
27	0	0	97.86	94.95
28	5	−4	148.96	103.01

2	−40	3	−73.43	100.10
−3	−35	2	60.35	101.69
−6	−34	1	133.78	109.61
−12	−26	−1	22.58	95.48
29	−25	6	22.71	83.33
23	−25	6	112.38	75.01
3	−25	4	−44.11	58.24
−8	−24	1	−60.09	104.33
27	−17	5	−74.48	101.95
−4	−15	2	142.76	68.67
23	−11	4	−14.26	75.94
−9	−5	−1	63.39	56.79
20	−3	2	181.19	79.76
−12	−3	−3	138.40	90.86
−14	−2	−9	21.13	98.12
34	−1	−1	−9.38	108.55
−14	−1	−7	59.43	82.80
28	3	−2	208.26	115.95
16	9	−5	62.60	76.99
10	10	−6	123.87	59.82
3	−42	3	−65.77	107.37
6	−40	4	16.90	89.80
3	−28	4	−50.32	85.05
−5	−25	2	24.30	96.27
−8	−21	1	−2.38	73.69
−14	−15	−2	82.54	83.33
28	−12	4	29.71	91.91
−17	−12	−5	71.05	119.78
−5	−9	1	−29.58	45.83
25	−4	2	−18.88	84.25
32	0	−1	63.39	105.78
19	0	1	310.48	91.39
26	5	−3	51.90	111.72
−4	6	−4	107.10	37.37
−5	7	−6	62.33	45.03
21	8	−5	54.28	104.46
7	10	−6	263.33	53.22
7	−42	4	−57.97	92.57
36	−27	6	38.83	99.84
−10	−25	0	−5.68	107.37
−5	−22	2	10.70	70.78
−14	−21	−2	−28.53	110.80
36	−20	5	−0.13	95.48
−15	−12	−3	76.07	79.90
28	−8	3	5.94	83.59
−14	−8	−3	6.60	70.78
−16	−8	−5	31.96	89.01
24	−1	1	3.70	82.54
30	1	−1	75.94	121.50
25	1	0	−6.74	78.31
5	2	0	20.21	51.90
−5	4	−3	393.28	48.73
−7	6	−7	−25.09	61.01
10	−37	5	−3.30	65.50

−2	−37	2	−123.34	110.14
−5	−36	1	46.88	108.29
17	−31	6	0.00	38.69
−9	−30	0	62.07	103.14
−7	−29	1	79.10	97.33
38	−28	6	4.89	102.08
38	−21	5	−126.51	105.38
12	−20	5	66.43	38.43
34	−19	5	68.14	94.29
−14	−18	−2	−58.77	109.87
−17	−15	−5	0.00	103.54
−12	−14	−1	170.49	80.95
−16	−12	−4	13.60	95.61
−11	−10	−1	297.27	90.20
−17	−10	−7	11.75	111.33
6	−9	3	174.72	31.83
−12	−7	−2	175.24	86.90
18	−6	3	−13.47	80.69
−16	−6	−7	−53.09	118.59
35	−5	1	61.54	105.91
−14	−3	−5	187.13	85.44
−13	−1	−9	−44.11	100.37
−3	1	−1	386.28	42.26
12	3	0	364.49	69.33
26	4	−2	7.00	106.31
10	5	−1	229.39	60.88
26	6	−4	114.63	108.16
0	−33	3	23.37	87.56
−4	−30	2	0.00	90.99
40	−29	6	−50.98	107.37
−1	−28	3	80.69	97.73
24	−24	6	36.45	75.80
−12	−23	−1	60.88	104.99
−15	−21	−3	33.94	114.76
13	−19	5	88.88	45.43
32	−18	5	−59.69	96.80
22	−16	5	41.60	67.88
−10	−16	0	−3.04	82.14
14	−11	4	323.42	77.39
36	−8	2	−40.28	98.91
−15	−8	−4	−53.88	88.08
34	−7	2	22.32	107.89
−16	−7	−6	73.29	109.48
15	−6	3	122.95	72.11
33	−4	1	134.04	111.46
−8	−4	−1	175.24	53.35
−15	−4	−8	−1.19	114.36
−13	−3	−4	145.66	78.18
−11	−2	−3	5.68	76.20
−14	−2	−6	98.78	78.84
−13	0	−7	6.07	76.07
28	2	−1	0.00	110.54
15	3	0	88.88	78.05
21	4	−1	166.13	79.76

4	6	−2	1178.78	105.12
11	8	−3	10.04	67.35
11	9	−4	62.33	62.86
8	9	−4	−12.41	54.15
−1	−39	2	42.39	105.78
4	−33	4	43.32	73.82
9	−32	5	−7.13	54.41
−11	−28	−1	−4.36	96.80
−10	−22	0	−61.54	99.97
−15	−18	−3	−25.36	117.53
−16	−18	−4	0.00	106.18
30	−17	5	32.88	103.80
−12	−17	−1	53.88	78.05
25	−16	5	15.05	84.65
−15	−15	−3	16.64	94.69
−16	−15	−4	15.58	118.72
26	−11	4	−29.19	77.65
−13	−11	−2	34.47	76.46
26	−7	3	178.41	89.67
−7	−7	0	1784.41	160.59
32	−6	2	−13.73	114.10
21	−6	3	125.19	71.71
31	−3	1	12.81	117.67
−12	0	−9	159.93	104.06
24	6	−3	101.03	105.38
19	6	−2	47.28	76.99
8	8	−3	267.29	68.54
13	10	−6	−14.66	73.82
0	−41	2	61.14	110.54
30	−24	6	−51.11	83.86
−1	−22	3	48.86	62.99
−10	−19	0	−60.88	78.18
19	−16	5	254.35	68.01
37	−15	4	51.24	96.01
35	−14	4	−18.49	99.97
−9	−12	0	236.52	84.25
−16	−8	−8	−27.20	105.38
−15	−6	−9	110.80	99.18
30	−5	2	0.00	111.72
23	−3	2	99.71	81.75
34	−2	0	148.04	101.03
−12	1	−7	−12.41	70.65
14	8	−3	129.55	68.94
−8	−32	0	−11.36	111.59
−1	−25	3	−47.81	74.35
4	−21	4	215.66	46.49
14	−18	5	249.07	53.88
33	−13	4	46.88	107.76
2	−13	3	31.56	33.41
21	−10	4	177.36	76.60
18	−10	4	210.64	68.14
29	−2	1	48.99	106.18
32	−1	0	71.71	110.14
−10	−1	−3	457.33	78.71

22	0	1	5.81	81.35
−11	1	−9	−17.83	107.23
23	2	0	11.49	80.03
9	3	0	580.28	72.11
24	5	−2	19.81	88.48
−8	5	−7	67.35	61.80
24	7	−4	−21.13	108.16
14	9	−4	81.48	68.54
11	−39	5	73.69	71.45
−6	−31	1	26.54	95.48
−7	−26	1	16.90	108.03
28	−16	5	35.79	106.71
31	−12	4	−89.01	120.57
37	−11	3	0.00	99.31
−16	−11	−5	86.37	110.93
35	−10	3	117.40	103.14
33	−9	3	150.68	110.27
−4	−8	1	247.09	48.07
−15	−5	−7	61.54	113.97
−7	−3	−1	1058.20	102.61
−14	−3	−8	102.48	115.82
15	−2	2	510.42	89.80
−13	−2	−5	35.79	70.12
−13	−1	−6	−56.65	73.82
−11	2	−7	33.68	71.18
26	3	−1	223.05	94.29
18	3	0	131.40	85.31
1	−35	3	166.79	97.99
−3	−32	2	−29.85	93.10
39	−27	6	0.00	104.72
−4	−27	2	45.43	104.06
−9	−27	0	35.13	104.06
37	−26	6	−28.26	104.72
20	−25	6	−14.39	64.05
28	−23	6	0.00	81.48
25	−23	6	−25.62	76.07
−7	−17	1	169.96	71.31
−14	−11	−3	41.20	81.88
−16	−9	−7	21.92	108.69
−13	−7	−3	166.79	76.33
−11	−6	−2	264.78	86.63
28	−4	2	80.29	90.46
18	−2	2	174.06	91.39
30	0	0	128.63	129.02
7	0	1	1159.23	107.76
14	1	1	290.27	75.01
33	3	−3	−25.49	101.03
−10	3	−7	162.96	71.31
−9	4	−7	−14.13	64.58
11	7	−2	303.87	78.05
19	9	−5	−14.13	100.23
−11	−25	−1	86.10	108.95
33	−24	6	−3.70	90.59
11	−21	5	126.38	29.45

−4	−18	2	−28.00	61.41
15	−17	5	−4.62	52.82
8	−14	4	4.75	36.98
−3	−14	2	569.71	81.75
−13	−14	−2	28.39	78.71
−16	−14	−5	136.42	116.08
−15	−11	−4	19.15	86.90
31	−8	3	−23.24	115.03
−14	−7	−4	242.46	81.22
24	−6	3	43.05	81.09
−15	−6	−6	6.74	83.99
27	−1	1	57.71	83.73
−9	0	−3	607.48	77.39
17	1	1	245.37	85.84
33	2	−2	0.00	99.18
31	4	−3	54.81	97.06
7	5	−1	819.57	83.99
14	7	−2	460.89	82.54
12	−41	5	0.00	74.75
5	−35	4	26.94	76.33
21	−24	6	17.30	66.16
−7	−23	1	102.48	85.84
−13	−20	−2	86.10	117.27
37	−19	5	36.58	103.80
35	−18	5	−8.45	95.22
0	−18	3	120.57	51.64
23	−15	5	37.37	71.84
−11	−13	−1	77.92	77.92
29	−11	4	47.94	112.65
−10	−9	−1	432.50	87.82
12	−6	3	349.30	66.16
−6	−6	0	358.02	55.07
−14	−5	−9	88.35	96.40
−6	−2	−1	1085.41	102.74
−12	0	−6	39.35	78.71
31	3	−2	−14.92	103.93
3	4	−1	2658.65	221.60
19	5	−1	23.90	73.29
−6	6	−6	252.76	56.52
22	7	−3	32.09	89.14
11	10	−5	144.21	62.60
2	−37	3	89.14	94.82
−5	−33	1	23.64	99.71
0	−30	3	112.78	90.46
31	−23	6	−28.53	90.46
−7	−20	1	41.73	71.71
33	−17	5	26.81	100.50
−13	−17	−2	36.18	87.56
9	−13	4	267.82	50.05
−6	−13	1	38.96	69.07
29	−7	3	66.16	111.46
−15	−7	−8	127.31	102.88
26	−3	2	278.25	85.84
8	−3	2	710.22	73.43

21	−2	2	45.43	76.60
12	−2	2	346.92	65.24
−13	−2	−8	129.95	110.40
−12	−1	−5	−18.49	69.46
−4	0	−1	2976.39	248.41
28	1	0	0.00	103.14
−8	1	−3	213.67	52.96
24	4	−1	3.70	81.22
31	5	−4	34.34	100.63
29	5	−3	0.00	110.93
24	8	−5	−24.04	94.95
17	8	−3	31.96	74.75
−4	−24	2	3.83	76.07
−9	−24	0	−22.19	107.89
−4	−21	2	−46.22	69.07
−14	−20	−3	−35.79	118.72
26	−15	5	−20.34	92.57
20	−15	5	212.09	70.12
−14	−14	−3	166.53	87.29
−15	−14	−4	−63.26	104.72
−16	−13	−6	50.71	117.40
15	−10	4	19.68	70.52
−12	−10	−2	10.17	71.18
7	−8	3	149.36	36.58
−14	−4	−7	122.42	96.40
−5	−1	−1	892.34	92.05
33	1	−1	109.87	99.18
11	1	1	153.19	54.28
21	3	0	149.76	80.95
22	6	−2	77.39	79.10
8	7	−2	140.12	57.84
22	8	−4	−61.14	109.21
17	9	−4	70.39	76.99
8	10	−5	202.85	55.86
−8	−29	0	−3.96	93.90
22	−23	6	0.00	72.11
26	−22	6	0.00	79.10
−11	−22	−1	101.42	113.18
−14	−17	−3	−1.32	110.80
−15	−17	−4	−44.77	117.93
31	−16	5	−52.56	97.59
−8	−11	0	228.86	83.59
35	−6	2	−67.35	104.59
19	−5	3	201.13	82.01
25	0	1	−84.52	78.84
−11	1	−6	77.52	69.86
29	4	−2	61.67	106.31
29	6	−4	93.63	102.35
−5	6	−5	361.19	62.33
17	7	−2	139.98	81.35
−4	7	−8	188.85	57.71
14	10	−5	92.44	69.86
3	−39	3	10.96	95.74
−6	−28	1	−9.38	99.44

38	−25	6	26.28	106.84
−12	−25	−2	−84.12	98.39
29	−22	6	−31.03	85.44
−11	−19	−1	53.22	80.56
16	−16	5	324.74	63.79
−11	−16	−1	8.72	80.29
36	−13	4	0.00	102.74
−15	−10	−5	−67.48	88.08
−3	−7	1	1150.91	103.14
27	−6	3	−30.11	82.54
33	−5	2	7.66	100.76
16	−5	3	188.19	77.92
−10	−5	−2	535.38	91.78
−13	−4	−9	63.13	99.57
34	−3	1	24.04	103.54
−11	0	−5	465.78	84.12
31	2	−1	0.00	106.71
26	2	0	14.92	88.22
−5	5	−4	542.51	61.94
5	9	−4	255.80	55.20
5	−43	3	89.54	106.57
4	−41	3	0.00	98.78
−10	−27	−1	0.00	96.67
34	−23	6	152.40	100.10
−9	−21	0	−9.90	81.88
29	−15	5	34.07	105.38
34	−12	4	−11.49	97.99
−15	−8	−7	157.94	128.50
22	−5	3	44.77	80.43
−14	−5	−6	75.27	85.71
32	−2	1	0.00	114.36
−12	−1	−8	62.86	103.54
−10	0	−4	1885.44	174.19
−10	2	−6	51.37	64.71
−7	5	−6	112.78	56.26
27	6	−3	112.12	102.61
5	8	−3	343.75	54.81
−1	−36	2	−17.70	97.20
−3	−29	2	−4.89	92.97
10	−12	4	82.67	49.26
32	−11	4	11.49	104.59
−13	−10	−3	−15.58	82.67
−14	−10	−4	73.29	82.80
−15	−10	−8	0.00	103.67
36	−9	3	0.00	98.65
−15	−9	−6	166.13	110.93
−13	−6	−4	193.07	74.61
−5	−5	0	−10.17	40.54
31	−4	2	−86.37	115.95
24	−2	2	−0.40	85.31
29	3	−1	120.04	114.89
27	5	−2	95.61	118.19
−3	−37	1	−11.75	105.91
31	−36	7	62.46	79.76

35	−35	7	78.05	93.63
10	−34	5	−0.79	56.65
−7	−31	0	18.36	95.61
4	−27	4	72.37	76.20
0	−27	3	6.21	89.01
4	−24	4	26.94	48.86
32	−22	6	41.07	89.93
23	−22	6	33.68	74.35
−12	−22	−2	52.96	114.23
−12	−13	−2	90.07	80.56
−15	−13	−5	17.56	125.06
34	−8	3	−106.04	102.74
−14	−8	−9	−2.77	96.54
−14	−6	−8	−64.18	108.82
−13	−3	−7	−0.13	87.56
30	−1	1	20.87	112.65
−9	3	−6	11.09	62.73
16	4	0	91.78	81.88
13	4	0	345.34	81.48
−8	4	−6	41.34	58.11
22	5	−1	60.75	81.75
27	7	−4	−124.14	101.16
20	8	−3	−31.69	78.71
22	9	−5	94.42	99.71
4	10	−6	220.54	37.24
7	−39	4	76.99	85.84
0	−38	2	0.00	103.67
32	−35	7	4.75	85.31
17	−28	6	42.79	42.52
38	−18	5	−58.64	100.23
36	−17	5	−26.81	97.73
−15	−16	−5	10.56	114.23
−9	−8	−1	52.16	68.01
33	0	0	0.00	106.44
−11	0	−8	41.34	92.44
23	1	1	−57.05	81.48
−9	1	−4	712.87	93.37
−10	1	−5	14.53	75.01
17	10	−5	−9.51	91.12
9	−43	4	−55.86	92.57
8	−41	4	−96.40	89.93
1	−40	2	52.56	104.06
33	−34	7	−30.11	85.58
−5	−30	1	−76.07	93.37
−8	−26	0	−22.85	105.12
−6	−25	1	−18.49	99.57
−13	−22	−3	4.62	111.33
27	−21	6	−15.85	82.41
0	−21	3	97.86	57.84
34	−16	5	62.99	93.63
17	−15	5	71.84	60.62
27	−14	5	28.92	101.16
21	−14	5	153.32	71.05
−15	−11	−7	22.19	113.70

32	−7	3	0.00	111.59
25	−5	3	−7.00	84.92
29	−3	2	7.79	103.93
−12	−3	−9	6.87	101.69
19	−1	2	−39.22	75.41
16	−1	2	223.18	90.20
24	3	0	−7.79	77.12
−6	4	−4	96.01	49.65
−6	5	−5	299.78	64.97
25	7	−3	−11.23	109.48
20	7	−2	2.77	77.12
20	9	−4	−135.10	99.44
2	−42	2	−64.18	107.50
36	−34	7	87.29	93.63
−9	−29	−1	−3.83	96.01
0	−24	3	37.90	61.41
−10	−24	−1	127.84	113.97
30	−21	6	−9.11	89.67
−12	−19	−2	−65.90	94.16
−14	−19	−4	46.09	108.55
−12	−16	−2	84.39	78.84
32	−15	5	−28.00	98.25
−13	−13	−3	0.00	81.61
−14	−13	−4	−52.03	91.65
13	−5	3	299.25	69.60
−9	−4	−2	20.47	56.39
−13	−4	−6	12.28	81.88
28	0	1	81.09	102.22
31	1	0	66.56	107.10
−8	2	−4	284.46	67.35
−9	2	−5	117.93	73.43
6	3	0	574.99	65.77
27	4	−1	50.18	108.03
19	4	0	185.28	68.28
25	6	−2	−65.90	106.04
17	6	−1	50.71	72.90
34	−33	7	76.99	90.73
−6	−33	0	29.45	97.99
5	−32	4	12.81	71.58
−3	−26	2	25.09	90.07
37	−23	6	5.02	101.16
24	−21	6	−8.19	76.86
−13	−19	−3	26.28	115.16
−3	−17	2	96.80	63.92
−14	−16	−4	31.43	113.44
−15	−15	−6	22.98	106.31
−5	−12	1	488.23	79.63
11	−11	4	369.24	62.99
−14	−9	−5	−44.77	90.73
8	−7	3	256.33	45.83
−14	−7	−7	126.12	114.50
30	−6	3	35.79	123.87
−2	−6	1	1346.89	117.27
−11	−5	−3	151.61	87.69

−10	1	−8	−15.98	80.69
15	2	1	425.24	91.78
−7	3	−4	1348.08	126.51
−5	6	−8	141.70	59.82
25	8	−4	101.16	99.05
12	9	−3	13.87	60.75
30	−34	7	29.71	80.69
−2	−31	2	−48.60	90.73
35	−22	6	0.00	100.23
−6	−22	1	101.95	74.09
−6	−19	1	14.00	72.37
−13	−16	−3	−39.88	90.59
−8	−14	0	248.41	83.20
−14	−8	−6	−90.86	94.42
−12	−5	−4	−4.09	74.48
−13	−5	−8	39.88	118.19
−4	−4	0	508.57	61.14
27	−2	2	36.71	87.95
−12	−2	−7	−10.70	80.29
22	−1	2	−95.48	86.10
18	2	1	186.34	83.46
−8	3	−5	132.46	71.18
10	4	0	1179.97	115.69
−7	4	−5	829.87	96.14
18	−39	6	29.19	25.49
29	−35	7	57.58	76.33
39	−34	7	−90.59	103.27
2	−34	3	−21.13	83.07
37	−33	7	−13.47	94.95
31	−33	7	1.58	80.43
−8	−23	0	−72.24	91.39
33	−21	6	22.58	94.69
−10	−15	−1	0.00	75.41
30	−14	5	101.42	104.72
−7	−10	0	962.72	108.42
−14	−9	−8	90.33	100.89
−13	−7	−9	−34.20	95.22
20	−4	3	189.64	77.65
9	−2	2	806.10	83.33
−11	−2	−9	63.92	100.63
13	−1	2	203.51	67.88
8	1	1	136.15	48.86
32	4	−2	0.00	95.61
32	5	−3	14.53	97.20
−2	8	−7	132.46	32.35
15	9	−3	−18.49	69.60
12	10	−4	288.16	69.99
35	−32	7	−4.89	88.22
32	−32	7	116.74	85.58
−4	−32	1	−43.18	94.16
28	−20	6	25.49	82.54
18	−14	5	233.88	68.54
35	−11	4	−0.40	102.88
−12	−9	−3	−0.66	77.78

−13	−9	−4	141.83	88.35
28	−5	3	95.61	96.14
17	−4	3	203.37	80.03
26	1	1	−13.07	88.61
25	5	−1	14.92	93.76
−6	5	−8	16.24	64.05
20	6	−1	36.32	73.43
−7	−28	0	0.00	102.61
−3	−23	2	116.87	70.52
25	−20	6	38.03	80.16
−3	−20	2	22.19	65.63
−10	−18	−1	165.60	79.10
25	−13	5	21.00	80.43
22	−13	5	28.66	70.52
−14	−12	−5	57.05	104.20
34	−4	2	−32.35	100.89
23	−4	3	114.89	78.84
21	2	1	−5.15	79.76
12	2	1	655.15	82.67
−8	3	−8	0.00	73.03
22	4	0	83.59	84.52
−7	4	−8	113.44	68.54
32	6	−4	−106.97	112.78
30	6	−3	55.33	97.59
23	8	−3	0.00	109.08
15	8	−2	74.88	69.46
12	8	−2	17.43	73.29
9	9	−3	12.28	65.90
20	10	−5	−17.17	108.03
15	10	−4	58.37	70.65
5	10	−5	−24.56	40.15
11	11	−6	−51.50	61.54
28	−36	7	101.16	73.69
11	−36	5	0.00	52.16
−1	−33	2	−35.52	89.14
1	−29	3	−11.36	94.29
−5	−27	1	0.13	103.67
−9	−26	−1	4.62	103.14
19	−23	6	40.41	59.56
20	−22	6	97.46	63.92
31	−20	6	−151.74	93.76
−8	−20	0	−68.54	78.05
−14	−18	−5	−70.26	111.86
1	−17	3	1.72	49.52
−8	−17	0	74.61	75.41
37	−16	5	25.49	98.12
−14	−15	−5	−55.07	121.89
3	−12	3	496.95	45.43
−11	−12	−2	144.34	77.92
12	−10	4	63.39	62.07
−14	−10	−7	16.24	119.25
−8	−7	−1	0.00	55.86
−12	−4	−5	116.48	74.61
32	−3	2	−71.18	106.57

−12	−3	−6	87.29	75.14
33	−1	1	0.00	106.84
−10	−1	−9	−16.77	103.54
32	3	−1	75.14	101.69
30	5	−2	24.04	97.99
23	7	−2	−21.66	89.67
18	9	−3	49.26	75.94
9	10	−4	100.10	54.41
3	−36	3	−10.56	92.05
−3	−34	1	27.86	97.20
40	−33	7	−43.18	105.78
38	−32	7	62.46	96.67
33	−31	7	64.31	81.75
−13	−21	−4	3.70	103.80
35	−15	5	53.75	99.18
28	−13	5	35.39	107.23
35	−7	3	−24.96	98.12
−13	−6	−7	−49.13	99.84
−12	−4	−8	0.00	108.82
−8	−3	−2	479.51	70.12
25	−1	2	−17.17	78.44
−11	−1	−7	0.00	71.18
8	6	−1	228.73	51.37
30	7	−4	−14.26	100.89
5	7	−2	308.36	49.79
23	9	−4	59.69	105.25
6	−34	4	−27.07	73.03
29	−32	7	37.11	78.71
36	−31	7	85.18	91.78
18	−24	6	2.91	54.81
38	−22	6	0.00	101.29
36	−21	6	25.88	100.89
21	−21	6	50.98	67.35
−11	−21	−2	−41.34	115.29
33	−14	5	159.40	97.73
−13	−12	−4	24.56	80.69
−9	−11	−1	188.98	86.37
−10	−8	−2	96.14	81.88
−13	−8	−5	102.74	81.48
33	−6	3	−64.18	103.67
9	−6	3	185.28	47.15
−12	−6	−9	94.82	96.01
−10	−4	−3	594.01	94.69
−3	−3	0	−19.28	43.71
31	0	1	169.83	110.54
28	7	−3	−18.36	101.42
18	8	−2	16.38	75.54
28	−33	7	74.48	75.01
30	−31	7	−14.66	77.92
−2	−28	2	0.00	102.88
−12	−21	−3	40.67	112.91
5	−20	4	75.01	22.19
−11	−15	−2	0.00	76.99
−14	−14	−6	9.90	120.04

−14	−13	−7	−40.01	103.40
−12	−12	−3	71.45	85.58
−13	−8	−8	−61.67	103.54
−13	−7	−6	−11.49	81.35
26	−4	3	83.46	82.80
14	−4	3	625.84	91.25
−11	−4	−4	34.86	85.31
30	−2	2	−37.77	117.53
24	2	1	10.56	80.56
30	4	−1	46.35	102.74
28	6	−2	150.02	102.08
1	7	−3	190.04	21.79
18	10	−4	−21.53	83.20
4	−38	3	−15.58	90.46
34	−30	7	−140.38	89.54
31	−30	7	54.01	83.33
−6	−30	0	−73.69	96.80
−10	−26	−2	0.00	96.93
34	−20	6	−116.48	98.91
29	−19	6	37.51	90.99
26	−19	6	117.80	82.01
−11	−18	−2	0.00	80.56
−13	−18	−4	91.52	117.40
−13	−15	−4	−55.47	96.01
19	−13	5	0.00	63.79
−1	−5	1	996.00	89.14
20	0	2	68.54	80.82
17	0	2	457.46	95.88
−9	0	−9	51.50	106.31
17	5	0	248.41	82.01
14	5	0	229.92	82.54
23	6	−1	44.90	87.42
9	8	−2	−35.66	65.77
7	−44	3	26.81	105.78
6	−42	3	83.99	100.10
5	−40	3	68.54	91.65
39	−31	7	−45.56	102.61
10	−31	5	40.01	51.11
5	−29	4	47.15	82.67
−8	−28	−1	88.08	97.20
−7	−25	0	13.47	110.14
−5	−24	1	100.50	81.35
−9	−23	−1	−78.71	106.57
22	−20	6	31.30	70.12
−12	−18	−3	3.30	102.74
−12	−15	−3	93.90	82.27
31	−13	5	133.78	100.63
−11	−7	−10	52.30	99.05
31	−5	3	−52.03	114.23
−11	−2	−6	95.35	71.18
−10	0	−7	161.64	76.07
29	1	1	105.25	114.50
28	8	−4	−113.18	98.91
12	11	−5	−0.66	60.62

8	11	−6	15.32	49.39
12	−38	5	−6.34	64.71
−1	−38	1	44.64	104.20
1	−37	2	33.02	97.46
27	−34	7	−11.49	67.62
37	−30	7	13.21	91.78
−4	−29	1	78.31	96.67
32	−19	6	49.92	87.69
26	−12	5	143.29	92.44
23	−12	5	−35.52	70.65
−4	−11	1	252.90	58.50
13	−9	4	163.62	67.88
−11	−8	−3	26.81	86.10
−12	−8	−4	63.92	76.46
24	−7	4	63.92	77.92
21	−7	4	541.98	90.20
21	−3	3	377.17	85.05
−11	−3	−5	211.03	75.41
28	−1	2	32.88	102.35
28	5	−1	24.43	107.76
15	7	−1	−17.83	74.61
21	9	−3	84.65	92.44
15	11	−5	93.50	79.90
7	−36	4	76.99	75.67
35	−29	7	13.07	86.24
32	−29	7	32.09	81.48
1	−26	3	19.55	71.45
−7	−13	0	183.83	82.14
−13	−11	−8	−53.22	97.06
−13	−11	−5	55.99	86.37
−6	−9	0	134.57	55.73
−13	−9	−7	−67.62	116.61
−12	−5	−7	−24.83	86.76
18	−3	3	41.20	77.65
−11	−3	−8	100.10	106.44
−7	−2	−2	570.37	71.05
10	−1	2	71.05	49.65
23	0	2	60.88	81.48
14	0	2	1373.17	141.70
−8	1	−9	−52.69	105.65
19	3	1	47.15	65.77
16	3	1	279.84	91.52
20	5	0	14.13	76.86
4	5	−1	828.81	79.37
26	7	−2	−24.30	111.59
18	7	−1	98.91	81.35
26	8	−3	−86.50	101.55
21	8	−2	55.99	79.10
4	−43	2	28.92	102.22
2	−39	2	−132.06	98.52
−5	−32	0	33.41	94.56
2	−31	3	0.00	76.07
−9	−28	−2	0.00	91.78
−5	−21	1	22.19	73.82

−9	−20	−1	34.60	82.67
23	−19	6	71.31	76.07
36	−14	5	99.71	102.08
−9	−14	−1	50.32	72.90
29	−12	5	18.36	104.99
−12	−9	−9	0.00	95.35
−7	−6	−1	24.83	50.84
−11	−5	−9	−38.17	101.69
29	−4	3	60.75	109.21
24	−3	3	40.81	80.56
−9	−3	−3	412.43	74.61
−2	−2	0	422.20	41.07
3	−41	2	95.35	100.89
13	−40	5	−23.51	70.12
17	−25	6	−9.38	58.37
−2	−25	2	1.06	76.46
−10	−23	−2	53.22	118.59
−11	−23	−3	−18.49	102.88
−7	−22	0	112.78	78.58
37	−20	6	49.92	96.01
27	−18	6	20.60	85.18
−5	−18	1	10.43	66.95
−9	−17	−1	87.03	76.46
−13	−17	−5	21.39	120.84
−2	−16	2	72.11	67.35
−13	−14	−5	−10.83	106.71
−13	−10	−6	67.09	97.46
27	−7	4	−17.96	92.84
10	−5	3	575.39	70.12
−9	1	−7	94.29	73.29
27	2	1	−19.55	102.48
9	2	1	755.92	83.46
7	4	0	1001.29	96.14
11	5	0	609.99	86.37
26	9	−4	−3.04	93.24
21	10	−4	−5.28	107.76
18	11	−5	69.73	104.06
40	−30	7	11.75	107.63
28	−30	7	1.19	72.24
−1	−30	2	0.00	90.33
−7	−30	−1	36.85	92.31
38	−29	7	29.45	101.16
33	−28	7	20.07	84.25
1	−20	3	92.71	54.15
35	−19	6	43.05	97.73
30	−18	6	−23.77	89.67
34	−13	5	−42.52	95.61
20	−12	5	75.14	71.05
−10	−11	−2	240.22	87.42
32	−8	4	0.00	96.80
−12	−7	−8	114.10	114.50
−12	−6	−6	−58.77	85.97
−10	−3	−4	295.68	86.24
22	3	1	90.73	82.67

13	3	1	245.90	75.54
26	6	−1	38.30	109.08
−3	7	−7	237.58	52.69
9	11	−5	95.74	56.39
−4	−34	0	0.00	100.23
27	−31	7	−48.47	69.86
−3	−31	1	21.92	90.73
36	−28	7	54.15	89.27
−6	−27	0	97.59	111.06
5	−26	4	125.33	59.03
1	−23	3	0.40	61.80
−12	−20	−4	−73.16	114.76
−7	−16	0	317.21	76.86
−13	−16	−6	0.00	104.99
−13	−13	−6	26.81	106.44
−13	−12	−7	−36.45	108.95
−12	−11	−4	103.40	83.73
−9	−7	−2	263.59	75.01
15	−3	3	147.25	77.26
33	−2	2	16.64	100.50
3	−1	1	452.97	46.22
−10	−1	−6	0.00	70.52
26	0	2	229.52	90.99
21	7	−1	125.46	79.37
10	−42	4	−54.41	85.44
9	−40	4	10.96	80.56
30	−28	7	14.26	78.18
−8	−25	−1	−39.88	117.01
−10	−20	−2	17.70	87.95
33	−18	6	94.95	92.31
24	−18	6	−0.26	75.94
14	−15	5	25.62	50.32
32	−12	5	59.82	94.69
−11	−11	−3	40.94	73.16
−8	−10	−1	−15.19	70.52
34	−5	3	35.13	95.48
27	−3	3	−11.49	100.76
−10	−2	−8	−5.15	91.65
−10	−2	−5	49.92	72.37
−8	2	−7	202.71	70.52
31	6	−2	82.80	94.42
31	7	−3	−6.74	96.54
6	9	−3	158.61	57.31
16	10	−3	127.31	74.09
13	10	−3	123.34	67.62
−3	−36	0	0.00	102.35
26	−35	7	−12.94	63.52
−6	−32	−1	85.71	99.18
34	−27	7	32.75	85.71
−4	−26	1	100.50	95.08
5	−23	4	115.42	47.15
−2	−22	2	0.00	64.58
−11	−20	−3	10.17	122.02
−12	−17	−4	94.56	106.84

13	−16	5	43.45	44.50
15	−14	5	49.92	54.67
−12	−14	−4	193.07	84.92
27	−11	5	−13.47	102.35
24	−11	5	74.75	79.37
30	−7	4	19.41	106.18
−10	−4	−9	115.55	100.76
31	−1	2	66.56	112.52
−6	−1	−2	88.74	46.22
32	1	1	5.81	113.04
24	8	−2	85.97	108.69
24	9	−3	−39.09	110.40
16	9	−2	105.38	74.22
26	−32	7	−0.92	67.09
39	−28	7	18.62	105.65
31	−27	7	123.74	82.80
−2	−19	2	43.32	59.56
−10	−17	−2	−5.68	88.88
−10	−14	−2	124.53	76.07
−11	−7	−4	118.46	72.24
22	−6	4	138.53	78.44
32	−4	3	69.99	109.08
−11	−4	−7	10.96	78.97
−1	−1	0	−1.58	6.21
21	1	2	0.00	81.75
18	1	2	169.04	83.20
25	3	1	67.62	85.31
31	5	−1	46.22	94.69
13	9	−2	−3.43	63.26
6	10	−4	65.90	50.58
−2	−33	1	−73.69	92.05
0	−32	2	−79.10	91.39
37	−27	7	160.98	100.89
−10	−25	−3	0.00	97.20
28	−17	6	172.60	95.35
−11	−17	−3	70.78	89.67
−11	−14	−3	95.35	90.46
21	−11	5	102.74	72.63
−12	−10	−8	−75.94	105.38
−12	−10	−5	17.30	79.90
−11	−8	−9	−34.86	99.18
−12	−8	−7	71.31	108.69
−10	−7	−3	220.67	88.88
25	−6	4	−44.11	76.20
22	−2	3	66.16	73.69
−8	−2	−3	48.73	56.79
−7	3	−7	246.43	71.84
−4	6	−7	−1.72	52.03
29	7	−2	57.31	104.06
24	10	−4	−69.20	98.12
19	10	−3	1.58	79.10
16	11	−4	41.73	75.67
13	11	−4	66.03	64.97
−5	−29	0	103.40	97.06

−12	−19	−5	−105.38	104.20
36	−18	6	41.86	95.22
31	−17	6	19.55	92.31
25	−17	6	−0.13	78.58
16	−13	5	14.92	56.52
30	−11	5	63.79	103.67
19	−6	4	165.08	74.35
−11	−6	−8	−92.97	116.61
11	−4	3	1335.53	128.50
19	−2	3	148.83	77.26
29	0	2	64.97	112.38
−9	0	−6	10.17	65.63
30	2	1	−73.29	117.93
24	7	−1	−17.04	96.54
29	8	−3	2.38	100.50
19	9	−2	70.39	74.22
10	10	−3	−20.87	60.62
2	−28	3	94.03	90.59
−7	−27	−1	60.75	102.48
35	−26	7	−55.86	92.31
32	−26	7	−16.38	82.01
−6	−24	0	17.17	87.42
−11	−22	−4	5.81	98.65
35	−12	5	14.39	93.37
−12	−9	−6	−34.20	81.48
28	−6	4	−137.34	105.52
−11	−5	−6	106.44	77.65
30	−3	3	−122.42	124.80
25	−2	3	−20.21	79.90
−9	−1	−8	−46.88	84.78
11	0	2	421.14	67.48
24	1	2	40.81	78.84
15	1	2	400.41	89.67
−6	4	−7	−18.49	61.80
−5	5	−7	115.03	59.30
15	6	0	108.42	81.35
4	−35	3	122.42	84.65
−1	−35	1	57.05	96.01
27	−28	7	15.72	71.71
10	−28	5	1.58	46.22
28	−27	7	19.15	74.75
−1	−27	2	−30.24	89.93
19	−20	6	33.68	61.67
34	−17	6	54.94	95.08
12	−17	5	151.08	41.60
−12	−16	−5	64.58	122.02
−4	−14	1	651.46	92.97
−12	−13	−5	−15.05	94.29
−6	−12	0	340.72	77.78
−12	−11	−7	0.00	117.80
−5	−8	0	657.00	75.27
−6	−5	−1	120.57	50.58
−9	−3	−9	164.42	105.52
20	4	1	0.13	85.97

17	4	1	374.00	86.24
29	6	−1	22.58	105.12
19	11	−4	143.15	103.27
1	−34	2	0.00	88.22
−4	−23	1	79.50	73.69
20	−19	6	78.05	65.90
−8	−13	−1	52.30	84.25
17	−12	5	73.69	62.60
25	−10	5	−15.85	89.01
33	−7	4	75.14	98.12
−9	−1	−5	0.00	74.48
31	4	0	127.04	97.20
16	8	−1	3.17	68.54
10	11	−4	153.32	61.67
9	−45	3	0.00	102.48
5	−37	3	−25.36	87.82
11	−33	5	9.38	56.39
−4	−31	0	−62.60	92.57
26	−29	7	39.22	69.73
−3	−28	1	−76.73	107.89
−8	−27	−2	73.29	102.48
38	−26	7	61.54	101.55
29	−26	7	42.39	77.52
33	−25	7	145.40	85.18
−9	−22	−2	−83.59	109.21
−10	−22	−3	42.66	115.55
18	−21	6	15.19	55.20
−8	−19	−1	64.45	77.92
21	−18	6	−21.79	67.75
29	−16	6	121.23	92.57
2	−16	3	5.94	49.92
−12	−15	−6	−19.94	112.78
−12	−14	−7	0.00	101.69
33	−11	5	2.51	88.35
28	−10	5	14.92	108.42
−3	−10	1	113.70	45.16
−9	−10	−2	207.73	91.78
−11	−10	−4	167.32	83.86
−8	−6	−2	204.30	68.80
0	−4	1	3420.91	279.57
−10	−3	−7	56.26	70.39
16	−2	3	36.45	79.50
4	0	1	2566.74	216.32
−5	0	−2	75.94	41.20
28	3	1	0.00	112.52
10	3	1	94.82	54.67
27	8	−2	132.46	101.69
19	8	−1	0.00	78.31
6	8	−2	155.30	50.32
27	9	−3	59.96	94.56
22	9	−2	2.77	93.63
10	9	−2	210.51	75.80
22	10	−3	16.11	108.55
2	−41	1	10.83	94.42

1	−39	1	0.13	101.42
25	−33	7	57.45	59.43
36	−25	7	−27.86	95.22
−11	−19	−4	46.62	116.74
32	−16	6	−1.19	91.39
26	−16	6	37.64	99.31
−8	−16	−1	10.04	74.75
22	−10	5	47.41	71.97
−10	−7	−9	0.00	95.74
28	−2	3	−40.94	118.59
−8	0	−8	−41.34	74.61
27	1	2	−9.51	103.40
−8	1	−6	31.43	60.62
23	4	1	87.95	80.43
14	4	1	903.03	114.76
12	6	0	403.31	87.03
13	8	−1	44.64	77.52
8	−43	3	7.53	92.71
7	−41	3	0.00	91.25
6	−39	3	−52.16	87.42
2	−36	2	0.00	81.75
−6	−29	−1	5.15	96.01
30	−25	7	−19.55	80.16
−6	−21	0	60.88	78.44
−4	−20	1	5.15	68.41
22	−17	6	96.40	70.65
−4	−17	1	194.39	62.86
−10	−10	−3	0.00	79.90
−11	−9	−8	0.53	106.04
−11	−7	−7	43.58	86.50
31	−6	4	1.19	104.72
23	−5	4	54.81	77.26
12	−3	3	376.51	71.45
8	5	0	743.64	81.61
5	6	−1	143.29	40.67
27	7	−1	13.21	114.36
6	−44	2	91.78	101.69
3	−38	2	3.57	93.76
7	−33	4	24.83	71.58
25	−30	7	32.09	64.84
−9	−19	−2	4.89	76.07
37	−17	6	−32.75	96.14
−11	−16	−4	−39.49	94.95
−6	−15	0	42.39	77.39
−11	−13	−4	37.11	82.93
18	−11	5	229.26	70.92
31	−10	5	106.31	97.46
−7	−9	−1	483.34	76.86
−9	−8	−10	−70.65	95.61
16	−6	4	76.07	74.35
26	−5	4	27.34	86.50
20	−5	4	39.35	71.84
−10	−5	−8	87.69	107.37
33	−3	3	0.00	107.89

−8	−2	−9	38.43	105.78
−7	−1	−3	167.59	54.28
32	0	2	−0.66	102.74
19	2	2	23.90	73.03
29	5	0	−42.13	103.01
22	8	−1	142.49	83.20
22	11	−4	55.60	99.18
16	12	−5	−45.16	97.59
13	12	−5	59.16	68.28
5	−42	2	0.00	96.27
4	−40	2	−41.60	99.57
−3	−33	0	46.35	89.41
−7	−29	−2	0.00	92.05
−5	−26	0	−21.39	104.20
34	−24	7	−44.77	92.31
31	−24	7	74.48	82.01
−7	−24	−1	−50.58	102.22
−10	−19	−3	32.35	89.14
−9	−13	−2	3.43	75.80
−11	−9	−5	10.43	86.90
−11	−8	−6	63.26	77.92
−9	−6	−3	117.14	76.86
−10	−4	−6	247.88	82.93
23	−1	3	69.33	82.93
20	−1	3	0.00	78.71
22	2	2	−19.55	80.56
3	−30	3	13.21	91.25
−2	−30	1	−45.43	96.40
0	−29	2	25.22	100.63
2	−25	3	0.00	59.82
−1	−24	2	−13.87	67.35
17	−22	6	0.92	51.11
35	−16	6	71.84	94.42
−9	−16	−2	−31.83	86.63
−10	−16	−3	189.90	82.80
30	−15	6	0.13	89.14
−1	−15	2	309.15	66.82
−10	−13	−3	0.00	81.75
−11	−12	−8	−6.87	104.20
29	−5	4	93.24	109.61
−10	−5	−5	−76.20	74.61
4	−4	2	2584.04	213.81
5	−3	2	285.12	43.05
−8	0	−5	41.60	70.39
12	1	2	223.71	69.86
−7	2	−6	99.05	59.30
26	4	1	16.77	97.46
−1	8	−6	38.69	29.05
25	9	−2	8.19	107.63
25	−36	7	17.43	53.88
−2	−35	0	82.14	95.08
−5	−31	−1	30.11	91.12
37	−24	7	−93.37	97.06
−8	−24	−2	0.00	119.12

−9	−24	−3	−79.24	106.31
−11	−18	−5	−30.37	121.36
23	−16	6	21.39	74.35
27	−15	6	47.67	99.05
−10	−10	−9	−108.42	95.35
−11	−10	−7	28.26	112.91
26	−9	5	24.83	95.61
31	−2	3	137.08	111.86
−9	−2	−7	166.66	73.56
26	−1	3	24.17	91.91
30	1	2	105.38	115.82
−7	1	−8	14.00	69.99
25	2	2	−15.72	86.24
16	2	2	156.23	91.91
31	3	1	0.00	96.01
25	10	−3	−75.41	99.31
17	11	−3	178.94	75.67
6	−28	4	49.79	77.39
−3	−25	1	25.49	78.05
33	−15	6	14.53	87.03
−11	−12	−5	2.51	84.78
34	−10	5	−26.68	93.24
19	−10	5	206.54	72.77
29	−9	5	0.00	104.72
23	−9	5	74.75	72.77
34	−6	4	25.75	99.31
17	−1	3	−9.38	73.82
5	1	1	969.59	97.06
−4	1	−2	166.53	40.28
27	6	0	−94.42	113.18
30	8	−2	−9.64	95.48
17	10	−2	−29.58	74.48
14	11	−3	154.64	73.03
6	11	−5	58.50	45.83
−1	−37	0	−3.30	94.42
8	−35	4	−54.94	73.43
26	−26	7	128.89	72.90
27	−25	7	7.79	72.50
35	−23	7	−31.03	95.22
32	−23	7	12.15	80.43
−10	−21	−4	35.66	120.31
−11	−17	−6	−63.13	108.55
−11	−16	−7	29.98	108.69
−11	−15	−5	−5.94	93.24
−11	−13	−7	0.00	115.03
0	−11	2	445.84	52.43
−9	−6	−9	11.75	102.74
6	−2	2	86.50	38.17
−7	−1	−9	56.92	103.93
21	5	1	17.56	79.90
18	5	1	24.83	81.75
16	7	0	445.31	84.39
25	8	−1	65.77	115.82
10	8	−1	29.19	66.69

30	9	−3	0.00	93.90
20	10	−2	31.03	87.69
14	10	−2	153.85	69.86
20	11	−3	56.26	99.31
12	−35	5	−43.32	59.16
−4	−33	−1	−3.04	97.33
−1	−32	1	−42.66	88.35
28	−24	7	79.10	75.80
−1	−21	2	22.32	63.13
−7	−21	−1	224.37	85.97
24	−15	6	79.24	78.97
−11	−14	−6	−45.43	115.69
−10	−9	−4	129.02	75.14
−10	−8	−8	−16.38	110.27
−8	−7	−10	−3.30	89.01
−10	−6	−7	−6.34	79.24
17	−5	4	96.27	72.50
24	−4	4	98.65	82.54
−5	−4	−1	671.00	73.16
−9	−4	−8	−30.77	96.27
13	−2	3	28.39	66.82
11	4	1	218.43	64.71
30	7	−1	−8.32	98.65
25	11	−4	−37.90	95.08
10	12	−5	73.56	59.03
9	12	−6	37.90	52.69
−4	−28	0	−16.77	104.33
25	−27	7	9.51	66.16
−6	−26	−1	−96.54	118.72
38	−23	7	−77.92	98.52
29	−23	7	−20.07	78.18
−5	−23	0	0.00	77.26
2	−22	3	14.26	55.20
2	−19	3	70.39	47.01
−1	−18	2	120.57	59.56
−7	−12	−1	371.09	85.05
32	−9	5	−6.87	95.35
32	−5	4	24.17	97.59
−7	−5	−2	388.13	69.99
27	−4	4	26.41	101.95
21	−4	4	71.45	82.41
29	−1	3	4.75	107.76
−6	0	−3	176.83	48.60
−7	0	−4	2134.11	190.56
28	2	2	47.54	110.01
−6	2	−8	35.79	66.56
−6	3	−6	157.81	60.62
29	4	1	23.90	120.31
7	10	−3	390.50	72.37
17	12	−4	83.86	85.18
14	12	−4	104.46	67.75
9	−37	4	−1.85	72.77
24	−31	7	−95.88	60.62
1	−31	2	−115.29	92.31

−8	−26	−3	−14.53	106.04
33	−22	7	−14.13	90.33
−8	−21	−2	40.28	81.88
−9	−21	−3	64.45	104.72
−10	−18	−4	34.60	91.12
36	−15	6	0.00	95.22
31	−14	6	38.56	95.61
28	−14	6	37.77	92.97
−5	−11	0	360.79	72.50
−8	−9	−2	66.82	72.77
−4	−7	0	844.53	84.25
−9	−5	−4	332.40	92.31
−7	1	−5	25.36	71.18
24	5	1	8.58	83.46
15	5	1	217.50	86.90
20	9	−1	71.05	74.35
11	11	−3	87.29	61.14
12	−43	4	7.26	83.07
0	−34	1	48.20	87.82
36	−22	7	−69.86	100.10
−7	−18	−1	89.80	73.69
20	−9	5	0.00	68.54
27	−8	5	33.41	109.87
−9	−3	−6	75.80	71.84
7	−1	2	165.60	45.03
−8	−1	−7	0.00	59.69
24	0	3	110.54	80.95
21	0	3	185.81	75.14
2	2	0	489.95	46.49
20	3	2	48.20	78.18
9	6	0	297.14	64.45
13	7	0	99.97	84.78
28	9	−2	10.96	89.41
23	10	−2	156.89	111.33
5	11	−6	165.87	40.54
20	12	−4	36.45	107.76
11	−41	4	3.30	77.39
10	−39	4	24.04	75.94
30	−22	7	−49.65	79.90
−3	−22	1	33.41	69.99
−10	−20	−5	70.65	112.65
11	−18	5	166.26	36.71
−10	−15	−4	54.01	80.69
34	−14	6	−51.64	90.46
25	−14	6	−12.15	98.52
−3	−13	1	231.50	60.62
−10	−12	−4	238.37	81.61
−10	−11	−8	4.89	107.23
−9	−9	−9	85.84	94.82
24	−8	5	48.33	78.44
−10	−7	−6	35.00	80.03
−8	−5	−3	268.74	81.35
30	−4	4	−22.98	104.72
−9	−4	−5	33.28	74.75

−6	0	−9	−24.17	103.93
13	2	2	527.85	89.80
23	3	2	102.08	83.99
−5	3	−8	78.58	65.24
−5	4	−6	60.75	53.75
30	6	0	75.67	93.24
25	7	0	43.98	103.40
14	9	−1	76.20	68.14
28	10	−3	−2.51	92.84
11	10	−2	136.29	69.46
23	11	−3	65.24	102.74
7	11	−4	176.43	53.62
13	−37	5	6.87	61.94
1	−36	1	0.00	93.10
24	−34	7	−9.90	51.50
−3	−30	0	102.22	95.74
24	−28	7	6.07	59.30
−2	−27	1	−26.54	89.01
0	−26	2	59.69	72.63
−9	−23	−4	−3.17	109.48
−10	−14	−8	−65.11	93.90
−8	−10	−10	−30.77	102.88
10	−9	4	45.96	51.64
30	−8	5	8.45	96.93
−8	−5	−9	9.64	96.54
6	2	1	1434.18	129.42
28	8	−1	−56.52	95.61
23	9	−1	3.70	100.37
7	9	−2	332.53	67.62
2	−33	2	27.34	85.31
−5	−28	−1	3.96	98.39
34	−21	7	26.94	92.84
−10	−19	−6	−39.09	98.52
−8	−18	−2	−24.83	88.08
−9	−18	−3	30.24	77.78
−10	−9	−7	41.73	94.82
−6	−8	−1	256.07	61.54
3	−5	2	331.34	34.73
18	−4	4	152.93	70.39
32	−1	3	33.02	95.22
14	−1	3	106.84	74.88
27	0	3	−43.84	102.35
18	0	3	108.42	79.90
31	2	2	74.75	98.52
−3	2	−2	412.56	48.60
17	3	2	108.82	76.73
27	5	1	40.28	106.97
−2	7	−6	48.47	38.03
2	8	−3	133.91	32.88
4	−42	1	12.02	101.95
15	−41	5	142.63	71.71
3	−40	1	−10.43	99.18
14	−39	5	11.23	63.92
−6	−28	−2	96.80	104.72

3	−27	3	−2.51	70.12
19	−17	6	−42.66	59.82
−10	−17	−5	97.73	115.29
20	−16	6	−22.85	62.86
−9	−15	−3	−7.92	78.31
29	−13	6	−97.86	94.16
−9	−12	−9	0.00	88.88
−9	−12	−3	23.24	72.24
25	−3	4	18.62	83.73
−8	−3	−8	48.99	85.18
26	3	2	103.80	99.18
−4	4	−8	156.36	62.86
−4	5	−6	146.98	51.24
11	12	−4	28.00	60.62
6	−25	4	25.36	46.88
16	−23	6	73.69	53.35
−8	−23	−3	122.82	111.33
37	−21	7	−3.43	97.73
−3	−19	1	136.02	66.29
18	−18	6	22.85	56.39
21	−15	6	12.94	68.28
−8	−15	−2	52.82	72.37
−10	−15	−7	−120.04	121.50
−5	−14	0	192.68	77.52
−10	−14	−5	115.03	82.93
32	−13	6	−19.28	89.41
−10	−12	−7	31.30	115.55
−10	−11	−5	132.33	81.09
9	−10	4	13.21	46.75
−10	−10	−6	153.32	84.39
−2	−9	1	673.64	69.60
33	−8	5	−26.02	96.80
21	−8	5	−36.98	71.05
−9	−7	−8	−26.54	115.95
13	−6	4	99.18	70.92
−9	−5	−7	51.64	73.16
22	−3	4	11.36	78.44
8	0	2	574.73	71.31
−5	1	−9	−37.64	97.06
−6	2	−5	61.28	62.60
−3	5	−8	62.86	57.05
−2	6	−8	45.16	53.62
−3	6	−6	197.83	48.07
26	10	−2	−106.44	97.73
23	12	−4	−12.28	89.93
8	−45	2	−42.79	97.59
10	−44	3	−48.07	91.12
3	−35	2	−25.22	84.52
−2	−32	0	−26.41	89.27
7	−30	4	100.50	78.44
−4	−25	0	−59.03	81.22
25	−24	7	11.09	70.52
26	−23	7	−28.79	69.33
−7	−23	−2	0.00	90.07

−5	−17	0	198.09	78.71
−10	−16	−6	66.82	119.91
26	−13	6	45.30	103.01
33	−4	4	74.88	93.50
28	−3	4	140.12	121.36
30	0	3	0.00	99.84
−7	0	−7	363.43	79.76
−6	1	−4	565.22	74.22
3	3	0	15.19	47.54
12	5	1	668.89	94.69
22	6	1	−2.25	78.05
19	6	1	−20.73	77.26
28	7	0	39.22	101.55
20	8	0	0.00	70.65
2	9	−4	660.44	63.65
26	11	−3	0.00	95.48
7	−43	2	−34.73	98.39
6	−36	3	−10.17	80.16
−4	−30	−1	5.68	90.20
−8	−25	−4	−29.05	101.03
−9	−22	−5	−9.90	102.48
−9	−20	−4	166.53	106.97
14	−12	5	62.73	50.71
15	−11	5	62.33	56.52
−9	−8	−4	6.60	74.61
28	−7	5	98.91	108.55
25	−7	5	−14.79	91.65
−8	−2	−6	1.45	65.63
26	9	−1	−48.86	102.08
18	11	−2	68.14	78.05
18	12	−3	173.79	84.39
9	−42	3	−56.39	86.50
6	−41	2	35.39	94.56
5	−39	2	−6.21	91.25
7	−38	3	−61.28	88.08
4	−37	2	−20.87	90.73
−1	−34	0	0.00	90.07
−5	−30	−2	67.75	92.05
−1	−29	1	−50.32	101.82
35	−20	7	0.13	87.29
22	−14	6	77.52	71.05
35	−13	6	−100.76	100.50
16	−10	5	7.26	59.30
−8	−8	−9	37.51	95.48
−7	−4	−9	−22.32	105.12
−8	−4	−4	149.10	79.37
22	1	3	74.75	80.56
29	3	2	17.30	102.08
7	3	1	1304.50	119.65
30	5	1	−1.32	100.76
25	6	1	−108.95	103.14
16	6	1	52.96	77.12
23	8	0	0.00	86.76
11	9	−1	297.40	81.35

21	11	−2	0.00	101.42
15	11	−2	98.39	74.61
15	12	−3	88.22	69.86
14	13	−5	42.52	81.61
8	−40	3	16.64	84.65
11	−30	5	85.05	48.47
24	−25	7	67.35	66.29
17	−19	6	−60.88	51.37
13	−13	5	27.20	47.81
30	−12	6	−22.85	89.14
−9	−10	−8	20.73	109.21
−7	−9	−10	14.53	88.74
31	−7	5	−120.44	101.03
−9	−6	−6	24.43	74.22
14	−5	4	270.86	81.61
−6	−4	−2	1031.00	104.72
31	−3	4	30.64	93.37
19	−3	4	347.32	83.86
25	1	3	−29.71	84.65
14	3	2	686.72	105.25
21	4	2	36.05	82.54
21	12	−3	−72.24	106.31
0	−36	0	47.67	96.67
−3	−32	−1	46.62	88.61
23	−29	7	−45.69	56.65
1	−28	2	128.89	85.84
0	−23	2	59.43	65.37
29	−20	7	34.34	72.90
−6	−20	−1	83.73	78.18
−8	−20	−3	−50.84	85.71
6	−19	4	147.91	33.02
−9	−17	−4	51.77	85.71
−9	−13	−8	34.73	103.14
27	−12	6	−0.13	98.78
17	−9	5	72.11	64.31
−7	−8	−2	110.80	70.52
22	−7	5	18.62	72.63
−4	−3	−1	2401.53	204.96
−8	−3	−5	230.45	80.95
−7	−2	−8	34.73	77.52
15	0	3	67.09	78.44
19	1	3	70.12	72.90
9	1	2	65.37	49.79
24	4	2	86.76	89.27
10	7	0	927.46	105.38
14	8	0	298.46	83.86
18	10	−1	173.66	76.60
1	−38	0	79.10	96.80
−5	−25	−1	79.37	89.67
−7	−25	−3	46.35	107.89
−2	−24	1	63.52	68.28
−9	−19	−5	−135.36	117.53
33	−12	6	0.66	89.41
−6	−11	−1	395.92	79.50

-9	-11	-4	103.80	82.27
-8	-8	-3	147.51	80.03
-9	-8	-7	62.73	83.33
-7	-4	-3	1177.46	119.52
28	1	3	-64.58	111.20
-5	3	-5	499.59	75.01
18	4	2	2.77	72.77
26	8	0	33.81	116.21
29	10	-2	-29.05	86.90
21	10	-1	-17.96	90.20
-2	-34	-1	-39.88	90.46
0	-31	1	59.56	92.71
-3	-27	0	-30.11	97.59
-6	-25	-2	12.41	102.74
6	-22	4	69.86	40.67
-4	-22	0	30.90	73.69
-7	-20	-2	-53.62	80.69
-9	-14	-4	62.86	78.97
-8	-11	-9	-20.21	96.80
34	-7	5	0.00	94.69
-8	-6	-8	0.00	100.23
-8	-4	-7	135.89	73.29
26	-2	4	-37.64	96.01
23	-2	4	86.10	80.03
-6	1	-7	34.20	66.29
4	4	0	452.04	61.41
28	6	1	-24.43	98.78
29	9	-1	-17.30	88.08
15	10	-1	126.12	72.24
24	11	-2	-21.66	100.63
8	11	-3	16.24	60.35
18	13	-4	94.42	103.14
23	-32	7	2.51	48.73
4	-29	3	99.44	89.01
3	-24	3	97.33	59.30
-8	-22	-4	51.90	122.55
30	-19	7	-27.60	83.33
-9	-18	-6	0.00	110.54
-6	-17	-1	3.83	70.12
-9	-17	-7	-25.09	89.67
18	-8	5	172.74	70.39
29	-6	5	-10.17	98.65
26	-6	5	-1.06	102.88
15	-4	4	71.84	76.20
8	-4	3	430.92	59.30
-2	3	-2	349.83	38.83
27	4	2	1.85	110.27
24	10	-1	-93.50	114.23
29	11	-3	-70.26	96.40
12	11	-2	222.79	67.88
24	12	-3	19.55	93.63
12	12	-3	115.03	66.69
15	13	-4	62.46	76.73
23	-26	7	-11.36	61.80

−8	−17	−3	−22.85	81.35
−9	−16	−5	−7.79	86.10
−6	−14	−1	189.64	86.63
−9	−14	−7	23.90	115.42
24	−12	6	0.00	92.31
−9	−11	−7	6.87	113.84
−9	−10	−5	71.45	79.50
−9	−9	−6	55.47	80.82
9	−3	3	−6.87	48.86
29	−2	4	66.16	103.93
−5	2	−4	379.94	61.01
8	10	−2	137.08	67.35
21	13	−4	−0.13	92.31
11	13	−5	27.34	57.84
−6	−27	−3	6.07	105.78
34	−18	7	0.66	94.16
−7	−17	−2	57.71	73.03
12	−14	5	50.18	45.30
31	−11	6	59.56	88.74
28	−11	6	83.33	92.44
−8	−11	−3	−11.23	79.90
−4	−10	0	1007.89	101.82
23	−6	5	14.26	77.52
−3	−6	0	364.22	51.77
7	−5	3	272.71	45.83
−6	−3	−9	67.48	103.27
20	−2	4	−20.47	75.94
−7	−1	−6	29.71	61.94
31	1	3	14.79	96.93
8	4	1	267.29	58.50
13	6	1	201.53	77.26
23	7	1	−0.40	87.95
20	7	1	0.00	87.03
7	12	−5	168.25	50.84
1	−33	1	−11.36	86.63
2	−30	2	78.31	98.12
−2	−29	0	145.93	107.10
−4	−27	−1	79.90	107.89
0	−20	2	51.50	56.79
31	−18	7	0.00	86.10
−9	−15	−6	−36.71	106.04
−8	−14	−3	71.71	72.63
−9	−13	−5	17.70	78.58
8	−11	4	32.35	39.22
−7	−11	−2	186.21	84.52
−6	−8	−10	58.37	88.74
−7	−7	−9	40.41	96.93
32	−6	5	−78.58	91.52
10	−2	3	123.61	55.73
−6	−1	−8	−13.21	70.78
16	1	3	−14.66	82.67
23	2	3	0.00	80.69
10	2	2	294.50	64.05
15	4	2	205.35	86.76

29	8	0	3.04	98.91
21	9	0	55.47	83.07
18	9	0	0.00	79.50
−7	−22	−3	−64.71	90.20
−2	−21	1	38.96	65.63
26	−20	7	74.35	75.14
−7	−14	−2	205.75	83.20
34	−11	6	13.21	94.69
−8	−9	−8	−47.41	112.38
19	−7	5	246.43	74.22
−5	−7	−1	574.33	74.88
16	−3	4	125.85	71.45
32	−2	4	88.88	89.93
26	2	3	76.73	103.54
20	2	3	43.58	74.75
30	4	2	0.00	95.35
−4	4	−5	1034.70	103.40
17	7	1	165.74	83.86
27	11	−2	6.34	88.61
8	12	−4	−21.53	52.30
6	−43	1	3.96	87.42
2	−35	1	15.98	84.25
−1	−26	1	0.00	72.11
−7	−24	−4	34.07	103.27
−8	−21	−5	−20.47	108.95
16	−20	6	46.09	46.75
27	−19	7	17.30	73.29
−8	−19	−4	−12.15	92.05
0	−17	2	470.53	78.05
25	−11	6	0.00	96.40
−8	−7	−4	118.33	86.63
−8	−5	−6	144.47	73.69
−5	2	−7	165.21	63.79
22	5	2	143.29	80.03
5	5	0	28.26	42.79
26	7	1	169.17	121.36
24	9	0	0.00	108.42
27	10	−1	42.92	95.88
12	10	−1	13.60	74.09
27	12	−3	27.07	88.48
19	12	−2	−58.37	83.86
12	13	−4	64.18	62.33
14	−44	4	−0.79	81.75
5	−41	1	2.91	95.61
3	−37	1	12.55	90.46
9	−34	4	−27.07	68.01
5	−31	3	58.50	87.95
−6	−22	−2	−32.35	77.12
3	−15	3	168.51	43.71
−8	−15	−8	−12.28	96.01
−2	−12	1	663.47	77.12
−8	−12	−8	24.96	103.93
−6	−11	−10	101.95	92.97
27	−5	5	−85.71	108.42

−7	−2	−5	88.74	77.92
27	−1	4	58.64	117.27
24	−1	4	−0.13	79.24
11	−1	3	545.81	80.69
29	2	3	−35.66	96.80
25	5	2	131.93	103.80
19	5	2	32.75	79.10
11	8	0	253.82	82.54
10	−46	2	0.00	95.35
3	−32	2	−18.09	87.95
−1	−31	0	−45.30	100.76
35	−30	8	−4.09	80.16
−3	−29	−1	150.81	101.95
1	−25	2	−26.54	63.92
−3	−24	0	−2.64	76.20
−8	−20	−6	22.58	94.95
28	−18	7	15.45	77.52
35	−17	7	−63.92	95.88
32	−17	7	19.94	89.93
−4	−13	0	219.49	67.22
32	−10	6	−21.79	88.22
29	−10	6	27.47	91.65
−7	−10	−9	74.48	92.05
−8	−7	−7	−18.22	76.60
30	−5	5	8.85	98.39
−7	−5	−8	40.54	83.99
−7	−3	−7	184.36	74.75
2	7	−2	731.62	68.01
3	9	−3	570.11	61.54
19	11	−1	104.06	80.16
22	12	−2	0.00	110.93
19	13	−3	112.25	103.40
16	13	−3	17.43	75.67
34	−31	8	−56.65	80.82
36	−29	8	−52.56	85.05
−4	−29	−2	40.41	101.42
7	−27	4	49.13	58.90
23	−23	7	48.60	64.84
3	−21	3	229.52	55.86
−4	−16	0	−15.85	81.09
−8	−16	−4	−76.60	77.26
19	−14	6	0.00	60.22
20	−13	6	23.51	61.54
−7	−13	−9	22.98	89.27
20	−6	5	−3.04	67.35
6	−6	3	67.48	32.75
24	−5	5	9.90	84.39
−5	−2	−9	0.00	100.10
30	−1	4	39.75	106.97
21	−1	4	−1.32	75.27
11	3	2	878.73	100.10
28	5	2	0.00	103.67
9	5	1	−1.58	54.54
27	9	0	−7.40	95.88

22	11	−1	0.00	98.65
22	13	−3	51.50	96.40
13	−42	4	−98.39	79.24
10	−36	4	−41.60	69.60
12	−32	5	16.51	52.56
37	−28	8	−42.13	87.56
−7	−19	−3	104.33	77.12
−2	−18	1	41.60	66.82
−8	−18	−5	−42.92	101.82
29	−17	7	30.11	82.01
−8	−16	−7	62.07	107.23
18	−15	6	77.65	58.37
21	−12	6	0.13	68.14
26	−10	6	−22.05	104.59
−8	−10	−4	101.42	81.61
−7	−7	−3	1117.63	123.48
33	−5	5	−25.09	90.99
−5	−3	−2	703.62	77.12
17	−2	4	65.50	68.94
17	2	3	47.94	70.52
−4	3	−4	369.90	55.33
8	9	−1	10.56	58.64
16	11	−1	34.20	73.16
12	−45	3	29.85	89.14
9	−44	2	97.59	95.88
12	−40	4	60.88	74.61
11	−38	4	8.06	69.99
4	−34	2	39.35	83.99
0	−33	0	−8.32	86.63
38	−27	8	24.70	91.65
22	−27	7	55.07	56.92
−6	−26	−4	−54.01	99.31
−6	−24	−3	37.90	109.21
−7	−23	−5	85.71	104.99
−5	−19	−1	52.69	69.60
33	−16	7	81.88	95.35
−2	−15	1	205.49	64.45
−8	−13	−4	108.03	75.80
−8	−10	−7	−18.09	86.24
−5	−7	−10	13.34	77.65
−6	−7	−2	24.17	59.69
−6	−6	−9	−72.77	99.05
−6	−3	−3	53.22	52.69
12	0	3	988.87	111.59
−5	0	−8	37.77	67.48
−6	0	−6	5.94	58.90
16	5	2	182.77	77.26
14	7	1	578.43	98.39
25	12	−2	41.60	92.44
8	−42	2	70.78	92.31
−2	−31	−1	58.64	101.42
−3	−31	−2	0.00	93.24
0	−28	1	70.65	87.29
−8	−17	−6	−34.07	114.10

−8	−13	−7	0.00	110.01
22	−11	6	41.34	69.20
−8	−8	−6	62.33	73.43
24	3	3	23.37	83.86
−4	3	−7	138.27	61.80
−3	5	−5	497.87	63.79
6	6	0	323.55	55.73
21	8	1	−64.84	75.54
25	11	−1	−47.67	97.33
13	12	−2	36.85	69.99
11	−43	3	−25.75	88.35
7	−40	2	−5.81	87.95
5	−36	2	20.34	76.20
1	−35	0	52.16	87.42
34	−28	8	1.06	77.26
4	−26	3	83.33	59.03
−5	−24	−2	1.32	90.99
−7	−21	−4	0.00	99.18
−6	−19	−2	−24.56	75.80
3	−18	3	7.40	45.69
30	−16	7	10.04	83.33
17	−16	6	25.88	54.94
−8	−15	−5	52.03	78.84
1	−10	2	68.01	30.24
30	−9	6	−5.02	90.20
−8	−9	−5	63.13	73.82
−7	−8	−8	−6.47	103.93
21	−5	5	40.15	74.61
25	0	4	99.31	95.22
27	3	3	−80.82	108.69
21	3	3	12.41	77.52
24	8	1	0.00	104.99
18	8	1	1.72	74.75
9	11	−2	119.25	70.65
9	12	−3	29.58	54.15
25	13	−3	78.84	93.63
13	13	−3	3.83	66.82
19	14	−4	133.25	101.55
3	−39	0	102.88	96.40
6	−38	2	−48.60	91.78
2	−37	0	58.50	92.44
−1	−33	−1	35.79	84.78
−2	−33	−2	−11.75	89.80
22	−30	7	−12.28	47.28
33	−29	8	−49.79	77.52
35	−27	8	−46.35	80.95
−7	−16	−3	173.79	78.44
−8	−14	−6	0.00	75.14
−8	−12	−5	19.81	73.29
−8	−11	−6	48.47	79.90
−5	−10	−1	157.02	67.35
33	−9	6	74.48	91.25
27	−9	6	52.03	102.88
−3	−2	−1	1051.47	101.55

28	0	4	49.79	115.55
23	6	2	−15.19	87.56
22	10	0	−13.34	91.52
19	10	0	−34.60	75.54
3	10	−4	401.07	52.56
6	12	−6	0.00	38.96
16	14	−4	−12.68	94.16
10	−41	3	45.96	85.71
18	−36	6	−50.05	35.00
0	−35	−1	77.78	88.61
36	−26	8	34.60	82.54
−2	−26	0	−6.47	79.50
22	−24	7	7.79	60.48
−1	−23	1	−34.86	68.54
25	−18	7	35.66	74.48
−5	−16	−1	45.30	78.18
23	−10	6	−35.79	86.76
−5	−10	−10	15.72	89.14
−7	−10	−3	260.42	90.33
−1	−8	1	67.62	34.07
18	−1	4	51.24	76.20
−4	−1	−9	−61.94	99.18
22	0	4	8.85	80.56
13	1	3	284.72	84.65
30	3	3	−15.19	91.91
12	4	2	429.86	85.71
26	6	2	14.00	111.33
20	6	2	−3.30	79.76
25	10	0	−20.34	101.16
9	−39	3	56.92	82.14
8	−37	3	−59.43	81.09
32	−30	8	−39.49	75.67
2	−27	2	29.98	68.94
−5	−26	−3	−41.20	114.36
24	−19	7	−14.92	70.39
26	−17	7	17.30	76.60
34	−15	7	−11.75	91.78
31	−15	7	78.05	87.42
−7	−14	−8	7.66	103.67
−7	−13	−3	53.09	75.67
−7	−11	−8	64.71	109.48
−6	−9	−9	−7.40	92.31
−6	−4	−8	10.56	78.31
31	0	4	−57.71	92.18
10	6	1	29.85	64.31
3	8	−2	578.16	61.14
28	11	−1	39.49	88.08
13	11	−1	103.54	67.88
28	12	−2	−32.62	90.07
20	−40	6	−28.66	47.81
1	−30	1	13.47	101.16
37	−25	8	−57.97	96.80
1	−22	2	25.22	59.43
−7	−20	−5	80.43	113.44

−6	−16	−2	−1.45	72.63
11	−15	5	126.12	40.54
−5	−13	−1	−33.94	75.01
−6	−12	−9	−24.56	88.48
−6	−10	−2	318.53	82.01
15	−8	5	120.97	61.80
−7	−6	−7	92.31	73.95
−7	−6	−4	214.20	80.29
−6	−2	−7	189.90	71.97
−6	−1	−5	79.24	68.41
18	3	3	145.27	71.71
16	10	0	138.66	74.22
20	13	−2	46.22	106.84
12	14	−5	32.35	66.29
13	−34	5	−9.51	54.81
−3	−26	−1	40.67	90.33
−6	−23	−4	70.26	108.55
−6	−21	−3	79.63	78.58
23	−20	7	69.33	69.46
−7	−19	−6	2.64	114.89
−7	−18	−4	−0.66	79.90
27	−16	7	−24.04	78.18
−6	−13	−2	80.03	84.92
24	−9	6	−15.58	95.74
14	−9	5	56.65	57.05
31	−8	6	54.41	87.82
16	−7	5	−3.83	63.52
−7	−5	−5	112.65	74.88
−4	1	−8	39.22	67.22
−3	4	−7	71.05	55.07
29	6	2	−5.55	96.80
−2	6	−5	61.41	41.60
7	7	0	1949.35	173.40
15	8	1	34.34	70.39
20	12	−1	23.11	94.42
17	13	−2	−56.13	74.09
17	−42	5	42.52	70.39
19	−38	6	−0.40	42.66
−4	−28	−3	−16.64	97.99
33	−26	8	55.20	77.78
34	−25	8	24.43	83.33
38	−24	8	−22.71	98.78
16	−17	6	−28.13	50.98
28	−8	6	−26.41	95.08
17	−6	5	−5.15	67.75
11	−5	4	260.03	67.75
−5	−5	−9	18.22	104.72
12	−4	4	162.83	71.31
−5	1	−6	−46.88	56.65
25	4	3	−8.06	108.16
17	6	2	−61.67	75.01
28	10	0	23.77	92.05
9	10	−1	415.20	83.73
4	10	−3	751.56	78.84

23	12	−1	0.00	109.87
23	13	−2	−102.74	100.89
9	13	−4	221.20	62.60
20	14	−3	158.74	100.76
13	14	−4	32.62	67.35
2	−32	1	−75.94	94.03
31	−31	8	2.77	62.73
8	−29	4	−54.28	78.84
−1	−28	0	−53.75	95.88
32	−27	8	34.20	75.27
35	−24	8	19.81	83.46
−4	−21	−1	168.38	78.31
−5	−21	−2	87.03	78.84
28	−15	7	16.38	81.09
−7	−15	−7	−8.72	113.57
35	−14	7	34.34	97.46
32	−14	7	2.64	87.29
13	−10	5	44.50	53.48
−7	−9	−7	99.97	78.05
13	−3	4	376.90	84.65
19	0	4	162.30	80.82
26	1	4	33.81	108.29
14	2	3	69.73	83.07
28	4	3	0.53	100.89
22	4	3	2.91	78.71
−3	4	−4	521.64	58.37
17	12	−1	92.97	74.75
8	13	−5	68.67	52.96
17	14	−3	104.99	90.59
24	−37	7	−0.13	20.21
14	−36	5	140.64	60.62
−7	−17	−5	122.55	85.58
−7	−15	−4	51.90	81.48
−3	−9	0	483.61	64.31
−4	−6	−1	1581.83	140.64
29	1	4	27.73	96.14
23	1	4	0.00	78.44
13	5	2	195.71	83.86
22	9	1	5.68	80.95
23	14	−3	0.53	85.05
12	−47	2	−67.75	97.33
7	−42	1	−109.48	98.39
16	−40	5	129.68	67.22
3	−34	1	22.32	84.52
3	−29	2	35.00	85.71
31	−28	8	−5.68	71.71
5	−28	3	42.13	68.67
−3	−28	−2	159.53	113.97
11	−27	5	53.35	47.94
36	−23	8	95.35	95.08
−3	−18	0	128.10	71.05
−7	−16	−6	29.85	91.91
−7	−12	−7	37.64	95.22
−7	−9	−4	37.77	78.97

25	−8	6	−42.66	100.63
−6	−7	−8	51.64	100.23
−7	−7	−6	471.19	87.69
18	−5	5	53.22	71.45
−3	0	−9	111.06	99.05
24	7	2	0.00	106.97
21	7	2	105.78	78.84
11	7	1	430.78	83.46
19	9	1	−9.24	73.95
26	12	−1	−46.22	89.27
26	13	−2	18.49	89.93
6	−40	1	30.77	94.42
15	−38	5	37.11	60.09
4	−36	1	41.20	84.12
0	−30	0	0.00	107.37
−2	−28	−1	−28.39	113.04
0	−25	1	70.65	67.88
−5	−25	−4	0.53	114.23
15	−24	6	0.00	30.24
−6	−22	−5	−66.95	110.54
15	−21	6	0.00	41.20
−1	−20	1	89.54	62.86
−6	−18	−3	116.61	78.97
−6	−16	−8	38.17	95.48
29	−14	7	33.15	84.39
−7	−12	−4	153.85	71.31
−4	−9	−10	0.00	89.01
−6	−6	−3	297.40	69.86
14	−2	4	75.67	82.54
19	4	3	43.18	80.29
27	7	2	−43.05	99.31
4	9	−2	63.39	43.84
23	11	0	−14.79	112.12
20	11	0	16.11	87.29
10	12	−2	48.99	59.56
14	13	−2	92.97	72.63
5	−38	1	0.40	91.65
−2	−23	0	−28.53	70.52
−5	−23	−3	0.00	87.56
−6	−21	−6	−6.07	100.76
−7	−14	−5	136.82	77.52
33	−13	7	−15.45	94.03
−4	−12	−10	12.02	91.12
−5	−8	−9	46.09	97.46
32	−7	6	0.00	89.14
29	−7	6	−6.60	89.01
−5	−3	−8	15.05	71.58
−5	−2	−3	541.71	68.41
−3	2	−8	9.24	62.20
−2	5	−7	87.69	52.30
−1	7	−5	92.44	37.64
8	8	0	543.96	76.33
28	9	1	57.31	90.86
10	13	−3	−26.02	60.62

14	14	−3	−80.16	68.14
−2	−30	−2	−52.82	101.55
30	−29	8	26.68	67.88
21	−25	7	−6.47	55.47
32	−24	8	−79.24	79.10
7	−24	4	79.76	45.30
37	−22	8	−7.40	99.31
−6	−20	−4	−16.77	83.33
1	−19	2	86.50	59.82
−6	−13	−8	29.58	112.65
18	−12	6	−17.04	60.35
19	−11	6	6.74	62.33
−7	−11	−5	298.99	82.80
20	−10	6	61.67	64.84
−6	−10	−8	−96.40	110.40
−7	−10	−6	148.70	78.44
−2	−5	0	−33.81	43.58
−6	−3	−6	27.20	68.54
27	−2	5	−68.14	100.89
−4	−2	−2	59.82	42.66
15	−1	4	−24.96	76.07
−5	−1	−7	52.43	67.75
20	1	4	3.30	82.67
15	3	3	15.58	78.18
26	11	0	31.30	97.86
17	11	0	64.97	78.05
14	12	−1	154.12	75.41
16	−45	4	−6.07	73.95
11	−45	2	−30.11	94.56
30	−32	8	15.05	70.12
1	−32	0	99.31	93.10
−1	−30	−1	0.00	102.88
31	−25	8	−31.83	74.48
33	−23	8	0.00	77.52
4	−23	3	79.24	54.54
−4	−18	−1	−20.07	68.01
−5	−18	−2	47.41	70.39
−3	−15	0	82.27	69.46
−5	−14	−9	48.20	89.14
30	−13	7	−33.02	83.46
−3	−12	0	411.63	64.71
12	−11	5	45.83	50.45
−5	−11	−9	−14.00	93.63
26	−7	6	220.94	103.40
−5	−6	−2	401.86	64.97
−6	−5	−7	19.81	69.86
−4	−4	−9	−17.30	100.37
30	−2	5	54.94	91.39
26	5	3	−125.85	106.57
14	6	2	50.71	82.80
18	7	2	0.00	76.60
16	9	1	2.11	80.69
17	15	−4	24.17	102.74
14	−46	3	2.51	91.78

4	−31	2	0.00	96.01
−4	−27	−4	9.90	94.42
−5	−24	−5	103.80	101.42
−4	−23	−2	1.85	80.95
34	−22	8	66.82	87.56
24	−16	7	−23.51	75.14
25	−15	7	32.09	76.20
−6	−15	−3	19.28	71.45
21	−9	6	0.00	71.84
24	−2	5	−11.62	98.39
−5	0	−5	647.63	89.80
27	2	4	−11.23	99.71
24	2	4	0.00	95.88
23	5	3	5.68	84.92
10	11	−1	475.29	91.78
4	11	−4	26.94	47.28
10	−43	2	67.35	92.84
2	−34	0	−55.73	94.82
0	−32	−1	6.07	95.08
−1	−32	−2	−18.22	89.01
9	−31	4	−23.77	71.97
30	−26	8	0.00	74.61
2	−24	2	45.30	65.63
23	−17	7	56.92	71.97
−6	−17	−7	0.00	111.72
26	−14	7	−0.26	86.24
17	−13	6	54.94	56.39
34	−12	7	152.66	91.52
−6	−9	−3	32.22	78.05
5	−7	3	248.01	34.47
30	2	4	25.09	96.93
−4	2	−6	121.63	56.13
29	5	3	92.84	95.08
12	8	1	284.59	93.90
5	11	−3	192.28	58.11
21	13	−1	−13.73	103.54
21	14	−2	−29.98	101.95
5	−40	0	88.88	96.54
4	−38	0	89.80	92.18
3	−36	0	0.00	90.46
23	−35	7	5.28	22.98
0	−34	−2	4.36	87.95
6	−30	3	0.00	86.76
21	−28	7	0.00	46.22
1	−27	1	−22.05	73.69
−4	−25	−3	−12.02	106.31
35	−21	8	−8.06	92.05
−6	−19	−5	1.45	93.24
−6	−18	−6	0.00	106.84
31	−12	7	66.43	84.65
−6	−12	−3	0.00	82.41
22	−8	6	−62.99	85.71
30	−6	6	33.94	95.88
−6	−5	−4	238.63	75.14

−6	−4	−5	97.46	80.82
−5	−1	−4	580.41	76.33
16	0	4	281.55	78.31
−1	4	−2	280.50	24.56
20	5	3	−19.02	78.58
24	13	−1	0.13	93.90
18	13	−1	−39.09	87.03
24	14	−2	90.73	89.93
18	14	−2	30.77	88.88
13	−44	3	0.00	86.76
15	−43	4	0.00	78.58
9	−41	2	−30.24	88.74
1	−34	−1	15.58	85.05
5	−33	2	14.00	85.84
22	−18	7	−40.28	70.26
−6	−17	−4	64.18	78.71
1	−16	2	276.27	62.20
27	−13	7	−89.54	83.46
−1	−11	1	73.29	42.92
−3	−8	−10	3.04	85.97
−6	−8	−7	33.94	73.69
27	−6	6	−21.00	93.90
21	2	4	21.66	74.35
−1	6	−7	50.58	46.09
25	8	2	−0.26	117.93
22	8	2	13.47	90.99
9	9	0	−1.72	66.29
26	10	1	146.59	94.42
5	10	−2	484.93	66.95
21	15	−3	26.02	91.65
2	−36	−1	0.40	89.93
6	−35	2	−91.39	82.80
−1	−25	0	0.00	73.95
−5	−22	−4	−35.92	94.82
36	−20	8	7.40	95.74
−5	−20	−3	−8.72	73.95
15	−18	6	−15.19	45.30
−4	−15	−1	87.82	76.60
−5	−15	−2	−3.17	84.25
−6	−14	−7	−10.56	104.33
−4	−9	−1	181.45	54.54
23	−7	6	36.98	93.37
−5	−6	−8	131.00	80.95
28	−1	5	38.17	102.08
7	2	2	765.69	82.01
−2	3	−8	3.04	57.45
14	11	0	142.89	70.78
10	14	−4	211.43	64.45
18	15	−3	−1.85	100.23
14	15	−4	−29.19	79.63
8	−39	2	76.60	90.86
29	−30	8	−6.60	65.90
29	−27	8	−88.88	72.77
−3	−27	−3	−40.41	106.97

−3	−25	−2	−39.35	89.41
31	−22	8	16.90	77.52
−1	−14	1	177.23	58.64
28	−12	7	−1.98	84.39
−6	−11	−7	0.00	87.42
−5	−9	−2	55.86	63.79
−4	−7	−9	61.28	105.91
−6	−6	−6	75.27	73.95
−4	−2	−8	3.04	68.67
25	−1	5	37.77	110.27
8	3	2	591.90	77.52
15	7	2	16.38	72.90
28	8	2	0.00	90.07
19	8	2	7.79	75.27
24	12	0	65.24	98.52
21	12	0	164.68	100.89
27	13	−1	41.86	91.39
11	13	−2	52.43	63.65
27	14	−2	−23.37	93.10
15	14	−2	59.69	69.20
24	15	−3	−139.59	90.07
12	−42	3	44.77	84.39
14	−41	4	67.88	75.54
10	−33	4	0.00	66.29
7	−32	3	−90.33	85.44
−2	−25	−1	0.00	73.29
30	−23	8	39.35	75.54
32	−21	8	0.00	76.60
−6	−16	−5	113.18	78.44
−5	−15	−8	−62.33	97.33
−6	−15	−6	16.77	86.50
16	−14	6	−29.32	54.28
−4	−12	−1	774.01	98.25
−5	−12	−2	73.95	82.41
32	−11	7	−42.52	90.99
−3	−11	−10	106.97	88.74
17	1	4	74.35	72.77
6	1	2	186.60	47.15
28	3	4	45.83	91.12
−2	4	−3	675.23	62.20
24	6	3	−21.79	108.55
17	10	1	32.75	76.86
7	13	−6	5.02	42.26
9	14	−5	18.75	55.20
2	−29	1	−4.09	86.10
0	−22	1	95.61	65.77
33	−20	8	−94.03	94.03
−6	−14	−4	3.83	72.77
−5	−9	−8	10.04	97.06
−6	−8	−4	−4.23	80.82
24	−6	6	−18.49	99.84
31	−5	6	−96.80	91.65
−3	−3	−9	0.00	97.99
−4	0	−7	166.93	66.95

25	3	4	30.64	105.65
9	4	2	64.31	57.18
−2	5	−4	401.07	46.88
27	6	3	−34.86	97.20
13	9	1	36.58	75.01
27	12	0	63.92	93.50
18	12	0	56.92	75.80
15	13	−1	9.77	73.43
11	14	−3	23.11	64.05
11	−40	3	115.03	85.84
11	−35	4	−37.51	67.62
8	−34	3	−63.13	78.97
29	−24	8	−29.85	69.99
−5	−21	−5	23.24	107.63
−4	−20	−2	58.24	77.52
34	−19	8	12.41	90.99
21	−19	7	31.30	65.50
−4	−13	−9	−17.70	87.16
7	−12	4	11.36	19.41
−5	−12	−8	−13.60	109.87
29	−11	7	22.85	83.33
−6	−11	−4	86.90	75.01
−4	−10	−9	99.57	98.91
28	−5	6	−12.28	92.18
−5	−2	−6	30.51	61.54
9	0	3	571.69	75.80
10	1	3	567.07	84.65
17	5	3	67.35	79.37
21	6	3	82.80	79.24
3	7	−1	1266.99	112.38
0	7	−7	567.86	63.26
11	12	−1	277.33	71.97
12	−37	4	42.00	70.12
−2	−29	−3	0.00	97.20
0	−27	0	31.17	77.52
3	−26	2	233.62	70.78
−4	−24	−4	30.24	111.72
−3	−20	−1	16.64	74.22
−5	−20	−6	22.32	113.70
−6	−13	−5	19.94	76.60
−6	−9	−6	192.28	80.29
−5	−4	−7	4.75	70.12
22	−1	5	22.58	80.56
8	−1	3	125.85	51.90
11	2	3	40.94	68.67
22	3	4	0.66	82.14
9	−43	1	0.00	94.69
10	−38	3	0.00	80.29
9	−36	3	36.58	75.41
3	−31	1	−39.75	101.42
−4	−22	−3	116.35	84.92
−5	−19	−4	47.94	76.99
−5	−17	−3	59.16	77.92
11	−12	5	0.79	43.71

33	−10	7	−27.47	90.99
−6	−10	−5	15.05	71.45
15	−5	5	−44.11	62.99
29	0	5	−3.17	99.57
5	0	2	72.77	42.92
18	2	4	92.97	73.16
−1	4	−8	0.53	54.67
10	5	2	392.62	75.94
6	5	1	85.05	44.50
2	6	−1	1520.02	130.61
23	9	2	28.66	105.38
24	11	1	−5.81	106.04
6	11	−2	128.63	61.54
6	12	−3	0.00	60.09
5	12	−4	−21.39	45.69
28	−28	8	−18.62	68.01
−1	−27	−1	11.09	83.07
5	−25	3	20.07	54.28
4	−20	3	216.45	51.37
35	−18	8	6.21	88.74
−5	−16	−7	71.84	111.06
14	−6	5	−6.07	61.80
25	−5	6	35.52	100.50
−5	−5	−3	445.71	69.86
26	0	5	187.26	107.89
−3	3	−6	836.08	91.52
5	4	1	810.99	83.59
16	8	2	0.00	77.78
26	9	2	19.02	94.03
22	14	−1	91.39	104.33
22	15	−2	−14.79	95.08
19	15	−2	−44.11	100.76
8	−41	1	0.00	91.25
−3	−26	−4	90.73	111.72
28	−25	8	−29.85	71.58
−4	−23	−5	54.28	111.46
−4	−21	−7	−31.30	100.63
23	−14	7	−9.11	75.67
24	−13	7	48.07	78.31
25	−12	7	−3.70	83.73
30	−10	7	−28.39	85.58
8	−8	4	20.60	46.75
29	−4	6	0.00	92.44
−4	−1	−3	194.13	48.33
−4	1	−5	271.91	66.69
12	3	3	217.50	81.09
7	6	1	20.87	53.35
1	8	−7	224.64	25.88
20	9	2	47.94	74.88
27	11	1	45.56	89.67
15	12	0	39.49	77.12
25	14	−1	0.00	86.37
19	14	−1	140.38	110.54
25	15	−2	−6.07	93.90

13	−46	2	−0.92	93.24
7	−39	1	122.82	92.31
4	−33	1	64.05	89.14
1	−29	0	−64.45	95.74
−1	−29	−2	63.13	107.10
20	−23	7	−5.02	55.73
2	−21	2	57.71	56.52
30	−20	8	−23.77	77.65
31	−19	8	−102.35	84.25
−2	−17	0	47.41	81.09
−4	−17	−8	48.99	98.12
22	−15	7	−20.07	71.05
19	−8	6	−2.51	67.35
13	−7	5	126.65	59.82
−2	−1	−1	510.55	51.90
29	4	4	114.63	97.86
26	4	4	60.48	98.65
18	6	3	113.18	78.31
11	6	2	242.20	83.99
25	7	3	0.00	101.82
14	10	1	209.18	76.20
22	13	0	−25.62	112.38
6	−37	1	8.85	84.78
5	−35	1	116.87	83.86
0	−29	−1	21.92	104.86
8	−26	4	−1.32	50.45
−1	−22	0	93.10	72.11
−3	−22	−2	−68.80	76.60
−4	−22	−6	−2.38	104.59
29	−21	8	−3.96	74.48
32	−18	8	37.37	89.14
−5	−18	−5	0.00	77.52
−4	−17	−2	105.38	72.37
−5	−17	−6	1.72	95.61
15	−15	6	57.45	50.58
−5	−14	−3	106.84	75.27
−2	−13	−10	−5.41	87.16
−5	−13	−7	0.00	95.61
26	−11	7	145.66	87.69
−2	−10	−10	−59.30	89.27
18	−9	6	−5.94	63.13
20	−7	6	−3.04	71.58
−5	−7	−7	152.66	78.18
−3	−6	−9	14.39	99.97
−4	−5	−8	117.93	81.22
−5	−3	−5	227.81	73.43
7	−2	3	481.23	61.14
−3	−1	−8	45.03	65.24
23	0	5	51.37	102.35
19	3	4	53.62	75.54
4	3	1	2836.80	239.56
23	4	4	−6.60	98.52
13	4	3	70.39	82.01
28	7	3	−0.26	95.08

8	7	1	15.05	57.71
2	9	−7	63.13	22.05
25	13	0	0.00	92.97
12	14	−2	184.36	71.58
11	15	−4	40.15	61.54
22	16	−3	105.91	87.82
16	−47	3	116.61	96.14
22	−33	7	−86.50	22.32
28	−31	8	−76.60	64.97
0	−31	−2	−45.16	96.54
4	−28	2	126.78	73.29
1	−24	1	−18.09	67.88
−3	−24	−3	19.55	86.37
7	−21	4	243.26	43.58
20	−20	7	−7.66	61.80
17	−10	6	159.79	64.71
−5	−10	−7	83.46	75.80
31	−9	7	−25.22	90.73
21	−6	6	−23.90	77.12
−3	−5	−1	33.02	44.50
26	−4	6	42.39	96.67
−2	−2	−9	20.87	101.03
22	7	3	54.67	92.18
19	13	0	−23.77	90.86
16	14	−1	−28.53	75.27
16	15	−2	−12.55	77.52
19	16	−3	−36.18	97.46
15	16	−4	70.39	98.39
7	−41	0	−14.79	96.14
1	−33	−2	3.43	93.50
2	−31	0	60.75	102.88
−2	−28	−4	40.15	98.78
28	−22	8	−26.94	74.09
−2	−22	−1	152.40	75.94
0	−19	1	335.30	73.43
33	−17	8	0.00	91.78
−3	−17	−1	−10.04	78.71
21	−16	7	2.51	68.41
−5	−16	−4	−67.62	79.76
−3	−15	−9	22.45	90.33
27	−10	7	41.20	83.07
−2	−8	0	39.09	40.67
−5	−8	−3	300.97	83.33
−4	−5	−2	1497.31	135.23
12	−1	4	143.95	74.75
−4	0	−4	1264.22	119.65
27	1	5	0.00	99.44
6	10	−1	17.30	53.35
7	12	−2	199.15	69.73
12	13	−1	−37.37	67.62
12	−44	2	−124.40	93.90
2	−35	−2	30.90	87.03
3	−33	0	59.16	89.67
1	−31	−1	12.02	100.10

20	−26	7	−7.00	45.96
−3	−25	−5	0.00	104.46
−4	−21	−4	27.73	80.29
−4	−14	−8	48.33	107.76
−5	−11	−3	91.39	76.99
−3	−9	−9	22.05	98.25
−4	−8	−8	72.90	87.95
22	−5	6	19.15	88.22
−5	−5	−6	18.62	67.75
30	−3	6	6.21	97.33
11	−2	4	712.07	93.63
19	−1	5	13.47	74.75
13	0	4	−37.24	75.94
−3	1	−7	−54.54	61.94
0	5	−8	−36.05	53.48
12	7	2	138.27	84.65
17	9	2	75.14	78.44
12	15	−3	166.53	69.60
6	−39	0	−118.99	96.40
5	−37	0	−39.35	86.50
4	−35	0	−34.73	84.65
2	−33	−1	107.37	92.57
27	−26	8	65.37	68.28
−3	−24	−6	26.54	100.50
−4	−19	−3	198.88	79.24
−4	−18	−7	127.17	120.97
34	−16	8	16.77	93.90
−2	−14	0	149.36	65.50
−3	−12	−9	26.81	94.82
−4	−11	−8	−108.29	109.35
28	−9	7	−11.36	83.99
12	−8	5	182.38	61.94
24	1	5	−27.20	106.04
14	1	4	0.00	70.12
14	5	3	203.64	83.99
9	8	1	50.58	65.77
27	10	2	−31.56	90.20
24	10	2	0.00	101.95
25	12	1	−20.21	94.16
22	12	1	13.34	109.61
7	13	−3	209.32	58.37
10	15	−5	−4.36	59.96
16	16	−3	−24.83	95.88
4	−37	−1	−3.04	91.52
3	−35	−1	34.47	86.90
−2	−26	−3	64.18	104.59
−3	−19	−8	64.97	86.37
−5	−15	−5	106.57	80.43
16	−11	6	109.08	59.82
32	−8	7	13.07	89.54
27	−3	6	−34.34	94.16
−4	−3	−7	188.85	69.60
−3	−1	−2	153.06	41.86
20	4	4	20.73	77.78

27	5	4	0.00	97.20
1	5	−1	964.31	86.50
19	7	3	115.55	79.24
21	10	2	−2.51	96.80
16	13	0	0.00	72.77
6	13	−4	206.94	50.84
15	−45	3	27.86	86.37
11	−42	2	13.47	90.07
5	−30	2	45.43	89.93
6	−27	3	17.04	60.88
−2	−27	−5	−86.10	94.42
−2	−24	−2	−34.60	79.63
27	−23	8	6.74	68.67
−4	−20	−5	9.77	91.91
−4	−19	−6	33.41	105.91
−4	−14	−2	119.12	84.25
−5	−13	−4	−16.24	76.07
−2	−11	0	57.45	49.92
−5	−11	−6	111.06	79.76
−5	−8	−6	17.56	72.63
23	−4	6	248.01	108.55
10	−3	4	1525.70	145.93
−4	−1	−6	119.91	60.09
20	0	5	8.45	75.41
24	5	4	0.00	106.18
26	8	3	−30.37	101.95
15	11	1	10.96	74.75
23	15	−1	55.33	88.08
20	15	−1	19.02	103.27
27	−29	8	46.62	62.99
2	−26	1	14.26	66.43
0	−24	0	53.35	70.92
29	−18	8	−24.43	76.33
30	−17	8	−55.07	85.84
20	−17	7	23.90	64.18
−3	−14	−1	866.19	105.38
29	−8	7	−48.60	89.93
−5	−7	−4	173.92	81.88
−5	−6	−5	12.94	73.29
4	−1	2	148.96	39.22
15	2	4	0.00	70.12
23	8	3	135.89	111.72
13	8	2	−31.30	75.80
7	11	−1	632.84	87.03
12	12	0	27.73	68.67
26	15	−1	−10.30	89.67
23	16	−2	−69.07	86.37
20	16	−2	−32.75	102.48
18	−41	5	−37.90	66.56
10	−40	2	−21.26	89.80
6	−32	2	−36.45	96.67
−1	−28	−3	15.45	109.35
−3	−23	−4	0.00	89.80
28	−19	8	38.69	72.77

4	−17	3	86.24	48.73
31	−16	8	−5.55	89.14
−4	−15	−7	4.89	105.25
−5	−12	−5	141.83	81.09
−5	−10	−4	46.88	92.97
−4	−8	−2	172.74	58.64
28	2	5	0.00	88.74
3	2	1	1251.81	116.35
−2	4	−6	110.54	51.37
15	6	3	86.10	75.01
10	9	1	686.59	100.23
3	11	−5	32.62	18.62
23	14	0	3.04	99.57
13	15	−2	5.68	67.75
26	16	−2	5.68	99.05
17	−44	4	25.09	79.24
−3	−19	−2	203.11	77.78
32	−15	8	8.32	92.31
−1	−12	−10	64.18	87.16
23	−11	7	58.90	92.44
−4	−11	−2	163.49	67.88
−1	−9	−10	−83.46	88.35
−2	−5	−9	−23.90	102.48
−3	−4	−8	256.99	76.60
24	−3	6	30.64	104.59
6	−3	3	762.39	76.99
28	−2	6	63.79	95.08
−2	0	−8	−3.57	62.99
25	2	5	0.00	104.20
16	3	4	170.09	74.35
18	10	2	93.50	75.14
8	13	−2	36.58	65.11
26	14	0	35.39	86.76
20	14	0	62.07	109.48
13	14	−1	105.52	76.73
11	−44	1	−22.85	94.82
14	−43	3	0.13	80.82
8	−36	2	33.94	82.01
7	−34	2	37.51	84.12
13	−31	5	10.96	50.58
9	−28	4	29.98	62.60
−1	−26	−2	38.03	86.90
3	−23	2	50.84	60.75
−3	−21	−3	100.89	77.92
27	−20	8	24.83	70.92
−1	−19	0	−13.07	70.39
2	−18	2	139.06	64.45
−4	−18	−4	14.92	81.88
10	−16	5	12.94	29.32
−3	−16	−8	127.31	107.89
−4	−16	−3	63.52	73.56
22	−12	7	−14.53	75.14
24	−10	7	41.34	92.31
−3	−8	−1	202.98	50.05

-1	-1	-9	10.96	93.10
21	1	5	-93.24	85.71
21	5	4	63.65	87.56
20	8	3	50.58	78.97
17	15	-1	35.26	87.16
17	16	-2	14.53	97.86
0	-30	-3	53.75	98.78
-3	-22	-5	-75.80	111.06
0	-16	1	382.71	73.56
33	-14	8	-5.28	94.03
15	-12	6	75.01	58.37
-4	-12	-7	-29.32	79.50
-3	-11	-1	1366.44	130.34
25	-9	7	0.00	89.54
30	-7	7	0.40	87.42
0	-7	1	948.20	85.05
-4	-6	-7	21.39	72.11
-3	2	-5	54.94	57.31
25	11	2	-13.60	103.80
8	14	-3	29.98	56.79
12	16	-4	-57.71	69.86
20	17	-3	-19.55	86.90
17	-39	5	-12.81	64.31
7	-29	3	78.31	75.41
3	-28	1	83.07	75.41
1	-26	0	-10.83	70.92
-2	-25	-4	-1.98	109.08
26	-24	8	-43.84	66.03
-3	-21	-6	34.47	111.72
-4	-17	-5	233.35	81.09
-4	-16	-6	10.56	79.90
-2	-14	-9	22.58	89.67
21	-13	7	-21.66	71.97
-4	-9	-7	-0.26	71.05
26	-8	7	60.62	84.78
9	-4	4	389.45	68.28
25	6	4	0.00	101.29
16	7	3	220.28	83.86
14	9	2	119.65	75.01
11	10	1	234.94	80.43
22	11	2	137.21	120.31
8	12	-1	272.71	75.01
26	13	1	-29.32	88.48
23	13	1	143.81	106.18
13	16	-3	0.00	67.75
15	-47	2	-39.35	93.24
16	-42	4	50.84	76.86
10	-42	1	-59.16	94.42
13	-41	3	-21.66	83.46
0	-26	-1	59.43	75.67
1	-21	1	-15.45	60.48
14	-19	6	37.24	38.83
-3	-13	-8	-41.34	109.74
-2	-11	-9	-93.24	97.20

11	−9	5	−1.58	56.39
−2	−8	−9	118.19	108.29
−4	−4	−3	357.36	61.80
25	−2	6	103.54	95.48
22	2	5	14.13	94.16
−2	2	−7	384.43	70.78
17	4	4	160.32	78.97
24	9	3	10.83	104.20
13	13	0	70.26	72.24
17	14	0	66.82	76.99
7	14	−4	354.19	63.65
14	−33	5	−6.60	53.22
26	−27	8	−69.86	62.73
5	−22	3	4.49	49.65
−2	−22	−7	0.00	97.20
26	−21	8	8.85	69.33
−3	−10	−8	1.06	93.90
−3	−7	−8	145.27	83.20
31	−6	7	−48.86	92.31
18	−6	6	−34.47	67.09
19	−5	6	−0.40	69.46
−4	−2	−5	100.23	70.26
29	−1	6	14.00	96.14
26	3	5	44.24	93.76
−1	6	−4	644.59	60.88
5	8	0	598.24	71.45
4	12	−5	118.46	36.32
20	13	1	−65.77	107.63
17	17	−3	0.00	102.74
16	−37	5	20.07	60.09
4	−30	1	134.83	90.46
−1	−27	−4	140.12	113.57
−2	−24	−5	−26.15	116.48
−3	−17	−7	−77.39	110.80
28	−16	8	−14.79	76.99
14	−16	6	17.96	46.88
20	−14	7	−13.60	68.67
−4	−13	−3	136.15	84.65
27	−7	7	−42.52	85.31
20	−4	6	54.94	78.18
−4	−4	−6	46.22	64.18
16	−1	5	75.01	71.18
22	6	4	0.00	101.95
4	7	0	1072.86	100.37
6	9	0	200.60	58.50
19	11	2	21.26	80.03
9	14	−2	171.02	64.31
24	16	−1	12.68	87.03
21	16	−1	110.40	97.33
9	−42	0	−80.69	98.12
12	−39	3	−40.54	83.46
15	−35	5	7.66	54.28
8	−31	3	−4.49	84.52
1	−30	−2	−31.30	111.46

2	−28	0	0.00	79.50
1	−28	−1	44.37	86.37
−2	−23	−6	24.04	107.63
−2	−23	−3	44.11	78.58
−2	−21	−2	45.03	78.84
−3	−20	−4	11.36	73.03
19	−18	7	32.75	60.22
−2	−18	−8	39.49	100.76
27	−17	8	11.75	72.90
−4	−15	−4	20.07	75.41
17	−7	6	190.43	68.01
−3	−2	−7	110.67	67.75
26	−1	6	−83.86	101.82
17	0	5	306.78	82.67
18	5	4	133.91	80.03
21	9	3	66.95	108.03
12	11	1	321.04	75.67
24	15	0	0.26	86.63
14	15	−1	43.71	70.65
14	16	−2	83.20	74.61
24	17	−2	−81.75	91.91
21	17	−2	−45.83	93.37
15	−40	4	6.47	73.82
8	−38	1	0.53	83.33
5	−32	1	−27.86	101.42
2	−32	−2	9.24	92.18
10	−30	4	−3.30	79.76
0	−29	−4	20.73	98.65
0	−14	−10	80.43	96.27
0	−13	1	245.63	52.43
28	−6	7	−60.22	84.39
13	−4	5	10.56	62.20
21	−3	6	238.24	90.99
17	8	3	0.00	76.60
15	10	2	74.75	76.46
7	10	0	1093.73	116.21
9	13	−1	−13.73	67.09
21	15	0	−43.45	110.14
14	−45	2	−93.90	94.42
11	−37	3	−0.13	76.46
7	−36	1	91.65	84.65
4	−36	−2	−35.66	87.56
6	−34	1	70.52	90.99
3	−34	−2	45.56	88.74
9	−33	3	−39.49	83.07
−1	−26	−5	−2.38	103.14
4	−25	2	71.18	62.86
4	−14	3	745.88	71.97
−4	−14	−5	155.57	78.05
10	−13	5	67.62	38.56
−4	−10	−6	201.92	77.26
16	−8	6	106.44	64.58
0	−8	−10	−58.64	89.01
−4	−7	−6	95.48	71.58

-4	-7	-3	1257.75	126.38
-1	-4	-9	-25.88	99.71
-3	0	-6	559.41	76.86
-3	0	-3	1143.38	105.12
18	1	5	37.64	77.78
23	3	5	3.04	109.61
26	7	4	-20.07	96.67
9	15	-3	54.94	62.73
18	16	-1	23.77	106.97
8	-40	0	26.41	93.10
27	-32	8	16.51	55.33
3	-30	0	-23.77	90.73
2	-30	-1	-3.04	99.31
-1	-25	-6	38.30	99.71
19	-24	7	2.25	45.16
37	-23	9	31.56	94.29
-1	-21	-1	41.47	69.73
-3	-19	-5	140.64	87.16
26	-18	8	-3.96	72.37
-3	-18	-6	-35.79	90.07
-3	-18	-3	232.30	78.18
-1	-16	-9	128.36	98.78
-1	-16	0	-27.86	69.20
32	-12	8	0.00	89.67
0	-11	-10	-3.57	86.24
-4	-10	-3	298.06	78.84
-1	-4	0	683.55	66.82
-2	-3	-8	-9.38	69.86
22	-2	6	64.45	105.78
-1	1	-8	34.07	59.96
-3	1	-4	84.25	45.96
27	4	5	-7.92	86.24
3	6	0	452.71	58.50
23	12	2	24.30	101.95
18	17	-2	90.86	103.93
14	-38	4	-84.25	72.77
7	-38	0	-54.54	88.35
6	-38	-1	17.04	89.67
4	-32	0	-8.19	103.40
3	-32	-1	-38.69	99.44
-1	-25	-3	61.80	85.31
36	-24	9	5.81	86.10
25	-22	8	3.17	64.97
-2	-16	-1	85.58	76.86
-3	-14	-7	56.65	89.93
-4	-12	-4	163.23	79.90
0	-10	1	171.94	39.22
29	-5	7	47.94	87.42
27	0	6	23.64	87.29
9	3	3	482.15	79.50
10	4	3	727.66	100.23
11	5	3	104.33	78.71
19	6	4	14.53	73.69
23	7	4	-67.48	111.72

25	10	3	76.99	100.76
24	14	1	105.25	93.10
14	14	0	92.71	76.46
18	15	0	-60.88	100.63
14	17	-3	75.80	85.71
13	17	-4	52.69	94.95
13	-36	4	3.70	69.73
6	-36	0	-78.31	90.33
5	-36	-1	-38.43	87.29
5	-34	0	-58.11	92.18
4	-34	-1	0.00	91.25
0	-28	-5	-19.68	97.59
25	-25	8	-18.49	60.88
11	-24	5	20.73	29.19
-2	-22	-4	-22.32	80.95
-1	-20	-8	17.83	95.22
19	-15	7	23.90	64.45
-2	-15	-8	54.81	117.14
-4	-11	-5	51.64	73.69
22	-9	7	93.90	97.33
7	-9	4	57.97	39.49
23	-8	7	-3.17	93.10
8	-5	4	101.03	48.60
-4	-5	-5	284.72	85.58
23	-1	6	38.03	104.46
19	2	5	90.86	76.73
-1	5	-6	53.75	44.37
8	6	2	243.52	65.63
18	9	3	0.00	74.48
20	12	2	17.30	102.08
13	12	1	-27.47	70.65
5	13	-5	15.98	41.07
21	14	1	24.30	109.48
10	15	-2	52.56	66.29
8	15	-4	82.54	56.26
17	-46	3	63.79	88.22
13	-43	2	0.00	90.20
12	-34	4	-36.18	66.43
2	-23	1	83.07	64.84
-1	-23	-2	-19.41	78.31
14	-13	6	19.02	51.24
-1	-13	-9	17.56	94.56
21	-10	7	117.67	75.67
24	-7	7	0.53	86.50
-1	-7	-9	-25.22	107.89
-4	-6	-4	265.05	80.56
12	-5	5	96.01	63.65
-3	-5	-7	9.90	68.14
24	4	5	36.32	112.52
7	5	2	239.29	57.71
12	6	3	10.70	78.71
9	7	2	581.46	90.86
22	10	3	6.60	112.78
16	11	2	29.19	77.12

10	14	−1	367.66	75.54
26	−30	8	−4.36	55.20
0	−27	−3	21.79	102.88
25	−19	8	0.00	63.79
−3	−13	−2	126.65	77.52
−3	−11	−7	205.62	78.84
−1	−10	−9	53.48	102.61
15	−9	6	162.83	63.79
−4	−9	−4	110.01	90.59
25	−6	7	−28.66	88.74
−3	−4	−2	2239.75	192.41
8	2	3	89.67	60.22
10	8	2	8.58	73.69
15	16	−1	88.88	75.67
22	17	−1	−31.30	99.05
18	18	−3	−1.85	92.44
13	−45	1	70.12	96.54
5	−27	2	−36.18	64.84
36	−21	9	85.44	97.20
−2	−21	−5	8.32	88.74
−2	−20	−6	46.75	119.91
−3	−17	−4	94.03	83.20
27	−14	8	57.18	79.24
28	−13	8	−57.71	88.08
29	−12	8	−21.39	90.07
−2	−12	−8	−72.24	106.44
−3	−8	−7	91.39	73.95
−2	−6	−8	33.68	77.78
30	−4	7	−15.19	89.01
12	2	4	37.64	83.59
20	3	5	95.22	88.61
13	3	4	225.56	80.82
−1	3	−7	19.28	55.60
20	7	4	35.66	88.35
13	7	3	129.42	78.44
22	16	0	75.27	90.59
25	17	−1	57.58	97.20
15	17	−2	3.17	85.05
12	−41	2	−17.70	88.61
1	−29	−3	−21.66	117.01
8	−23	4	14.26	42.52
0	−23	−1	114.89	76.60
35	−22	9	33.41	82.14
3	−20	2	135.23	57.97
−2	−20	−3	120.84	77.12
20	−11	7	−13.73	73.82
−2	−9	−8	31.30	86.10
26	−5	7	−22.98	89.93
24	0	6	50.84	98.52
28	1	6	−46.75	88.48
11	1	4	167.98	78.97
14	4	4	264.91	83.73
6	4	2	1077.75	105.52
24	8	4	11.23	103.93

18	14	1	−0.92	89.01
22	18	−2	34.47	94.82
2	−31	−3	−7.79	94.16
0	−25	−2	−37.64	78.97
6	−24	3	16.11	54.15
−1	−24	−4	−52.96	89.54
34	−23	9	17.70	82.01
7	−18	4	94.16	29.45
−2	−18	−2	73.69	70.39
−3	−16	−5	51.77	77.52
−3	−15	−3	26.41	81.61
30	−11	8	5.81	90.46
11	9	2	2.51	84.12
19	10	3	130.74	92.84
15	15	0	−44.24	72.90
10	16	−3	14.92	62.33
19	17	−1	147.25	107.37
17	−48	2	99.71	98.39
19	−45	4	−42.79	80.82
16	−44	3	0.00	84.65
21	−39	6	−45.96	54.67
−1	−17	−8	−25.09	101.16
−2	−16	−7	−34.34	103.54
1	−13	−10	29.05	91.39
−1	−13	0	169.57	57.71
−2	−13	−1	347.72	75.67
31	−10	8	25.62	92.18
−3	−10	−2	255.93	69.86
5	−4	3	371.09	47.01
10	0	4	2104.13	194.39
−2	3	−5	80.29	54.94
21	4	5	68.54	110.93
25	5	5	16.24	92.97
15	5	4	209.98	76.07
14	8	3	37.64	74.75
17	12	2	65.90	76.20
10	13	0	55.99	71.45
6	14	−5	24.96	45.30
19	16	0	−52.82	106.44
19	18	−2	16.77	91.52
12	−43	1	−21.26	94.03
11	−39	2	2.11	84.39
6	−29	2	9.51	69.46
3	−25	1	−25.88	68.14
33	−24	9	−24.70	78.84
−1	−23	−5	21.79	104.86
−1	−22	−6	60.48	107.89
10	−10	5	96.67	51.77
1	−10	−10	56.92	84.52
−3	−7	−2	3329.26	282.48
11	−6	5	−6.34	66.29
27	−4	7	−44.50	91.39
−2	−4	−1	708.24	78.31
0	−3	−9	163.89	100.50

−3	−3	−6	142.23	65.11
−2	−1	−7	1.32	64.84
25	1	6	4.62	95.48
7	1	3	92.18	55.20
2	1	1	502.10	52.56
23	11	3	0.00	101.16
21	13	2	−12.55	105.52
22	15	1	38.96	99.84
11	15	−1	63.79	69.99
11	16	−2	63.39	69.46
9	16	−4	3.96	58.64
25	−28	8	0.00	55.20
1	−27	−2	−26.02	87.16
0	−26	−4	−40.94	107.63
24	−20	8	−10.43	66.69
18	−19	7	0.00	56.39
0	−15	−9	−23.51	90.33
19	−12	7	46.22	67.62
2	−12	2	304.66	43.32
−3	−12	−6	157.28	83.20
18	−3	6	97.86	76.86
19	−2	6	33.54	74.75
−3	−1	−5	362.38	75.41
0	2	−8	−21.53	60.75
−1	5	−3	249.46	28.79
16	6	4	134.04	78.71
21	8	4	92.57	112.91
12	10	2	22.45	73.43
15	18	−3	−40.28	101.03
10	−37	2	26.81	82.14
7	−31	2	22.58	95.22
24	−23	8	0.00	60.48
0	−23	−7	24.43	96.80
14	−22	6	15.58	28.26
34	−20	9	108.16	88.22
35	−19	9	24.83	95.08
−1	−18	−1	56.26	79.76
18	−16	7	−21.26	61.14
17	−4	6	101.95	70.78
28	−3	7	0.92	93.63
−1	−2	−8	−6.07	67.35
20	−1	6	−1.19	89.27
2	5	0	−26.81	43.84
15	9	3	21.66	79.37
9	−35	2	0.00	83.73
8	−33	2	89.80	94.29
1	−28	−4	170.62	120.57
2	−25	0	53.09	70.78
−1	−22	−3	86.10	79.10
33	−21	9	8.98	77.39
−2	−19	−4	37.11	75.80
0	−18	0	399.22	87.56
−2	−17	−6	12.81	79.10
−3	−14	−4	212.62	75.94

14	−10	6	294.63	65.90
2	−9	2	694.24	62.20
−3	−9	−6	238.24	76.20
16	−5	6	95.88	68.54
3	−2	2	378.62	46.75
22	5	5	−25.49	106.18
26	6	5	19.81	87.56
6	8	1	211.03	56.39
25	9	4	30.51	91.39
7	9	1	426.82	73.82
20	11	3	63.52	101.16
19	15	1	39.49	112.38
16	17	−1	−17.83	92.97
16	−46	2	0.00	93.37
15	−42	3	147.38	84.25
6	−37	−2	48.20	87.95
4	−27	1	3.43	64.84
32	−25	9	96.27	79.37
0	−25	−5	0.00	114.63
0	−24	−6	58.11	118.46
0	−19	−8	35.52	98.25
−2	−18	−5	27.20	72.63
24	−17	8	0.92	64.71
−1	−14	−8	152.27	110.27
−2	−13	−7	119.25	79.63
−3	−13	−5	−40.81	72.90
0	−12	−9	85.05	97.86
−3	−12	−3	46.22	80.29
−1	−10	0	466.18	59.43
−2	−10	−1	705.60	81.48
−3	−6	−6	18.49	66.69
9	−1	4	636.27	87.82
21	0	6	65.11	105.12
14	0	5	370.56	78.05
15	1	5	132.46	73.82
−2	1	−6	213.28	61.28
16	2	5	0.00	80.69
5	3	2	876.09	87.56
17	7	4	80.43	77.39
8	10	1	6.47	71.18
13	11	2	93.10	73.95
18	13	2	24.17	91.65
15	14	1	0.00	67.62
11	14	0	8.45	71.58
16	16	0	−26.54	90.46
23	17	0	−16.77	92.97
23	18	−1	−10.30	90.07
16	18	−2	−74.09	104.99
18	−43	4	33.81	78.05
10	−41	0	174.32	96.40
5	−35	−2	−35.52	85.97
4	−33	−2	0.00	99.97
3	−31	−2	105.65	114.76
2	−30	−4	−34.86	96.14

2	-27	-1	25.88	74.88
-1	-20	-2	-6.21	70.26
-1	-18	-7	4.75	111.86
26	-12	8	0.00	80.95
27	-11	8	-49.65	86.63
28	-10	8	1.45	87.56
21	-7	7	162.04	97.33
-1	-7	0	707.71	74.75
22	-6	7	176.30	98.39
0	-6	-9	71.45	103.01
23	-5	7	-48.99	92.84
-3	-3	-3	708.24	79.24
-2	0	-2	97.99	38.83
9	11	1	296.08	86.24
12	16	-1	16.51	69.46
11	17	-3	71.45	66.82
20	18	-1	0.40	97.73
20	-42	5	30.77	66.82
10	-39	1	0.00	86.63
8	-39	-1	-56.92	91.52
20	-37	6	14.92	48.99
20	-29	7	12.41	28.39
1	-27	-5	2.77	107.23
7	-26	3	173.13	58.77
32	-22	9	33.28	78.71
5	-19	3	152.40	49.79
2	-15	-10	-16.24	89.54
-2	-15	-2	65.37	80.29
29	-9	8	35.00	88.22
0	-9	-9	72.90	108.55
-2	-7	-1	1047.90	101.55
24	-4	7	-28.92	90.20
-2	-4	-7	0.00	66.43
22	1	6	31.69	102.74
17	3	5	4.75	73.03
5	7	1	735.71	84.12
22	9	4	-0.40	108.03
16	10	3	103.14	77.39
24	12	3	-15.05	91.91
7	15	-5	102.48	50.58
20	17	0	-54.15	95.74
12	17	-2	169.04	71.71
5	-29	1	7.79	72.24
3	-27	0	-42.79	70.52
1	-26	-6	-6.60	99.44
0	-24	-3	87.82	75.27
1	-17	-9	28.00	88.48
-2	-17	-3	104.33	69.20
25	-13	8	-47.94	75.41
18	-13	7	-24.17	65.90
-1	-11	-8	38.30	96.14
-2	-10	-7	66.43	71.31
20	-8	7	0.00	90.07
15	-6	6	109.48	65.77

-1	-5	-8	30.11	70.39
18	4	5	67.22	77.12
23	6	5	22.58	101.95
23	16	1	-31.83	93.76
14	-40	3	53.09	83.73
9	-39	0	0.00	89.14
9	-37	1	61.67	83.20
7	-37	-1	8.19	85.71
3	-29	-1	68.41	84.12
2	-29	-5	-11.36	99.71
24	-26	8	-57.18	58.37
4	-22	2	6.74	54.01
-1	-21	-4	46.35	77.39
33	-18	9	5.15	91.78
34	-17	9	-13.21	97.73
-3	-11	-4	194.13	94.03
-3	-10	-5	4.62	69.07
-3	-9	-3	245.77	73.95
30	-8	8	-24.96	93.63
-2	-7	-7	50.05	73.82
-3	-4	-5	902.24	106.18
25	-3	7	12.94	89.80
6	0	3	310.08	55.86
18	8	4	31.69	78.58
21	12	3	-12.02	106.31
14	12	2	152.00	80.56
10	12	1	98.12	81.22
12	15	0	18.75	73.43
10	17	-4	-13.60	63.13
20	19	-2	43.05	88.08
19	-47	3	36.32	90.73
15	-44	2	0.00	88.61
8	-37	0	36.71	85.58
8	-35	1	-14.39	90.33
6	-35	-1	40.54	89.14
7	-33	1	24.70	98.12
5	-33	-1	-39.22	102.08
6	-31	1	-32.22	96.54
4	-31	-1	53.35	105.65
4	-29	0	-38.17	79.24
31	-23	9	-40.28	75.94
1	-21	-8	-53.48	87.42
2	-20	1	120.18	65.37
32	-19	9	-49.26	80.29
-1	-19	-6	20.60	91.65
2	-12	-10	-12.55	87.42
-1	-8	-8	5.41	79.50
-3	-6	-3	192.94	58.11
0	4	-7	141.31	53.88
0	6	-6	41.47	38.03
16	15	1	76.60	84.65
20	16	1	-9.77	100.10
16	19	-3	-23.64	96.54
31	-26	9	-18.75	74.48

1	−26	−3	1.85	94.16
0	−22	−2	166.79	76.20
0	−20	−1	64.45	67.09
−1	−20	−5	74.88	81.88
1	−15	1	201.39	59.69
24	−14	8	58.37	76.73
19	−9	7	6.34	72.50
2	−9	−10	−45.83	86.24
26	−2	7	−52.56	88.88
1	−2	−9	0.00	98.12
19	5	5	46.35	84.92
17	11	3	0.79	72.24
19	14	2	164.15	108.29
17	18	−1	151.08	115.29
17	−41	4	−22.45	75.54
13	−38	3	0.00	78.71
19	−35	6	−26.02	45.43
7	−35	0	3.83	89.01
6	−33	0	17.43	99.44
17	−31	6	−19.28	35.66
5	−31	0	46.88	104.20
8	−28	3	15.19	62.46
9	−25	4	62.33	47.15
23	−21	8	44.50	63.52
31	−20	9	−56.39	79.24
23	−18	8	21.92	69.60
0	−16	−8	29.32	109.08
−2	−16	−4	113.31	76.20
13	−14	6	52.16	48.47
−3	−8	−4	88.22	74.61
10	−7	5	374.66	70.39
−3	−5	−4	78.84	64.31
4	6	1	1631.09	144.87
24	7	5	0.00	96.54
19	9	4	−10.17	90.20
23	10	4	−6.34	97.06
4	12	−2	359.07	64.84
11	13	1	116.21	72.90
5	13	−2	207.20	65.11
17	17	0	−23.51	111.59
13	17	−1	−0.66	69.46
2	−28	−3	−152.53	110.01
0	−23	−4	44.64	84.78
1	−20	0	93.76	71.71
−1	−15	−7	−24.56	89.27
−1	−15	−1	154.12	70.12
−2	−15	−5	−1.98	83.07
1	−14	−9	−10.17	96.40
8	−2	4	315.49	65.11
−1	0	−7	37.37	59.30
−2	2	−4	482.02	58.77
1	3	−8	76.73	59.03
15	13	2	−50.71	76.07
23	15	2	−7.40	89.67

12	18	−3	66.69	80.95
17	19	−2	88.48	99.97
19	−40	5	98.39	68.14
18	−33	6	−30.11	41.07
3	−30	−3	80.43	113.31
0	−21	−6	40.81	106.84
−1	−19	−3	42.00	73.16
14	−7	6	515.57	81.09
27	−1	7	−75.14	89.14
20	6	5	108.42	104.46
5	12	−1	374.13	72.90
6	14	−2	180.13	66.82
13	16	0	−35.13	72.37
8	16	−5	−2.77	53.88
21	18	0	0.00	92.84
13	18	−2	40.94	78.58
21	19	−1	84.39	89.93
14	−44	1	−43.98	96.01
14	−42	2	−63.39	87.29
5	−34	−3	34.47	97.59
9	−30	3	−3.83	79.10
1	−22	−7	0.00	100.37
0	−22	−5	66.69	89.41
13	−17	6	−15.98	38.30
3	−17	2	514.91	75.54
32	−16	9	−16.51	89.67
33	−15	9	−34.34	96.40
−2	−12	−2	83.33	68.54
13	−11	6	72.63	54.28
−2	−11	−6	145.80	77.78
18	−10	7	77.39	66.95
26	−9	8	7.26	81.22
27	−8	8	−52.96	90.59
−2	−2	−6	153.32	63.26
0	−1	−8	−10.43	65.77
4	2	2	132.59	46.49
10	7	3	397.77	91.25
11	8	3	52.82	78.97
3	11	−2	591.77	70.39
18	12	3	58.50	94.69
22	13	3	7.79	101.42
6	13	−1	97.06	68.14
17	16	1	0.00	97.99
18	−45	3	−12.81	87.42
11	−34	3	0.00	80.43
10	−32	3	−7.00	90.73
12	−26	5	0.00	45.83
1	−25	−4	9.11	87.56
30	−24	9	74.22	74.88
1	−24	−2	−22.32	76.86
30	−21	9	36.32	74.75
0	−15	0	136.02	65.63
−2	−14	−3	0.00	85.97
1	−11	−9	10.43	103.27

25	−10	8	−32.35	83.99
28	−7	8	−65.50	91.91
1	−5	−9	39.09	103.93
17	−1	6	−49.26	69.86
18	0	6	110.67	80.69
19	1	6	0.00	90.59
25	4	6	83.59	98.65
12	5	4	0.00	75.14
13	6	4	164.28	78.05
9	6	3	168.64	84.25
20	10	4	−7.79	114.23
4	11	−1	205.49	53.48
7	15	−2	13.87	58.24
11	18	−4	−7.92	74.22
16	−39	4	0.00	70.39
23	−24	8	−46.09	58.64
5	−24	2	−25.75	60.35
1	−22	−1	−21.26	71.97
1	−18	−8	15.19	100.10
31	−17	9	80.56	85.44
17	−17	7	40.81	56.13
−1	−17	−2	155.83	83.46
3	−14	−10	93.90	94.16
0	−13	−8	32.49	123.21
24	−11	8	−71.05	81.48
1	−8	−9	59.43	108.16
21	−4	7	103.93	105.25
22	−3	7	−11.75	95.08
11	4	4	110.14	87.03
21	7	5	−41.86	120.18
14	7	4	220.41	83.07
24	11	4	−36.45	90.33
4	13	−3	123.74	58.77
16	14	2	114.63	80.82
7	14	−1	623.20	94.82
21	17	1	24.43	89.80
21	20	−2	1.85	91.65
2	−24	−7	11.49	107.63
1	−23	−6	15.45	119.78
3	−22	1	43.58	61.80
−1	−16	−6	−80.16	76.20
17	−14	7	20.73	62.60
−1	−12	−7	45.56	81.48
−2	−8	−6	−29.05	71.05
29	−6	8	108.42	91.52
20	−5	7	−73.95	100.63
23	−2	7	2.77	91.39
8	5	3	335.57	74.88
13	10	3	29.71	72.63
5	14	−3	187.66	57.97
18	18	0	0.00	103.14
14	18	−1	102.08	91.25
18	19	−1	0.00	94.95
18	−47	2	56.52	94.03

13	−42	1	−14.26	89.54
13	−40	2	103.27	90.73
18	−38	5	−29.45	64.31
2	−27	−4	17.56	108.29
2	−26	−2	0.00	79.37
1	−24	−5	−16.90	103.93
30	−18	9	72.63	80.69
−1	−18	−4	−43.58	78.71
0	−17	−7	−0.40	102.22
2	−16	−9	106.04	96.54
−2	−12	−5	75.80	71.97
−2	−5	−6	162.70	66.56
−2	0	−5	277.59	67.62
10	3	4	238.77	84.39
15	8	4	123.34	77.92
19	13	3	−55.07	108.03
13	15	1	97.06	78.71
8	15	−1	156.76	66.69
6	15	−3	41.86	53.62
14	17	0	−9.11	79.24
12	−42	0	55.20	94.56
3	−29	−4	−8.98	121.50
10	−27	4	0.00	53.88
0	−21	−3	236.92	81.75
−1	−17	−5	166.40	83.73
−2	−13	−4	47.41	75.54
23	−12	8	46.75	76.60
3	−11	−10	−26.68	87.69
0	−10	−8	256.20	94.42
19	−6	7	−0.13	93.10
0	−4	−8	256.99	75.67
15	−3	6	10.04	70.92
−1	−3	−7	34.20	65.11
24	−1	7	−65.50	95.74
8	9	2	107.63	75.14
9	10	2	232.03	87.16
21	11	4	−0.40	105.25
3	12	−3	192.02	53.22
23	14	3	5.55	92.05
8	16	−2	75.41	65.11
14	19	−2	−13.47	101.29
13	19	−3	45.56	97.20
18	20	−2	−35.39	88.74
10	−40	−1	56.79	88.08
15	−37	4	31.43	65.50
7	−36	−2	7.40	88.48
4	−31	−4	3.70	99.05
3	−28	−2	36.58	87.95
2	−26	−5	−40.15	120.97
2	−25	−6	23.77	108.16
2	−24	−1	106.04	80.43
29	−22	9	−19.41	73.16
2	−20	−8	43.71	100.89
22	−19	8	22.98	66.16

−1	−9	−7	4.09	71.71
−2	−9	−2	817.59	92.05
−1	2	−6	228.73	60.35
14	3	5	98.78	81.88
15	4	5	140.78	82.41
−1	4	−5	305.99	55.99
22	8	5	99.71	102.08
7	8	2	74.48	66.43
16	9	4	47.67	79.24
14	11	3	69.73	78.31
10	11	2	174.32	81.48
18	17	1	79.63	115.95
22	19	0	−12.02	90.46
17	−43	3	−54.81	84.39
12	−38	2	8.72	81.88
6	−34	−2	−52.30	97.99
5	−32	−2	−66.69	114.10
6	−26	2	13.73	63.79
29	−19	9	69.33	76.33
31	−14	9	85.31	90.73
32	−13	9	−42.39	94.95
17	−11	7	39.09	63.92
13	−8	6	−36.98	60.62
0	−7	−8	181.72	78.31
−1	−6	−7	78.31	71.97
2	−1	−9	2.77	94.29
25	0	7	54.81	89.93
−1	0	−1	804.65	69.20
22	4	6	50.32	88.35
16	5	5	43.58	72.90
3	5	1	90.73	46.49
2	10	−2	251.18	43.84
21	16	2	0.00	104.06
9	16	−1	58.37	68.67
7	16	−3	107.76	61.14
22	20	−1	60.35	92.57
12	−40	1	85.97	94.03
3	−28	−5	−81.22	103.40
3	−27	−6	−37.77	100.10
6	−21	3	43.05	47.54
0	−18	−6	24.30	81.48
4	−16	−10	19.02	92.84
30	−15	9	−59.16	89.41
1	−15	−8	−27.86	118.85
−1	−12	−1	451.91	70.65
−2	−11	−3	30.64	70.52
18	−7	7	112.78	73.16
−2	−3	−2	353.40	54.15
5	−1	3	304.93	49.52
13	2	5	47.41	73.56
9	2	4	741.39	99.05
7	4	3	1218.00	122.68
17	6	5	−7.00	85.18
5	11	0	415.99	77.92

8	14	0	201.00	76.33
22	18	1	−61.14	89.54
21	−48	3	7.92	94.95
11	−40	0	−30.37	93.90
9	−38	−1	−41.86	89.01
17	−36	5	3.83	58.64
11	−36	2	26.68	82.01
14	−35	4	−10.43	68.01
11	−29	4	7.79	72.77
4	−24	1	−28.13	65.90
1	−23	−3	101.95	86.37
0	−20	−4	102.35	78.71
1	−19	−7	−53.48	114.36
0	−19	−2	115.29	69.86
3	−18	−9	96.93	90.73
0	−17	−1	231.50	82.27
2	−13	−9	152.00	101.55
1	−12	1	86.76	41.20
14	−4	6	191.09	72.37
10	−4	5	149.10	71.71
26	1	7	27.73	90.73
23	5	6	33.28	94.82
23	9	5	−1.85	88.74
17	10	4	−89.14	88.61
15	12	3	131.27	76.73
20	14	3	57.18	105.78
14	16	1	34.86	75.41
9	17	−2	65.11	66.16
15	19	−1	67.35	112.52
17	−45	2	−58.90	90.46
20	−44	4	−63.65	82.80
7	−28	2	54.28	65.63
19	−27	7	−7.40	28.53
29	−25	9	−17.83	72.24
17	−20	7	76.33	51.24
0	−19	−5	223.05	82.27
29	−16	9	51.77	81.22
−1	−16	−3	−75.01	79.10
0	−14	−7	345.87	91.12
22	−13	8	29.98	75.54
24	−8	8	−30.64	86.50
25	−7	8	−6.74	83.46
26	−6	8	−33.41	86.50
−2	−6	−2	1396.68	127.31
27	−5	8	−2.51	92.05
12	1	5	75.27	67.35
2	4	−8	38.69	55.60
1	5	−7	52.82	48.33
18	7	5	−8.06	100.89
6	7	2	64.45	55.99
22	12	4	23.24	100.23
12	13	2	−36.18	75.94
9	15	0	43.18	68.54
15	18	0	−22.58	95.35

19	19	0	60.48	99.71
19	20	−1	21.92	90.07
11	−38	1	10.70	84.25
10	−38	0	4.23	87.16
8	−36	−1	62.99	90.46
10	−34	2	−1.19	89.27
12	−31	4	74.48	80.03
13	−28	5	−11.62	60.09
22	−22	8	102.22	61.28
−2	−10	−4	−17.30	83.73
7	−3	4	1251.81	119.38
−2	−3	−5	564.69	86.24
1	4	0	922.18	84.12
4	10	0	1212.45	117.53
10	17	−1	57.58	74.75
8	17	−3	16.11	64.31
7	−34	−1	−2.77	96.40
9	−32	2	3.96	96.93
8	−30	2	20.07	73.82
4	−28	−1	−9.38	74.88
2	−25	−3	45.30	82.67
28	−20	9	−12.28	73.69
1	−17	0	−41.34	68.01
−1	−14	−2	112.12	78.31
4	−13	−10	−0.66	88.48
0	−12	0	546.20	68.94
2	−10	−9	130.21	108.16
23	−9	8	74.61	83.73
−2	−6	−5	205.49	82.01
28	−4	8	−2.77	90.73
2	−4	−9	22.05	101.42
1	0	−8	7.00	66.43
24	6	6	−21.53	91.78
19	8	5	73.16	106.44
18	11	4	−59.43	104.06
16	13	3	−12.41	85.97
13	14	2	−25.22	78.44
19	18	1	0.00	96.14
15	20	−2	38.17	105.52
14	20	−3	−81.48	104.46
16	−41	3	20.60	85.44
16	−34	5	23.77	55.99
6	−32	−1	83.73	103.40
5	−30	−1	−46.49	90.33
5	−26	1	91.65	70.39
4	−26	0	68.54	70.26
1	−22	−4	85.31	77.52
1	−20	−6	4.23	97.59
−1	−15	−4	51.11	70.78
−1	−14	−5	42.92	82.54
9	−11	5	1.58	45.16
17	−8	7	8.98	68.94
2	−7	−9	102.61	105.65
−2	−1	−4	83.59	50.71

0	1	−7	47.54	59.96
2	11	−3	45.16	41.47
10	16	0	56.13	74.48
22	17	2	22.58	85.31
15	17	1	21.92	86.37
10	18	−2	28.53	67.48
19	21	−2	−36.85	88.74
16	−45	1	−31.30	96.93
10	−36	1	108.82	90.59
9	−36	0	73.82	93.10
7	−35	−3	57.58	97.99
3	−27	−3	19.15	93.90
28	−23	9	56.39	70.52
1	−21	−2	116.48	72.37
28	−17	9	38.69	77.65
2	−17	−8	−77.39	114.63
30	−12	9	61.41	94.69
1	−12	−8	90.59	104.86
31	−11	9	−85.71	92.57
−2	−8	−3	278.65	66.16
20	−2	7	57.05	110.80
21	−1	7	10.96	97.33
22	0	7	10.04	97.33
11	0	5	533.92	87.29
21	15	3	−32.22	100.37
22	−43	5	62.73	71.97
16	−43	2	−54.15	91.39
9	−34	1	−25.22	98.12
8	−34	0	−83.20	96.40
15	−32	5	−54.15	54.81
25	−31	8	−7.66	43.84
5	−31	−3	0.00	108.29
4	−29	−3	−0.26	109.21
6	−28	1	35.39	68.28
5	−28	0	−32.62	76.73
1	−21	−5	14.39	77.65
4	−19	2	176.04	64.45
3	−15	−9	0.00	96.01
29	−13	9	16.90	89.67
22	−10	8	72.50	76.99
4	−10	−10	6.21	88.35
−1	−10	−6	56.39	73.03
9	−8	5	105.12	57.05
19	−3	7	28.79	98.39
−2	−2	−3	694.38	73.56
16	1	6	42.79	70.78
8	1	4	553.47	81.09
6	3	3	47.01	50.84
5	6	2	468.55	68.41
20	9	5	−3.04	113.44
19	12	4	11.36	112.52
11	18	−1	24.56	69.33
9	18	−3	41.34	64.05
16	19	0	3.17	112.91

16	20	−1	−42.66	102.08
8	−32	1	60.48	100.89
7	−32	0	15.19	103.14
7	−30	1	70.92	78.97
6	−30	0	−74.88	80.56
3	−23	−7	−29.19	104.86
1	−19	−1	−17.43	80.29
21	−14	8	180.40	75.54
0	−11	−7	249.73	81.75
−2	−7	−4	14.79	67.48
13	−5	6	91.52	65.11
−1	−1	−6	230.58	65.24
23	1	7	−3.83	87.95
7	12	1	243.65	86.37
8	13	1	112.91	88.22
17	14	3	121.50	101.55
14	15	2	52.96	76.20
20	20	0	7.66	88.22
20	−48	2	24.43	95.61
20	−46	3	47.67	90.46
19	−42	4	−84.78	80.16
15	−39	3	4.09	83.46
24	−29	8	33.41	47.41
2	−24	−4	−34.07	80.16
2	−22	−6	−0.92	105.38
0	−18	−3	112.65	75.41
1	−16	−7	−15.45	89.41
16	−15	7	44.37	59.03
16	−12	7	0.00	60.88
1	−9	−8	−45.03	84.65
−1	−9	−1	65.77	44.90
−2	−5	−3	245.11	58.50
15	0	6	91.65	73.03
24	2	7	48.73	87.29
20	5	6	115.16	117.67
1	7	−6	10.56	34.07
3	9	0	654.23	74.61
6	11	1	500.51	86.90
9	14	1	13.87	64.58
19	17	2	29.05	96.80
11	17	0	90.86	78.31
20	21	−1	16.24	91.52
2	−23	−5	54.28	90.20
2	−23	−2	−32.49	72.63
3	−19	−8	65.11	102.22
5	−15	−10	−3.96	87.56
28	−14	9	8.58	87.82
18	−4	7	34.73	88.74
1	−3	−8	43.45	66.82
21	10	5	35.00	96.67
5	15	−4	94.16	49.39
16	18	1	−8.72	111.20
20	19	1	−3.96	95.88
11	19	−2	70.78	63.52

15	−43	1	−92.05	97.86
14	−43	0	−81.88	98.52
3	−26	−4	0.00	90.20
3	−24	−6	43.05	116.21
7	−23	3	111.33	52.96
27	−18	9	45.03	76.20
5	−16	3	92.05	43.45
12	−12	6	289.87	57.45
−1	−7	−6	16.51	66.82
1	−6	−8	162.30	76.60
24	−5	8	−17.56	85.31
25	−4	8	−72.37	88.35
3	1	2	117.53	45.16
25	3	7	25.09	91.39
21	6	6	35.92	106.84
11	7	4	263.73	77.65
12	8	4	0.00	72.37
13	9	4	35.79	85.31
20	13	4	−18.49	105.78
6	16	−4	122.42	55.73
16	21	−2	−59.82	95.08
15	−41	2	23.90	93.10
9	−37	−2	64.97	87.03
6	−32	−4	−73.29	101.29
5	−30	−4	28.79	109.48
4	−28	−4	0.00	115.82
3	−25	−5	41.07	106.71
27	−21	9	31.56	71.84
21	−20	8	35.39	59.96
4	−17	−9	−153.45	96.01
0	−17	−4	312.46	79.63
0	−16	−5	333.98	94.42
−1	−13	−3	27.34	74.61
3	−12	−9	−31.69	104.46
21	−11	8	19.68	76.73
−1	−11	−5	56.79	70.52
12	−9	6	225.82	61.67
0	−8	−7	−4.75	78.44
23	−6	8	24.43	84.12
−1	−4	−6	190.83	67.62
26	−3	8	5.68	90.86
0	−2	−7	205.88	70.78
14	−1	6	−0.26	69.33
14	10	4	118.59	83.99
10	10	3	16.11	77.65
5	10	1	192.94	65.50
11	11	3	222.92	79.90
4	14	−4	274.55	52.56
18	15	3	67.22	114.10
12	18	0	54.28	73.43
12	19	−1	−23.64	76.20
10	19	−3	−11.36	69.73
14	−37	3	−24.30	82.54
23	−27	8	0.00	47.67

4	−26	−6	64.18	107.37
3	−25	−2	124.40	79.37
8	−20	4	45.96	34.73
2	−19	0	106.97	74.75
0	−16	−2	246.69	83.59
27	−15	9	−1.19	80.82
2	−14	−8	−0.13	113.18
30	−9	9	−19.94	92.97
16	−9	7	96.27	63.92
17	−5	7	−22.19	82.54
27	−2	8	82.14	94.56
10	6	4	57.84	80.03
22	7	6	−92.44	95.61
9	9	3	0.00	91.25
12	12	3	120.31	78.18
11	16	1	86.76	81.88
7	17	−4	108.55	59.43
17	20	0	−46.75	99.05
8	−35	−2	21.00	91.91
5	−29	−5	74.09	105.78
4	−27	−5	82.54	114.76
28	−26	9	50.71	67.88
4	−21	−8	68.28	106.31
2	−21	−1	−17.70	66.82
1	−20	−3	88.74	79.90
2	−18	−7	146.85	102.61
1	−17	−6	26.54	76.86
0	−14	−1	1244.01	125.33
5	−12	−10	8.72	90.33
−1	−12	−4	214.73	94.29
−1	−11	−2	204.43	65.24
29	−10	9	28.26	93.63
22	−7	8	−40.28	86.50
9	−5	5	133.12	68.01
4	−5	3	365.28	42.66
0	−5	−7	91.52	70.39
13	5	5	−36.32	76.99
14	6	5	30.77	78.97
15	7	5	70.12	76.07
16	8	5	−49.13	79.50
22	11	5	−44.64	94.95
15	11	4	110.40	81.48
20	18	2	9.11	98.91
17	21	−1	−0.92	96.93
14	−41	1	71.84	95.08
13	−41	0	0.00	93.50
23	−40	6	0.00	61.28
11	−39	−1	−23.64	87.95
23	−35	7	5.41	26.28
7	−33	−2	55.73	108.82
4	−27	−2	108.03	82.27
28	−11	9	−14.79	93.76
1	−9	1	128.76	35.00
−1	−6	−1	204.03	49.39

8	8	3	212.75	87.82
13	13	3	0.13	87.42
21	14	4	178.02	97.46
17	19	1	7.13	110.27
12	20	−2	53.48	93.24
21	21	0	−22.58	92.05
19	−46	2	−52.69	95.22
19	−44	3	−108.69	91.39
21	−41	5	−27.07	72.24
18	−40	4	−19.41	74.88
6	−31	−2	−121.63	109.87
5	−19	−9	−57.18	97.06
1	−13	−7	85.84	76.46
0	−12	−6	509.36	90.20
3	−9	−9	21.00	108.69
0	−9	0	1065.07	100.76
3	−3	−9	144.74	103.01
7	0	4	2454.75	214.73
0	3	−6	548.58	70.65
12	4	5	123.08	76.60
2	4	1	751.43	81.75
9	5	4	204.56	83.73
3	5	−8	33.81	54.54
0	7	−4	2087.49	172.21
23	8	6	−106.97	101.69
17	9	5	29.85	99.18
1	9	−2	82.27	33.68
16	12	4	−0.26	94.42
19	16	3	−10.04	98.25
12	17	1	36.05	80.95
8	18	−4	42.39	59.03
21	22	−1	49.13	98.25
13	−35	3	29.19	82.14
8	−25	3	34.34	56.52
5	−21	2	155.96	58.24
16	−18	7	−8.58	52.56
27	−12	9	54.81	89.80
21	−8	8	−6.21	83.73
12	−6	6	50.05	59.96
3	−6	−9	122.82	103.67
−1	−3	−1	694.64	73.95
13	−2	6	56.13	69.60
20	1	7	67.48	113.97
2	1	−8	55.20	62.33
−1	1	−5	59.82	57.84
4	5	2	1788.63	158.74
14	14	3	48.73	82.41
13	19	0	0.00	90.99
13	20	−1	−59.03	99.31
11	20	−3	0.00	93.50
17	22	−2	83.86	90.59
10	−37	−1	−18.22	89.01
27	−24	9	−43.18	68.80
3	−23	−1	83.46	75.41

2	−22	−3	65.11	86.63
26	−19	9	134.17	74.88
1	−19	−4	51.11	77.12
1	−18	−5	143.42	79.76
26	−16	9	−40.28	74.48
3	−16	−8	51.64	122.02
20	−15	8	−14.00	69.20
12	−15	6	155.44	44.50
9	−14	5	30.90	28.92
20	−12	8	3.96	71.97
2	−11	−8	69.20	90.33
19	0	7	28.66	105.52
21	2	7	37.24	101.03
5	2	3	476.21	67.35
4	9	1	216.32	58.24
18	10	5	45.30	113.04
17	13	4	65.24	107.89
3	13	−4	87.42	42.92
18	−46	1	62.86	91.39
12	−39	0	−51.64	87.69
12	−33	3	22.32	86.50
22	−25	8	17.04	50.45
18	−25	7	−37.77	15.58
2	−19	−6	−50.98	91.39
4	−14	−9	54.28	105.52
16	−6	7	36.18	65.37
18	−1	7	42.66	102.74
22	3	7	58.77	90.07
−1	3	−4	131.27	40.28
2	6	−7	286.70	53.35
7	7	3	1580.77	158.34
8	12	2	141.17	94.42
9	13	2	1.58	71.31
10	14	2	196.51	76.86
18	21	0	−52.82	92.44
22	−45	4	64.45	84.25
13	−39	1	−50.45	86.90
9	−36	−3	89.80	90.46
9	−35	−1	−25.22	96.54
9	−27	3	−3.57	57.97
3	−21	0	118.33	64.45
0	−15	−3	146.19	86.90
1	−14	0	126.91	58.90
26	−13	9	1.32	77.92
1	2	−7	30.37	58.37
11	3	5	186.47	75.01
23	4	7	0.00	88.48
17	5	6	74.75	102.08
18	6	6	0.00	109.61
2	8	0	9.64	40.01
19	11	5	−28.66	109.08
7	11	2	319.32	85.18
15	15	3	16.77	94.82
20	17	3	−110.80	94.03

17	18	2	−27.34	104.86
13	18	1	−35.92	81.75
9	19	−4	11.89	65.50
18	20	1	−4.49	95.88
13	21	−2	90.99	107.50
18	22	−1	−11.49	90.99
13	−37	2	−27.34	85.18
11	−31	3	6.34	89.93
10	−29	3	−5.15	67.35
3	−24	−3	241.14	85.05
4	−21	1	293.84	75.41
20	−18	8	10.30	63.79
1	−18	−2	107.23	82.67
6	−14	−10	−3.43	87.03
0	−13	−5	11.09	74.09
−1	−10	−3	133.78	72.24
23	−3	8	22.19	88.35
24	−2	8	−37.90	94.95
25	−1	8	−35.00	92.84
19	7	6	−87.82	107.10
18	14	4	57.05	110.01
11	15	2	114.63	81.22
14	20	0	4.36	105.25
18	−44	2	50.05	96.93
18	−42	3	0.00	83.73
17	−38	4	23.90	62.60
11	−37	0	148.44	91.78
8	−33	−1	−10.56	107.50
2	−21	−4	71.97	80.82
2	−20	−5	0.00	79.90
2	−15	−7	16.77	85.18
2	−14	1	644.19	73.82
1	−10	−7	−4.89	72.77
27	−9	9	−58.50	96.14
20	−9	8	82.27	77.26
0	−9	−6	27.73	68.80
−1	−9	−4	73.29	77.78
2	−8	−8	1.58	77.26
−1	−5	−5	581.46	91.25
22	−4	8	−20.73	86.76
17	−2	7	−12.94	90.59
8	4	4	101.82	76.73
14	21	−1	52.03	108.95
12	21	−3	40.41	104.33
22	−47	3	31.17	95.88
12	−37	1	0.92	85.58
7	−32	−3	5.02	112.38
7	−31	−1	0.00	94.56
6	−30	−3	5.41	111.06
6	−29	−1	−59.30	78.05
5	−28	−3	9.24	88.74
4	−26	−3	164.02	80.56
5	−24	−7	0.00	101.42
6	−23	2	24.04	56.79

26	−22	9	3.96	70.78
3	−21	−6	−67.62	92.84
4	−18	−8	43.32	120.31
0	−14	−4	564.69	101.69
15	−10	7	40.15	59.69
−1	−8	−2	2040.61	180.00
6	−4	4	151.34	48.33
2	−2	−8	0.00	65.90
−1	−2	−5	17.56	66.03
20	8	6	−9.38	108.16
1	8	−1	48.86	35.00
6	10	2	221.86	76.86
20	12	5	3.43	97.73
16	16	3	−68.14	121.36
12	16	2	−21.53	78.05
14	19	1	64.31	101.16
20	−39	5	0.00	67.35
12	−35	2	−1.72	87.16
3	−23	−4	19.55	76.73
5	−16	−9	−15.85	97.33
1	−16	−1	125.19	73.03
15	−13	7	51.24	55.60
4	−11	−9	−112.91	108.16
21	−5	8	102.61	88.48
2	−5	−8	87.03	70.92
12	−3	6	77.65	66.43
4	−2	3	755.39	74.61
5	15	−1	185.15	75.67
6	16	−1	131.93	68.67
7	17	−1	135.23	71.71
18	19	2	−33.28	94.95
17	−44	1	−160.59	104.06
10	−35	0	37.77	97.73
4	−23	−6	94.29	117.67
3	−22	−5	25.88	86.37
25	−17	9	−21.00	74.09
25	−14	9	−4.62	77.92
3	−13	−8	71.18	103.01
6	−11	−10	58.11	89.93
26	−10	9	−36.58	91.65
10	2	5	203.51	78.71
6	6	3	427.88	76.33
21	9	6	−65.50	105.12
19	15	4	30.11	100.10
8	18	−1	44.24	70.26
19	22	0	111.06	92.44
22	−38	6	−15.98	60.09
11	−38	−2	105.38	92.31
11	−35	1	7.00	90.07
8	−33	−4	142.10	98.78
4	−25	−4	−5.94	80.82
21	−23	8	−7.79	53.09
5	−23	1	48.33	61.14
5	−20	−8	0.00	103.93

2	−20	−2	0.00	69.07
7	−16	−10	38.30	91.78
0	−13	−2	506.45	85.31
15	−7	7	57.05	63.79
1	−7	−7	−4.62	72.90
16	−3	7	−17.83	77.78
0	5	−5	553.20	66.29
3	8	1	364.88	57.84
6	15	0	92.05	78.84
7	16	0	265.57	76.73
8	17	0	79.90	70.26
6	17	−2	−1.45	60.62
19	21	1	83.59	98.12
15	21	0	4.89	106.71
14	22	−2	−31.03	95.88
16	−36	4	34.60	69.73
11	−33	2	49.13	99.97
9	−33	0	0.00	106.71
7	−31	−4	79.90	102.88
5	−27	−4	77.39	95.08
7	−25	2	116.21	63.52
5	−25	−6	13.87	115.69
5	−25	0	80.56	72.11
4	−24	−5	−14.79	90.33
13	−20	6	19.28	18.75
3	−17	−7	44.50	92.71
1	−17	−3	−11.23	78.44
0	−11	−1	1230.15	118.72
4	−8	−9	49.52	108.82
0	−6	−6	305.19	72.63
0	−6	0	2416.32	205.49
1	−1	−7	22.58	63.52
0	0	−6	1161.87	118.33
13	1	6	0.00	72.63
1	10	−3	524.15	53.75
12	11	4	16.38	73.56
4	14	−1	574.99	87.03
5	16	−2	117.01	62.99
17	17	3	30.64	107.89
7	18	−2	98.52	72.24
15	20	1	0.00	107.76
15	22	−1	−88.74	98.91
19	23	−1	−24.96	92.84
21	−43	4	50.58	80.16
17	−42	2	18.88	91.65
17	−40	3	9.38	85.44
8	−31	0	−67.48	87.69
6	−29	−4	34.20	109.35
6	−27	−6	59.96	98.52
6	−27	0	72.11	72.63
5	−26	−5	0.00	110.54
25	−20	9	0.00	68.80
6	−18	−9	−18.22	91.25
1	−15	−5	45.16	73.56

19	−13	8	13.21	71.58
25	−11	9	−48.73	83.59
20	−6	8	59.82	89.93
−1	−6	−4	150.15	66.03
4	−2	−9	131.80	102.22
14	9	5	58.77	78.58
5	9	2	33.41	64.58
11	10	4	185.68	78.44
13	12	4	18.75	82.67
5	14	0	161.38	71.97
9	18	0	10.17	74.88
9	19	−1	60.48	72.11
21	−47	2	−25.62	97.46
15	−42	0	28.00	98.91
13	−40	−1	61.80	93.24
10	−36	−2	63.92	96.54
10	−33	1	−39.22	103.54
7	−30	−5	74.35	110.67
7	−29	0	59.30	71.31
6	−28	−5	5.94	116.87
8	−27	2	−2.38	63.13
6	−25	1	30.90	70.65
9	−22	4	141.04	42.92
6	−22	−8	−35.92	104.33
32	−17	10	−15.85	89.01
19	−10	8	0.00	75.14
6	−10	4	34.34	19.68
−1	−7	−3	143.42	57.45
27	−6	9	−47.54	95.35
4	−5	−9	92.97	107.50
1	−4	−7	81.48	68.94
0	−3	−6	934.33	106.84
19	3	7	16.51	106.04
7	3	4	1639.93	157.42
20	4	7	48.33	96.67
13	8	5	38.83	76.99
10	9	4	213.81	73.82
15	10	5	1.58	87.95
14	13	4	11.09	85.44
4	15	−2	135.89	68.41
8	19	−2	−24.70	66.69
19	20	2	−56.65	102.61
9	−31	1	0.00	81.75
9	−29	2	29.85	69.20
3	−22	−2	146.32	76.20
1	−16	−4	167.85	76.33
5	−13	−9	51.50	101.42
2	−12	−7	150.81	76.73
11	−7	6	240.75	60.35
23	0	8	−2.77	92.97
1	0	1	897.22	76.60
18	2	7	5.55	102.35
21	5	7	62.60	90.86
12	7	5	224.77	84.52

16	11	5	25.88	106.71
18	18	3	−13.21	96.40
10	19	0	147.51	76.33
10	20	−1	54.67	75.67
21	−45	3	0.00	89.41
9	−34	−2	−8.06	97.86
8	−29	1	0.00	70.26
7	−27	1	58.90	72.37
7	−20	−9	39.62	88.08
4	−19	−7	−60.48	115.03
2	−18	−1	142.76	79.37
4	−15	−8	78.44	114.50
11	−10	6	95.61	51.90
3	−10	−8	21.39	86.10
−1	−5	−2	1331.70	122.02
22	−1	8	0.00	87.29
6	−1	4	598.90	76.86
−1	0	−4	91.12	46.49
24	1	8	35.13	88.61
17	1	7	32.49	103.67
−1	1	−2	357.62	45.83
3	4	2	2131.60	185.41
22	6	7	22.19	89.93
17	12	5	−31.17	110.14
10	13	3	79.76	77.52
15	14	4	0.00	99.31
11	14	3	192.28	86.37
9	20	−2	50.98	68.41
16	22	0	−57.45	99.05
15	23	−2	31.83	90.99
19	−37	5	−43.18	65.37
4	−24	−2	90.86	79.10
2	−19	−3	158.34	76.07
15	−16	7	9.77	52.30
2	−16	0	163.36	65.77
24	−15	9	−82.14	75.67
7	−13	−10	9.24	92.18
0	−12	−3	79.63	72.63
1	−11	−6	49.52	78.31
19	−7	8	−22.32	78.58
1	−6	1	1038.79	91.65
15	−4	7	−5.55	69.20
21	−2	8	8.85	87.16
12	0	6	65.77	70.92
9	1	5	55.60	76.33
3	2	−8	53.09	61.41
9	8	4	88.88	75.67
3	13	−1	151.34	61.67
12	15	3	−20.21	83.20
9	17	1	85.31	75.14
15	19	2	−36.58	112.78
16	21	1	45.96	99.71
14	−40	0	−113.57	94.42
12	−38	−1	−0.53	92.57

8	−32	−2	−50.84	112.91
27	−27	9	8.58	61.67
5	−26	−2	31.56	77.39
6	−18	3	35.00	49.26
4	−16	2	372.68	61.28
24	−12	9	61.54	75.01
0	−11	−4	382.84	94.16
8	−6	5	200.86	64.97
5	5	3	700.85	85.71
15	6	6	0.00	86.90
11	6	5	17.04	74.48
16	7	6	152.53	108.95
17	8	6	31.17	113.57
8	11	3	69.33	83.99
18	13	5	−17.04	100.89
6	14	1	160.98	82.14
16	15	4	0.00	116.21
10	18	1	36.45	80.16
11	20	0	41.60	83.46
16	23	−1	−60.62	90.73
16	−38	3	52.16	79.37
6	−28	−2	−17.17	75.94
17	−23	7	24.30	24.96
20	−21	8	−41.73	55.60
31	−18	10	−2.25	83.59
24	−18	9	−40.94	69.60
3	−18	−6	0.00	77.39
2	−17	−5	−12.81	77.39
11	−4	6	583.71	82.80
20	−3	8	106.04	90.86
16	0	7	−38.30	92.97
18	9	6	0.00	105.91
3	14	−2	560.73	79.90
13	16	3	−20.87	81.61
11	21	−1	101.42	92.44
24	−46	4	30.51	90.33
15	−40	1	0.66	88.88
26	−25	9	−59.69	65.11
2	−18	−4	19.41	78.97
31	−15	10	−1.32	94.03
1	−15	−2	78.97	73.56
4	1	3	377.30	56.26
14	5	6	35.79	76.33
19	10	6	92.57	105.25
2	12	−4	26.68	27.34
5	13	1	−22.98	74.88
17	16	4	0.00	103.93
6	18	−3	41.60	59.82
11	19	1	71.18	83.86
16	20	2	−49.52	103.01
10	21	−2	46.22	86.10
24	−48	3	70.65	99.71
20	−45	2	0.00	95.22
20	−41	4	14.79	79.24

11	−36	−1	50.98	93.90
22	−33	7	11.49	20.34
14	−32	4	0.00	73.43
3	−21	−3	269.67	87.03
3	−20	−1	12.02	79.63
5	−17	−8	69.20	119.91
3	−16	1	241.14	62.99
6	−15	−9	10.56	98.39
3	−14	−7	245.37	88.61
5	−10	−9	−40.28	110.40
8	−9	5	167.85	52.96
3	−7	−8	156.10	76.20
−1	−4	−3	289.21	53.75
8	−3	5	194.00	71.18
−1	−2	−2	445.44	57.31
10	5	5	47.94	71.58
8	7	4	103.80	85.18
4	8	2	116.21	62.86
7	10	3	187.53	96.54
19	14	5	11.75	101.42
14	17	3	15.45	105.25
5	17	−3	118.72	59.69
7	19	−3	96.14	65.50
12	21	0	32.35	99.71
17	23	0	−76.20	93.63
19	−45	1	−89.54	98.12
13	−38	0	68.80	88.48
21	−36	6	−54.67	56.92
7	−25	−7	99.18	103.67
10	−24	4	157.55	49.52
4	−20	−6	13.07	80.03
8	−15	−10	−3.83	90.33
2	−9	−7	124.80	75.80
14	−8	7	39.22	61.80
0	−7	−5	1346.63	141.17
26	−4	9	−52.69	96.93
20	11	6	44.64	97.59
17	22	1	115.95	93.10
23	−42	5	−3.57	76.46
9	−33	−3	78.18	101.82
25	−23	9	35.26	64.05
3	−19	−5	92.71	75.41
30	−16	10	−50.58	85.71
1	−12	−5	33.68	73.56
18	−11	8	−3.30	72.50
14	−11	7	153.45	60.35
1	−11	0	358.15	55.73
25	−5	9	109.87	97.86
19	−4	8	42.92	93.24
3	−4	−8	11.75	68.80
15	−1	7	−50.05	82.93
3	−1	−8	40.94	68.01
2	3	−7	124.53	58.64
18	17	4	70.65	96.27

15	18	3	−131.14	114.36
12	20	1	−23.37	92.31
8	20	−3	−4.75	67.75
12	22	−1	12.55	106.18
11	22	−2	−20.73	105.52
17	24	−1	−40.28	97.86
20	−43	3	−46.75	90.20
13	−39	−2	−47.41	91.25
15	−38	2	0.00	86.10
18	−35	5	0.00	62.33
10	−34	−1	58.11	111.59
8	−31	−3	−53.75	117.01
13	−30	4	92.05	82.54
4	−23	−3	166.00	82.54
3	−20	−4	49.52	70.26
30	−19	10	−41.47	82.41
6	−19	−8	15.45	108.69
23	−13	9	12.02	74.88
2	−13	−6	132.46	77.52
4	−12	−8	−1.06	96.93
0	−10	−2	4347.32	365.28
18	−8	8	90.73	77.39
1	−8	−6	−67.09	68.01
14	−5	7	35.00	65.37
0	−3	0	9020.82	732.67
−1	−1	−3	135.76	40.28
1	4	−6	94.03	47.01
1	7	0	753.14	77.12
9	16	2	14.00	79.24
4	16	−3	183.96	55.86
10	17	2	77.92	78.84
6	17	−5	41.60	50.71
14	−38	1	−124.80	88.88
10	−34	−4	−50.71	95.74
7	−29	−3	−3.04	88.88
12	−28	4	−14.13	63.26
6	−27	−3	29.58	80.95
11	−26	4	21.13	51.64
5	−25	−3	−6.21	86.63
5	−22	−6	−39.49	94.56
3	−18	0	57.84	74.88
7	−17	−9	118.19	94.95
30	−13	10	67.62	98.39
1	−13	−1	285.91	64.31
23	−10	9	−19.68	78.18
5	−7	−9	24.30	108.42
11	−1	6	9.11	66.82
22	2	8	46.35	92.31
6	2	4	413.75	77.12
23	3	8	17.96	92.05
18	5	7	−13.60	100.63
19	6	7	0.00	98.91
2	7	1	1204.92	115.03
4	12	1	531.81	87.95

7	14	2	292.38	85.05
17	21	2	−40.54	92.84
9	21	−3	16.64	84.12
13	22	0	125.72	111.59
23	−48	2	7.40	99.31
12	−36	0	−38.83	96.27
9	−32	−1	−2.77	103.67
6	−24	−6	147.38	117.80
4	−22	−4	8.19	81.88
7	−21	−8	−6.74	101.82
4	−21	−5	30.24	78.97
9	−17	−10	−17.83	99.31
23	−16	9	7.66	68.41
4	−16	−7	−23.64	83.73
18	−14	8	0.00	67.88
1	−14	−3	−20.87	77.26
11	−13	6	64.45	45.83
0	−8	−1	113.84	47.15
24	−6	9	8.06	91.25
21	1	8	0.00	90.59
17	4	7	74.88	112.25
20	7	7	7.00	89.80
3	7	−7	274.16	50.84
2	8	−6	40.67	31.96
6	9	3	50.45	76.99
2	12	−1	729.37	81.75
16	19	3	−64.45	101.03
13	21	1	44.37	111.20
18	23	1	0.00	89.67
18	24	0	21.53	100.50
17	−43	0	0.00	98.25
15	−41	−1	27.47	93.90
12	−37	−2	−42.26	88.74
9	−32	−4	90.20	106.04
8	−30	−1	31.03	82.80
8	−28	−6	−10.04	110.14
7	−26	−6	−43.84	124.80
5	−24	−1	29.05	73.03
19	−19	8	56.13	59.69
2	−17	−2	123.21	87.29
1	−13	−4	90.33	81.61
2	−6	−7	30.24	65.90
5	−4	−9	17.70	103.40
0	−4	−5	1046.98	117.80
20	0	8	33.94	94.56
0	2	−5	271.65	60.75
9	4	5	−11.89	71.84
7	6	4	75.94	81.61
5	16	−5	34.47	45.83
12	19	2	−2.11	89.14
13	23	−1	47.54	104.72
19	−43	2	−88.74	93.76
9	−31	−5	−33.41	96.80
8	−30	−4	0.00	118.59

7	−28	−1	158.34	79.63
5	−24	−4	−58.90	83.33
8	−23	−8	5.41	101.95
5	−23	−5	165.21	82.93
24	−21	9	24.17	67.22
8	−19	−9	−2.91	92.71
8	−12	−10	0.00	91.78
6	−12	−9	0.53	107.23
0	−8	−4	16.77	74.61
18	−5	8	−48.99	93.76
16	3	7	−61.67	102.74
13	11	5	38.30	78.97
14	12	5	1.32	95.35
15	13	5	1.85	108.03
11	13	4	24.56	80.16
6	13	2	191.88	89.93
2	13	−2	377.56	64.71
12	14	4	8.58	77.65
12	23	−2	47.94	104.20
19	−39	4	11.89	74.35
14	−36	2	0.00	88.74
13	−36	1	43.32	92.57
14	−34	3	22.32	84.52
8	−29	−5	−54.41	116.74
7	−28	−4	−80.03	101.42
7	−27	−5	113.97	121.50
6	−26	−4	71.05	84.25
6	−25	−5	−38.56	97.73
29	−17	10	55.99	81.75
29	−14	10	24.17	87.69
5	−14	−8	−25.88	109.21
3	−11	2	621.22	60.62
0	−9	−3	249.33	67.48
23	−7	9	−42.13	91.39
14	−2	7	119.38	68.01
12	10	5	73.03	78.44
10	12	4	275.35	83.73
13	15	4	27.73	94.95
10	22	−3	−1.72	101.95
14	23	0	105.52	101.42
23	−46	3	−6.07	90.20
23	−44	4	1.58	82.93
11	−34	0	68.28	108.55
7	−20	3	131.80	49.13
4	−20	0	30.37	76.73
5	−18	−7	−18.49	100.23
5	−18	2	260.69	65.50
2	−14	−5	115.29	73.69
3	−11	−7	73.82	77.26
4	−9	−8	21.66	78.18
1	−5	−6	66.69	67.09
2	−3	−7	2.38	64.71
2	−3	2	436.99	45.16
19	−1	8	0.00	89.93

0	−1	−5	143.02	64.97
2	0	−7	283.27	67.75
11	9	5	0.00	77.78
16	14	5	48.86	109.87
3	15	−3	52.56	54.41
14	16	4	0.00	114.50
13	20	2	−75.41	116.61
14	22	1	0.40	103.80
19	−41	3	3.96	89.27
14	−39	−1	73.16	96.01
11	−35	−2	−53.75	103.54
17	−33	5	7.79	59.30
14	−14	7	221.60	57.18
29	−11	10	−2.38	99.31
2	−11	1	608.41	64.97
25	−2	9	22.19	92.18
2	0	2	150.95	40.67
15	2	7	−52.69	93.37
4	4	3	498.40	69.33
15	9	6	−58.37	108.03
16	10	6	0.00	110.93
17	11	6	−40.81	113.97
9	11	4	95.61	75.01
17	15	5	9.38	102.08
14	24	−1	14.79	91.65
16	−41	0	−20.60	95.61
10	−32	0	31.69	93.63
29	−20	10	−5.02	78.84
3	−19	−2	147.64	90.46
2	−16	−3	78.71	85.58
22	−11	9	22.45	76.99
10	−5	6	81.88	60.22
24	−3	9	0.00	93.90
11	2	6	185.02	76.07
3	7	2	3874.14	326.98
14	8	6	−92.31	93.90
2	11	0	130.48	51.37
18	12	6	101.16	106.31
5	12	2	106.57	75.67
15	17	4	0.00	125.06
13	24	−2	2.64	94.42
20	−34	6	0.00	52.96
12	−34	1	22.19	104.46
10	−33	−2	−94.95	123.34
13	−32	3	86.63	95.08
6	−20	−7	20.34	118.59
23	−19	9	−3.57	65.77
2	−15	−4	112.52	80.56
22	−14	9	33.41	71.84
9	−14	−10	−112.52	91.91
7	−14	−9	11.62	102.22
2	−10	−6	36.98	71.58
17	−9	8	22.19	75.01
6	−9	−9	34.86	110.93

18	−2	8	−85.71	94.29
1	−2	−6	272.57	69.86
1	1	−6	333.19	64.45
4	3	−8	284.72	66.82
1	3	1	902.77	90.20
10	8	5	119.91	79.76
5	8	3	237.18	76.73
3	11	1	1483.05	140.51
9	15	3	87.29	79.76
10	16	3	65.37	81.35
11	17	3	72.50	81.09
14	21	2	103.80	110.80
15	23	1	−23.11	93.37
15	24	0	23.77	89.54
22	−46	2	−14.00	94.95
22	−40	5	5.28	74.35
12	−36	−3	6.21	89.27
13	−34	2	15.85	96.93
9	−30	0	13.87	77.26
6	−16	−8	26.54	115.95
28	−15	10	62.60	83.07
28	−12	10	−59.56	93.24
17	−6	8	0.00	77.78
23	−4	9	80.56	94.29
13	7	6	15.85	80.03
8	10	4	47.94	71.58
18	16	5	4.36	91.52
16	18	4	17.04	96.01
12	18	3	149.36	97.20
7	19	0	205.75	77.92
18	−41	2	−24.17	95.61
17	−41	1	1.19	94.03
4	−21	−2	151.61	77.12
2	−15	−1	378.49	76.20
17	−12	8	−45.69	69.20
10	−8	6	−46.49	52.96
13	−6	7	37.51	64.58
4	−6	−8	3.30	71.31
10	−2	6	43.84	64.31
8	3	5	213.81	83.99
21	4	8	−41.86	92.44
6	5	4	294.76	77.26
8	14	3	−20.73	76.73
6	18	0	−6.60	73.56
6	19	−1	−29.98	69.07
8	20	0	38.30	75.67
7	20	−1	−18.36	72.50
15	25	−1	0.13	91.91
15	22	2	33.54	105.52
13	19	3	0.00	115.29
9	21	0	64.18	76.33
8	21	−1	−47.41	75.27
18	8	7	1.98	81.22
20	3	8	−34.60	92.18

5	18	−1	66.82	66.43
5	17	0	105.78	73.69
12	6	6	15.45	76.07
14	1	7	61.14	81.88
28	−18	10	23.64	80.69
13	−9	7	0.79	59.56
4	−13	−7	−24.04	76.73
1	−12	−2	163.89	65.24
3	−16	−5	18.09	75.41
5	−20	1	127.31	72.50
8	−24	−7	0.00	96.01
9	−26	−7	65.11	108.16
7	−26	0	118.72	75.80
8	−28	0	−5.28	69.99
11	−32	1	31.43	91.25
13	−37	−1	41.20	91.12
17	19	4	−13.34	99.05
9	20	1	12.28	76.73
8	19	1	−38.83	79.24
7	20	−4	−13.60	65.11
7	18	1	−43.05	75.80
17	7	7	23.90	103.01
19	2	8	1.06	89.27
7	13	3	−9.38	75.14
28	−9	10	44.77	99.05
4	15	−5	53.09	40.67
22	−5	9	35.66	90.86
13	−3	7	30.11	67.48
5	−2	4	432.50	61.67
3	−8	−7	52.96	71.97
10	−16	−10	20.21	95.48
5	−11	−8	−1.45	87.69
8	−16	−9	−30.51	101.55
7	−18	−8	59.03	116.08
8	−22	3	−5.15	52.03
5	−19	−6	46.22	79.37
3	−18	−3	117.40	84.52
12	−30	3	−10.17	74.35
5	−23	−2	30.24	74.61
18	−37	4	28.66	73.56
12	−32	2	149.76	99.84
8	−29	−2	40.28	82.14
15	−39	0	29.05	87.42
16	25	0	−2.38	90.20
16	24	1	43.84	88.61
10	22	0	−4.09	89.41
9	22	−1	−68.54	86.10
6	19	−4	−12.15	56.92
16	6	7	−13.34	107.23
9	7	5	10.04	73.29
4	11	2	78.05	70.92
17	−3	8	159.13	97.86
22	−17	9	−13.47	68.14
4	0	−8	264.91	71.71

4	−3	−8	46.09	66.82
6	−6	−9	49.39	103.27
0	−7	−2	7511.89	617.39
2	−13	0	120.70	50.45
6	−20	2	168.64	64.45
3	−17	−4	265.97	81.09
16	−31	5	−17.30	59.69
6	−25	−2	71.58	80.95
7	−27	−2	93.10	82.67
18	−39	3	30.90	83.33
10	21	1	2.77	86.37
4	17	−1	206.02	73.69
7	9	4	220.54	90.33
27	−13	10	18.49	83.33
2	3	2	387.33	55.07
1	−10	−4	58.37	80.69
8	−20	−8	15.45	107.23
4	−18	−5	147.25	81.48
11	−28	3	68.28	63.13
10	−30	1	−14.13	73.29
22	−44	3	−68.80	89.41
10	−32	−3	0.00	114.63
12	−35	−1	−75.14	102.74
16	23	2	−80.16	96.54
11	23	0	−122.42	111.72
10	23	−1	−37.77	118.85
7	21	−2	162.83	74.09
6	20	−2	20.87	69.46
5	18	−4	90.07	56.26
23	−1	9	−3.43	94.56
15	5	7	50.45	107.89
4	16	0	20.87	80.16
18	1	8	3.83	90.07
27	−10	10	−37.37	93.50
10	1	6	38.83	68.14
21	−12	9	6.34	73.56
5	1	4	647.76	82.41
7	−4	5	890.88	102.88
6	−3	−9	123.48	100.50
7	−11	−9	99.18	107.76
9	−18	−9	−51.90	94.16
11	−21	5	169.43	32.62
1	−11	−3	160.59	68.67
9	−24	3	−6.47	53.88
6	−21	−6	−11.36	87.42
10	−26	3	141.70	61.41
6	−22	1	125.72	65.50
4	−20	−3	39.49	75.14
11	−30	2	−18.22	75.01
10	−29	−6	64.97	100.76
22	−42	4	0.00	79.76
11	−33	−4	0.00	95.35
11	22	1	12.28	111.06
13	14	5	55.86	100.76

6	12	3	33.68	84.65
21	−6	9	31.43	82.27
1	11	−1	77.26	42.39
27	−16	10	50.84	81.48
0	8	−2	537.88	51.90
0	−6	−3	32.09	50.45
3	−12	−6	320.78	87.69
5	−15	−7	−19.55	79.24
9	−22	−8	51.90	104.33
3	−17	−1	915.05	108.42
7	−23	−6	−32.35	108.55
8	−25	−6	−60.35	117.27
9	−27	−6	11.49	118.59
9	−28	1	19.94	67.62
9	−30	−3	87.69	101.95
16	−39	1	−14.26	88.08
14	−38	−2	−48.86	96.93
8	22	−2	53.62	78.97
14	15	5	29.98	117.40
8	18	2	0.79	76.33
12	13	5	−62.33	92.05
5	19	−2	21.92	65.50
22	−2	9	8.32	92.97
2	14	−3	52.43	54.67
13	0	7	39.88	76.60
4	7	3	1474.20	143.55
0	7	−1	990.85	89.14
2	−7	−6	107.89	66.43
1	−6	−5	453.50	88.22
10	−20	−9	−133.91	103.14
7	−22	2	42.26	58.37
5	−20	−5	171.28	85.71
4	−19	−4	66.69	72.77
7	−24	1	95.48	66.29
5	−22	−3	32.88	75.80
8	−26	1	71.97	69.46
10	−31	−4	−68.54	120.31
17	−39	2	64.31	86.90
21	−44	2	97.86	97.99
14	−37	0	3.30	98.65
20	−44	1	118.33	102.74
12	24	0	16.11	103.01
12	23	1	−74.75	116.35
11	24	−1	150.55	110.67
9	23	−2	81.09	99.84
15	16	5	−6.47	101.42
10	20	2	0.00	85.71
16	13	6	−50.58	99.05
15	12	6	34.73	119.52
11	16	4	33.15	85.71
14	11	6	−39.35	111.86
7	17	2	129.55	82.41
11	12	5	6.87	86.63
27	−7	10	−18.75	96.01

14	4	7	−53.48	93.24
17	0	8	135.36	98.39
8	6	5	1.85	70.12
2	10	1	712.47	83.07
16	−7	8	95.74	75.27
28	−21	10	100.63	78.71
0	−5	−1	705.07	82.14
6	−13	−8	−66.95	99.97
19	−32	6	3.83	49.13
15	−29	5	−54.01	63.79
24	−39	6	3.04	64.45
25	−43	5	43.84	81.88
10	−28	2	192.54	67.09
6	−24	−3	92.18	79.24
7	−26	−3	164.42	83.86
10	−30	−5	62.73	105.91
8	−28	−3	−12.81	82.80
25	−47	3	80.69	94.16
11	−33	−1	97.06	108.69
16	17	5	−44.77	99.97
11	21	2	1.19	115.16
17	14	6	0.00	104.06
12	17	4	138.27	105.78
10	15	4	45.16	83.73
3	16	−1	234.28	79.90
1	12	−2	279.84	50.45
16	−4	8	33.41	92.05
3	4	−7	260.82	59.03
17	−15	8	0.00	61.54
13	−12	7	42.66	53.35
3	−5	−7	132.59	73.43
0	−2	−4	587.94	73.03
10	−13	−10	58.11	90.20
1	−10	−1	1.32	45.16
8	−24	2	−9.51	59.03
6	−22	−5	43.98	79.10
5	−21	−4	29.19	82.80
21	−38	5	21.53	67.22
9	−26	2	57.84	64.31
7	−24	−5	99.18	84.65
17	−35	4	61.80	71.45
8	−26	−5	−61.14	100.37
9	−28	−5	136.68	121.10
9	−29	−4	152.53	125.06
16	−40	−1	−87.42	99.97
13	18	4	0.79	112.38
20	6	8	106.44	93.24
13	10	6	−70.52	98.39
9	14	4	127.17	80.82
6	16	2	0.00	71.97
4	18	−2	26.15	61.28
10	11	5	151.47	84.25
4	15	1	142.89	90.20
21	−3	9	−36.18	89.80

3	15	0	126.78	74.61
26	−11	10	−41.20	86.24
6	8	4	10.96	82.14
7	2	5	32.88	76.20
21	−15	9	37.24	70.39
16	−10	8	−37.11	68.14
5	−8	−8	30.51	79.50
15	−19	7	24.96	55.73
4	−10	−7	50.45	74.88
6	−17	−7	19.68	84.78
2	−14	−2	150.29	69.73
6	−23	−4	58.24	84.25
7	−25	−4	27.73	80.29
8	−27	−4	54.54	99.97
10	−31	−1	50.32	91.65
13	−36	−2	89.27	105.38
18	−42	0	−150.68	100.50
13	24	1	0.00	105.25
12	25	−1	−57.45	95.74
12	22	2	182.11	123.08
10	24	−2	−47.81	110.01
14	19	4	−1.06	117.01
19	5	8	−29.71	91.25
4	17	−4	175.64	55.60
5	11	3	5.02	82.93
26	−14	10	47.81	81.22
5	4	4	297.93	73.95
27	−19	10	7.26	77.26
0	4	−4	1334.08	117.40
3	0	3	1246.52	113.04
10	−11	6	52.16	47.01
7	−8	−9	−107.23	110.54
8	−12	5	170.36	37.51
8	−13	−9	−46.49	102.22
1	−8	0	577.77	66.95
4	−14	−6	316.29	91.25
4	−19	−1	180.53	83.59
25	−45	4	86.76	90.73
15	−37	1	146.32	90.73
13	−35	0	−39.22	101.29
24	−47	2	0.00	99.71
13	25	0	25.62	92.97
18	4	8	−22.71	89.14
12	9	6	38.96	84.39
26	−8	10	11.49	94.16
20	−7	9	−14.66	79.90
3	10	2	293.97	65.50
1	10	0	451.65	61.28
12	−4	7	43.18	65.37
1	6	1	189.11	51.64
0	3	0	9627.64	781.80
24	−24	9	−5.28	57.18
7	−7	5	1342.27	127.04
7	−15	−8	−18.09	115.82

2	−12	−4	17.17	78.31
7	−19	−7	17.70	99.18
14	−27	5	−22.05	61.80
21	−42	3	−86.76	91.52
14	−37	−3	40.01	92.05
13	23	2	99.71	101.03
15	20	4	−21.26	100.63
18	11	7	60.62	92.05
8	13	4	20.73	80.16
5	15	2	42.92	80.29
9	10	5	122.95	80.69
13	3	7	−42.39	84.78
16	−1	8	0.00	98.25
2	6	2	1403.41	129.02
9	−3	6	12.28	59.82
3	−2	−7	54.01	64.84
25	−26	9	−10.43	55.99
11	−15	−10	0.92	93.76
12	−18	6	−9.90	26.02
3	−15	0	303.48	64.31
9	−29	−1	132.85	78.71
16	−37	2	1.32	88.61
12	−34	−2	67.88	118.33
11	25	−2	27.34	97.06
10	19	3	19.02	87.16
9	18	3	−27.34	79.10
22	1	9	−92.97	93.50
15	8	7	36.58	109.74
12	−1	7	50.84	71.18
3	3	3	721.19	79.37
12	−7	7	39.35	61.41
3	1	−7	101.42	62.73
23	−22	9	0.00	59.56
0	1	−4	145.80	46.62
2	−4	−6	99.05	67.75
5	−13	3	250.78	37.51
3	−13	−5	72.11	74.88
12	−23	5	69.07	36.85
2	−13	−3	−49.13	76.86
8	−21	−7	0.00	114.63
5	−21	−1	9.64	78.58
21	−40	4	102.61	80.43
19	−42	1	0.00	94.82
15	−38	−1	5.55	94.29
14	25	1	−2.38	97.73
13	26	−1	−37.77	90.33
11	20	3	54.41	116.48
8	17	3	136.29	81.35
17	3	8	−9.51	94.95
3	17	−2	84.12	62.73
11	8	6	81.88	83.07
20	−4	9	23.77	84.25
26	−17	10	−22.32	77.92
9	0	6	69.46	65.77

5	4	−8	71.58	59.56
1	6	−5	138.14	45.83
5	−5	−8	0.00	73.95
1	−3	−5	78.84	65.11
9	−15	−9	46.49	106.84
3	−13	1	125.33	45.69
13	−25	5	9.64	45.56
10	−25	−7	−149.49	104.99
6	−23	−1	50.45	70.52
7	−25	−1	104.86	76.99
12	−33	0	10.17	106.71
20	−42	2	49.26	94.95
14	26	0	97.33	97.33
14	24	2	−99.57	94.69
21	0	9	−1.72	93.24
7	12	4	165.47	77.26
3	14	1	844.00	109.74
25	−9	10	20.60	89.41
25	−12	10	19.15	83.59
7	5	5	102.74	80.69
20	−13	9	10.83	71.58
9	−6	6	−1.32	55.47
7	−5	−9	173.00	107.23
12	−17	−10	56.92	96.93
3	−9	−6	0.00	59.96
1	−7	−4	89.14	68.94
8	−17	−8	70.52	125.72
3	−16	−2	99.97	75.41
14	−35	1	−12.68	100.89
17	−40	0	−12.15	88.48
8	23	−3	1.85	86.37
7	22	−3	132.59	79.90
6	21	−3	−10.96	66.29
7	16	3	228.86	86.10
14	7	7	61.94	119.25
2	15	−1	198.49	69.33
2	5	−6	−2.25	44.50
22	−20	9	−14.53	62.60
3	−3	3	1187.49	104.72
6	−10	−8	−1.45	86.37
5	−12	−7	3.17	72.90
16	−35	3	16.38	83.59
24	−45	3	34.34	89.67
15	26	1	27.60	97.86
25	−6	10	43.71	95.61
16	2	8	1.06	96.93
4	14	2	146.59	87.69
8	9	5	99.05	81.35
4	8	−7	78.97	41.20
16	−13	8	0.00	66.43
8	−10	−9	−0.92	112.65
0	−3	−3	1219.06	112.52
10	−17	−9	43.45	106.04
9	−19	−8	−5.55	118.06

3	−14	−4	241.41	86.76
6	−18	−6	−32.49	78.58
13	−34	−4	48.73	111.46
16	−39	−2	73.43	93.76
5	20	−3	−15.45	62.60
20	−1	9	42.79	91.52
10	7	6	162.04	84.52
12	2	7	131.27	76.60
4	10	3	320.91	85.44
5	7	4	85.71	84.39
25	−15	10	19.02	73.56
5	−2	−8	67.48	70.52
4	−7	−7	125.46	75.27
0	−4	−2	2825.58	238.77
2	−8	−5	56.65	82.01
1	−9	−2	1604.67	148.04
10	−21	−8	68.54	112.78
4	−15	−5	78.31	73.43
18	−30	6	0.00	45.83
3	−15	−3	44.77	78.84
20	−36	5	15.19	64.97
15	−35	2	−40.41	93.90
11	−31	0	43.84	80.16
10	−30	−2	−20.87	82.80
27	−48	3	−0.13	100.23
12	−33	−3	−94.95	117.01
14	−36	−1	84.65	100.37
22	−45	1	−107.89	100.23
15	27	0	0.00	93.90
14	23	3	3.57	92.31
15	15	6	−110.93	100.76
13	17	5	−4.75	115.16
7	22	0	−17.30	78.44
14	14	6	13.21	102.61
12	16	5	−11.36	118.19
19	8	8	−35.92	100.10
6	15	3	74.88	81.75
13	6	7	52.43	105.12
19	−5	9	0.00	83.20
15	−2	8	8.06	105.38
19	−8	9	−52.16	78.58
15	−8	8	44.50	70.26
5	1	−8	117.53	64.58
2	−1	−6	266.50	66.95
11	−19	−9	110.40	103.80
11	−23	−8	47.41	99.18
7	−20	−6	35.52	87.16
4	−17	0	278.65	71.31
4	−18	−2	90.86	83.86
24	−41	5	−54.54	77.12
11	−28	−6	−39.88	111.20
23	−45	2	−0.92	96.54
18	−40	1	128.36	91.91
14	18	5	−13.87	99.84

8	23	0	21.79	85.84
8	22	1	−1.32	83.20
7	21	1	165.21	81.48
6	21	0	−8.98	73.82
13	13	6	0.00	110.67
11	15	5	−26.68	98.12
18	7	8	21.79	96.80
3	16	−4	250.78	54.28
6	11	4	133.12	75.41
3	6	3	219.22	61.54
21	−18	9	56.52	63.13
11	−12	−10	−36.71	83.20
14	−17	7	40.15	46.62
20	−24	8	11.62	41.34
1	−8	−3	105.25	54.81
6	−14	−7	104.46	83.99
2	−12	−1	801.61	89.41
8	−22	−6	21.79	103.40
9	−24	−6	0.00	108.95
10	−26	−6	0.00	113.84
24	−43	4	−82.01	86.10
12	−31	−5	−74.48	105.78
9	−28	−2	−32.49	83.07
20	−40	3	38.17	87.42
13	−33	1	−25.49	105.38
12	−32	−4	−0.13	121.63
15	19	5	5.15	90.46
9	24	0	73.69	110.54
9	23	1	−25.22	100.63
6	20	1	59.43	79.63
5	20	0	69.33	78.18
21	3	9	71.58	95.35
12	12	6	−12.94	106.57
10	14	5	17.96	83.46
17	6	8	0.00	95.61
4	19	−3	51.90	61.67
24	−7	10	113.97	95.74
24	−10	10	−50.32	83.59
2	2	−6	176.70	57.97
12	−10	7	128.36	58.11
1	0	−5	50.58	59.56
19	−22	8	45.30	46.75
7	−12	−8	79.37	92.57
4	−13	2	43.32	36.32
5	−17	−5	144.21	89.27
23	−37	6	−38.03	63.52
15	−31	4	−43.05	85.84
10	−29	0	11.36	75.94
11	−31	−3	87.69	109.35
16	−38	0	53.75	90.99
20	−43	0	−1.19	96.01
18	−41	−1	3.30	94.95
10	24	1	−45.96	119.91
11	19	4	41.47	118.06

7	23	−1	97.59	82.54
10	18	4	−6.07	95.08
6	22	−1	33.94	74.22
2	16	−2	163.76	69.73
19	−2	9	−24.04	87.69
15	1	8	50.05	101.82
0	6	0	2783.98	235.07
9	−12	−9	128.23	108.29
4	−11	−6	255.93	80.56
4	−16	−4	121.36	83.46
4	−17	−3	262.27	90.73
6	−22	−2	0.53	71.84
20	−38	4	0.00	71.31
15	−33	3	0.00	94.03
11	−29	−5	0.00	118.06
8	−26	−2	−14.39	78.05
13	−34	−1	1.19	117.27
15	−37	−2	63.26	92.57
10	25	0	0.00	108.82
12	20	4	−0.13	110.93
8	24	−1	159.66	102.74
8	21	2	9.90	81.48
16	12	7	0.00	93.37
15	11	7	0.13	93.76
9	17	4	78.05	90.59
5	19	1	32.22	74.35
20	2	9	−55.60	94.16
9	13	5	7.00	79.76
5	14	3	146.32	78.84
7	8	5	88.74	78.44
2	13	1	346.79	74.35
24	−13	10	−1.19	78.18
2	9	2	889.56	99.31
1	9	1	49.52	50.84
6	1	5	68.28	75.01
1	3	−5	313.91	58.90
6	−19	−5	−5.55	79.10
5	−19	0	199.68	76.07
10	−27	−5	38.17	112.52
7	−24	−2	−12.81	76.73
9	−27	0	−4.23	72.90
27	−46	4	29.19	94.29
14	−33	2	15.05	103.01
11	−30	−4	−0.40	112.91
10	−29	−3	42.39	96.80
11	25	1	−9.51	98.12
13	21	4	57.18	100.37
10	23	2	53.09	115.69
9	22	2	81.48	99.44
5	21	−1	28.13	70.52
4	19	0	146.46	74.09
11	11	6	77.12	90.73
16	5	8	−7.53	93.10
24	−4	10	−13.07	94.42

12	5	7	18.49	92.05
3	13	2	189.51	81.48
9	6	6	165.60	77.39
11	−2	7	81.61	68.14
26	−20	10	−7.66	66.29
8	−7	−9	−120.31	113.04
6	−7	−8	15.98	76.33
12	−14	−10	−48.99	91.65
7	−16	−7	136.95	85.44
9	−25	−5	113.97	91.39
11	26	0	−17.43	98.52
9	25	−1	109.21	113.04
6	19	2	−80.82	77.39
8	16	4	−56.13	81.09
3	14	−5	146.72	33.81
11	1	7	21.53	62.86
11	−5	7	0.00	62.46
20	−16	9	41.07	65.63
15	−11	8	29.05	67.48
4	−4	−7	146.98	72.24
18	−20	8	0.00	48.33
3	−6	−6	201.26	72.11
21	−26	8	9.51	33.68
8	−14	−8	−13.60	110.93
4	−15	1	152.79	54.01
5	−18	−4	27.60	72.11
7	−21	−5	128.36	81.09
5	−19	−3	42.79	83.07
8	−23	−5	41.47	86.76
6	−21	0	106.18	79.24
8	−25	0	69.46	71.71
10	−28	−4	−33.15	93.50
9	−27	−3	117.67	80.16
12	−31	1	−5.55	77.26
11	24	2	−10.83	109.48
4	20	−1	27.73	69.73
19	1	9	−24.83	89.93
6	4	5	205.88	82.93
25	−18	10	3.83	75.80
4	3	4	1181.95	122.02
4	0	4	1230.15	117.80
13	−16	−10	2.25	95.48
10	−14	−9	15.45	101.82
5	−9	−7	17.83	74.75
2	−9	−4	19.41	68.54
8	−18	−7	34.86	89.41
6	−20	−4	77.78	76.60
6	−21	−3	61.94	74.75
9	−26	−4	24.83	87.16
8	−25	−3	79.37	83.46
23	−43	3	54.81	91.25
12	−32	−1	3.83	99.71
17	−38	1	−107.63	92.71
15	−36	0	15.45	98.78

14	−35	−2	−19.15	106.31
17	−39	−1	38.43	90.73
12	26	1	−22.98	94.82
10	26	−1	38.43	100.50
7	15	4	−2.25	82.01
10	10	6	6.87	82.54
8	12	5	52.43	85.05
23	−8	10	14.39	90.86
5	10	4	16.11	87.82
1	13	−3	160.06	47.81
18	−6	9	0.26	78.31
14	−3	8	103.14	82.01
8	−1	6	54.28	63.65
9	−9	6	14.13	48.47
5	−13	−6	16.90	75.14
9	−20	−7	12.94	110.67
12	−26	−7	89.93	113.70
14	−29	4	−5.94	73.16
7	−22	−4	112.25	84.12
8	−24	−4	10.83	88.61
7	−23	−3	162.57	77.26
22	−43	2	−81.48	96.67
12	27	0	66.03	94.82
12	25	2	28.26	94.56
5	18	2	−0.92	77.39
3	18	−3	127.84	58.77
15	4	8	89.80	94.16
23	−5	10	−17.04	94.03
18	−3	9	−0.26	84.52
1	14	−1	239.56	63.65
3	9	3	362.77	83.46
8	2	6	50.58	67.22
14	−6	8	−7.13	70.26
9	−16	−8	−23.37	122.02
3	−14	−1	1451.35	138.40
10	−22	−7	31.69	107.23
14	−31	3	17.04	89.41
13	−31	2	0.00	67.22
11	−29	1	19.81	69.33
19	−38	3	8.19	84.39
19	−41	0	−0.13	91.39
11	27	−1	34.86	94.69
9	21	3	−9.38	107.37
8	20	3	−64.45	85.18
3	18	0	98.52	73.03
4	13	3	37.11	90.59
11	4	7	7.26	77.78
14	0	8	245.77	110.01
23	−11	10	59.16	78.44
8	−4	−9	48.73	104.33
17	−18	8	−4.75	55.20
2	−5	−5	416.39	81.75
11	−16	−9	4.49	97.86
2	−11	−2	788.80	92.44

19	−34	5	0.00	64.18
26	−46	3	15.45	93.90
18	−38	2	28.66	85.44
13	27	1	33.15	98.65
13	26	2	29.98	89.80
11	23	3	−36.32	111.99
8	25	−2	15.32	105.91
7	24	−2	82.54	96.40
6	23	−2	43.71	76.73
17	9	8	87.82	91.91
4	6	4	109.35	74.09
24	−16	10	−8.45	75.14
19	−14	9	55.07	69.86
10	−18	−8	−8.98	117.93
2	−10	−3	449.40	78.71
2	−10	0	5.55	41.07
25	−36	7	44.77	54.01
17	−28	6	12.15	43.45
11	−30	−1	7.92	81.22
13	−33	−2	24.17	109.61
25	−46	2	−97.73	101.03
14	17	6	66.03	99.97
13	16	6	−27.60	100.10
5	22	−2	−12.15	71.31
7	19	3	70.52	79.90
16	8	8	6.74	99.18
3	19	−1	180.79	67.88
23	−2	10	−48.73	96.67
12	8	7	45.96	108.16
6	14	4	47.94	79.10
18	0	9	−12.02	88.48
9	9	6	110.67	80.03
6	7	5	136.95	79.24
8	−4	6	71.31	58.37
4	−1	−7	148.44	67.09
9	−9	−9	−28.00	112.38
13	−15	7	60.35	46.62
7	−9	−8	41.07	82.14
0	−2	−1	1274.92	109.48
12	−18	−9	−18.22	93.37
11	−20	−8	−37.11	107.37
23	−39	5	16.11	73.56
13	−29	−6	9.77	103.01
23	−41	4	39.09	85.84
14	−34	−3	9.51	108.82
14	−34	0	−31.96	112.38
13	28	0	71.18	94.16
12	24	3	28.13	96.54
9	26	−2	−56.92	99.71
13	20	5	−12.28	95.35
12	19	5	23.51	108.55
11	18	5	10.96	119.25
12	15	6	0.00	115.16
19	4	9	30.51	93.10

4	17	2	222.65	87.95
7	11	5	214.07	82.54
14	3	8	51.50	100.50
4	5	−7	36.32	52.43
6	−11	−7	31.43	74.88
1	−7	−1	236.26	53.22
13	−20	−9	−64.45	95.08
12	−22	−8	60.88	109.87
5	−17	1	274.82	68.67
19	−36	4	59.30	75.14
10	−27	1	21.26	68.94
14	−32	−5	−20.07	105.25
16	−36	1	87.95	98.52
16	−37	−1	33.94	99.97
12	28	−1	0.00	84.78
15	14	7	−6.07	94.03
10	17	5	−61.54	116.61
6	18	3	72.63	83.20
22	−6	10	−6.74	94.16
11	−8	7	147.38	60.88
6	−5	5	251.31	63.13
5	−8	4	151.74	36.71
26	−40	6	−2.64	70.39
13	−27	4	7.26	58.11
12	−27	−6	−93.24	121.36
12	−29	2	−6.60	68.41
14	−33	−4	40.41	108.42
20	−41	1	−55.07	94.29
14	13	7	−18.88	98.78
11	14	6	28.92	112.91
4	21	−2	−15.45	67.88
15	7	8	0.00	93.63
2	12	2	145.40	73.69
22	−9	10	58.11	82.14
23	−14	10	8.32	75.27
0	9	0	1317.57	119.65
4	2	−7	25.09	57.71
14	−9	8	3.30	68.54
4	−3	4	451.38	60.35
4	−8	−6	9.24	71.05
3	−11	−4	17.43	75.41
20	−29	7	10.17	25.49
4	−16	−1	586.09	90.99
13	−29	3	36.58	69.73
9	−25	1	−16.90	68.67
18	−39	0	2.38	87.82
17	−38	−2	0.00	93.37
9	16	5	−21.66	88.74
18	3	9	26.68	91.91
22	−3	10	78.71	98.52
2	17	0	111.99	79.63
11	7	7	127.04	99.44
17	−4	9	36.18	85.44
1	12	1	332.40	67.48

6	−1	−8	130.61	72.90
13	−13	−10	45.16	81.88
3	−3	−6	689.75	92.18
11	−16	6	23.64	30.64
2	−8	1	1195.68	104.72
8	−17	4	12.28	33.15
8	−19	−6	53.88	79.10
11	−25	−6	172.47	116.61
13	−30	−5	6.07	120.57
26	−44	4	−89.67	89.41
13	−32	−3	−41.60	124.93
11	22	4	20.07	102.74
10	21	4	36.58	122.42
9	20	4	−17.83	113.44
13	12	7	37.11	99.97
10	13	6	43.05	95.08
5	17	3	93.50	87.16
17	−1	9	8.98	86.90
1	15	−2	294.63	66.03
4	9	4	158.61	88.48
10	0	7	88.35	69.46
18	−12	9	−23.37	71.31
1	5	2	1535.87	139.98
10	−11	−9	66.03	109.74
5	−6	−7	31.43	69.73
8	−11	−8	22.19	88.22
7	−13	−7	93.76	80.03
3	−13	−2	158.74	68.01
9	−21	−6	−11.49	83.46
22	−35	6	53.75	60.22
10	−23	−6	−2.11	96.54
7	−21	1	449.93	86.50
8	−23	1	107.50	65.11
9	−26	−1	27.07	79.90
18	−36	3	−12.41	83.86
13	−31	−4	−142.36	121.89
22	−41	3	13.21	88.74
17	−36	2	15.45	89.54
13	−32	0	31.43	87.56
12	23	4	81.48	95.22
8	15	5	−36.58	79.90
2	18	−1	112.52	71.45
8	8	6	35.79	78.05
3	12	3	4.89	82.80
10	3	7	105.38	77.92
13	−1	8	6.34	104.59
0	11	−2	461.95	54.54
17	−7	9	15.98	77.92
6	2	−8	28.39	64.45
10	−3	7	126.51	68.41
1	−5	−3	345.21	59.96
5	−14	−5	2.77	74.75
3	−12	−3	388.13	78.58
5	−18	−1	−2.38	78.58

26	−42	5	−75.80	82.93
11	−27	2	4.36	66.16
8	−24	−1	−6.60	69.73
15	−35	−1	−120.44	107.23
23	−44	1	−88.61	102.88
8	25	1	54.15	118.46
7	24	1	25.62	94.16
8	19	4	30.11	89.27
14	6	8	63.13	102.48
3	16	2	99.57	84.65
2	16	1	160.98	84.25
3	9	−6	71.58	34.34
22	−12	10	20.73	76.86
13	−4	8	134.70	78.71
2	5	3	193.34	58.50
9	−6	−9	−135.36	110.54
14	−15	−10	−65.90	94.03
0	−1	−2	449.01	57.05
6	−15	3	74.61	43.05
12	−25	4	−29.71	50.98
6	−20	−1	143.95	82.93
7	−22	−1	125.33	72.63
12	−28	−5	44.64	112.91
12	−30	−3	177.62	97.73
11	−29	−2	29.32	79.24
19	−40	−1	−61.28	92.97
9	26	1	0.00	108.69
7	25	0	−59.82	109.61
6	24	0	20.34	87.82
6	23	1	34.34	85.31
5	21	−4	69.60	62.07
3	20	−2	32.09	66.43
17	2	9	−19.94	93.10
2	17	−3	61.67	52.16
13	2	8	28.26	107.50
1	2	2	421.01	59.43
7	−6	−8	−82.41	76.46
11	−13	−9	−17.96	102.61
1	−6	−2	1453.07	132.19
8	−15	−7	45.69	83.33
4	−13	−4	90.86	83.59
18	−32	5	−31.83	62.73
12	−27	3	73.16	62.60
12	−29	−4	13.21	102.22
25	−44	3	17.17	93.24
15	−34	1	0.13	108.16
16	−36	−2	40.94	106.04
21	−42	0	−13.07	96.27
9	25	2	29.05	112.65
8	26	0	7.00	118.19
8	24	2	40.81	119.65
7	23	2	27.07	98.39
5	23	0	31.43	74.48
16	11	8	−36.18	93.10

5	22	1	144.87	79.10
7	18	4	0.00	81.09
9	12	6	19.94	86.37
21	−4	10	91.52	96.27
2	15	−4	66.29	40.28
27	−11	11	−38.30	89.67
21	−7	10	−35.39	84.25
1	8	2	2084.98	184.62
5	3	5	100.89	84.65
2	2	3	693.58	76.20
7	−10	5	219.09	48.20
9	−13	−8	28.26	103.14
3	−7	−5	927.33	116.21
6	−16	−5	1.98	72.50
11	−26	−5	157.42	101.16
10	−27	−2	51.50	79.63
28	−47	3	−31.43	97.33
24	−44	2	−83.59	101.16
19	−39	1	−17.17	86.10
10	27	1	22.85	95.61
10	26	2	−9.77	98.65
9	27	0	37.11	97.33
6	22	2	75.14	84.52
15	10	8	−17.70	101.16
18	6	9	3.04	94.82
7	14	5	335.43	99.97
10	6	7	−32.35	83.73
7	1	6	36.71	65.50
15	−14	8	−34.34	61.41
2	−2	−5	340.06	73.03
1	−1	−4	132.06	50.45
12	−13	7	92.18	48.73
5	−10	−6	73.69	71.58
9	−17	−7	−2.51	89.54
7	−18	−5	281.03	83.86
4	−15	−2	223.18	78.18
10	−24	−5	128.10	89.80
10	−25	2	64.84	66.29
11	−28	−3	115.16	81.88
12	−30	0	0.79	78.05
17	−37	0	−58.90	98.12
13	5	8	55.86	102.48
5	6	5	57.18	81.48
2	8	3	219.88	66.56
12	−15	−9	27.20	103.93
10	−19	−7	2.51	98.39
11	−21	−7	71.18	119.25
4	−14	−3	144.21	80.56
6	−17	2	109.48	54.54
11	−23	4	31.83	49.39
8	−20	−5	74.35	89.93
9	−22	−5	83.33	84.52
11	−27	−4	95.08	90.46
22	−39	4	−54.41	81.88

14	−33	−1	−5.94	105.25
11	28	1	36.85	92.71
11	27	2	−28.39	97.99
10	28	0	37.24	94.42
12	18	6	−7.00	99.05
6	24	−3	4.09	79.24
4	22	0	46.88	81.35
4	21	1	176.70	86.90
6	17	4	−27.07	83.07
16	−2	9	117.40	88.88
21	−10	10	−16.38	78.31
13	−7	8	85.84	71.31
25	−21	10	3.04	68.80
3	0	−6	893.92	99.71
8	−7	6	−2.38	52.03
6	−8	−7	176.04	74.61
10	−15	−8	−5.68	115.69
3	−12	0	530.49	66.03
9	−19	4	148.04	41.20
11	−25	3	99.71	59.56
9	−23	2	92.71	60.62
22	−37	5	0.00	69.99
9	−25	−2	115.55	80.95
16	−34	2	−16.90	104.86
8	27	−1	−7.26	100.10
9	24	3	13.34	120.44
7	26	−1	66.16	113.04
6	25	−1	45.43	95.74
11	17	6	3.17	108.03
14	9	8	45.16	93.24
17	5	9	36.05	97.33
8	11	6	24.70	87.16
16	1	9	−0.92	88.08
1	16	0	87.56	78.84
4	12	4	191.09	87.03
16	−5	9	4.75	79.76
24	−19	10	−27.86	70.78
10	−6	7	99.31	60.35
0	3	−3	1117.90	97.59
10	−8	−9	5.81	115.16
13	−17	−9	12.94	93.50
14	−19	−9	41.20	97.99
11	−17	−8	33.68	121.63
10	−21	4	195.32	45.56
5	−17	−2	2.64	76.86
10	−25	−4	56.39	85.84
8	−23	−2	110.80	75.54
10	−26	−3	277.06	85.84
14	−32	1	56.79	88.22
20	−39	2	161.25	89.93
16	−35	−3	58.77	105.65
15	−34	−2	33.28	116.74
11	29	0	−74.22	101.69
10	25	3	−58.64	100.89

11	21	5	−21.39	96.93
10	20	5	0.00	113.84
7	22	3	−6.34	97.06
5	24	−1	32.75	79.37
13	15	7	−50.84	96.14
5	23	−3	−16.11	73.56
4	20	−4	29.05	56.26
26	−9	11	−9.11	97.33
2	15	2	68.54	93.10
7	7	6	416.39	88.74
0	13	−1	352.87	61.54
0	12	0	721.58	81.88
3	5	4	93.10	69.33
23	−17	10	0.92	72.24
7	−2	6	57.18	62.73
0	5	1	709.96	81.22
5	−3	−7	141.97	70.52
12	−19	−8	137.61	115.29
13	−21	−8	171.94	115.42
14	−23	−8	29.32	98.91
6	−17	−4	86.63	82.01
5	−16	−3	68.01	83.73
7	−19	2	197.30	66.69
8	−21	2	0.00	61.01
6	−19	−2	293.70	100.10
9	−23	−4	98.52	76.60
7	−21	−2	244.18	83.33
17	−34	3	2.64	89.14
11	−28	0	73.82	81.48
21	−39	3	18.22	86.10
16	−34	−4	4.62	99.44
22	−42	1	−55.99	100.37
19	−39	−2	−93.37	96.93
18	−38	−1	−19.68	91.91
11	26	3	12.41	94.69
9	28	−1	−10.83	91.39
9	19	5	−24.17	123.87
6	21	3	140.38	85.18
10	16	6	32.62	118.59
3	21	0	98.12	74.75
2	19	−2	30.90	61.80
6	13	5	144.74	92.05
20	−5	10	−55.33	92.31
5	9	5	126.51	76.46
12	1	8	31.69	105.25
0	8	1	88.88	47.28
20	−19	9	−8.45	58.64
21	−21	9	5.15	54.94
8	−8	−8	1.32	81.48
4	−5	−6	74.75	66.29
6	−12	−6	101.55	77.65
7	−19	−4	222.92	80.56
8	−21	−4	102.88	84.25
14	−28	−6	59.43	114.10

9	−24	−3	53.09	79.76
4	23	−1	45.56	73.03
12	14	7	114.89	115.29
3	20	1	48.60	75.41
13	8	8	−14.39	100.10
5	16	4	0.00	79.37
20	−2	10	15.32	97.20
3	15	3	61.80	84.78
1	17	−1	265.57	79.63
1	15	1	134.31	75.27
1	11	2	218.56	67.62
9	2	7	98.78	72.24
3	2	4	498.66	71.58
7	−3	−8	336.10	82.54
7	−17	3	333.59	57.31
6	−18	−3	−30.24	96.14
10	−23	3	42.52	55.86
8	−22	−3	27.73	85.97
15	−31	−5	−90.33	106.84
25	−42	4	−25.88	84.52
13	−31	−1	17.96	82.41
16	−35	0	3.43	107.37
25	−45	1	0.00	101.03
20	−40	0	−10.04	89.41
12	30	0	0.00	97.33
10	29	−1	−21.00	85.31
8	18	5	−10.96	106.18
4	22	−3	39.22	67.09
9	15	6	5.41	106.71
16	4	9	10.83	89.01
12	4	8	151.47	108.16
26	−12	11	−34.47	88.08
2	11	3	5.41	73.43
3	8	4	37.37	80.16
9	−1	7	134.83	67.09
22	−15	10	54.94	74.88
19	−17	9	−5.94	61.67
14	−12	8	−49.26	65.37
1	−5	0	825.65	77.92
2	−9	−1	1058.73	103.40
13	−26	−6	61.01	118.72
7	−20	−3	40.67	78.44
10	−26	0	74.09	74.61
28	−45	4	−26.94	92.84
15	−33	−3	82.67	114.36
18	−37	1	−22.19	94.16
5	20	3	85.97	87.16
20	1	10	−7.13	94.03
9	5	7	−0.40	72.77
20	−8	10	−24.43	81.35
16	−8	9	0.00	75.80
3	3	−6	198.09	54.94
11	−10	−9	173.00	107.63
22	−23	9	15.98	50.05

7	−10	−7	381.92	89.80
7	−14	−6	398.69	89.41
9	−21	3	16.38	49.79
25	−38	6	10.43	68.94
15	−32	−4	67.88	126.25
23	−42	2	−42.00	97.99
21	−41	−1	41.20	95.35
10	24	4	49.79	102.22
9	23	4	−75.41	117.67
8	22	4	−7.53	113.18
3	22	−1	28.39	73.29
11	13	7	−42.00	108.29
3	19	2	15.19	73.95
25	−7	11	40.81	103.01
7	10	6	276.27	91.39
2	−7	−3	227.94	62.20
4	−14	0	1174.29	114.50
8	−19	3	34.34	51.90
12	−24	−6	−91.25	111.86
17	−32	4	41.60	85.31
15	−32	2	41.86	94.03
13	−30	1	−23.90	75.80
30	−48	3	0.00	100.63
24	−42	3	−3.43	92.31
23	−43	0	−23.24	98.25
14	12	8	24.43	101.29
7	17	5	39.22	93.50
15	0	9	−39.49	88.61
5	9	−7	10.96	39.75
3	6	−6	40.54	44.24
21	−13	10	8.06	73.95
18	−15	9	0.00	64.05
16	−16	−10	0.00	94.69
11	−11	7	232.56	57.58
9	−10	−8	−36.05	88.08
10	−14	6	196.24	40.15
11	−22	−6	−104.06	94.03
21	−33	6	48.07	56.52
25	−40	5	−40.41	78.31
14	−29	−5	−15.32	115.95
27	−45	3	30.11	96.40
26	−45	2	30.24	95.61
18	−37	−2	71.71	99.31
17	−36	−1	0.00	101.29
7	27	−2	0.00	101.95
6	26	−2	−56.65	109.08
7	21	4	24.70	110.27
16	7	9	146.59	96.93
4	19	3	98.25	86.10
8	14	6	96.40	102.48
4	18	−5	11.89	45.30
2	20	0	54.41	70.78
2	19	1	−27.60	74.22
12	7	8	10.83	102.61

4	15	4	78.97	77.39
9	8	7	27.60	91.12
5	12	5	156.10	82.80
25	−10	11	8.19	85.84
15	−3	9	44.64	84.92
0	11	1	1399.05	132.85
12	−5	8	52.96	70.52
10	−5	−9	132.99	109.21
5	0	−7	155.96	68.14
2	1	−5	48.20	59.30
5	−3	5	490.08	77.78
2	−1	3	282.21	45.30
3	−8	−4	100.50	73.43
10	−20	−6	118.33	89.41
10	12	7	52.16	114.50
15	3	9	94.82	88.48
19	−3	10	−2.51	94.16
3	11	4	280.37	95.35
7	0	−8	278.38	75.67
12	−12	−9	88.88	103.80
8	−12	−7	136.42	77.12
2	−8	−2	1303.57	122.68
24	−34	7	−21.79	52.43
28	−43	5	−22.45	84.39
21	−37	4	13.07	78.05
16	−32	3	0.13	93.76
14	−30	−4	1.06	118.33
14	−31	−3	0.00	109.48
19	−37	2	40.01	86.63
15	−33	0	−34.86	109.87
5	25	−2	8.06	90.99
6	20	4	22.98	91.12
13	11	8	21.00	98.39
3	21	−3	92.71	65.50
6	−5	−7	12.94	71.05
5	−16	0	523.62	78.05
13	−27	−5	114.23	106.71
8	−22	0	167.45	75.01
13	−30	−2	28.79	85.84
21	−40	1	−25.09	91.39
19	−38	0	−13.34	89.14
7	27	1	15.05	109.48
6	26	1	69.99	119.52
11	20	6	0.00	97.99
6	16	5	127.04	83.07
3	19	−4	102.88	55.33
2	14	3	87.03	94.16
1	14	2	94.95	79.50
19	−6	10	7.00	81.09
20	−11	10	3.70	75.54
6	3	6	58.64	69.86
4	5	5	263.20	87.03
9	−4	7	0.00	64.05
17	−13	9	8.45	66.16

13	−10	8	61.01	67.62
13	−14	−9	−3.83	100.10
3	−4	−5	443.86	81.48
10	−12	−8	38.69	103.01
5	−7	−6	−5.94	64.18
9	−14	−7	52.43	82.27
6	−18	0	349.83	80.56
7	−20	0	53.35	75.41
12	−28	1	−4.49	71.84
17	−35	1	75.41	105.65
8	28	1	50.18	98.25
8	27	2	−10.70	108.16
7	26	2	117.01	117.80
6	25	2	101.42	114.36
5	25	1	84.25	96.40
12	17	7	−1.58	94.69
10	19	6	−18.49	111.59
4	24	−2	98.65	77.12
2	21	−1	−1.85	69.33
15	6	9	−23.51	94.29
2	18	2	−24.96	76.60
1	18	−2	59.03	62.60
1	16	−3	30.37	52.82
24	−8	11	75.41	92.71
11	3	8	145.80	109.21
0	14	−2	1281.65	122.16
11	0	8	139.85	96.67
7	3	−8	150.95	68.14
15	−6	9	29.19	77.26
8	−5	−8	8.06	78.44
1	2	−4	150.81	44.77
7	−5	6	19.02	55.20
14	−16	−9	−16.24	104.46
12	−25	−5	0.66	89.80
21	−35	5	83.46	69.73
13	−28	−4	−58.77	92.05
11	−27	−1	0.00	72.77
24	−43	1	−4.49	92.31
20	−39	−1	11.09	92.71
9	29	1	24.43	94.56
9	28	2	42.92	92.18
10	23	5	16.51	95.61
5	19	4	8.06	85.18
12	10	8	35.13	100.89
3	18	3	249.33	87.16
7	13	6	88.35	82.54
4	8	5	136.29	80.95
3	−1	4	132.46	50.98
0	2	1	1145.50	99.18
15	−18	−9	174.19	97.59
11	−14	−8	130.87	114.76
16	−20	−9	40.41	94.03
10	−16	−7	67.88	82.54
6	−13	−5	136.02	82.27

12	−20	−7	61.80	112.38
3	−11	−1	331.47	62.73
13	−22	−7	13.47	124.40
14	−30	2	81.22	75.54
13	−29	−3	0.00	87.69
17	−35	−2	−30.11	116.08
16	−34	−1	3.57	116.74
7	28	0	−6.74	100.37
8	26	3	10.83	102.08
6	27	0	97.33	111.72
9	22	5	0.00	105.52
5	26	0	17.17	102.88
5	24	2	86.37	91.65
4	24	1	−9.64	79.24
11	16	7	72.90	106.44
9	18	6	18.49	112.78
11	6	8	66.43	110.93
6	9	6	85.84	86.76
25	−13	11	47.67	83.46
8	4	7	49.26	76.86
5	3	−7	52.69	56.52
6	0	6	119.12	63.26
4	2	5	44.77	72.50
11	−7	−9	97.59	113.97
4	−2	−6	152.40	65.11
1	−2	−3	161.77	44.77
4	−10	−4	151.74	76.99
15	−22	−8	51.11	105.65
11	−18	−7	−55.07	94.29
3	−10	1	1130.71	101.55
11	−23	−5	95.88	85.71
16	−29	−6	62.20	112.25
24	−40	4	0.00	87.56
12	−28	−2	72.50	84.39
22	−40	2	−62.07	94.03
22	−41	0	−26.41	95.08
10	30	1	74.35	103.93
8	29	0	−13.87	92.71
9	27	3	80.82	95.48
7	25	3	−11.23	116.61
8	21	5	−9.90	120.18
1	19	0	82.27	80.82
3	14	4	302.02	81.88
14	2	9	−1.45	88.08
14	−1	9	−36.98	88.74
8	1	7	51.11	72.24
12	−16	−8	59.96	129.95
13	−18	−8	−68.01	122.42
14	−20	−8	−17.04	110.01
3	−9	−3	237.31	61.28
7	−15	−5	195.58	84.12
10	−21	−5	213.67	88.08
17	−32	−5	−6.74	103.54
10	−25	−1	42.52	75.94

30	−46	4	3.57	101.55
14	−31	0	57.18	83.20
9	30	0	−51.50	94.82
4	25	0	90.20	84.25
4	23	2	−40.01	81.35
3	23	−2	157.81	71.84
10	15	7	−11.49	105.65
8	17	6	−0.13	117.93
5	15	5	57.97	84.65
0	16	−1	304.66	75.67
8	7	7	70.12	84.25
4	11	5	95.74	75.54
18	−4	10	12.02	91.12
0	14	1	1350.59	137.61
5	6	−7	45.30	50.58
19	−9	10	28.53	78.18
11	−3	8	43.98	75.27
1	7	3	197.70	60.35
16	−11	9	14.53	73.56
10	−9	7	18.49	53.09
2	−3	−4	192.68	54.94
8	−17	−5	69.46	73.69
9	−19	−5	29.71	77.65
16	−30	4	0.00	90.59
12	−26	−4	146.72	84.39
12	−27	−3	33.68	81.09
11	−26	1	121.50	72.50
27	−43	4	−17.04	85.05
23	−40	3	0.00	89.41
17	−34	−3	−7.13	109.48
28	−46	2	94.95	101.03
7	20	5	1.32	118.59
3	23	1	28.53	80.56
15	9	9	0.00	93.37
4	18	4	39.22	84.52
18	2	10	−5.68	97.33
14	5	9	9.11	89.54
23	−6	11	27.86	98.78
24	−11	11	40.15	85.84
1	10	3	215.66	74.35
7	−7	−7	411.37	85.44
1	−1	2	429.73	50.84
6	−8	5	275.74	56.13
6	−9	−6	44.77	70.26
15	−27	−6	−73.29	125.06
15	−30	3	36.98	76.99
11	−26	−2	105.91	84.65
17	−33	−4	11.49	113.31
18	−35	2	−6.87	93.76
25	−43	2	−12.94	96.54
18	−36	0	31.56	101.69
10	31	0	−75.14	101.95
5	23	3	−35.26	96.54
11	9	8	4.89	99.71

6	12	6	24.17	79.90
12	-8	8	9.77	66.29
9	-7	-8	-7.66	82.67
3	-10	-2	3657.83	309.42
16	-28	5	15.19	72.50
11	-24	-4	-17.70	79.37
9	-23	-1	45.30	74.09
29	-46	3	0.00	98.12
26	-43	3	89.67	93.90
20	-38	1	-2.11	91.91
6	28	-1	-3.57	107.23
8	25	4	-38.17	101.69
3	24	0	-8.72	76.33
2	20	-3	-3.43	60.75
1	20	-1	104.20	66.82
14	-4	9	0.00	81.22
0	10	2	4721.84	396.18
2	7	4	38.43	75.80
0	7	2	7735.74	636.53
2	4	-5	131.00	54.15
12	-9	-9	8.19	106.97
9	-12	6	166.93	39.35
5	-12	-4	73.43	83.86
4	-13	-1	284.99	61.54
16	-30	-5	113.97	119.91
16	-33	1	-8.85	101.82
15	-32	-1	97.20	96.14
20	-38	-2	67.48	96.93
19	-37	-1	39.62	107.76
7	29	-1	22.19	99.71
5	27	-1	98.91	108.82
7	24	4	100.89	117.80
4	22	3	35.52	81.75
12	13	8	27.60	94.29
6	19	5	34.73	101.95
9	14	7	74.88	120.44
7	16	6	97.86	119.91
2	17	3	86.76	82.93
1	17	2	80.29	96.80
23	-9	11	2.38	82.93
2	10	4	45.43	85.71
8	-2	-8	59.03	76.07
1	4	3	336.10	62.73
4	-6	-5	155.30	78.58
3	-6	3	620.42	55.99
14	-25	-6	108.95	119.12
10	-22	-4	386.94	90.99
8	-21	-1	160.45	88.08
11	-25	-3	58.50	82.14
20	-35	4	-32.09	76.46
13	-28	2	-10.17	66.16
14	8	9	64.97	92.44
10	5	8	23.64	109.48
18	-7	10	155.96	82.93

8	−2	7	179.74	70.39
6	−2	−7	−21.66	68.94
7	−11	−6	360.26	85.84
4	−11	−3	1424.67	142.76
6	−14	−4	−18.62	84.65
5	−15	−1	224.64	65.50
24	−38	5	−59.16	76.60
10	−24	−2	131.53	80.03
10	−24	1	197.04	71.45
19	−35	3	−33.28	88.74
13	−29	0	30.24	77.26
4	26	−1	50.45	92.57
6	23	4	136.42	117.14
2	22	−2	0.00	66.82
2	22	1	201.00	85.44
22	−4	11	−64.05	101.42
17	1	10	−31.30	95.88
4	14	5	183.43	86.37
1	13	3	86.63	83.99
10	2	8	150.55	104.59
2	4	4	148.44	67.62
13	−11	−9	0.00	102.61
8	−9	−7	56.52	76.60
13	−23	−6	30.90	104.06
9	−20	−4	79.76	83.20
6	−17	−1	531.15	89.67
24	−36	6	2.38	65.63
7	−19	−1	384.43	86.37
10	−23	−3	103.01	81.48
16	−31	−4	−38.43	122.82
16	−32	−3	0.00	115.29
23	−41	1	47.67	94.82
21	−39	0	−19.68	90.59
2	23	0	6.21	77.92
11	12	8	61.94	96.67
3	17	4	67.48	88.08
13	4	9	−13.87	87.95
13	1	9	−17.04	85.84
5	8	6	69.07	73.82
0	13	2	486.64	83.33
0	6	−3	260.16	25.49
21	−16	10	17.56	68.01
22	−18	10	25.75	67.75
10	−9	−8	80.16	91.12
8	−13	−6	69.86	84.78
19	−27	7	211.69	26.81
4	−12	−2	50.18	59.43
7	−16	−4	42.66	86.37
8	−18	−4	127.97	78.84
15	−28	−5	−20.47	118.59
9	−22	−2	0.00	71.97
27	−41	5	57.18	82.14
22	−40	−1	−13.73	94.95
9	21	6	6.87	98.52

5	22	4	−43.58	105.78
3	21	3	107.37	86.50
5	18	5	9.11	84.12
17	4	10	41.20	99.05
8	13	7	−27.60	117.40
10	8	8	101.29	106.97
0	18	0	394.07	89.14
22	−7	11	37.11	86.37
2	13	4	98.39	89.41
−1	11	0	403.45	59.43
−1	8	0	883.75	86.50
20	−14	10	7.79	71.84
23	−20	10	−41.60	66.69
14	−13	−9	63.13	100.50
1	−3	−2	466.18	60.88
1	−4	−1	709.17	75.14
5	−13	−3	432.90	87.03
12	−21	−6	60.22	88.74
20	−31	6	1.19	55.86
9	−21	−3	−22.71	76.99
15	−31	−2	56.92	94.16
14	−30	−1	66.03	91.52
21	−38	2	39.88	86.24
10	18	7	10.70	96.93
6	15	6	30.11	91.12
13	7	9	−52.30	93.24
0	17	1	413.88	89.01
7	6	7	47.54	81.75
0	12	−3	769.26	76.86
10	−1	8	−6.07	75.80
6	−3	6	271.25	66.69
9	−7	7	54.94	57.71
5	−4	−6	32.09	64.71
15	−15	−9	−37.37	103.93
17	−19	−9	−10.30	92.84
9	−15	−6	170.49	86.37
4	−12	1	196.37	44.64
8	−20	−2	29.45	83.59
9	−22	1	83.99	72.90
14	−28	3	56.65	66.43
19	−36	−2	0.00	104.72
17	−34	0	14.26	111.20
24	−42	0	173.13	101.42
8	24	5	26.54	93.90
8	20	6	11.62	102.22
7	9	7	−38.56	89.14
5	11	6	103.14	86.10
3	10	5	86.10	77.12
17	−5	10	21.39	81.22
2	10	−5	83.20	16.11
13	−2	9	64.05	82.93
24	−14	11	−31.43	82.41
7	3	7	227.14	76.60
3	7	5	623.20	108.42

11	−6	8	24.83	67.88
4	1	−6	91.12	55.86
0	4	2	3265.21	274.03
11	−11	−8	241.28	102.74
16	−17	−9	4.36	99.57
9	−11	−7	93.37	78.71
17	−23	−8	5.41	96.54
15	−23	−7	−55.99	119.52
6	−15	−3	0.00	85.18
5	−14	−2	73.03	66.03
8	−19	−3	43.58	87.82
14	−26	−5	80.03	97.99
20	−33	5	13.07	67.62
15	−28	4	−31.43	69.20
15	−29	−4	43.32	108.16
12	−26	2	125.99	68.41
12	−27	0	44.24	76.07
17	−33	2	55.07	104.59
15	−31	1	61.41	78.58
19	−36	1	−3.83	96.14
18	−35	−1	−8.85	108.42
8	30	2	4.36	92.84
7	29	2	−80.56	94.69
6	28	2	49.65	103.14
9	17	7	−58.11	98.25
4	21	4	0.00	86.37
1	21	1	76.20	79.10
10	11	8	−19.02	100.76
2	18	−4	84.65	49.39
21	−2	11	−22.05	96.80
−1	12	−1	125.72	45.83
19	−12	10	6.07	63.92
14	−7	9	10.17	72.63
5	2	6	−8.98	67.62
3	−1	−5	6.74	61.94
5	−8	−5	143.81	80.82
16	−21	−8	−2.51	100.50
14	−21	−7	62.73	118.72
7	−17	−3	398.16	98.12
6	−16	−2	157.81	81.09
7	−18	−2	148.83	83.33
27	−39	6	−18.75	72.50
23	−38	4	−64.45	83.33
15	−30	−3	−7.26	94.56
31	−47	3	15.85	99.97
27	−44	2	−83.20	99.97
7	30	1	97.20	95.74
6	29	1	−36.18	108.42
5	28	1	98.25	115.95
5	27	2	30.37	116.74
7	23	5	15.32	106.31
4	25	−3	65.77	77.12
7	19	6	0.00	120.31
1	16	3	114.76	96.54

0	17	−2	428.14	80.43
23	−12	11	−7.26	80.16
8	1	−8	225.69	73.95
2	7	−5	77.26	41.73
12	−6	−9	93.37	108.69
24	−22	10	−7.26	61.80
12	−13	−8	27.34	109.61
10	−13	−7	−16.51	80.29
29	−44	4	0.00	88.35
19	−34	−4	−39.88	103.14
19	−35	−3	−0.13	102.61
8	31	1	83.20	96.01
7	28	3	51.50	94.95
6	27	3	89.93	116.21
4	27	1	49.79	113.31
4	26	2	50.98	105.52
11	15	8	41.86	96.80
2	24	−1	100.76	77.39
13	10	9	64.71	95.88
1	22	0	−11.75	72.77
2	20	3	178.41	84.12
4	17	5	122.55	81.61
16	3	10	0.00	94.82
21	−5	11	39.75	97.20
16	0	10	48.33	95.35
3	4	5	880.71	113.70
9	−4	−8	183.17	81.88
13	−15	−8	−2.91	118.33
14	−17	−8	16.38	116.21
15	−19	−8	57.05	108.95
11	−15	−7	8.06	82.14
12	−17	−7	0.00	94.95
13	−19	−7	0.00	104.06
17	−28	−6	−44.11	104.86
13	−24	−5	−9.51	88.22
26	−41	4	0.00	86.10
28	−44	3	−33.54	94.16
22	−39	−2	−35.39	97.33
5	28	−2	−28.92	106.18
5	26	3	−43.45	116.74
6	22	5	−0.40	113.57
1	21	−2	21.53	64.71
5	14	6	20.87	82.54
0	19	−1	215.13	73.95
2	16	4	127.04	83.33
3	13	5	20.21	75.80
0	16	2	405.30	95.22
−1	10	1	85.71	52.30
18	−10	10	−4.23	74.75
1	5	−4	677.34	67.62
7	−4	−7	442.54	87.03
8	−10	6	−8.58	43.32
2	−7	0	22.05	45.16
5	−14	1	3.43	46.49

15	−26	5	9.64	59.82
7	−18	1	563.11	85.97
18	−31	−5	39.09	124.93
14	−27	−4	29.19	88.88
14	−29	−2	102.35	85.05
25	−41	3	−63.26	92.97
20	−37	0	−11.23	99.05
6	30	0	−52.56	96.14
5	29	0	5.55	101.95
8	16	7	0.00	105.91
12	6	9	34.20	90.86
9	7	8	34.73	117.40
12	3	9	−24.43	97.33
9	4	8	12.28	103.14
22	−10	11	16.11	80.43
7	0	7	381.52	81.35
6	1	−7	127.31	64.97
2	−4	−3	798.04	83.46
6	−16	1	26.68	53.22
19	−33	4	111.99	81.88
14	−28	−3	23.51	82.01
22	−39	1	47.15	88.08
21	−38	−1	−59.03	95.35
7	31	0	22.71	92.05
4	27	−2	64.31	107.37
3	26	1	12.68	93.24
3	25	2	−0.13	92.57
6	18	6	0.00	113.18
3	20	4	−14.53	80.56
3	17	−5	66.69	39.35
16	−3	10	−7.79	81.88
−1	13	1	374.13	72.77
2	1	4	231.64	57.84
0	2	−2	110.54	30.11
6	−6	−6	98.52	68.01
16	−26	−6	56.52	132.59
12	−22	−5	132.59	83.07
16	−32	0	20.47	87.29
25	−42	1	2.91	99.05
8	32	0	−21.13	94.42
6	26	4	−48.20	103.14
4	28	0	−95.08	111.06
3	24	−3	120.84	70.65
5	21	5	−109.21	118.72
10	14	8	1.32	92.71
1	19	−3	13.07	57.18
9	10	8	6.07	105.12
12	0	9	38.69	87.03
9	1	8	49.26	85.58
13	−5	9	−22.19	76.07
10	−4	8	77.52	69.86
13	−8	−9	−15.58	104.99
5	−6	5	329.89	62.86
11	−24	2	82.93	63.65

18	−32	−4	9.77	119.38
14	−29	1	51.64	74.48
17	−33	−1	121.63	111.72
23	−40	0	−36.85	90.59
12	9	9	10.70	92.31
20	−3	11	21.13	96.01
−1	15	−1	200.86	60.35
21	−8	11	−79.50	87.03
17	−8	10	−2.38	76.46
8	−5	7	58.90	61.28
1	1	3	228.60	45.69
23	−32	7	−3.04	49.26
11	−20	−5	2.25	82.14
13	−25	−4	−17.43	84.92
13	−26	3	43.71	61.94
18	−33	−3	−24.17	121.10
20	−36	2	83.46	93.63
5	25	4	8.19	125.33
3	27	0	42.39	100.50
3	26	−2	85.71	89.93
2	25	1	−58.50	81.75
2	24	2	8.98	80.82
1	23	−1	−6.34	73.29
7	15	7	−0.13	119.38
0	20	1	109.87	76.07
3	16	5	173.92	94.42
1	14	−4	22.19	35.00
6	8	7	68.80	78.84
4	10	6	277.46	87.95
1	12	4	118.19	89.93
0	12	3	94.56	79.76
4	7	6	254.61	80.16
1	9	4	66.03	77.92
0	9	3	179.34	65.90
−1	7	1	230.84	54.28
10	−6	−8	51.11	83.20
14	−15	8	0.00	53.09
10	−18	−5	106.44	82.80
15	−24	−6	−30.64	120.57
17	−29	−5	35.66	121.89
23	−36	5	144.08	72.77
13	−27	−2	108.55	83.07
12	−26	−1	138.14	78.84
16	−31	2	−17.30	82.41
18	−34	1	−29.58	108.29
4	20	5	−22.32	96.01
5	17	6	−77.12	99.57
0	21	0	149.49	73.29
15	5	10	−26.28	92.84
1	19	3	54.67	79.63
15	2	10	−2.77	94.69
14	−10	−9	74.09	102.35
5	−1	6	−4.23	61.28
8	−6	−7	197.96	75.94

4	−7	−4	1422.03	139.98
8	−14	−5	−13.34	75.27
9	−16	−5	50.45	74.22
14	−26	4	47.67	57.58
13	−26	−3	−37.90	82.14
29	−45	2	61.67	100.76
21	−37	−2	−9.38	97.99
7	22	6	67.88	110.93
4	24	4	44.64	116.61
3	22	−4	40.28	60.75
9	13	8	−80.29	98.91
2	19	4	60.35	85.18
−1	17	0	−37.37	73.16
−1	16	1	188.85	80.29
15	−12	−9	21.00	103.93
16	−14	9	89.80	63.79
2	0	−4	593.35	71.97
17	−16	9	11.23	57.84
13	−13	8	3.96	55.99
7	−8	−6	46.88	69.86
17	−24	−7	0.00	111.72
12	−23	−4	115.55	89.41
29	−42	5	12.28	82.67
23	−39	2	−64.97	86.50
5	30	−1	−3.43	95.48
2	26	0	30.37	87.42
8	19	7	8.85	93.24
0	19	2	209.84	79.24
4	13	6	33.81	80.43
0	15	3	0.00	84.12
20	−6	11	−22.19	82.93
16	−6	10	8.06	78.05
4	4	−6	81.75	50.32
1	6	4	138.80	70.92
16	−14	−9	2.38	95.61
17	−16	−9	6.21	94.03
18	−18	−9	−23.11	99.71
14	−22	−6	19.28	93.90
26	−39	5	−31.17	76.33
17	−30	−4	1.19	117.14
31	−45	4	0.00	95.35
21	−36	−3	69.73	103.01
21	−36	3	64.05	87.82
17	−32	−2	0.00	88.48
26	−42	2	−79.90	97.73
19	−35	0	−38.56	107.37
4	29	−1	29.85	109.08
2	23	3	−7.00	89.93
1	24	1	21.66	80.95
11	8	9	31.17	90.20
19	−1	11	41.07	96.14
11	5	9	−27.86	91.12
1	15	4	43.71	80.29
0	15	−3	16.90	53.75

8	6	8	47.15	107.37
2	9	5	−26.68	81.22
12	−3	9	−3.83	76.73
9	−1	−8	0.00	75.01
15	−12	9	47.15	65.77
11	−8	−8	29.71	89.27
4	−3	−5	367.53	75.67
18	−18	9	82.01	55.86
7	−8	6	46.49	46.22
18	−22	−8	57.05	104.20
3	−8	2	756.31	66.03
19	−29	−6	−5.28	110.67
23	−34	6	−18.75	64.58
16	−27	−5	33.28	117.01
10	−22	2	53.62	60.75
12	−25	−2	188.72	79.63
22	−36	4	40.28	80.69
30	−45	3	21.53	96.54
15	−30	0	0.00	74.22
20	−36	−1	−25.49	107.76
6	25	5	−34.73	104.86
6	21	6	0.00	103.80
2	25	−2	−6.47	74.48
2	23	−3	12.15	65.24
1	23	2	−56.39	82.93
6	14	7	0.00	116.48
8	9	8	−1.72	109.61
2	12	5	91.91	76.46
11	2	9	43.98	91.12
−1	12	2	259.37	68.28
6	4	−7	113.84	56.39
6	2	7	328.44	78.58
9	−2	8	39.62	72.50
0	6	3	7.00	54.81
−1	6	−1	978.44	87.42
5	−1	−6	122.29	63.65
−1	5	0	849.94	79.76
12	−11	8	−8.32	61.80
16	−22	−7	71.05	123.87
13	−20	−6	29.45	77.39
11	−21	−4	125.33	86.37
20	−32	−5	0.00	105.38
12	−24	−3	−24.96	83.59
9	−21	0	180.53	83.59
11	−24	−1	−19.28	72.77
17	−31	−3	−41.86	111.46
13	−27	1	101.82	73.56
16	−31	−1	33.54	84.12
21	−37	1	29.58	92.18
3	23	4	−17.83	103.40
7	18	7	0.00	113.31
3	19	5	−4.89	84.52
−1	9	2	1697.12	155.04
9	−8	−7	88.48	75.41

8	−10	−6	15.85	71.45
17	−20	−8	−8.06	103.01
3	−6	−3	635.08	76.60
2	−5	−2	357.36	59.03
5	−9	−4	515.04	88.61
17	−31	3	38.03	90.99
27	−42	3	20.87	90.46
24	−39	3	0.00	87.82
23	−39	−1	0.00	92.71
6	31	2	−8.85	92.44
6	30	3	6.60	91.25
5	30	2	−72.11	97.06
3	28	−1	−60.09	110.01
5	24	5	64.45	121.50
1	25	0	69.99	75.27
11	11	9	−18.88	92.05
8	12	8	31.83	108.69
4	16	6	13.87	93.37
0	20	−2	148.30	62.60
14	4	10	−15.19	94.69
19	−4	11	−9.11	93.24
−1	15	2	40.94	79.37
8	3	8	−9.51	91.65
22	−13	11	−30.90	77.78
23	−15	11	35.00	75.14
2	6	5	214.34	82.01
7	−3	7	218.96	68.14
12	−10	−8	41.86	97.59
16	−18	−8	65.37	118.72
16	−19	8	1.45	43.18
2	−6	−1	705.47	80.82
15	−20	−7	−22.58	122.02
3	−9	0	427.35	59.69
10	−19	−4	147.25	78.31
12	−24	3	59.43	57.71
28	−42	4	−1.98	83.20
25	−39	4	−60.75	87.42
27	−43	1	6.60	98.39
24	−40	1	28.79	91.91
22	−38	0	74.48	91.91
6	32	1	18.62	94.69
5	31	1	78.44	92.57
5	29	3	29.98	98.65
4	29	2	11.89	104.06
5	20	6	52.43	123.74
0	22	−1	97.33	70.52
14	7	10	45.16	92.57
2	15	5	7.66	80.29
−1	18	−1	234.94	77.78
−1	13	−2	227.14	49.39
15	−4	10	22.05	77.92
7	−1	−7	−2.91	65.50
13	−12	−8	−17.30	107.50
15	−16	−8	39.09	114.36

9	−12	−6	72.63	82.67
10	−14	−6	246.95	88.88
11	−16	−6	167.32	84.92
18	−27	−6	−11.09	113.97
19	−29	6	13.87	53.75
15	−25	−5	−10.17	88.48
16	−28	−4	−6.34	89.14
18	−31	4	10.96	90.33
15	−29	2	43.45	73.03
20	−35	−2	22.05	110.93
19	−34	2	144.74	104.06
17	−32	1	19.41	87.56
25	−41	0	−3.96	95.88
4	30	1	−76.20	102.08
4	28	3	8.45	104.33
1	22	3	−52.03	87.16
18	1	11	−42.39	96.80
1	18	4	38.43	83.99
0	18	3	235.33	83.07
14	1	10	−10.04	91.78
21	−11	11	−28.26	78.58
11	−9	8	−3.43	63.79
14	−14	−8	152.66	121.76
10	−10	−7	268.35	82.27
14	−18	−7	159.93	99.31
6	−11	−4	215.39	91.12
9	−17	−4	57.05	80.82
14	−24	5	48.07	48.33
29	−40	6	−15.85	76.60
11	−22	−3	28.79	77.52
9	−20	2	65.24	70.78
8	−19	0	296.74	82.27
11	−23	−2	200.47	75.27
10	−22	−1	96.67	82.80
20	−33	−4	36.18	102.61
16	−30	−2	95.88	85.58
3	28	2	31.56	114.89
2	27	−1	30.51	89.14
4	23	5	66.03	124.40
2	22	4	0.00	91.78
6	17	7	−7.53	112.65
0	23	1	210.77	85.84
11	−1	9	40.67	80.16
−1	10	−2	93.10	32.49
3	2	−5	482.95	70.78
11	−12	−7	49.65	74.88
13	−16	−7	−11.49	83.46
7	−13	−4	172.74	90.46
8	−15	−4	126.25	94.82
26	−35	7	41.34	61.54
26	−37	6	−11.36	69.99
19	−30	−5	58.37	112.25
16	−29	−3	128.76	89.67
20	−34	−3	−30.77	109.74

3	29	1	0.00	114.10
3	27	3	−60.75	113.57
8	15	8	−102.88	97.59
−1	20	0	59.43	75.80
10	7	9	60.48	92.31
18	−2	11	72.11	92.44
3	12	6	63.92	80.69
5	7	7	185.55	79.24
3	9	6	240.62	82.93
20	−9	11	31.17	77.92
13	−8	9	104.46	75.67
19	−20	9	−55.07	47.94
12	−14	−7	9.90	86.10
14	−23	−5	10.17	81.09
14	−28	0	125.99	80.56
23	−38	−2	−27.47	97.46
19	−34	−1	49.39	115.16
2	27	2	116.48	110.80
1	24	−2	8.58	69.33
5	13	7	0.00	98.12
−1	18	2	51.90	89.14
10	4	9	80.43	95.35
6	7	−7	−10.43	49.52
14	−7	−9	171.42	103.93
18	−13	10	−20.87	68.01
4	1	6	51.11	64.71
19	−15	10	0.00	64.97
5	−5	−5	758.16	102.22
4	−4	5	988.61	102.74
17	−25	−6	38.43	122.95
10	−20	−3	−25.62	85.18
7	−17	0	798.57	98.78
13	−24	4	38.96	52.16
33	−46	4	59.30	100.76
12	−25	1	43.05	67.88
32	−46	3	−52.43	100.50
18	−33	0	128.63	103.93
6	33	0	0.00	97.06
5	32	0	13.73	94.03
4	27	4	31.03	104.06
2	28	1	58.11	110.54
0	24	0	91.78	76.86
4	19	6	58.11	111.72
10	10	9	57.31	90.73
2	18	5	−26.81	80.56
7	8	8	0.40	107.10
9	2	−8	9.11	68.41
4	7	−6	393.01	57.58
8	0	8	73.56	74.09
17	−11	10	−46.35	69.33
1	3	4	445.44	72.50
6	−6	6	272.18	58.50
4	−8	−3	319.72	66.69
19	−25	−7	85.05	104.59

4	−11	0	785.37	81.48
15	−26	−4	−38.43	88.35
10	−21	−2	262.54	85.18
28	−43	2	−117.67	104.20
22	−37	2	21.53	85.18
4	31	0	43.18	94.29
3	22	5	73.95	116.21
13	6	10	−16.64	100.23
7	11	8	0.00	107.89
3	15	6	4.23	87.03
−1	16	−2	288.69	66.16
19	−7	11	−13.47	80.56
0	11	4	344.02	92.18
15	−9	−9	149.23	99.71
20	−17	10	15.58	62.46
10	−7	8	92.05	64.97
6	−3	−6	282.48	69.99
5	−13	0	498.13	67.48
6	−15	0	207.20	61.01
9	−20	−1	264.78	89.14
31	−43	5	35.79	88.08
20	−34	3	34.20	90.07
25	−40	2	−107.10	97.59
22	−37	−1	21.53	107.63
20	−35	1	−40.67	106.71
3	30	0	50.71	107.76
1	26	−1	49.79	76.20
2	21	−4	54.01	54.28
1	22	−3	33.68	62.86
13	3	10	−95.61	94.29
0	14	4	403.18	93.90
7	5	8	39.62	100.76
5	4	7	99.84	74.48
12	−6	9	21.92	72.90
6	−1	7	21.39	66.29
2	3	5	306.65	81.22
19	−17	−9	−42.66	92.97
3	−7	−2	2069.40	181.45
13	−21	−5	58.90	81.88
18	−28	−5	71.71	118.06
8	−18	2	109.08	62.07
22	−34	5	−56.79	69.60
19	−31	−4	−73.95	120.70
15	−28	−2	0.00	79.37
25	−40	−1	−63.65	93.24
3	26	4	−84.78	113.84
5	23	6	−24.96	102.61
1	26	2	75.80	88.22
6	20	7	−6.47	96.80
10	13	9	−6.34	91.65
1	21	4	−2.38	81.75
5	16	7	5.81	116.21
0	21	3	464.85	96.93
17	0	11	−27.73	93.63

-1	14	3	-47.81	86.37
-1	11	3	123.87	67.88
16	-11	-9	-13.34	97.06
8	-3	-7	22.19	69.33
-1	6	2	1392.98	128.76
17	-13	-9	29.58	93.24
18	-15	-9	71.18	95.48
5	-10	-3	572.88	82.93
16	-23	-6	62.99	113.04
9	-19	-2	254.35	88.88
11	-22	3	64.71	53.62
15	-27	-3	42.39	81.09
19	-32	-3	-17.17	117.80
21	-36	0	0.00	100.50
2	29	0	32.88	110.40
1	27	1	76.46	90.73
7	14	8	65.77	97.99
0	18	-3	108.16	52.69
1	14	5	62.86	80.29
18	-5	11	-30.51	82.27
1	11	5	13.73	73.16
10	1	9	-51.11	91.52
-2	13	0	167.32	54.81
11	-5	-8	-36.58	85.71
3	-2	-4	134.83	50.84
6	-7	-5	93.63	78.97
19	-21	-8	-40.01	97.73
18	-23	-7	6.60	119.52
3	-8	-1	79.90	48.99
12	-19	-5	142.76	82.80
8	-16	-3	221.99	89.54
8	-18	-1	66.43	75.80
30	-43	4	-21.13	87.95
16	-29	3	-12.15	70.39
14	-27	2	33.81	68.80
29	-43	3	99.71	93.90
16	-30	1	120.84	75.54
27	-42	0	4.23	92.84
26	-41	1	-63.26	96.14
23	-38	1	51.90	89.14
3	29	-2	23.77	108.69
3	18	6	11.09	99.18
-1	22	1	122.16	78.58
-1	21	-1	24.17	64.31
0	17	4	209.05	84.92
13	0	10	19.68	84.12
-2	15	1	243.52	73.95
15	-7	10	0.00	75.01
0	8	4	12.02	76.86
0	1	2	357.62	53.22
6	-12	-3	313.64	76.20
7	-14	-3	1136.52	129.68
14	-24	-4	67.62	84.39
28	-40	5	0.00	78.58

22	−34	−4	30.51	103.01
14	−27	−1	39.35	79.37
13	−26	0	211.83	79.37
23	−37	3	0.00	81.75
22	−36	−2	8.72	97.86
18	−32	2	32.49	96.80
24	−39	0	58.90	89.67
4	26	5	0.00	104.06
2	25	4	−13.34	121.50
4	22	6	0.00	110.14
2	21	5	135.76	96.80
−1	21	2	−46.22	73.56
9	9	9	6.34	88.74
1	17	−4	65.77	44.11
−1	17	3	−15.85	85.58
−2	12	1	741.13	86.37
1	8	5	221.07	93.50
9	−5	8	146.85	67.88
0	3	3	1397.60	127.57
21	−19	10	22.19	57.58
18	−19	−8	38.69	107.37
10	−12	7	79.37	45.69
11	−14	7	110.80	41.73
15	−21	−6	−17.43	86.50
20	−28	−6	66.29	101.69
8	−17	−2	29.71	80.03
25	−37	5	−0.13	71.45
11	−23	1	158.74	69.46
26	−40	3	−8.32	92.18
18	−32	−1	−56.52	95.48
1	17	5	123.87	88.88
9	6	9	6.34	90.73
4	9	7	62.99	83.46
7	2	−7	84.39	61.01
11	−4	9	52.43	76.99
12	−7	−8	160.98	90.86
−1	4	1	841.76	86.50
7	−5	−6	8.58	64.97
7	−9	−5	31.56	83.73
17	−21	−7	0.00	119.91
4	−9	−2	1389.81	130.61
11	−17	−5	61.28	78.84
17	−26	−5	14.92	104.46
7	−16	−1	447.95	76.73
21	−31	−5	60.75	102.88
18	−29	−4	21.26	108.42
18	−29	5	−31.96	78.05
27	−40	4	−34.73	85.58
22	−35	−3	−35.00	101.69
17	−31	0	39.35	85.71
4	32	2	6.87	99.71
1	28	0	45.16	98.25
0	26	1	290.93	86.24
0	25	−1	10.56	71.05

0	25	2	-11.75	86.76
5	19	7	-41.47	102.35
0	23	-2	148.57	69.86
-1	23	0	102.61	78.97
9	12	9	7.40	85.84
12	8	10	38.83	94.42
16	2	11	1.19	93.63
12	5	10	54.81	92.71
17	-3	11	68.94	87.69
5	2	-6	68.80	56.13
9	-5	-7	53.35	70.52
17	-17	-8	128.76	111.99
9	-10	7	-7.26	50.32
10	-15	-5	17.43	75.01
4	-10	-1	1395.09	131.00
7	-15	-2	119.78	72.24
14	-25	-3	137.61	90.86
17	-29	4	-5.28	82.41
14	-26	-2	53.35	82.27
24	-37	4	0.00	80.29
30	-44	2	130.61	99.71
4	31	3	23.90	92.18
3	31	2	71.71	95.88
2	28	-2	-0.66	89.93
3	25	5	83.07	114.23
0	20	4	39.49	82.93
4	15	7	22.71	117.80
6	10	8	-68.28	112.65
2	14	6	-29.45	80.16
4	10	-6	185.28	39.35
2	11	6	178.02	85.18
14	-5	10	64.18	75.80
-1	8	3	98.65	58.90
3	3	6	143.02	69.07
13	-9	-8	82.01	100.10
5	-4	6	95.08	56.26
2	-4	3	678.00	64.05
16	-19	-7	11.89	117.67
5	-11	-2	431.71	70.52
4	-10	2	1049.89	92.84
6	-13	-2	120.44	64.71
5	-12	-1	604.84	77.78
6	-14	-1	167.59	62.20
13	-22	-4	165.21	82.67
18	-31	-2	-27.07	94.16
3	31	-1	66.82	94.82
3	30	3	86.24	103.67
1	24	4	105.78	96.01
0	24	3	-2.77	80.82
6	13	8	-10.30	103.80
-1	20	3	82.41	74.75
-1	19	-2	19.41	62.99
6	7	8	-59.16	111.46
9	3	9	147.51	95.22

−2	14	2	117.01	80.56
10	0	−8	320.38	82.27
−2	10	0	−27.20	42.79
14	−11	−8	225.56	111.72
15	−13	−8	62.73	113.70
16	−15	−8	0.00	113.84
3	−2	5	98.91	61.54
19	−26	−6	111.46	120.04
18	−30	−3	38.43	95.35
21	−35	−1	93.24	108.42
5	34	1	−8.72	97.33
4	33	1	85.84	94.29
2	30	2	−35.13	102.88
2	26	−3	−38.96	75.14
3	21	6	14.13	115.16
−2	19	0	64.58	84.25
−2	17	2	64.58	85.31
12	2	10	7.40	89.67
4	6	7	−3.57	73.82
5	1	7	−8.32	68.01
8	−7	−6	5.15	70.26
2	−2	4	423.92	57.97
13	−17	−6	14.53	79.24
22	−32	6	30.77	59.82
24	−38	−1	−15.85	91.25
21	−35	2	107.10	96.67
19	−33	1	113.97	105.25
3	32	1	0.00	89.93
2	29	3	93.90	113.84
1	20	5	31.43	83.99
2	17	6	−40.81	92.44
16	−1	11	44.11	89.93
−2	14	−1	695.43	81.09
2	8	6	−15.45	74.48
20	−12	11	9.24	71.84
10	−2	9	0.00	76.86
−2	9	1	1220.77	115.82
8	−3	8	−38.69	67.09
4	0	−5	1133.61	116.87
21	−18	−9	−23.11	97.99
10	−7	−7	262.93	79.90
8	−8	7	92.84	54.54
15	−17	−7	−49.26	103.80
12	−16	7	40.54	35.39
5	−12	2	224.90	41.47
16	−24	−5	−98.65	87.56
12	−20	−4	0.00	76.20
12	−22	4	196.77	55.33
10	−20	3	0.00	50.18
21	−32	−4	9.64	105.65
13	−25	−1	63.39	78.97
31	−44	3	0.00	92.18
24	−38	2	−52.16	87.82
20	−34	0	28.92	111.99

2	31	1	−25.36	106.57
2	30	−1	−35.13	109.21
0	27	0	118.85	79.50
2	24	5	0.00	113.18
6	16	8	−10.70	94.56
4	18	7	−33.15	109.74
19	−10	11	36.98	75.80
−2	11	2	926.54	103.40
21	−14	11	−40.67	72.50
14	−15	−7	−26.15	84.25
12	−15	−6	50.58	80.69
22	−30	7	−46.75	50.05
20	−29	−5	115.29	125.72
13	−22	5	44.37	40.54
17	−27	−4	40.94	91.91
13	−23	−3	21.26	87.16
32	−44	4	6.87	88.74
15	−28	1	13.73	72.11
28	−42	1	40.28	94.03
1	29	2	23.90	111.72
4	25	6	29.45	96.14
1	27	−2	12.55	86.76
5	22	7	−46.62	94.42
−1	25	1	−49.92	76.86
−1	24	2	−14.26	85.71
8	11	9	22.98	87.95
8	8	9	−46.62	99.44
−2	17	−1	195.98	70.65
18	−8	11	29.32	75.01
1	5	5	353.53	88.61
20	−16	−9	−57.05	90.99
11	−9	−7	32.49	72.90
12	−11	−7	−27.34	77.39
13	−13	−7	96.01	80.16
9	−9	−6	168.64	75.27
21	−22	−8	−57.97	93.50
10	−11	−6	−5.41	72.50
20	−22	9	10.30	37.24
11	−13	−6	161.11	81.75
20	−24	−7	41.60	109.35
17	−21	8	19.28	28.39
13	−24	−2	−68.14	76.60
21	−33	−3	−78.58	117.40
13	−25	2	87.16	67.75
26	−40	0	78.18	90.86
23	−37	0	84.12	91.91
22	−36	1	44.90	97.06
1	28	3	56.65	118.06
−2	21	1	5.68	79.90
11	7	10	0.00	92.57
−1	16	4	49.39	78.84
−1	13	4	219.22	95.08
6	4	8	−4.89	89.80
16	−8	−9	46.75	98.12

0	5	4	632.97	85.44
0	1	−1	947.01	78.97
18	−24	−6	67.62	119.25
28	−38	6	−47.28	72.63
24	−36	−3	−40.67	92.18
15	−27	3	50.18	64.97
24	−37	−2	−14.39	98.65
16	−29	0	20.73	75.54
25	−39	1	−1.58	86.24
4	34	0	100.50	95.61
1	30	1	−75.14	112.12
1	29	−1	−104.06	107.37
0	23	4	0.00	86.24
−1	24	−1	−33.68	71.45
8	14	9	−8.72	92.31
2	20	6	4.23	118.06
−2	20	2	−35.00	73.95
0	16	5	211.30	88.08
0	13	5	82.67	77.52
17	−6	11	−25.09	76.86
11	−2	−8	0.00	80.82
17	−10	−9	14.53	94.42
18	−12	−9	−0.26	91.91
19	−14	−9	83.20	91.91
22	−21	10	−31.83	53.22
7	−6	7	−33.68	58.77
18	−25	7	18.49	32.88
15	−22	−5	49.92	88.48
11	−18	−4	106.57	79.50
25	−35	6	17.30	65.50
17	−28	−3	121.50	86.10
17	−29	−2	101.03	81.09
28	−41	3	−50.05	92.31
3	33	0	75.27	97.99
3	24	6	73.29	97.33
1	23	5	−4.89	106.84
−1	23	3	65.11	83.46
0	21	−3	192.02	62.86
5	12	8	−57.05	116.21
3	14	7	81.48	99.84
15	1	11	−53.62	93.24
11	4	10	−72.37	89.80
8	5	9	43.32	91.78
9	0	9	22.19	81.48
1	8	−4	606.56	57.45
4	−2	6	113.44	61.94
20	−20	−8	14.92	97.73
25	−31	8	0.79	45.03
30	−41	5	86.50	81.75
3	28	5	23.90	96.27
0	28	2	219.35	108.82
4	21	7	−15.72	92.84
−2	22	0	105.65	71.84
−1	19	4	6.60	73.95

5	9	8	35.26	113.70
−2	16	3	62.86	95.22
0	10	5	64.31	83.73
−1	10	4	120.84	81.48
8	0	−7	79.90	65.63
7	−1	8	0.00	66.43
19	−22	−7	63.52	119.25
19	−27	−5	21.26	113.31
16	−25	−4	2.64	78.44
12	−21	−3	0.00	77.12
20	−30	−4	−91.91	125.33
21	−32	5	−18.62	67.62
11	−22	0	57.97	80.82
20	−32	4	0.00	84.65
22	−35	3	−79.63	89.01
20	−33	−1	0.13	107.76
2	32	0	−16.11	95.61
1	27	4	19.81	113.31
0	27	3	2.11	112.38
1	25	−3	8.06	66.95
−1	26	0	114.23	76.46
5	15	8	−59.56	102.88
3	17	7	−6.21	121.63
0	19	5	249.99	88.08
−2	20	−1	63.26	72.50
20	−4	12	1.85	89.93
16	−4	11	50.32	77.78
−2	13	3	12.94	74.61
3	5	−5	130.34	52.69
4	3	7	258.44	76.86
2	3	−4	424.31	57.05
17	−22	−6	56.79	99.57
10	−16	−4	255.14	87.29
29	−41	4	−24.56	84.39
0	29	1	48.99	105.91
−1	22	−2	−5.81	64.05
1	16	6	−7.92	85.97
−2	19	3	130.61	81.35
1	13	6	45.83	80.95
7	5	−7	185.41	58.50
3	8	7	43.84	80.69
6	0	−6	235.46	63.13
19	−18	−8	−99.97	101.95
21	−27	−6	−11.36	114.63
14	−20	−5	−0.13	87.82
12	−22	−2	56.26	70.92
33	−45	3	0.00	94.82
29	−42	2	16.90	96.40
26	−39	−1	−3.96	91.39
23	−36	−1	−33.41	98.65
1	31	0	39.09	106.71
2	27	5	−46.88	98.39
0	28	−1	62.33	87.29
10	9	10	30.77	92.05

14	3	11	−59.30	92.44
12	−4	−8	38.30	88.48
6	−4	7	0.00	59.43
18	−20	−7	22.19	120.04
9	−14	−4	269.14	104.46
28	−36	7	−26.02	65.37
23	−33	−4	14.79	97.73
34	−45	4	52.96	94.29
27	−38	5	−46.22	77.65
20	−31	−3	50.05	124.53
20	−32	−2	−20.21	114.89
16	−28	−1	52.16	81.09
19	−32	0	−75.27	88.22
18	−31	1	161.64	84.25
0	26	−2	147.25	72.90
2	23	6	−82.41	109.35
−2	24	1	96.27	79.24
1	20	−4	29.58	51.77
−1	22	4	22.71	82.54
7	13	9	−71.45	91.91
7	10	9	49.52	96.14
11	1	10	−12.02	84.65
5	6	8	44.90	91.39
1	10	6	70.78	77.26
−2	8	2	1250.75	118.19
5	−2	−5	512.13	84.12
18	−16	−8	0.13	108.42
13	−11	9	−27.86	66.95
6	−8	−4	588.60	90.33
8	−12	−4	248.54	87.82
25	−33	7	10.17	56.26
22	−30	−5	118.59	102.61
11	−19	−3	19.28	85.31
9	−18	3	−6.47	54.01
16	−26	−3	27.86	82.01
24	−35	5	−38.69	71.97
16	−27	4	17.17	64.45
26	−38	4	15.72	83.33
14	−26	1	289.61	76.86
28	−41	0	14.92	92.05
20	−33	2	36.05	104.59
2	32	3	11.09	88.48
0	26	4	64.84	118.19
−1	27	2	23.90	84.65
3	20	7	−87.42	101.42
0	22	5	−34.47	87.82
1	19	6	−22.19	95.61
10	6	10	7.79	85.84
15	−2	11	−29.98	83.07
8	2	9	14.79	93.50
15	−10	10	84.78	65.63
−1	5	3	340.06	60.48
22	−25	−7	81.88	99.05
7	−10	−4	1565.72	160.32

23	−34	−3	−28.39	102.35
12	−23	2	149.63	62.07
15	−27	0	2.25	75.94
26	−39	2	−19.55	88.48
2	33	2	−77.78	96.80
1	26	5	−5.81	111.72
−1	28	1	−25.36	86.63
−2	22	3	52.03	84.12
7	7	9	86.37	94.69
−3	17	1	745.88	100.76
−2	10	3	211.17	75.94
13	−6	−8	−15.72	93.63
9	−2	−7	41.20	67.75
6	1	8	52.96	71.84
14	−8	10	30.51	70.26
22	−17	−9	67.48	90.73
16	−12	10	−28.79	64.45
11	−7	9	34.47	70.26
17	−14	−8	40.01	113.97
14	−13	9	0.00	58.77
1	0	4	135.89	51.11
13	−18	−5	36.98	77.26
18	−25	−5	111.06	100.76
15	−23	−4	−1.58	77.92
19	−28	−4	−98.25	97.46
17	−27	5	42.79	75.94
11	−21	−1	383.37	96.93
16	−27	−2	24.70	78.84
27	−40	1	90.33	92.97
25	−38	0	88.74	90.99
23	−36	2	26.02	85.44
22	−35	0	−61.54	103.01
1	31	3	40.01	93.24
0	30	0	61.41	118.46
−3	14	1	1424.28	138.40
0	7	5	1228.83	138.53
14	−8	−8	222.13	104.06
16	−12	−8	−12.02	112.12
3	0	6	676.28	89.67
17	−18	−7	0.00	109.74
20	−25	−6	0.00	116.74
11	−20	−2	36.18	84.78
10	−20	0	180.13	78.84
30	−42	3	0.00	91.39
24	−37	1	−105.12	93.50
21	−34	1	35.00	106.97
3	35	1	−47.81	94.42
1	32	2	50.58	98.39
−2	25	0	−16.90	76.60
13	5	11	−23.64	92.44
4	14	8	139.98	105.91
2	16	7	83.07	110.54
4	11	8	−30.37	109.08
14	0	11	0.40	87.56

-3	16	2	86.76	81.61
5	5	-6	87.82	50.71
3	5	7	20.34	72.37
13	-6	10	16.11	70.92
21	-15	-9	-0.13	85.71
15	-10	-8	92.44	109.35
17	-14	10	3.96	59.82
1	0	-2	399.22	51.50
15	-18	-6	0.00	74.09
13	-20	6	11.36	22.85
10	-17	-3	335.57	94.56
11	-20	4	62.46	43.32
8	-17	1	52.16	61.80
18	-30	3	36.45	81.61
2	34	1	-6.07	90.59
1	22	6	3.43	115.55
-2	23	-1	44.24	70.12
-3	19	2	155.44	90.07
-3	18	0	168.77	78.97
-1	15	5	38.03	86.63
10	3	10	-61.80	85.31
-2	15	4	68.28	85.05
-1	7	4	143.95	71.31
10	-5	9	2.25	69.86
7	-2	-6	52.69	61.41
5	-2	7	118.99	66.16
22	-21	-8	73.29	98.25
16	-16	-7	7.79	96.01
12	-16	-5	203.64	80.82
32	-42	5	86.10	87.95
14	-25	3	290.40	69.86
19	-31	-1	25.22	90.46
16	-28	2	62.20	69.99
0	31	2	104.20	105.65
0	30	3	-10.43	105.65
2	26	6	0.00	100.63
3	23	7	112.12	97.86
-1	27	-1	-23.77	75.01
-1	25	4	20.60	98.52
9	11	10	71.45	91.25
-1	18	5	139.32	82.01
-2	18	4	17.04	75.27
2	10	7	42.13	75.27
12	-4	10	8.98	73.56
20	-13	-9	-12.94	88.35
6	-4	-5	77.52	73.82
-1	3	2	487.17	63.52
15	-15	9	17.83	52.30
3	-3	-3	1319.95	120.70
21	-23	-7	-41.20	112.65
21	-28	-5	92.84	117.40
22	-31	-4	-18.22	114.10
19	-29	-3	201.79	99.57
31	-42	4	0.00	89.01

31	−43	2	90.33	93.24
1	33	1	−35.52	89.41
0	25	5	−0.13	122.95
−2	26	2	2.77	77.52
2	19	7	−2.91	109.48
6	12	9	14.92	90.99
9	8	10	48.73	96.27
11	1	−8	78.97	73.16
7	4	9	0.00	90.33
−1	12	5	−24.96	80.43
−3	13	2	392.88	74.35
18	−9	−9	22.32	91.12
19	−11	−9	31.03	88.08
10	−4	−7	99.31	71.71
1	−2	3	230.84	39.22
14	−16	−6	100.23	84.39
11	−14	−5	148.04	77.92
23	−28	−6	−19.68	107.37
17	−23	−5	−53.62	89.14
14	−21	−4	216.32	84.12
30	−39	6	50.32	74.09
15	−24	−3	60.09	79.24
19	−30	−2	85.58	90.46
15	−26	−1	164.94	78.71
25	−37	−1	0.00	95.35
22	−34	−1	12.94	117.14
1	30	−2	104.86	104.99
−1	29	0	7.26	90.20
−2	27	1	20.60	78.97
0	24	−3	114.89	62.99
−2	25	3	35.26	79.63
6	15	9	1.06	96.01
−1	21	5	66.03	82.41
−2	21	4	57.84	86.76
0	18	6	−1.19	83.59
−3	21	0	125.59	76.33
0	15	6	22.85	75.94
19	−5	12	21.39	80.03
4	8	8	17.70	93.63
5	3	8	−18.49	75.01
9	−3	9	−56.79	73.95
1	2	5	19.94	66.43
21	−19	−8	−48.07	101.82
15	−14	−7	−18.88	79.90
19	−23	−6	125.46	111.99
9	−15	−3	99.18	81.88
10	−18	−2	68.94	85.44
10	−19	−1	391.03	90.86
27	−39	3	19.55	90.33
18	−30	0	9.64	74.48
1	32	−1	−13.60	94.42
0	32	1	−27.73	99.31
4	20	8	−0.13	88.61
−1	25	−2	39.49	66.56

−3	23	1	147.12	77.65
−2	18	−2	84.92	67.22
13	2	11	−65.90	89.80
6	9	9	5.41	87.03
−2	12	4	58.37	83.99
17	−9	11	15.85	69.20
18	−11	11	−19.28	67.22
11	−6	−7	63.13	71.18
−2	7	0	−8.58	47.28
14	−12	−7	0.00	76.60
7	−6	−5	35.26	87.82
18	−26	−4	−1.45	84.12
25	−35	−3	0.00	97.20
22	−32	−3	−114.63	125.59
15	−25	−2	−14.00	77.39
13	−24	1	26.81	64.84
21	−33	3	22.98	96.01
17	−29	1	66.56	73.82
1	29	5	−1.06	93.63
−1	30	2	−55.20	111.86
−1	29	3	52.16	114.10
1	25	6	−76.07	103.67
2	22	7	21.53	96.93
−1	17	−3	5.02	52.56
18	−3	12	−70.26	83.73
7	8	−7	67.48	49.52
−3	18	3	47.94	82.67
−2	15	−2	324.34	62.07
0	12	6	193.21	82.01
16	−7	11	7.66	72.50
12	−8	−7	97.46	74.61
8	−4	−6	64.58	66.29
2	2	6	51.24	65.50
13	−10	−7	84.92	74.75
18	−16	10	36.18	55.47
9	−8	8	74.22	60.22
10	−10	8	228.60	59.56
8	−8	−5	39.22	91.39
20	−21	−7	40.01	117.80
13	−14	−6	52.16	80.29
9	−10	−5	0.00	80.69
21	−30	6	−3.04	56.65
9	−18	0	94.29	69.46
25	−36	−2	71.71	101.16
22	−33	−2	59.30	108.42
29	−41	1	−60.88	97.33
27	−39	0	−98.52	88.08
24	−36	3	−27.60	82.67
2	35	0	8.72	95.74
0	21	6	157.02	113.97
9	5	10	−52.56	89.01
−3	15	3	146.59	86.24
8	3	−7	152.66	63.39
15	−5	11	26.94	75.54

-3	11	1	353.53	62.86
4	0	7	199.41	69.07
-2	6	1	713.66	82.80
20	-17	-8	65.50	106.31
8	-13	-3	357.89	85.97
13	-19	-4	205.22	77.78
7	-15	1	12.41	48.99
11	-21	2	168.25	65.77
32	-43	3	-23.37	90.59
28	-39	4	16.11	80.69
19	-30	4	-50.84	91.25
28	-40	2	23.64	91.39
19	-31	2	30.90	83.99
0	31	-1	-57.45	108.69
3	16	8	-19.28	98.78
-3	21	3	262.27	85.97
3	13	8	-19.02	113.57
2	7	7	65.90	76.60
8	-1	9	21.79	70.39
19	-13	11	18.62	66.03
9	-6	-6	24.17	67.35
8	-6	8	31.69	60.75
12	-12	-6	7.53	80.29
4	-5	-3	246.95	54.15
16	-21	-5	57.05	83.86
8	-16	3	245.11	53.62
27	-36	6	-38.56	68.54
29	-39	5	152.66	79.50
24	-36	0	58.50	95.88
21	-33	0	48.33	111.33
1	34	0	12.15	89.67
-1	31	1	15.05	108.55
0	29	-2	-2.11	99.31
-1	28	4	-45.96	116.35
-1	24	5	11.89	105.52
-2	24	4	-38.03	82.01
8	10	10	17.30	89.01
17	-1	12	-14.00	91.78
12	4	11	38.96	89.01
1	15	7	-33.94	93.90
6	6	9	-67.22	91.78
14	-3	11	78.18	76.86
12	-1	-8	16.24	88.08
10	0	10	26.02	72.90
-3	12	0	526.66	67.88
10	-8	-6	22.98	69.99
0	2	4	422.73	65.37
11	-10	-6	69.20	74.75
11	-12	8	85.97	50.84
22	-26	-6	-21.39	108.69
7	-11	-3	1295.65	131.93
20	-26	-5	-13.34	110.40
9	-16	-2	412.03	86.90
21	-29	-4	-36.71	111.99

14	−22	−3	13.60	78.31
12	−20	5	0.00	36.98
18	−27	−3	119.78	80.29
26	−38	1	−2.38	85.58
25	−37	2	62.60	87.03
20	−32	1	147.78	93.37
0	28	5	85.31	98.52
−2	28	0	−49.26	77.26
1	23	−4	10.83	54.67
−2	26	−1	−0.13	72.50
−3	25	2	103.14	80.29
−3	24	0	53.22	77.52
5	14	9	123.87	93.37
1	18	7	45.03	111.33
−2	21	−2	−3.43	57.97
−1	14	−3	181.19	47.01
4	5	8	97.06	82.54
−1	9	5	179.34	91.39
23	−16	−9	−25.49	89.54
19	−15	−8	0.00	103.93
7	−4	8	217.11	65.77
19	−19	−7	57.05	122.16
5	−7	−3	456.67	69.99
6	−9	−3	87.29	59.56
24	−32	−4	−0.13	101.29
9	−17	−1	6.34	72.77
34	−43	5	−13.73	88.88
24	−33	6	7.66	61.28
33	−43	4	−67.22	87.16
22	−34	2	0.00	97.06
1	35	2	112.12	88.08
−2	29	2	37.24	104.99
−2	28	3	−79.37	103.67
0	24	6	9.90	110.40
−1	20	−3	12.15	53.48
5	11	9	30.51	85.58
−3	19	−1	244.18	79.76
3	10	8	−51.11	106.57
0	9	6	−17.43	73.29
−2	7	3	193.21	60.48
3	1	−4	525.47	66.29
23	−24	−7	5.41	103.54
23	−29	−5	−23.64	106.04
17	−24	−4	45.69	82.01
18	−28	−2	9.11	82.27
14	−24	−1	94.56	77.26
27	−38	−1	−35.26	91.91
25	−36	4	−29.45	80.16
23	−35	1	152.00	102.08
0	33	0	−68.94	95.88
0	33	3	19.41	88.48
−3	26	1	144.61	78.44
1	21	7	−26.15	108.55
−3	24	3	179.87	86.90

8	7	10	51.50	85.97
−2	17	5	45.56	82.54
−3	16	−1	259.63	63.65
13	−1	11	62.46	78.18
13	−3	−8	−16.51	90.99
−3	12	3	459.04	85.44
22	−14	−9	−0.26	84.25
7	1	9	−76.07	77.26
20	−15	11	−31.56	64.45
18	−13	−8	50.71	110.14
16	−17	9	0.00	45.30
12	−17	−4	−5.02	78.05
8	−16	0	408.73	72.77
14	−23	−2	171.02	78.18
26	−36	5	53.35	72.11
15	−25	4	26.54	55.47
15	−26	2	46.75	67.48
0	34	2	−58.64	87.69
−1	30	−1	10.43	100.89
−2	20	5	28.92	83.33
−3	17	4	280.23	83.46
4	3	−5	1063.09	108.16
−2	9	4	89.67	77.52
−3	10	2	3811.28	322.89
3	2	7	122.68	70.52
0	4	5	1036.02	119.65
23	−20	−8	36.32	94.29
15	−19	−5	39.75	78.84
21	−30	−3	1.98	101.69
27	−37	−2	−15.72	88.88
23	−33	5	54.01	66.69
31	−42	1	−49.92	83.20
29	−40	3	83.33	87.16
24	−35	−1	−3.30	98.78
21	−32	−1	−34.34	101.55
17	−28	3	57.05	68.14
−2	30	1	90.99	101.29
−1	27	5	−14.39	103.80
−2	27	4	148.57	111.59
−1	20	6	−4.09	91.12
−3	22	−1	110.40	66.56
11	6	11	−40.28	85.18
−3	20	4	72.24	80.69
−1	17	6	−10.70	79.10
−2	14	5	94.16	71.58
9	2	10	−108.55	82.01
21	−12	−9	4.09	86.63
14	−5	−8	−68.28	98.39
17	−11	−8	47.94	109.08
1	4	6	122.68	68.14
6	−2	8	93.50	63.79
18	−17	−7	−40.81	102.61
21	−24	−6	46.62	113.97
19	−24	−5	−1.45	88.35

8	−14	−2	212.49	68.80
6	−13	1	52.16	43.45
32	−40	6	9.38	75.41
24	−33	−3	−21.26	106.04
34	−44	3	12.41	91.52
21	−31	−2	−28.66	98.78
13	−23	3	29.98	57.71
29	−40	0	12.28	86.50
17	−28	0	16.64	74.35
1	36	1	−18.88	85.84
−1	32	0	39.88	102.48
−1	32	3	22.71	92.44
−2	23	5	26.94	88.48
−4	18	2	161.11	85.97
12	1	11	116.08	85.84
5	8	9	100.63	91.39
−1	14	6	119.91	84.52
9	1	−7	19.15	62.86
6	3	−6	12.41	53.22
15	−7	−8	28.13	104.46
16	−9	−8	57.84	103.27
13	−20	−3	139.19	78.44
13	−23	0	0.00	69.46
12	−22	1	82.80	75.27
16	−27	1	3.30	72.77
−1	33	2	14.39	95.48
−1	28	−2	−10.83	82.54
1	24	7	16.77	93.10
−3	23	4	15.05	83.73
7	12	10	77.26	87.29
2	16	−5	15.05	20.73
2	15	8	−71.18	105.65
−4	21	2	43.84	71.18
−3	14	4	249.99	91.52
20	−10	−9	29.71	86.10
1	9	7	−36.58	76.99
19	−18	10	−20.07	48.47
22	−22	−7	−3.17	102.22
2	−2	−2	142.76	46.49
0	0	3	396.71	52.30
12	−14	8	26.02	60.35
20	−27	−4	−35.00	89.27
8	−15	−1	−3.57	65.77
17	−25	−3	57.71	81.48
17	−25	6	31.17	57.97
35	−44	4	0.00	88.88
0	35	1	24.70	87.69
−1	31	4	−13.73	102.22
0	27	−3	0.00	69.46
0	27	6	−14.92	93.37
−3	28	2	68.01	86.63
−3	27	0	4.36	72.63
−1	23	6	39.09	113.44
−2	24	−2	82.27	67.35

-4	22	1	-23.64	75.01
18	-6	12	-11.09	77.52
-4	16	1	464.46	86.24
3	7	8	25.75	85.18
6	3	9	6.60	91.91
22	-18	-8	-68.14	91.39
17	-15	-7	62.86	88.61
16	-17	-6	15.85	78.31
24	-27	-6	-29.45	101.95
14	-17	-5	131.14	87.95
16	-22	-4	172.74	85.44
30	-40	4	-46.62	82.54
26	-37	0	0.00	85.84
26	-37	3	53.62	79.63
4	16	9	0.00	87.29
4	13	9	-23.77	87.69
0	17	7	21.13	106.84
7	9	10	-3.83	88.35
2	12	8	-57.45	107.63
17	-4	12	-23.24	76.33
8	4	10	-15.98	79.90
-4	15	2	55.73	72.50
5	0	8	-17.70	66.29
22	-27	-5	-22.05	122.82
7	-12	-2	379.28	69.73
23	-30	-4	48.07	117.53
21	-28	7	17.30	46.62
10	-18	4	122.42	42.26
31	-40	5	22.98	76.86
16	-25	5	-2.64	55.73
17	-27	-1	-8.45	76.33
28	-39	1	-9.90	85.84
20	-31	0	-62.86	80.82
-2	31	3	0.00	107.10
-3	29	1	96.54	99.18
2	21	8	-22.58	89.54
-1	23	-3	9.38	58.11
0	20	7	74.48	107.23
16	-2	12	0.00	80.16
11	3	11	92.57	89.41
0	14	7	261.35	92.57
-2	12	-2	291.99	45.96
-2	11	5	-23.77	80.95
2	4	7	-6.21	71.05
-2	8	-1	1207.70	106.71
-1	4	4	495.23	72.37
25	-30	-5	0.13	103.54
7	-14	0	1650.37	150.42
13	-21	-2	-0.40	78.31
17	-26	-2	37.90	87.16
13	-22	-1	51.24	76.99
10	-19	2	234.14	70.65
27	-38	2	-7.79	79.76
23	-34	0	89.41	113.44

23	−34	3	−51.50	88.74
−1	34	1	−59.03	91.78
−2	32	2	−2.38	97.73
−2	31	0	−20.34	112.25
−2	29	−1	−56.79	85.05
−2	26	5	−91.91	110.14
−3	26	4	−19.41	94.42
−3	25	−1	−47.15	69.86
−4	20	0	−76.73	79.76
−4	17	3	54.81	87.82
−1	11	6	39.35	76.07
16	−13	−7	5.15	78.84
4	−1	−4	510.42	67.35
−2	5	2	309.15	55.47
21	−20	−7	1.58	112.91
10	−13	−4	375.32	93.63
18	−22	−5	3.04	78.71
12	−18	−3	46.62	89.41
5	−11	1	97.46	38.43
27	−34	7	25.49	59.96
18	−29	2	46.62	67.09
−1	30	5	37.51	93.10
−1	26	6	0.00	91.91
0	23	7	27.47	94.29
−4	25	1	244.97	82.80
−4	23	3	147.64	86.63
4	10	9	−20.87	89.01
−4	17	0	332.27	79.37
−3	13	−1	135.89	48.33
24	−15	−9	35.92	89.01
10	−1	−7	59.03	65.90
21	−16	−8	0.00	91.65
12	−7	10	0.00	67.75
15	−15	−6	141.31	78.18
13	−15	−5	114.76	77.92
6	−10	−2	935.92	99.71
26	−34	−3	−75.01	99.05
20	−28	−3	−4.89	82.41
32	−42	2	−0.40	93.10
31	−41	3	−36.58	90.99
26	−36	−1	5.15	92.31
25	−36	1	−28.00	88.35
19	−30	1	88.48	75.14
2	19	−5	−0.66	33.54
−4	23	0	201.53	72.37
−3	19	5	163.62	83.59
15	0	12	80.29	87.69
5	8	−6	85.58	43.98
5	5	9	27.34	91.12
11	−5	10	−25.49	67.48
4	2	8	15.32	67.62
−1	6	5	393.01	88.48
15	−11	−7	77.52	75.27
13	−9	10	7.66	63.92

7	−13	−1	22.45	55.07
23	−31	−3	0.00	123.74
24	−35	2	−24.56	88.08
22	−33	1	10.30	101.42
21	−32	2	56.52	98.12
−3	22	5	70.39	85.97
−2	19	6	−16.24	86.50
10	5	11	0.00	85.97
8	6	−7	98.12	54.94
7	6	10	45.56	81.48
2	9	8	62.60	95.22
7	1	−6	−13.07	58.24
−3	9	3	167.19	62.46
3	−4	−2	127.57	49.79
23	−25	−6	−21.39	117.67
9	−11	−4	791.44	112.12
5	−8	−2	955.86	96.67
15	−20	−4	38.43	83.73
19	−25	−4	−5.41	78.05
24	−31	7	−5.15	52.82
16	−23	−3	138.40	82.27
7	−14	3	1292.61	116.74
29	−37	6	−5.15	67.22
20	−29	−2	−54.41	84.92
27	−37	4	8.32	82.01
23	−33	−1	119.25	106.97
18	−28	4	−42.39	70.39
−2	33	1	111.72	101.69
−3	30	3	51.64	114.89
−4	27	2	−0.79	83.73
−4	26	3	120.70	79.63
−2	22	6	−0.53	104.06
1	17	8	10.04	94.82
6	11	10	5.02	90.73
−2	16	6	7.92	76.60
−3	16	5	−19.55	75.80
23	−13	−9	15.58	85.05
−4	14	3	421.27	90.59
14	−6	11	−41.73	70.52
11	−3	−7	87.16	66.69
−3	11	4	8.45	76.20
15	−8	11	1.72	67.75
20	−14	−8	45.03	97.86
5	1	−5	441.22	74.48
14	−9	−7	22.05	80.16
24	−23	−7	−84.39	100.76
14	−13	−6	−22.98	83.33
4	−6	−2	154.78	50.58
21	−26	8	14.00	25.09
23	−32	−2	30.37	114.76
12	−21	0	251.97	86.10
14	−24	2	204.56	66.43
−3	31	2	0.00	105.78
−2	29	5	−1.58	97.59

3	18	9	0.00	78.58
0	19	−4	124.67	48.33
3	15	9	35.92	91.91
−4	22	4	124.01	86.76
14	2	12	10.30	90.73
−4	19	4	50.45	80.95
13	−4	11	68.28	70.65
−4	13	1	253.03	63.65
1	6	7	14.66	71.18
13	−7	−7	23.77	72.24
14	−11	10	90.07	59.56
20	−18	−7	1.85	112.91
5	−3	−4	339.79	64.84
8	−9	−4	716.03	99.57
21	−25	−5	4.89	101.69
22	−28	−4	−70.52	108.29
32	−41	4	30.77	81.22
28	−37	5	−28.00	77.26
30	−40	1	0.00	88.08
28	−38	0	11.49	84.65
−1	33	−1	29.98	91.39
−3	30	0	46.75	91.12
−3	29	4	32.88	120.31
−2	27	−2	91.91	67.88
−3	25	5	−18.88	112.65
1	14	8	−34.20	107.63
22	−11	−9	18.49	84.52
12	−2	11	−8.19	71.71
19	−12	−8	86.24	107.63
16	−10	11	−51.11	67.62
24	−19	−8	−53.75	87.42
8	−4	9	8.98	67.22
9	−6	9	58.64	66.16
4	−5	6	123.34	50.84
5	−7	6	33.41	45.30
17	−20	−5	−7.66	82.01
24	−28	−5	−7.26	105.91
11	−16	−3	119.52	92.44
25	−31	−4	142.49	104.59
6	−12	0	300.04	54.41
12	−19	−2	317.87	95.48
33	−41	5	−21.92	78.18
15	−25	1	11.36	70.12
−1	36	2	64.18	89.01
−1	35	0	−45.03	83.20
−4	28	1	152.40	79.37
−2	25	6	58.50	111.99
−4	26	0	−15.45	72.77
−1	19	7	35.39	107.37
13	0	−8	1.45	93.37
−4	16	4	153.45	84.92
4	7	9	12.81	87.82
3	8	−5	98.78	37.51
−4	12	2	178.02	61.67

3	4	8	122.55	74.48
−3	8	1	114.50	52.03
13	−11	−6	127.57	75.54
10	−8	9	93.10	62.07
−1	2	3	229.52	46.62
6	−11	−1	111.99	54.81
4	−9	1	760.80	74.22
16	−24	−2	0.00	71.71
12	−20	−1	294.89	94.95
29	−39	2	3.17	86.63
21	−31	4	−48.33	89.14
16	−26	3	87.95	61.80
−2	34	3	60.48	87.03
−3	32	1	−128.23	111.46
−1	26	−3	−13.87	65.24
−3	28	−1	60.62	74.88
−1	22	7	34.07	99.71
−4	25	4	122.55	84.39
0	16	−4	166.79	32.49
3	12	9	0.00	86.76
−1	16	7	9.11	93.37
6	8	10	0.00	82.27
11	0	11	0.00	71.97
−2	13	6	44.90	85.18
8	1	10	14.39	74.22
8	−1	−6	69.60	59.69
7	−2	9	−9.38	69.73
1	4	−3	281.16	37.24
11	−11	−5	621.88	94.29
3	−3	6	713.53	86.50
19	−26	−3	116.48	81.09
33	−42	3	21.66	84.39
16	−25	−1	51.64	71.18
25	−35	0	−18.62	94.95
19	−29	0	113.18	81.61
−5	20	2	25.75	87.82
−3	13	5	23.37	72.50
−4	14	0	1220.64	118.85
18	−10	−8	−71.97	106.44
−1	8	6	−10.70	65.77
−2	6	4	212.75	67.35
19	−16	−7	159.00	112.38
23	−21	−7	18.62	93.90
24	−27	9	66.29	16.90
14	−18	−4	193.34	79.24
27	−32	8	0.00	48.47
26	−34	6	−2.25	63.52
28	−37	−1	−71.18	93.90
19	−28	5	−92.57	81.48
14	−23	4	118.19	55.07
12	−21	3	35.66	51.11
22	−32	0	35.52	93.76
−2	35	2	−51.37	91.91
−4	30	2	52.03	110.54

-3	28	5	67.22	101.82
-4	29	3	47.15	104.86
5	13	10	47.54	85.58
-3	20	-2	32.62	58.50
-5	21	1	12.02	75.94
14	-2	-8	42.39	94.82
1	11	8	-35.13	101.42
17	-8	-8	-62.20	104.72
23	-17	-8	41.73	87.16
-2	8	5	155.83	82.93
-3	9	0	333.72	54.54
17	-12	11	-8.98	61.28
6	-1	-5	436.86	79.37
12	-9	-6	206.68	74.22
15	-13	10	34.20	54.54
18	-18	-6	103.67	85.58
3	-5	5	492.06	66.69
6	-9	6	0.00	35.00
18	-23	-4	136.15	82.14
25	-32	-3	-41.20	106.18
22	-29	-3	40.81	93.90
28	-36	-2	-30.51	88.35
20	-28	6	4.49	49.39
25	-34	5	-53.35	66.69
27	-37	1	16.64	84.39
-2	34	0	-13.07	88.35
-2	32	-1	50.45	104.33
-2	28	6	-13.47	90.20
-1	25	7	30.90	88.08
0	22	-4	94.42	51.11
-4	21	-1	220.41	71.58
-5	22	3	153.59	87.95
10	2	11	-32.35	80.82
15	-4	-8	32.09	99.18
16	-6	-8	24.83	103.54
7	3	10	3.70	75.14
9	-3	-6	3.70	62.33
6	0	9	19.55	70.39
11	-7	-6	322.63	77.39
10	-9	-5	111.46	82.27
2	-1	6	149.49	60.75
5	-5	7	60.48	57.05
1	-1	-1	1008.55	86.10
25	-26	-6	45.56	95.61
16	-18	-5	56.26	80.16
20	-23	-5	-77.26	92.44
10	-14	-3	708.24	104.72
15	-21	-3	85.05	73.43
9	-17	2	49.52	53.48
32	-41	1	1.72	86.63
30	-39	0	41.34	88.48
-1	37	1	59.30	86.37
-3	33	3	263.07	94.69
-4	28	4	0.00	117.67

−5	26	2	42.00	81.61
−3	23	−2	−16.64	61.41
2	17	9	2.11	81.75
−5	24	1	−8.98	77.65
−4	21	5	65.63	78.97
−5	19	3	60.88	81.75
−5	18	1	34.73	80.95
15	−3	12	45.03	72.77
−5	17	2	−23.24	88.35
16	−5	12	−48.99	75.41
17	−7	12	68.54	72.77
2	6	8	24.70	80.69
0	8	7	52.96	76.73
10	−5	−6	84.92	65.63
7	−3	−5	1045.13	116.35
9	−7	−5	70.52	80.56
4	−3	7	155.57	59.30
6	−7	7	56.79	51.24
2	−3	5	75.67	56.65
4	−7	5	952.69	93.10
5	−9	−1	672.59	76.33
5	−10	0	−2.25	45.56
34	−42	4	31.83	79.90
25	−33	−2	−34.60	108.16
23	−31	6	−17.56	59.30
19	−27	−2	19.68	76.99
25	−34	−1	−0.53	108.16
19	−28	−1	14.00	73.56
26	−36	2	82.67	84.65
25	−35	3	−3.17	79.50
18	−28	1	−6.47	72.37
−3	34	2	−36.18	87.95
−3	32	4	−40.15	90.73
−4	29	0	0.00	77.26
0	22	8	18.09	84.65
−4	24	−1	−71.84	69.60
−5	25	3	108.95	80.69
−3	21	6	16.51	93.37
0	16	8	43.58	107.63
5	10	10	19.81	87.56
−3	18	6	18.36	74.48
−4	18	−1	678.79	89.54
3	9	9	49.26	89.14
25	−14	−9	−18.22	88.08
18	−14	−7	7.79	90.59
−3	7	2	2041.53	179.47
8	−5	−5	205.75	74.09
23	−26	−5	193.73	115.69
27	−32	−4	−24.83	86.63
24	−29	8	114.36	28.92
21	−26	−4	9.11	86.37
11	−17	−2	248.67	82.67
35	−42	5	6.60	80.82
22	−30	−2	−6.34	92.05

29	−38	4	0.00	78.05
24	−34	1	−85.58	99.18
19	−29	3	123.74	71.84
17	−27	2	58.11	66.95
−2	36	1	55.60	84.92
−4	31	1	17.96	103.01
−3	24	6	23.11	110.67
−4	24	5	−7.13	92.18
2	14	9	0.00	85.31
−3	17	−2	511.08	75.27
−4	18	5	124.67	80.82
14	−1	12	38.96	75.27
9	4	−7	−3.17	55.60
9	4	11	61.54	83.73
−4	13	4	156.49	86.24
22	−15	−8	64.45	94.95
5	2	9	−5.68	73.69
22	−19	−7	36.05	109.74
3	−1	7	21.92	59.43
26	−29	−5	46.75	92.84
13	−16	−4	48.20	80.16
3	−7	1	2523.82	209.84
24	−29	−4	34.47	118.59
31	−38	6	41.34	70.78
11	−19	0	300.44	79.37
31	−40	2	61.94	89.93
15	−24	0	−4.09	66.43
22	−32	3	−13.60	94.16
21	−31	1	31.83	82.01
−3	33	0	−18.49	95.48
−5	27	1	77.52	75.27
−2	21	7	85.97	106.71
13	1	12	4.09	80.82
6	5	10	0.79	78.71
−4	11	3	1579.05	152.53
1	1	6	349.43	71.84
17	−16	−6	5.81	76.07
1	−1	5	130.74	63.65
30	−38	5	−136.42	77.65
30	−39	3	9.11	83.46
23	−33	2	36.32	99.97
−2	30	−2	−20.34	96.01
−3	31	5	0.00	90.20
−4	32	3	61.80	100.50
−5	24	4	24.96	77.12
−5	22	0	201.00	72.77
−2	18	7	36.32	103.40
−5	21	4	59.82	78.58
−5	16	3	−21.26	87.69
−2	10	6	12.41	70.65
17	−12	−7	34.20	83.73
12	−12	9	49.79	54.01
17	−19	9	21.00	26.02
7	−9	7	46.75	45.03

4	−7	−1	2678.46	228.73
17	−21	−4	113.84	81.61
18	−24	−3	69.86	83.07
15	−22	−2	24.96	70.78
11	−18	−1	410.97	85.31
10	−18	1	64.05	65.77
27	−36	0	31.83	87.69
20	−30	2	27.34	75.67
−3	31	−1	82.14	101.03
−3	27	6	−6.74	94.69
−5	29	2	103.01	88.22
−4	27	5	85.97	106.57
−2	24	7	−70.92	94.56
0	13	8	−7.13	105.12
−3	15	6	101.55	77.92
21	−13	−8	0.00	99.97
4	4	9	102.35	94.56
18	−14	11	28.13	58.37
25	−22	−7	−15.58	96.67
−2	4	3	733.20	80.03
24	−24	−6	0.00	93.10
20	−20	10	225.03	34.73
15	−16	−5	96.27	77.65
19	−21	−5	31.69	78.44
9	−12	−3	500.25	85.71
29	−35	7	0.00	63.13
27	−33	−3	123.34	96.14
36	−43	4	2.51	84.25
15	−23	−1	52.56	68.41
29	−38	1	−12.28	81.88
−3	35	1	−20.21	84.78
−4	33	2	25.22	95.35
−3	26	−2	165.60	69.07
−4	27	−1	47.28	67.62
−2	22	−3	5.81	55.99
−5	25	0	95.48	73.03
−2	19	−3	59.43	49.13
12	3	12	0.00	89.41
8	6	11	53.88	87.03
−5	19	0	219.62	76.73
2	11	9	−3.96	87.29
1	8	8	56.65	83.20
−1	10	7	−47.81	72.37
25	−18	−8	−13.47	82.01
−3	10	5	336.49	89.93
−3	8	4	140.25	75.54
2	1	7	0.13	64.45
2	−3	−1	488.36	55.07
3	−5	−1	1188.29	114.10
14	−19	−3	105.65	84.65
14	−23	1	58.37	63.39
13	−22	2	14.92	59.96
−5	27	4	36.85	95.35
4	12	10	53.35	82.27

−2	15	7	20.60	87.95
5	7	10	30.77	82.01
16	−10	−7	−20.87	76.20
21	−17	−7	15.19	115.95
16	−14	−6	151.08	76.46
4	−8	0	356.56	54.81
24	−30	−3	32.49	112.52
21	−27	−3	−31.96	88.22
27	−35	−1	11.49	98.91
24	−33	0	−24.04	111.72
18	−27	0	50.18	72.50
1	19	9	0.00	80.95
−1	21	8	−27.34	95.88
10	2	−7	177.49	63.26
6	6	−6	200.20	52.16
−5	15	1	220.41	65.63
20	−11	−8	−25.09	101.95
−5	14	2	−1.58	70.26
0	3	6	777.31	96.93
16	−15	10	−16.51	49.26
12	−14	−4	−6.87	83.07
5	−9	5	196.77	41.20
10	−15	−2	383.77	87.95
17	−23	7	127.84	27.07
−4	32	0	−43.84	99.71
−4	30	5	0.00	98.25
−5	30	1	−11.49	87.56
1	16	9	0.00	82.27
1	3	7	113.44	66.69
5	−3	8	75.67	58.77
20	−19	−6	21.53	85.71
6	−5	8	78.58	56.65
25	−27	−5	0.00	111.86
22	−24	−5	−66.43	95.22
8	−10	−3	132.33	65.24
2	−5	1	1309.52	112.12
26	−30	−4	16.77	107.50
20	−24	−4	−27.47	78.18
18	−25	−2	141.70	77.65
11	−18	5	147.78	36.85
31	−39	4	−57.18	78.97
28	−37	2	26.41	79.37
21	−30	0	40.01	78.58
17	−26	4	2.25	57.05
−4	34	1	10.96	93.24
−5	31	3	0.00	106.97
−5	28	0	24.96	72.50
−4	23	6	−42.00	102.74
−5	23	5	−13.87	81.48
−4	20	6	47.81	90.20
−6	22	2	8.45	70.26
3	6	9	−32.49	90.20
24	−16	−8	−32.35	82.67
15	−8	−7	−1.58	71.71

-4	10	1	1508.27	137.48
4	-1	8	15.19	62.20
23	-27	-4	38.17	105.52
24	-31	-2	24.96	111.46
32	-40	3	54.01	85.84
24	-32	-1	20.21	108.16
18	-26	-1	-37.77	77.52
15	-23	5	24.96	47.94
27	-36	3	-81.35	80.03
26	-35	1	-1.98	87.16
-3	37	2	151.34	89.93
-5	32	2	6.47	105.38
-5	30	4	56.39	113.70
-2	25	-3	11.75	59.03
-4	26	6	-18.22	99.71
-6	24	3	164.15	82.01
-5	20	5	43.84	82.01
-6	21	3	46.75	79.24
-1	15	8	-78.71	110.54
11	0	-7	275.08	70.65
-4	15	-1	221.33	57.18
19	-9	-8	-64.05	101.55
0	10	8	166.40	95.22
11	-3	11	19.15	68.14
12	-5	11	14.00	66.43
24	-20	-7	-11.09	103.54
15	-12	-6	38.69	75.94
7	-7	8	230.97	59.82
16	-19	-4	233.75	80.29
7	-11	6	119.12	34.60
33	-39	6	32.62	73.29
28	-35	6	-68.01	67.75
21	-28	-2	61.67	81.75
9	-16	4	144.87	41.60
31	-39	1	-0.40	84.52
29	-37	0	-5.41	83.46
27	-35	5	101.69	73.69
21	-29	-1	-19.68	80.82
23	-32	4	15.45	77.92
15	-24	3	7.13	58.64
-4	35	3	14.53	86.50
-5	26	5	-32.09	106.84
-3	23	7	104.72	96.93
3	14	10	-63.79	84.25
-3	20	7	-40.54	107.37
-6	23	1	-22.71	70.92
1	13	9	-18.36	87.95
4	9	10	-10.83	78.31
-6	19	2	107.76	85.18
12	-2	-7	149.36	66.43
-5	15	4	81.61	84.25
10	-1	11	3.04	71.84
14	-6	-7	93.76	71.45
2	6	-4	400.94	50.18

20	−15	−7	−3.96	103.67
9	−4	10	95.22	67.75
3	1	8	17.96	61.14
−1	3	5	−65.63	70.92
26	−25	−6	−29.58	97.33
18	−19	−5	137.08	77.12
14	−20	−2	125.85	83.20
10	−16	−1	40.81	65.50
32	−39	5	14.66	75.01
10	−17	0	1013.17	110.67
20	−29	4	−5.15	82.27
−3	29	−2	124.93	78.18
−4	30	−1	96.80	84.39
−3	26	7	−33.54	84.52
−6	28	2	−14.92	76.07
−6	26	1	84.52	84.52
−4	17	6	96.80	74.35
18	−7	−8	−6.07	101.16
9	1	11	−49.39	71.58
−5	13	3	371.88	85.31
7	0	10	104.33	73.69
13	−7	11	−0.26	64.84
−4	9	2	1448.84	135.23
10	−6	10	42.92	62.73
−1	5	6	219.75	69.33
11	−12	−4	91.91	85.71
3	−6	0	−2.11	45.30
13	−17	−3	12.41	90.86
17	−22	−3	70.92	76.99
6	−12	3	359.87	44.37
14	−22	0	182.24	81.22
23	−32	1	32.09	95.74
17	−26	1	72.63	71.71
−3	36	0	−3.43	82.54
−4	36	2	173.92	87.29
−6	23	4	−5.15	84.39
6	10	11	51.64	82.41
−3	17	7	120.04	97.73
2	8	9	−18.36	89.80
−4	12	5	135.63	79.10
−4	10	4	143.55	77.12
0	5	7	129.16	70.39
−3	6	3	677.74	80.95
19	−17	−6	137.48	79.37
7	−8	−3	146.06	59.82
8	−11	7	56.65	39.75
26	−32	7	66.43	58.50
29	−36	−1	−71.58	89.67
11	−19	3	26.28	54.81
25	−34	2	−17.43	88.48
−3	34	−1	−4.23	87.29
−4	29	6	59.56	87.56
−5	29	5	0.00	98.65
−6	26	4	−4.49	84.65

0	18	9	38.43	87.16
15	−1	−8	59.03	99.05
16	−3	−8	−64.18	100.76
17	−5	−8	38.83	100.37
−5	17	5	105.25	78.71
−6	18	3	−7.92	90.99
13	−2	12	23.77	70.26
8	3	11	74.35	81.09
23	−14	−8	109.08	87.69
6	2	10	0.13	71.58
2	3	8	83.46	66.03
14	−10	−6	305.46	81.75
2	2	−3	666.64	65.63
13	−14	9	0.00	46.75
28	−31	−4	62.60	92.71
9	−13	−2	128.23	61.94
26	−31	−3	42.00	108.69
14	−21	−1	484.80	102.35
24	−33	3	−60.62	86.76
20	−29	1	43.45	70.92
16	−25	2	66.16	64.84
−3	38	1	92.31	85.18
−5	34	3	−31.03	90.59
−5	33	1	76.99	101.29
−5	33	4	94.16	90.07
−5	31	0	47.67	92.31
−6	30	3	3.83	104.33
−2	23	8	77.12	85.18
−6	20	4	27.34	77.78
12	0	12	5.94	71.84
14	−4	12	−21.39	69.07
−4	11	0	913.73	90.99
19	−13	−7	23.77	89.41
23	−18	−7	3.17	111.99
14	−9	11	19.41	61.01
19	−16	11	0.00	51.64
11	−8	10	30.37	58.50
27	−28	−5	−5.81	94.29
21	−22	−5	−26.81	83.07
19	−22	−4	−20.47	81.48
23	−28	−3	1.72	90.86
20	−25	−3	72.50	77.26
34	−41	3	−0.26	83.46
33	−40	4	−74.48	83.99
16	−23	6	31.56	53.48
9	−16	1	287.36	62.73
30	−38	2	54.41	79.76
26	−34	0	−5.55	97.46
−6	29	1	−32.62	77.26
−5	23	−1	56.65	65.11
3	11	10	71.45	80.16
−2	16	−3	28.26	49.79
7	4	−6	283.93	60.09
−1	12	8	27.20	96.27

4	6	−5	115.69	49.92
−1	11	−3	462.35	47.01
15	−6	12	6.21	66.56
−2	5	5	523.49	90.73
24	−25	−5	124.14	107.10
8	−9	8	34.47	49.39
33	−40	1	47.81	80.16
31	−38	0	47.94	83.86
28	−36	4	27.47	78.71
24	−32	5	38.30	64.58
22	−31	2	−33.28	90.33
21	−30	3	17.04	76.60
18	−27	3	105.25	64.31
−4	35	0	−50.84	87.69
−5	35	2	23.77	90.99
−2	28	−3	−26.02	67.88
−6	31	2	−8.19	99.71
−6	29	4	85.44	107.37
−5	26	−1	22.19	66.82
5	11	−6	91.39	36.32
0	15	9	−9.64	86.10
−2	17	8	7.53	98.52
11	2	12	−24.17	79.24
7	5	11	−47.67	83.73
−3	14	−2	500.12	61.67
22	−12	−8	−23.64	90.20
26	−17	−8	−32.22	82.01
5	4	10	13.34	75.41
1	5	8	208.52	72.90
−1	7	7	−48.07	70.65
26	−21	−7	−56.13	95.74
13	−8	−6	78.31	71.45
22	−20	−6	0.00	112.12
17	−17	−5	287.63	83.46
10	−10	−4	2142.82	199.02
6	−6	−3	283.27	59.16
15	−17	−4	59.69	74.48
25	−28	−4	−10.83	108.16
22	−25	−4	19.94	85.44
31	−36	7	0.00	65.63
34	−40	5	−58.11	77.39
26	−32	−2	−45.30	106.97
17	−23	−2	−18.62	81.75
26	−33	−1	71.05	111.06
29	−37	3	0.00	79.50
28	−36	1	−24.30	83.33
13	−21	4	50.58	48.07
19	−28	2	7.92	68.41
−4	37	1	14.66	79.63
−5	25	6	−22.85	108.82
−4	22	−2	45.16	58.11
−5	22	6	−34.20	92.44
1	10	9	63.92	87.29
−4	14	6	470.67	95.48

12	−10	−5	148.04	75.27
31	−37	−1	−18.09	85.18
25	−32	6	−20.73	60.62
23	−31	0	11.49	83.59
18	−26	5	0.00	67.22
−4	25	−2	26.81	62.99
−4	25	7	−55.60	91.91
−6	25	5	−7.66	94.56
−4	22	7	65.37	103.27
−6	24	0	132.33	71.31
−6	22	5	30.77	76.33
−5	20	−1	224.11	78.58
10	4	12	0.00	83.20
−6	16	2	225.16	79.50
18	−11	−7	76.33	81.61
18	−15	−6	56.39	81.48
0	−1	4	113.18	45.43
2	−4	0	387.73	39.35
1	−3	4	127.97	42.13
12	−15	−3	208.92	85.31
23	−29	−2	48.33	89.54
23	−29	7	6.60	50.84
20	−26	−2	21.79	76.20
17	−24	−1	104.46	77.92
21	−29	5	26.41	75.14
20	−28	0	1.19	73.56
−4	33	−1	−9.38	95.61
−6	33	3	32.75	92.18
−5	28	6	41.47	89.41
−6	28	5	−12.02	104.99
−6	27	0	36.18	72.24
9	7	−7	109.74	55.47
−5	19	6	95.88	86.10
−6	17	1	442.80	80.82
−6	17	4	99.05	77.92
4	6	10	62.60	77.78
12	−6	−6	172.60	70.39
5	−1	9	5.02	68.01
6	−3	9	51.77	68.28
12	−10	10	17.17	55.99
5	−4	−3	282.48	54.01
28	−32	−3	0.00	89.54
16	−20	−3	197.96	80.95
13	−18	−2	402.65	96.27
35	−41	4	−56.26	81.88
20	−26	7	−1.06	44.64
12	−20	2	16.90	70.39
−6	32	1	−74.48	104.33
−7	27	2	47.54	76.20
−7	26	3	206.41	80.95
−7	23	3	138.14	80.95
2	13	10	−16.64	79.63
−4	19	7	76.07	101.82
−6	21	0	50.18	78.71

21	−10	−8	26.02	97.06
8	2	−6	−31.69	55.07
−5	14	5	51.24	74.22
0	7	8	239.82	81.61
−5	12	4	129.95	82.54
22	−16	−7	98.25	104.72
4	1	9	−12.68	71.58
15	−11	11	39.88	56.52
−4	8	3	352.47	65.37
7	−5	9	91.52	64.71
4	−2	−3	590.58	66.82
−1	1	4	150.29	49.92
9	−14	−1	−1.19	56.26
30	−36	6	4.09	69.46
32	−39	2	−44.37	83.07
29	−36	5	−13.34	73.95
20	−27	−1	63.65	76.07
−5	29	−1	−19.55	68.54
−3	22	8	8.45	83.99
−6	19	5	149.36	79.24
−2	14	8	−35.52	105.12
25	−15	−8	0.00	81.22
−6	15	3	187.53	89.14
11	−4	−6	277.99	71.71
−5	12	1	555.98	72.11
−3	7	5	1028.89	119.52
11	−8	−5	109.87	82.93
20	−20	−5	51.24	75.67
8	−11	−2	636.67	81.48
36	−41	5	−11.62	78.58
9	−15	0	280.63	61.67
28	−35	0	−5.41	89.93
27	−35	2	0.00	81.22
25	−33	1	0.00	102.35
−5	36	1	25.75	80.03
−5	34	0	−33.28	91.78
−6	34	2	13.73	93.10
−4	28	−2	156.62	69.20
−7	25	4	25.09	76.46
−1	17	9	−16.11	81.22
−4	19	−2	90.99	62.73
−7	21	2	242.73	80.16
0	12	9	24.17	92.05
3	8	10	−18.22	78.58
9	0	−6	59.30	60.88
17	−9	−7	40.81	76.60
10	−2	−6	29.05	60.48
25	−19	−7	63.13	100.76
3	3	9	92.44	73.16
−3	9	6	−9.90	71.71
−5	11	2	344.15	65.63
21	−18	−6	16.24	88.22
26	−26	−5	153.19	108.82
23	−23	−5	251.05	98.52

27	−29	−4	−94.03	108.16
18	−20	−4	135.36	85.18
25	−29	−3	−56.52	117.14
28	−33	−2	23.90	101.95
30	−37	1	71.45	79.37
22	−29	6	1.72	55.60
25	−33	4	1.98	72.24
−6	31	5	25.88	88.08
−6	30	0	−43.45	79.90
−7	30	2	17.30	94.56
−7	28	4	8.72	99.31
−7	25	1	107.63	82.67
−7	20	3	36.98	72.37
20	−8	−8	86.76	96.27
17	−13	−6	4.09	71.58
14	−15	−4	76.20	86.24
22	−26	−3	0.00	75.27
19	−23	−3	94.29	77.92
28	−34	−1	−6.21	92.18
13	−19	−1	18.49	79.50
30	−37	4	37.37	79.50
13	−20	0	182.77	77.92
26	−34	3	−16.38	80.16
−7	22	4	55.60	80.82
−4	16	7	−17.17	87.95
−5	16	6	127.70	80.03
2	5	9	81.75	91.91
24	−21	−6	11.49	114.89
8	−7	9	−37.77	60.22
−2	3	4	162.44	55.07
24	−26	−4	27.73	93.90
21	−23	−4	−4.09	73.95
16	−24	1	−7.79	64.71
−5	38	2	−27.20	87.29
−7	28	1	136.02	76.99
−6	24	6	−106.84	110.93
1	15	10	9.64	81.09
−7	22	1	−4.62	76.86
24	−13	−8	0.00	81.09
16	−7	−7	98.12	73.03
5	4	−5	164.28	57.58
−1	9	8	−45.69	83.59
21	−14	−7	79.63	105.25
3	4	−4	1242.83	112.65
10	−6	−5	190.30	80.56
17	−17	10	−14.26	39.75
−1	1	1	980.69	83.99
1	−2	0	35.52	7.53
9	−11	8	30.11	45.96
38	−42	5	−24.04	79.63
34	−40	2	0.00	82.80
22	−30	1	39.88	74.48
−6	36	3	103.01	87.95
−7	32	3	−24.30	102.22

−1	24	−4	52.03	52.43
−5	27	7	16.64	80.16
−6	27	6	−7.40	92.97
−1	21	−4	37.11	45.43
−5	24	7	11.89	89.93
10	5	−7	109.48	57.31
4	11	11	30.24	80.82
19	−6	−8	21.92	99.44
2	10	10	−4.75	75.67
11	−13	−3	−5.94	71.58
25	−30	−2	59.30	108.42
16	−21	−2	97.73	71.58
25	−32	0	76.33	100.76
24	−32	2	45.56	98.78
19	−27	1	−22.19	69.86
−6	35	1	0.00	88.48
−5	32	−1	−10.43	98.91
−3	24	−3	68.28	56.39
−7	27	5	−115.55	105.25
−7	24	5	71.58	81.09
−5	21	7	26.41	112.91
−6	21	6	−33.28	89.14
−1	14	9	19.81	86.24
−3	16	8	32.22	99.31
−5	17	−1	523.09	77.92
−6	18	0	302.16	77.12
27	−16	−8	−45.43	82.41
8	0	11	23.77	67.75
1	7	9	14.53	91.78
−3	11	7	118.59	75.54
−4	11	6	219.88	75.54
−4	9	5	219.88	89.27
15	−18	−3	46.35	76.60
32	−38	1	−34.47	81.09
30	−36	0	−73.69	85.84
22	−30	4	65.37	87.95
16	−24	4	17.56	53.75
14	−22	3	65.63	55.33
−7	33	2	71.05	100.89
−7	31	1	−17.30	83.33
−2	19	9	0.00	78.97
18	−4	−8	96.80	102.74
−7	19	4	235.86	83.07
15	−5	−7	73.69	71.58
−6	16	5	75.27	72.50
7	2	11	−1.19	67.22
−6	14	4	3.30	86.10
27	−20	−7	0.00	83.46
24	−17	−7	47.01	104.20
9	−2	11	−4.23	64.45
16	−11	−6	189.51	78.05
9	−4	−5	309.55	75.14
−5	10	3	655.68	82.80
7	−4	−4	1361.55	131.93

-3	5	4	481.23	73.82
28	-27	-5	33.28	91.25
19	-18	-5	-17.04	73.29
29	-30	-4	5.81	88.74
7	-9	-2	739.67	82.93
30	-34	-2	25.49	85.71
12	-16	-2	306.12	92.84
28	-33	7	-3.30	58.77
22	-27	-2	52.16	73.43
19	-24	-2	50.84	76.86
31	-37	5	25.22	73.95
29	-36	2	10.04	76.73
26	-33	5	25.75	66.95
15	-23	2	100.63	60.35
-6	37	2	-2.91	79.24
-4	31	-2	-47.81	92.97
-6	33	0	11.49	100.89
-7	30	5	110.67	98.12
17	-2	-8	-44.37	97.33
23	-11	-8	31.43	83.20
10	1	12	-38.96	69.60
-7	18	2	225.43	80.69
11	-1	12	21.66	69.20
-2	11	8	70.26	94.42
-5	13	0	539.47	68.80
10	-4	11	24.30	63.65
20	-16	-6	32.88	78.84
16	-13	11	-0.13	52.16
13	-12	10	-7.53	49.39
27	-30	-3	12.81	110.14
26	-30	8	14.26	50.45
32	-37	6	-7.26	68.28
30	-35	-1	-52.69	85.71
33	-39	3	18.09	82.54
16	-22	-1	59.16	76.99
8	-14	1	195.58	49.26
27	-34	1	33.02	90.20
22	-29	0	40.28	69.60
16	-23	0	255.80	75.54
23	-31	3	-17.30	93.24
19	-27	4	38.17	65.37
-7	34	4	0.00	82.01
-3	27	-3	-1.85	61.28
-3	21	-3	244.44	55.73
11	3	-7	182.11	62.86
-7	21	5	212.62	83.46
1	12	10	28.79	76.99
9	3	12	-65.37	79.50
6	4	11	37.37	78.71
-7	17	3	231.90	88.35
12	-3	12	50.84	67.22
20	-12	-7	51.37	92.44
6	2	-5	140.25	61.28
8	-2	-5	209.84	71.58

25	−24	−5	61.28	106.97
22	−21	−5	19.55	82.80
13	−13	−4	46.75	93.24
17	−18	−4	25.62	69.73
8	−12	−1	183.04	54.15
32	−38	4	−17.17	77.65
22	−28	−1	95.35	74.75
12	−18	6	9.11	24.56
7	−13	2	96.01	40.81
21	−29	2	20.47	68.80
−5	39	1	−18.88	87.16
−5	37	0	−48.33	80.43
−7	35	3	−83.73	82.67
−6	25	−1	−3.04	66.56
−8	26	2	41.07	75.67
−8	25	3	0.00	72.90
12	1	−7	92.97	64.71
−5	18	7	−28.00	95.08
13	−1	−7	95.08	66.95
−6	18	6	−3.04	73.95
−7	19	1	345.87	89.80
0	9	9	16.64	86.76
7	0	−5	1323.12	129.68
−6	13	2	84.92	62.33
4	2	−4	195.05	48.33
26	−22	−6	−43.98	105.25
23	−19	−6	197.43	110.14
6	−2	−4	171.42	57.71
26	−27	−4	−25.22	107.76
27	−33	6	−1.85	52.96
19	−25	−1	0.00	76.99
12	−19	1	351.28	73.69
18	−26	2	−43.71	66.82
−6	28	−1	−41.60	65.77
−8	29	2	10.83	81.35
−7	26	0	69.07	71.84
−8	27	4	33.68	82.67
−2	16	9	−0.26	82.01
8	5	12	−84.52	81.88
26	−14	−8	0.00	79.37
5	6	11	−0.79	79.50
13	−5	12	16.11	64.71
4	3	10	68.01	72.37
5	1	10	12.02	69.07
5	0	−4	606.82	73.82
−2	3	1	490.47	52.69
24	−27	−3	28.13	83.46
18	−21	−3	276.93	79.76
32	−37	0	44.64	78.97
28	−35	3	38.96	75.14
20	−28	3	31.17	68.41
17	−25	3	−18.09	60.62
−7	34	1	7.53	97.46
−8	31	3	177.89	101.69

-7	29	0	22.05	67.22
-8	30	4	37.51	104.99
-8	24	4	99.44	79.10
-8	23	2	108.03	71.05
-4	18	8	19.15	100.76
-8	22	3	29.98	71.45
22	-9	-8	55.60	87.03
23	-15	-7	-34.34	111.99
-5	13	6	5.15	72.37
-6	14	1	156.36	59.03
15	-9	-6	151.34	74.88
-5	11	5	89.93	81.22
7	-3	10	66.43	62.07
-4	7	4	1366.44	136.29
1	0	7	46.09	60.09
23	-24	-4	-9.90	79.76
20	-21	-4	3.04	72.50
10	-11	-3	658.98	89.93
21	-24	-3	90.99	80.16
32	-36	-1	8.98	76.20
27	-31	-2	-38.56	108.29
35	-40	3	52.56	81.09
8	-13	0	613.29	74.88
27	-33	0	71.84	97.73
10	-17	3	226.62	59.43
-7	36	2	-94.95	89.01
-7	29	6	-33.54	86.37
-6	26	7	-16.64	83.33
-7	26	6	-19.68	103.93
-6	23	7	7.13	98.65
-6	22	-1	9.38	63.52
-7	23	0	274.29	71.84
-3	13	8	9.64	96.80
19	-10	-7	76.07	89.01
3	5	10	-19.02	74.48
0	2	7	144.34	63.52
2	-2	7	143.95	59.30
29	-31	-3	3.83	97.20
33	-38	5	-89.93	76.99
12	-17	-1	423.52	87.95
31	-37	2	-23.24	75.67
-6	38	1	-32.88	82.01
-5	35	-1	22.71	84.92
-8	32	2	-31.30	103.01
-7	23	6	55.47	95.35
0	14	10	35.79	78.44
-7	18	5	294.23	81.35
-1	11	9	54.28	85.97
26	-18	-7	-16.11	89.41
19	-14	-6	94.29	77.26
14	-11	-5	173.13	71.18
-1	4	7	42.66	64.45
34	-38	6	0.00	72.77
34	-39	4	-28.53	80.95

12	−18	0	387.86	84.12
26	−33	2	−13.34	92.05
24	−31	1	−63.79	91.52
−6	36	0	20.60	83.33
−6	31	−1	11.23	83.46
−8	33	4	−43.32	90.59
−5	27	−2	−48.60	64.31
−5	24	−2	−11.36	61.01
−8	27	1	11.09	72.90
−8	26	5	59.03	97.06
6	9	−6	101.95	43.98
2	7	10	0.00	75.01
−7	16	4	207.86	90.99
14	−7	12	−14.26	64.31
−6	12	3	371.49	76.73
8	−5	10	0.00	61.01
1	2	8	65.37	63.92
27	−25	−5	3.57	105.12
2	0	8	4.62	59.30
0	0	6	1000.49	105.78
6	−7	−2	168.11	49.13
14	−16	−3	16.24	85.18
15	−19	−2	206.41	86.90
29	−35	1	−0.92	82.01
23	−30	5	−24.70	60.62
14	−21	5	171.02	46.62
11	−18	2	284.99	68.80
−8	34	3	−42.00	88.88
−7	32	0	0.00	93.50
−8	30	1	40.67	74.88
−8	29	5	0.00	100.50
−5	23	8	−45.43	81.75
−8	24	1	−40.54	73.95
21	−7	−8	−14.26	89.41
25	−12	−8	37.64	82.93
3	10	11	92.44	83.73
−8	21	4	202.32	78.58
14	−7	−6	552.15	88.48
28	−23	−6	19.81	93.50
12	−8	11	36.32	58.50
0	4	8	226.35	68.14
12	−11	−4	81.48	78.84
3	−2	8	17.43	58.50
−1	2	6	224.64	64.45
28	−28	−4	−4.89	105.91
24	−28	−2	66.95	79.24
9	−13	7	−21.66	29.98
−1	18	−4	29.32	36.45
−3	18	9	85.05	80.95
−8	23	5	−19.41	80.16
−6	20	7	−50.58	102.48
−7	20	6	−6.21	75.54
−4	16	−2	426.69	64.84
25	−20	−6	102.35	112.65

22	−17	−6	−23.11	88.61
−2	6	7	40.01	68.01
−5	9	4	483.21	87.42
24	−22	−5	38.43	90.33
21	−19	−5	−19.15	74.22
−2	4	6	103.14	62.99
−3	5	1	1229.62	116.74
16	−16	−4	169.57	70.92
3	−4	7	123.74	54.01
1	−2	6	229.13	59.69
26	−28	−3	29.05	98.39
14	−16	9	−17.70	35.13
29	−32	−2	−83.86	94.82
11	−14	−2	677.87	90.20
21	−28	1	104.72	70.39
−5	30	−2	75.01	77.39
−8	32	5	−10.96	85.58
−8	20	2	80.95	80.82
−2	13	9	12.15	84.78
−4	15	8	−51.90	97.46
−8	19	3	70.26	82.54
18	−8	−7	−33.54	77.92
1	9	10	18.09	75.01
22	−13	−7	42.52	104.86
−6	15	6	148.17	80.82
−6	13	5	177.62	74.75
−2	2	2	140.91	43.58
36	−40	4	−7.00	80.03
35	−39	5	−36.32	75.54
21	−25	−2	−10.17	83.86
18	−22	−2	−8.32	72.77
33	−38	2	0.53	83.99
29	−34	0	46.09	92.05
24	−29	−1	−14.39	82.67
30	−36	3	−56.26	77.65
28	−34	5	60.35	74.35
24	−30	0	44.24	80.69
25	−32	3	−28.39	84.52
−7	37	1	9.90	80.56
−8	35	2	50.32	92.57
20	−5	−8	−37.77	86.90
−1	16	10	3.17	84.52
−7	20	0	124.27	78.31
28	−19	−7	51.90	89.41
13	−9	−5	2.51	71.58
25	−25	−4	94.56	96.01
−1	0	2	415.07	51.64
36	−39	6	−2.11	77.12
30	−34	7	18.88	61.67
29	−33	−1	77.26	96.67
25	−30	7	−17.04	53.35
24	−31	4	−22.19	80.56
18	−25	1	−23.77	71.18
−8	33	1	10.04	104.46

−9	30	3	1.32	96.93
−9	27	3	−33.15	76.33
−5	21	−2	40.54	57.05
2	12	11	14.26	80.16
25	−16	−7	−61.80	94.29
13	−5	−6	0.00	64.31
−7	15	2	52.43	68.28
18	−12	−6	21.26	73.95
−3	8	7	63.65	68.01
29	−26	−5	−4.09	95.48
9	−7	10	87.42	59.96
19	−19	−4	125.46	77.78
9	−9	−3	608.54	83.20
4	−4	8	254.88	60.62
23	−25	−3	−112.91	76.73
17	−19	−3	4.89	75.27
7	−10	−1	1034.30	100.89
23	−27	8	32.09	44.50
3	−7	4	1070.49	93.37
31	−36	1	−13.60	81.61
15	−20	−1	171.28	86.37
24	−30	6	−10.83	58.64
23	−30	2	57.84	80.69
20	−27	5	18.09	75.54
−8	37	3	0.00	84.12
−6	34	−1	0.00	91.25
−7	35	0	59.69	90.46
−8	28	6	−27.60	96.01
−9	29	4	3.30	100.50
−9	28	2	4.89	74.88
−7	25	7	23.11	93.37
−9	26	4	0.00	78.44
19	−3	−8	16.51	92.84
−3	18	−3	59.69	46.75
24	−10	−8	0.00	82.54
−2	8	8	50.98	74.35
30	−29	−4	141.31	90.73
−3	4	2	163.10	48.73
22	−22	−4	29.05	78.18
10	−11	9	−7.79	49.39
35	−39	2	−27.34	83.20
29	−34	6	−41.34	67.09
21	−26	−1	−8.85	75.80
15	−21	0	69.99	79.50
12	−19	4	244.05	48.60
−9	33	3	15.32	99.71
−9	31	2	23.24	88.61
−8	25	6	14.13	105.52
−4	20	9	−27.20	82.67
−9	24	3	65.90	76.07
27	−13	−8	−76.60	80.03
−6	19	−1	47.01	67.22
−8	20	5	49.52	70.65
17	−6	−7	66.43	71.84

0	11	10	100.23	78.58
−6	17	7	61.54	85.44
−7	14	3	1354.68	146.59
1	4	9	30.77	70.78
2	2	9	54.67	67.75
3	0	9	169.70	69.20
31	−33	−2	20.34	92.31
20	−22	−3	47.81	76.20
2	−4	6	164.55	52.69
37	−40	5	64.05	75.94
18	−23	−1	−12.55	67.09
28	−34	2	0.00	84.39
26	−32	1	206.28	102.08
17	−24	5	33.28	53.75
−7	22	7	71.31	101.82
−3	15	9	25.75	82.54
21	−11	−7	106.84	101.03
−8	18	4	137.74	73.43
−7	16	1	377.03	71.97
−2	9	−2	662.55	60.22
27	−21	−6	−37.11	98.25
21	−15	−6	−17.30	76.33
−4	10	7	56.65	70.12
−6	11	4	328.30	85.58
26	−23	−5	64.45	106.84
11	−9	−4	745.22	102.61
−2	4	0	403.05	40.54
5	−5	−2	70.26	44.77
28	−29	−3	35.13	102.48
26	−29	−2	90.99	92.44
31	−35	0	55.47	78.84
32	−37	3	−37.11	82.93
18	−24	0	66.03	67.35
−8	38	2	−22.71	78.44
−8	28	0	−24.30	72.11
−9	28	5	59.16	106.44
7	7	−6	398.96	61.01
−9	23	4	97.33	82.01
−5	17	8	2.25	106.18
7	4	12	−4.09	76.33
8	2	12	−24.70	70.12
−7	17	6	252.63	81.88
12	−3	−6	135.76	67.22
9	0	12	15.45	69.07
5	3	11	−70.12	71.58
−7	15	5	89.93	68.54
6	1	11	20.60	67.22
−3	10	8	44.77	84.12
24	−18	−6	154.38	103.93
0	6	9	−30.90	92.71
13	−10	11	14.26	56.52
−4	7	1	2727.59	231.90
17	−15	11	0.00	47.67
4	−2	9	103.40	67.75

14	−14	10	−7.79	44.11
13	−14	−3	184.62	83.59
4	−6	7	−1.98	49.79
31	−34	−1	−3.43	81.88
33	−37	1	25.22	82.01
14	−21	2	336.36	70.78
−8	36	1	103.40	84.52
−9	35	4	−51.37	83.99
−9	34	2	−9.64	97.20
−8	31	0	13.34	78.58
−9	31	5	−47.41	93.76
−7	27	−1	0.00	67.22
−8	25	0	41.86	66.95
−6	22	8	4.23	83.86
−9	25	5	47.41	85.84
23	−8	−8	104.06	82.93
−8	22	6	2.11	87.03
16	−4	−7	33.94	70.26
6	6	12	20.60	82.80
−1	13	10	−41.47	81.61
4	5	11	76.73	80.43
17	−10	−6	308.76	77.26
12	−7	−5	90.46	78.44
23	−20	−5	58.90	77.26
−4	6	2	97.20	47.67
27	−26	−4	−35.66	111.72
15	−14	−4	44.77	74.61
14	−17	−2	496.68	95.48
10	−13	8	33.94	49.26
26	−30	−1	29.85	88.22
11	−15	−1	283.67	67.75
26	−31	0	52.96	95.74
7	−12	1	526.79	62.60
22	−29	3	54.54	77.92
21	−28	4	−31.56	80.43
20	−27	2	30.11	60.62
−7	40	1	−2.91	81.75
−9	36	3	−28.26	86.37
−7	30	−1	134.57	74.61
27	−17	−7	−43.05	86.10
24	−14	−7	58.64	101.16
10	−2	12	−34.20	66.16
−1	8	9	−3.70	90.59
7	−1	11	36.58	65.11
−5	10	6	44.24	71.31
5	−6	8	84.25	52.30
28	−31	8	59.30	55.60
7	−11	0	846.91	85.97
31	−36	4	6.74	76.60
30	−35	5	17.04	72.11
11	−17	1	405.96	72.37
17	−24	2	87.69	62.46
−4	26	−3	38.30	57.45
−9	29	1	−9.77	71.31

−7	24	−1	131.80	64.97
5	8	12	61.01	80.56
−4	17	9	38.30	87.69
−9	21	3	−41.86	70.52
11	−1	−6	−17.83	61.41
−5	12	7	112.38	80.95
−3	4	5	328.70	76.33
10	−12	−2	395.92	71.84
33	−36	0	−24.96	80.95
32	−35	7	42.00	65.50
23	−26	−2	148.96	75.41
27	−33	3	−79.76	83.99
13	−20	3	78.05	52.82
−7	38	0	−11.23	79.90
−9	32	1	−25.36	93.10
−8	27	7	4.62	82.54
26	−11	−8	−27.34	79.76
15	−2	−7	64.84	68.01
8	5	−6	58.64	51.90
−9	22	2	6.34	70.65
−7	19	7	−50.45	94.56
−5	14	−1	24.43	45.83
−8	17	2	99.84	73.69
−4	12	8	49.13	93.37
29	−22	−6	−3.83	89.14
11	−4	12	13.07	64.45
8	−3	11	13.21	65.24
16	−12	−5	227.14	76.86
−4	6	5	77.52	73.82
24	−23	−4	14.79	80.56
10	−9	10	−20.21	54.01
8	−7	−3	172.74	54.94
5	−4	9	−16.77	62.46
30	−30	−3	10.17	93.76
25	−26	−3	46.22	80.69
28	−30	−2	84.78	107.10
0	−2	2	77.12	28.39
30	−35	2	−7.00	77.78
28	−33	1	−30.64	89.67
11	−16	0	545.28	78.97
23	−29	1	33.41	71.05
8	−14	4	−10.83	36.45
−7	33	−1	40.28	97.06
−8	34	0	80.95	98.65
−9	30	6	−59.82	90.73
−9	27	6	45.56	99.05
22	−6	−8	0.00	84.52
−2	15	10	43.18	81.61
−6	19	8	−107.89	97.20
−9	22	5	−5.15	78.44
9	3	−6	252.24	62.99
20	−9	−7	−7.00	92.05
10	1	−6	75.01	59.56
−2	10	9	109.48	87.82

−8	16	3	273.76	83.59
−7	13	4	87.69	76.73
28	−24	−5	−38.03	103.40
−5	8	2	725.54	80.69
18	−17	−4	85.97	82.67
20	−23	−2	−13.21	79.76
17	−20	−2	116.08	78.84
31	−35	6	−39.35	69.33
22	−27	7	−24.30	50.45
19	−26	3	135.89	65.11
18	−25	4	34.47	56.79
15	−22	4	75.67	53.09
−9	37	2	−12.02	80.95
−10	32	3	65.37	103.80
−10	31	4	−20.60	106.57
−4	23	−3	99.05	56.92
−10	28	4	66.56	89.27
−8	24	7	24.43	101.55
14	0	−7	322.89	75.67
−8	19	6	59.69	73.16
−9	20	4	76.86	79.50
−8	17	5	−7.53	77.26
20	−13	−6	89.14	75.27
−3	6	0	−8.98	44.11
29	−27	−4	13.21	108.69
21	−20	−4	0.00	76.33
16	−17	−3	−8.58	80.82
36	−39	3	49.26	82.93
23	−27	−1	75.67	78.18
20	−24	8	42.26	35.52
26	−32	4	65.50	77.39
25	−31	2	130.61	98.25
−8	39	1	−20.07	78.71
−6	29	−2	107.63	69.20
−6	26	−2	113.97	61.54
11	6	−7	72.63	58.11
−7	24	8	19.81	90.99
−10	26	3	52.56	77.92
−8	22	0	261.88	84.52
1	11	11	55.60	81.22
29	−18	−7	57.31	80.95
16	−8	−6	97.59	72.24
26	−19	−6	158.21	107.37
11	−5	−5	100.89	72.50
2	4	10	−22.45	71.18
−6	12	6	168.25	74.09
3	2	10	0.00	67.75
−5	9	1	724.62	79.10
−5	8	5	316.81	85.05
22	−23	−3	−34.34	76.86
33	−37	4	0.00	78.18
28	−32	0	25.75	103.40
23	−28	0	59.03	71.97
16	−23	3	190.43	60.09

-9	35	1	-109.87	95.61
-10	34	4	-0.13	92.97
-2	23	-4	31.17	46.62
-10	30	2	-123.34	81.48
-10	30	5	34.07	98.25
21	-4	-8	112.65	86.10
25	-9	-8	140.38	82.80
12	4	-7	72.90	61.14
13	2	-7	159.13	68.67
-9	24	6	38.43	101.16
-10	25	4	0.13	75.27
-5	19	9	22.19	84.39
-9	23	1	24.17	70.12
26	-15	-7	13.87	95.22
23	-12	-7	2.77	100.23
-8	18	1	86.76	73.95
-3	12	9	-31.03	84.25
-5	14	8	85.84	98.52
23	-16	-6	12.68	92.71
1	6	10	-5.94	72.90
25	-21	-5	22.45	90.59
9	-5	11	-7.00	62.73
4	0	10	94.56	68.80
-3	3	3	1162.00	107.63
19	-20	-3	56.26	73.56
6	-8	-1	1181.55	111.59
0	-2	5	447.69	68.28
3	-6	6	159.53	45.83
32	-36	2	-24.17	78.71
32	-36	5	179.74	78.44
14	-18	-1	526.79	90.73
20	-26	1	79.50	69.86
-10	35	3	50.84	92.18
-10	33	2	22.85	106.04
-10	33	5	90.33	81.88
-10	27	2	52.30	73.82
-10	27	5	-87.82	103.27
28	-12	-8	4.09	78.84
-3	17	10	0.00	83.59
0	8	10	-3.57	73.29
12	-6	12	-19.02	64.97
30	-25	-5	-79.50	95.74
22	-18	-5	-6.47	77.26
14	-12	-4	106.04	84.92
4	-3	-2	144.74	44.77
-4	5	3	414.54	62.86
30	-31	-2	14.53	93.63
27	-31	7	-12.94	59.16
26	-31	6	0.00	63.26
18	-24	6	-15.72	65.37
15	-21	6	-12.15	42.39
-6	32	-2	83.33	94.56
3	11	-5	147.91	31.03
0	13	11	0.00	79.10

−5	18	−2	246.29	62.46
−8	21	7	0.00	100.76
19	−7	−7	7.00	86.90
−8	15	4	207.47	86.50
31	−28	−4	33.81	93.76
27	−27	−3	−30.24	99.97
12	−12	−3	812.57	104.06
6	−6	9	62.86	59.43
−2	1	3	240.09	43.45
25	−27	−2	−52.69	87.56
33	−36	6	17.43	71.97
30	−34	1	70.12	90.20
20	−24	−1	−87.42	71.45
29	−34	3	−14.00	76.73
17	−23	1	−39.49	64.71
−8	37	0	−33.15	82.14
−7	21	−1	562.18	89.01
−4	14	9	90.07	88.74
−1	10	10	59.82	75.94
−7	14	6	740.20	103.67
15	−10	−5	12.94	68.54
−6	10	2	997.06	101.03
−6	10	5	104.06	80.43
26	−24	−4	−24.17	96.54
−5	7	3	410.71	67.88
35	−38	4	−19.02	81.35
20	−25	0	23.77	70.39
14	−19	0	149.63	73.82
6	−11	2	448.48	51.37
−10	36	2	32.35	85.71
−9	30	0	11.89	70.12
−6	23	−2	41.60	57.58
−10	24	5	−11.62	78.31
−10	23	3	19.55	75.54
−2	13	−3	65.37	33.94
−6	16	8	−34.73	99.71
−9	19	5	113.31	79.37
−7	16	7	77.78	76.99
28	−20	−6	−79.24	106.18
10	−3	−5	496.15	85.31
−4	8	0	377.17	54.94
7	−5	−3	422.33	64.45
34	−37	2	22.05	83.73
34	−37	5	−72.37	76.33
30	−33	0	−36.45	94.69
5	−8	7	33.54	43.84
17	−21	8	0.00	21.92
25	−30	1	128.36	84.78
24	−30	3	39.49	92.05
22	−28	2	−22.85	66.43
10	−16	5	−6.74	27.47
−9	38	1	−24.43	80.69
−10	29	6	70.52	92.97
24	−7	−8	48.07	80.43

−9	26	7	72.50	90.33
−9	21	6	−20.87	76.07
−9	19	2	148.70	82.54
15	−6	−6	214.20	71.45
−9	18	3	41.34	82.14
19	−11	−6	67.35	72.50
27	−22	−5	0.00	107.37
−6	11	1	101.69	49.65
9	−5	−4	633.23	87.82
14	−12	11	35.79	52.43
32	−32	−2	13.73	86.50
37	−39	4	−3.30	81.35
13	−15	−2	509.10	86.76
6	−8	8	−1.45	47.41
27	−32	2	94.29	94.69
17	−22	0	194.66	75.27
22	−28	5	0.00	76.60
−9	33	0	82.67	103.01
−8	29	−1	−5.94	66.29
−10	31	1	−41.47	78.44
5	14	−6	36.71	30.64
−9	27	0	22.71	67.62
27	−10	−8	29.45	79.37
−6	21	9	−0.40	81.61
18	−5	−7	15.32	78.71
28	−16	−7	−29.45	87.56
22	−10	−7	−29.32	100.37
−10	22	4	240.75	80.95
−2	12	10	81.61	77.78
25	−17	−6	139.59	104.20
−7	12	5	158.87	78.31
23	−21	−4	−14.79	78.71
17	−15	−4	102.08	78.97
29	−28	−3	−11.49	103.67
24	−24	−3	121.76	80.56
9	−10	−2	97.46	55.73
36	−38	2	0.00	72.77
30	−32	8	0.00	58.90
22	−24	−2	114.10	78.18
32	−35	1	0.40	78.44
25	−28	−1	42.92	77.12
10	−13	−1	150.95	54.28
28	−33	4	−41.47	77.78
27	−32	5	−51.50	70.92
19	−24	7	13.21	43.71
−11	31	3	12.68	107.10
−11	30	4	4.75	105.78
−10	28	1	28.00	69.20
−10	26	6	23.77	108.55
25	−13	−7	−38.30	96.01
−5	16	9	22.98	83.20
5	5	12	54.01	76.20
6	3	12	50.98	70.52
22	−14	−6	−49.79	80.03

10	−7	11	69.86	61.54
−6	9	3	37.24	53.48
20	−18	−4	125.99	78.05
6	−4	10	−17.17	62.99
36	−38	5	−22.71	79.76
35	−37	6	−0.13	72.50
31	−35	3	40.15	78.18
25	−29	0	30.90	82.01
23	−29	4	56.79	86.24
−8	32	−1	−7.79	89.01
−10	34	1	0.00	99.18
−11	34	3	89.80	100.23
−11	28	3	48.33	76.33
−8	23	8	−48.07	85.84
−9	23	7	5.15	99.05
4	7	12	−108.82	80.82
−6	16	−1	41.60	53.75
30	−21	−6	−15.32	85.84
7	1	12	0.00	66.43
−8	16	6	59.30	79.50
−9	17	4	37.37	78.31
24	−19	−5	−4.62	80.03
18	−13	−5	92.18	71.45
13	−8	12	0.00	54.54
−7	12	2	253.95	65.90
28	−25	−4	95.08	111.72
−2	5	8	72.90	63.79
15	−15	−3	55.73	88.88
32	−33	−1	58.37	91.25
27	−28	−2	−17.04	95.22
32	−34	0	−31.43	82.27
19	−21	−2	79.24	73.16
16	−18	−2	209.71	89.80
9	−15	3	157.81	48.60
−10	39	2	34.73	81.09
−11	32	5	−0.13	96.14
−8	26	−1	20.87	64.71
−11	29	5	16.77	99.18
−11	27	4	0.00	76.86
3	9	12	3.57	80.16
−3	14	10	65.37	79.10
−7	18	8	37.64	96.54
−8	18	7	139.06	90.86
9	−1	−5	169.83	71.05
−3	7	8	115.16	75.80
−1	3	8	−25.22	62.86
−3	5	7	112.65	67.09
21	−21	−3	65.11	81.88
11	−11	10	3.83	49.52
38	−39	5	−16.77	80.29
37	−38	6	0.00	76.99
34	−36	1	0.00	80.95
−9	36	0	63.26	88.88
−11	32	2	63.39	101.29

−4	20	−3	186.87	51.11
−9	24	0	70.52	65.90
14	−4	−6	651.32	91.39
2	6	11	12.02	77.39
8	−1	12	18.22	66.43
3	4	11	5.02	69.73
29	−23	−5	−48.73	110.40
14	−8	−5	12.02	63.79
4	2	11	50.58	69.20
−7	11	3	1124.50	116.48
13	−10	−4	321.04	91.65
−4	7	7	149.10	69.07
31	−29	−3	59.03	95.88
0	1	8	−17.83	61.28
−2	3	7	−4.36	62.20
18	−18	−3	261.74	82.14
−1	−1	3	235.73	39.22
1	−4	5	224.90	52.82
19	−25	2	66.43	66.43
−9	41	1	−55.86	87.69
−8	35	−1	21.79	88.61
−10	37	1	−19.68	86.10
−11	37	3	66.69	83.07
29	−11	−8	−58.50	86.37
−11	29	2	23.90	76.73
−10	25	1	0.26	74.35
17	−3	−7	238.11	77.78
21	−8	−7	10.83	97.20
−10	23	6	−5.41	102.35
−8	19	0	220.01	74.22
−10	21	5	180.40	80.95
18	−9	−6	103.01	70.26
−3	11	−2	586.35	58.37
−8	14	5	42.66	70.65
−4	9	8	60.35	80.82
−5	10	0	−29.05	41.07
3	−1	−2	557.03	61.28
34	−35	0	22.32	80.29
11	−13	9	−48.86	42.26
33	−36	3	6.60	81.22
22	−25	−1	−8.72	78.71
29	−33	2	−29.85	87.03
28	−32	6	−47.28	65.77
27	−31	1	−27.47	97.86
22	−27	1	134.70	70.65
21	−27	3	71.97	66.56
13	−19	2	28.92	66.29
−11	35	2	2.11	94.95
26	−8	−8	60.35	83.20
−10	28	7	0.00	82.54
−11	26	5	86.24	93.90
−11	25	3	42.39	79.63
27	−14	−7	18.88	94.56
−4	16	10	−6.21	78.58

−6	18	9	−9.77	80.29
−10	20	3	35.39	78.84
27	−18	−6	−0.79	111.46
−7	13	1	101.16	56.26
8	−3	−4	162.96	62.46
−2	7	9	13.73	93.76
30	−26	−4	21.53	100.89
−1	5	9	30.77	71.97
−5	9	7	146.72	72.50
0	3	9	195.58	72.90
26	−25	−3	−90.86	85.97
11	−10	−3	695.43	91.52
−1	1	7	40.94	60.35
7	−8	9	−27.34	52.16
27	−29	−1	0.00	91.39
29	−32	7	47.15	62.60
25	−28	8	7.40	50.71
6	−9	0	363.96	54.01
10	−14	0	1036.41	104.86
26	−31	3	15.05	92.97
10	−15	1	248.80	58.37
−5	28	−3	−2.91	61.80
−11	31	6	−70.92	91.39
24	−11	−7	78.97	103.80
−11	24	4	−30.64	77.92
−10	21	2	212.09	91.65
8	1	−5	153.85	68.28
−9	18	6	225.16	83.86
−3	9	9	66.82	87.82
−5	11	8	48.33	91.65
5	0	11	84.92	66.69
25	−22	−4	−32.09	77.92
6	−3	−3	796.59	82.93
29	−29	−2	71.45	105.38
27	−30	0	12.68	91.65
23	−28	6	5.68	59.43
19	−25	5	6.21	65.77
16	−22	2	50.45	59.96
−7	31	−2	0.00	86.10
−7	28	−2	108.82	63.92
−11	28	6	49.13	98.65
−9	25	8	12.68	81.35
−1	12	11	47.28	80.56
−9	20	7	102.08	103.93
24	−15	−6	15.19	94.69
21	−12	−6	−93.10	75.67
−10	19	4	91.39	74.09
31	−24	−5	−0.92	89.93
26	−20	−5	−13.21	101.69
9	−3	12	34.86	67.62
−8	14	2	149.36	65.50
−6	11	7	5.94	67.48
1	1	9	85.18	68.54
7	−6	10	6.07	59.30

−5	6	4	567.47	83.59
1	−1	8	14.26	56.52
24	−25	−2	65.37	74.61
35	−37	3	11.89	81.61
13	−16	−1	319.85	77.39
29	−33	5	−44.77	76.20
24	−29	2	−11.75	74.61
−9	39	0	−47.41	76.60
−11	38	2	0.53	81.61
−2	20	−4	56.52	41.07
16	−1	−7	58.50	71.71
−10	25	7	−12.41	87.82
13	−2	−6	90.73	67.09
−4	11	9	42.13	87.03
−9	16	5	31.17	72.24
−8	13	3	412.03	86.90
16	−13	−4	58.90	72.11
−6	8	4	429.86	81.88
−4	4	4	1274.92	122.95
5	−6	−1	982.01	99.44
19	−22	−1	129.29	71.05
6	−10	1	1469.44	128.23
20	−26	4	0.00	62.86
−12	35	4	2.77	86.76
−10	32	0	73.69	84.39
−12	33	3	22.98	103.40
−5	25	−3	−14.13	54.01
−12	32	4	11.75	102.74
−8	23	−1	110.93	63.92
4	9	−5	15.05	36.18
−5	18	10	4.89	82.27
−7	20	9	90.86	81.22
29	−19	−6	33.02	95.74
−6	13	8	−29.85	94.82
17	−11	−5	44.64	72.77
6	−2	11	−14.13	64.18
22	−19	−4	−5.81	75.94
31	−30	−2	−78.44	98.12
−4	5	6	53.09	61.94
0	−1	7	64.31	57.71
23	−25	9	24.30	36.45
31	−34	2	48.47	80.16
29	−32	1	−49.79	95.22
16	−19	−1	233.48	92.05
19	−24	1	−10.83	67.62
−10	40	1	0.13	83.20
−11	33	1	93.24	98.25
28	−9	−8	−36.05	83.33
1	18	−5	−5.02	15.19
−10	29	0	1.19	67.62
−11	30	1	40.28	74.48
−12	31	5	80.56	94.29
−12	29	4	−14.13	94.29
29	−15	−7	−45.30	85.84

20	−6	−7	−44.64	94.69
−6	20	−2	82.80	60.88
−11	25	6	−7.53	103.67
−2	14	11	0.00	83.86
−11	23	5	84.39	83.86
7	3	−5	308.76	67.35
19	−16	−4	244.84	77.92
28	−26	−3	−37.77	99.18
11	−9	11	34.34	55.73
23	−22	−3	−1.06	78.18
2	−1	9	106.57	66.69
−3	3	6	529.30	79.24
29	−30	−1	−33.02	105.91
12	−13	−2	210.90	67.09
−3	2	4	84.39	50.58
24	−28	7	−31.03	55.73
19	−23	0	285.38	75.27
25	−30	4	80.03	83.73
18	−24	3	112.91	62.73
−10	35	0	−19.68	95.35
−12	36	3	−22.71	88.08
15	1	−7	−48.60	73.16
17	−7	−6	295.16	74.75
−10	20	6	145.40	85.58
−5	13	9	19.55	85.05
13	−6	−5	235.07	73.69
20	−14	−5	115.55	75.67
−1	7	10	47.81	75.41
−6	12	0	343.23	56.79
0	5	10	26.28	71.84
27	−23	−4	−47.28	99.31
−7	10	4	1458.09	147.51
8	−8	−2	554.66	68.28
21	−22	−2	26.15	81.61
29	−31	0	−23.64	99.31
30	−33	6	6.34	71.58
24	−29	5	55.73	74.88
−11	36	1	−61.28	91.65
−9	31	−1	23.11	65.11
−7	25	−2	104.06	61.54
−12	28	5	0.00	107.23
26	−12	−7	118.59	96.80
23	−9	−7	10.43	100.89
6	5	−5	24.56	52.96
−7	18	−1	466.70	76.99
−11	22	3	78.18	82.14
−7	15	8	−13.87	98.65
28	−21	−5	43.18	120.70
−2	9	10	6.21	77.12
−8	15	1	−1.19	59.96
7	−1	−4	148.44	57.58
1	3	10	22.05	72.77
−7	11	6	483.21	86.90
33	−31	−2	−12.55	87.56

14	−13	−3	131.40	77.39
33	−35	2	1.32	83.73
31	−33	7	−17.17	67.48
24	−26	−1	12.02	73.43
31	−34	5	90.46	75.80
28	−32	3	−71.97	87.16
24	−28	1	92.71	77.52
13	−17	0	215.79	70.39
13	−18	1	13.60	68.01
16	−22	5	−8.32	48.99
−12	34	2	−45.83	108.42
−12	31	2	28.26	82.67
−11	27	1	34.73	70.52
−12	27	3	53.62	82.14
−12	26	4	64.18	75.80
12	0	−6	550.17	81.22
5	7	−5	79.10	45.56
−9	21	0	223.98	82.67
−10	22	7	46.22	100.76
26	−16	−6	181.19	112.78
−6	15	9	61.41	87.16
−3	11	10	−6.21	74.75
−10	18	5	111.33	75.54
−9	16	2	383.90	84.12
−9	15	3	175.38	85.05
10	−5	12	−3.04	64.45
20	−19	−3	117.14	75.54
26	−26	−2	19.81	80.56
2	−3	8	10.30	53.48
−2	1	6	197.04	62.20
34	−36	4	−28.00	80.43
31	−33	1	0.00	85.84
9	−11	−1	712.21	82.01
16	−20	0	−31.30	81.22
4	−8	6	−0.79	36.45
13	−19	5	45.43	41.47
12	−18	3	0.00	61.14
−9	34	−1	81.09	96.80
−9	28	−1	28.53	66.29
−12	30	6	26.41	93.90
14	3	−7	11.75	66.95
−10	26	0	104.06	71.71
−11	27	7	40.28	90.46
−8	22	9	29.45	89.27
−11	23	2	73.16	73.69
20	−10	−6	105.78	76.33
−11	21	4	189.51	76.60
−8	12	4	54.01	76.60
30	−27	−3	1.98	109.35
2	1	10	12.81	66.82
17	−16	−3	48.73	87.16
35	−36	2	0.00	82.80
18	−19	−2	93.76	75.67
15	−16	−2	235.86	84.65

26	−30	2	0.00	94.42
17	−23	4	54.67	56.79
15	−21	3	88.35	57.18
−10	38	0	−5.41	81.61
−12	37	2	19.55	83.99
13	5	−7	1.45	62.20
19	−4	−7	0.00	88.61
23	−13	−6	0.00	81.48
2	8	12	11.49	82.01
3	6	12	0.00	77.65
−4	13	10	−5.28	78.71
−8	17	8	10.70	110.80
29	−24	−4	−61.54	114.10
−8	13	6	88.61	75.41
7	−4	11	41.34	63.26
10	−8	−3	198.88	66.69
36	−37	4	−3.04	85.44
31	−32	0	−1.45	97.20
23	−28	3	−22.05	75.67
21	−26	2	81.48	62.33
11	−17	4	45.30	42.66
−11	39	1	17.43	87.95
−3	25	−4	12.68	48.47
6	12	−6	4.89	35.26
−12	28	2	67.09	73.29
−12	25	5	−50.71	81.48
1	10	12	0.00	76.20
30	−22	−5	0.00	101.16
4	4	12	−14.26	72.37
24	−20	−4	52.30	76.99
25	−23	−3	−57.31	76.20
38	−38	4	−1.32	81.75
3	−3	9	80.95	63.26
33	−34	1	57.05	80.29
33	−35	5	5.55	77.92
1	−3	7	50.05	54.54
28	−13	−7	0.26	84.92
−12	27	6	30.11	106.84
−10	24	8	0.00	83.07
11	2	−6	135.10	62.20
28	−17	−6	23.24	98.12
16	−5	−6	133.25	70.52
−11	22	6	1.98	84.39
−7	17	9	48.07	83.86
−4	13	−2	66.56	38.43
−9	17	7	47.94	87.29
16	−9	−5	127.31	75.01
5	2	12	−11.09	68.94
−7	14	0	1560.83	142.10
−9	14	4	316.55	90.86
32	−28	−3	20.34	101.03
33	−33	0	38.96	81.48
33	−34	7	−10.04	68.54
32	−34	6	−52.03	74.35

21	−23	−1	35.66	71.05
−2	0	4	500.64	62.99
30	−33	3	80.69	79.76
7	−10	8	119.38	42.13
−13	33	5	−8.72	89.27
−13	31	4	70.65	108.95
22	−7	−7	0.00	103.67
−11	24	7	−10.83	98.39
−5	15	10	4.09	84.39
−11	20	5	26.54	76.60
12	−4	−5	403.31	76.99
−9	17	1	33.81	68.54
−10	17	3	245.24	93.24
31	−25	−4	−37.90	101.42
−9	15	6	232.03	85.31
15	−11	−4	75.41	82.14
2	1	−2	500.64	53.35
28	−27	−2	44.11	92.57
35	−35	1	41.07	78.71
8	−8	10	−2.91	56.79
26	−27	−1	25.62	77.52
27	−31	4	−67.75	82.14
14	−20	4	−5.15	48.07
−12	40	2	56.26	85.31
−13	35	3	0.00	92.44
−13	32	3	−76.60	102.88
−13	30	5	−105.25	104.20
25	−10	−7	121.50	101.29
−3	15	−3	133.51	39.75
−12	24	3	128.63	82.01
−9	19	8	17.83	103.14
−10	18	2	−8.19	88.88
22	−15	−5	47.28	74.88
0	7	11	7.92	82.67
21	−17	−4	151.34	79.63
35	−34	0	−15.85	80.82
23	−23	−2	71.84	73.82
35	−36	5	−28.13	79.24
−1	−1	6	335.57	62.99
26	−29	1	5.02	82.41
25	−29	6	−50.45	64.58
20	−25	6	5.55	69.07
−13	38	3	54.54	81.75
−12	35	1	−71.45	99.57
−12	32	1	47.94	85.58
−5	22	−3	13.60	49.13
18	−2	−7	56.52	86.24
−9	25	−1	0.00	62.99
10	4	−6	283.67	63.79
−11	24	1	8.85	69.46
−8	19	9	−7.40	81.22
−12	23	4	54.15	80.03
19	−12	−5	78.84	81.75
6	1	−4	110.40	54.54

6	0	12	19.02	70.12
1	5	11	2.64	75.01
18	−14	−4	144.34	73.82
15	−14	11	−30.11	36.98
35	−35	7	−0.53	74.48
34	−35	6	0.00	73.29
15	−16	10	24.83	26.02
27	−29	8	−20.21	57.97
28	−31	2	40.01	97.06
21	−24	0	58.77	74.61
21	−25	1	170.89	74.09
−8	33	−2	0.66	91.52
−11	34	0	80.43	99.44
−8	30	−2	−21.66	70.65
−11	31	0	12.55	79.37
30	−14	−7	54.67	90.46
−13	28	4	119.91	83.99
30	−18	−6	−5.28	95.61
25	−14	−6	48.33	102.88
−6	17	10	−20.87	83.46
−2	11	11	13.34	83.20
−10	19	7	−13.34	101.42
−10	16	4	39.75	84.39
26	−21	−4	129.95	84.92
11	−6	−4	322.49	79.37
2	3	11	73.16	69.20
27	−24	−3	142.10	82.67
30	−28	−2	−38.56	107.10
22	−20	−3	7.00	77.12
−7	9	5	113.31	80.56
−6	7	5	101.29	77.12
37	−37	5	−13.60	83.59
32	−34	3	93.10	78.97
12	−14	−1	578.96	79.10
0	−3	3	1064.15	93.10
26	−30	5	18.88	68.80
22	−27	4	−68.41	78.31
18	−23	2	−26.68	65.90
9	−14	2	66.16	46.62
7	10	−6	243.52	50.98
−8	20	−1	281.55	71.05
−12	24	6	−6.60	101.03
22	−11	−6	91.25	87.82
19	−8	−6	38.03	70.39
−3	13	11	−29.85	83.86
−10	17	6	238.24	85.84
11	−7	12	27.07	64.45
36	−36	6	11.23	77.65
28	−28	−1	100.50	91.78
−5	5	5	822.21	102.35
−11	37	0	44.37	87.56
−12	38	1	−69.73	85.58
−13	36	2	12.55	90.07
−13	33	2	44.77	104.33

−12	29	1	73.29	73.29
−13	29	6	16.24	93.50
9	6	−6	249.07	59.16
−7	22	−2	46.88	55.60
−13	27	5	76.07	103.40
−10	23	0	43.18	68.80
−11	19	3	−25.22	80.16
−8	11	5	110.93	83.73
13	−11	−3	103.14	72.77
8	−6	11	−13.34	60.88
38	−37	6	−46.49	79.90
11	−11	−2	500.91	75.94
34	−35	3	44.77	84.39
15	−17	−1	317.61	84.65
3	−5	8	11.89	51.64
29	−32	4	−32.75	78.05
26	−29	7	0.00	61.41
9	−12	0	473.83	66.69
25	−29	3	−55.73	91.12
6	−10	7	21.26	26.94
2	−6	5	77.92	42.79
21	−26	5	18.36	80.43
−11	28	0	101.29	70.65
27	−11	−7	0.00	89.27
21	−5	−7	0.00	99.57
8	8	−6	13.60	50.18
−12	26	7	97.73	92.05
15	−3	−6	166.00	71.05
−9	21	9	−5.41	83.33
−12	22	5	48.73	79.37
3	1	11	−104.20	70.39
−6	10	8	15.45	82.67
32	−29	−2	10.70	102.22
−5	8	8	63.79	75.80
−4	6	8	37.90	68.28
4	−3	10	−11.49	66.29
20	−20	−2	64.58	69.86
30	−32	2	−25.62	89.27
28	−30	1	136.82	102.48
23	−27	2	−24.30	64.71
20	−25	3	−6.74	64.71
−13	39	2	−100.50	92.31
−10	33	−1	34.60	97.59
−6	27	−3	17.83	55.86
−8	27	−2	0.00	59.30
17	0	−7	57.84	82.14
−13	30	2	−43.58	83.07
24	−8	−7	−20.87	100.10
−11	21	7	29.58	104.06
−10	19	1	400.01	85.05
−11	20	2	99.18	85.84
28	−22	−4	26.02	101.29
7	−2	12	−31.03	70.12
−5	10	9	0.00	91.39

29	−25	−3	0.00	98.91
−4	8	9	−4.09	92.31
−9	13	5	57.97	74.75
−3	6	9	37.90	82.54
19	−17	−3	75.27	70.78
25	−24	−2	−70.92	81.75
7	−6	−2	253.69	51.50
36	−36	3	−34.86	84.78
28	−29	0	25.62	97.33
23	−24	−1	0.00	78.05
4	−5	9	0.00	59.69
22	−25	8	−17.70	47.94
−10	36	−1	−83.07	91.12
−14	36	4	−3.83	84.78
−10	30	−1	−8.98	71.05
−13	26	3	−52.43	76.46
27	−15	−6	26.68	115.03
−13	25	4	−31.17	76.73
11	−2	−5	−9.51	60.88
24	−16	−5	5.41	77.78
15	−7	−5	91.78	68.41
−11	19	6	73.29	79.37
−11	18	4	97.33	73.82
−6	12	9	24.56	84.92
23	−18	−4	139.46	80.82
−7	12	8	49.52	90.33
34	−30	−2	−49.52	97.20
16	−14	−3	−2.51	79.63
30	−29	−1	4.62	112.12
12	−11	11	0.00	52.82
−3	4	8	35.26	66.29
18	−21	0	90.99	72.90
21	−25	7	28.53	50.98
15	−20	2	65.24	68.01
−12	41	1	6.34	87.16
−14	34	3	−21.53	100.63
−14	32	5	0.00	95.22
−5	15	−2	421.54	59.56
9	−6	−3	79.50	52.30
−7	10	7	80.43	66.43
−2	4	9	7.79	71.05
−6	8	7	256.33	75.27
17	−17	−2	464.85	97.33
14	−14	−2	1100.86	126.25
12	−13	10	−16.11	28.53
9	−13	1	196.77	50.18
−14	37	3	−2.64	90.86
29	−12	−7	112.25	85.44
−14	30	4	41.34	110.01
−13	26	6	−11.75	113.44
21	−13	−5	50.84	77.78
5	3	−4	117.40	47.01
−7	14	9	34.73	84.65
−8	14	8	143.42	103.54

31	−26	−3	−101.82	109.35
−10	15	5	28.79	72.11
24	−21	−3	80.56	81.35
−5	6	7	26.02	67.35
4	−4	−1	3405.06	284.59
32	−33	2	−21.92	84.12
30	−31	1	104.99	98.52
31	−33	4	31.83	77.12
23	−25	0	−4.36	77.39
5	−7	0	−13.87	47.54
27	−30	6	−22.32	64.97
23	−26	1	124.93	79.10
2	−5	7	131.66	52.03
16	2	−7	−16.77	78.05
−14	31	3	66.56	101.16
−12	26	1	178.55	76.60
29	−16	−6	37.24	93.24
24	−12	−6	25.49	99.18
18	−6	−6	22.85	70.78
−13	24	5	53.35	81.48
18	−10	−5	94.03	74.75
30	−23	−4	−0.40	107.89
−4	10	10	54.54	78.31
20	−15	−4	88.22	80.43
−3	8	10	83.20	79.76
−8	12	7	13.87	71.58
4	−1	11	−6.47	66.16
32	−30	−1	0.00	98.25
30	−30	0	−48.60	107.23
29	−30	8	−7.53	61.67
15	−18	0	96.54	70.26
12	−15	0	393.67	70.78
0	−3	6	586.75	73.95
−1	−2	4	126.38	42.00
−13	34	1	−30.90	101.55
−3	22	−4	66.16	43.45
20	−3	−7	4.49	98.25
−14	31	6	−61.41	92.18
−13	28	7	0.00	85.71
−14	29	5	0.00	103.67
−13	27	2	60.48	71.97
14	−1	−6	79.37	68.67
21	−9	−6	66.03	74.88
−12	23	7	4.89	109.21
0	9	12	28.66	83.33
−12	21	6	27.07	78.58
1	7	12	34.86	82.80
−8	16	9	22.85	83.20
−12	20	4	−12.68	74.22
−5	12	10	−4.75	77.65
−9	16	8	−25.09	102.48
33	−27	−3	115.55	95.48
17	−12	−4	0.00	74.09
−2	6	10	−15.58	76.99

27	−25	−2	−1.19	83.59
−1	2	9	0.00	68.67
34	−34	2	24.56	78.97
−2	2	8	−25.49	60.75
−4	4	7	194.66	68.28
8	−9	−1	63.13	46.35
27	−30	3	−48.86	95.88
15	−19	1	137.61	77.26
−13	37	1	30.37	92.31
−12	33	0	11.09	94.82
26	−9	−7	102.35	90.99
−10	27	−1	42.92	66.82
26	−17	−5	45.43	94.16
5	4	13	−26.94	78.44
−9	18	0	56.65	59.82
32	−24	−4	−30.37	101.03
−6	14	10	60.88	79.63
2	5	12	−20.47	74.88
10	−4	−4	432.63	76.60
−11	17	5	46.09	80.29
36	−35	2	21.66	87.16
32	−31	0	26.02	98.52
32	−32	1	−84.78	93.24
25	−25	−1	3.57	74.48
20	−21	−1	44.64	71.71
28	−30	7	−17.43	56.26
25	−28	2	0.13	77.92
24	−28	4	158.47	92.71
17	−22	6	−30.64	56.65
−12	36	0	32.75	92.31
−14	35	2	22.05	99.57
−13	31	1	−57.97	80.29
23	−6	−7	31.17	105.12
31	−17	−6	0.00	92.57
−11	25	0	29.71	67.35
−9	22	−1	109.21	69.20
−12	22	2	72.50	72.63
25	−19	−4	0.00	75.01
8	−4	12	−5.55	64.31
−1	4	10	74.61	73.16
22	−21	−2	−18.62	70.12
35	−35	4	16.51	85.97
12	−16	1	413.22	72.77
19	−24	4	−17.43	59.96
−13	40	1	−28.79	84.92
−14	38	2	111.33	91.25
15	4	−7	77.12	71.97
−14	32	2	−7.66	98.78
−14	28	3	31.69	76.86
−12	25	8	27.20	84.78
−14	27	4	34.34	85.18
10	0	−5	39.49	62.86
−7	16	10	33.41	88.74
−9	18	9	−68.41	86.10

−10	18	8	−18.75	100.76
−4	9	−1	725.54	70.65
26	−22	−3	79.76	82.67
21	−18	−3	220.41	79.50
34	−32	0	−20.07	87.42
37	−36	4	8.32	85.31
34	−33	1	29.58	80.29
5	−5	10	29.98	62.07
30	−32	5	−12.68	73.56
20	−24	2	1.72	67.75
17	−22	3	141.04	61.41
−12	39	0	51.11	83.86
−9	32	−2	−13.34	88.88
−15	35	4	−86.50	89.80
−15	34	5	104.20	88.74
−12	30	0	24.04	71.97
−14	28	6	−12.94	97.59
26	−13	−6	−90.46	111.33
−4	17	−3	99.44	50.45
3	3	12	20.34	71.45
29	−26	−2	−22.45	91.91
36	−34	1	52.16	84.25
−7	9	2	789.99	84.78
25	−26	9	2.38	49.92
−3	2	7	18.75	62.73
29	−31	6	−10.30	73.95
28	−10	−7	9.77	87.69
−6	24	−3	5.41	53.88
−15	32	4	89.54	106.04
−8	24	−2	48.47	58.24
−13	25	7	−7.26	91.39
−14	26	5	35.79	94.95
−13	23	3	54.28	79.10
−13	23	6	32.22	93.63
23	−14	−5	62.07	76.07
14	−5	−5	85.31	66.95
−13	22	4	125.19	82.54
−12	19	5	172.21	78.18
12	−9	−3	410.58	76.73
−8	11	2	622.80	81.61
0	2	10	21.39	69.07
−8	10	3	147.64	70.39
9	−8	11	−67.22	60.09
−7	8	3	192.15	60.09
31	−31	8	−44.50	68.28
0	0	9	178.28	70.26
−1	0	8	20.60	59.56
29	−31	3	−72.24	89.14
25	−27	1	71.58	74.88
23	−27	5	88.88	84.12
22	−26	3	162.04	68.80
18	−23	5	20.60	54.94
−11	35	−1	−4.62	94.42
−15	36	3	22.05	93.24

19	−1	−7	0.00	95.61
−15	31	5	−96.67	97.86
13	1	−6	28.66	63.39
17	−4	−6	8.19	71.31
−6	17	−2	132.72	58.11
−8	18	10	45.56	86.24
−10	20	9	4.62	82.41
4	5	−4	493.25	60.48
−11	20	8	−4.23	96.54
−4	12	11	0.00	85.84
27	−20	−4	39.09	89.27
−11	18	7	51.11	93.37
22	−16	−4	62.60	79.50
31	−27	−2	−43.84	109.35
−9	13	2	55.33	60.48
−9	12	3	595.46	89.93
−9	12	6	113.97	73.69
5	−3	11	15.32	64.05
27	−26	−1	49.39	82.27
−6	7	2	196.37	55.47
32	−33	5	−1.58	81.61
17	−18	−1	255.01	88.35
9	−10	10	−11.09	50.71
20	−22	0	116.61	74.09
−11	32	−1	47.81	81.09
−9	29	−2	38.83	66.56
25	−7	−7	−22.98	90.99
−15	33	3	10.56	109.74
−13	28	1	46.62	70.52
−14	29	2	36.71	81.35
23	−10	−6	−23.90	89.01
−10	20	0	262.80	80.29
−5	14	11	53.48	94.95
−6	13	−1	29.05	39.49
28	−23	−3	61.41	91.78
4	1	12	4.89	70.78
−1	6	11	−82.41	81.88
−3	7	−1	2610.98	216.71
−10	14	6	221.20	77.92
18	−15	−3	14.13	78.71
19	−18	−2	89.54	80.16
−6	6	6	−2.51	61.80
30	−31	7	−1.85	68.67
27	−29	2	−22.19	95.74
30	−11	−7	79.76	84.52
22	−4	−7	−85.58	103.40
28	−14	−6	5.68	103.67
20	−7	−6	51.90	76.73
−13	24	2	42.39	76.60
20	−11	−5	−8.19	75.80
17	−8	−5	57.84	71.05
33	−28	−2	5.02	96.27
−10	14	3	641.42	99.84
15	−12	−3	186.47	82.93

24	−22	−2	122.16	79.24
10	−9	−2	62.86	53.88
34	−34	5	94.56	81.88
−6	6	3	373.73	62.20
31	−32	3	−15.72	81.48
11	−12	−1	38.69	54.81
26	−29	4	77.92	89.41
4	−7	8	129.68	49.92
−15	29	4	−28.13	93.24
−12	23	1	167.85	74.35
29	−21	−4	−1.72	102.61
−12	20	7	73.16	106.31
−13	21	5	28.79	79.50
−10	15	2	317.74	81.22
−11	16	6	189.24	78.71
0	4	11	68.41	72.77
38	−36	5	−6.74	82.80
36	−35	5	52.43	83.07
22	−22	−1	64.84	71.97
31	−32	6	0.00	75.01
14	−15	−1	283.53	78.31
5	−7	9	87.03	57.97
5	−9	2	1525.70	129.02
14	−19	3	122.29	58.37
8	−13	3	267.82	47.41
−7	29	−3	72.11	65.90
−14	36	1	−8.58	99.44
−12	27	0	−59.69	70.39
−15	30	6	−87.29	90.07
−14	27	7	71.84	92.44
−11	22	9	14.00	90.07
−14	25	6	−30.77	106.18
25	−15	−5	0.00	81.09
30	−24	−3	40.15	109.35
19	−13	−4	41.60	77.12
23	−19	−3	43.84	77.26
8	−4	−3	413.75	63.13
29	−27	−1	30.77	94.56
−9	11	4	908.05	117.53
16	−15	−2	390.77	86.90
−8	9	4	806.89	102.61
33	−33	3	31.83	80.95
27	−28	1	−27.60	83.99
−5	4	6	66.69	62.60
20	−22	9	−26.94	35.79
−14	39	1	−21.53	84.25
−15	37	2	−70.39	89.14
18	1	−7	51.37	95.08
−14	33	1	−65.63	95.88
−11	29	−1	13.73	66.43
30	−15	−6	−0.79	98.12
−10	24	−1	246.29	68.01
−15	28	5	−28.00	111.06
9	2	−5	−7.79	59.03

−14	25	3	100.76	77.26
−14	24	4	36.58	80.82
9	−2	−4	73.29	64.31
−11	16	3	142.23	83.99
−7	10	1	1119.48	108.29
−10	13	4	400.67	98.12
35	−34	3	−17.04	84.39
13	−12	−2	267.42	71.18
33	−33	6	11.75	76.46
32	−32	7	−47.94	71.71
−5	5	2	92.84	48.20
29	−30	2	0.00	106.04
−2	0	7	180.13	60.48
5	−8	1	70.92	34.07
−13	38	0	−13.34	86.63
−15	40	2	6.60	83.20
−13	35	0	164.81	102.61
27	−8	−7	13.73	86.76
−15	34	2	−51.24	110.93
12	3	−6	146.46	64.84
25	−11	−6	4.23	99.97
31	−22	−4	−31.83	109.08
−7	15	−1	13.60	49.92
32	−25	−3	92.84	106.84
24	−17	−4	95.35	77.65
3	3	−3	642.87	64.05
−12	18	6	11.49	81.22
1	3	−2	159.40	28.13
−8	12	1	158.21	53.62
31	−28	−1	54.81	119.25
9	−6	12	14.39	65.24
37	−35	3	69.33	90.33
6	−4	−2	53.75	44.24
−7	7	4	2408.66	214.07
23	−26	7	101.69	61.80
−2	−1	5	37.37	56.26
17	−21	2	189.11	67.35
16	−21	4	10.04	54.41
−16	33	5	19.68	92.44
32	−16	−6	1.85	84.65
33	−23	−4	70.39	98.12
13	−3	−5	87.42	62.60
−13	22	7	14.26	106.71
−14	23	5	39.75	77.78
34	−26	−3	30.64	95.48
−11	17	2	185.02	80.43
−9	14	1	−15.45	54.94
5	−1	12	−13.34	68.01
−11	15	4	21.66	86.37
39	−36	6	−67.35	85.05
26	−23	−2	50.05	76.33
1	2	11	−2.64	70.12
37	−35	6	−37.90	80.56
−6	8	1	1247.32	117.53

35	−34	6	91.25	79.50
34	−33	7	7.53	75.94
1	−2	9	31.30	65.11
28	−30	4	0.00	84.12
24	−26	8	−5.94	53.88
17	−19	0	147.91	87.16
0	−2	8	−15.85	57.31
24	−27	3	−22.05	73.16
22	−25	2	0.00	68.67
11	−16	3	34.73	56.65
−14	42	1	67.35	88.88
−16	38	3	23.90	83.59
24	−5	−7	−17.43	91.39
21	−2	−7	−17.96	99.18
−13	32	0	15.45	82.27
16	−2	−6	17.56	66.56
27	−16	−5	22.32	103.27
−11	22	0	107.89	78.05
−2	10	12	69.20	85.84
−12	18	3	−6.34	89.80
−8	13	9	21.26	85.58
33	−29	−1	0.26	94.56
−7	11	9	33.15	92.31
−6	9	9	143.95	95.88
−8	11	8	93.50	87.95
36	−34	7	0.00	79.10
31	−31	2	55.73	89.01
29	−29	1	9.64	107.37
22	−23	0	0.00	73.56
−5	4	3	406.09	60.35
21	−25	4	60.62	63.26
14	−19	6	−5.68	38.43
29	−9	−7	−8.85	84.52
−16	35	3	64.31	99.05
−9	26	−2	43.32	62.73
−14	30	1	98.39	74.88
−15	31	2	−10.04	88.88
22	−8	−6	52.03	83.73
−7	19	−2	209.32	65.24
−13	25	1	12.68	75.94
−15	27	6	−95.08	109.08
22	−12	−5	178.28	78.58
3	7	−4	1484.76	129.82
3	5	13	−13.60	77.39
−1	8	12	4.89	80.29
−13	20	6	−13.47	76.33
−9	15	9	143.95	88.61
35	−30	−1	−64.05	89.14
25	−20	−3	41.86	81.09
−12	17	4	−40.54	73.29
20	−16	−3	252.63	82.54
−9	13	8	85.84	96.01
31	−29	0	−48.86	106.57
21	−19	−2	114.50	77.26

−5	7	9	50.45	94.56
−7	9	8	−51.24	79.76
24	−23	−1	25.62	77.78
6	−5	11	34.34	63.26
22	−24	1	175.38	75.41
17	−20	1	17.83	72.77
17	3	−7	−47.01	83.59
27	−12	−6	−43.05	112.65
−16	31	4	−33.68	109.21
19	−5	−6	−6.60	66.43
−5	19	−3	239.56	53.09
−15	26	4	−33.41	79.10
−12	19	2	281.03	93.24
−7	13	10	87.42	80.03
−10	16	1	65.77	64.31
−6	11	10	−48.47	81.09
−10	15	8	−19.94	101.95
28	−24	−2	0.00	81.61
−5	9	10	16.38	81.22
35	−33	2	−36.98	80.03
33	−32	2	−27.07	84.12
31	−30	1	−2.77	98.78
19	−19	−1	129.95	78.31
−6	5	4	701.64	86.90
8	−10	0	34.34	43.45
−4	2	6	117.14	62.86
19	−23	3	105.38	64.05
31	−10	−7	−25.49	87.42
−16	30	5	−34.20	110.40
29	−17	−5	−26.02	113.18
−15	27	3	0.00	75.14
19	−9	−5	182.38	76.86
16	−6	−5	0.00	66.69
−14	24	7	35.13	93.63
−8	17	−1	28.26	55.20
26	−18	−4	17.43	76.73
−8	15	10	−14.79	81.88
−10	17	9	50.32	84.25
0	6	12	−34.73	79.90
37	−34	2	53.88	85.44
33	−30	0	60.35	94.56
27	−27	9	30.11	56.52
2	−2	10	46.09	59.56
30	−31	4	−19.28	79.63
6	−7	10	50.71	56.26
1	−5	6	26.28	43.58
15	−20	5	89.27	50.45
−12	37	−1	−3.96	84.65
−12	34	−1	−34.34	96.80
−4	24	−4	−3.96	45.96
−7	26	−3	67.62	57.05
11	5	−6	131.00	61.41
−16	32	3	5.02	102.74
−15	25	5	−43.05	87.16

21	−14	−4	288.03	82.41
−13	20	3	99.71	75.41
−11	17	8	40.01	104.99
−13	19	4	189.24	77.92
35	−31	0	−19.28	91.65
35	−32	1	0.00	83.73
−4	7	10	49.39	78.97
33	−31	1	61.01	89.41
2	0	11	18.75	67.48
−10	12	5	111.72	73.82
−4	5	9	3.04	75.01
−9	10	5	66.95	86.50
14	−16	0	202.45	72.63
−15	38	1	−88.08	87.16
−10	31	−2	34.47	74.61
26	−6	−7	−28.13	90.59
29	−13	−6	0.00	99.44
−13	29	0	77.26	71.05
31	−18	−5	23.77	102.74
8	4	−5	152.40	57.84
−9	17	10	−15.72	90.33
−11	19	9	0.00	84.25
−14	22	6	56.39	85.44
27	−21	−3	0.00	75.67
30	−25	−2	−7.40	104.06
17	−13	−3	117.80	85.58
11	−7	−3	1276.50	125.85
−10	13	7	−34.86	74.61
−11	14	5	135.10	76.73
−9	11	7	109.74	73.43
−5	6	1	914.26	93.90
32	−32	4	48.60	81.48
−5	5	8	−9.24	65.24
26	−28	3	8.85	94.16
11	−13	0	50.32	51.90
3	−7	7	188.98	49.26
−16	39	2	−43.45	84.92
20	0	−7	127.17	98.65
−15	35	1	42.92	105.12
−16	36	2	0.00	91.39
−17	36	4	19.41	93.10
−11	26	−1	−53.88	65.24
−16	29	6	−2.77	96.54
24	−13	−5	0.00	75.80
28	−19	−4	−50.84	95.88
12	−5	−4	190.17	79.24
−11	18	1	480.04	87.82
−12	19	8	66.69	97.46
1	4	12	−7.53	72.90
−12	16	5	96.54	76.46
38	−35	4	−61.54	90.07
26	−24	−1	17.70	84.78
34	−33	4	8.58	82.01
−8	9	7	26.41	74.61

24	−24	0	99.31	76.46
−8	8	5	69.60	76.99
27	−29	5	0.79	76.60
24	−26	2	−7.13	69.07
19	−22	8	0.00	43.05
−15	41	1	−5.28	83.20
23	−3	−7	−0.66	96.14
−12	31	−1	−2.51	73.43
31	−14	−6	35.39	93.76
24	−9	−6	24.96	97.59
15	0	−6	76.46	66.82
33	−19	−5	149.89	94.56
−15	28	2	−8.06	80.29
−12	24	0	7.13	65.24
−15	26	7	−3.70	98.25
−10	19	10	−21.26	84.65
−13	21	2	25.09	74.22
34	−27	−2	25.36	96.01
−6	13	11	−27.86	85.18
32	−26	−2	94.82	109.61
14	−10	−3	988.34	123.34
23	−20	−2	24.96	80.03
6	−3	12	−19.02	66.56
18	−16	−2	133.51	94.56
−3	5	10	21.26	74.09
7	−7	−1	923.77	93.76
−4	3	2	172.87	44.77
14	−17	1	795.93	101.16
1	−4	2	139.19	19.02
−1	−2	7	4.89	55.86
20	−24	5	22.45	67.35
−14	37	0	12.28	90.20
28	−7	−7	0.13	83.99
−14	27	1	35.79	75.14
−16	28	4	1.32	86.76
30	−20	−4	11.89	105.65
−9	19	−1	349.17	77.26
−12	21	9	−16.38	85.05
−7	15	11	−54.94	87.42
−14	22	3	5.81	77.65
29	−22	−3	57.71	91.78
−14	21	4	329.62	89.80
22	−17	−3	75.94	83.86
16	−16	−1	280.76	81.75
−3	3	9	210.51	74.61
−7	7	7	722.37	97.46
24	−25	1	90.46	75.67
25	−27	7	9.24	60.22
13	−18	4	51.50	47.28
−14	40	0	2.91	81.35
−14	34	0	−32.62	101.16
−17	37	3	3.04	90.99
10	7	−6	365.02	66.69
−15	32	1	−25.49	85.18

−16	33	2	80.43	101.95
−17	33	4	74.48	98.25
−17	32	5	−24.83	94.03
−8	21	−2	−2.64	57.97
−16	29	3	99.18	91.25
12	−1	−5	16.11	60.62
−16	27	5	0.00	105.25
−15	24	6	−107.10	103.67
31	−23	−3	−45.16	108.29
23	−15	−4	97.86	78.05
8	0	−4	−44.11	59.69
−13	21	8	−49.79	91.52
−13	18	5	23.64	76.73
28	−25	−1	0.00	79.24
28	−29	3	0.00	94.69
26	−27	8	34.07	59.16
19	−20	0	104.72	78.05
−7	6	5	37.37	75.01
26	−28	6	44.11	68.94
−5	3	4	416.65	67.35
23	−26	4	51.64	76.99
19	−23	6	67.09	72.11
30	−8	−7	−39.35	85.97
21	−6	−6	13.73	74.35
18	−3	−6	−46.09	67.35
26	−14	−5	13.60	92.05
32	−21	−4	144.21	109.74
−12	20	1	274.42	87.56
−13	19	7	−24.56	105.12
2	2	12	31.96	72.37
−3	7	11	33.41	79.10
9	−7	−2	748.52	83.73
21	−20	−1	−15.32	71.45
−4	3	8	82.67	63.65
2	−4	9	124.01	65.24
−4	2	3	711.28	74.22
19	−22	2	83.46	65.90
−17	34	3	121.63	110.27
26	−10	−6	100.10	110.40
21	−10	−5	213.28	79.37
33	−24	−3	−11.23	102.61
−14	20	5	53.75	75.14
15	−13	−2	431.44	86.90
26	−25	0	−67.75	80.56
−2	3	10	−72.50	71.05
10	−10	11	102.61	53.88
26	−27	2	43.71	79.37
8	−11	1	223.58	47.01
−3	0	6	791.71	91.39
11	−15	2	58.24	51.24
8	−12	2	165.74	42.66
25	−4	−7	−70.12	90.73
19	2	−7	35.52	95.88
−10	28	−2	152.93	66.82

−6	21	−3	20.60	48.20
−17	31	6	103.80	87.56
−16	28	7	−21.00	86.10
2	9	−4	604.18	60.88
−14	23	2	119.65	75.01
−15	24	3	−29.58	78.05
−15	23	4	−22.58	81.75
30	−26	−1	59.96	94.29
25	−21	−2	93.76	80.69
−2	5	11	76.07	72.90
3	−2	11	0.00	65.37
30	−30	3	37.77	85.71
26	−26	1	95.35	79.24
13	−13	11	45.96	45.30
−6	5	7	21.13	64.97
11	−14	1	1287.46	122.42
1	−4	8	−50.45	53.88
22	−1	−7	18.49	97.86
−14	31	0	−38.83	73.56
28	−15	−5	95.48	116.21
7	6	−5	163.10	54.28
−17	30	4	162.30	107.23
18	−7	−5	338.47	82.93
15	−4	−5	71.18	65.11
−16	26	6	135.10	111.46
−14	21	7	47.94	102.48
24	−18	−3	84.52	82.80
32	−27	−1	0.00	112.25
39	−35	5	−92.84	88.08
38	−34	3	105.78	89.27
12	−10	−2	341.25	70.26
10	−8	12	44.50	63.26
32	−31	3	−44.11	81.88
3	−2	−1	496.02	52.56
13	−13	−1	376.51	70.39
10	−10	−1	241.67	54.54
21	−24	3	61.94	66.16
9	−12	9	4.89	40.67
−13	36	−1	0.00	92.84
−17	38	2	0.00	87.56
9	9	−6	91.12	54.15
28	−11	−6	12.94	104.46
14	2	−6	71.45	64.84
−12	28	−1	14.00	67.22
−16	30	2	94.56	78.58
−13	26	0	121.10	75.80
−17	29	5	51.24	108.55
−10	21	−1	266.37	77.26
25	−16	−4	33.41	83.07
36	−29	−1	0.00	79.90
−15	22	5	−47.28	74.88
34	−28	−1	−0.92	93.90
19	−14	−3	110.67	79.50
−8	13	0	636.27	77.78

37	−34	5	−14.13	83.73
36	−33	3	90.86	83.20
−12	15	6	106.71	79.10
35	−33	5	7.00	81.61
34	−32	3	27.47	81.75
28	−26	0	53.09	82.54
−11	13	6	112.38	75.67
33	−32	5	−71.58	81.75
−10	11	6	187.26	74.88
28	−28	2	43.32	99.05
28	−29	6	59.16	73.43
3	−4	10	13.34	63.52
−2	1	9	0.53	68.01
18	−22	4	0.26	58.37
16	−20	3	142.23	60.62
8	−12	8	0.00	35.39
−16	40	1	−15.32	86.37
−17	41	2	60.75	83.99
27	−5	−7	104.20	84.39
−16	37	1	−41.86	96.54
23	−7	−6	34.47	90.33
−18	34	5	0.00	87.29
30	−16	−5	8.85	116.35
−15	29	1	57.97	73.03
−15	25	8	0.53	73.95
1	6	13	−60.35	80.16
−4	11	12	−46.62	84.65
−9	15	0	182.38	58.50
27	−22	−2	−14.13	75.94
−7	11	0	895.11	88.22
−13	17	6	104.86	79.10
20	−17	−2	60.22	82.80
27	−28	7	−52.56	66.56
−1	−3	5	267.16	56.92
11	−16	6	52.43	29.05
−11	33	−2	−10.83	92.44
−8	28	−3	13.21	58.50
−13	33	−1	7.92	89.54
30	−12	−6	−20.07	90.20
−17	35	2	−57.31	101.55
32	−17	−5	0.00	96.14
−2	17	−4	8.45	28.26
23	−11	−5	31.69	70.92
−16	25	4	34.73	79.90
−15	23	7	36.18	101.03
−3	9	12	−24.70	81.35
3	0	12	7.26	68.28
23	−21	−1	−4.75	74.22
28	−27	1	−28.53	81.88
28	−28	8	−42.79	64.45
7	−7	11	37.11	57.71
25	−27	4	15.45	93.90
10	−15	4	19.68	47.28
−16	43	1	−7.92	84.52

−15	39	0	15.58	82.67
−18	39	3	−34.60	76.20
32	−13	−6	27.47	83.46
−16	34	1	51.37	102.61
−9	23	−2	129.95	57.97
−17	28	6	−19.41	96.80
27	−17	−4	132.59	84.12
−15	25	2	61.41	78.58
−16	26	3	59.03	76.99
26	−19	−3	114.50	82.54
−14	19	6	90.20	78.05
−10	14	9	49.65	88.08
−9	12	9	−17.17	86.50
−1	3	11	−7.92	70.26
30	−29	2	−22.85	100.50
21	−21	0	140.38	72.90
−4	4	1	3659.67	305.46
16	−17	0	28.00	75.14
−3	1	8	27.20	61.67
−5	3	7	94.82	65.77
22	−25	5	41.34	81.75
12	−17	5	101.16	39.75
29	−6	−7	−60.09	83.73
24	−2	−7	48.60	89.67
−15	36	0	41.47	97.99
0	20	−5	4.89	21.92
8	11	−6	2.77	45.69
−18	36	3	−14.39	96.01
20	−4	−6	26.94	73.03
17	−1	−6	−35.39	67.48
11	−3	−4	37.24	70.12
−16	24	5	−15.19	83.99
2	5	−3	348.64	41.34
36	−30	0	−21.53	87.69
29	−23	−2	28.00	90.20
34	−29	0	−33.41	95.74
−2	7	12	−6.21	77.65
−9	14	10	0.00	79.76
−11	16	9	−11.62	85.05
38	−34	6	−41.34	83.33
36	−32	2	13.34	81.75
−8	12	10	−31.03	79.50
34	−31	2	96.93	83.59
32	−30	2	−39.62	90.20
30	−28	1	−48.86	103.14
7	−5	12	44.90	63.79
−8	10	9	27.73	88.35
30	−30	6	−19.68	76.73
4	−5	0	709.70	70.92
31	−7	−7	124.80	83.73
−18	32	4	1.72	100.63
−18	31	5	−2.91	96.27
11	1	−5	42.39	59.56
29	−18	−4	−31.03	96.54

−16	25	7	12.68	101.16
31	−24	−2	33.28	104.72
2	4	13	−3.04	74.75
−10	16	10	−61.28	83.20
−12	18	9	23.64	83.86
36	−31	1	93.76	84.52
16	−11	−3	246.82	83.07
36	−33	6	0.26	77.39
32	−29	1	−20.34	98.65
5	−2	−2	438.84	56.13
−6	9	0	387.33	56.13
−7	10	10	59.16	78.31
−11	14	8	111.99	102.74
32	−31	6	43.45	75.94
18	−17	−1	162.96	87.42
29	−29	7	1.32	69.33
−8	7	6	21.00	66.16
21	−23	2	60.35	69.99
7	−12	4	117.80	32.75
5	−10	6	22.45	26.41
21	1	−7	−7.66	94.95
7	13	−6	10.96	43.45
25	−8	−6	57.05	103.80
−17	32	2	41.20	94.16
25	−12	−5	−47.81	87.42
−11	23	−1	159.13	66.95
−14	24	1	300.44	83.20
35	−26	−2	49.13	101.29
33	−25	−2	0.00	113.84
28	−20	−3	55.73	79.90
−11	19	0	316.15	79.24
−11	18	10	44.50	87.03
−15	21	6	−56.52	83.07
39	−34	7	0.00	83.73
10	−5	−3	657.00	83.33
34	−30	1	−26.94	92.44
−12	16	8	10.83	99.18
34	−32	6	23.24	77.65
−10	12	8	115.82	97.33
30	−29	8	52.03	65.63
27	−28	4	−25.88	90.46
23	−25	3	5.28	62.99
−11	30	−2	54.81	66.95
−15	33	0	−17.56	88.74
6	8	−5	0.00	42.26
−14	28	0	26.68	70.12
33	−20	−4	0.00	93.90
31	−19	−4	−2.25	109.08
20	−8	−5	89.67	74.22
−17	27	4	46.22	86.37
30	−21	−3	45.96	95.35
22	−13	−4	122.29	86.50
14	−6	−4	9.51	81.22
−13	20	9	61.28	87.16

21	−15	−3	−26.81	76.20
13	−8	−3	484.93	85.97
−13	18	8	10.96	98.39
37	−33	7	−79.37	81.22
22	−18	−2	29.45	73.16
−1	5	12	13.34	72.50
25	−22	−1	−32.09	74.75
−12	15	3	123.48	84.12
32	−30	8	−14.66	68.01
−11	13	3	−1.58	83.99
−7	8	9	−46.75	89.67
−9	10	8	35.92	86.90
20	−23	7	−5.28	53.48
16	−19	2	109.74	71.58
−4	1	4	386.15	59.43
26	−3	−7	129.02	85.84
13	4	−6	42.26	62.46
−13	30	−1	6.21	70.65
−7	23	−3	111.72	55.33
−16	31	1	47.15	78.31
−18	33	3	−125.19	106.31
−18	30	6	0.26	89.27
−17	28	3	55.07	79.37
32	−22	−3	54.94	110.14
7	2	−4	110.14	57.05
−17	26	5	−6.47	99.71
−8	14	11	−5.94	81.88
−13	17	3	177.36	89.41
35	−32	7	−31.30	73.69
34	−31	8	−53.35	72.77
17	−14	−2	522.96	98.91
−6	8	10	−43.05	78.05
−12	14	4	0.00	84.65
31	−30	7	−1.45	71.05
−10	11	3	521.38	83.86
16	−18	1	88.48	71.31
21	−24	6	66.56	77.65
5	−9	8	19.28	42.13
−17	39	1	−62.33	80.95
−18	40	2	54.01	86.10
27	−9	−6	−107.63	106.04
27	−13	−5	−52.82	102.61
14	−2	−5	19.68	62.33
34	−23	−3	106.04	100.50
−17	27	7	80.82	91.12
−14	22	9	31.17	81.09
−16	23	6	19.55	97.86
−14	20	8	16.77	90.46
−13	16	4	122.29	77.78
33	−31	7	−44.50	72.24
23	−22	0	67.09	72.24
0	1	11	−1.72	68.28
−11	12	4	286.57	93.10
29	−29	4	−74.88	84.25

4	−4	11	32.49	63.26
−8	8	8	−30.90	82.41
22	−23	9	3.57	45.56
−1	−1	9	90.86	67.22
0	−4	7	198.49	55.20
−17	42	1	−95.48	85.18
−18	37	2	42.39	96.27
22	−5	−6	77.26	90.20
−10	25	−2	72.50	61.80
17	−5	−5	190.83	64.71
−12	21	0	208.79	81.61
−14	19	3	173.13	83.46
27	−23	−1	−11.09	73.03
−12	16	2	300.44	85.31
−14	18	4	204.96	85.31
−11	14	2	661.36	89.14
31	−30	4	−28.53	78.18
−6	6	9	−11.36	75.27
13	−14	0	145.27	61.01
−7	5	6	70.12	65.11
17	−21	5	61.41	53.62
28	−4	−7	−94.03	86.76
23	0	−7	30.11	89.41
29	−10	−6	29.19	92.18
−17	36	1	68.67	99.57
−19	38	3	0.00	82.67
29	−14	−5	−19.81	113.04
−19	33	5	47.54	92.18
−15	26	1	118.19	71.97
24	−14	−4	223.32	83.73
−15	22	8	−34.34	87.42
−13	18	2	202.58	84.12
35	−32	4	12.81	84.12
33	−31	4	8.58	77.65
4	−2	12	63.39	65.50
−10	12	2	354.98	70.78
−12	14	7	73.95	76.46
−5	6	10	153.32	76.86
−9	9	3	536.30	80.16
−10	10	4	2218.89	206.54
25	−26	3	−2.51	76.46
23	−24	2	−58.37	69.86
24	−26	5	14.00	89.93
10	−12	10	−7.13	41.73
7	−9	10	−20.87	51.90
−2	−2	6	123.34	53.22
30	−5	−7	−2.91	81.35
−14	35	−1	−14.92	100.23
31	−11	−6	57.31	86.50
16	1	−6	21.26	65.37
31	−15	−5	7.00	99.57
22	−9	−5	53.22	75.67
−12	25	−1	−33.41	64.84
−18	29	4	32.09	99.97

−18	28	5	38.43	105.38
−17	25	6	−9.51	109.21
23	−16	−3	110.67	83.07
−15	21	3	103.80	74.88
29	−24	−1	83.33	90.99
24	−19	−2	7.40	81.22
−15	20	4	24.04	81.75
0	3	12	5.55	71.05
−5	8	11	−60.09	81.22
20	−18	−1	0.00	79.10
15	−14	−1	220.94	73.69
−11	12	7	59.69	76.33
−3	1	2	455.35	55.60
20	−23	4	−53.09	60.48
−4	1	7	102.35	63.65
15	−19	7	19.02	50.45
13	−17	3	86.50	62.20
−16	38	0	−20.60	86.90
−9	30	−3	80.69	66.69
33	−16	−5	−53.48	91.25
19	−2	−6	0.00	71.71
−3	19	−4	171.15	39.09
−18	34	2	−36.18	101.95
−17	29	2	−1.32	75.80
35	−27	−1	111.20	101.42
33	−26	−1	20.21	106.44
31	−25	−1	56.79	111.59
−15	20	7	−26.02	104.33
25	−23	0	−0.66	78.31
18	−18	0	426.29	97.99
−10	10	7	232.96	77.39
−7	6	8	−76.20	68.41
21	−23	8	15.85	49.65
18	−21	3	269.93	67.62
3	−6	9	48.60	57.58
−2	−1	8	0.00	56.13
24	−6	−6	−31.30	101.16
−19	35	3	0.00	96.27
−15	30	0	9.77	71.71
−13	23	0	44.37	70.26
18	−12	−3	−100.23	88.22
−14	20	2	152.66	81.22
−16	22	4	193.73	83.20
14	−11	−2	718.15	94.42
−14	17	5	61.01	78.97
−9	10	2	57.18	55.73
−12	13	5	115.82	72.50
−3	0	3	1233.05	110.01
3	−8	5	144.87	35.00
−16	35	0	0.00	98.65
12	6	−6	36.05	58.77
−17	33	1	26.68	96.14
10	3	−5	92.18	57.05
1	11	−4	99.57	26.41

26	−15	−4	73.16	78.71
−16	23	3	23.90	77.65
26	−20	−2	0.00	82.14
−1	7	13	20.73	78.84
−6	12	12	−20.73	82.67
−15	19	5	92.71	79.37
−4	6	11	−9.64	70.12
−5	7	0	24.56	47.81
27	−27	3	39.62	94.56
−11	11	5	888.77	109.48
26	−27	5	−24.17	78.84
10	−11	0	328.30	58.77
−5	4	9	29.05	71.97
−9	8	4	717.49	98.52
−16	28	1	−14.66	76.86
−18	27	6	−33.68	100.37
25	−17	−3	6.07	73.43
−16	22	7	54.15	101.55
37	−32	3	−47.81	80.95
−5	10	12	74.22	82.14
27	−24	0	−50.05	79.76
8	−5	−2	832.25	85.58
25	−25	2	23.11	69.86
−4	4	10	162.70	76.99
7	−8	0	651.85	72.77
−9	8	7	39.88	69.33
4	−6	10	−30.77	55.99
16	−20	6	48.20	48.99
15	−19	4	62.46	54.41
−12	32	−2	−54.15	84.78
−19	39	2	21.66	88.48
26	−7	−6	72.37	104.33
−14	32	−1	0.00	78.05
−8	25	−3	14.79	55.47
−20	36	4	0.00	73.03
5	10	−5	11.09	40.01
24	−10	−5	72.37	79.50
19	−6	−5	66.16	76.33
28	−16	−4	4.75	88.22
−19	31	4	72.50	106.97
−19	30	5	85.44	101.42
28	−21	−2	161.11	81.61
−15	22	2	28.53	69.33
38	−33	5	−0.13	82.93
−16	21	5	58.11	75.41
35	−31	3	18.22	76.86
11	−8	−2	496.55	73.03
29	−28	3	−0.66	96.54
−8	7	3	246.29	58.77
23	−25	6	55.20	71.31
13	−15	1	400.67	71.71
2	−6	8	−6.60	48.20
−18	41	1	0.00	82.01
−11	27	−2	−25.09	63.79

34	−19	−4	−50.18	100.37
32	−18	−4	83.07	111.33
30	−17	−4	25.88	115.03
27	−18	−3	−17.30	78.84
16	−7	−4	256.86	81.22
10	−1	−4	69.07	67.75
30	−22	−2	49.13	86.76
−17	24	4	66.29	77.92
−17	24	7	0.00	100.89
31	−26	0	−33.54	99.05
29	−25	0	−18.75	80.56
33	−30	3	39.88	79.76
22	−19	−1	141.04	73.43
31	−29	3	71.45	89.93
12	−11	−1	127.17	58.37
28	−28	5	−18.36	75.54
1	−1	11	7.40	54.81
18	−19	1	27.73	78.31
−10	9	5	70.52	80.16
18	−20	2	−34.07	65.77
−6	3	6	157.28	63.26
28	−8	−6	30.90	98.65
−18	38	1	−16.38	85.71
21	−3	−6	6.47	80.56
26	−11	−5	−9.90	86.50
−13	27	−1	64.18	67.35
−18	31	2	−28.13	83.33
−14	25	0	22.19	69.33
34	−24	−2	70.78	102.08
−19	29	6	−29.05	86.37
32	−23	−2	40.41	109.35
−17	25	3	49.65	85.84
37	−30	1	−70.39	82.67
37	−31	2	42.26	81.61
15	−9	−3	127.17	78.71
−17	23	5	−62.86	83.86
0	5	13	−13.34	74.22
−12	17	1	415.46	90.46
36	−32	5	0.00	82.67
−4	8	12	−32.09	77.26
−11	15	1	245.90	66.82
27	−25	1	24.17	76.07
1	1	12	2.38	67.62
27	−26	2	−12.41	77.92
−8	8	2	585.43	71.97
22	−24	4	4.89	63.65
22	−24	7	−0.26	55.20
−6	4	8	45.03	64.05
9	−14	5	19.28	28.79
30	−9	−6	0.00	96.67
−19	36	2	28.53	90.07
35	−22	−3	13.07	97.06
16	−3	−5	−4.09	67.62
13	0	−5	94.82	61.14

33	−21	−3	14.79	107.37
31	−20	−3	−13.60	102.35
29	−19	−3	51.50	84.65
13	−4	−4	239.82	79.10
−16	24	2	−9.90	84.25
35	−29	1	−10.56	91.25
−13	19	1	57.18	87.95
−13	19	10	−77.26	85.84
35	−30	2	−29.85	83.07
−12	17	10	15.05	83.33
−11	15	10	30.77	82.54
−13	17	9	31.96	80.56
34	−31	5	−62.99	83.99
29	−26	1	69.99	85.58
−10	13	1	117.80	62.86
−10	13	10	10.56	80.16
−12	15	9	71.31	84.65
29	−27	2	43.71	96.80
−14	16	6	4.49	79.90
30	−29	5	64.71	73.16
8	−7	12	45.43	60.09
6	−5	−1	766.35	83.73
−3	4	11	−27.20	69.07
1	−5	3	238.50	29.05
32	−10	−6	−79.24	83.07
−15	37	−1	16.90	85.05
18	0	−6	38.69	67.75
15	3	−6	17.30	64.05
−20	37	3	87.42	84.78
28	−12	−5	−57.18	106.84
−16	32	0	−79.10	82.27
−18	26	4	−61.67	78.44
−18	26	7	21.39	91.65
20	−13	−3	258.97	78.97
33	−28	1	197.56	95.35
−14	19	9	−13.87	84.65
33	−29	2	−20.07	87.16
31	−27	1	−24.43	102.08
31	−28	2	−31.43	99.18
−15	18	6	49.13	76.73
−11	13	9	3.96	82.93
20	−19	0	155.57	84.25
−13	14	6	−33.02	81.88
24	−24	9	16.77	50.98
−8	6	4	446.89	75.80
−8	6	7	155.83	68.67
2	−7	6	40.28	37.90
−17	40	0	51.50	79.24
30	−13	−5	45.56	105.12
−4	21	−4	180.13	44.90
−18	35	1	−26.41	101.16
−17	30	1	77.65	73.69
−20	33	4	29.05	106.97
−20	32	5	40.41	92.31

−18	25	5	−34.86	93.63
39	−33	6	7.92	79.76
−15	21	9	85.58	82.54
21	−16	−2	143.55	81.22
−10	15	11	47.94	80.29
24	−20	−1	−0.26	74.09
−16	20	6	0.00	77.52
17	−15	−1	391.43	86.24
−9	11	10	−13.21	78.44
9	−8	−1	55.07	49.65
−10	11	9	42.66	88.22
20	−22	3	87.29	68.80
−4	2	9	87.56	70.12
0	−3	9	149.89	66.69
−17	37	0	−19.02	89.14
23	−4	−6	−4.89	95.35
11	8	−6	27.60	55.99
32	−14	−5	−9.64	93.50
21	−7	−5	355.38	81.75
6	4	−4	154.64	54.41
−18	27	3	28.79	78.44
12	−6	−3	542.77	80.43
−15	19	8	34.34	90.07
−3	6	12	−12.81	68.67
−14	17	8	83.59	99.57
−13	15	8	12.15	98.52
5	−4	12	−29.85	61.80
−3	2	10	−4.36	66.69
1	−3	10	32.88	61.94
−9	7	5	90.73	81.09
19	−22	5	69.07	57.05
−15	27	0	17.83	72.37
−20	31	6	−2.25	82.93
−19	28	7	42.66	82.41
37	−32	6	50.71	76.60
−17	22	6	87.42	94.56
−9	11	1	592.56	74.48
−12	13	8	31.43	98.12
15	−15	0	241.01	73.95
23	−24	8	43.32	52.82
−3	−1	7	64.18	56.79
−4	0	5	1165.83	119.65
−20	41	2	0.00	78.58
−15	34	−1	28.26	95.22
−9	27	−3	10.83	55.60
−14	29	−1	47.81	70.39
−19	33	2	−63.52	107.10
−20	34	3	−56.26	107.76
36	−26	−1	−38.43	91.52
−19	28	4	−102.22	95.61
−19	27	5	−19.28	101.69
26	−21	−1	−70.39	87.95
−16	21	8	18.09	87.56
16	−12	−2	273.76	83.46

−8	9	10	56.65	76.73
−9	9	9	72.37	87.95
8	−9	11	−3.30	50.18
5	−6	11	−10.30	56.52
10	−14	3	311.93	58.50
−19	43	1	0.00	77.39
25	−5	−6	24.56	100.37
−12	29	−2	−1.06	62.99
9	5	−5	55.99	52.16
−8	18	−2	78.05	58.90
−7	16	−2	438.84	67.35
22	−14	−3	16.64	76.46
38	−32	4	13.34	81.09
38	−32	7	−47.01	75.27
−17	23	8	−34.07	84.52
−18	24	6	19.15	104.86
35	−31	6	−61.28	75.94
22	−20	0	101.42	78.31
20	−20	1	42.66	68.41
−11	11	8	321.17	97.99
24	−25	7	−29.05	60.48
20	−21	2	54.81	67.75
−7	5	3	392.22	61.67
−3	−1	4	485.72	62.99
−19	40	1	110.93	81.61
−20	38	2	7.66	82.01
4	12	−5	126.12	38.96
23	−8	−5	45.96	74.61
−19	29	3	−17.70	79.10
34	−25	−1	43.98	101.16
32	−24	−1	98.65	101.29
−10	18	−1	111.06	67.48
30	−23	−1	102.74	83.86
−9	16	−1	334.51	65.77
28	−22	−1	46.09	76.20
23	−17	−2	−32.35	77.12
26	−25	9	15.32	54.28
−2	2	11	14.66	65.50
10	−12	1	727.39	78.84
−5	2	8	45.96	61.80
27	−6	−6	−12.02	100.10
20	−1	−6	27.60	77.39
−17	34	0	−1.06	97.46
18	−4	−5	157.02	73.29
−18	32	1	30.77	86.24
−9	20	−2	4.75	66.43
17	−10	−3	253.69	85.71
−19	26	6	90.07	100.89
36	−31	4	15.72	83.59
33	−30	6	35.13	75.67
31	−29	6	−14.53	73.03
−2	4	12	−47.94	69.99
−7	9	11	111.20	80.95
29	−28	6	−101.82	74.61

2	−1	12	−20.87	63.13
26	−26	4	58.90	92.05
−7	4	7	216.71	70.39
−1	−3	8	64.18	55.20
29	−7	−6	1.45	85.31
14	5	−6	−21.79	62.33
−19	37	1	33.68	87.42
−20	30	4	−5.28	113.44
24	−15	−3	−48.33	86.37
−11	20	−1	286.31	79.50
−16	25	1	30.64	71.97
−20	29	5	−62.20	104.59
−6	14	−2	193.47	47.67
25	−18	−2	157.02	79.76
−8	14	−1	227.14	52.69
36	−31	7	0.00	71.58
−3	8	13	−12.28	79.24
34	−30	4	14.79	76.73
−16	19	7	47.01	88.35
−7	7	10	110.14	73.03
22	−23	3	35.52	65.37
−3	2	1	543.70	55.99
−7	6	2	216.98	51.24
−8	7	9	84.78	85.71
−10	9	8	2.91	82.93
−10	8	6	0.00	69.20
16	−19	8	0.00	38.03
15	−18	3	30.77	63.13
−5	1	6	182.51	60.75
31	−8	−6	8.58	84.78
10	10	−6	92.97	53.88
17	2	−6	40.28	67.35
25	−9	−5	58.50	78.44
−16	29	0	−5.68	72.11
35	−23	−2	−72.24	96.01
−17	21	7	135.63	103.40
32	−29	4	101.95	79.76
24	−21	0	108.95	76.20
19	−16	−1	260.69	86.24
28	−27	4	52.82	90.86
−8	9	1	51.37	50.58
−14	15	7	−94.42	87.16
26	−26	7	−12.81	60.09
25	−25	8	84.12	57.18
2	−3	11	20.73	59.56
4	−9	7	24.56	35.39
−5	23	−4	144.34	46.62
−20	35	2	71.45	96.93
−21	36	3	6.74	87.03
26	−16	−3	52.69	78.18
15	−5	−4	52.96	78.97
−12	22	−1	63.65	72.11
36	−27	0	22.98	85.84
27	−19	−2	50.32	79.63

38	−31	3	0.00	78.44
35	−30	8	18.22	70.52
34	−30	7	−13.73	68.67
−7	11	12	−5.94	75.41
−15	18	3	107.76	81.35
−16	19	4	271.12	84.25
30	−28	4	−39.22	84.39
28	−26	9	−38.03	58.11
−15	17	4	78.44	76.33
22	−21	1	102.22	72.63
−13	13	7	118.99	77.26
15	−16	1	186.47	75.14
17	−19	9	15.05	30.11
17	−20	4	67.35	56.52
10	−13	2	211.30	52.96
−7	4	4	1170.72	118.85
−8	5	5	201.66	80.16
−18	39	0	44.37	79.24
33	−13	−5	−61.28	83.07
22	−2	−6	43.84	91.39
27	−10	−5	38.56	104.33
34	−20	−3	0.00	94.42
15	−1	−5	−23.64	66.29
12	2	−5	143.95	61.01
−10	22	−2	40.54	56.39
33	−22	−2	0.00	105.91
28	−17	−3	−15.85	78.31
20	−9	−4	40.15	73.95
31	−21	−2	54.15	93.37
29	−20	−2	77.78	83.20
−20	28	6	−85.18	93.90
18	−13	−2	197.17	86.24
−18	23	7	−90.20	101.42
26	−22	0	−3.17	74.61
13	−9	−2	63.65	63.39
−16	20	3	260.16	78.31
−17	21	4	177.36	83.20
14	−12	−1	805.70	102.08
−14	16	3	56.79	88.74
−6	7	11	10.96	72.11
−14	15	4	71.84	78.71
22	−22	2	107.76	71.97
21	−23	5	1.58	66.95
−3	0	9	43.05	66.82
14	−18	5	121.89	49.52
0	−5	5	620.16	72.63
−16	36	−1	−76.60	95.48
31	−12	−5	33.54	94.16
29	−11	−5	−14.66	104.20
−13	31	−2	109.08	75.14
32	−19	−3	63.39	105.25
30	−18	−3	0.00	93.37
−19	30	2	0.00	76.60
−21	32	4	69.73	99.05

9	1	-4	48.73	61.28
-17	27	1	-39.88	75.14
-21	31	5	0.00	95.35
38	-30	2	15.85	76.07
34	-26	0	14.66	99.18
39	-32	5	13.60	77.78
36	-30	3	-33.54	77.78
-17	22	3	116.87	72.37
33	-29	8	25.75	65.24
-2	6	13	111.59	71.84
32	-29	7	62.86	69.20
30	-28	7	-1.72	68.28
28	-27	7	2.11	64.31
27	-26	8	4.23	57.84
17	-16	0	83.99	81.09
-13	14	3	207.86	90.59
24	-24	3	142.89	67.62
-13	13	4	-59.03	86.63
-12	11	7	58.37	71.71
18	-21	6	55.60	58.37
-1	-4	6	27.73	47.81
-10	29	-3	8.85	59.96
-21	40	2	-52.30	79.24
20	-5	-5	46.49	70.78
-19	34	1	58.11	98.52
12	-2	-4	126.12	72.90
36	-28	1	-3.57	86.24
32	-25	0	0.00	102.22
30	-24	0	6.34	85.97
-19	25	7	79.37	95.22
28	-23	0	15.45	75.54
-18	23	4	127.31	79.50
4	0	-2	3163.52	263.46
-6	9	12	-31.69	75.01
-17	20	5	89.93	81.35
-16	18	5	81.88	79.63
-15	16	5	38.69	75.94
12	-12	0	545.41	76.07
11	-12	11	23.51	36.45
-6	5	10	-24.70	69.20
7	-9	1	1130.84	103.27
-9	7	8	-47.28	76.73
1	-6	7	59.96	47.01
-20	42	1	37.64	82.01
24	-3	-6	-62.99	97.33
-18	36	0	0.00	95.61
-17	31	0	10.30	72.63
-13	24	-1	2.38	62.99
-21	30	6	-60.88	90.07
19	-11	-3	93.63	90.73
36	-29	2	-0.40	76.20
14	-7	-3	1298.69	136.15
-13	20	0	151.87	81.48
34	-29	3	0.00	78.31

−11	16	0	509.49	78.44
21	−17	−1	407.54	94.42
−14	18	10	−3.30	75.01
−18	22	5	−32.88	75.41
31	−28	8	−48.33	64.58
−13	16	10	−50.45	78.97
−15	18	9	24.04	87.82
29	−27	8	46.35	60.62
26	−25	3	12.81	74.48
−1	2	12	−3.96	61.80
−14	14	5	355.11	83.07
−7	5	9	−42.66	72.11
−9	6	6	99.18	65.24
12	−16	4	156.89	52.56
−20	39	1	76.20	79.24
−11	24	−2	51.37	56.79
22	−10	−4	167.72	83.46
34	−27	1	−64.97	87.82
−20	27	7	20.21	77.92
37	−31	5	−47.28	81.61
−18	24	3	−14.39	77.92
−19	25	4	12.68	76.86
32	−28	3	−24.83	84.65
−15	19	2	217.50	99.57
−16	20	9	−0.13	76.07
−14	17	2	748.52	112.52
28	−26	3	27.34	93.24
−12	14	10	37.51	80.03
−14	16	9	−7.40	76.99
24	−23	2	16.77	72.11
−12	12	3	803.33	110.14
−1	0	11	−1.58	62.73
26	−4	−6	1.06	94.95
9	12	−6	35.79	51.37
19	1	−6	−54.94	75.01
−21	37	2	89.54	85.31
−22	38	3	44.77	84.39
−21	33	3	112.52	103.80
−18	29	1	49.92	73.43
−22	33	5	79.37	80.56
−14	22	0	153.98	82.14
5	2	−3	327.38	50.71
−5	12	−2	227.81	41.20
34	−28	2	51.11	84.65
32	−26	1	−70.39	107.89
−16	21	2	150.29	78.44
−17	22	9	4.49	84.65
−19	24	5	−25.75	94.16
30	−27	3	18.49	93.90
−13	15	2	456.67	90.07
−4	5	0	773.48	75.94
−13	14	9	−9.64	76.46
−5	5	11	111.06	68.80
23	−24	5	−0.53	80.29

−12	11	4	41.86	85.58
−11	9	7	67.88	70.92
17	−20	7	64.71	60.48
32	−7	−6	−33.94	76.60
30	−6	−6	−24.83	77.12
28	−5	−6	50.45	87.56
13	7	−6	34.60	59.03
22	−6	−5	11.62	72.11
8	7	−5	80.16	51.90
−20	32	2	7.13	95.35
−22	34	4	−68.67	87.42
−19	26	3	68.67	74.61
20	−14	−2	98.91	94.03
32	−27	2	9.77	94.56
30	−25	1	79.10	87.16
10	−6	−2	1168.87	114.10
−10	14	0	1088.45	110.40
26	−24	2	26.68	75.14
−5	7	12	−33.28	68.80
−11	12	10	31.17	73.95
−13	12	5	36.32	71.58
4	−6	1	689.89	68.94
−4	0	8	249.33	64.31
−6	2	7	0.00	62.07
16	4	−6	12.55	67.75
−6	25	−4	−20.73	45.96
3	14	−5	97.86	28.92
−16	33	−1	96.01	87.03
24	−11	−4	15.32	72.50
−14	26	−1	30.51	68.67
35	−24	−1	25.22	99.57
17	−6	−4	70.39	74.09
−20	27	4	0.00	87.16
−17	23	2	−6.60	74.75
−20	26	5	−47.81	98.12
23	−18	−1	55.33	74.22
30	−26	2	49.65	96.93
28	−25	2	−1.85	78.71
−17	20	8	−16.90	83.07
−18	21	6	11.09	87.82
−16	18	8	−56.26	88.88
−17	19	6	98.12	83.20
−16	17	6	−34.73	74.35
−12	12	9	112.25	81.09
−19	41	0	−47.28	77.26
−20	36	1	0.00	93.63
17	−2	−5	27.07	70.65
26	−12	−4	19.15	71.18
−7	20	−3	129.68	50.71
−6	18	−3	29.71	53.22
21	−12	−3	1.32	76.46
−15	24	0	18.49	73.29
33	−29	5	−11.36	73.29
7	−3	−2	2230.91	192.15

−18	22	8	−37.51	86.76
19	−17	0	139.06	86.90
−12	13	2	194.13	74.75
−15	16	8	80.56	95.22
6	−6	12	−19.28	52.56
−15	15	6	145.27	79.24
19	−21	4	54.54	59.43
5	−8	10	0.00	47.67
−6	3	3	761.07	82.41
−8	5	8	31.56	63.79
21	0	−6	57.71	87.16
−17	38	−1	−8.72	80.03
−14	33	−2	4.89	88.08
24	−7	−5	116.08	74.22
−18	33	0	45.83	93.24
36	−22	−2	48.33	87.82
−12	26	−2	132.85	66.29
33	−23	−1	0.26	103.67
−20	28	3	−93.76	81.61
38	−31	6	15.05	71.58
−18	25	2	10.70	86.37
25	−19	−1	−29.45	79.90
−19	24	8	−41.34	74.88
−19	23	6	17.17	96.40
11	−9	−1	720.00	87.29
25	−25	5	−40.94	87.69
20	−20	10	10.96	31.43
17	−17	1	289.61	83.99
3	−3	12	−7.66	58.90
−7	7	1	934.99	95.22
−10	10	10	45.30	73.43
−14	14	8	107.50	96.40
−11	10	3	667.83	90.46
17	−19	3	26.15	60.75
−5	3	10	−38.56	68.28
2	−5	10	−7.92	54.01
1	−5	9	−24.83	55.99
32	−11	−5	69.99	88.35
−11	31	−3	−7.26	57.58
−19	38	0	−28.92	83.99
26	−8	−5	32.88	82.27
35	−19	−3	−69.07	93.37
−22	35	3	51.90	86.37
−19	31	1	18.36	76.60
5	6	−4	238.50	55.60
31	−22	−1	53.88	86.63
29	−21	−1	29.19	75.01
1	7	−3	120.57	23.51
−21	29	4	−41.34	98.52
27	−20	−1	−26.28	83.99
22	−15	−2	97.06	75.01
−21	28	5	79.63	104.72
15	−10	−2	464.59	88.48
−20	25	6	22.45	95.74

31	−28	5	−1.85	68.94
16	−13	−1	237.71	81.22
29	−27	5	11.75	74.75
27	−26	5	−50.45	78.44
−11	10	9	53.09	82.67
−14	13	6	−25.75	71.97
20	−22	6	−3.17	73.03
−11	9	4	798.04	108.29
−12	10	5	186.73	77.12
−2	−3	7	94.03	54.28
30	−10	−5	51.11	107.37
28	−9	−5	−45.69	101.95
−22	39	2	−42.39	77.26
14	1	−5	40.15	63.52
−8	22	−3	11.23	51.37
34	−21	−2	−36.58	101.82
−21	34	2	−21.26	99.97
37	−26	0	11.23	84.92
23	−13	−3	35.00	74.48
−16	26	0	117.67	75.67
37	−30	4	20.60	76.46
−4	5	12	−26.41	63.13
−9	10	11	0.66	74.22
−6	3	9	32.09	66.56
−10	7	7	327.38	77.26
23	−1	−6	131.66	96.14
−21	41	1	0.00	79.10
33	−18	−3	17.30	102.08
11	4	−5	87.16	59.82
−15	28	−1	35.39	68.54
−5	16	−3	120.44	43.18
39	−31	7	−3.43	68.80
24	−16	−2	6.07	75.94
16	−8	−3	228.99	91.12
−21	27	6	−48.73	90.33
−9	12	0	416.12	70.12
−9	12	12	9.77	71.84
−4	3	11	32.22	63.79
−13	12	8	76.46	93.10
−6	4	2	152.13	48.60
−8	4	6	60.62	64.05
19	−3	−5	120.44	73.03
31	−17	−3	114.89	103.40
32	−20	−2	0.00	98.78
19	−7	−4	167.59	73.82
25	−14	−3	37.11	78.84
14	−3	−4	232.30	83.46
−21	30	3	0.00	103.67
−22	30	5	107.63	92.84
36	−30	6	−48.86	73.56
9	−9	0	1140.88	109.08
0	0	12	82.14	60.88
−11	11	2	534.72	80.03
−9	8	10	59.30	70.52

7	−10	2	3658.62	302.55
−1	−2	10	69.73	59.16
−6	2	4	140.64	59.56
−3	−2	5	494.44	72.77
−4	−1	6	63.39	55.86
31	−5	−6	−40.67	74.75
25	−2	−6	15.19	91.25
18	3	−6	12.81	74.09
12	9	−6	−22.71	53.88
−17	35	−1	0.00	93.63
−13	28	−2	17.83	62.60
29	−16	−3	−7.40	82.54
27	−15	−3	41.34	74.61
30	−19	−2	−17.43	83.73
35	−25	0	−44.77	92.05
28	−18	−2	60.35	78.31
26	−17	−2	60.62	84.52
−22	31	4	−41.20	102.48
35	−29	4	30.24	74.88
−15	20	1	238.37	89.80
−6	10	−1	1517.25	133.12
−14	18	1	761.20	113.70
−18	20	7	−3.30	95.74
21	−22	4	−21.79	62.20
12	−13	1	232.82	58.77
13	−17	6	71.05	34.07
−2	−2	9	100.37	57.71
29	−4	−6	−46.75	80.29
27	−3	−6	38.96	81.35
−21	38	1	16.11	79.90
−19	35	0	−5.41	92.84
−9	24	−3	27.20	50.84
−23	37	3	155.70	82.80
−20	33	1	6.07	95.61
−17	28	0	55.33	72.37
37	−27	1	−19.55	76.46
−20	29	2	0.00	74.09
37	−29	3	68.54	75.80
−22	29	6	98.39	85.84
−4	7	13	−59.43	65.63
−13	16	1	240.48	79.90
−19	22	7	136.68	95.35
19	−18	1	28.92	86.50
14	−13	0	194.13	63.52
−8	8	11	−76.46	73.16
−16	16	7	38.03	87.82
3	−5	11	59.03	46.22
−10	8	9	18.22	82.93
−12	10	8	137.74	90.59
15	6	−6	5.28	60.22
35	−15	−4	−4.23	85.18
−22	36	2	1.19	82.27
33	−24	0	9.77	99.31
37	−30	7	43.84	67.09

34	−29	6	28.00	69.73
23	−19	0	291.46	80.69
18	−14	−1	420.48	96.40
−20	24	7	−54.41	86.24
−8	10	12	−20.73	71.45
22	−23	6	96.27	67.09
19	−20	3	53.35	62.73
−10	8	3	166.66	69.73
16	−19	5	88.48	47.01
−11	8	5	185.15	80.95
−2	−2	3	651.72	64.45
−15	35	−2	−60.22	83.73
21	−4	−5	197.04	75.27
−16	30	−1	177.62	70.26
21	−8	−4	140.91	82.54
37	−28	2	0.00	77.78
−23	32	5	−19.41	81.48
31	−23	0	5.41	89.41
33	−28	4	−0.53	74.48
−21	26	7	−70.65	79.90
−15	14	7	31.69	79.50
0	−2	11	44.90	47.28
12	−15	3	50.98	49.39
7	−11	9	21.26	28.00
−7	3	8	185.81	63.13
0	−5	8	−20.60	41.47
33	−10	−5	27.60	73.43
20	2	−6	1.85	71.58
−20	40	0	−5.81	68.94
33	−14	−4	21.13	90.20
36	−23	−1	−45.69	80.56
11	0	−4	455.74	80.29
8	3	−4	29.05	52.30
−23	33	4	14.53	85.05
35	−26	1	0.00	79.10
18	−9	−3	9.64	79.63
−18	26	1	36.98	66.95
35	−28	3	−14.00	70.52
29	−22	0	8.32	72.37
27	−21	0	58.37	69.86
17	−11	−2	246.43	90.59
25	−20	0	48.33	77.92
32	−28	6	−106.84	61.80
−18	21	9	51.64	68.41
8	−6	−1	1271.09	119.91
−15	17	10	46.22	68.01
23	−23	4	50.05	62.33
19	−19	2	103.93	53.48
18	−20	8	−18.49	34.73
6	−8	11	−9.24	35.66
−10	7	4	1523.19	149.36
−9	5	7	62.60	55.99
3	−8	8	7.53	32.49
−22	43	1	−21.79	64.05

−10	26	−3	80.29	42.13
−14	30	−2	141.97	54.81
23	−9	−4	135.23	73.82
−18	30	0	84.25	61.41
−21	31	2	−7.92	75.14
38	−30	5	95.88	66.29
35	−29	7	39.09	49.79
31	−27	4	8.72	66.69
−19	22	4	151.74	74.61
−12	14	1	621.35	83.99
−17	19	9	−12.02	73.56
−18	20	4	50.84	70.92
−14	15	10	−12.15	62.07
−16	17	9	25.62	63.65
−17	18	4	−30.51	63.52
24	−24	6	43.05	54.67
−3	3	12	84.12	48.60
12	−14	2	114.76	46.22
−8	6	10	−37.11	58.50
8	−11	10	110.80	26.41
−5	0	7	132.99	47.15
31	−9	−5	21.79	66.95
31	−13	−4	−45.83	88.22
23	−5	−5	88.35	56.39
16	0	−5	238.37	57.97
7	9	−5	115.16	38.43
−21	35	1	84.78	73.82
35	−27	2	−21.39	74.09
33	−25	1	38.83	80.69
36	−29	8	−28.26	50.58
12	−7	−2	234.14	50.98
−20	24	4	47.67	55.86
30	−27	6	−75.80	52.03
29	−26	4	81.48	65.90
27	−25	4	44.24	68.28
21	−19	1	−2.51	58.11
−19	21	5	−7.53	56.26
25	−24	4	20.07	55.20
−7	8	12	−28.92	51.64
−18	19	5	5.68	57.05
−14	12	7	50.84	54.67
−2	−1	2	460.63	48.07
32	−4	−6	−87.42	50.45
29	−8	−5	268.74	73.56
27	−7	−5	−189.64	77.39
25	−6	−5	−46.35	54.81
−18	37	−1	−6.07	61.67
−22	40	1	25.88	47.81
29	−12	−4	42.79	69.99
27	−11	−4	−79.10	52.69
25	−10	−4	33.02	55.86
34	−22	−1	125.72	77.65
16	−4	−4	124.27	50.84
−23	34	3	−88.61	60.35

```
 -19  28   1    7.66   53.09
  13  -5  -3  499.06   72.63
  33 -27   3  227.81   59.30
  20 -15  -1  200.07   60.22
 -19  23   3   45.30   51.11
 -18  21   3   76.20   59.69
 -20  23   5  -19.41   59.43
   2   0  -1 1044.47   91.91
  -3   5  13    0.00   39.35
  -8  10   0   11.89   31.56
 -17  19   3    0.92   52.69
  26 -25   6   98.25   47.94
  22 -21  10  -35.52   29.71
  21 -21   3  461.69   64.18
 -13  13  10  -25.22   49.79
 -15  15   9   47.01   55.86
 -16  16   4  399.75   70.12
 -17  17   5  441.74   68.67
  19 -20   9   -0.13   26.54
  -7   6  11 -194.92   48.86
 -10   9   2   61.14   47.67
  -9   6   9   17.43   45.03
 -11   8   8   -8.19   61.28
  -5   1   9   14.79   42.26
  22   1  -6   -5.94  139.46
 -20  37   0   65.11  141.70
 -23  38   2  -25.88  122.95
 -21  26   4  389.05  169.57
  -3   1  11   29.32  113.04
  14 -17   4  283.67   95.35
  -3  -2   8  192.94   98.25
   2  16  -5  103.01   49.26
   0   0   0    0.00    0.00


_computing_structure_solution     'SHELXT 2014/4 (Sheldrick, 2014)'
_computing_structure_solution     'SHELXT 2014/4 (Sheldrick, 2014)'
_computing_structure_solution     'SHELXT 2018/2 (Sheldrick, 2018)'
_computing_structure_solution     'SHELXT 2018/2 (Sheldrick, 2018)'
;
_shelx_hkl_checksum                5548
_olex2_submission_special_instructions  'No special instructions
were received'
```
